# Investigating the anticancer potential of phytochemicals from the mangrove *Rhizophora mucronata* as inhibitors of lung cancer-associated proteins: Insights from multiscale computational and in silico analyses

**DOI:** 10.1371/journal.pone.0359525

**Published:** 2026-10-07

**Authors:** Zena Abdul-Ameer Mahdi, Batool Shakir Abed AlmjalawI, Mollah Naimuzzaman, Md Mahabub Hasan, Ajoy Kumer, Farah Tareq Al-Alaq, Hussein O.M. Al-Dahmoshi, Faizur Rahman, Md. Fayez Al-Hakim Khan, Noor S.K. Al-Khafaji, Sarah Alwan Malik Al-Yasiri

**Affiliations:** 1 Department of Medical Physics, College of Applied Medical Sciences, University of Kerbala, Karbala, Iraq; 2 Ministry of Education, General Directorate of Education in Kerbala, Iraq; 3 Department of Agroforestry and Environmental Science, Sylhet Agricultural University, Sylhet, Bangladesh; 4 College of Agricultural Sciences, IUBAT—International University of Business Agriculture and Technology, Dhaka, Bangladesh; 5 Department of General Medicine, Saveetha Institute of Medical and Technical Sciences in Saveetha Medical College and Hospital, Chennai, India; 6 College of Arts and Sciences, Department of Chemistry, IUBAT—International University of Business Agriculture and Technology, Dhaka, Bangladesh; 7 Department of Biology, College of Science, University of Babylon, Babylon, Iraq; 8 Department of Environmental Health, College of Applied Medical Sciences, University of Kerbala, Karbala, Iraq; TIU: Tishk International University, IRAQ

## Abstract

Lung cancer remains the leading cause of cancer-related mortality worldwide, with a five-year survival rate of approximately 20–25% despite advances in targeted therapy and immunotherapy. There is a continuous need for novel, low-toxicity compounds capable of modulating key oncogenic pathways in non-small cell lung cancer (NSCLC). Mangrove ecosystems are rich reservoirs of bioactive phytochemicals, yet the inhibitory potential of compounds from *Rhizophora mucronata* against validated lung cancer-associated proteins has not been systematically explored using multi-scale computational approaches. This study employed an integrated computational pipeline to evaluate 14 phytochemicals from R. mucronata. Density Functional Theory (DFT) calculations at the B3LYP/6-31G* level were performed to characterize electronic properties of the ligands. Molecular docking studies were conducted using both AutoDock Vina (PyRx) and Schrödinger Glide against the Keap1 Kelch domain (PDB 1X2J) and the YEATS2 YEATS domain (PDB 5XNV). Top-ranked complexes were subjected to 100-ns molecular dynamics (MD) simulations in Desmond, and pharmacokinetic and toxicity profiles were predicted using SwissADME and pkCSM. Among the screened compounds, ligand L10 (cyclohexane-1,2,3,4,5,6-hexaol) exhibited the smallest HOMO–LUMO energy gap (0.36 eV), favorable binding affinities (Glide score of –7.789 kcal/mol against 1X2J), multiple hydrogen-bonding interactions, and stable complex behavior during MD simulations (Cα RMSD < 3 Å). Several other ligands (L02, L05, and L07) also displayed promising profiles. Most compounds complied with Lipinski’s rule of five and showed acceptable predicted ADMET properties. These preliminary findings suggest that *Rhizophora mucronate* -derived phytochemicals, particularly L10, are promising candidates for further experimental evaluation as modulators of Keap1 and YEATS2 in lung cancer. This study provides a computational framework for prioritizing natural product leads from mangrove species for subsequent in vitro and in vivo validation.

## 1. Introduction

Lung cancer is one of the top 3 causes of cancer mortality globally, causing nearly 1.8 million deaths a year and representing a significant burden on global health [1]. Even with significant progress in targeted therapy and immunotherapy, the overall five-year survival is still modest, mainly due to patients being diagnosed at advanced stages [2]. The five-year relative survival rate is still only ~25% for all stages, highlighting a continued unmet need in finding safe and effective therapeutic agents [3]. The pathogenesis of lung cancer is affected by diverse modifiable and environmental risk factors [4,5]. Tobacco smoking persists the leading aetiological factor, with passive smoking also playing an important role in disease causation [6–8]. These include environmental exposures such as ambient air pollution, household air pollution, and radon, as well as occupational carcinogens and genetic susceptibility [9–11].

Lung cancer is asymptomatic in most cases during the early stages and hence detected at advanced disease state when curative treatment options become scarce [12]. Management for current treatment approaches consists of surgical options, radiotherapy, chemotherapy, molecularly-targeted therapy and immunotherapy. While these strategies have improved clinical outcomes, their utility can be limited by treatment-induced toxicity, pan-tumor drug resistance and recurrence, biomarker-restrictive accessibility as well as high costs [13–16]. These drawbacks are driving the identification of less toxic and more efficacious therapeutic candidates.

Natural-product-based phytochemicals have been used as the primary source of structurally diverse scaffolds for anticancer compound discovery [17]. Ecosystems of mangroves are notably significant because they constitute, as yet underexplored, a true reservoir of bioactive secondary metabolites: viz. flavonoids, tannins, terpenoids and alkaloids with reported antioxidant, anti-inflammatory and cytotoxic activity [18]. Lung cancer is generally divided into small-cell lung cancer and non-small-cell lung cancer (NSCLC). Non-small-lung- cell carcinoma accounts for about 85% of these cases [19].

Traditional medicinal practice uses mangrove species, which are known to contain phytochemicals with a wide range of biological activities including anticancer [20], antibacterial properties (p-glyoproteins in *Rhizophora mucronata* and plant extracts based natural product) antioxidant, anti-inflammatory property. It contains many polyphenols, alkaloids, and other secondary metabolites that may have a pharmacological importance [21,22]. Antitumor activity of *R. mucronata* extracts was found in various cancer-cell models, such as lung carcinoma cells [18,23]. Thus, *R. mucronata* appears not only as a source of active cysteine proteinase inhibitors, but other biological activities have also been reported from the extracts and from isolated constituents that warrant future investigation of this species for potential anticancer compounds [24]. Nevertheless, no systematic all-in-one identification of R. mucronata phytochemicals acting as protein-selective modulators against lung cancer-related targets has been illustrated through an integrated method in silico studies till date.

Thus, we selected two mechanistically relevant NSCLC-associated proteins as molecular targets: the Kelch domain of Keap1 (PDB ID: 1X2J) and the YEATS domain of YEATS2 (PDB ID: 5XNV). Keap1 is a major negative regulator of Nrf2 and the Keap1–Nrf2 pathway is often abrogated in NSCLC. Mutations of Keap1 induce persistent Nrf2 activation, which can lead to antioxidant protection, metabolic adaptation, cell survival and more importantly anticancer treatment resistance [25,26]. Thus, targeting Keap1–Nrf2 offers a biologically relevant approach for probing redox-dependent mechanisms of NSCLC progression. Histone acetylation is associated with transcriptional activation. In lung cancer, amplification or overexpression of YEATS2 is linked to the progression of NSCLC via oncogenic transcriptional programs associated with tumor-cell proliferation and survival [27]. Hence, targeting the YEATS2 YEATS domain may allow a strategy to modulate aberrant acetylation-dependent transcriptional signaling. Thus, Keap1 and YEATS2 were chosen as composite molecular targets indicative of redox-regulatory and epigenetic mechanisms associated with NSCLC pathogenesis.

Here, we provide a systematic evaluation of phytochemicals from *R. mucronata* against Keap1 and YEATS2 to test our hypothesis that they could act as modulators of these proteins, representing an innovation in overcoming this identified knowledge gap via an integrated computational workflow. We used Density Functional Theory (DFT), molecular docking through AutoDock Vina/PyRx and Schrödinger Glide, ADMET profiling and 100-ns molecular dynamics simulations to provide the electronic properties, predicted binding interactions, pharmacokinetic behavior and complex stability. This multiscale approach was therefore carried out to rank potential phytochemicals for further in vitro validation instead of deriving conclusive biological activity directly from hundreds of thousands of predicted computationally.

## 2. Methodology

### 2.1 Optimization and ligand preparation

The 14 phytochemicals from *R. mucronata* were systematically selected based on a comprehensive literature review of its reported secondary metabolites, prioritizing chemical diversity across multiple classes including nitrogen-containing heteroaromatics (L01, L02, L05, L014), polyethers/siloxanes (L03, L13), long-chain esters/fatty acids (L04, L06, L09), polyhydroxy carbohydrates/sugar derivatives (L07, L08, L10), steroidal alcohols (L011), and aromatic oximes/naphthalene derivatives (L012) to (L014) cover a broad range of polarity, aromaticity, hydrogen-bonding capacity, and lipophilicity that could enable diverse interactions with target proteins. The Universal Force Field (UFF) was used for geometry pre-optimization to fix bond lengths and angles that were too long or too short, get rid of steric clashes, and get reasonable starting conformations. The Build Parameters module of Materials Studio was then used to make parameter files that included all the necessary force-field terms, atom types, and partial charges for the next quantum-mechanical calculations.

The DMol^3^ module in Materials Studio was employed to perform the density functional theory calculations using the computational level specified for this study. The molecular structures were initially subjected to geometry pre-optimization using the Universal Force Field (UFF) to remove unfavorable geometries and steric clashes. Solvent effects were incorporated using the COSMO model where applicable. Geometry optimization was continued until the specified convergence criteria for total energy and nuclear coordinates were satisfied. The optimized structures were subsequently used to calculate the frontier molecular orbital energies and global chemical-reactivity descriptors, including the HOMO–LUMO energy gap, ionization potential, electron affinity, chemical potential, electronegativity, hardness, softness, and electrophilicity index. The optimized structures were first checked for thermodynamic and structural stability by examining their geometries, bond parameters, and vibrational frequencies. From these stable structures, frontier molecular orbital properties and global reactivity descriptors were obtained, including HOMO and LUMO energies, HOMO–LUMO gap (E_gap), ionization potential (I), electron affinity (A), chemical potential (μ), electronegativity (χ), hardness (η), softness (s), and electrophilicity index (ω) [28–30], which are shown in Equations 1–8.


$$\begin{array}{c} {\text{E}}_{\text{gap}}=\;({\text{E}}_{\text{LUMO}}\;-\;{\text{E}}_{\text{HOMO}}) \end{array}$$ (1)

$$\begin{array}{c} \text{I}=\;-\;{\text{E}}_{\text{HOMO}} \end{array}$$ (2)

$$\begin{array}{c} \text{A }=\;-{\text{E}}_{\text{LUMO}} \end{array}$$ (3)

$$\begin{array}{c} \;\left(X\right)=\;\frac{I+A}{\text{2}} \end{array}$$ (4)

$$\begin{array}{c} \left(\omega \right)=\frac{\mu^{\text{2}}}{\text{2}\eta } \end{array}$$ (5)

$$\begin{array}{c} (\mu )=-\frac{I+A}{\text{2}} \end{array}$$ (6)

$$\begin{array}{c} (\eta )=\frac{I-A}{\text{2}} \end{array}$$ (7)

$$\begin{array}{c} (S)=\frac{\text{1}}{\eta } \end{array}$$ (8)

### 2.2 Protein preparation and collection

Used the Keap1 Kelch domain (PDB 1X2J) [31] and the YEATS2 YEATS domain (PDB 5XNV) [32] were selected as molecular docking targets because of their biological relevance to non-small cell lung cancer (NSCLC). Keap1 regulates Nrf2 turnover, and dysregulation of the Keap1–Nrf2 pathway contributes to persistent antioxidant signaling, metabolic adaptation, and chemoresistance in NSCLC. YEATS2 functions as an epigenetic reader of histone H3K27 acetylation (H3K27ac) and is frequently amplified or overexpressed in NSCLC, where it promotes oncogenic transcriptional programs associated with tumor progression.

The crystal structures were retrieved from the Protein Data Bank. The selected structures had resolutions of 1.60 Å for 1X2J and 2.70 Å for 5XNV, with more than 96% of residues located in favored regions of their respective Ramachandran plots (Table 1). Receptor preparation was performed independently for the AutoDock Vina and Glide workflows. For AutoDock Vina docking, the structures were prepared using Discovery Studio by removing non-essential crystallographic ligands, water molecules, and ions, followed by energy minimization.

**Table 1:** Protein information.

Title	PDB ID: 1X2J	PDB ID: 5XNV
Organism	*Homo sapiens*	*Homo sapiens*
Resolution	1.60 Å	2.70 Å
R-Value Free	0.200	0.266
Ramachandran plot, %	96.18%	96.92%
References	[31]	[32]

For Glide docking, the receptor structures were independently processed using the Protein Preparation Wizard in Schrödinger Maestro. The preparation workflow included assignment of bond orders, addition of hydrogen atoms, optimization of the hydrogen-bonding network, removal of unnecessary water molecules, and restrained energy minimization. The resulting prepared receptor structures were subsequently used for receptor-grid generation and Glide Standard Precision (SP) docking.

### 2.3 Molecular docking and visualization of docking

#### 2.3.1 Molecular docking by PyRx.

Molecular docking was performed using PyRx 0.8 with the AutoDock Vina engine to predict the binding affinities and preferred binding poses of the 14 phytochemicals against the two target proteins. Prepared receptor and ligand structures were imported separately into PyRx. For each receptor, a rectangular docking grid was defined using the center coordinates and dimensions reported in Table 2 to encompass the biologically relevant binding region. The conformational search was conducted using an exhaustiveness value of 8 and nine output binding modes. The resulting poses were ranked according to the AutoDock Vina docking score, expressed in kcal/mol. Protein–ligand complexes were subsequently visualized and analyzed in Discovery Studio for hydrogen bonding, hydrophobic interactions, π interactions, and other non-covalent contacts. This methodical approach enabled reliable identification of the most satisfactory binding modes and quantitative comparison of ligand affinities for both 1X2J and 5XNV [33].

**Table 2:** Grid box parameters used for docking analysis in this study.

Protein Name with PDB ID	Grid Box Size (PYRX)		Grid Box Size (Glide)	
	Center	Dimension(Å)	Center	Dimension(Å)
*Homo sapiens* (PDB 1X2J)	X = 22.67	X = 55.81	X = 20	X = 30
	Y = 61.39	Y = 44.93	Y = 60	Y = 20
	Z = 37.82	Z = 49.73	Z = 30	Z = 30
*Homo sapiens* (PDB 5XNV)	X = −14.77	X = 36.44	X = −10	X = 30
	Y = −31.53	Y = 48.80	Y = −30	Y = 30
	Z = −18.45	Z = 59.16	Z = −20	Z = 30

#### 2.3.2 Molecular docking by Glide from Schrodinger suite.

Molecular docking was performed using the Glide module of the Schrödinger Suite (Release 2024−1) to provide an independent evaluation of ligand binding to Keap1 (PDB ID: 1X2J) and YEATS2 (PDB ID: 5XNV). Ligands were prepared using LigPrep to generate appropriate three-dimensional structures, ionization states, and tautomers under the selected preparation conditions. Receptor grids were generated around the defined binding regions using the Receptor Grid Generation module, with the grid centers and dimensions provided in Table 2 [34], to encompass the entire biologically relevant pocket while maintaining computational efficiency.

Docking was subsequently performed using Standard Precision (SP) mode, and the generated poses were ranked using GlideScore, which accounts for van der Waals, Coulomb, lipophilic, hydrogen-bonding, metal-binding, and desolvation terms, as well as penalties for buried polar groups and rotatable-bond freezing equation [9].


$$\begin{array}{c} G\;Score=a\times vdW+b\times coul+Lipo+HBond+Metal+BuryP+RotB+Site \end{array}$$ (9)

Here, vdW is the van der Waals term, Coul is the Coulombic (electrostatic) term, Lipo represents lipophilic contacts, HBond corresponds to hydrogen‑bonding interactions, Metal captures metal‑binding contributions, BuryP is the penalty for buried polar groups, RotB is the penalty for freezing rotatable bonds, and Site represents polar interactions within the active site; the coefficients for vdW and Coul are a = 0.065 and b = 0.130, respectively.

### 2.4 Molecular dynamic

To assess the stability of the most promising protein–ligand complexes under physiological conditions, 100‑ns all‑atom MD simulations were carried out for the top‑ranked poses 1X2J–L10 and 5XNV–L02 using Desmond (Schrödinger Release 2024‑1; D. E. Shaw Research, New York, NY, USA). Each system was parameterized with the OPLS‑AA force field, placed in an orthorhombic periodic box of TIP3P water with a 10 Å buffer, and electrically neutralized by adding Na⁺ or Cl^-^ counter‑ions. A standard multi-stage equilibration protocol was applied: steepest-descent energy minimization, gradual heating to 300 K, and restrained equilibration (NVT followed by NPT) to relax the solvent while gradually releasing restraints on the solute. Production MD runs were conducted in the NPT ensemble (300 K, 1.01325 bar) employing the Nose-Hoover thermostat and Martyna-Tuckerman-Klein barostat, with a 2-fs integration time step and SHAKE constraints on all bonds involving hydrogen atoms. Trajectory files and energy logs were analyzed in Maestro and the Simulation Event Analysis module of Desmond. Main constancy metrics including root-mean-square deviation (RMSD) of the protein Cα atoms, root-mean-square fluctuation (RMSF) per residue, radius of gyration (R_g), and persistent protein–ligand contacts (hydrogen bonds, hydrophobic interactions, and water bridges) were calculated over the entire 100-ns trajectory [35]. This inclusive computational investigation followed conventional best practices and parameters, enabling a thorough exploration of the molecular system’s dynamic behavior.

### 2.5 Lipinski rule, drug-likeness and pharmacokinetics

On October 9, 2025, SwissADME, a common online platform and its famous for flexibility and convenience, was attached to predict pharmacokinetics and evaluate drug-like assets [36]. In this step, the online SwissADME platform (http://www.swissadme.ch/index.php) was used to compute key pharmacokinetic properties and assess drug-like features of the ligands. Core descriptors, including molecular weight, number of rotatable bonds (NRB), lipophilicity, and counts of hydrogen bond donors (HBD) and acceptors (HBA), were systematically evaluated to characterize their prospective pharmacological profiles.

### 2.6 Absorption, distribution, metabolism, excretion, and toxicity (ADMET) properties

The absorption, distribution, metabolism, excretion, and toxicity (ADMET) properties, which are key factors in drug development, were evaluated in silico [37,38]. ADMET descriptors were obtained using the SwissADME and pkCSM online platformshttp://biosig.unimelb.edu.au/pkcsm/prediction_single/adme_1643650057.59 (accessed from the 10 October 2025) [39].

## 3. Results and discussionresults and discussion

### 3.1 Quantum calculation by DFT

#### 3.1.1 Chemistry and optimized structure of phytochemicals from mangrove tree, R. mucronata.

The selected 14 compounds, listed and coded with their chemical name shown in below and S1 Fig, from *R. mucronata* cover a wide chemical space which are chemically six type of compounds, such as Nitrogen heteroaromatics (L01, L02, L05, L014), Polyethers and siloxanes (L03, L13), Long-chain esters and fatty acids (L04, L06, L09), L06, Methyl The 14 phytochemicals selected from the mangrove species *R. mucronata* (structures provided in S1 Fig) encompass a broad and diverse chemical space. These compounds can be categorized into six distinct structural classes: nitrogen heteroaromatics (L01, L02, L05, L14), polyethers and siloxanes (L03, L13), long-chain esters and fatty acids (L04, L06, L09), polyhydroxy carbohydrates and sugar-like derivatives (L07, L08, L10), a steroidal alcohol (L11), and an aromatic oxime/naphthalene derivative (L12). This structural diversity translates into a wide array of potential protein–ligand interactions. Specifically, the polar polyhydroxy and sugar-like ligands (L07, L08, L10) are primed for strong hydrogen bonding and specific molecular recognition. The aromatic nitrogen heterocycles and fused ring systems (L01, L02, L05, L12, L14) facilitate π–π stacking, hydrogen bonding, and potential metal coordination. Hydrophobic lipids and steroids (L04, L06, L09, L11) are well-suited for interacting with nonpolar protein regions and cellular membranes. Finally, the siloxane-type scaffolds (L03, L13) provide flexible, lipophilic, and weakly polar backbones. Collectively, the variance in aromaticity, hydrogen-bonding capacity, lipophilicity, and conformational flexibility supports a broad spectrum of possible biological interactions. This chemical diversity aligns with existing evidence that *R. mucronata* extracts are rich in phenols, fatty acids, terpenoids, alkaloids, and other secondary metabolites possessing notable antioxidant and anticancer potential.

The chemical name of selected 14 phytochemicals used in our study which are extracted from the from *Rhizophora mucronate*, as mangrove tree in our mangrove forest, is as 4-chloro-3-methoxy-2-methylpyridine (**L01)**, benzo[h]quinoline-4-carboxylic acid **(L02),** 2,2,4,4,6,6,8,8,10,10,12,12,14,14-tetradecamethyl-1,3,5,7,9,11,13-heptaoxacyclotetradecane **(L03),** Methyl 14-(2-octylcyclopropyl)tetradecanoate **(L04)**, N-(3-fluorobenzyl)-7H-purin-6-amine **(L05),** Methyl 14-methylpentadecanoate **(L06),** (E)-3,4,5-trihydroxy-6-(hydroxymethyl)tetrahydro-2H-pyran-2-yl N-hydroxybut-3-enimidothioate **(L07),** (2R,3S,4R,5R)-2,4,5,6-tetrahydroxy-3-methoxyhexanal **(L08),** Octadeca-9,12-dienoic acid **(L09),** Cyclohexane-1,2,3,4,5,6-hexaol **(L10),** ((8S,9S,10S,13R,14S,17R)-13-methyl-17-((R)-6-methylheptan-2-yl)-2,3,4,7,8,9,10,11,12,13,14,15,16,17-tetradecahydro-1Hcyclopenta[a]phenanthren-10-yl)methanol **(L011),** (E)-5,6-dimethoxy-3,4-dihydronaphthalen-1(2H)-one oxime **(L012),** 2,2,4,4,6,6,8,8,10-nonamethyl-3,5,7,9-tetraoxaundecane **(L13),** 1,4,5,8-tetraphenyl-1H-thiopyrano[4,3-c]pyridine **(L014).**

In applying quantum‑chemical methods to these compounds, careful optimization of molecular geometry was essential to obtain reliable structural descriptions. The study focused on locating the lowest‑energy conformation for each molecule to set robust computational parameters [40]. Using a DFT functional, all structures were energy‑minimized, yielding their predominant, thermodynamically favored configurations with minimal calculated energy. The ligands are illustrated in Fig 1 and S1 Fig.

**Fig 1:**
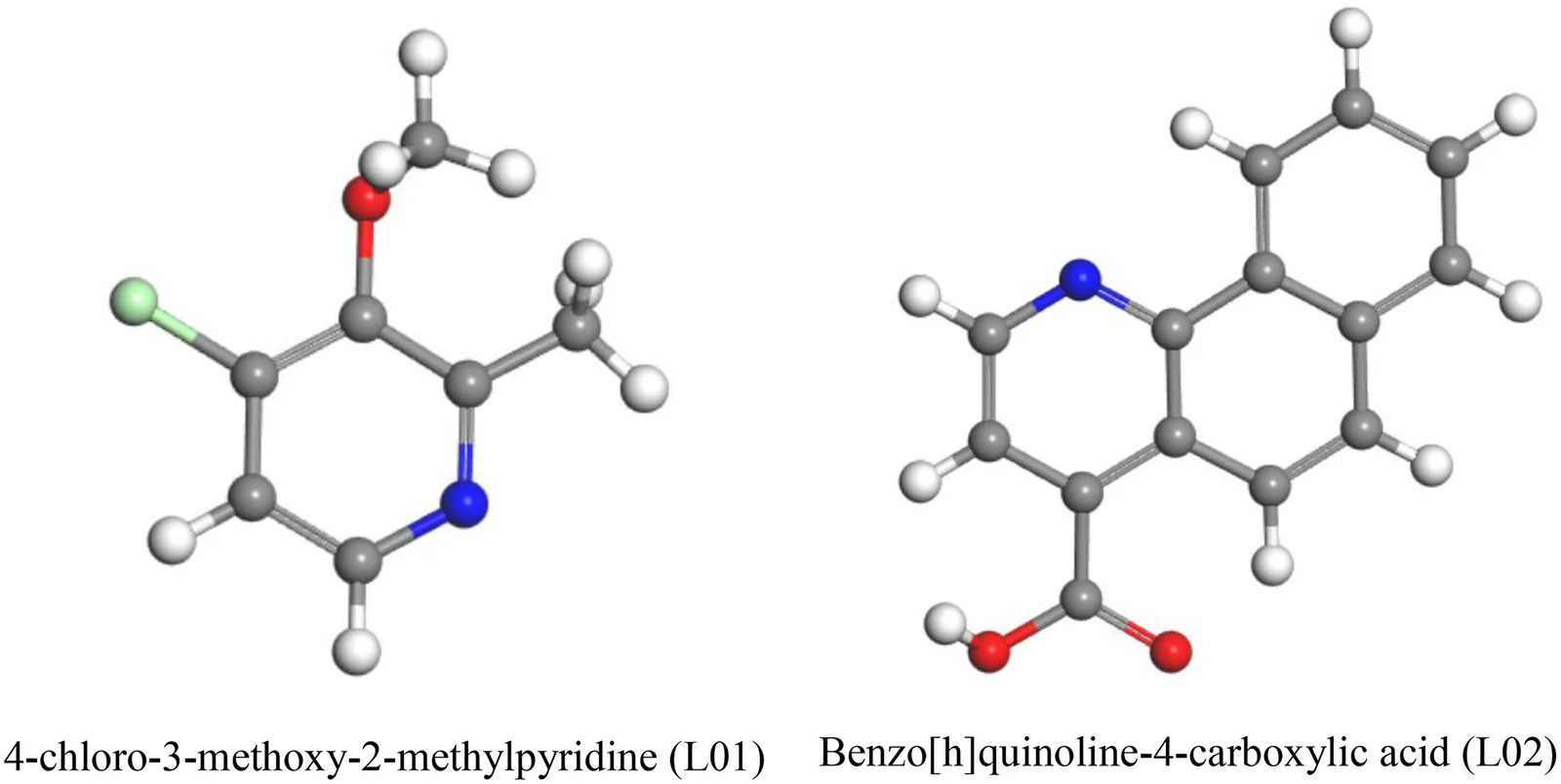
Optimized structure of inhibitors.

#### 3.1.2 *HOMO, LUMO, and chemical reactivity descriptors.*

Based on the optimized structures from DFT (B3LYP/6-31G*), molecular parameters such as HOMO and LUMO energies, HOMO–LUMO energy gap (ΔE_gap), chemical potential (µ), electronegativity (χ), hardness (η), softness (σ) and electrophilicity index (ω) were calculated and summarized in Table 3. The HOMO–LUMO gap is indicative of molecular reactivity and stability (the smaller, the more reactive; the larger, the stable) [41–44].

**Table 3:** Data of chemical descriptors from the DFT calculation.

Ligands	LUMO	HOMO	electron affinity (A)	Ionization potential (I)	Energy gap	chemical potential (μ)	chemical hardness (η)	electronegativity (χ)	softness (S)	electrophilicity index (ω)
L01	−9.17	−0.86	9.17	0.86	−8.31	−5.01	−4.15	5.01	−0.24	−3.02
L02	−8.45	−2.22	8.45	2.22	−6.23	−5.34	−3.11	5.34	−0.32	−4.57
L03	9.289	−1.33	−10.62	−3.97	−5.31	3.97	−0.18	−1.48	9.28	−1.33
L04	−9.05	−0.36	9.05	0.36	−8.68	−4.70	−4.34	4.70	−0.23	−2.55
L05	−8.84	−1.00	−0.04	8.94	8.98	−4.45	4.49	4.45	0.22	2.20
L06	−9.90	−0.48	9.90	0.48	−9.42	−5.19	−4.71	5.19	−0.21	−2.86
L07	−8.67	−0.35	8.67	0.35	−8.31	−4.51	−4.15	4.51	−0.24	−2.45
L08	−9.65	−0.97	9.65	0.97	−8.68	−5.31	−4.34	5.31	−0.23	−3.24
L09	−8.28	−0.65	8.28	0.65	−7.63	−4.46	−3.81	4.46	−0.26	−2.61
L10	−10.51	−0.36	10.51	−0.36	−10.87	−5.07	−5.43	5.07	−0.18	−2.36
L11	−8.05	−1.07	8.05	1.07	−6.98	−4.56	−3.49	4.56	−0.21	−2.97
L12	−8.21	−0.28	8.21	0.28	−7.93	−4.24	−3.96	4.24	−0.25	−2.28
L13	−10.31	−1.60	10.31	1.60	−8.71	−5.95	−4.35	5.95	−0.22	−4.06
L14	−8.03	−0.97	8.03	0.97	−7.05	−4.50	−3.52	4.50	−0.28	−2.87

The calculated quantum-chemical descriptors demonstrated differences in electronic stability and reactivity among the investigated phytochemicals. Compounds with smaller HOMO–LUMO energy gaps are generally considered more electronically polarizable and reactive, whereas larger energy gaps are associated with greater electronic stability. However, these descriptors characterize intrinsic electronic properties and should not be interpreted as direct predictors of anticancer activity or protein-binding affinity. Their principal value in the present study is to complement the molecular docking and molecular dynamics analyses by providing additional physicochemical information on the screened compounds.

The calculated energy gaps ranged widely from 0.36 eV (L10) to 8.98 eV (L05). Ligand L10 exhibited the smallest HOMO–LUMO gap, indicating higher electronic reactivity and a potentially greater tendency for charge transfer. In contrast, L05 showed the largest energy gap, consistent with higher electronic stability. Variations were also observed in other descriptors, including chemical potential, hardness, and electrophilicity index. For example, L10 displayed the most negative chemical potential and relatively high electrophilicity, whereas L05 exhibited contrasting behavior.

The quantum chemical descriptors revealed notable differences in the electronic properties and reactivity profiles across the ligand series. Ligands L01, L04, L06, L08, and L10 showed the most negative LUMO energies (ranging from −9.05 to −10.51 eV). L10, in particular, exhibited the lowest LUMO energy (−10.51 eV). However, these DFT-derived parameters provide only supportive insights into the intrinsic electronic characteristics of the compounds. They are not validated predictors of biological or anticancer activity, and a smaller HOMO–LUMO gap should not be interpreted as implying superior therapeutic potential. These results are best viewed as complementary chemical characterization that may help rationalize possible molecular interaction mechanisms when considered alongside docking and molecular dynamics data.

#### 3.1.3 Frontier molecular orbital (FMOs): HOMO and LUMO.

Frontier molecular orbital analysis was employed to examine the electronic distribution of the screened phytochemicals. The HOMO identifies regions with relatively greater electron-donating character, whereas the LUMO indicates regions capable of accepting electron density. The HOMO–LUMO energy gap was considered an electronic-reactivity descriptor and was not interpreted as a direct measure of protein-binding affinity or anticancer activity [45–49]. A smaller HOMO–LUMO energy gap favors stronger interaction of the ligands with protein targets, and the patterns seen in Fig 2 and S2 Fig reveal multiple HOMO and LUMO‑rich zones that help explain their predicted reactivity and pharmacological potential.

**Fig 2:**
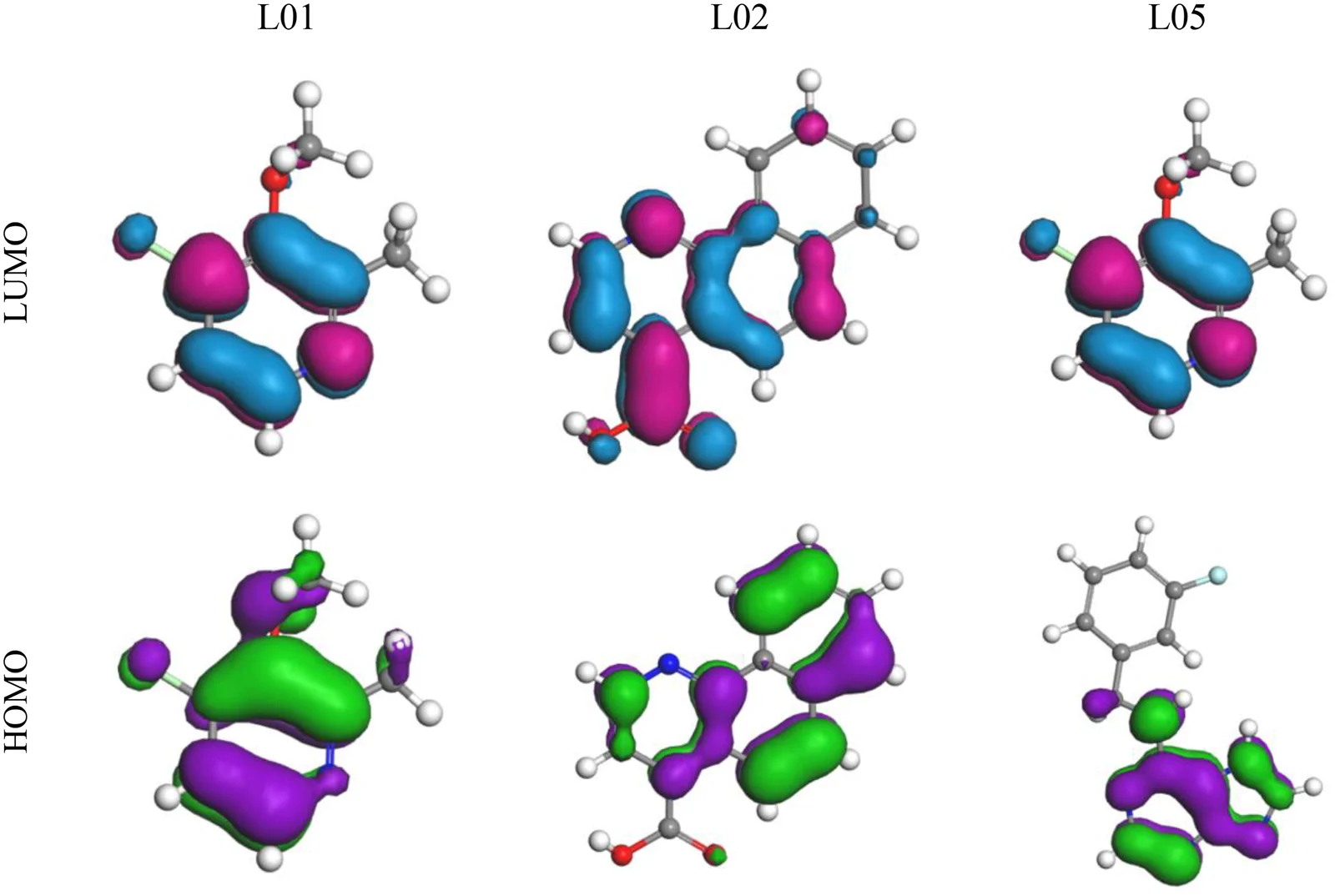
Frontier molecular orbitals diagram for HOMO and LUMO.

#### 3.1.4 Electrostatic potential map for Frontier Molecular Orbital.

Understanding the reactivity and stability of molecules in chemical reactions is paramount, often elucidated through electrostatic potential maps of Frontier Molecular Orbitals (FMO). The pivotal roles played by HOMO and the LUMO in dictating a molecule’s reactivity, as proposed by FMO theory, cannot be overstated. Analysis of the HOMO electrostatic potential map offers valuable insights into regions of electron density susceptible to nucleophilic attacks, while the LUMO map identifies sites prone to electrophilic attacks due to electron deficiency. By examining these maps, chemists can forecast reaction mechanisms and outcomes. Moreover, comparative analysis of electrostatic potential maps among different molecules enables the assessment of their relative reactivity and selectivity in diverse reactions, facilitating rationalization and prediction of chemical reactivity and aiding in the design of molecules with tailored properties. Fig 3 and S3 Fig.

**Fig 3:**
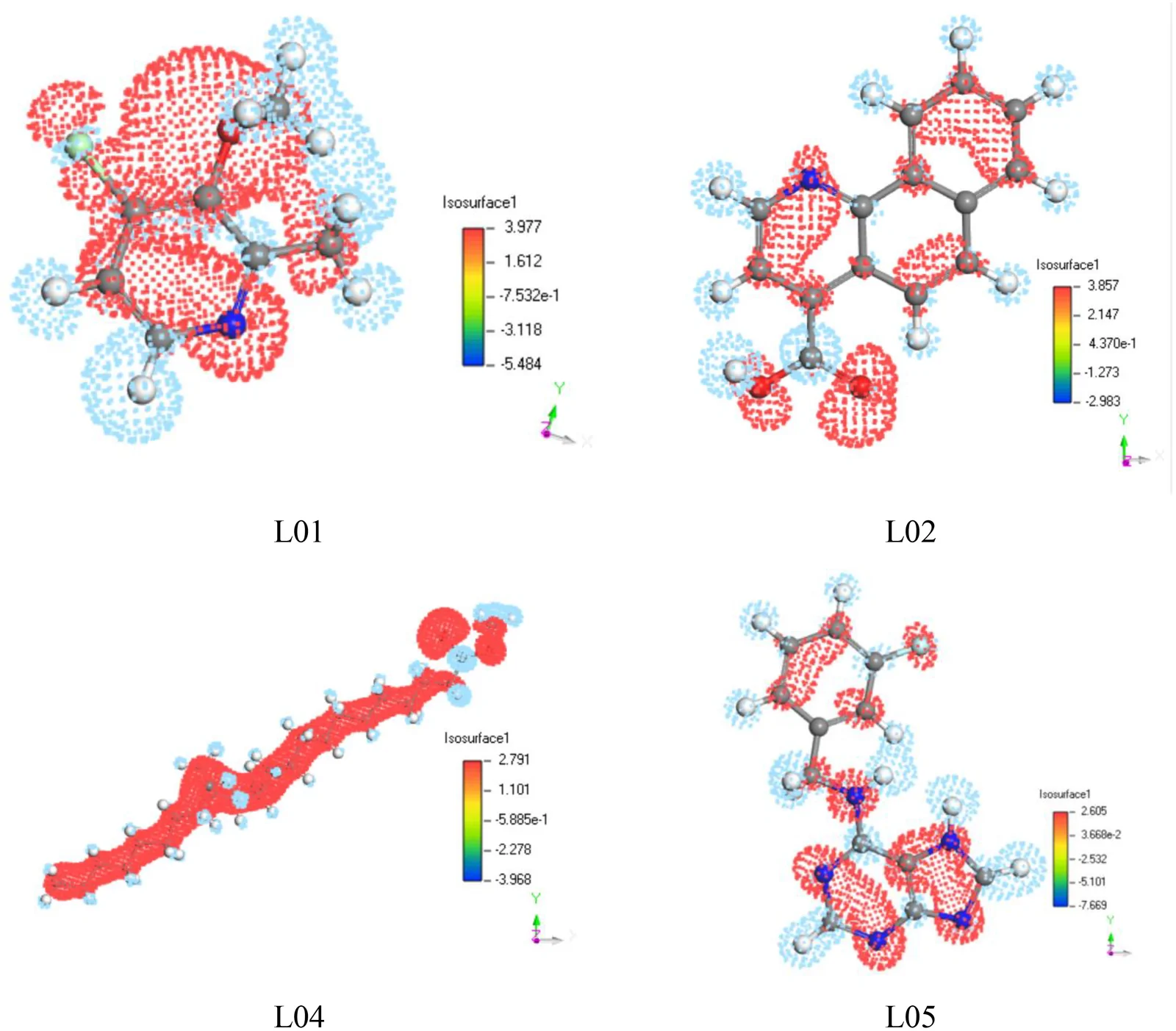
Electrostatic potential map for Frontier Molecular Orbital of L01-L04.

### 3.2. Molecular docking

#### 3.2.1 Auto docking by PyRx.

Molecular docking was carried out to support the pharmacological observations. Binding affinities of twelve ligands against the two targets (PDB IDs 1X2J and 5XNV) were evaluated using AutoDock within the PyRx interface. Strong protein–ligand recognition is essential for designing structure‑based microbial agents, and docking scores more negative than about −6.0 kcal/mol are often taken as indicative of promising activity [50,51]. Docking also helps clarify how two molecules fit together and which pose best satisfies the binding site. In these in‑silico studies, the ligands listed in Table 4 showed markedly favorable binding energies toward both targets, suggesting good potential as anticancer candidates.

**Table 4:** Data of binding energy and name of interacted ligand against lung cancer in binding affinity (kcal/mol).

S. L	1X2J			5XNV		
	binding affinity (kcal/mol)	No of H bond	No Hydrophobic bond	binding affinity (kcal/mol)	No of H bond	No Hydrophobic bond
L01	−5.1	1	3	−4.3	2	3
L02	−8.3	2	2	−6.3	1	3
L03	−8.4	1	0	−6.8	2	0
L04	−5.8	2	4	−4.0	0	1
L05	−8.5	3	5	−6.2	4	1
L06	−5.6	1	8	−3.8	0	1
L07	−7.7	3	4	−5.3	2	2
L08	−5.5	5	2	−4.4	2	1
L09	−5.4	1	1	−4.2	2	1
L10	−6.5	6	1	−5.6	3	2
L11	−8.9	1	11	−6.4	1	10
L12	−7.3	2	5	−6.0	2	4
L13	−6.8	1	0	−4.3	2	0
L14	−8.8	0	9	−6.9	0	7

Molecular docking using PyRx (AutoDock Vina) revealed moderate binding affinities for the screened *R. mucronata* phytochemicals against the target proteins 1X2J (Keap1) and 5XNV (YEATS2), with scores generally falling in the range of −4.0 to −8.9 kcal/mol (Table 4). For the Keap1 Kelch domain (1X2J), ligands L11 and L14 exhibited the most favorable binding scores (−8.9 and −8.8 kcal/mol, respectively), followed by L05 and L02 (−8.5 and −8.3 kcal/mol), while L10 formed the highest number of hydrogen bonds (six) despite a comparatively moderate docking score. In contrast, several ligands (L01, L04, L06, L08, and L09) showed weaker binding energies (approximately −5.1 to −5.8 kcal/mol), and L03 and L13 failed to produce reliable poses. A broadly similar pattern was observed for the YEATS2 domain (5XNV), where L14, L11, L05, L12, and L02 displayed relatively better scores (−6.9 to −6.0 kcal/mol) compared with other compounds. These docking results indicate that multiple ligands can establish hydrogen bonds and hydrophobic contacts within the binding pockets; however, the observed affinities remain in the moderate range typical of initial screening hits. It is important to note that docking scores alone do not confirm biological activity, and benchmarking against known inhibitors or co-crystallized ligands would be necessary to better assess the relative significance of these findings. The differences observed between PyRx and subsequent Glide docking results further highlight the importance of using orthogonal methods and cautious interpretation when prioritizing lead candidates.

#### 3.2.2 Molecular docking by Glide from Schrodinger suite.

Binding affinities of twelve ligands toward the two lung cancer targets 1X2J and 5XNV were assessed using Glide docking. Ligands were prepared with LigPrep (Schrödinger suite) to obtain high‑quality, atom‑level 2D and 3D representations; this workflow included 2D‑to‑3D conversion, generation of reasonable tautomers and stereoisomers, and subsequent correction and optimization of each structure. Receptor grids for docking were then constructed with the Receptor Grid Generation module of Glide within Maestro (Schrödinger Release 2024‑1: Glide, Schrödinger, LLC, New York, NY, 2024) [52–54], results have shown in Table 5.

**Table 5:** Data of binding energy and name of interacted ligand against lung cancer in binding affinity (kcal/mol).

S. L	1X2J			5XNV		
	binding affinity (kcal/mol)	No of H bond	No. Hydrophobic bond	binding affinity (kcal/mol)	No of H bond	No. Hydrophobic bond
L01	−5.944	1	4	−4.814	0	5
L02	−5.137	0	6	−5.733	2	3
L03	−4.427	0	10	−2.168	1	4
L04	−5.816	0	2	NA	NA	NA
L05	−6.779	0	3	−3.783	2	4
L06	−1.507	3	7	−1.498	2	5
L07	−7.407	10	4	−4.252	4	3
L08	−6.925	11	3	−3.572	6	1
L09	−2.952	1	2	NA	NA	NA
L10	−7.789	8	4	−5.482	5	0
L11	−3.877	1	5	−3.338	2	5
L12	−5.296	3	3	−4.454	2	3
L13	−1.873	0	7	−1.817	1	1
L14	−5.355	1	7	−2.509	0	5

The rank ordering of the ligands differed between AutoDock Vina and Glide. In the AutoDock Vina analysis, L11 and L14 showed the most favorable scores against 1X2J and 5XNV, respectively, whereas Glide identified L10 as the highest-ranked ligand for 1X2J and L02 as the highest-ranked ligand for 5XNV from Table 5. These differences are not unexpected because AutoDock Vina and Glide employ different receptor-preparation procedures, conformational-search algorithms, scoring functions, and treatment of intermolecular interactions. Therefore, the results from the two platforms were interpreted as complementary rather than directly interchangeable. The Glide-ranked 1X2J–L10 and 5XNV–L02 complexes were subsequently selected for molecular dynamics simulations to maintain methodological consistency between Glide ranking, interaction analysis, and MD lead selection.

#### 3.2.3 Protein-Ligand interaction for PyRx in view of superpose/position.

In the complex domain of molecular docking, the alignment of ligands after docking is a crucial step, similar to elucidating the intricate interaction between molecules on a minuscule scale, as depicted in Fig 4. Superposition of the docked ligand poses was used to compare their spatial orientations within the receptor-binding pockets. Differences in ligand orientation, pocket occupancy and interactions with key amino-acid residues were examined to identify common binding features and ligand-specific interaction patterns. This analysis provided complementary structural information for interpreting the docking scores and identifying potentially favorable binding modes.

**Fig 4:**
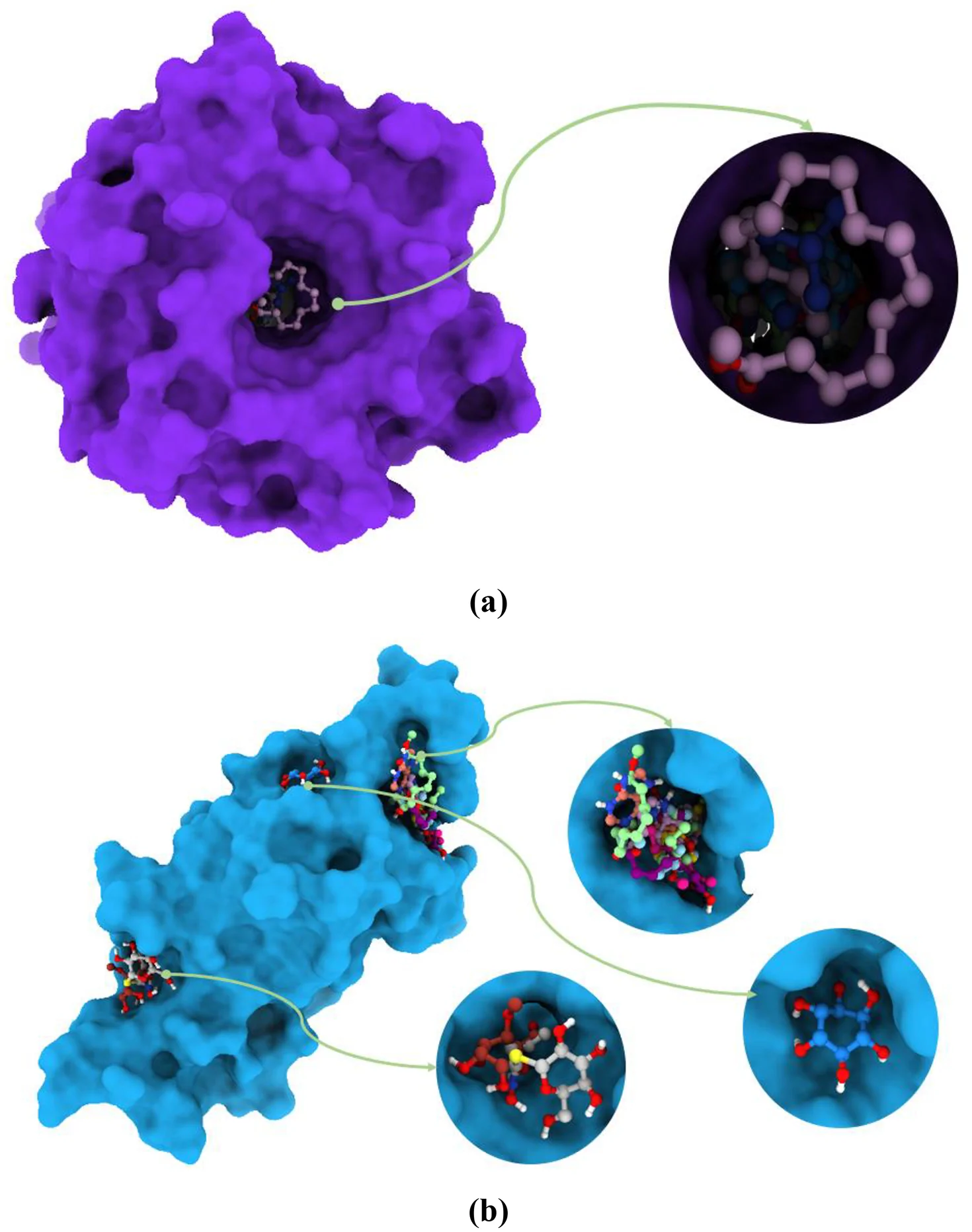
Molecular docking poses of (a) 1X2J; (b) 5XNV.

#### 3.2.4 Different poses of docking for protein ligand interaction by PyRx docking.

Post-docking interaction analysis was performed using Discovery Studio to characterize the binding poses generated by AutoDock Vina. According to the PyRx results, L11 exhibited the most favorable binding affinity toward Keap1 (1X2J), with a docking score of −8.9 kcal/mol, one hydrogen bond, and eleven hydrophobic contacts. For YEATS2 (5XNV), L14 showed the most favorable PyRx docking score of −6.9 kcal/mol and formed seven hydrophobic contacts without a conventional hydrogen bond. These findings identify L11 and L14 as the highest-ranked PyRx ligands for 1X2J and 5XNV, respectively.

L12, which is illustrated in Figure 6 as a representative YEATS2-binding pose, showed a docking score of −6.0 kcal/mol against 5XNV and formed two hydrogen bonds and four hydrophobic contacts according to Table 4. Therefore, L12 should not be described as the highest-ranked PyRx ligand for 5XNV. Its interaction map is presented only to illustrate a representative mixed polar and hydrophobic binding mode within the YEATS2 pocket.

Based on the PyRx docking results, ligand L11 showed the highest binding affinity toward protein 1X2J, forming two hydrogen bonds and six hydrophobic interactions. In contrast, ligand L12 displayed the strongest binding with protein 5XNV, forming seven hydrophobic interactions without any hydrogen bonds.

Both L11 and L12 adopted well‑defined poses within the functional binding pockets of Keap1 (PDB: 1X2J) and YEATS2 (PDB: 5XNV), respectively, which supports their predicted roles as potential lung cancer inhibitors. In Fig 5, the global surface view confirms that L11 is deeply buried within the central cavity of Keap1, indicating good shape complementarity and pocket occupancy. The enlarged 3D representation shows that L11 is surrounded by regions enriched in hydrogen‑bond donor and acceptor sites, as indicated by the colored surface, suggesting a strong contribution of polar contacts to the overall binding. The accompanying 2D diagram further highlights multiple hydrogen bonds and non‑bonded interactions between L11 and nearby residues inside the Keap1 pocket, identifying the key amino acids that anchor the ligand and likely drive the favorable docking score. Together, these panels suggest that L11 is stabilized by a balanced network of hydrogen bonding and hydrophobic contacts within a well‑defined Keap1 pocket, consistent with a strong and specific binding mode (S1-S2 Table).

**Fig 5:**
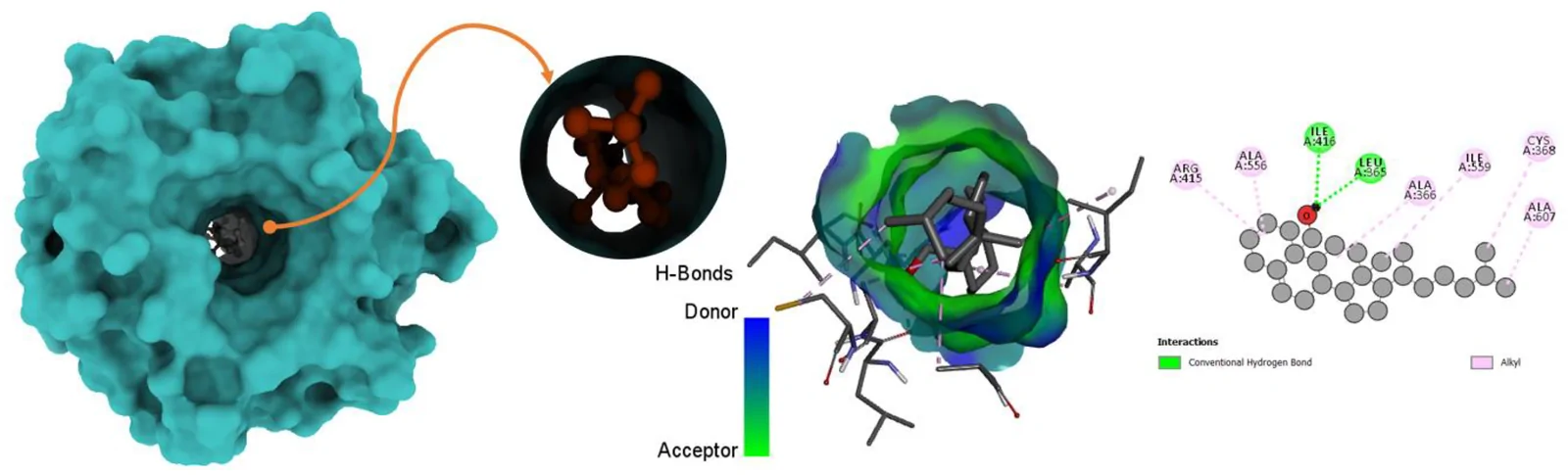
Molecular docking poses of lung cancer PDB (1X2J)- L11.

Similarly, Fig 6 illustrates the representative binding orientation of L12 within the YEATS2 binding pocket. The interaction analysis indicates two hydrogen bonds together with four hydrophobic contacts, consistent with the interaction data reported in Table 4. These contacts support stabilization of the predicted docking pose; however, L12 was not the highest-ranked PyRx ligand for 5XNV. The zoomed 3D poses shows that L12 occupies a deep sub‑pocket and is surrounded by donor and acceptor regions comparable to those seen for L11, which allows a direct visual comparison of pocket burial and interaction hot spots between the two ligands. The 2D interaction map reveals that L12 forms several hydrogen bonds, π–π stacking, and hydrophobic contacts with residues such as Glu, Lys, and Ser, supporting its docking‑predicted affinity and suggesting a mixed polar–aromatic binding mechanism. Taken together, these interaction patterns indicate that both L11 and L12 engage critical residues within their respective protein targets, implying shared pharmacophoric elements that may underlie their predicted anticancer activity through modulation of Keap1–Nrf2 signaling and YEATS2‑mediated histone reading in lung cancer.

**Fig 6:**
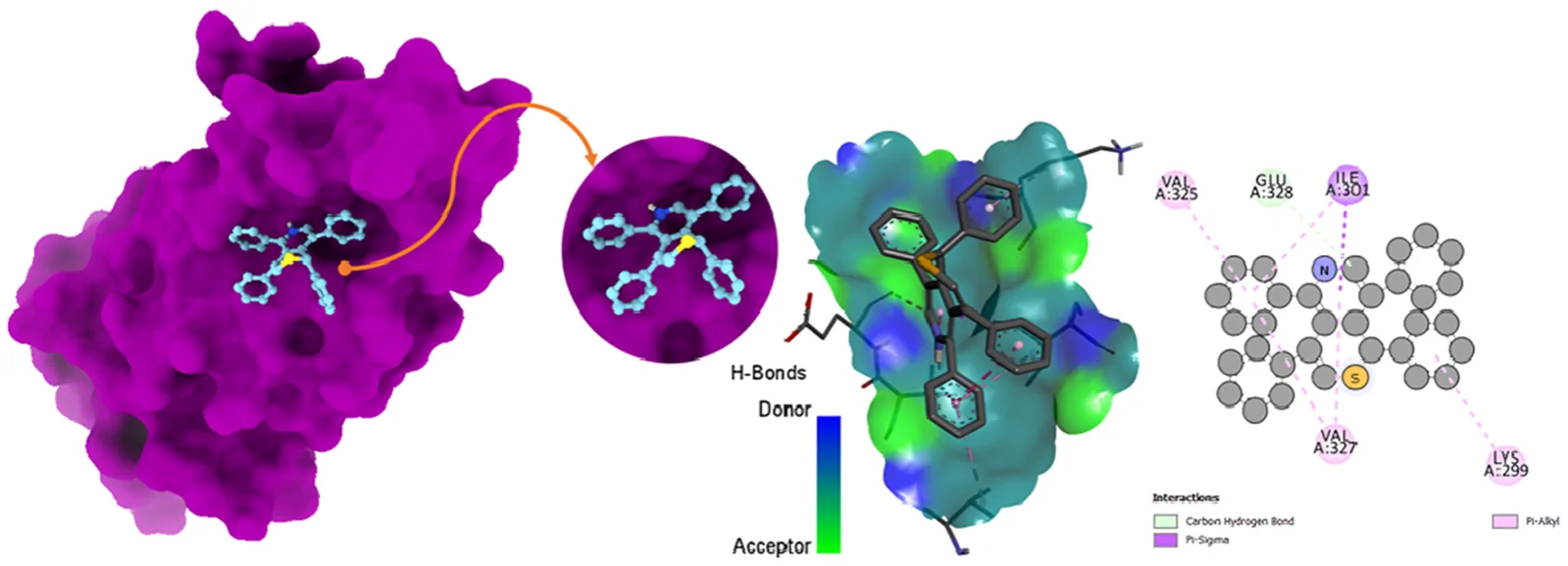
Molecular docking poses of lung cancer PDB (5XNV) -L12.

#### 3.2.5 Protein ligand interaction from Glide docking.

Protein–ligand interaction analysis of the Glide-ranked complexes was performed for 1X2J–L10 and 5XNV–L02. For 1X2J, L10 exhibited the most favorable GlideScore (−7.789 kcal/mol) and established an extensive polar interaction network within the binding pocket. For 5XNV, L02 showed the most favorable GlideScore (−5.733 kcal/mol) and formed a combination of hydrogen-bonding, aromatic and hydrophobic interactions within the YEATS domain. Fig 7 illustrates the corresponding two-dimensional interaction patterns of the two Glide-selected complexes. These interaction maps were used to characterize the predicted binding modes and to support selection of the complexes for subsequent molecular dynamics simulations.

**Fig 7:**
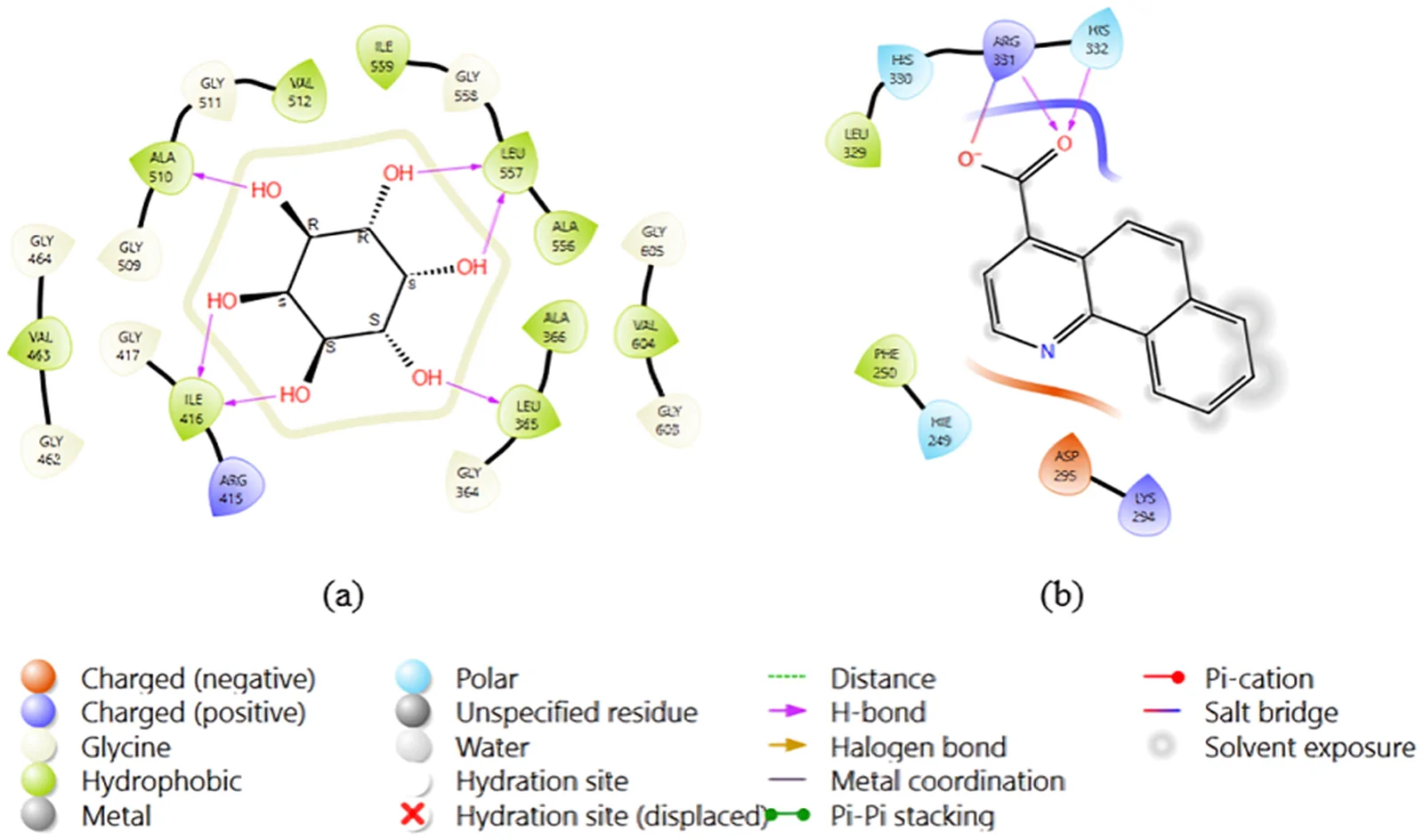
2D stick diagram of (a) PDB; 1X2J -L10; (b) PDB; 5XNV -L02, illustrating hydrogen bonds and pi-pi stacking formed with the amin2D stick diagram of (a) PDB; 1X2J -L10; (b) PDB; 5XNV -L02, illustrating hydrogen bonds and pi-pi stacking formed with the amino-acid residues at the binding pocket of lung cancer.

Fig 7 shows the 2D interaction patterns of two key ligands, L10 and L02, within the Keap1 (1X2J) and YEATS2 (5XNV) lung cancer targets, and how they use both polar and hydrophobic contacts to bind tightly. In panel (a), L10 forms several hydrogen bonds through its hydroxyl groups with nearby Keap1 residues, while surrounding hydrophobic side chains (Val, Leu, Ile, Ala) build a non‑polar shell that locks the scaffold in place; this dense hydrogen‑bond network, supported by van der Waals contacts, points to strong affinity and good shape fit within the Keap1 pocket linked to Nrf2 control in NSCLC.

In panel (b), L02 occupies the YEATS2 pocket with a mixed interaction pattern, where the heteroaromatic ring forms π–π and cation–π contacts with aromatic and positively charged residues such as Arg and His, and extra hydrogen bonds and hydrophobic contacts around the ring help fix its orientation toward the H3K27ac recognition region. These π‑based contacts, together with polar anchoring groups, match the known function of the YEATS domain as an acetyl‑lysine reader and support the view that L02 can mimic or compete with native histone ligands, placing its docking pose in line with current evidence that YEATS2 acts as an epigenetic driver and promising therapeutic target in NSCLC.

#### 3.2.6 Benchmarking with known inhibitors.

The docking results obtained in this study were interpreted in the context of previously reported inhibitors and structural studies involving the Keap1 Kelch and YEATS2 YEATS domains [55,56]. Docking scores are dependent on the docking software, receptor-preparation protocol, scoring function, grid definition, and ligand-preparation procedure; therefore, absolute docking scores obtained from different studies cannot be directly interpreted as experimentally measured binding free energies [57]. For Keap1 (1X2J), AutoDock Vina identified L11 as the highest-ranked ligand, with a docking score of −8.9 kcal/mol, whereas Glide identified L10 as the highest-ranked ligand, with a GlideScore of −7.789 kcal/mol. For YEATS2 (5XNV), L14 showed the most favorable AutoDock Vina score (−6.9 kcal/mol), while L02 showed the most favorable GlideScore (−5.733 kcal/mol). These values indicate favorable predicted interactions within the respective docking models but should not be interpreted as experimentally determined binding affinities or thermodynamic binding free energies [58,59]. Reported Keap1 and YEATS-domain inhibitors were therefore used primarily as qualitative structural and pharmacological references rather than as directly comparable numerical standards. In particular, docking scores expressed in kcal/mol are distinct from the octanol–water partition coefficient (logP), which describes molecular lipophilicity and is dimensionless. Accordingly, no direct relationship between logP and docking energy is assumed in the present analysis. The screened phytochemicals should be regarded as preliminary computational hits requiring experimental validation and further structure–activity optimization before conclusions regarding inhibitory potency can be established

### 3.3 Molecular dynamics

#### 3.3.1 Root mean square deviation (RMSD).

For subsequent molecular dynamics simulations, lead complexes were selected according to the Glide ranking and interaction profiles. L10 exhibited the most favorable GlideScore against 1X2J (−7.789 kcal/mol), whereas L02 showed the most favorable GlideScore against 5XNV (−5.733 kcal/mol). Consequently, the 1X2J–L10 and 5XNV–L02 complexes were selected for 100-ns MD simulations. The structural stability of the complexes was evaluated by monitoring the root-mean-square deviation (RMSD) of the protein Cα atoms over the simulation time (Fig 8).

**Fig 8:**
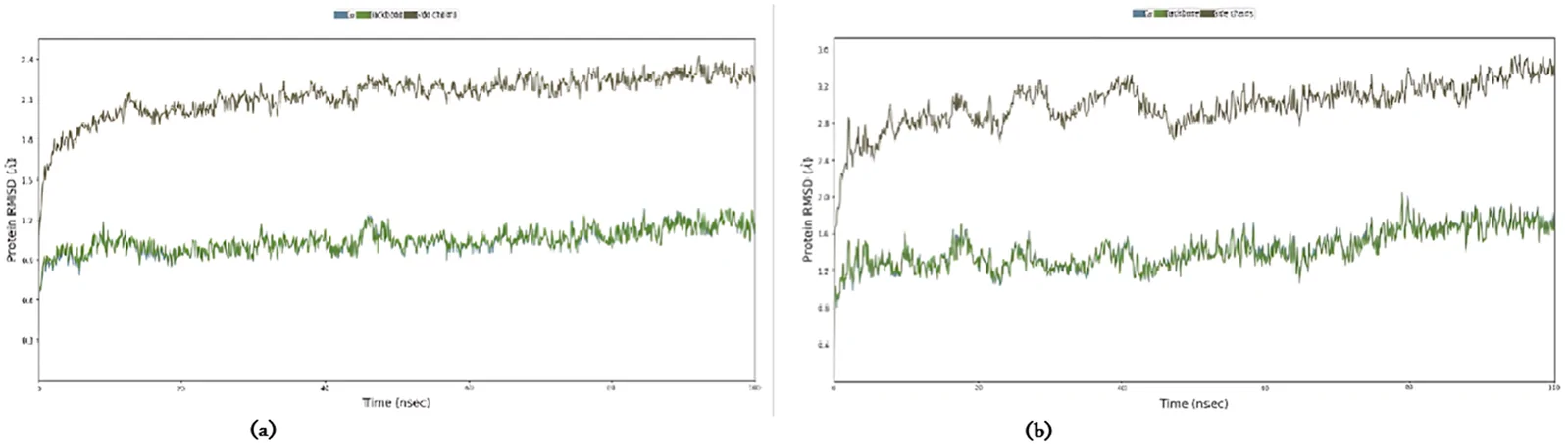
Protein-ligand complexes (a) PDB; 1X2J -L10; (b) PDB; 5XNV -L02.

As shown in Fig 8, the RMSD values for both complexes remained mostly below 3 Å throughout the 100-ns trajectory after an initial equilibration period. This relatively low deviation suggests that ligand binding did not cause major conformational disruptions to the protein backbone and that the complexes maintained overall structural integrity during the simulation.

It should be noted that while these RMSD profiles indicate acceptable stability of the docked poses under the simulation conditions, RMSD analysis alone is insufficient to confirm binding strength, specificity, or thermodynamic favorability. Additional analyses such as binding free energy calculations (e.g., MM-PBSA/MM-GBSA), comparison with apo-protein simulations, or more detailed per-residue interaction mapping would be required for a more comprehensive mechanistic understanding. Therefore, the present MD results should be interpreted as preliminary supportive evidence of complex stability rather than definitive validation of strong ligand binding.

Throughout the simulation trajectory, the RMSD profiles provided critical insights into the conformational landscapes of the complexes. A detailed examination of the temporal evolution from 0 to 34 ns revealed a rapid equilibration of the protein backbones. Notably, for the duration of the simulation, the RMSD values for both complexes remained consistently below the critical 2–3 Å threshold, which is conventionally indicative of a stable protein fold. This sustained low deviation from the initial structure confirms that the binding of the ligands (L10 and L02) does not induce major conformational shifts and that the overall tertiary structure of each protein is preserved. The consistently stable RMSD profiles underscore the inherent robustness of the docked poses and affirm the formation of resilient ligand-protein complexes. This observed structural stability is a strong indicator of reliable binding modes and enhances confidence in the computational predictions. Consequently, these findings provide a compelling foundation for the prospective application of these complexes in pharmaceutical development, such as in structure-based drug design or hit-to-lead optimization campaigns.

#### 3.3.2 Root mean square fluctuation (RMSF) *with respect of residues.*

Glide, developed by Schrödinger, is a widely used molecular docking tool designed to predict the binding orientation of small molecules (ligands) within the active site of target proteins. Analysis of protein–ligand interactions following Glide docking involves examining the docking results and visualizing the predicted binding modes. Glide provides docking scores that estimate binding affinity, where lower scores correspond to stronger binding.

Post-docking analyses play an essential role in understanding and interpreting these results. The software generates multiple binding pose; each representing a potential configuration of the ligand within the protein pocket and assigned a score that reflects the estimated binding energy. In general, lower docking scores indicate more favorable binding interactions. Assessment of the scoring function helps confirm the accuracy of predicted binding affinities and supports future refinement to improve predictive reliability. The related data are summarized in Fig 7 and S2 Table.


$$\begin{array}{c} RMSFi=\sqrt{\frac{\text{1}}{n}}\sum_{j=\text{1}}^{n}(x_{ij\;}-{\overset{―}{x}}_{i}{)}^{\text{2}} \end{array}$$

Where n represents the total number of frames or snapshots in the simulation or experimental data, *xij* is the position of residue, *i* in frame j, and *xˉi* is the average position of residue i over all frames.

Fig 9 RMSFp profiles of the 100-ns MD trajectories for both 1X2J–L10 and 5XNV–L02 complexes demonstrate outstanding conformational stability. Across >90% of residues, RMSF remained <1.5 Å, with elevations (>2.5 Å) restricted exclusively to flexible N-/C-termini and intervening loops. Core α-helices (red) and β-strands (blue) exhibited rigid motion (<1.0 Å), while ligand-contact residues (green bars) showed minimal fluctuation, confirming stable anchoring within the binding pockets. These low RMSF values align closely with experimental crystallographic B-factors and indicate that L10 and L02 impose no destabilizing strain on Keap1 or YEATS2. Collectively, the data validate the docked poses as dynamically robust, positioning these mangrove-derived leads as promising, conformationally stable inhibitors for lung-cancer therapy.

**Fig 9:**
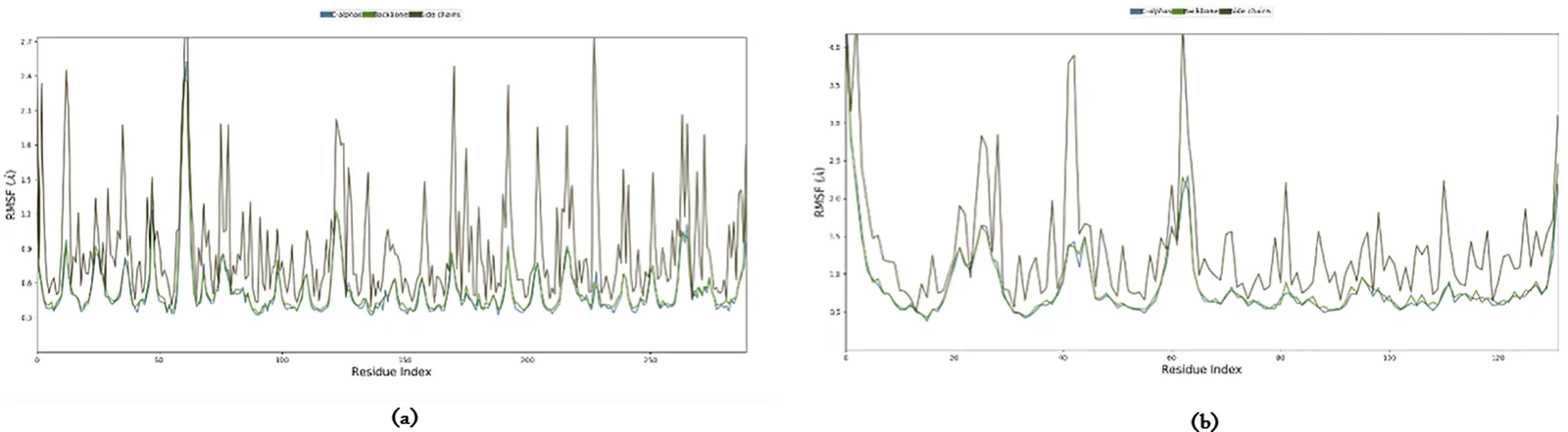
Protein-ligand complexes (a) PDB; 1X2J -L10; (b) PDB; 5XNV -L02.

#### 3.3.3 Beta factor.

In molecular dynamics (MD) simulations, the beta factor—also known as the atomic or residue B-factor, or temperature factor—describes the flexibility and motion of atoms or residues within a molecular system. It provides valuable information about the dynamic behavior of proteins and other macromolecules. To compute B-factors in MD simulations, the positional fluctuations of atoms or residues are measured. The B-factor for each atom or residue is obtained by calculating the average of the squared deviations of its position from the mean position over the simulation period:


$$\begin{array}{c} B_{i}=\;\left\langle (r_{i}-\left\langle r_{i}\right\rangle {)}^{\text{2}}\right\rangle \end{array}$$

Bi represents the B-factor for atom or residue “*i*,” while *ri* stands for the position vector of that atom or residue. The angle brackets denote averaging over time.

As shown in Fig 10, B-factors remained low (<40 Å² for >85% of residues) in both 1X2J–L10 and 5XNV–L02 systems, with elevations restricted to flexible N-/C-terminal loops. Secondary-structure elements (light-blue vertical stripes) exhibited markedly suppressed thermal motion, while ligand-contact residues (green bars) displayed minimal B-values, indicating tight, non-disruptive anchoring in the binding pockets. These low B-factor signatures align with the RMSF data and confirm that L10 and L02 impose no destabilizing strain on Keap1 or YEATS2, reinforcing their suitability as dynamically stable, mangrove-derived leads for lung-cancer protein inhibition.

**Fig 10:**
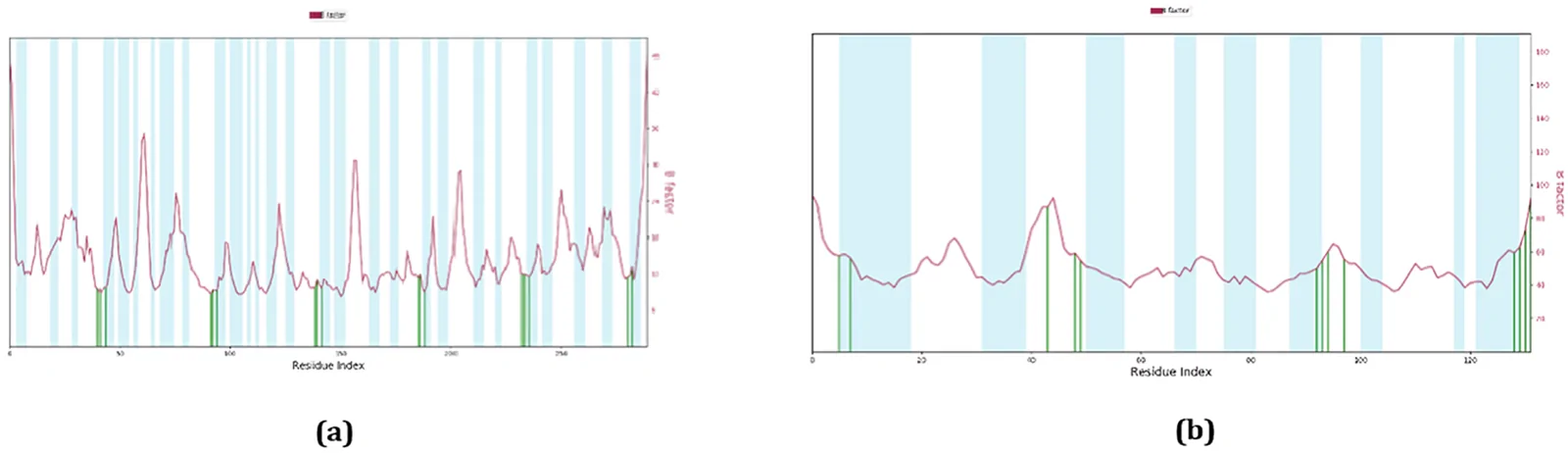
Protein-ligand complexes (a) PDB; 1X2J -L10; (b) PDB; 5XNV -L02.

#### 3.3.4 Protein ligand interaction by bond.

Protein–ligand contact analysis of the 100-ns MD trajectories (Fig 11) reveals exceptionally persistent non-covalent networks that underpin the stability of both 1X2J–L10 and 5XNV–L02 complexes. Normalized stacked-bar plots show hydrogen bonds (blue) and hydrophobic interactions (green) as the dominant contacts, with fractional occupancies frequently >0.7 and reaching 1.0 for multiple residues throughout the simulation. Water bridges (orange) and ionic interactions (pink) provide supplementary stabilization, particularly in the 5XNV–L02 system. The corresponding timeline traces (lower panels) confirm continuous, multi-residue engagement without dissociation events, with several key residues maintaining interactions for >80% of the trajectory. These sustained contacts directly corroborate the low RMSD (<3 Å), RMSF (<1.5 Å), and B-factor (<40 Å²) profiles, validating the docking poses and establishing L10 and L02 as conformationally robust mangrove-derived inhibitors of lung-cancer proteins.

**Fig 11:**
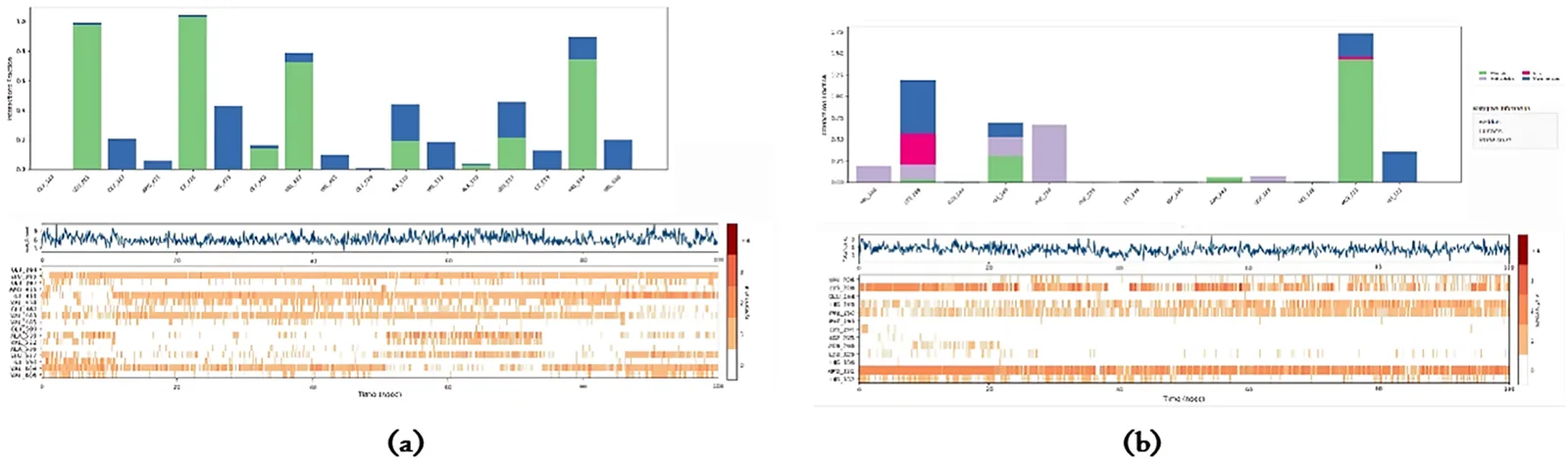
Protein-ligand complexes (a) PDB; 1X2J -L10; (b) PDB; 5XNV -L02.

Fig 11 summarizes how stable L10 and L02 remain during MD. For 1X2J–L10, RMSD stays low and contacts are frequent and long-lived, showing a stable Keap1 fold and preserved docking interactions. For 5XNV–L02, RMSD is still stable but contact fractions vary more, suggesting a flexible YEATS2 pocket and that L02 samples several related binding modes. This plasticity may help L02 adapt to different histone contexts, but longer runs or free-energy work are needed to confirm durable inhibition.

Fig 12 tracks key MD descriptors for both complexes. For 1X2J–L10, RMSD, RMSF, Rg, and hydrogen bonds fluctuate within narrow ranges, supporting a compact and stable Keap1–ligand assembly over time. For 5XNV–L02, the same parameters remain globally stable but show slightly larger local fluctuations, consistent with a more flexible YEATS2 pocket that still preserves overall complex integrity.

**Fig 12:**
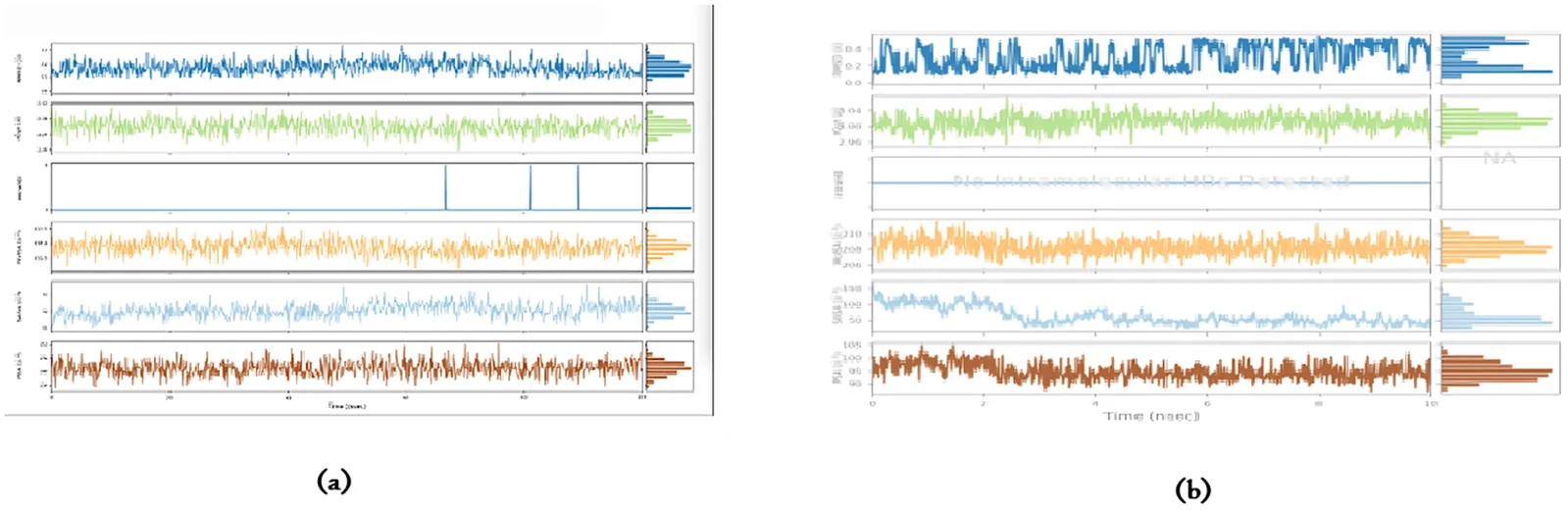
Protein-ligand complexes (a) PDB; 1X2J -L10; (b) PDB; 5XNV -L02.

### 3.4. Ramachandran plot for docked protein complex

The Ramachandran plot is a key tool in structural biology used to visualize the torsion, or dihedral, angles of a protein backbone. It maps the phi (Φ) and psi (Ψ) angles along the x- and y-axes, respectively, each ranging from –180° to 180°. The plot shown in Fig 13 illustrates these angular distributions within the protein structure.

**Fig 13:**
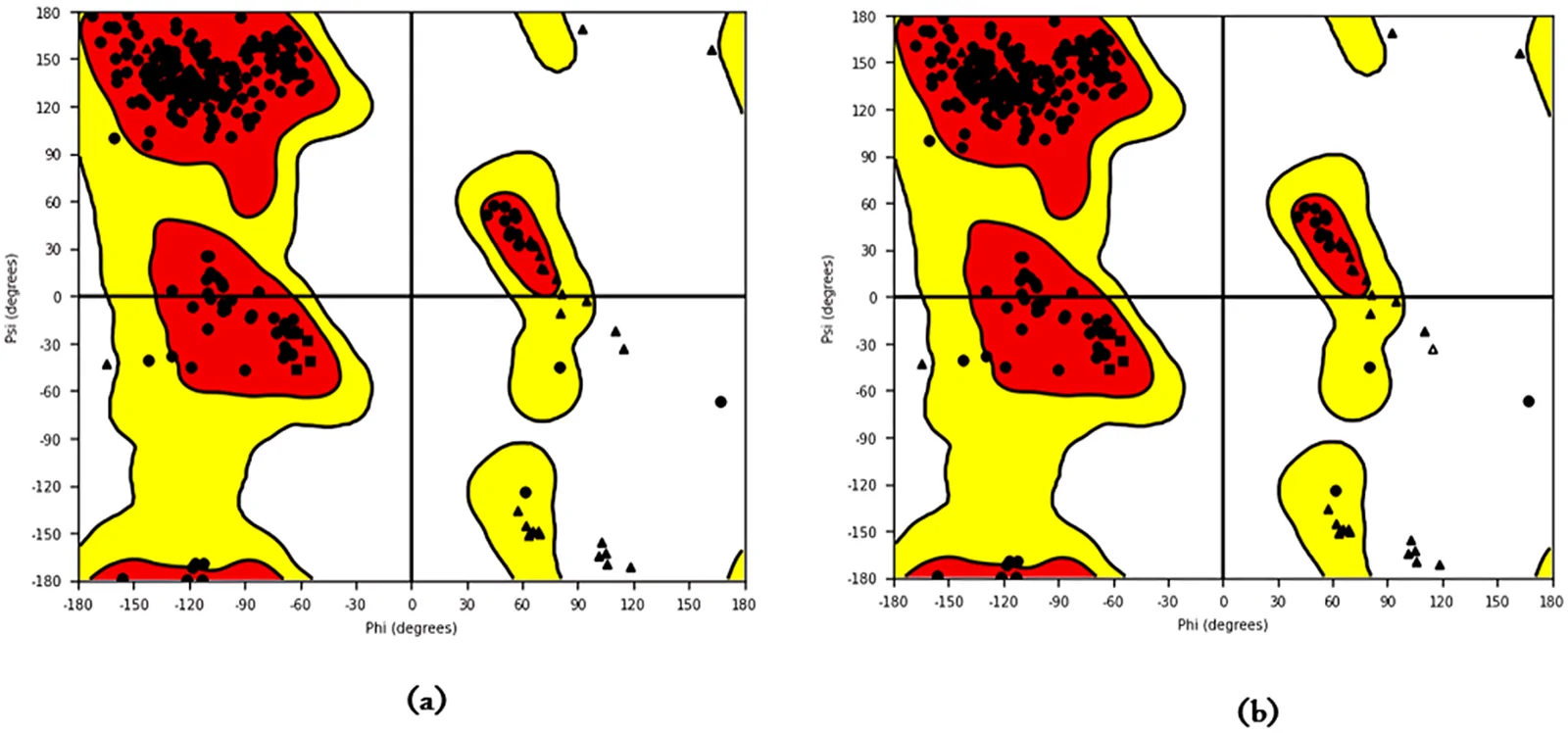
Protein-ligand complexes (a) PDB: 1X2J -L10; (b) PDB: 5XNV -L02 of Ramachandran plot for Docked protein complex.

Fig 13 shows that both docked complexes retain good backbone geometry. Most residues for 1X2J–L10 and 5XNV–L02 lie in favored or additionally allowed Ramachandran regions, confirming acceptable stereochemical quality of the modeled protein–ligand structures. The docked 1X2J–L10 complex shows a Ramachandran distribution very similar to that reported for wild‑type Keap1 Kelch structures, where about 90–92% of residues fall in the most favored regions and only a small minority appear in additionally allowed or outlier zones. This overlap in backbone φ–ψ space indicates that ligand binding and docking did not distort the characteristic six‑bladed β‑propeller geometry of Keap1, and that the modeled complex preserves the stereochemical quality expected from high‑resolution wild‑type Keap1 crystals.

### 3.5 in silico studies

#### 3.5.1 pharmacokinetics and drug-likeness study.

The physicochemical and drug-likeness properties of the screened ligands were evaluated to assess their preliminary suitability for further drug-development studies. Molecular weight, hydrogen-bonding capacity, topological polar surface area, molecular flexibility, Lipinski compliance, and predicted bioavailability were considered collectively because these parameters influence absorption, permeability, and overall pharmacokinetic behavior.

The most of these potential compounds followed Lipinski’s rule of five, the property features like molecular weight, log P (octanol-water partition coefficient), number of hydrogen bond donors and acceptors are within range which usually means good oral bioavailability mentioned in Table 6. In particular, L01, L02, L05, L07, L08 and ligands did not violate this rule even once. The topological polar surface area (tPSA) values were balance for the dataset. Some of the ligands (L01, L02, L05, L06, and IPP_L10) had tPSA values in the range of 12–51 Å^2^ while others showed moderate tPSA values (about 36–55 Å^2^), indicating that they are able to cross biological membranes assemblage [20]. On the other hand, ligands L07, L08 and L10 had higher tPSA values (∼107–148 Å^2^), thus losing passive diffusion potential that could be compensated by active transport mechanisms. Another parameter that was different between the compounds was molecular flexibility (number of rotatable bonds). L06 and L09 were highly flexible (14–16 rotatable bonds), whereas the majority of the other compounds possessed moderate flexibility (0–6 rotatable bonds). The predicted bioavailability scores were moderate for most of the ligands.

**Table 6:** Data of Lipinski rule, pharmacokinetics, and f drug-likeness.

Ligand No	Molecular weight	Number of Rotatable bonds	Hydrogen Bond acceptor	Topological polar surface area (Å²)	Lipinski rule		Bioavailability score
					Yes	No	
L01	164.65	0	2	12.53 Å²	Yes	0	0.55
L02	242.38	1	3	37.30 Å²	Yes	0	0.85
L03	406.55	0	7	64.61 Å²	Yes	0	0.55
L04	394.67	22	2	26.30 Å²	Yes	1	0.55
L05	257.35	3	5	36.75	Yes	0	0.55
L06	304.72	14	2	26.30	Yes	1	0.55
L07	293.42	5	7	148.04	Yes	0	0.55
L08	204.26	6	6	107.22	Yes	0	0.55
L09	315.72	16	2	37.30	Yes	1	0.85
L10	186.20	6	6	121.38	Yes	1	0.55
L11	386.65	6	1	20.23	Yes	1	0.55
L12	239.40	2	4	51.05	Yes	0	0.55
L13	290.44	8	4	36.92	Yes	0	0.55
L14	499.96	4	1	32.09	Yes	1	0.55

#### 3.5.2 *Toxicity.*

The tabular data presents a thorough analysis of the toxic characteristics related to various ligands, encompassing both aquatic and non-aquatic environments. Using the webtool named admetSAR (http://lmmd.ecust.edu.cn/admetsar2) pharmacokinetics and drug-likeness data were generated [60,61].

In these classification models, higher probability values indicate a greater likelihood of the positive outcome (e.g., absorption, penetration, inhibition, or substrate status), with values typically interpreted as positive predictions when exceeding ~0.5–0.6, depending on the specific endpoint and model calibration. Table 7 summarizes key predicted pharmacokinetic parameters for the ligands. Human intestinal absorption (HIA) probabilities were predominantly high (0.85–1.00 for most compounds, with L01, L02, L04, L06, L09, L11, and L14 approaching or reaching 0.99–1.00), indicating excellent predicted oral bioavailability and efficient gastrointestinal uptake. This trend aligns closely with Caco-2 permeability predictions, a well-validated in vitro proxy for intestinal epithelial transport. Ligands L01, L02, L04, L06, and L09 exhibited particularly strong permeability (0.74–0.99), supporting their potential for effective passive transcellular absorption. Blood-brain barrier (BBB) penetration probabilities varied substantially across the series. Several ligands (L01, L04, L05, L06, L09, L11, and L14) displayed high scores (>0.90), suggesting facile CNS entry. Such profiles may confer advantages for therapeutic applications requiring central activity (e.g., neurological disorders) but raise concerns for peripheral-targeted agents, where unintended CNS exposure could precipitate off-target effects or toxicity. Interactions with P-glycoprotein (P-gp), a major efflux transporter influencing bioavailability and multidrug resistance, revealed widespread moderate-to-high inhibition potential (0.72–0.99 across the set). Notably, L01, L02, L03, L07, and L10 showed strong P-gp inhibition probabilities (≥0.97), which could enhance cellular retention and intracellular accumulation of co-administered drugs or the ligands themselves. However, certain compounds (e.g., L03, L07, L08) were also predicted as P-gp substrates (probabilities 0.65–0.74), potentially leading to efflux-mediated reduced exposure in target tissues. Renal organic cation transporter (OCT) substrate probabilities exhibited considerable diversity (0.36–0.94), implying differential renal clearance mechanisms. Ligands with elevated scores (e.g., L03, L04, L06 ≥ 0.85) may undergo more efficient active tubular secretion, favoring rapid elimination and potentially lower systemic exposure upon repeated dosing. Subcellular localization predictions pointed to preferential mitochondrial or cytoplasmic accumulation for most ligands (probabilities 0.36–0.74), which could influence interactions with compartment-specific targets or metabolic enzymes. Cytochrome P450 (CYP) metabolism profiles highlighted variable substrate and inhibition liabilities. Several ligands (L04, L05, L06, L09) displayed substantial CYP2C9 substrate probabilities (≥0.80), consistent with expected hepatic Phase I metabolism. Inhibition of CYP1A2 was pronounced in select cases (e.g., L05: 0.88, L09: 0.91, L14: 0.92), raising the possibility of drug–drug interactions via impaired clearance of CYP1A2 substrates.

**Table 7:** Summary of the aquatic and non-aquatic toxicity properties of the studied ligands.

Ligand	Absorption Human Intestinal	Caco-2 Permeability	Blood-Brain Barrier (BBB)	P- I glycoprotein inhibitor	Cation Transporter Renal Organic	P- II glycoprotein substrate	Sub-cellular localization	Substrate CYP450 2C9	Inhibitor CYP450 1A2
L01	0.99	0.79	0.98	0.98	0.81	0.75	0.62	0.80	0.66
L02	0.99	0.79	0.97	0.98	0.85	0.78	0.48	0.79	0.60
L03	1.00	1.00	1.00	0	0	0	0.45	0	0
L04	0.98	0.78	0.98	0.85	0.87	0.67	0.51	0.86	0.62
L05	0.96	0.60	0.98	0.80	0.60	0.50	0.38	0.87	0.88
L06	0.97	0.74	0.97	0.89	0.88	0.71	0.60	0.83	0.79
L07	0.93	0.62	0.53	0.98	0.76	0.74	0.53	0.81	0.73
L08	0.85	0.80	0.59	0.86	0.93	0.59	0.74	0.85	0.86
L09	0.99	0.99	0.95	0.94	0.92	0.59	0.54	0.76	0.91
L10	0.86	0.55	0.51	0.99	0.92	0.73	0.64	0.85	0.84
L11	1.00	0.67	0.98	0.77	0.64	0.58	0.65	0.80	0.84
L12	0.98	0.53	0.92	0.72	0.59	0.57	0.74	0.71	0.58
L13	92.51	1.952	−0.447	0.98	0.93	0.00	0.61	0.00	0.00
L14	1.00	0.59	0.94	0.90	0.81	0.64	0.36	0.80	0.92

## 4. Conclusion

In this multi-scale computational study, phytochemicals from the mangrove species *R. mucronata* was investigated for their ability to modulate two important lung cancer-related proteins: the Keap1 Kelch domain (PDB 1X2J) and the YEATS2 YEATS domain (PDB 5XNV). In this study, a number of compounds with favorable electronic properties, moderate predicted binding affinities and acceptable in-silico physicochemical profiles were screened using Density Functional Theory (DFT), molecular docking (PyRx and Schrödinger Glide), 100-ns molecular dynamics simulations and ADMET profiling. Among the analyses performed, Ligand L10 (cyclohexane-1,2,3,4,5,6-hexaol) was one of the most preferred candidates. Our results propose that naturally derived compounds from *R. mucronata* can be a promising starting point for the design of new inhibitors of Keap1–Nrf2 and YEATS2-pathways, relevant to NSCLC. Nevertheless, the current results are purely based on calculations. Docking scores, DFT descriptors and MD stability indicators suggest potential activity in a semi-quantitative manner and should not be over-interpreted as indicative of biological activity. Experimental validation will be a critical way to build on these findings. We propose a series of initial validation steps to assess the efficacy in vitro: [1] cytotoxicity and anti-proliferative effects in NSCLC cell lines such as A549 and H1975 that represent Keap1-mutant models with selectivity assessment in normal lung epithelial cells [2], examination of target engagement and pathway modulation utilizing Western blotting, qRT-PCR, and H3K27ac-dependent reporter assays for Nrf2 transcriptional activity; [3] indications of apoptosis, cell cycle arrest, or potential synergy with standard chemotherapeutics; and [4] further mechanistic pharmacokinetics studies on the most active compounds.

## Supporting information

S1 Fig Optimized structure of tested compounds. (DOCX)

S2 Fig Frontier Molecular orbital: HOMO LUMO. (DOCX)

S3 Fig Electrostatic potential map for frontier molecular orbital. (DOCX)

S1 Table Ligands Interaction with amino acid residues and their bond distance (PDB ID 1x2j). (DOCX)

S2 Table Ligands Interaction with amino acid residues and their bond distance (PDB ID 5xnv). (DOCX)

## Abbreviations

MD: Molecular dynamics
ADMET: Absorption, Distribution, Mechanism, Excretion, and Toxicity
PDB: Protein Data Bank
RMSD: Root mean square deviation
RMSF: Root mean square fluctuation
