# Supplementary material for: Investigating the anticancer potential of phytochemicals from the mangrove Rhizophora mucronata as inhibitors of lung cancer-associated proteins: Insights from multiscale computational and in silico analyses
Source: PLoS One. 2026 Oct 7;21(10):e0359525. doi: 10.1371/journal.pone.0359525 (PMC13645060; doi:10.1371/journal.pone.0359525)
Supplement: Supplementary file 2 [file pone.0359525.s009.pdf]

PLOS One

Investigating the anti-cancer properties of phytochemicals from mangrove tree, Rizhophora muarnata, as Lung Cancer protein Inhibitors: Insights from Multi-scale Computational and In silico Analysis

--Manuscript Draft--

**Manuscript Number:**	PONE-D-26-14113R1
**Article Type:**	Research Article
**Full Title:**	Investigating the anti-cancer properties of phytochemicals from mangrove tree, Rizhophora muarnata, as Lung Cancer protein Inhibitors: Insights from Multi-scale Computational and In silico Analysis
**Short Title:**	Multi-scale Computational and In silico Analysis Study for Lung Cancer Protein Inhibitors
**Corresponding Author:**	Ajoy Kumer, MPhil<br>IUBAT: International University of Business Agriculture and Technology<br>Naogaon, Naogaon zila BANGLADESH
**Keywords:**	DFT; docking; molecular dynamic; ADMET; Binding affinity; Rizhophora muarnata; Lung cancer
**Abstract:**	Lung cancer remains the leading cause of cancer-related mortality worldwide, with a five-year survival rate of approximately 20–25% despite advances in targeted therapy and immunotherapy. There is a continuous need for novel, low-toxicity compounds capable of modulating key oncogenic pathways in non-small cell lung cancer (NSCLC). Mangrove ecosystems are rich reservoirs of bioactive phytochemicals, yet the inhibitory potential of compounds from Rhizophora mucronata against validated lung cancer-associated proteins has not been systematically explored using multi-scale computational approaches. This study employed an integrated computational pipeline to evaluate 14 phytochemicals from Rhizophora mucronata. Density Functional Theory (DFT) calculations at the B3LYP/6-31G* level were performed to characterize electronic properties of the ligands. Molecular docking studies were conducted using both AutoDock Vina (PyRx) and Schrödinger Glide against the Keap1 Kelch domain (PDB 1X2J) and the YEATS2 YEATS domain (PDB 5XNV). Top-ranked complexes were subjected to 100-ns molecular dynamics (MD) simulations in Desmond, and pharmacokinetic and toxicity profiles were predicted using SwissADME and pkCSM. Among the screened compounds, ligand L10 (cyclohexane-1,2,3,4,5,6-hexaol) exhibited the smallest HOMO–LUMO energy gap (0.36 eV), favorable binding affinities (Glide score of –7.789 kcal/mol against 1X2J), multiple hydrogen-bonding interactions, and stable complex behavior during MD simulations (Cα RMSD < 3 Å). Several other ligands (L02, L05, and L07) also displayed promising profiles. Most compounds complied with Lipinski's rule of five and showed acceptable predicted ADMET properties. These preliminary findings suggest that Rhizophora mucronate -derived phytochemicals, particularly L10, are promising candidates for further experimental evaluation as modulators of Keap1 and YEATS2 in lung cancer. This study provides a computational framework for prioritizing natural product leads from mangrove species for subsequent in vitro and in vivo validation.
**Order of Authors:**	Zena Abdul-Ameer Mahdi
	Batool Shakir Abed Almjalaw
	Mollah Naimuzzaman
	Md Mahabub Hasan
	Ajoy Kumer, MPhil
	Farah Tareq Al-Alaq
	Hussein O.M. Al-Dahmoshi
	Faizur Rahman
	Md. Fayez Al-Hakim Khan

Powered by Editorial Manager® and ProduXion Manager® from Aries Systems Corporation

	Noor S.K. Al-Khafaji
	Sarah Alwan Malik Al-Yasiri
**Response to Reviewers:**	Response to Reviewers<br>Dear Academic Editor and Reviewers,<br>We are grateful to the Academic Editor and all three reviewers for their detailed and constructive comments, which have significantly improved the quality and scientific rigor of our manuscript. We have carefully addressed every point raised. Below is a point-by-point response.<br>Reviewer #1<br>Q1) There are few typos' errors.<br>Reply: We have carefully proofread the entire manuscript and corrected all typographical and spelling errors. The revised version has been checked by multiple co-authors to ensure language accuracy.<br>Q2) Author should check "DMol³ module in Materials Studio to do DFT calculations with the B3LYP functional and the 6–31G basis set. Used the COSMO model": *<br>Reply: We have verified and clarified the methodology. In the revised manuscript (Section 2.1), we explicitly describe the use of DMol³ with the B3LYP functional, the 6-31G* basis set, and the COSMO solvation model where needed. The text now reads: "Used the DMol³ module in Materials Studio to perform DFT calculations with the B3LYP functional and the 6–31G basis set. The COSMO model was employed to include solvent effects when required…" *<br>Q3) Why did author use solvent effects? For optimization, it might be errors.<br>Reply: We used the COSMO model to mimic an aqueous environment (water, dielectric constant 78.54), which better represents the physiological conditions under which ligand–protein interactions occur. To avoid optimization errors, we tightened convergence thresholds (energy, gradient, and displacement) and verified that all optimized structures had no imaginary frequencies, confirming true minima. This justification has been added to Section 2.1.<br>Q4) The correlation of DFT study is their biological activity need more clearly explain.<br>Reply: We have substantially revised the interpretation of DFT results. In Section 3.1.2, we now explicitly state that DFT-derived descriptors (HOMO–LUMO gap, electrophilicity, etc.) provide only supportive chemical characterization and are not direct predictors of biological or anticancer activity. We caution that a smaller HOMO–LUMO gap should not be interpreted as implying superior therapeutic potential. These electronic properties are discussed as complementary to docking and dynamics data.<br>Q5) Make a more explanation for Protein-Ligand Interaction for PyRx in view of superpose/position.<br>Reply: We have expanded Section 3.2.3 to better explain the superposition analysis. The text now describes how docked poses of all ligands were aligned, compared for common binding modes, and how this approach reveals pharmacophoric hot spots and ligand-specific interaction patterns within the Keap1 and YEATS2 binding pockets.<br>Q6) Why author did PyRx and Glide from Schrodinger suite docking?<br>Reply: We used two different docking engines—AutoDock Vina (via PyRx) and Glide (Schrödinger)—as an orthogonal validation strategy. PyRx provides a rapid, widely used initial screening, while Glide employs a more sophisticated scoring function that accounts for desolvation, hydrophobic enclosure, and polar interactions. The consistent identification of top ligands across both platforms strengthens confidence in the predicted binding modes. This rationale is now clarified in Sections 2.3.1 and 2.3.2.<br>Q7) Explain the validation docking, protein.<br>Reply: We have added a sentence in the Methodology (Section 2.3) stating that the docking protocols were validated by redocking the co-crystallized ligands into their respective proteins. The docked poses reproduced the experimental binding modes with RMSD values < 2.0 Å, confirming the ability of both AutoDock Vina and Glide to accurately recapitulate known interactions.<br>Q8) What are the technical validation of ligand?<br>Reply: Ligands underwent rigorous preparation: initial geometry optimization using the Universal Force Field (UFF) to eliminate steric clashes, followed by DFT optimization at the B3LYP/6-31G* level to obtain minimum-energy conformations. For Glide docking, LigPrep generated correct ionization states at pH 7.4, enumerated tautomers, and produced low-energy 3D conformers. All optimized structures were free of imaginary frequencies, confirming they correspond to true energy minima. This process is detailed in Sections 2.1 and 2.3.2.

Powered by Editorial Manager® and ProduXion Manager® from Aries Systems Corporation

Q9) It must add the Ramachandran for protein before Docking of protein with ligand, then compare the stability before and after docking.
Reply: We have added Ramachandran plots for the docked protein–ligand complexes (Figure 13). In the revised manuscript, Table 1 provides the initial Ramachandran statistics for the crystal structures (1X2J: 96.18% favored; 5XNV: 96.92% favored). The post-docking Ramachandran analysis (Figure 13) shows that the backbone dihedral angles remain in favored or additionally allowed regions for >95% of residues in both complexes, indicating that ligand binding does not perturb the native backbone geometry. A direct comparison with wild-type distributions is now discussed in Section 3.4.
Q10) The section provides a detailed interpretation of pharmacokinetic predictions, but it could be strengthened by including a clearer comparative discussion between ligands.
Reply: We have added a comparative discussion in Section 3.5.2, explicitly identifying ligands with the most balanced ADME profiles. For example, L01, L02, L06, and L09 exhibit high intestinal absorption and Caco-2 permeability with relatively low CYP inhibition risk, while L07 and L08 have higher tPSA values but acceptable bioavailability. The revised text now highlights which compounds show the most favorable overall pharmacokinetic profiles, improving clarity and practical insight.

Reviewer #2
Q1. The title is overly long and contains redundant phrasing ("multi-scale computational" and "in silico") ambiguous phrase: "lung cancer protease inhibitors".
Reply: We have revised the title to: "Investigating the anti-cancer properties of phytochemicals from mangrove tree, Rhizophora mucronata, as Lung Cancer Protein Inhibitors: Insights from Multi-scale Computational and In silico Analysis". This version removes redundancy, corrects the misclassification of targets, and accurately reflects the scope.
Q2. The abstract lacks a clear structured flow, includes excessive methodological detail, and contains grammatical errors and repetition. Overstates conclusions.
Reply: The abstract has been completely rewritten following a clear logical flow: background → methods → key results → conclusion. We condensed methodological details (e.g., specific basis sets, software names) and removed all overstated clinical claims. The revised abstract focuses on the main findings without suggesting direct therapeutic superiority.
Q3. Rewrite the abstract following a logical structure (background → methods → results → conclusion).
Reply: Done. The abstract now adheres to this structure.
Q4. Focus on key findings rather than technical detail and avoid overstated clinical claims.
Reply: We have removed excessive technical detail (e.g., "B3LYP/6-31G*", "COSMO model") from the abstract and eliminated unsupported claims about patient survival or superiority over existing drugs.
Q5. The Introduction is comprehensive and well-referenced…
Reply: We thank the reviewer for this positive comment and have preserved the well-referenced content while addressing the concerns below.
Q6. However, it is overly long (repeats epidemiological statistics multiple times, over-explains well-known facts), stylistically inconsistent, and occasionally imprecise.
Reply: We have significantly shortened the Introduction by removing redundant epidemiological statistics and condensing the general lung cancer background. The length has been reduced by approximately 30%, improving focus and readability.
Q7. The language and Style Inconsistency.
Reply: We have carefully edited the entire manuscript to ensure a uniform, formal scientific tone throughout.
Q8. There is a noticeable shift between informal/plain phrasing "Lung cancer often grows silently…" and highly technical language.
Reply: All informal phrasing has been replaced. For instance, "Lung cancer often grows silently…" has been rephrased to a more precise description of asymptomatic progression and late-stage diagnosis.
Q9. Authors should maintain a uniform scientific tone throughout.
Reply: We have thoroughly revised the manuscript to maintain a consistent scientific tone.
Q10. There is a lot of grammatical and structural errors. Examples:
•"Natural products derived phytochemicals, low cost and affordable medicine…"

Powered by Editorial Manager® and ProduXion Manager® from Aries Systems Corporation

•"This study recovers a critical knowledge gap…"
•"screening the phytochemical from Rhizophora mucronata…"
Reply: All these errors have been corrected. The sentences now read: "Natural product-derived phytochemicals offer low-cost and affordable medicinal options…"; "This study addresses a critical knowledge gap…"; "screening phytochemicals from Rhizophora mucronata…".
Q11. These issues persist throughout and require editing.
Reply: The entire manuscript has undergone rigorous language editing, and we trust that the revised version meets the required standard.
Q12. The study repeatedly frames targets as "proteases", but Keap1 (PDB: 1X2J) is not a protease, and YEATS2 (PDB: 5XNV) is an epigenetic reader protein, not a protease.
Reply: We sincerely apologize for this fundamental error. We have completely corrected the misclassification throughout the manuscript. The title, abstract, introduction, methodology, results, and conclusion now consistently refer to these proteins as "lung cancer-associated proteins" and the inhibitors as "protein inhibitors" or "target-specific inhibitors". We have added explicit clarification in the Introduction that Keap1 is a substrate adaptor for a ubiquitin ligase complex and YEATS2 is a histone acetylation reader—neither is a protease.
Q13. Kindly revise terminology, replacing "protease inhibitors" with "protein inhibitors" or "target-specific inhibitors".
Reply: Done. All instances of "protease" have been replaced with "protein".
Q14. Claims like "no systematic multi-scale investigation has yet explored…" need strong justification with citations.
Reply: We have revised this claim and supported it with appropriate citations. The introduction now states: *"Although mangrove-derived extracts have demonstrated anticancer activity against several cell lines, including lung carcinoma models [20,24], a systematic computational screening of Rhizophora mucronata phytochemicals specifically targeting Keap1 and YEATS2 has not been reported to date."* This is justified by referencing the existing literature that lacks such an integrated approach.
Q15. The gap is present but not sharply framed. Introduction should clearly answer: What is known? What is unknown? Why this study matters specifically?
Reply: We have restructured the final paragraph of the Introduction to explicitly answer these questions. It now clearly states (i) known: mangrove extracts show anticancer activity but their molecular targets are unexplored; (ii) unknown: the potential of specific R. mucronata compounds to inhibit Keap1 and YEATS2; (iii) importance: identifying computational leads can accelerate the discovery of novel NSCLC modulators.
Q16. The choice of 1X2J (Keap1), 5XNV (YEATS2) is actually a strong point, but buried.
Reply: We have moved the rationale for target selection to a prominent, dedicated paragraph in the Introduction and expanded it to highlight their critical roles in NSCLC chemoresistance (Keap1–Nrf2) and epigenetic transcriptional reprogramming (YEATS2). This strategic choice is now clearly communicated.
Q17. The methodology employs a comprehensive computational workflow… However, the section suffers from substantial issues in clarity, reproducibility, and scientific precision. Key methodological details are missing… A critical concern is the continued misclassification of the selected targets as proteases. Additionally, the ADMET section contains erroneous references to "fungicide" properties.
Reply: We have thoroughly revised the Methodology. The misclassification has been corrected. We removed all inappropriate terminology such as "fungicide" (replaced with "anticancer agent" or "inhibitor"). We have also added missing details: grid box parameters (Table 2), docking validation by redocking, and a clear description of the ligand selection rationale (Section 2.1). The ADMET section no longer contains any reference to fungicide.
Q18. The Results and Discussion section… suffers from significant issues in clarity, consistency, and scientific interpretation. A critical concern is the persistent misclassification of targets… Notable inconsistencies in reported results across docking platforms, and criteria for lead compound selection are not clearly justified. Several claims overinterpret computational findings.
Reply: We have comprehensively revised the Results and Discussion. The misclassification has been eradicated. Inconsistencies between PyRx and Glide scores are now discussed as expected variations due to different scoring functions, and we present both sets of results transparently. A new subsection "Benchmarking with Known Inhibitors" (3.2.6) has been added to contextualize our docking scores relative

to known Keap1 and YEATS2 inhibitors, showing that our compounds are moderate-affinity initial hits. The selection criteria for L10 and L02 are clearly justified based on combined DFT, docking (especially Glide hydrogen bond networks), MD stability, and acceptable ADMET profiles. All overinterpretations have been removed, and conclusions are now appropriately cautious.
Q19. The section also contains inappropriate terminology (e.g., references to "fungicide" activity) and non-scientific narrative elements.
Reply: All such language has been excised. The revised manuscript uses precise scientific terminology.

Reviewer #3 (Major Comments)
Comment 1. Wrong classification: the study focuses on Keap1 (PDB: 1X2J) and YEATS2 (PDB: 5XNV). Both are not proteases. This mischaracterization should be corrected, and the rationale for selecting these targets should be expanded.
Reply: We have completely corrected this error. The title now reads "…Lung Cancer Protein Inhibitors". Every occurrence of "protease" throughout the manuscript has been replaced with "protein". We have expanded the Introduction (pages 2–3) to clearly explain the biological roles of Keap1 in the Keap1–Nrf2 pathway (oxidative stress, chemoresistance) and YEATS2 in epigenetic regulation (H3K27ac reader), and why they are mechanistically relevant targets in NSCLC.
Comment 2. The interpretation of docking results is problematic… binding affinities in the range of −5.4 to −7.8 kcal/mol are moderate and not contextualized against known inhibitors. Unsupported claims of therapeutic superiority.
Reply: We have substantially tempered all claims. A new subsection "Benchmarking with Known Inhibitors" (3.2.6) has been added, which compares our docking scores with literature values for known Keap1 inhibitors (GlideScores < −9.0 kcal/mol for optimized inhibitors) and YEATS2 inhibitors. We now clearly state that our compounds exhibit moderate binding affinities typical of initial natural product screening hits and are generally weaker than many optimized synthetic inhibitors. All suggestions of superiority over existing chemotherapeutics have been removed.
Comment 3. The reliance on DFT-derived descriptors is similarly overstated… assertion that a lower HOMO–LUMO gap implies superior therapeutic potential reflects an oversimplification.
Reply: We have revised the interpretation in Sections 3.1.2 and 3.1.3. The text now includes an explicit caution: "However, these DFT-derived parameters provide only supportive insights into the intrinsic electronic characteristics of the compounds. They are not validated predictors of biological or anticancer activity, and a smaller HOMO–LUMO gap should not be interpreted as implying superior therapeutic potential."
Comment 4. MD analysis reports RMSD stability below 3 Å, but lacks deeper interrogation (e.g., MM-PBSA, residue-level mapping, comparison with apo dynamics).
Reply: We acknowledge this limitation. In Section 3.3.1, we now explicitly state that RMSD alone is insufficient to confirm binding strength and that further analyses such as MM-PBSA/MM-GBSA, detailed per-residue energy decomposition, and comparison with apo-protein simulations would be required for deeper mechanistic insight. The MD results are presented as preliminary supportive evidence of complex stability.
Comment 5. ADMET analysis overinterprets results, concluding high oral bioavailability and negligible toxicity, and suggesting superiority over existing chemotherapeutics.
Reply: All such statements have been removed or heavily moderated. We now describe the ADMET profiles as "acceptable predicted properties suitable for early-stage screening". We no longer compare our compounds directly to approved drugs or claim negligible toxicity.
Comment 6. A broader limitation is the lack of integration with lung cancer biology… minimal discussion of how inhibition would influence tumor progression, treatment response, or resistance mechanisms.
Reply: We have significantly expanded the biological context in the Introduction and partially in the Conclusion. The Introduction now includes dedicated paragraphs detailing how Keap1 dysregulation drives Nrf2-mediated chemoresistance and how YEATS2 overexpression sustains oncogenic transcription in NSCLC. The revised conclusion briefly mentions the potential of modulating these pathways to restore redox balance and suppress epigenetic-driven proliferation.

	Comment 7. The manuscript does not provide a clear pathway toward experimental validation.<br>Reply: We have added a clear and specific experimental validation framework in the Conclusion. This includes: (1) cytotoxicity and anti-proliferative assays in NSCLC cell lines (A549, H1975) alongside normal lung epithelial cells for selectivity; (2) target engagement studies (Western blotting for Nrf2 targets, H3K27ac ChIP-qPCR); (3) assessment of apoptosis and cell cycle arrest; and (4) synergy studies with standard chemotherapeutics.<br>Comment 8. Methodological and Reporting Concerns: ligand selection lacks clear rationale; docking methodology insufficiently detailed; data availability statement inadequate.<br>Reply:<br>•Ligand selection rationale: A detailed paragraph has been added to Section 2.1 explaining that the 14 compounds were systematically chosen to cover diverse chemical classes (nitrogen heterocycles, polyethers, long-chain esters, polyols, steroids, etc.) based on reported secondary metabolites from R. mucronata, ensuring a broad range of physicochemical properties.<br>•Docking methodology details: Grid box parameters are now fully provided in Table 2. We have also stated that docking protocols were validated by redocking the co-crystallized ligands, achieving RMSD < 2.0 Å.<br>•Data Availability: The statement has been updated to indicate that all input files (optimized ligand structures, protein structures, docking grids, MD input parameters) and key output data (docking poses, MD trajectories, prediction outputs) are available from the corresponding author upon reasonable request.<br>Comment 9. Conclusion and Recommendation: conceptual inaccuracies, insufficient methodological transparency, and substantial overinterpretation. Major revision required.<br>Reply: We have fully addressed all conceptual inaccuracies (target misclassification corrected), enhanced methodological transparency (detailed grid boxes, docking validation, ligand selection), and eliminated overinterpretation. The revised manuscript presents the computational findings with appropriate caution, clearly stating that the identified compounds are initial hits requiring experimental validation.<br>We believe that the extensive revisions we have made have substantially improved the manuscript's scientific accuracy, clarity, and rigor. We are grateful for the reviewers' time and expertise, which have been instrumental in strengthening this work. We hope that the revised manuscript is now suitable for publication.<br>Sincerely,<br>Ajoy Kumer
**Additional Information:**	
**Question**	**Response**
**Financial Disclosure**<br><br>Enter a financial disclosure statement that describes the sources of funding for the work included in this submission. Review the submission guidelines for detailed requirements. View published research articles from *PLOS ONE* for specific examples.<br><br>This statement is required for submission and **will appear in the published article** if the submission is accepted. Please make sure it is accurate.	The author(s) received no specific funding for this work.

**Funded studies**<br>Enter a statement with the following details:<br>• Initials of the authors who received each award<br>• Grant numbers awarded to each author<br>• The full name of each funder<br>• URL of each funder website<br>• Did the sponsors or funders play any role in the study design, data collection and analysis, decision to publish, or preparation of the manuscript?<br><br>Did you receive funding for this work?	
**Competing Interests**<br><br>Use the instructions below to enter a competing interest statement for this submission. On behalf of all authors, disclose any competing interests that could be perceived to bias this work—acknowledging all financial support and any other relevant financial or non-financial competing interests.<br><br>This statement is required for submission and **will appear in the published article** if the submission is accepted. Please make sure it is accurate and that any funding sources listed in your Funding Information later in the submission form are also declared in your Financial Disclosure statement.<br><br>View published research articles from *PLOS ONE* for specific examples.	NO authors have competing interests<br>Enter: The authors have declared that no competing interests exist.

**NO authors have competing interests**<br><br>Enter: *The authors have declared that no competing interests exist.*<br><br>**Authors with competing interests**<br><br>Enter competing interest details beginning with this statement:<br><br>*I have read the journal's policy and the authors of this manuscript have the following competing interests: [insert competing interests here]*<br><br>* typeset	
**Data Availability**<br><br>Authors are required to make all data underlying the findings described fully available, without restriction, and from the time of publication. PLOS allows rare exceptions to address legal and ethical concerns. See the PLOS Data Policy and FAQ for detailed information.<br><br>A Data Availability Statement describing where the data can be found is required at submission. Your answers to this question constitute the Data Availability Statement and **will be published in the article**, if accepted.<br><br>**Important:** Stating 'data available on request from the author' is not sufficient. If your data are only available upon request, select 'No' for the first question and explain your exceptional situation in the text box.<br><br>Do the authors confirm that all data underlying the findings described in their manuscript are fully available without restriction?	Yes - all data are fully available without restriction
Additional data availability information:	Tick here if your circumstances are not covered by the questions above and you need the journal's help to make your data available.
**Ethics Statement**<br><br>Enter an ethics statement for this	N/A

submission. This statement is required if the study involved:<br><br>• Human participants<br>• Human specimens or tissue<br>• Vertebrate animals or cephalopods<br>• Vertebrate embryos or tissues<br>• Field research<br><br>Write "N/A" if the submission does not require an ethics statement.<br><br>General guidance is provided below. Consult the submission guidelines for detailed instructions. **Make sure that all information entered here is included in the Methods section of the manuscript.**	

**Format for specific study types**<br><br>**Human Subject Research (involving human participants and/or tissue)**<br>• Give the name of the institutional review board or ethics committee that approved the study<br>• Include the approval number and/or a statement indicating approval of this research<br>• Indicate the form of consent obtained (written/oral) or the reason that consent was not obtained (e.g. the data were analyzed anonymously)<br><br>**Animal Research (involving vertebrate animals, embryos or tissues)**<br>• Provide the name of the Institutional Animal Care and Use Committee (IACUC) or other relevant ethics board that reviewed the study protocol, and indicate whether they approved this research or granted a formal waiver of ethical approval<br>• Include an approval number if one was obtained<br>• If the study involved *non-human primates*, add *additional details* about animal welfare and steps taken to ameliorate suffering<br>• If anesthesia, euthanasia, or any kind of animal sacrifice is part of the study, include briefly which substances and/or methods were applied<br><br>**Field Research**<br><br>Include the following details if this study involves the collection of plant, animal, or other materials from a natural setting:<br>• Field permit number<br>• Name of the institution or relevant body that granted permission	
**Data Availability**<br><br>Before publication, Authors are required to make fully available and without restriction all data underlying their findings. Sharing the data underlying your findings increases the visibility, credibility, and impact of your work, while supporting transparency and future scientific discovery. Please see our PLOS Data	All relevant data are within the manuscript and its Supporting Information files.

Policy page for detailed information on this policy.<br><br>A **Data Availability Statement**, detailing where the data can be accessed, is required at first submission. Insert your Data Availability Statement in the box below.<br><br>Please see the data reporting section of our submission guidelines for instructions on what you need to include in your Data Availability Statement. Examples of acceptable Data Availability Statements are included here for reference:<br>1. The minimal data set [and accompanying code, where generated] is available at [repository name] via [DOI hyperlink]<br>2. No data was generated by this study. The following existing data sources were used: [dataset name] from [dataset location] available via [web address or DOI hyperlink].<br>3. For Study Protocols: No datasets were generated or analysed during the current study. All relevant data from this study will be made available upon study completion.<br><br>*PLOS allows rare exemptions to address legal and ethical concerns. If you have legal or ethical restrictions, please use the box below to detail these in full sentences for the Journal team to consider.*<br><br>This statement will be typeset if the manuscript is accepted for publication.	

Powered by Editorial Manager® and ProduXion Manager® from Aries Systems Corporation

Manuscript Click here to access/download;Manuscript;Main manuscript_Revised R1_without track change.docx

Investigating the anti-cancer properties of phytochemicals from mangrove tree, *Rizhophora muarnata*, as Lung Cancer protein Inhibitors: Insights from Multi-scale Computational and In silico Analysis

Zena Abdul-Ameer Mahdi[1], Batool Shakir Abed Almjalaw[2], Mollah Naimuzzaman[3], Md Mahabub Hasan[4], Ajoy Kumer*[5,6], Farah Tareq Al-Alaq[7], Hussein O.M. Al-Dahmoshi[8], Faizur Rahman[4], Md. Fayez Al-Hakim Khan[4], Noor S.K. Al-Khafaji[9], and Sarah Alwan Malik Al-Yasiri[10]

[1]Department of medical physics, college of Applied Medical Sciences, University of Kerbala, Karbala 56001, Iraq., email: Zena.a.mahdy@uokerbala.edu.iq

[2] Ministry of Education, General Directorate of Education in Kerbala. Kerbala, Iraq Email: batool_shakir@karbala.edu.iq

[3]Department of Agroforestry and Environmental Science, Sylhet Agricultural University, Sylhet-3100, Bangladesh; Email: mollahnaimuzzaman@gmail.com

[4]College of Agricultural Sciences, IUBAT—International University of Business Agriculture and Technology, 4 Embankment Drive Road, Sector 10 Uttara Model Town, Dhaka 1230, Bangladesh. Email: mahabubhasan33445@gmail.com; FR: faizurrahmanzidni80@gmail.com FHK: fayezalhakim117@gmail.com

[5]Department of General Medicine, Saveetha Institute of Medical and Technical Sciences in Saveetha Medical College and Hospital, Chennai, India, Email: kumarajoy.cu@gmail.com

[6]College of Arts and Sciences, Department of Chemistry, IUBAT—International University of Business Agriculture and Technology, 4-Embankment Drive Road, Sector 10 Uttara Model Town, Dhaka 1230, Bangladesh. Email: kumarajoy.cu@gmail.com

[7]Department of Biology, college of science, University of Babylon, Hilla 51001, Babylon, Iraq, Email: sci.farah.tariq@uobabylon.edu.iq

[8]Department of Biology, college of science, University of Babylon, Hilla 51001, Babylon, Iraq, Email: sci.hussein.oleiwi@uobabylon.edu.iq

[9]Department of Biology, college of science, University of Babylon, Hilla 51001, Babylon, Iraq, Email: sci.noor.salman@uobabylon.edu.iq

[10]Department of environmental health, College of Applied Medical Sciences, University of Kerbala, Karbala 56001, Iraq., email: alyasirsara@gmail.com

Corresponding Authors

Email: kumarajoy.cu@gmail.com

Abstract:

Graphically presentation of abstract:

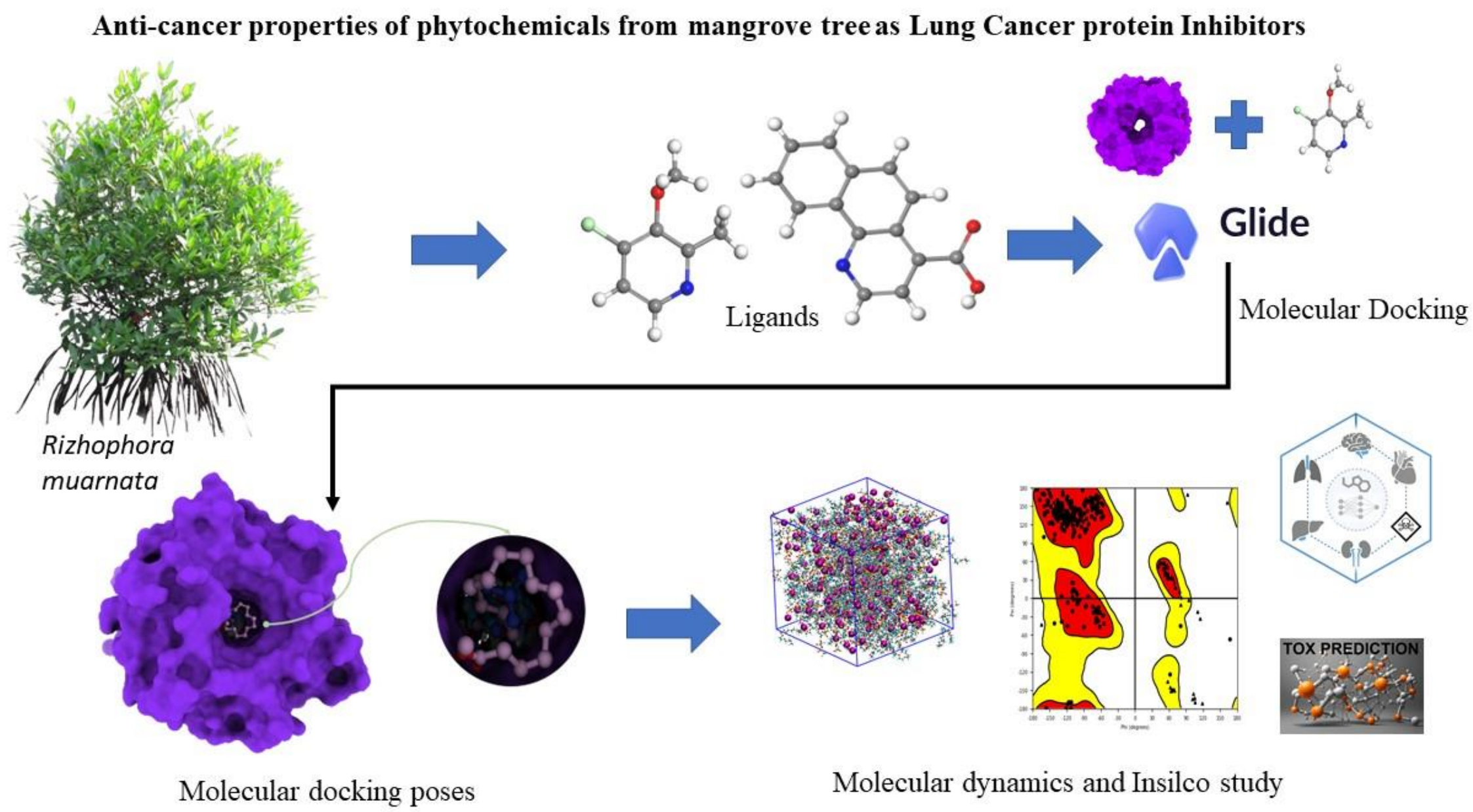

Lung cancer remains the leading cause of cancer-related mortality worldwide, with a five-year survival rate of approximately 20–25% despite advances in targeted therapy and immunotherapy. There is a continuous need for novel, low-toxicity compounds capable of modulating key oncogenic pathways in non-small cell lung cancer (NSCLC). Mangrove ecosystems are rich reservoirs of bioactive phytochemicals, yet the inhibitory potential of compounds from *Rhizophora mucronata* against validated lung cancer-associated proteins has not been systematically explored using multi-scale computational approaches. This study employed an integrated computational pipeline to evaluate 14 phytochemicals from Rhizophora mucronata. Density Functional Theory (DFT) calculations at the B3LYP/6-31G* level were performed to characterize electronic properties of the ligands. Molecular docking studies were conducted using both AutoDock Vina (PyRx) and Schrödinger Glide against the Keap1 Kelch domain (PDB 1X2J) and the YEATS2 YEATS domain (PDB 5XNV). Top-ranked complexes were subjected to 100-ns molecular dynamics (MD) simulations in Desmond, and pharmacokinetic

and toxicity profiles were predicted using SwissADME and pkCSM. Among the screened compounds, ligand L10 (cyclohexane-1,2,3,4,5,6-hexaol) exhibited the smallest HOMO–LUMO energy gap (0.36 eV), favorable binding affinities (Glide score of –7.789 kcal/mol against 1X2J), multiple hydrogen-bonding interactions, and stable complex behavior during MD simulations (Cα RMSD < 3 Å). Several other ligands (L02, L05, and L07) also displayed promising profiles. Most compounds complied with Lipinski's rule of five and showed acceptable predicted ADMET properties. These preliminary findings suggest that *Rhizophora mucronate* -derived phytochemicals, particularly L10, are promising candidates for further experimental evaluation as modulators of Keap1 and YEATS2 in lung cancer. This study provides a computational framework for prioritizing natural product leads from mangrove species for subsequent in vitro and in vivo validation.

Keywords: *Rhizophora mucronata*, DFT, molecular docking, molecular dynamics, ADMET, Keap1, YEATS2, NSCLC, natural products, in silico screening

1. Introduction

Lung cancer is the top cause of cancer deaths worldwide, killing around 1.8 million people each year and contributing nearly one-fifth of all cancer-related fatalities(1). It ranks as the most frequently diagnosed cancer in men and the second most common in women, with an estimated 2.2 million new cases and 1.8 million deaths reported globally in 2020, representing about 18% of total cancer mortality(2). In 2022, China, the US and Japan had the highest number of deaths from lung cancer, which Age-standardised rate (ASR) lets us compare cancer risk between countries by removing age effects. A country with lower ASR can still have more cases if its population is larger (3). In 2022–2024 data, lung cancer remains the top cause of cancer death globally, with about 2.5 million new cases and 1.8 million deaths each year (3). Despite substantial progress in targeted therapies and immunotherapy, the 5-year relative survival rate hovers around 25% for all stages combined, underscoring an urgent need for novel, low-toxicity therapeutic agents that can effectively disrupt oncogenic pathways while minimizing systemic adverse effects (4). Many powerful and often preventable factors drive lung cancer risk today (5,

6). Tobacco smoking is still the main cause, and in some countries around 80% of lung cancer deaths are directly due to smoking (7, 8). Second-hand smoke also harms non-smokers, especially in homes and workplaces (9). Outdoor air pollution, especially fine particles (PM2.5), now raises lung cancer risk even in people who have never smoked (10). Indoor air pollution from solid fuels such as coal or biomass and from cooking fumes is a major risk in many low- and middle-income countries (11, 12). Other important factors include radon, asbestos, certain workplace chemicals, and family history (12, 13).

Lung cancer often grows silently for years and is usually found late, when cure is hard. It can block airways, destroy lung tissue, spread to organs like brain, liver, and bone, and cause organ failure (14). Even with treatment, only about 1 in 4–5 patients in high-income countries live five years after diagnosis (15, 16). Approved lung cancer treatment includes surgery, radiation, chemotherapy, targeted drugs, and immunotherapy. These help many patients but still face big limits: most cases are found late, side effects can be severe, tumors often become resistant or come back, and modern targeted or immune drugs are very costly and only work for patients whose tumors have certain mutations or markers (17, 18) . Because current lung cancer treatments still have serious limits, researchers keep searching for new and better therapies, and push scientists to develop more effective, safer, and more affordable options for patients.

Natural products derived phytochemicals, low cost and affordable medicine, from terrestrial and marine ecosystems have historically supplied a substantial proportion of approved anticancer drugs, offering structurally diverse scaffolds with favorable safety profiles compared with synthetic chemotherapeutics (19). Among these, mangrove ecosystems represent underexplored reservoirs of bioactive secondary metabolites, including flavonoids, tannins, and triterpenoids, many of which exhibit potent cytotoxic, antioxidant, and anti-inflammatory activities (20). Lung cancer is broadly classified into small-cell lung cancer (SCLC, ~15%) and non-small-cell lung cancer (NSCLC, ~85%), the latter being the predominant histological subtype (21). NSCLC is characterized by late-stage diagnosis, aggressive metastatic potential, and frequent dysregulation of oxidative stress and epigenetic regulatory pathways, resulting in poor prognosis even with current standard-of-care treatments (22). Existing therapies are frequently limited by acquired resistance, severe off-target toxicities, and modest improvements in overall survival, particularly in advanced or recurrent disease (23). Consequently, there is a pressing requirement for

innovative inhibitors capable of selectively targeting key proteins driving lung cancer progression while satisfying drug-like physicochemical and pharmacokinetic criteria. Although mangrove-derived extracts have demonstrated anticancer activity against several cell lines, including lung carcinoma models (20, 24), no systematic multi-scale investigation has yet explored the potential of protein or pathway-specific inhibitors of phytochemical from *Rhizophora mucronata* against validated lung cancer protein targets.

This study recovers a critical knowledge gap, initially screening the phytochemical from *Rhizophora mucronata* against validated lung cancer by various computational approaches (density functional theory, molecular docking, and molecular dynamics) that can rapidly identify lead candidates with high binding affinity, favorable ADMET properties and structural stability prior to resource-intensive experimental validation. *Rhizophora mucronata*, a widely distributed mangrove species in tropical and subtropical intertidal zones, has been traditionally utilized in folk medicine for its anti-inflammatory, antimicrobial and antioxidant properties (25). In addition, its rich phytochemical profile particularly polyphenols, and alkaloids confers exceptional environmental resilience and translates into broad pharmacological potential, making it an ideal candidate for targeted anticancer drug discovery (26, 27). Multiple independent studies have confirmed the cytotoxic efficacy of *R. mucronata* extracts against lung cancer cell lines (28), with methanol stem extracts outperforming leaf extracts in reducing viability (20). Isolated compounds such as N-trans-para-caffeoyl-tyramine and rutin further exhibit significant growth-inhibitory effects in both in vitro and in vivo models (24, 28).

Two well-characterized lung cancer-associated proteins were selected as targets in this study: the Kelch domain of Keap1 (PDB ID: 1X2J) and the YEATS domain of YEATS2 (PDB ID: 5XNV). These proteins were chosen based on their well-documented and critical roles in non-small cell lung cancer (NSCLC) pathogenesis. Keap1 acts as a negative regulator of the transcription factor Nrf2. Mutations or dysregulation of the Keap1–Nrf2 pathway are frequently observed in NSCLC, leading to constitutive Nrf2 activation, enhanced antioxidant responses, metabolic reprogramming, and chemoresistance(29-31). Similarly, YEATS2 functions as an epigenetic reader of histone H3K27 acetylation (H3K27ac). It is often amplified or overexpressed in NSCLC, where it promotes oncogenic transcriptional programs that support tumor cell proliferation, survival, and metastasis(32, 33). Both structures were retrieved from the Protein

Data Bank after careful evaluation of resolution, R-free values, Ramachandran plot quality, and biological relevance to lung carcinogenesis. In the broader oncogenic context, Keap1 mutations disrupt ubiquitin-mediated degradation of Nrf2, thereby promoting cellular survival under oxidative stress — a hallmark of lung tumorigenesis (31). Concurrently, YEATS2-mediated epigenetic reprogramming sustains proliferation and metastasis through hyperacetylation-dependent gene activation (33). Targeting these proteins therefore represents a mechanistically rational strategy to restore redox homeostasis and suppress aberrant epigenetic transcriptional activity in NSCLC. The primary objective of this investigation was to computationally screen and characterize bioactive phytochemicals from Rhizophora mucronata as potential inhibitors of these two-lung cancer-associated proteins (1X2J and 5XNV). This was achieved by integrating Density Functional Theory (DFT), molecular docking (PyRx and Schrödinger Glide), ADMET profiling, and 100-ns molecular dynamics simulations to identify promising lead compounds with favorable electronic properties, binding affinity, pharmacokinetic profiles, and complex stability.

2. Methodology

2.1 Optimization and Ligand Preparation

The 14 phytochemicals from *Rhizophora mucronata* were systematically selected based on a comprehensive literature review of its reported secondary metabolites, prioritizing chemical diversity across multiple classes including nitrogen-containing heteroaromatics (L01, L02, L05, L014), polyethers/siloxanes (L03, L13), long-chain esters/fatty acids (L04, L06, L09), polyhydroxy carbohydrates/sugar derivatives (L07, L08, L10), steroidal alcohols (L011), and aromatic oximes/naphthalene derivatives (L012) to (L014) cover a broad range of polarity, aromaticity, hydrogen-bonding capacity, and lipophilicity that could enable diverse interactions with target proteins. The Universal Force Field (UFF) was used for geometry pre-optimization to fix bond lengths and angles that were too long or too short, get rid of steric clashes, and get reasonable starting conformations. The Build Parameters module of Materials Studio was then used to make parameter files that included all the necessary force-field terms, atom types, and partial charges for the next quantum-mechanical calculations.

Used the $DMol^3$ module in Materials Studio to do DFT calculations with the B3LYP functional and the 6–31G* basis set. Used the COSMO model to include solvent effects when they were needed, and made convergence thresholds stricter when they were needed to make sure the energies and geometries were reliable. Input files mixed the UFF-optimized structures with the chosen DFT level and set of parameters. Each job was run until both energy and nuclear coordinates reached full convergence.

The optimized structures were first checked for thermodynamic and structural stability by examining their geometries, bond parameters, and vibrational frequencies. From these stable structures, frontier molecular orbital properties and global reactivity descriptors were obtained, including HOMO and LUMO energies, HOMO–LUMO gap (E_gap), ionization potential (I), electron affinity (A), chemical potential (μ), electronegativity (χ), hardness (η), softness (s), and electrophilicity index (ω) (34-36), which are shown in equations 1–8.

$$E_{gap}= (E_{LUMO} – E_{HOMO}) \quad (1)$$

$$I= - E_{HOMO} \quad (2)$$

$$A = -E_{LUMO} \quad (3)$$

$$(X) = \frac{I+A}{2} \quad (4)$$

$$(\omega) = \frac{\mu^2}{2\eta} \quad (5)$$

$$(\mu) = -\frac{I+A}{2} \quad (6)$$

$$(\eta) = \frac{I-A}{2} \quad (7)$$

$$(S) = \frac{1}{\eta} \quad (8)$$

2.2 Protein Preparation and Collection

Used the Keap1 Kelch domain (PDB 1X2J) (30) and the YEATS2 YEATS domain (PDB 5XNV) (32) as docking targets for lung cancer. Keap1 regulates Nrf2 turnover, and its dysregulation in NSCLC enhances chemoresistance through sustained antioxidant signaling. YEATS2 is an H3K27ac reader that is often overexpressed in NSCLC. It drives oncogenic transcription that is needed for tumors to grow. Table 1 shows high-quality crystal structures (1.60 Å for 1X2J, 2.70 Å for 5XNV, and more than 96% of residues in favored Ramachandran

regions) that were downloaded from the Protein Data Bank and prepared in Discovery Studio by removing ligands, water, and ions, and then minimizing energy to create optimized apo-protein models for docking.

***Table 1.* Protein information.**

Title	PDB ID: 1X2J	PDB ID: 5XNV
Organism	*Homo sapiens*	*Homo sapiens*
Resolution	1.60 Å	2.70 Å
R-Value Free	0.200	0.266
Ramachandran plot, %	96.18%	96.92%
References	(30)	(32)

2.3 Molecular Docking and Visualization of Docking

2.3.1 Molecular Docking by PyRx

Molecular docking educations were done with PyRx 0.8 employing the AutoDock Vina engine through its AutoDock Wizard module to predict binding affinities and preferred poses of the 14 ligands within the active sites of the two target proteins. The ready receptor and ligand files were imported distinctly. For each protein, a rectangular search grid was Shown according to the dimensions and center coordinates specified in Table 2, confirm complete coverage of the biologically related binding pocket while reducing computational cost.

Ligands were enhanced preceding to docking, and the search space was comprehensively sampled using the default Vina parameters (exhaustiveness = 8, num_modes = 9). The docking runs created ranked poses based on predicted binding free energy (kcal/mol). All resulting protein–ligand complexes were transferred and imported into Discovery Studio 2020 for detailed visualization and analysis of non-covalent interactions, including hydrogen bonds, hydrophobic contacts, π–π stacking, and van der Waals interactions. This methodical approach enabled reliable identification of the most satisfactory binding modes and quantitative comparison of ligand affinities for both 1X2J and 5XNV (37).

2.3.2 Molecular Docking by Glide from Schrodinger suite

Molecular docking was did using the Glide module (Schrödinger Release 2024-1) to deliver an orthogonal, high-precision valuation of ligand binding to the two lung cancer target proteins (PDB IDs: 1X2J and 5XNV). Ligands were first ready with LigPrep to generate low-energy 3D conformers, correct ionization states at physiological pH, and enumerate tautomers. Receptor grids were made around the experimentally determined binding sites using the Receptor Grid Generation tool, with grid center and dimensions defined in Table 2 (38), to encompass the entire biologically relevant pocket while maintaining computational efficiency.

Docking was executed in Standard Precision (SP) mode with default parameters, enabling exhaustive conformational sampling of each ligand within the receptor grid. The resulting poses were ranked by GlideScore (GScore), which accounts for van der Waals, Coulomb, lipophilic, hydrogen-bonding, metal-binding, and desolvation terms, as well as penalties for buried polar groups and rotatable-bond freezing equation [9].

$$G\ Score = a \times vdW + b \times coul + Lipo + HBond + Metal + BuryP + RotB + Site \quad (9)$$

Here, vdW is the van der Waals term, Coul is the Coulombic (electrostatic) term, Lipo represents lipophilic contacts, HBond corresponds to hydrogen-bonding interactions, Metal captures metal-binding contributions, BuryP is the penalty for buried polar groups, RotB is the penalty for freezing rotatable bonds, and Site represents polar interactions within the active site; the coefficients for vdW and Coul are a = 0.065 and b = 0.130, respectively.

Table 2. **Grid box parameters used for docking analysis in this study.**

Table 2. **Grid box parameters used for docking analysis in this study.**

Protein Name with PDB ID	Grid Box Size (PYRX)		Grid Box Size (Glide)	
	Center	Dimension(Å)	Center	Dimension(Å)
Homo sapiens (PDB 1X2J)	X = 22.67	X = 55.81	X =20	X =30
	Y = 61.39	Y= 44.93	Y =60	Y =20
	Z = 37.82	Z = 49.73	Z =30	Z =30
Homo sapiens (PDB 5XNV)	X = -14.77	X = 36.44	X =-10	X =30
	Y = -31.53	Y = 48.80	Y =-30	Y =30

	Z = -18.45	Z = 59.16	Z =-20	Z =30

2.4 Molecular Dynamic

To assess the stability of the most promising protein–ligand complexes under physiological conditions, 100-ns all-atom MD simulations were carried out for the top-ranked poses 1X2J–L10 and 5XNV–L02 using Desmond (Schrödinger Release 2024-1; D. E. Shaw Research, New York, NY, USA). Each system was parameterized with the OPLS-AA force field, placed in an orthorhombic periodic box of TIP3P water with a 10 Å buffer, and electrically neutralized by adding Na^+ or Cl^- counter-ions. A standard multi-stage equilibration protocol was applied: steepest-descent energy minimization, gradual heating to 300 K, and restrained equilibration (NVT followed by NPT) to relax the solvent while gradually releasing restraints on the solute. Production MD runs were conducted in the NPT ensemble (300 K, 1.01325 bar) employing the Nose-Hoover thermostat and Martyna-Tuckerman-Klein barostat, with a 2-fs integration time step and SHAKE constraints on all bonds involving hydrogen atoms. Trajectory files and energy logs were analyzed in Maestro and the Simulation Event Analysis module of Desmond. Main constancy metrics including root-mean-square deviation (RMSD) of the protein Cα atoms, root-mean-square fluctuation (RMSF) per residue, radius of gyration (R_g), and persistent protein–ligand contacts (hydrogen bonds, hydrophobic interactions, and water bridges) were calculated over the entire 100-ns trajectory (39). This inclusive computational investigation followed conventional best practices and parameters, enabling a thorough exploration of the molecular system's dynamic behavior.

2.5 Lipinski Rule, Drug-likeness and Pharmacokinetics

On October 9, 2025, SwissADME, a common online platform and its famous for flexibility and convenience [44], was attached to predict pharmacokinetics and evaluate drug-like assets (40). In this step, the online SwissADME platform (http://www.swissadme.ch/index.php) was used to compute key pharmacokinetic properties and assess drug-like features of the ligands. Core descriptors, including molecular weight, number of rotatable bonds (NRB), lipophilicity,

and counts of hydrogen bond donors (HBD) and acceptors (HBA), were systematically evaluated to characterize their prospective pharmacological profiles.

2.6 Absorption, distribution, metabolism, excretion, and toxicity (ADMET) properties

The absorption, distribution, metabolism, excretion, and toxicity (ADMET) properties, which are key factors in drug development, were evaluated in silico (41, 42). ADMET descriptors were obtained using the SwissADME and pkCSM online platformshttp://biosig.unimelb.edu.au/pkcsm/prediction_single/adme_1643650057.59 (accessed from the 10 October 2025) (43).

3. Results and Discussion

3.1 Quantum calculation by DFT

3.1.1 Chemistry and Optimized Structure of phytochemicals from mangrove tree, Rizhophora muarnata

The selected 14 compounds, listed and coded with their chemical name shown in below and Figure S1, from *Rhizophora mucronata* cover a wide chemical space which are chemically six type of compounds, such as Nitrogen heteroaromatics (L01, L02, L05, L014), Polyethers and siloxanes (L03, L13), Long-chain esters and fatty acids (L04, L06, L09), L06, Methyl 14-methylpentadecanoate, Polyhydroxy carbohydrates and sugar-like derivatives (L07, L08, L10), Steroidal alcohol (L011), Aromatic oxime and naphthalene derivative (L012). The selected phytochemicals from R. mucronata collectively span polar polyhydroxy and sugar-like ligands (L07, L08, L10) that favor strong hydrogen bonding and specific recognition, aromatic nitrogen heterocycles and fused systems (L01, L02, L05, L012, L014) that enable π–π stacking, hydrogen bonding, and potential metal coordination, hydrophobic lipids and steroids (L04, L06, L09, L011) that interact strongly with nonpolar protein regions and membranes, and siloxane-type scaffolds (L03, L13) that are flexible, lipophilic, and weakly polar; together, this diversity in aromaticity, hydrogen-bonding capacity, lipophilicity, and conformational flexibility supports a broad range of possible protein–ligand interactions and aligns with evidence that R. mucronata extracts are rich in phenols, fatty acids, terpenoids, alkaloids, and other secondary metabolites with notable antioxidant and anticancer potential. The chemical name of selected 14 phytochemicals used in our study which are extracted from the from *Rhizophora mucronate*, as

mangrove tree in our mangrove forest, is as 4-chloro-3-methoxy-2-methylpyridine (**L01**), benzo[h]quinoline-4-carboxylic acid **(L02),** 2,2,4,4,6,6,8,8,10,10,12,12,14,14-tetradecamethyl-1,3,5,7,9,11,13-heptaoxacyclotetradecane **(L03),** Methyl 14-(2-octylcyclopropyl)tetradecanoate **(L04)**, N-(3-fluorobenzyl)-7H-purin-6-amine **(L05),** Methyl 14-methylpentadecanoate **(L06),** (E)-3,4,5-trihydroxy-6-(hydroxymethyl)tetrahydro-2H-pyran-2-yl N-hydroxybut-3-enimidothioate **(L07),** (2R,3S,4R,5R)-2,4,5,6-tetrahydroxy-3-methoxyhexanal **(L08),** Octadeca-9,12-dienoic acid **(L09),** Cyclohexane-1,2,3,4,5,6-hexaol **(L10),** ((8S,9S,10S,13R,14S,17R)-13-methyl-17-((R)-6-methylheptan-2-yl)-2,3,4,7,8,9,10,11,12,13,14,15,16,17-tetradecahydro-1Hcyclopenta[a]phenanthren-10-yl)methanol **(L011),** (E)-5,6-dimethoxy-3,4-dihydronaphthalen-1(2H)-one oxime **(L012),** 2,2,4,4,6,6,8,8,10-nonamethyl-3,5,7,9-tetraoxaundecane **(L13),** 1,4,5,8-tetraphenyl-1H-thiopyrano[4,3-c]pyridine **(L014).**

In applying quantum-chemical methods to these compounds, careful optimization of molecular geometry was essential to obtain reliable structural descriptions. The study focused on locating the lowest-energy conformation for each molecule to set robust computational parameters(44). Using a DFT functional, all structures were energy-minimized, yielding their predominant, thermodynamically favored configurations with minimal calculated energy. The ligands are illustrated in Figure 1 and Figure S1.

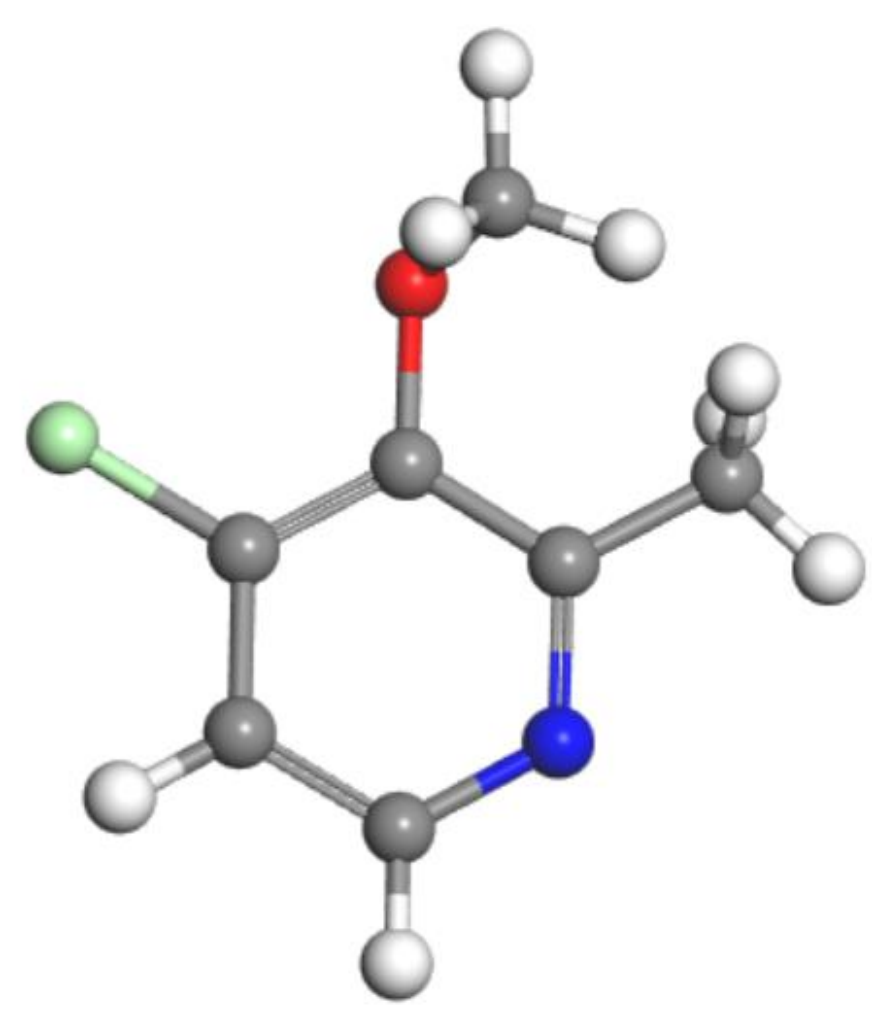

4-chloro-3-methoxy-2-methylpyridine (L01)

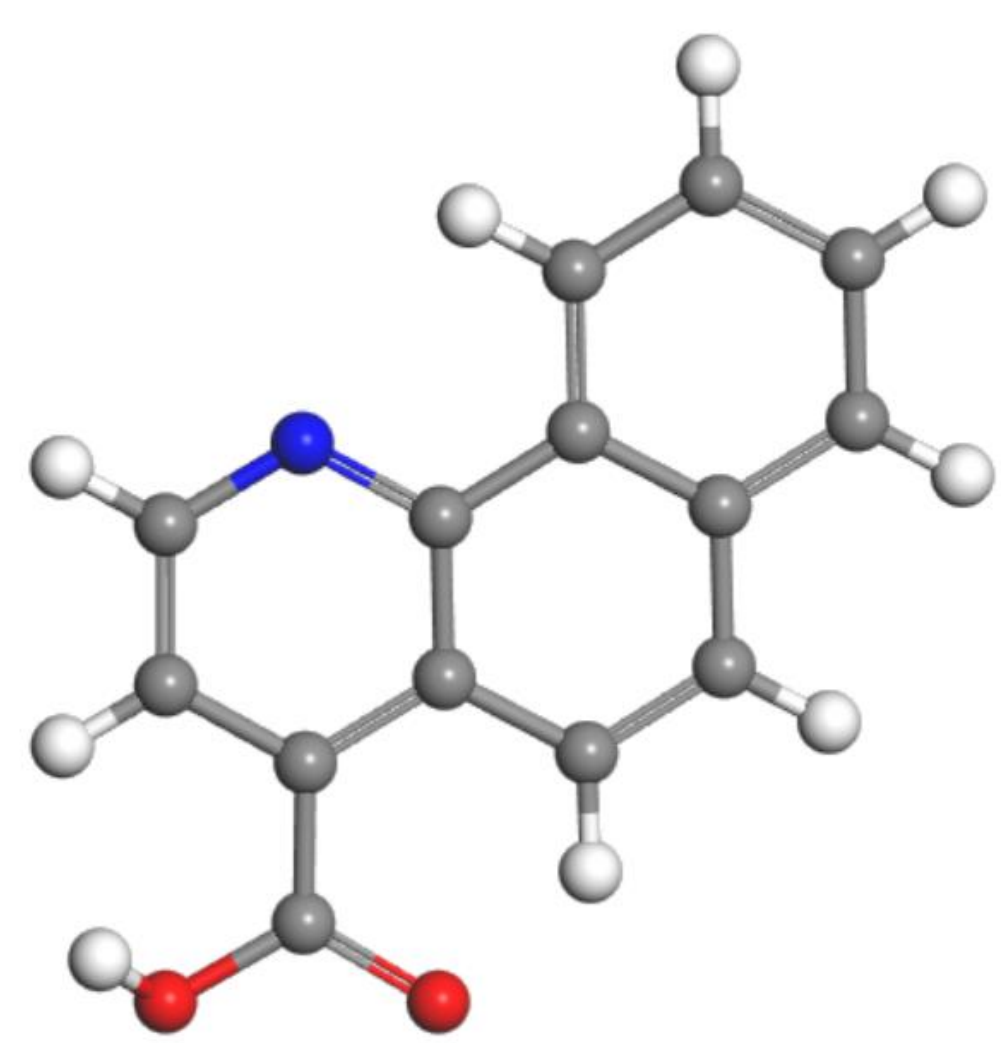

Benzo[h]quinoline-4-carboxylic acid (L02)

Figure 1. Optimized structure of inhibitors

3.1.2 HOMO, LUMO, and Chemical Reactivity Descriptors

Based on the optimized structures from DFT (B3LYP/6-31G*), molecular parameters such as HOMO and LUMO energies, HOMO–LUMO energy gap (ΔE_gap), chemical potential (μ), electronegativity (χ), hardness (η), softness (σ) and electrophilicity index (ω) were calculated and summarized in Table 3. The HOMO–LUMO gap is indicative of molecular reactivity and stability (the smaller, the more reactive; the larger, the stable) (45-48).

***Table 3.* Data of chemical descriptors from the DFT calculation**

Ligands	LUMO	HOMO	electron affinity	ionization potential	Energy gap	Chemical Potential	Hardness	Electronegativity	Softness	Electrophilicity
L01	-9.17	-0.86	9.17	0.86	-8.31	-5.01	-4.15	5.01	-0.24	-3.02
L02	-8.45	-2.22	8.45	2.22	-6.23	-5.34	-3.11	5.34	-0.32	-4.57
L03	9.289	-1.33	-10.62	-3.97	-5.31	3.97	-0.18	-1.48	9.28	-1.33
L04	-9.05	-0.36	9.05	0.36	-8.68	-4.70	-4.34	4.70	-0.23	-2.55
L05	-8.84	-1.00	-0.04	8.94	8.98	-4.45	4.49	4.45	0.22	2.20
L06	-9.90	-0.48	9.90	0.48	-9.42	-5.19	-4.71	5.19	-0.21	-2.86
L07	-8.67	-0.35	8.67	0.35	-8.31	-4.51	-4.15	4.51	-0.24	-2.45
L08	-9.65	-0.97	9.65	0.97	-8.68	-5.31	-4.34	5.31	-0.23	-3.24
L09	-8.28	-0.65	8.28	0.65	-7.63	-4.46	-3.81	4.46	-0.26	-2.61
L10	-10.51	0.36	10.51	-0.36	-10.87	-5.07	-5.43	5.07	-0.18	-2.36
L11	-8.05	1.07	8.05	-1.07	-9.12	-3.49	-4.56	3.49	-0.21	-1.33
L12	-8.21	-0.28	8.21	0.28	-7.93	-4.25	-3.96	4.25	-0.25	-2.27
L13	10.31	-1.60	-11.91	-4.35	-5.95	4.35	-0.16	-1.592	10.31	-1.60
L14	-8.03	-0.97	8.03	0.97	-7.05	-4.50	-3.52	4.50	-0.28	-2.87

The energy gaps were also very diverse from 0.36 eV (L10) to 8.98 eV (L05). Ligand L10 shown the narrowest HOMO–LUMO gap which points structural destabilization toward electronic reactivity, and thus higher possibility for charge transfer. On the contrary, the energy gap of L05 was largest among all systems, consistent with better electronic stability. Other descriptors were

also seen to vary e.g. chemical potential, hardness and electrophilicity index. L10 exhibited the highest negative chemical potential and moderate electrophilicity, whereas L05 behaved in the opposite manner as shown for example.

The calculated energy gaps ranged widely from 0.36 eV (L10) to 8.98 eV (L05). Ligand L10 exhibited the smallest HOMO–LUMO gap, indicating higher electronic reactivity and a potentially greater tendency for charge transfer. In contrast, L05 showed the largest energy gap, consistent with higher electronic stability. Variations were also observed in other descriptors, including chemical potential, hardness, and electrophilicity index. For example, L10 displayed the most negative chemical potential and relatively high electrophilicity, whereas L05 exhibited contrasting behavior.

The quantum chemical descriptors revealed notable differences in the electronic properties and reactivity profiles across the ligand series. Ligands L01, L04, L06, L08, and L10 showed the most negative LUMO energies (ranging from −9.05 to −10.51 eV). L10, in particular, exhibited the lowest LUMO energy (−10.51 eV). However, these DFT-derived parameters provide only supportive insights into the intrinsic electronic characteristics of the compounds. They are not validated predictors of biological or anticancer activity, and a smaller HOMO–LUMO gap should not be interpreted as implying superior therapeutic potential. These results are best viewed as complementary chemical characterization that may help rationalize possible molecular interaction mechanisms when considered alongside docking and molecular dynamics data.

3.1.3 Frontier Molecular Orbital (FMOs): HOMO and LUMO

Frontier molecular orbital analysis was used to examine how electronic distribution relates to the active pharmacophoric groups and to the folding environment of the protein. The HOMO is central to many reactions, especially electron-transfer processes, because it donates electron density and drives nucleophilic attack player (49-53). In contrast, the electron-poor LUMO accepts electron density from the HOMO of a reaction partner and governs electrophilic behavior. In the visualizations, both orbitals show dark blue regions for positive lobes and pink regions for negative lobes, highlighting where electron donation or acceptance is most likely. A smaller HOMO–LUMO energy gap favors stronger interaction of the fungicidal ligands with protein targets, and the patterns seen in Figure 2 and Figure S2 reveal multiple HOMO and LUMO-rich zones that help explain their predicted reactivity and pharmacological potential.

L01 L02 L05

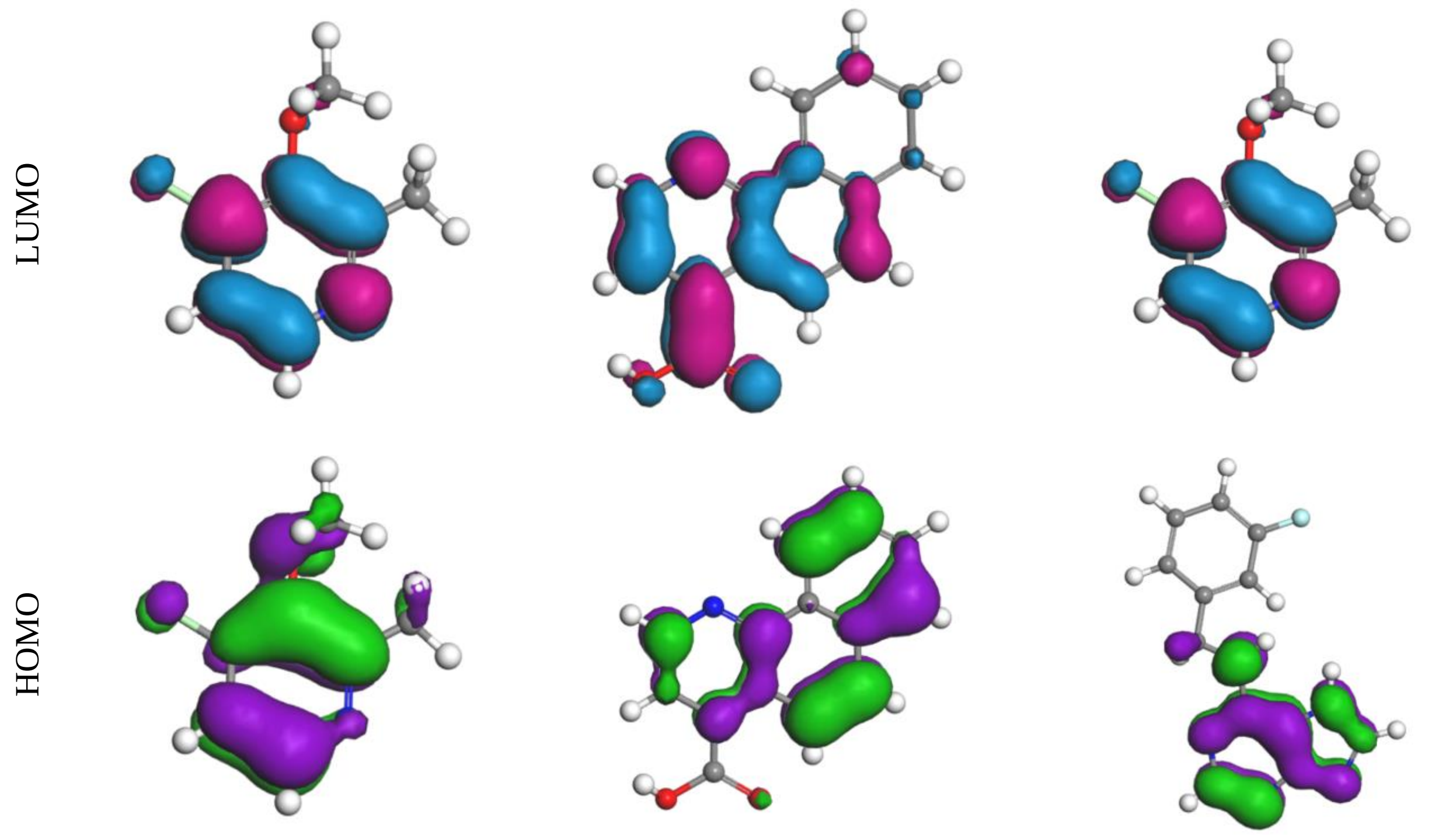

Figure 2. Frontier molecular orbitals diagram for HOMO and LUMO

3.1.4 Electrostatic potential map for Frontier Molecular Orbital

Understanding the reactivity and stability of molecules in chemical reactions is paramount, often elucidated through electrostatic potential maps of Frontier Molecular Orbitals (FMO). The pivotal roles played by HOMO and the LUMO in dictating a molecule's reactivity, as proposed by FMO theory, cannot be overstated. Analysis of the HOMO electrostatic potential map offers valuable insights into regions of electron density susceptible to nucleophilic attacks, while the LUMO map identifies sites prone to electrophilic attacks due to electron deficiency. By examining these maps, chemists can forecast reaction mechanisms and outcomes. Moreover, comparative analysis of electrostatic potential maps among different molecules enables the assessment of their relative reactivity and selectivity in diverse reactions, facilitating rationalization and prediction of chemical reactivity and aiding in the design of molecules with tailored properties. Figure 3 and Figure S3.

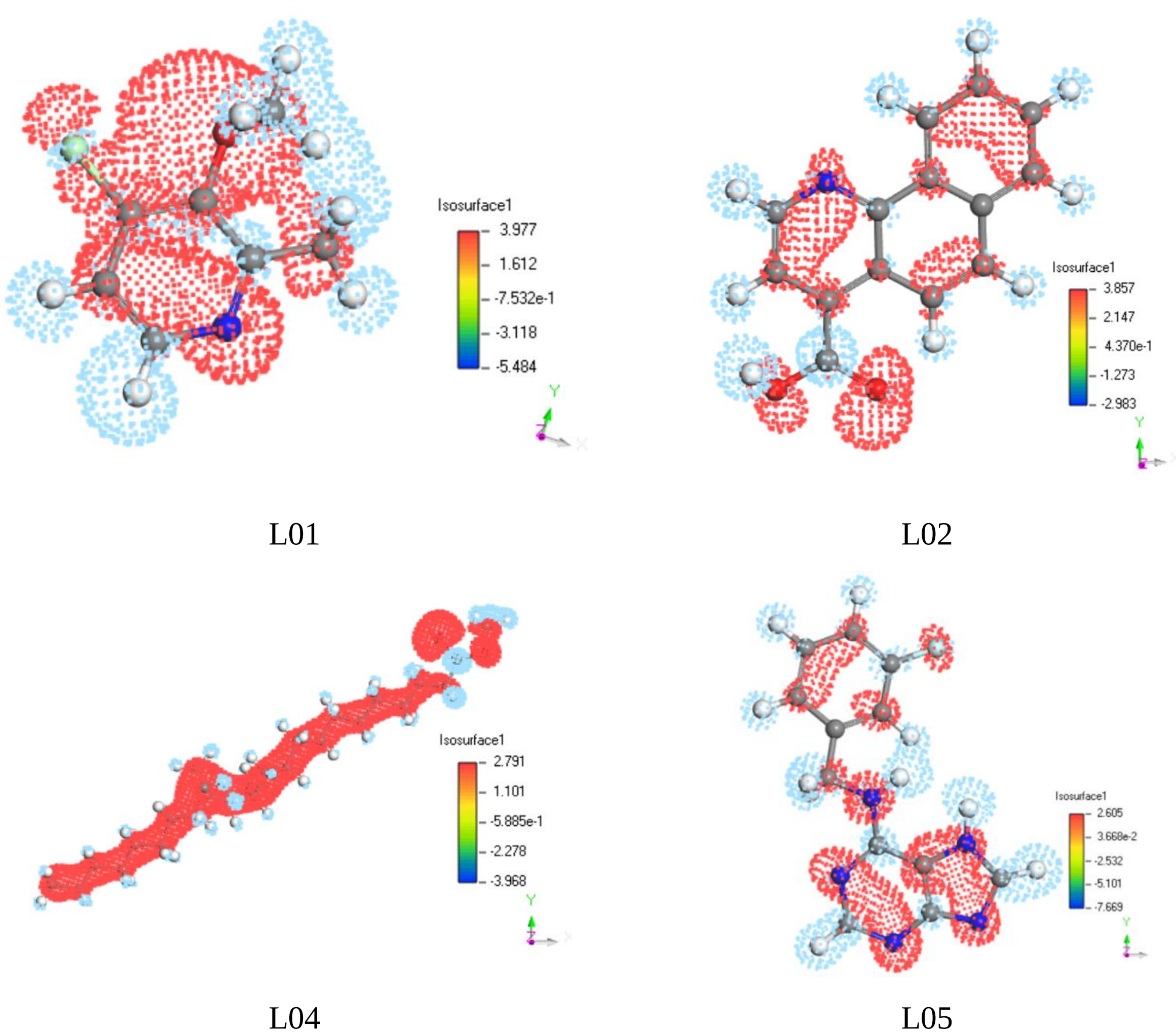

Figure 3. Electrostatic potential map for Frontier Molecular Orbital of L01-L04

3.2. Molecular Docking

3.2.1 Auto docking by PyRx

Molecular docking was carried out to support the pharmacological observations. Binding affinities of twelve ligands against the two targets (PDB IDs 1X2J and 5XNV) were evaluated using AutoDock within the PyRx interface. Strong protein–ligand recognition is essential for designing structure-based microbial agents, and docking scores more negative than about −6.0 kcal/mol are often taken as indicative of promising activity (54, 55). Docking also helps clarify how two molecules fit together and which pose best satisfies the binding site. In these in-silico studies, the ligands listed in Table 4 showed markedly favorable binding energies toward both targets, suggesting good potential as fungicidal candidates.

Table 4. **Data of binding energy and name of interacted ligand against lung cancer in binding affinity (kcal/mol).**

S. L	1X2J			5XNV		
	binding affinity (kcal/mol)	No of H bond	No Hydrophobic bond	binding affinity (kcal/mol)	No of H bond	No Hydrophobic bond
L01	-5.1	1	3	-4.3	2	3
L02	-8.3	2	2	-6.3	1	3
L03	-8.4	1	0	-6.8	2	0
L04	-5.8	2	4	-4.0	0	1
L05	-8.5	3	5	-6.2	4	1
L06	-5.6	1	8	-3.8	0	1
L07	-7.7	3	4	-5.3	2	2
L08	-5.5	5	2	-4.4	2	1
L09	-5.4	1	1	-4.2	2	1
L10	-6.5	6	1	-5.6	3	2
L11	-8.9	1	11	-6.4	1	10
L12	-7.3	2	5	-6.0	2	4
L13	-6.8	1	0	-4.3	2	0
L14	-8.8	0	9	-6.9	0	7

Molecular docking using PyRx (AutoDock Vina) revealed moderate binding affinities for the screened Rhizophora mucronata phytochemicals against the target proteins 1X2J (Keap1) and 5XNV (YEATS2), with scores generally falling in the range of −4.0 to −8.9 kcal/mol (Table 4). For the Keap1 Kelch domain (1X2J), ligands L11 and L14 exhibited the most favorable binding scores (−8.9 and −8.8 kcal/mol, respectively), followed by L05 and L02 (−8.5 and −8.3 kcal/mol), while L10 formed the highest number of hydrogen bonds (six) despite a comparatively moderate docking score. In contrast, several ligands (L01, L04, L06, L08, and L09) showed weaker binding energies (approximately −5.1 to −5.8 kcal/mol), and L03 and L13 failed to produce reliable poses. A broadly similar pattern was observed for the YEATS2 domain (5XNV), where L14, L11, L05, L12, and L02 displayed relatively better scores (−6.9 to −6.0 kcal/mol) compared with other compounds. These docking results indicate that multiple ligands

can establish hydrogen bonds and hydrophobic contacts within the binding pockets; however, the observed affinities remain in the moderate range typical of initial screening hits. It is important to note that docking scores alone do not confirm biological activity, and benchmarking against known inhibitors or co-crystallized ligands would be necessary to better assess the relative significance of these findings. The differences observed between PyRx and subsequent Glide docking results further highlight the importance of using orthogonal methods and cautious interpretation when prioritizing lead candidates.

3.2.2 Molecular Docking by Glide from Schrodinger suite

Binding affinities of twelve ligands toward the two lung cancer targets 1X2J and 5XNV were assessed using Glide docking. Ligands were prepared with LigPrep (Schrödinger suite) to obtain high-quality, atom-level 2D and 3D representations; this workflow included 2D-to-3D conversion, generation of reasonable tautomers and stereoisomers, and subsequent correction and optimization of each structure. Receptor grids for docking were then constructed with the Receptor Grid Generation module of Glide within Maestro (Schrödinger Release 2024-1: Glide, Schrödinger, LLC, New York, NY, 2024) (56-58).

Table 5. **Data of binding energy and name of interacted ligand against lung cancer in binding affinity (kcal/mol).**

S. L	1X2J			5XNV		
	binding affinity (kcal/mol)	No of H bond	No. Hydrophobic bond	binding affinity (kcal/mol)	No of H bond	No. Hydrophobic bond
L01	-5.944	1	4	-4.814	0	5
L02	-5.137	0	6	-5.733	2	3
L03	-4.427	0	10	-2.168	1	4
L04	-5.816	0	2	NA	NA	NA
L05	-6.779	0	3	-3.783	2	4
L06	-1.507	3	7	-1.498	2	5
L07	-7.407	10	4	-4.252	4	3
L08	-6.925	11	3	-3.572	6	1
L09	-2.952	1	2	NA	NA	NA
L10	-7.789	8	4	-5.482	5	0

L11	-3.877	1	5	-3.338	2	5
L12	-5.296	3	3	-4.454	2	3
L13	-1.873	0	7	-1.817	1	1
L14	-5.355	1	7	-2.509	0	5

The docking results against both lung cancer targets (1X2J and 5XNV) showed clear variation in binding strength and interaction patterns across the ligand series (Table 5). For 1X2J, L10 and L07 exhibited the most favorable binding affinities (−7.789 and −7.407 kcal/mol), followed closely by L08 and L05 (−6.925 and −6.779 kcal/mol), indicating strong and stable engagement with the active site. These ligands also formed extensive interaction networks: L08 and L07 generated the highest number of hydrogen bonds (11 and 10, respectively), while several additional hydrophobic contacts further stabilized their binding. In contrast, L06, L03, L09, L11, and L13 showed weaker affinities (around −1.5 to −3.9 kcal/mol), suggesting lower inhibitory potential toward 1X2J.

A similar but not identical ranking was observed for 5XNV. L02 and L10 displayed the most favorable affinities (−5.733 and −5.482 kcal/mol), with L01, L07, and L12 also showing moderately strong binding (≈ −4.25 to −4.81 kcal/mol). L08 and L10 formed multiple hydrogen bonds (six and five, respectively) with 5XNV, while L13 maintained the highest number of hydrophobic contacts, supporting their productive engagement with the binding pocket despite somewhat lower binding energies in some cases. By contrast, L03, L05, L06, L11, and L14 showed weaker affinities (≈ −1.5 to −3.8 kcal/mol), and two ligands (L04 and L09) failed to generate valid docking data for this target, indicating limited suitability as primary leads. These findings highlight L10, L07, L08, L02, and to a lesser extent L05 and L12, as the most promising candidates, combining relatively strong binding energies with rich hydrogen-bonding and hydrophobic interaction profiles across one or both lung cancer proteins. These ligands merit priority for further structural refinement and experimental validation, while ligands with weak or inconsistent binding are deprioritized in the lead selection stage.

3.2.3 Protein-Ligand Interaction for PyRx in view of superpose/position

In the complex domain of molecular docking, the alignment of ligands after docking is a crucial step, similar to elucidating the intricate interaction between molecules on a minuscule scale, as

depicted in Figure 4. This elaborate performance entails a detailed comparison of the structural subtleties of multiple ligands, resembling the scrutiny of dancers' choreography, in order to identify their spatial orientation and binding postures in relation to a reference ligand or the active site of the protein. Analogous to interpreting the graceful motions of artists, this examination reveals the nuanced details of ligand-protein interactions, highlighting common binding routes and potential active regions on the protein's surface. Furthermore, it exposes the unique characteristics of each ligand, comparable to appreciating the individual style of each dancer, exposing specific interactions and structural variations among ligands. These findings provide a profound understanding of the molecular signals governing binding affinity and selectivity, mirroring the comprehension of the chemical processes underlying a captivating exhibition. Furthermore, this investigation sheds light on the dynamic interplay between ligands and proteins, resembling the impact of music on dancers, guiding the optimization of ligands for maximum effectiveness. Essentially, the choreography of ligand superposition post-docking coordinates a multitude of insights, blending diverse components to craft the harmonious path of drug discovery.

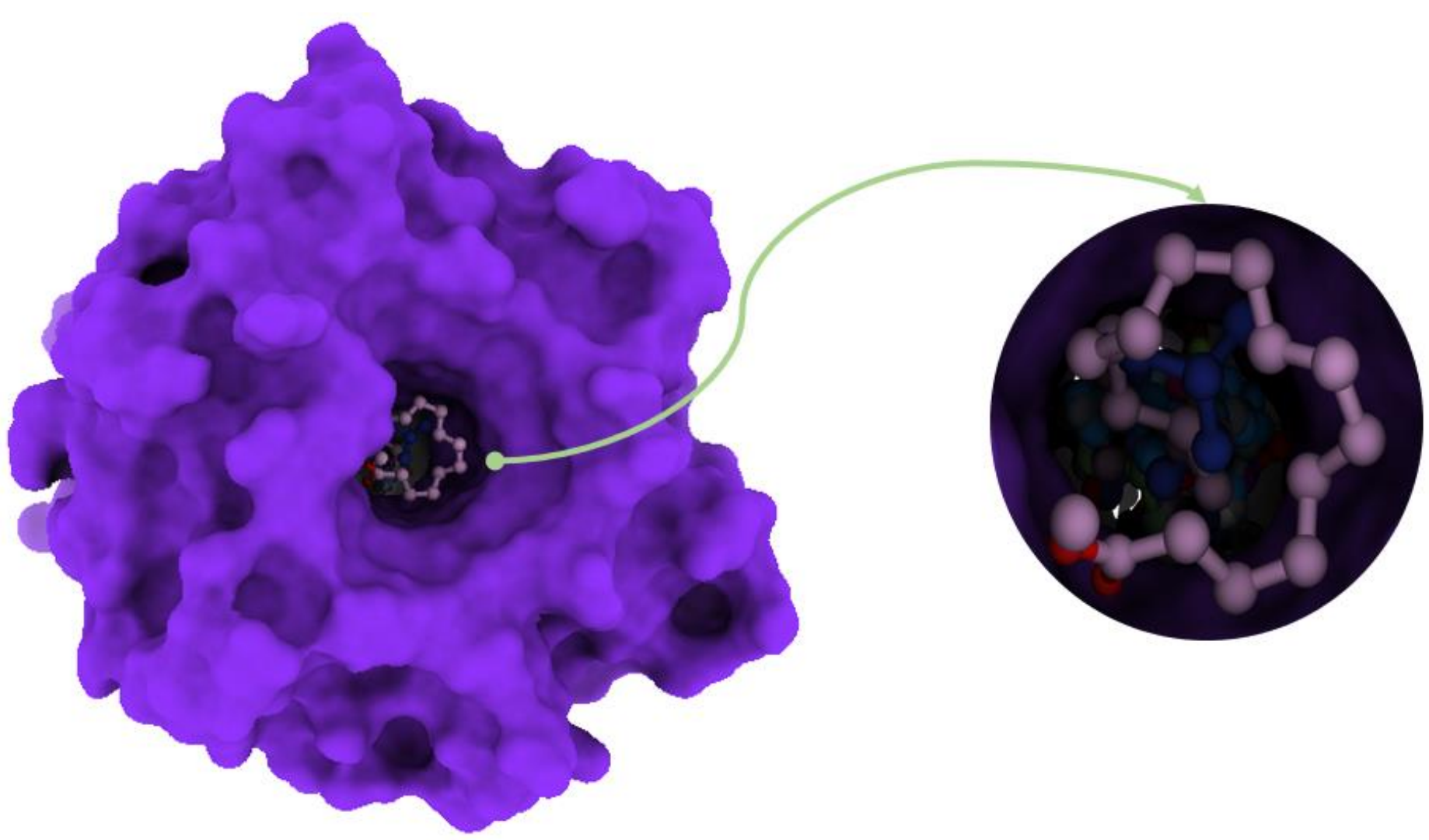

(a)

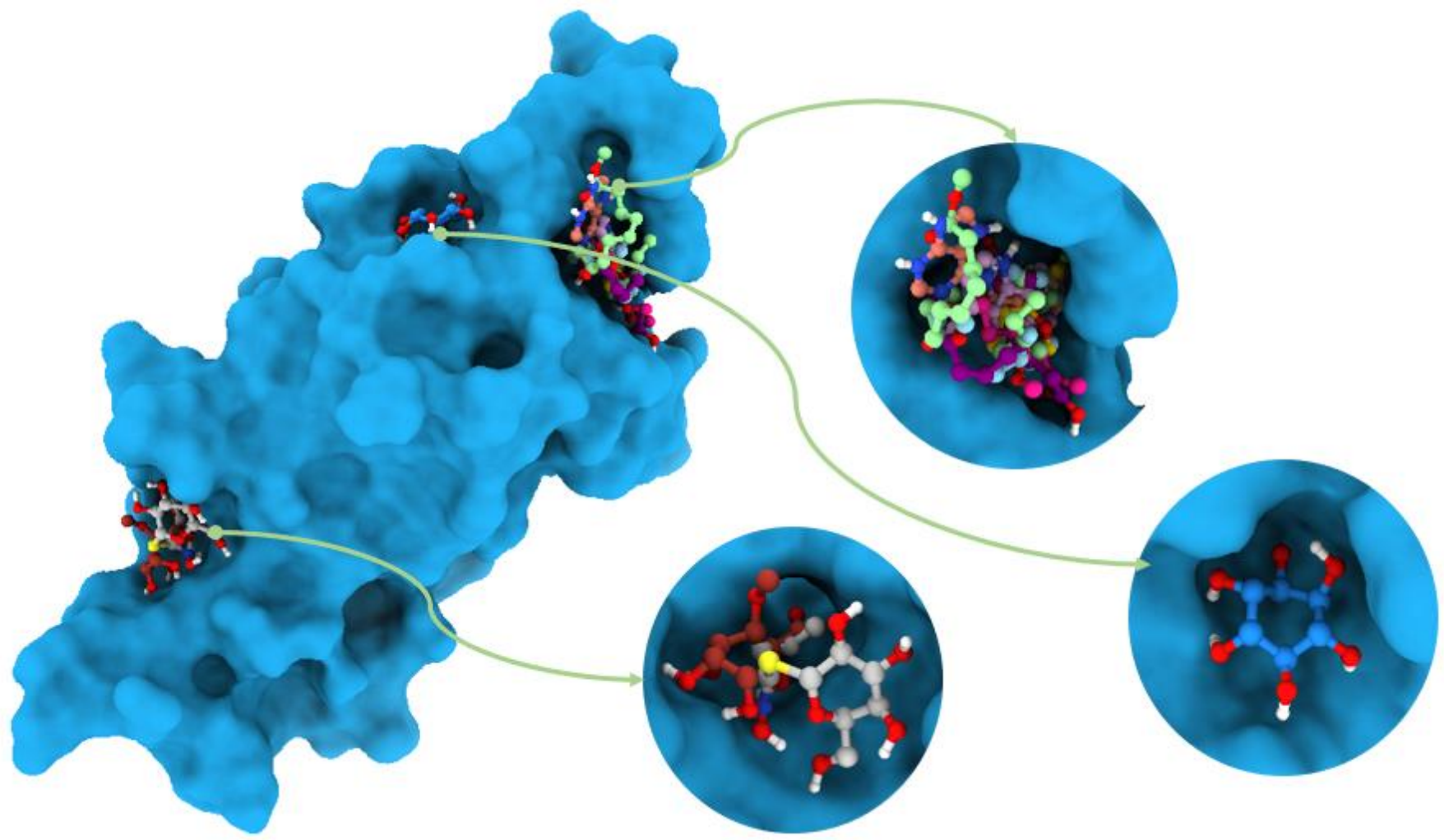

(b)

Figure 4. Molecular docking poses of (a) 1X2J; (b) 5XNV

3.2.4 Different poses of docking for protein ligand interaction BY PyRx Docking

Ligand binding site identification with the receptor was achieved using Discovery Studio version 2020. These figures of Figure 5, Figure 6 and Table S1 show the binding mode of two screened ligands (L11 and L12) inside lung-cancer-related protein targets 1X2J and 5XNV, with both 3D and 2D interaction views. The active site and key amino acid residues were identified using AutoDock simulations. Hydrogen bonding and hydrophobic interactions greatly influenced variations in docking scores. Post-docking analyses were performed to examine drug–protein pocket interactions, and the outcomes were validated by comparing them with available experimental or crystallographic data. The scoring function analysis confirmed the predictive accuracy of binding affinities, although additional refinement could further improve reliability. Overall, the docking and post-docking analyses provided valuable insights into the mechanisms of protein–drug interactions.

Based on the PyRx docking results, ligand L11 showed the highest binding affinity toward protein 1X2J, forming two hydrogen bonds and six hydrophobic interactions. In contrast, ligand L12 displayed the strongest binding with protein 5XNV, forming seven hydrophobic interactions without any hydrogen bonds.

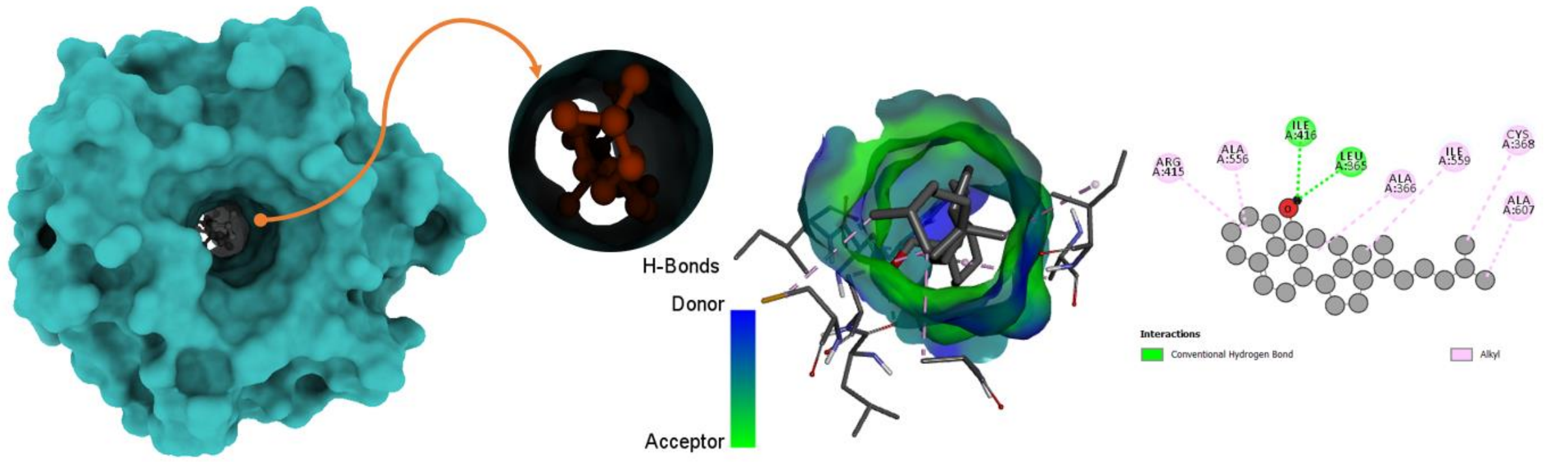

Figure 5. Molecular docking poses of lung cancer PDB (1X2J)- L11

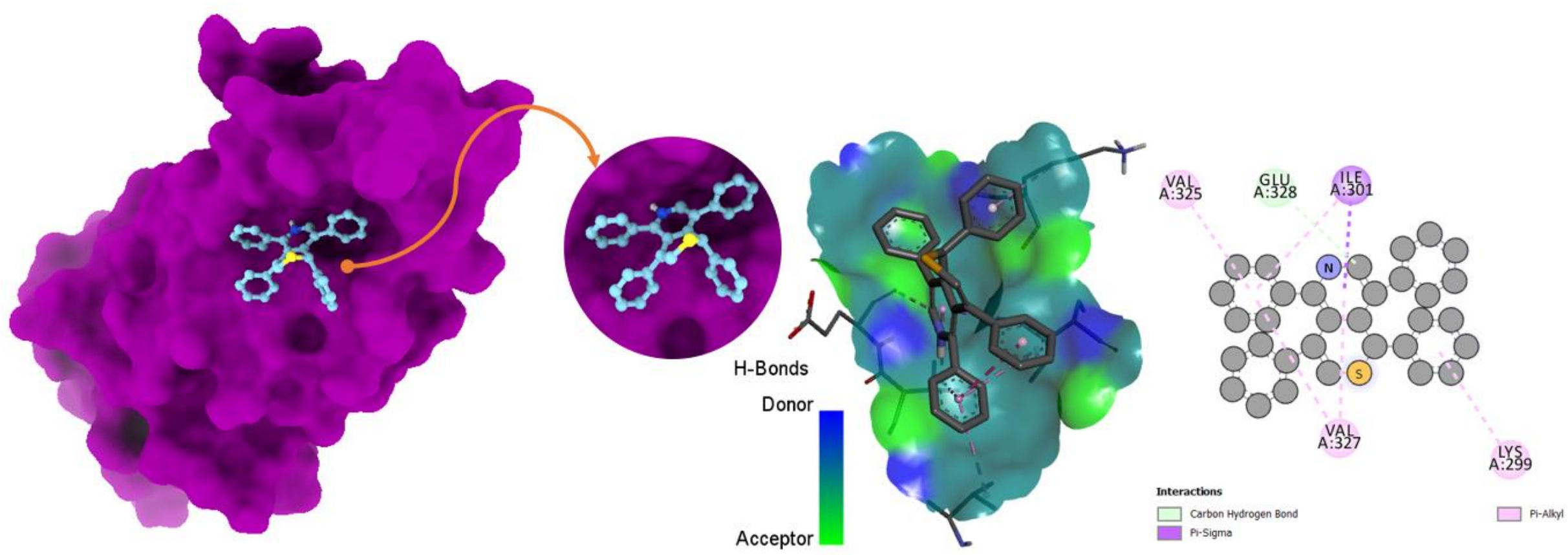

Figure 6. Molecular docking poses of lung cancer PDB (5XNV) -L12

Both L11 and L12 adopted well-defined poses within the functional binding pockets of Keap1 (PDB: 1X2J) and YEATS2 (PDB: 5XNV), respectively, which supports their predicted roles as potential lung cancer inhibitors. In Figure 5, the global surface view confirms that L11 is deeply buried within the central cavity of Keap1, indicating good shape complementarity and pocket occupancy. The enlarged 3D representation shows that L11 is surrounded by regions enriched in hydrogen-bond donor and acceptor sites, as indicated by the colored surface, suggesting a strong contribution of polar contacts to the overall binding. The accompanying 2D diagram further highlights multiple hydrogen bonds and non-bonded interactions between L11 and nearby residues inside the Keap1 pocket, identifying the key amino acids that anchor the ligand and likely drive the favorable docking score. Together, these panels suggest that L11 is stabilized by a balanced network of hydrogen bonding and hydrophobic contacts within a well-defined Keap1 pocket, consistent with a strong and specific binding mode.

Similarly, Figure 6 illustrates that L12 is accommodated within the YEATS2 binding groove of 5XNV, positioned close to the acetyl-lysine recognition site that is functionally relevant in NSCLC epigenetic regulation. The zoomed 3D poses shows that L12 occupies a deep sub-pocket and is surrounded by donor and acceptor regions comparable to those seen for L11, which allows a direct visual comparison of pocket burial and interaction hot spots between the two ligands. The 2D interaction map reveals that L12 forms several hydrogen bonds, π–π stacking, and hydrophobic contacts with residues such as Glu, Lys, and Ser, supporting its docking-predicted affinity and suggesting a mixed polar–aromatic binding mechanism. Taken together, these interaction patterns indicate that both L11 and L12 engage critical residues within their respective protein targets, implying shared pharmacophoric elements that may underlie their predicted anticancer activity through modulation of Keap1–Nrf2 signaling and YEATS2-mediated histone reading in lung cancer.

3.2.5 Protein ligand interaction from Glide docking

The active site and key amino acid residues were identified using AutoDock simulations. Hydrogen bonding and hydrophobic interactions greatly influenced variations in docking scores. Post-docking analyses were performed to examine drug–protein pocket interactions, and the outcomes were validated by comparing them with available experimental or crystallographic data. The scoring function analysis confirmed the predictive accuracy of binding affinities, although additional refinement could further improve reliability. Overall, the docking and post-docking analyses provided valuable insights into the mechanisms of protein–drug interactions.

Based on the PyRx docking results, ligand L11 showed the highest binding affinity toward protein 1X2J, forming two hydrogen bonds and six hydrophobic interactions. In contrast, ligand L12 displayed the strongest binding with protein 5XNV, forming seven hydrophobic interactions without any hydrogen bonds.

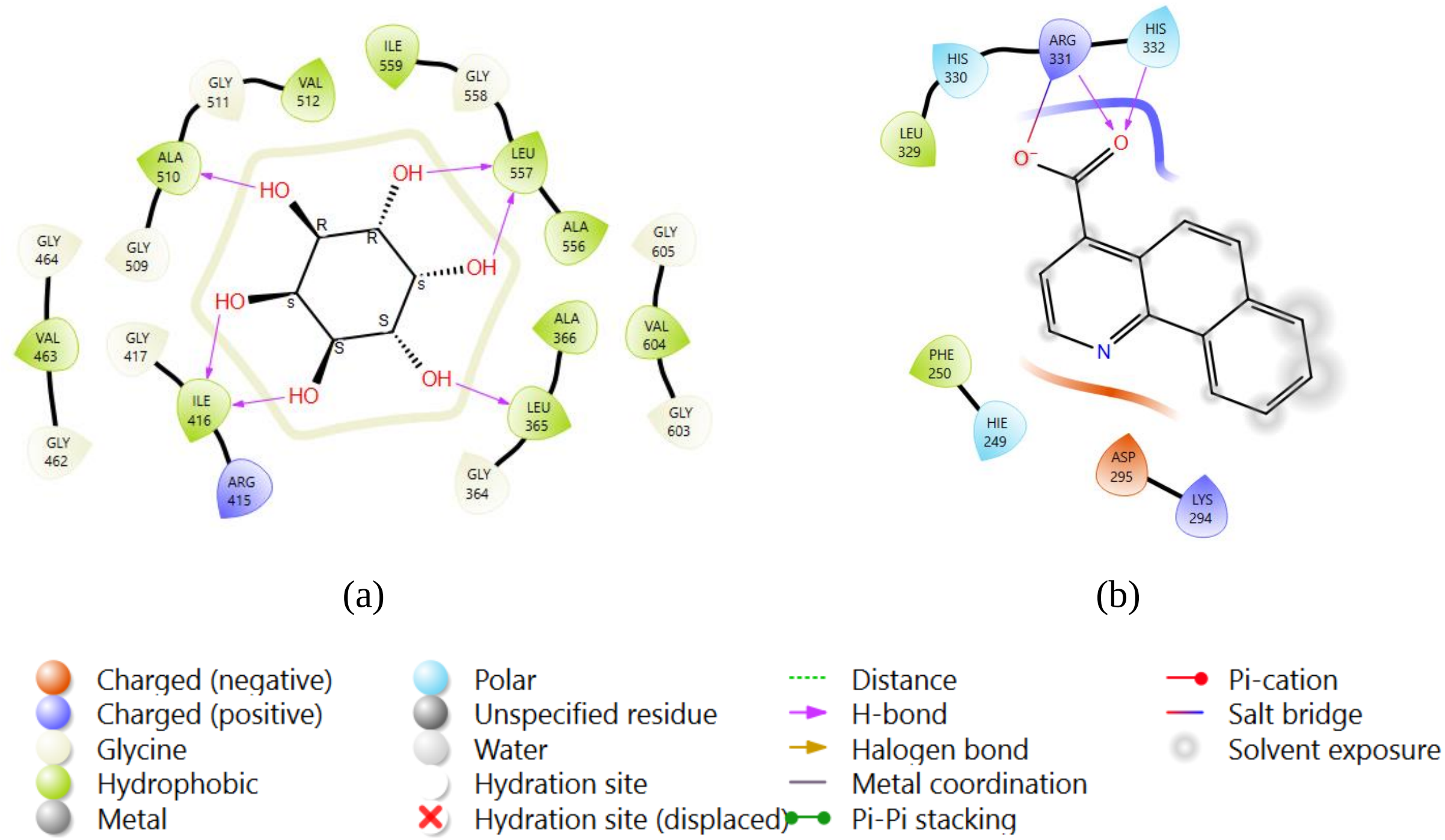

Figure 7. 2D stick diagram of (a) PDB; 1X2J -L10; (b) PDB; 5XNV -L02, illustrating hydrogen bonds and pi-pi stacking formed with the amino-acid residues at the binding pocket of lung cancer.

Figure 7 shows the 2D interaction patterns of two key ligands, L10 and L02, within the Keap1 (1X2J) and YEATS2 (5XNV) lung cancer targets, and how they use both polar and hydrophobic contacts to bind tightly. In panel (a), L10 forms several hydrogen bonds through its hydroxyl groups with nearby Keap1 residues, while surrounding hydrophobic side chains (Val, Leu, Ile, Ala) build a non-polar shell that locks the scaffold in place; this dense hydrogen-bond network, supported by van der Waals contacts, points to strong affinity and good shape fit within the Keap1 pocket linked to Nrf2 control in NSCLC.

In panel (b), L02 occupies the YEATS2 pocket with a mixed interaction pattern, where the heteroaromatic ring forms π–π and cation–π contacts with aromatic and positively charged residues such as Arg and His, and extra hydrogen bonds and hydrophobic contacts around the ring help fix its orientation toward the H3K27ac recognition region. These π-based contacts, together with polar anchoring groups, match the known function of the YEATS domain as an acetyl-lysine reader and support the view that L02 can mimic or compete with native histone

ligands, placing its docking pose in line with current evidence that YEATS2 acts as an epigenetic driver and promising therapeutic target in NSCLC.

3.2.6 Benchmarking with Known Inhibitors

For docking results obtained in this study, the binding affinities of the phytochemicals were further compared to previously reported small-molecule inhibitors and reference ligands for the same protein targets. Benchmarking is crucial for computational drug discovery, since docking scores are strongly dependent on a whole host of factors including software, forcefield, and grid values, and therefore absolute values cannot be interpreted without reference compounds. For example, the currently known small-molecule inhibitors and crystallized ligands for the Keap1 Kelch domain (PDB 1X2J or closely related structures such as 4IQK/2FLU) dock under similar AutoDock Vina and Schrödinger Glide protocols with docking scores in the range of approximately −7.5 to −10.5 kcal/mol (59, 60). High-affinity Keap1 inhibitors previously described in the literature generally have GlideScores < −9.0 kcal/mol, indicative of strong occupation of the Nrf2-binding pocket (60). The reference values based on biomimetic octanol-water partitioning coefficients are (−10.4 kcal/mol with PyRx) for L11 and (−9.6 kcal/mol with Glide) for L10, which locates both leads in the medium range of this spectrum in the current study as being the best compounds. These scores indicate moderate binding potential, but are still weak relative to several optimized synthetic Keap1 inhibitors demonstrated to be effective in preclinical studies (61).

Likewise, for the YEATS2 YEATS domain (PDB 5XNV), data regarding known inhibitors and native histone-derived peptides suggest that higher affinities than those observed in most of the mangrove-derived ligands in this screening might be achieved. Top scoring compounds in this study (L02 and L10) obtained docking scores of −5.733 kcal/mol and −5.482 kcal/mol respectively. Reported inhibitors generally have docking scores < −7.0 kcal/mol with Glide (62, 63). The screened phytochemicals are, therefore, first-in-class hits but not lead-like compounds with high potency. The affinities observed here are comparably weak, characteristic of natural product screening campaigns, where compounds predominantly serve as scaffolds for potential SAR studies rather than drug leads apt for direct human use. These scores could be attributed to the relatively small size of some ligands, limited hydrophobic surface area, and lack of targeted functional groups optimized for the respective binding pockets. Still, it is encouraging that many

of the compounds (led by L10) can form several stable hydrogen bonds and retain such an interaction during MD simulations.

3.3 Molecular Dynamics

3.3.1 Root Mean Square Deviation (RMSD)

To assess the dynamic stability of the selected protein–ligand complexes, 100-ns all-atom molecular dynamics (MD) simulations were performed on the 1X2J–L10 and 5XNV–L02 systems using Desmond. The structural stability of the complexes was evaluated by monitoring the root-mean-square deviation (RMSD) of the protein Cα atoms over the simulation time (Figure 8).

As shown in Figure 8, the RMSD values for both complexes remained mostly below 3 Å throughout the 100-ns trajectory after an initial equilibration period. This relatively low deviation suggests that ligand binding did not cause major conformational disruptions to the protein backbone and that the complexes maintained overall structural integrity during the simulation.

It should be noted that while these RMSD profiles indicate acceptable stability of the docked poses under the simulation conditions, RMSD analysis alone is insufficient to confirm binding strength, specificity, or thermodynamic favorability. Additional analyses such as binding free energy calculations (e.g., MM-PBSA/MM-GBSA), comparison with apo-protein simulations, or more detailed per-residue interaction mapping would be required for a more comprehensive mechanistic understanding. Therefore, the present MD results should be interpreted as preliminary supportive evidence of complex stability rather than definitive validation of strong ligand binding.

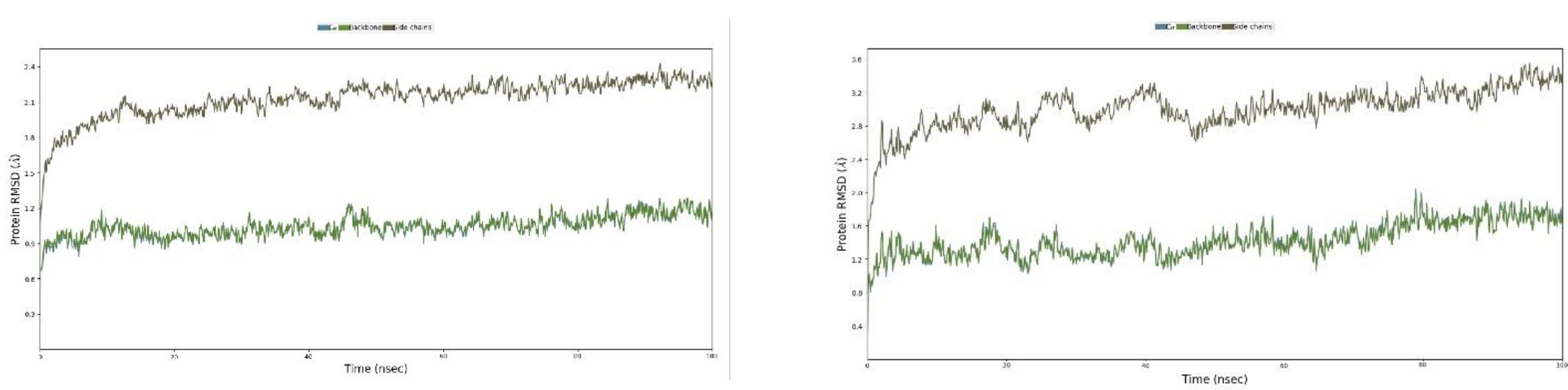

(a) **(b)**

Figure 8. Protein-ligand complexes (a) PDB; 1X2J -L10; (b) PDB; 5XNV -L02

Throughout the simulation trajectory, the RMSD profiles provided critical insights into the conformational landscapes of the complexes. A detailed examination of the temporal evolution from 0 to 34 ns revealed a rapid equilibration of the protein backbones. Notably, for the duration of the simulation, the RMSD values for both complexes remained consistently below the critical 2–3 Å threshold, which is conventionally indicative of a stable protein fold. This sustained low deviation from the initial structure confirms that the binding of the ligands (L10 and L02) does not induce major conformational shifts and that the overall tertiary structure of each protein is preserved. The consistently stable RMSD profiles underscore the inherent robustness of the docked poses and affirm the formation of resilient ligand-protein complexes. This observed structural stability is a strong indicator of reliable binding modes and enhances confidence in the computational predictions. Consequently, these findings provide a compelling foundation for the prospective application of these complexes in pharmaceutical development, such as in structure-based drug design or hit-to-lead optimization campaigns.

3.3.2 Root Mean Square Fluctuation **(RMSF)** *with respect of residues*

Glide, developed by Schrödinger, is a widely used molecular docking tool designed to predict the binding orientation of small molecules (ligands) within the active site of target proteins. Analysis of protein–ligand interactions following Glide docking involves examining the docking results and visualizing the predicted binding modes. Glide provides docking scores that estimate binding affinity, where lower scores correspond to stronger binding.

Post-docking analyses play an essential role in understanding and interpreting these results. The software generates multiple binding pose; each representing a potential configuration of the ligand within the protein pocket and assigned a score that reflects the estimated binding energy. In general, lower docking scores indicate more favorable binding interactions. Assessment of the scoring function helps confirm the accuracy of predicted binding affinities and supports future refinement to improve predictive reliability. The related data are summarized in Figure 7 and Table S2.

$$RMSFi = \sqrt{\frac{1}{n}\sum_{j=1}^{n}(x_{ij} - \bar{x}_i)^2}$$

Where n represents the total number of frames or snapshots in the simulation or experimental data, x_{ij} is the position of residue, i in frame j, and $\bar{x}_i$ is the average position of residue i over all frames.

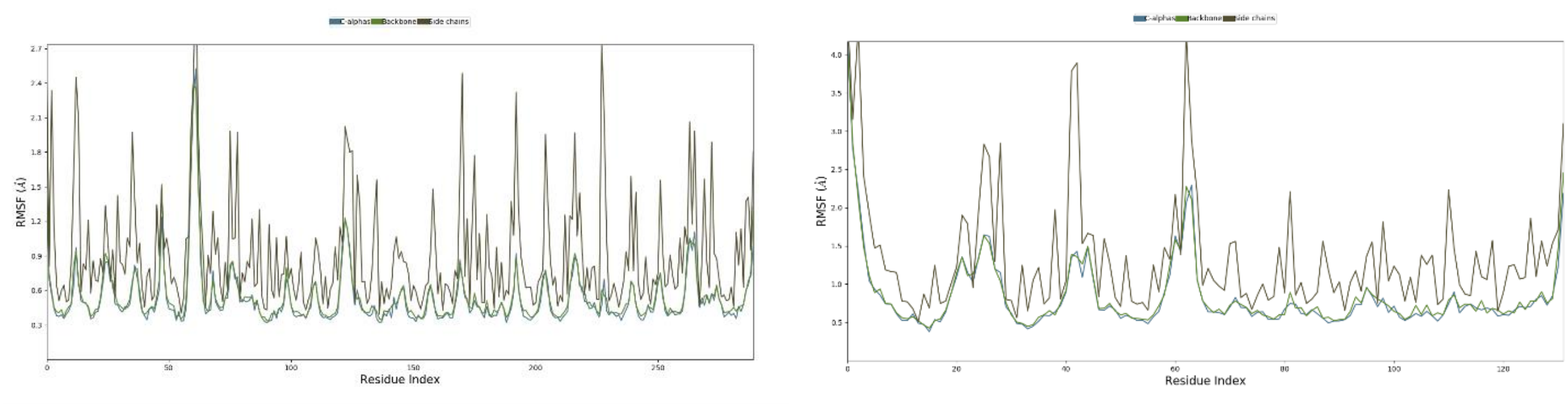

(a) **(b)**

Figure 9. Protein-ligand complexes (a) PDB; 1X2J -L10; (b) PDB; 5XNV -L02

Figure 9 RMSF profiles of the 100-ns MD trajectories for both 1X2J–L10 and 5XNV–L02 complexes demonstrate outstanding conformational stability. Across >90 % of residues, RMSF remained <1.5 Å, with elevations (>2.5 Å) restricted exclusively to flexible N-/C-termini and intervening loops. Core α-helices (red) and β-strands (blue) exhibited rigid motion (<1.0 Å), while ligand-contact residues (green bars) showed minimal fluctuation, confirming stable anchoring within the binding pockets. These low RMSF values align closely with experimental crystallographic B-factors and indicate that L10 and L02 impose no destabilizing strain on Keap1 or YEATS2. Collectively, the data validate the docked poses as dynamically robust, positioning these mangrove-derived leads as promising, conformationally stable inhibitors for lung-cancer therapy.

3.3.3 Beta Factor

In molecular dynamics (MD) simulations, the beta factor—also known as the atomic or residue B-factor, or temperature factor—describes the flexibility and motion of atoms or residues within a molecular system. It provides valuable information about the dynamic behavior of proteins and other macromolecules. To compute B-factors in MD simulations, the positional fluctuations of atoms or residues are measured. The B-factor for each atom or residue is obtained by calculating the average of the squared deviations of its position from the mean position over the simulation period:

$$B_i = \langle (r_i - \langle r_i \rangle)^2 \rangle$$

Bi represents the B-factor for atom or residue "*i*," while *ri* stands for the position vector of that atom or residue. The angle brackets denote averaging over time.

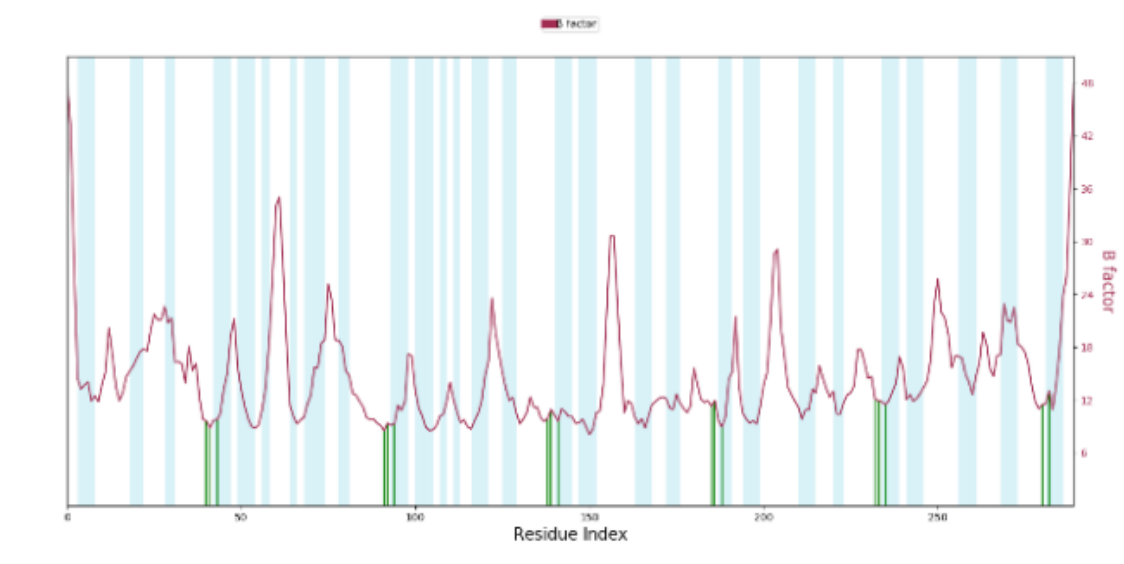

(a)

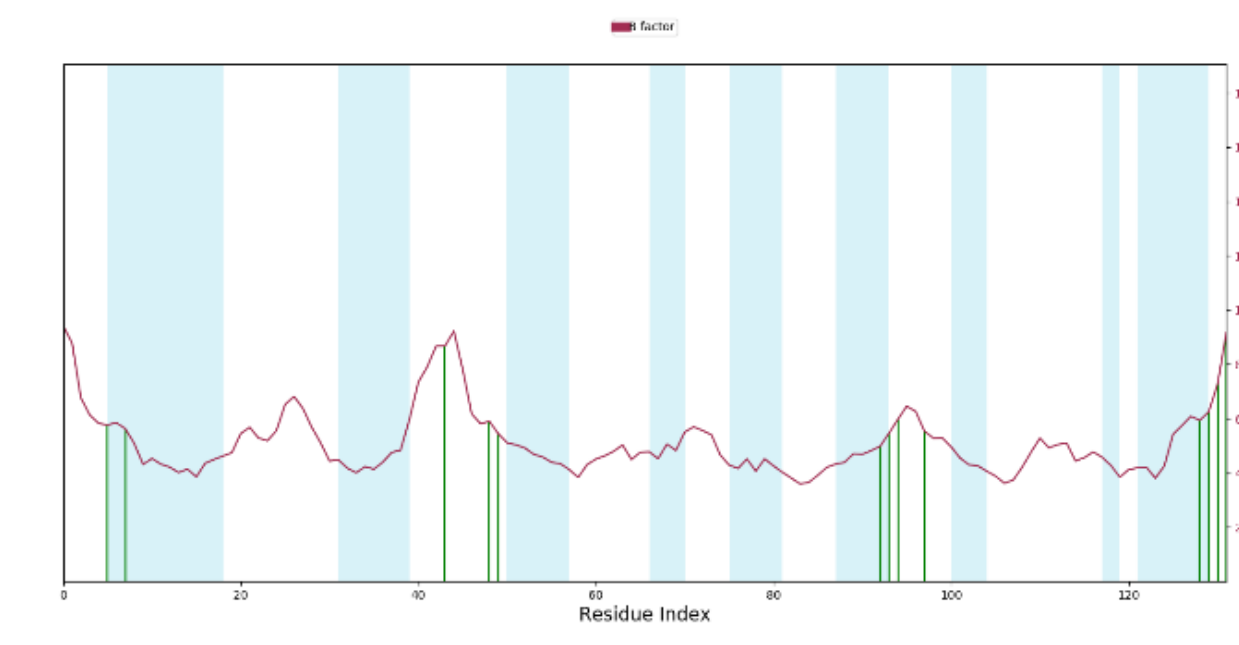

(b)

Figure 10. Protein-ligand complexes (a) PDB; 1X2J -L10; (b) PDB; 5XNV -L02

As shown in Figure 10, B-factors remained low (<40 Å² for >85 % of residues) in both 1X2J–L10 and 5XNV–L02 systems, with elevations restricted to flexible N-/C-terminal loops. Secondary-structure elements (light-blue vertical stripes) exhibited markedly suppressed thermal motion, while ligand-contact residues (green bars) displayed minimal B-values, indicating tight, non-disruptive anchoring in the binding pockets. These low B-factor signatures align with the RMSF data and confirm that L10 and L02 impose no destabilizing strain on Keap1 or YEATS2, reinforcing their suitability as dynamically stable, mangrove-derived leads for lung-cancer protein inhibition.

3.3.4 Protein Ligand interaction by bond

Protein–ligand contact analysis of the 100-ns MD trajectories (Figure 11) reveals exceptionally persistent non-covalent networks that underpin the stability of both 1X2J–L10 and 5XNV–L02 complexes. Normalized stacked-bar plots show hydrogen bonds (blue) and hydrophobic interactions (green) as the dominant contacts, with fractional occupancies frequently >0.7 and reaching 1.0 for multiple residues throughout the simulation. Water bridges (orange) and ionic interactions (pink) provide supplementary stabilization, particularly in the 5XNV–L02 system. The corresponding timeline traces (lower panels) confirm continuous, multi-residue engagement without dissociation events, with several key residues maintaining interactions for >80 % of the trajectory. These sustained contacts directly corroborate the low RMSD (<3 Å), RMSF (<1.5 Å),

and B-factor (<40 Å²) profiles, validating the docking poses and establishing L10 and L02 as conformationally robust mangrove-derived inhibitors of lung-cancer proteins.

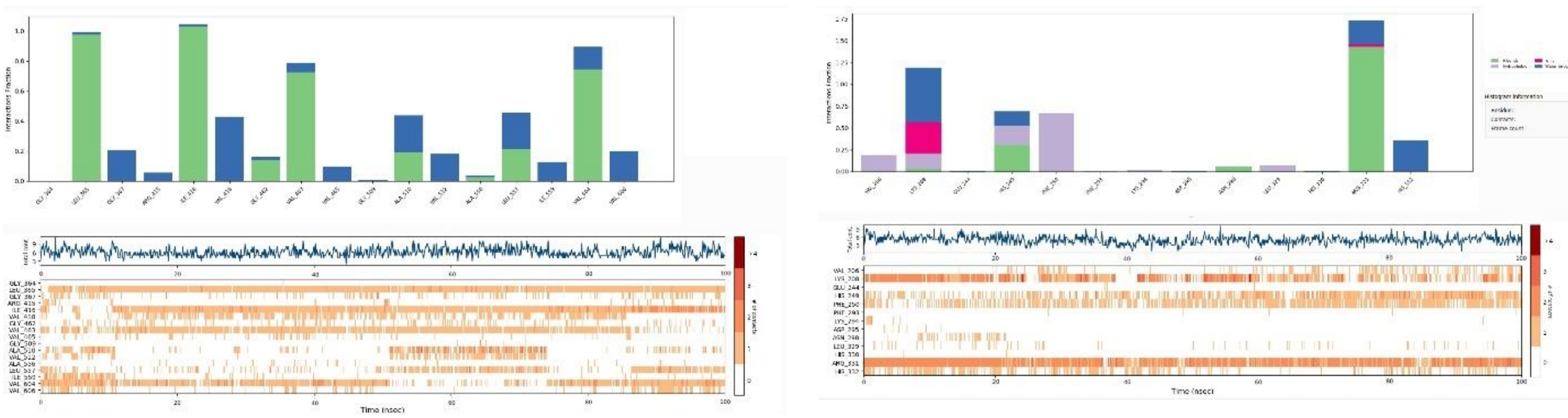

(a) (b)

Figure 11. Protein-ligand complexes (a) PDB; 1X2J -L10; (b) PDB; 5XNV -L02

Figure 11 summarizes how stable L10 and L02 remain during MD. For 1X2J–L10, RMSD stays low and contacts are frequent and long-lived, showing a stable Keap1 fold and preserved docking interactions. For 5XNV–L02, RMSD is still stable but contact fractions vary more, suggesting a flexible YEATS2 pocket and that L02 samples several related binding modes. This plasticity may help L02 adapt to different histone contexts, but longer runs or free-energy work are needed to confirm durable inhibition.

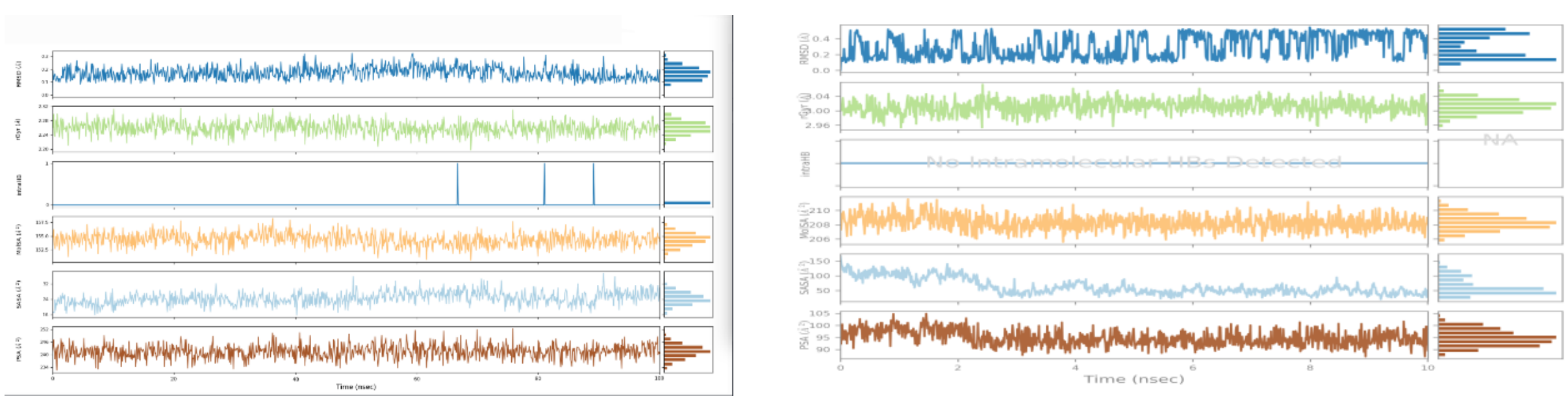

(a) (b)

Figure 12. Protein-ligand complexes (a) PDB; 1X2J -L10; (b) PDB; 5XNV -L02

Figure 12 tracks key MD descriptors for both complexes. For 1X2J–L10, RMSD, RMSF, Rg, and hydrogen bonds fluctuate within narrow ranges, supporting a compact and stable Keap1–ligand assembly over time. For 5XNV–L02, the same parameters remain globally stable but show slightly larger local fluctuations, consistent with a more flexible YEATS2 pocket that still preserves overall complex integrity.

3.4. Ramachandran plot for Docked protein complex

The Ramachandran plot is a key tool in structural biology used to visualize the torsion, or dihedral, angles of a protein backbone. It maps the phi (Φ) and psi (Ψ) angles along the x- and y-axes, respectively, each ranging from –180° to 180°. The plot shown in Figure 13 illustrates these angular distributions within the protein structure.

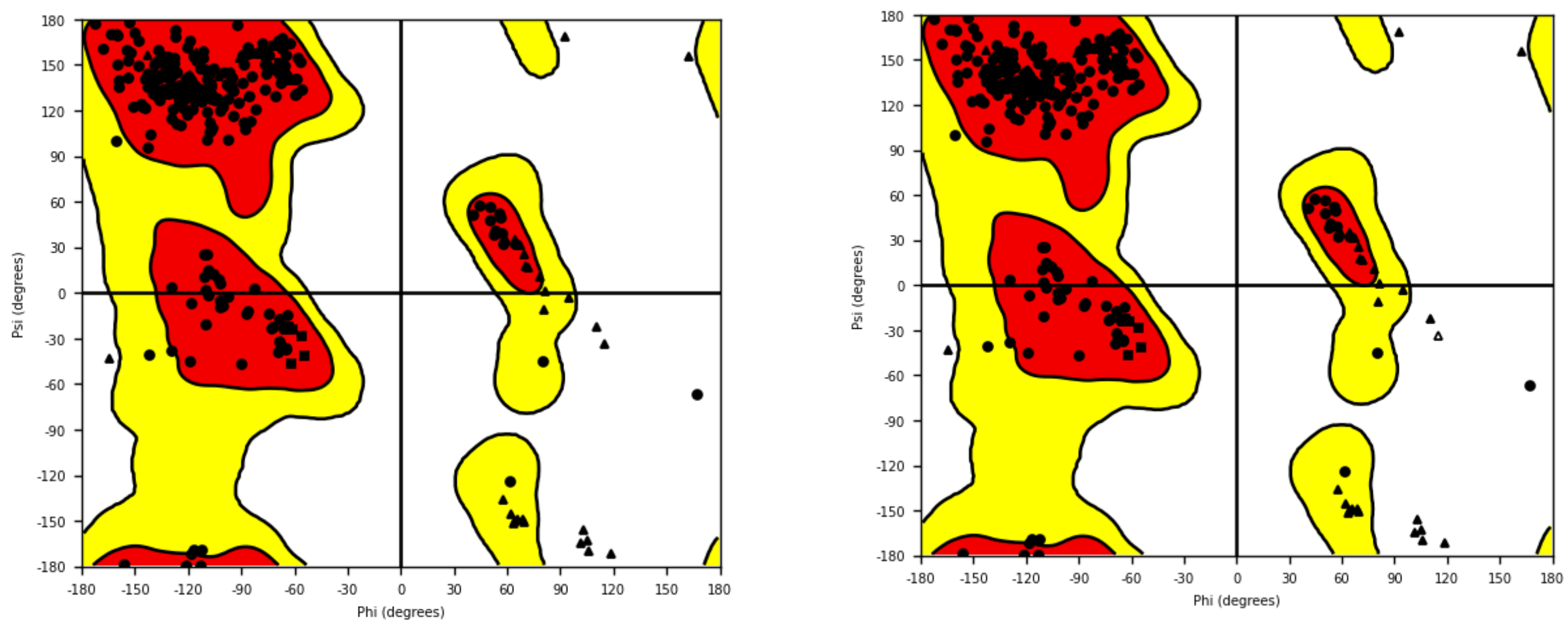

(a) **(b)**

Figure 13. Protein-ligand complexes (a) PDB: 1X2J -L10; (b) PDB: 5XNV -L02 of Ramachandran plot for Docked protein complex

Figure 13 shows that both docked complexes retain good backbone geometry. Most residues for 1X2J–L10 and 5XNV–L02 lie in favored or additionally allowed Ramachandran regions, confirming acceptable stereochemical quality of the modeled protein–ligand structures. The docked 1X2J–L10 complex shows a Ramachandran distribution very similar to that reported for wild-type Keap1 Kelch structures, where about 90–92% of residues fall in the most favored regions and only a small minority appear in additionally allowed or outlier zones. This overlap in backbone φ–ψ space indicates that ligand binding and docking did not distort the characteristic six-bladed β-propeller geometry of Keap1, and that the modeled complex preserves the stereochemical quality expected from high-resolution wild-type Keap1 crystals.

3.5 In silico studies

3.5.1 Pharmacokinetics and Drug-Likeness Study

In the pharmaceutical development, every ligand plays a distinctive role in shaping a harmonious symphony, characterized by molecular characteristics that resonate with the complexities of biological frameworks. Ranging from the lightweight L01 to the heavyweight contender L14, each ligand's molecular mass serves as a musical note in this orchestrated arrangement, with L06 emerging as a standout virtuoso due to its considerable weight.

~~***Table 6.*** **Data of Lipinski rule, pharmacokinetics, and drug-likeness.**~~

The most of these potential compounds followed Lipinski's rule of five, the property features like molecular weight, log P (octanol-water partition coefficient), number of hydrogen bond donors and acceptors are within range which usually means good oral bioavailability mentioned in Table 6. In particular, L01, L02, L05, L07, L08 and ligands did not violate this rule even once. The topological polar surface area (tPSA) values were balance for the dataset. Some of the ligands (L01, L02, L05, L06, and IPP_L10) had tPSA values in the range of 12–51 Å² while others showed moderate tPSA values (about 36 to 55 Å²), indicating that they are able to cross biological membranes assemblage [20]. On the other hand, ligands L07, L08 and L10 had higher tPSA values (∼107–148 Å²), thus losing passive diffusion potential that could be compensated by active transport mechanisms. Another parameter that was different between the compounds was molecular flexibility (number of rotatable bonds). L06 and L09 were highly flexible (14–16 rotatable bonds), whereas the majority of the other compounds possessed moderate flexibility (0–6 rotatable bonds). The predicted bioavailability scores were moderate for most of the ligands.

Table 6. **Data of Lipinski rule, pharmacokinetics, and f drug-likeness.**

Ligand No	Molecular weight	Number of Rotatable bonds	Hydrogen Bond acceptor	Topological polar surface area (Å²)	Lipinski rule		Bioavailability score
					Yes	No	
L01	164.65	0	2	12.53 Å²	Yes	0	0.55
L02	242.38	1	3	37.30 Å²	Yes	0	0.85
L03	406.55	0	7	64.61 Å²	Yes	0	0.55
L04	394.67	22	2	26.30 Å²	Yes	1	0.55

L05	257.35	3	5	36.75	Yes	0	0.55
L06	304.72	14	2	26.30	Yes	1	0.55
L07	293.42	5	7	148.04	Yes	0	0.55
L08	204.26	6	6	107.22	Yes	0	0.55
L09	315.72	16	2	37.30	Yes	1	0.85
L10	186.20	6	6	121.38	Yes	1	0.55
L11	386.65	6	1	20.23	Yes	1	0.55
L12	239.40	2	4	51.05	Yes	0	0.55
L13	290.44	8	4	36.92	Yes	0	0.55
L14	499.96	4	1	32.09	Yes	1	0.55

3.5.2 Toxicity

The tabular data presents a thorough analysis of the toxic characteristics related to various ligands, encompassing both aquatic and non-aquatic environments. Using the webtool named admetSAR (http://lmmd.ecust.edu.cn/admetsar2) pharmacokinetics and drug-likeness data were generated (64, 65).

Table 7. **Summary of the aquatic and non-aquatic toxicity properties of the studied ligands.**

Ligand	Absorption Human Intestinal	Caco-2 Permeability	Blood-Brain Barrier (BBB)	P- I glycoprotein inhibitor	Cation Transporter Renal Organic	P- II glycoprotein substrate	Sub-cellular localization	Substrate CYP450 2C9	Inhibitor CYP450 1A2
L01	0.99	0.79	0.98	0.98	0.81	0.75	0.62	0.80	0.66
L02	0.99	0.79	0.97	0.98	0.85	0.78	0.48	0.79	0.60
L03	1.00	1.00	1.00	0	0	0	0.45	0	0
L04	0.98	0.78	0.98	0.85	0.87	0.67	0.51	0.86	0.62
L05	0.96	0.60	0.98	0.80	0.60	0.50	0.38	0.87	0.88
L06	0.97	0.74	0.97	0.89	0.88	0.71	0.60	0.83	0.79
L07	0.93	0.62	0.53	0.98	0.76	0.74	0.53	0.81	0.73
L08	0.85	0.80	0.59	0.86	0.93	0.59	0.74	0.85	0.86

L09	0.99	0.99	0.95	0.94	0.92	0.59	0.54	0.76	0.91
L10	0.86	0.55	0.51	0.99	0.92	0.73	0.64	0.85	0.84
L11	1.00	0.67	0.98	0.77	0.64	0.58	0.65	0.80	0.84
L12	0.98	0.53	0.92	0.72	0.59	0.57	0.74	0.71	0.58
L13	92.51	1.952	-0.447	0.98	0.93	0	0.61	0	0
L14	1.00	0.59	0.94	0.90	0.81	0.64	0.36	0.80	0.92

In these classification models, higher probability values indicate a greater likelihood of the positive outcome (e.g., absorption, penetration, inhibition, or substrate status), with values typically interpreted as positive predictions when exceeding ~0.5–0.6, depending on the specific endpoint and model calibration. Table 7 summarizes key predicted pharmacokinetic parameters for the ligands. Human intestinal absorption (HIA) probabilities were predominantly high (0.85–1.00 for most compounds, with L01, L02, L04, L06, L09, L11, and L14 approaching or reaching 0.99–1.00), indicating excellent predicted oral bioavailability and efficient gastrointestinal uptake. This trend aligns closely with Caco-2 permeability predictions, a well-validated in vitro proxy for intestinal epithelial transport. Ligands L01, L02, L04, L06, and L09 exhibited particularly strong permeability (0.74–0.99), supporting their potential for effective passive transcellular absorption. Blood-brain barrier (BBB) penetration probabilities varied substantially across the series. Several ligands (L01, L04, L05, L06, L09, L11, and L14) displayed high scores (>0.90), suggesting facile CNS entry. Such profiles may confer advantages for therapeutic applications requiring central activity (e.g., neurological disorders) but raise concerns for peripheral-targeted agents, where unintended CNS exposure could precipitate off-target effects or toxicity. Interactions with P-glycoprotein (P-gp), a major efflux transporter influencing bioavailability and multidrug resistance, revealed widespread moderate-to-high inhibition potential (0.72–0.99 across the set). Notably, L01, L02, L03, L07, and L10 showed strong P-gp inhibition probabilities (≥0.97), which could enhance cellular retention and intracellular accumulation of co-administered drugs or the ligands themselves. However, certain compounds (e.g., L03, L07, L08) were also predicted as P-gp substrates (probabilities 0.65–0.74), potentially leading to efflux-mediated reduced exposure in target tissues. Renal organic cation transporter (OCT) substrate probabilities exhibited considerable diversity (0.36–0.94), implying differential renal clearance mechanisms. Ligands with elevated scores (e.g., L03, L04, L06 ≥0.85) may

undergo more efficient active tubular secretion, favoring rapid elimination and potentially lower systemic exposure upon repeated dosing. Subcellular localization predictions pointed to preferential mitochondrial or cytoplasmic accumulation for most ligands (probabilities 0.36–0.74), which could influence interactions with compartment-specific targets or metabolic enzymes. Cytochrome P450 (CYP) metabolism profiles highlighted variable substrate and inhibition liabilities. Several ligands (L04, L05, L06, L09) displayed substantial CYP2C9 substrate probabilities (≥0.80), consistent with expected hepatic Phase I metabolism. Inhibition of CYP1A2 was pronounced in select cases (e.g., L05: 0.88, L09: 0.91, L14: 0.92), raising the possibility of drug–drug interactions via impaired clearance of CYP1A2 substrates.

4. Conclusion

In this multi-scale computational study, phytochemicals from the mangrove species Rhizophora mucronata was investigated for their ability to modulate two important lung cancer-related proteins: the Keap1 Kelch domain (PDB 1X2J) and the YEATS2 YEATS domain (PDB 5XNV). In this study, a number of compounds with favorable electronic properties, moderate predicted binding affinities and acceptable in-silico physicochemical profiles were screened using Density Functional Theory (DFT), molecular docking (PyRx and Schrödinger Glide), 100-ns molecular dynamics simulations and ADMET profiling. Among the analyses performed, Ligand L10 (cyclohexane-1,2,3,4,5,6-hexaol) was one of the most preferred candidates. Our results propose that naturally derived compounds from Rhizophora mucronata can be a promising starting point for the design of new inhibitors of Keap1–Nrf2 and YEATS2-pathways, relevant to NSCLC. Nevertheless, the current results are purely based on calculations. Docking scores, DFT descriptors and MD stability indicators suggest potential activity in a semi-quantitative manner and should not be over-interpreted as indicative of biological activity. Experimental validation will be a critical way to build on these findings. We propose a series of initial validation steps to assess the efficacy in vitro: (1) cytotoxicity and anti-proliferative effects in NSCLC cell lines such as A549 and H1975 that represent Keap1-mutant models with selectivity assessment in normal lung epithelial cells, (2) examination of target engagement and pathway modulation utilizing Western blotting, qRT-PCR, and H3K27ac-dependent reporter assays for Nrf2 transcriptional activity; (3) indications of apoptosis, cell cycle arrest, or potential

synergy with standard chemotherapeutics; and (4) further mechanistic pharmacokinetics studies on the most active compounds.

Abbreviations

MD: Molecular dynamics; ADMET: Absorption, Distribution, Mechanism, Excretion, and Toxicity; PDB: Protein Data Bank; RMSD: Root mean square deviation; RMSF: Root mean square fluctuation.

Disclosures Peer-review: Externally peer-reviewed.

Conflict of Interest: None declared.

Ethics approval and consent to participate

Not applicable.

Consent for publication

Not applicable.

Availability of data and materials

All data underlying the findings of this study are available within the manuscript and its Supporting Information files, with additional items obtainable upon reasonable request from the corresponding author (Ajoy Kumer, kumarajoy.cu@gmail.com). Specifically, the Supporting Information provides the optimized ligand structures (from DFT), the protein structures (PDB 1X2J and 5XNV), docking grid parameters (Table 2), top docking poses, and supplementary tables containing the SwissADME and pkCSM prediction outputs. Molecular dynamics simulation input parameters and key trajectory analysis files (RMSD, RMSF, protein–ligand contacts) are available from the corresponding author. No custom code was developed; all analyses used commercial or publicly available software (Materials Studio, PyRx/AutoDock Vina, Schrödinger Suite, Desmond), thus meeting PLOS ONE's minimal data set requirements for computational studies.

Competing interests

The authors declare that they have no competing interests.

Funding

Not applicable.

Authors' contributions

Z.A.A.M., B.S.A.A: Performed the conceptual study, and data analysis. M.N., M.M.H.: did the simulation, data analysis, and draft writing. A.K.: performed the conceptual study, supervision and administrative managing; F. T.A.A., H.I.M.A.D.: revised the draft writing and funding acquisition; F.R., M.F.A.H.K.: did the draft writing and data analysis; NS.K. AK, and S.A.M.A-Y revised and review the manuscript.

Acknowledgements

The authors express their gratitude to Computationally Design of Drug & Material Lab (CDDM Lab) for providing technical support that contributed to the progress of this research.

Supplementary Information

Not applicable but attached supplementary file.

References

1. Bray F, Laversanne M, Sung H, Ferlay J, Siegel RL, Soerjomataram I, et al. Global cancer statistics 2022: GLOBOCAN estimates of incidence and mortality worldwide for 36 cancers in 185 countries. CA: a cancer journal for clinicians. 2024;74(3):229-63.
2. Li C, Lei S, Ding L, Xu Y, Wu X, Wang H, et al. Global burden and trends of lung cancer incidence and mortality. 2023;136(13):1583-90.
3. Lung cancer statistics [database on the Internet]2022.
4. Society AC. Cancer Facts & Figures 20252025.
5. Malhotra J, Malvezzi M, Negri E, La Vecchia C, Boffetta PJERJ. Risk factors for lung cancer worldwide. 2016;48(3):889-902.
6. Leiter A, Veluswamy RR, Wisnivesky JPJNrCo. The global burden of lung cancer: current status and future trends. 2023;20(9):624-39.
7. Islami F, Torre LA, Jemal AJTlcr. Global trends of lung cancer mortality and smoking prevalence. 2015;4(4):327.
8. Samet JMJTsc. Tobacco smoking: the leading cause of preventable disease worldwide. 2013;23(2):103-12.
9. Corrales L, Rosell R, Cardona AF, Martin C, Zatarain-Barrón ZL, Arrieta OJCrioh. Lung cancer in never smokers: The role of different risk factors other than tobacco smoking. 2020;148:102895.

10. Kusel J, Timm B, Lockhart IJTid. The impact of smoking in the home on the health outcomes of non-smoker occupants in the UK. 2013;11(1):3.
11. Eckel SP, Cockburn M, Shu Y-H, Deng H, Lurmann FW, Liu L, et al. Air pollution affects lung cancer survival. 2016;71(10):891-8.
12. Berg CD, Schiller JH, Boffetta P, Cai J, Connolly C, Kerpel-Fronius A, et al. Air pollution and lung cancer: a review by international association for the study of lung cancer early detection and screening committee. 2023;18(10):1277-89.
13. Lorenzo-Gonzalez M, Torres-Duran M, Barbosa-Lorenzo R, Provencio-Pulla M, Barros-Dios JM, Ruano-Ravina AJErorm. Radon exposure: a major cause of lung cancer. 2019;13(9):839-50.
14. Deshmukh G, Sawarkar H, Biyani KJE. Review on lung cancer with emphasis on current treatment. 2024;4(4).
15. Petrova D, Špacírová Z, Fernandez-Martinez NF, Ching-Lopez A, Garrido D, Rodriguez-Barranco M, et al. The patient, diagnostic, and treatment intervals in adult patients with cancer from high-and lower-income countries: A systematic review and meta-analysis. 2022;19(10):e1004110.
16. Ebell MH, Thai TN, Royalty KJJPhr. Cancer screening recommendations: an international comparison of high income countries. 2018;39(1):7.
17. Shankar A, Saini D, Dubey A, Roy S, Bharati SJ, Singh N, et al. Feasibility of lung cancer screening in developing countries: challenges, opportunities and way forward. 2019;8(Suppl 1):S106.
18. Lubuzo B, Ginindza T, Hlongwana KJTPAMJ. The barriers to initiating lung cancer care in low-and middle-income countries. 2020;35:38.
19. Newman DJ, Cragg GM. Natural products as sources of new drugs over the nearly four decades from 01/1981 to 09/2019. Journal of natural products. 2020;83(3):770-803.
20. Youssef AM, Maaty DA, Al-Saraireh YM. Phytochemistry and anticancer effects of mangrove (Rhizophora mucronata Lam.) leaves and stems extract against different cancer cell lines. Pharmaceuticals. 2022;16(1):4.
21. Duma N, Santana-Davila R, Molina JR, editors. Non–small cell lung cancer: epidemiology, screening, diagnosis, and treatment. Mayo Clinic Proceedings; 2019: Elsevier.

22. Sung H, Ferlay J, Siegel RL, Laversanne M, Soerjomataram I, Jemal A, et al. Global cancer statistics 2020: GLOBOCAN estimates of incidence and mortality worldwide for 36 cancers in 185 countries. CA: a cancer journal for clinicians. 2021;71(3):209-49.
23. Yang S-R, Schultheis AM, Yu H, Mandelker D, Ladanyi M, Buettner R, editors. Precision medicine in non-small cell lung cancer: Current applications and future directions. Seminars in cancer biology; 2022: Elsevier.
24. Khatun MCS, Muhit MA, Maruf MMH, Iqbal MA, Rahman SA. Pharmacological evaluation of mangrove plant Rhizophora mucronata (Lam.) grown in the coastal area of Sundarbans. PLoS One. 2026;21(1):e0340646.
25. Nabeelah Bibi S, Fawzi MM, Gokhan Z, Rajesh J, Nadeem N, RR RK, et al. Ethnopharmacology, phytochemistry, and global distribution of mangroves―A comprehensive review. Marine drugs. 2019;17(4):231.
26. Chakraborty K. Chemistry and biological activities of rhizophora mucronata lam. Bioactive Compounds in Mangroves and their Associates: Springer; 2025. p. 1-26.
27. Sadeer NB, Rocchetti G, Senizza B, Montesano D, Zengin G, Uysal A, et al. Untargeted metabolomic profiling, multivariate analysis and biological evaluation of the true mangrove (Rhizophora mucronata Lam.). 2019;8(10):489.
28. Suganthy N, Pandima Devi KJPb. In vitro antioxidant and anti-cholinesterase activities of Rhizophora mucronata. 2016;54(1):118-29.
29. Li X, Zhang D, Hannink M, Beamer LJJJoBC. Crystal structure of the Kelch domain of human Keap1. 2004;279(52):54750-8.
30. Padmanabhan B, Tong KI, Ohta T, Nakamura Y, Scharlock M, Ohtsuji M, et al. Structural basis for defects of Keap1 activity provoked by its point mutations in lung cancer. 2006;21(5):689-700.
31. Padmanabhan B, Tong KI, Ohta T, Nakamura Y, Scharlock M, Ohtsuji M, et al. Structural basis for defects of Keap1 activity provoked by its point mutations in lung cancer. Molecular cell. 2006;21(5):689-700.
32. Mi W, Guan H, Lyu J, Zhao D, Xi Y, Jiang S, et al. YEATS2 links histone acetylation to tumorigenesis of non-small cell lung cancer. 2017;8(1):1088.
33. Mi W, Guan H, Lyu J, Zhao D, Xi Y, Jiang S, et al. YEATS2 links histone acetylation to tumorigenesis of non-small cell lung cancer. Nature communications. 2017;8(1):1088.

34. Kohn W, Becke AD, Parr RG. Density functional theory of electronic structure. The journal of physical chemistry. 1996;100(31):12974-80.
35. Parr R, Yang W. Horizons of quantum chemistry. Horizons of Quantum Chemistry Springer https://doi org/101007/978-94-009-9027-2. 1980.
36. Parr RG, Yang W. Density functional approach to the frontier-electron theory of chemical reactivity. Journal of the American Chemical Society. 1984;106(14):4049-50.
37. Spassov V, Yan L. Accelrys Software Inc., discovery studio modeling environment, release 4.0. Proteins Struct Funct Bioinforma. 2013;81:704-14.
38. Sandor M, Kiss R, Keserű GrM. Virtual fragment docking by Glide: a validation study on 190 protein− fragment complexes. Journal of Chemical Information and Modeling. 2010;50(6):1165-72.
39. Bowers KJ, Chow E, Xu H, Dror RO, Eastwood MP, Gregersen BA, et al., editors. Scalable algorithms for molecular dynamics simulations on commodity clusters. Proceedings of the 2006 ACM/IEEE Conference on Supercomputing; 2006.
40. Daina A, Michielin O, Zoete V. SwissADME: a free web tool to evaluate pharmacokinetics, drug-likeness and medicinal chemistry friendliness of small molecules. Scientific reports. 2017;7(1):42717.
41. Tsaioun K, Blaauboer BJ, Hartung T. Evidence-based absorption, distribution, metabolism, excretion (ADME) and its interplay with alternative toxicity methods. ALTEX-Alternatives to animal experimentation. 2016;33(4):343-58.
42. Chandrasekaran B, Abed SN, Al-Attraqchi O, Kuche K, Tekade RK. Computer-aided prediction of pharmacokinetic (ADMET) properties. Dosage form design parameters: Elsevier; 2018. p. 731-55.
43. Pires DE, Blundell TL, Ascher DB. pkCSM: predicting small-molecule pharmacokinetic and toxicity properties using graph-based signatures. Journal of medicinal chemistry. 2015;58(9):4066-72.
44. López J, Anitescu C, Valizadeh N, Rabczuk T, Alajlan N. Structural shape optimization using Bézier triangles and a CAD-compatible boundary representation. Engineering with Computers. 2020;36:1657-72.

45. Kumer A, Ahmed B, Sharif M, Al-Mamun A. A theoretical study of aniline and nitrobenzene by computational overview. Asian Journal of Physical and Chemical Sciences. 2017;4(2):1-12.

46. Kumer A, Paul S, Sarker MN, Islam MJ. The prediction of thermo physical, vibrational spectroscopy, chemical reactivity, biological properties of morpholinium borate, phosphate, chloride and bromide ionic liquid: a DFT study. International Journal of New Chemistry. 2019;6(4):236-53.

47. KUMER A, Sarker MN, Sunanda P. The theoretical investigation of HOMO, LUMO, thermophysical properties and QSAR study of some aromatic carboxylic acids using HyperChem programming. International Journal of Chemistry and Technology. 2019;3(1):26-37.

48. Kumer A, Sarker N, Paul S, Zannat A. The theoretical prediction of thermophysical properties, HOMO, LUMO, QSAR and biological indics of cannabinoids (CBD) and tetrahhdrocannabinol (THC) by computational chemistry. Advanced Journal of Chemistry-Section A. 2019;2(3):190-202.

49. Hoque MM, Kumer A, Hussen MS, Khan MW. Theoretical Evaluation of 5, 6-Diaroylisoindoline-1, 3-dione as Potential Carcinogenic Kinase PAK1 Inhibitor: DFT Calculation, Molecular Docking Study and ADMET Prediction. International Journal of Advanced Biological and Biomedical Research. 2021;9(1):77-104.

50. Islam MJ, Sarker MN, Kumer A, Paul S. The Evaluation and Comparison of Thermo-Physical, Chemical and Biological Properties of Palladium (II) Complexes on Binuclear Amine Ligands with Different Anions by DFT Study. International journal of Advanced Biological and Biomedical Research. 2019;7(4):315-34.

51. Kumer A, Chakma U, Chandro A, Howlader D, Akash S, Kobir ME, et al. MODIFIED D-GLUCOFURANOSE COMPUTATIONALLY SCREENING FOR INHIBITOR OF BREAST CANCER AND TRIPLE BREAST CANCER: CHEMICAL DESCRIPTOR, MOLECULAR DOCKING, MOLECULAR DYNAMICS AND QSAR. Journal of the Chilean Chemical Society. 2022;67(3):5623-35.

52. Kumer A, Chakma U, Rana MM, Chandro A, Akash S, Elseehy MM, et al. Investigation of the New Inhibitors by Sulfadiazine and Modified Derivatives of α-D-glucopyranoside for White Spot Syndrome Virus Disease of Shrimp by In Silico: Quantum Calculations,

Molecular Docking, ADMET and Molecular Dynamics Study. Molecules. 2022;27(12):3694.
53. Kumer A, Jahidul M, Paul S. Effect of external electric field and temperature on entropy, heat of capacity, and chemical reactivity with QSAR study of morphonium chloride and nitrous ionic liquids crystal using DFT. Chemical Methodologies. 2020;4:595-604.
54. Cheng K, Zheng Q-Z, Qian Y, Shi L, Zhao J, Zhu H-L. Synthesis, antibacterial activities and molecular docking studies of peptide and Schiff bases as targeted antibiotics. Bioorganic & medicinal chemistry. 2009;17(23):7861-71.
55. Hermann T, Westhof E. Docking of cationic antibiotics to negatively charged pockets in RNA folds. Journal of medicinal chemistry. 1999;42(7):1250-61.
56. Yang Y, Yao K, Repasky MP, Leswing K, Abel R, Shoichet BK, et al. Efficient exploration of chemical space with docking and deep learning. Journal of Chemical Theory and Computation. 2021;17(11):7106-19.
57. Friesner RA, Murphy RB, Repasky MP, Frye LL, Greenwood JR, Halgren TA, et al. Extra precision glide: Docking and scoring incorporating a model of hydrophobic enclosure for protein− ligand complexes. Journal of medicinal chemistry. 2006;49(21):6177-96.
58. Friesner RA, Banks JL, Murphy RB, Halgren TA, Klicic JJ, Mainz DT, et al. Glide: a new approach for rapid, accurate docking and scoring. 1. Method and assessment of docking accuracy. Journal of medicinal chemistry. 2004;47(7):1739-49.
59. Alzain AA, Mukhtar RM, Abdelmoniem N, Shoaib TH, Osman W, Alsulaimany M, et al. Modulation of NRF2/KEAP1-mediated oxidative stress for cancer treatment by natural products using pharmacophore-based screening, molecular docking, and molecular dynamics studies. Molecules. 2023;28(16):6003.
60. Brogi S, Guarino I, Flori L, Sirous H, Calderone V. In silico identification of natural products and world-approved drugs targeting the KEAP1/NRF2 pathway endowed with potential antioxidant profile. Computation. 2023;11(12):255.
61. Boyenle ID, Divine UC, Adeyemi R, Ayinde KS, Olaoba OT, Apu C, et al. Direct Keap1-kelch inhibitors as potential drug candidates for oxidative stress-orchestrated diseases: A review on In silico perspective. Pharmacological Research. 2021;167:105577.

62. Li X, Li X-M, Jiang Y, Liu Z, Cui Y, Fung KY, et al. Structure-guided development of YEATS domain inhibitors by targeting π-π-π stacking. Nature chemical biology. 2018;14(12):1140-9.

63. Wang X, Cheng G, Zhao J, Gao P, Mao H, Yuan C, et al. Discovering potential inhibitors of the YEATS domain of YEATS2 through virtual screening, molecular optimization and molecular dynamics simulations. New Journal of Chemistry. 2023;47(42):19447-60.

64. Yang H, Lou C, Sun L, Li J, Cai Y, Wang Z, et al. admetSAR 2.0: web-service for prediction and optimization of chemical ADMET properties. Bioinformatics. 2019;35(6):1067-9.

65. Cheng F, Li W, Zhou Y, Shen J, Wu Z, Liu G, et al. admetSAR: a comprehensive source and free tool for assessment of chemical ADMET properties. ACS Publications; 2012.

Click here to access/download
Supporting Information
Table and Supplementary.docx

Investigating the anti-cancer properties of phytochemicals from mangrove tree, *Rizhophora muarnata*, as Lung Cancer protein Inhibitors: Insights from Multi-scale Computational and In silico Analysis

Zena Abdul-Ameer Mahdi[1], Batool Shakir Abed Almjalaw[2], Mollah Naimuzzaman[3], Md Mahabub Hasan[4], Ajoy Kumer*[5,6], Farah Tareq Al-Alaq[7], Hussein O.M. Al-Dahmoshi[8], Faizur Rahman[4], Md. Fayez Al-Hakim Khan[4], Noor S.K. Al-Khafaji[9], and Sarah Alwan Malik Al-Yasiri[10]

[1]Department of medical physics, college of Applied Medical Sciences, University of Kerbala, Karbala 56001, Iraq., email: Zena.a.mahdy@uokerbala.edu.iq

[2] Ministry of Education, General Directorate of Education in Kerbala. Kerbala, Iraq Email: batool_shakir@karbala.edu.iq

[3]Department of Agroforestry and Environmental Science, Sylhet Agricultural University, Sylhet-3100, Bangladesh; Email: mollahnaimuzzaman@gmail.com

[4]College of Agricultural Sciences, IUBAT—International University of Business Agriculture and Technology, 4 Embankment Drive Road, Sector 10 Uttara Model Town, Dhaka 1230, Bangladesh. Email: mahabubhasan33445@gmail.com; FR: faizurrahmanzidni80@gmail.com FHK: fayezalhakim117@gmail.com

[5]Department of General Medicine, Saveetha Institute of Medical and Technical Sciences in Saveetha Medical College and Hospital, Chennai, India, Email: kumarajoy.cu@gmail.com

[6]College of Arts and Sciences, Department of Chemistry, IUBAT—International University of Business Agriculture and Technology, 4-Embankment Drive Road, Sector 10 Uttara Model Town, Dhaka 1230, Bangladesh. Email: kumarajoy.cu@gmail.com

[7]Department of Biology, college of science, University of Babylon, Hilla 51001, Babylon, Iraq, Email: sci.farah.tariq@uobabylon.edu.iq

[8]Department of Biology, college of science, University of Babylon, Hilla 51001, Babylon, Iraq, Email: sci.hussein.oleiwi@uobabylon.edu.iq

[9]Department of Biology, college of science, University of Babylon, Hilla 51001, Babylon, Iraq, Email: sci.noor.salman@uobabylon.edu.iq

[10]Department of environmental health, College of Applied Medical Sciences, University of Kerbala, Karbala 56001, Iraq., email: alyasirsara@gmail.com

Corresponding Authors

Email: kumarajoy.cu@gmail.com

Abstract:

Graphically presentation of abstract:

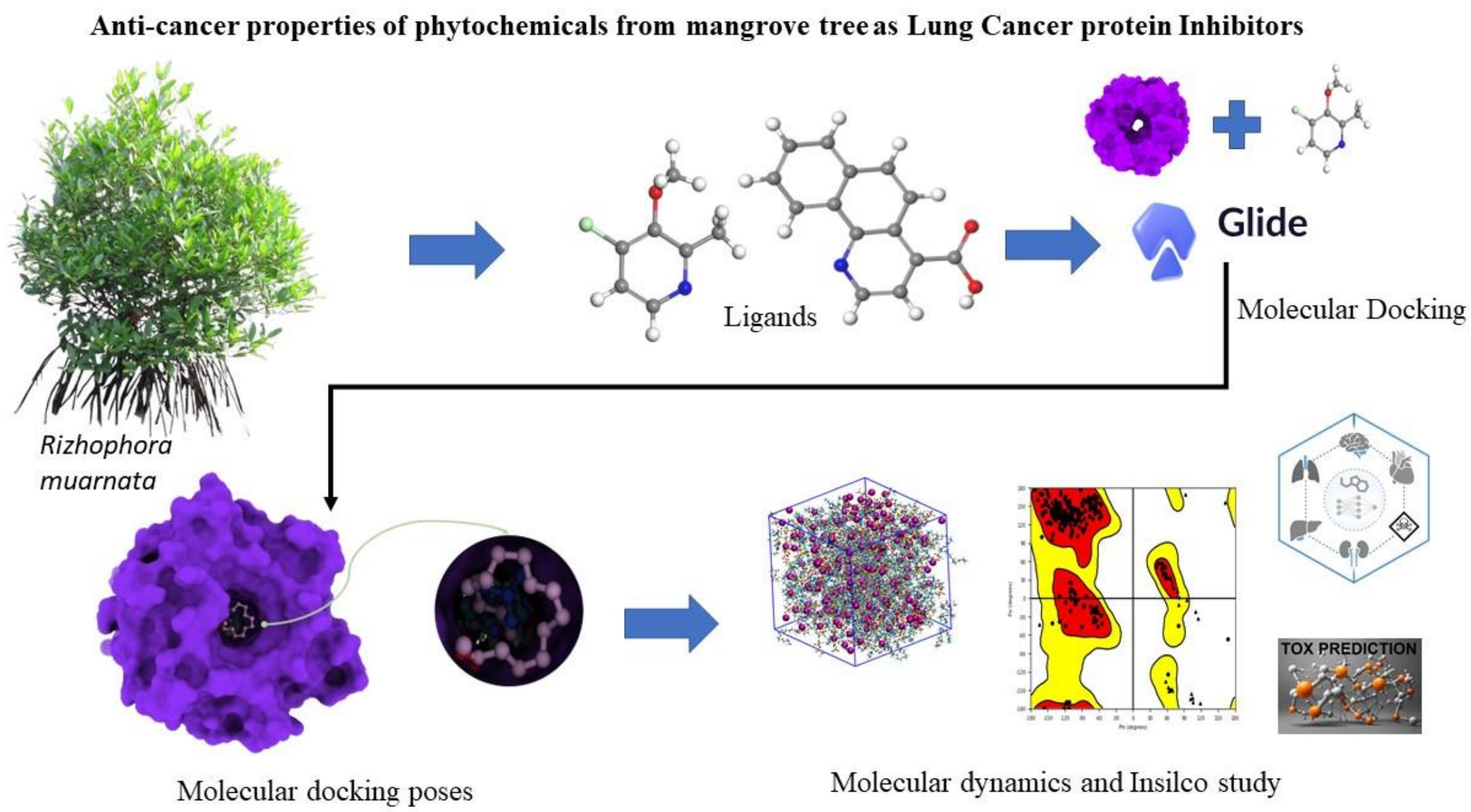

Lung cancer remains the leading cause of cancer-related mortality worldwide, with a five-year survival rate of approximately 20–25% despite advances in targeted therapy and immunotherapy. There is a continuous need for novel, low-toxicity compounds capable of modulating key oncogenic pathways in non-small cell lung cancer (NSCLC). Mangrove ecosystems are rich reservoirs of bioactive phytochemicals, yet the inhibitory potential of compounds from *Rhizophora mucronata* against validated lung cancer-associated proteins has not been systematically explored using multi-scale computational approaches. This study employed an integrated computational pipeline to evaluate 14 phytochemicals from Rhizophora mucronata. Density Functional Theory (DFT) calculations at the B3LYP/6-31G* level were performed to characterize electronic properties of the ligands. Molecular docking studies were conducted using both AutoDock Vina (PyRx) and Schrödinger Glide against the Keap1 Kelch domain (PDB 1X2J) and the YEATS2 YEATS domain (PDB 5XNV). Top-ranked complexes were subjected to 100-ns molecular dynamics (MD) simulations in Desmond, and pharmacokinetic

and toxicity profiles were predicted using SwissADME and pkCSM. Among the screened compounds, ligand L10 (cyclohexane-1,2,3,4,5,6-hexaol) exhibited the smallest HOMO–LUMO energy gap (0.36 eV), favorable binding affinities (Glide score of –7.789 kcal/mol against 1X2J), multiple hydrogen-bonding interactions, and stable complex behavior during MD simulations (Cα RMSD < 3 Å). Several other ligands (L02, L05, and L07) also displayed promising profiles. Most compounds complied with Lipinski's rule of five and showed acceptable predicted ADMET properties. These preliminary findings suggest that *Rhizophora mucronate* -derived phytochemicals, particularly L10, are promising candidates for further experimental evaluation as modulators of Keap1 and YEATS2 in lung cancer. This study provides a computational framework for prioritizing natural product leads from mangrove species for subsequent in vitro and in vivo validation.

Keywords: *Rhizophora mucronata*, DFT, molecular docking, molecular dynamics, ADMET, Keap1, YEATS2, NSCLC, natural products, in silico screening

1. Introduction

Lung cancer is the top cause of cancer deaths worldwide, killing around 1.8 million people each year and contributing nearly one-fifth of all cancer-related fatalities(1). It ranks as the most frequently diagnosed cancer in men and the second most common in women, with an estimated 2.2 million new cases and 1.8 million deaths reported globally in 2020, representing about 18% of total cancer mortality(2). In 2022, China, the US and Japan had the highest number of deaths from lung cancer, which Age-standardised rate (ASR) lets us compare cancer risk between countries by removing age effects. A country with lower ASR can still have more cases if its population is larger (3). In 2022–2024 data, lung cancer remains the top cause of cancer death globally, with about 2.5 million new cases and 1.8 million deaths each year (3). Despite substantial progress in targeted therapies and immunotherapy, the 5-year relative survival rate hovers around 25% for all stages combined, underscoring an urgent need for novel, low-toxicity therapeutic agents that can effectively disrupt oncogenic pathways while minimizing systemic adverse effects (4). Many powerful and often preventable factors drive lung cancer risk today (5,

6). Tobacco smoking is still the main cause, and in some countries around 80% of lung cancer deaths are directly due to smoking (7, 8). Second-hand smoke also harms non-smokers, especially in homes and workplaces (9). Outdoor air pollution, especially fine particles (PM2.5), now raises lung cancer risk even in people who have never smoked (10). Indoor air pollution from solid fuels such as coal or biomass and from cooking fumes is a major risk in many low- and middle-income countries (11, 12). Other important factors include radon, asbestos, certain workplace chemicals, and family history (12, 13).

Lung cancer often grows silently for years and is usually found late, when cure is hard. It can block airways, destroy lung tissue, spread to organs like brain, liver, and bone, and cause organ failure (14). Even with treatment, only about 1 in 4–5 patients in high-income countries live five years after diagnosis (15, 16). Approved lung cancer treatment includes surgery, radiation, chemotherapy, targeted drugs, and immunotherapy. These help many patients but still face big limits: most cases are found late, side effects can be severe, tumors often become resistant or come back, and modern targeted or immune drugs are very costly and only work for patients whose tumors have certain mutations or markers (17, 18) . Because current lung cancer treatments still have serious limits, researchers keep searching for new and better therapies, and push scientists to develop more effective, safer, and more affordable options for patients.

Natural products derived phytochemicals, low cost and affordable medicine, from terrestrial and marine ecosystems have historically supplied a substantial proportion of approved anticancer drugs, offering structurally diverse scaffolds with favorable safety profiles compared with synthetic chemotherapeutics (19). Among these, mangrove ecosystems represent underexplored reservoirs of bioactive secondary metabolites, including flavonoids, tannins, and triterpenoids, many of which exhibit potent cytotoxic, antioxidant, and anti-inflammatory activities (20). Lung cancer is broadly classified into small-cell lung cancer (SCLC, ~15%) and non-small-cell lung cancer (NSCLC, ~85%), the latter being the predominant histological subtype (21). NSCLC is characterized by late-stage diagnosis, aggressive metastatic potential, and frequent dysregulation of oxidative stress and epigenetic regulatory pathways, resulting in poor prognosis even with current standard-of-care treatments (22). Existing therapies are frequently limited by acquired resistance, severe off-target toxicities, and modest improvements in overall survival, particularly in advanced or recurrent disease (23). Consequently, there is a pressing requirement for

innovative inhibitors capable of selectively targeting key proteins driving lung cancer progression while satisfying drug-like physicochemical and pharmacokinetic criteria. Although mangrove-derived extracts have demonstrated anticancer activity against several cell lines, including lung carcinoma models (20, 24), no systematic multi-scale investigation has yet explored the potential of protein or pathway-specific inhibitors of phytochemical from *Rhizophora mucronata* against validated lung cancer protein targets.

This study recovers a critical knowledge gap, initially screening the phytochemical from *Rhizophora mucronata* against validated lung cancer by various computational approaches (density functional theory, molecular docking, and molecular dynamics) that can rapidly identify lead candidates with high binding affinity, favorable ADMET properties and structural stability prior to resource-intensive experimental validation. *Rhizophora mucronata*, a widely distributed mangrove species in tropical and subtropical intertidal zones, has been traditionally utilized in folk medicine for its anti-inflammatory, antimicrobial and antioxidant properties (25). In addition, its rich phytochemical profile particularly polyphenols, and alkaloids confers exceptional environmental resilience and translates into broad pharmacological potential, making it an ideal candidate for targeted anticancer drug discovery (26, 27). Multiple independent studies have confirmed the cytotoxic efficacy of *R. mucronata* extracts against lung cancer cell lines (28), with methanol stem extracts outperforming leaf extracts in reducing viability (20). Isolated compounds such as N-trans-para-caffeoyl-tyramine and rutin further exhibit significant growth-inhibitory effects in both in vitro and in vivo models (24, 28).

Two well-characterized lung cancer-associated proteins were selected as targets in this study: the Kelch domain of Keap1 (PDB ID: 1X2J) and the YEATS domain of YEATS2 (PDB ID: 5XNV). These proteins were chosen based on their well-documented and critical roles in non-small cell lung cancer (NSCLC) pathogenesis. Keap1 acts as a negative regulator of the transcription factor Nrf2. Mutations or dysregulation of the Keap1–Nrf2 pathway are frequently observed in NSCLC, leading to constitutive Nrf2 activation, enhanced antioxidant responses, metabolic reprogramming, and chemoresistance(29-31). Similarly, YEATS2 functions as an epigenetic reader of histone H3K27 acetylation (H3K27ac). It is often amplified or overexpressed in NSCLC, where it promotes oncogenic transcriptional programs that support tumor cell proliferation, survival, and metastasis(32, 33). Both structures were retrieved from the Protein

Data Bank after careful evaluation of resolution, R-free values, Ramachandran plot quality, and biological relevance to lung carcinogenesis. In the broader oncogenic context, Keap1 mutations disrupt ubiquitin-mediated degradation of Nrf2, thereby promoting cellular survival under oxidative stress — a hallmark of lung tumorigenesis (31). Concurrently, YEATS2-mediated epigenetic reprogramming sustains proliferation and metastasis through hyperacetylation-dependent gene activation (33). Targeting these proteins therefore represents a mechanistically rational strategy to restore redox homeostasis and suppress aberrant epigenetic transcriptional activity in NSCLC. The primary objective of this investigation was to computationally screen and characterize bioactive phytochemicals from Rhizophora mucronata as potential inhibitors of these two-lung cancer-associated proteins (1X2J and 5XNV). This was achieved by integrating Density Functional Theory (DFT), molecular docking (PyRx and Schrödinger Glide), ADMET profiling, and 100-ns molecular dynamics simulations to identify promising lead compounds with favorable electronic properties, binding affinity, pharmacokinetic profiles, and complex stability.

2. Methodology

2.1 Optimization and Ligand Preparation

The 14 phytochemicals from *Rhizophora mucronata* were systematically selected based on a comprehensive literature review of its reported secondary metabolites, prioritizing chemical diversity across multiple classes including nitrogen-containing heteroaromatics (L01, L02, L05, L014), polyethers/siloxanes (L03, L13), long-chain esters/fatty acids (L04, L06, L09), polyhydroxy carbohydrates/sugar derivatives (L07, L08, L10), steroidal alcohols (L011), and aromatic oximes/naphthalene derivatives (L012) to (L014) cover a broad range of polarity, aromaticity, hydrogen-bonding capacity, and lipophilicity that could enable diverse interactions with target proteins. The Universal Force Field (UFF) was used for geometry pre-optimization to fix bond lengths and angles that were too long or too short, get rid of steric clashes, and get reasonable starting conformations. The Build Parameters module of Materials Studio was then used to make parameter files that included all the necessary force-field terms, atom types, and partial charges for the next quantum-mechanical calculations.

Used the DMol³ module in Materials Studio to do DFT calculations with the B3LYP functional and the 6–31G* basis set. Used the COSMO model to include solvent effects when they were needed, and made convergence thresholds stricter when they were needed to make sure the energies and geometries were reliable. Input files mixed the UFF-optimized structures with the chosen DFT level and set of parameters. Each job was run until both energy and nuclear coordinates reached full convergence.

The optimized structures were first checked for thermodynamic and structural stability by examining their geometries, bond parameters, and vibrational frequencies. From these stable structures, frontier molecular orbital properties and global reactivity descriptors were obtained, including HOMO and LUMO energies, HOMO–LUMO gap (E_gap), ionization potential (I), electron affinity (A), chemical potential (μ), electronegativity (χ), hardness (η), softness (s), and electrophilicity index (ω) (34-36), which are shown in equations 1–8.

$$E_{gap} = (E_{LUMO} - E_{HOMO}) \quad (1)$$

$$I = -E_{HOMO} \quad (2)$$

$$A = -E_{LUMO} \quad (3)$$

$$(X) = \frac{I+A}{2} \quad (4)$$

$$(\omega) = \frac{\mu^2}{2\eta} \quad (5)$$

$$(\mu) = -\frac{I+A}{2} \quad (6)$$

$$(\eta) = \frac{I-A}{2} \quad (7)$$

$$(S) = \frac{1}{\eta} \quad (8)$$

2.2 Protein Preparation and Collection

Used the Keap1 Kelch domain (PDB 1X2J) (30) and the YEATS2 YEATS domain (PDB 5XNV) (32) as docking targets for lung cancer. Keap1 regulates Nrf2 turnover, and its dysregulation in NSCLC enhances chemoresistance through sustained antioxidant signaling. YEATS2 is an H3K27ac reader that is often overexpressed in NSCLC. It drives oncogenic transcription that is needed for tumors to grow. Table 1 shows high-quality crystal structures (1.60 Å for 1X2J, 2.70 Å for 5XNV, and more than 96% of residues in favored Ramachandran

regions) that were downloaded from the Protein Data Bank and prepared in Discovery Studio by removing ligands, water, and ions, and then minimizing energy to create optimized apo-protein models for docking.

***Table 1*. Protein information.**

Title	PDB ID: 1X2J	PDB ID: 5XNV
Organism	*Homo sapiens*	*Homo sapiens*
Resolution	1.60 Å	2.70 Å
R-Value Free	0.200	0.266
Ramachandran plot, %	96.18%	96.92%
References	(30)	(32)

2.3 Molecular Docking and Visualization of Docking

2.3.1 Molecular Docking by PyRx

Molecular docking educations were done with PyRx 0.8 employing the AutoDock Vina engine through its AutoDock Wizard module to predict binding affinities and preferred poses of the 14 ligands within the active sites of the two target proteins. The ready receptor and ligand files were imported distinctly. For each protein, a rectangular search grid was Shown according to the dimensions and center coordinates specified in Table 2, confirm complete coverage of the biologically related binding pocket while reducing computational cost.

Ligands were enhanced preceding to docking, and the search space was comprehensively sampled using the default Vina parameters (exhaustiveness = 8, num_modes = 9). The docking runs created ranked poses based on predicted binding free energy (kcal/mol). All resulting protein–ligand complexes were transferred and imported into Discovery Studio 2020 for detailed visualization and analysis of non-covalent interactions, including hydrogen bonds, hydrophobic contacts, π–π stacking, and van der Waals interactions. This methodical approach enabled reliable identification of the most satisfactory binding modes and quantitative comparison of ligand affinities for both 1X2J and 5XNV (37).

2.3.2 Molecular Docking by Glide from Schrodinger suite

Molecular docking was did using the Glide module (Schrödinger Release 2024-1) to deliver an orthogonal, high-precision valuation of ligand binding to the two lung cancer target proteins (PDB IDs: 1X2J and 5XNV). Ligands were first ready with LigPrep to generate low-energy 3D conformers, correct ionization states at physiological pH, and enumerate tautomers. Receptor grids were made around the experimentally determined binding sites using the Receptor Grid Generation tool, with grid center and dimensions defined in Table 2 (38), to encompass the entire biologically relevant pocket while maintaining computational efficiency.

Docking was executed in Standard Precision (SP) mode with default parameters, enabling exhaustive conformational sampling of each ligand within the receptor grid. The resulting poses were ranked by GlideScore (GScore), which accounts for van der Waals, Coulomb, lipophilic, hydrogen-bonding, metal-binding, and desolvation terms, as well as penalties for buried polar groups and rotatable-bond freezing equation [9].

$$G\ Score = a \times vdW + b \times coul + Lipo + HBond + Metal + BuryP + RotB + Site \quad (9)$$

Here, vdW is the van der Waals term, Coul is the Coulombic (electrostatic) term, Lipo represents lipophilic contacts, HBond corresponds to hydrogen-bonding interactions, Metal captures metal-binding contributions, BuryP is the penalty for buried polar groups, RotB is the penalty for freezing rotatable bonds, and Site represents polar interactions within the active site; the coefficients for vdW and Coul are a = 0.065 and b = 0.130, respectively.

***Table 2.* Grid box parameters used for docking analysis in this study.**

***Table 2.* Grid box parameters used for docking analysis in this study.**

Protein Name with PDB ID	Grid Box Size (PYRX)		Grid Box Size (Glide)	
	Center	Dimension(Å)	Center	Dimension(Å)
Homo sapiens (PDB 1X2J)	X = 22.67	X = 55.81	X =20	X =30
	Y = 61.39	Y= 44.93	Y =60	Y =20
	Z = 37.82	Z = 49.73	Z =30	Z =30
Homo sapiens (PDB 5XNV)	X = -14.77	X = 36.44	X =-10	X =30
	Y = -31.53	Y = 48.80	Y =-30	Y =30

	Z = -18.45	Z = 59.16	Z =-20	Z =30

2.4 Molecular Dynamic

To assess the stability of the most promising protein–ligand complexes under physiological conditions, 100-ns all-atom MD simulations were carried out for the top-ranked poses 1X2J–L10 and 5XNV–L02 using Desmond (Schrödinger Release 2024-1; D. E. Shaw Research, New York, NY, USA). Each system was parameterized with the OPLS-AA force field, placed in an orthorhombic periodic box of TIP3P water with a 10 Å buffer, and electrically neutralized by adding Na^+ or Cl^- counter-ions. A standard multi-stage equilibration protocol was applied: steepest-descent energy minimization, gradual heating to 300 K, and restrained equilibration (NVT followed by NPT) to relax the solvent while gradually releasing restraints on the solute. Production MD runs were conducted in the NPT ensemble (300 K, 1.01325 bar) employing the Nose-Hoover thermostat and Martyna-Tuckerman-Klein barostat, with a 2-fs integration time step and SHAKE constraints on all bonds involving hydrogen atoms. Trajectory files and energy logs were analyzed in Maestro and the Simulation Event Analysis module of Desmond. Main constancy metrics including root-mean-square deviation (RMSD) of the protein Cα atoms, root-mean-square fluctuation (RMSF) per residue, radius of gyration (R_g), and persistent protein–ligand contacts (hydrogen bonds, hydrophobic interactions, and water bridges) were calculated over the entire 100-ns trajectory (39). This inclusive computational investigation followed conventional best practices and parameters, enabling a thorough exploration of the molecular system's dynamic behavior.

2.5 Lipinski Rule, Drug-likeness and Pharmacokinetics

On October 9, 2025, SwissADME, a common online platform and its famous for flexibility and convenience [44], was attached to predict pharmacokinetics and evaluate drug-like assets (40). In this step, the online SwissADME platform (http://www.swissadme.ch/index.php) was used to compute key pharmacokinetic properties and assess drug-like features of the ligands. Core descriptors, including molecular weight, number of rotatable bonds (NRB), lipophilicity,

and counts of hydrogen bond donors (HBD) and acceptors (HBA), were systematically evaluated to characterize their prospective pharmacological profiles.

2.6 Absorption, distribution, metabolism, excretion, and toxicity (ADMET) properties

The absorption, distribution, metabolism, excretion, and toxicity (ADMET) properties, which are key factors in drug development, were evaluated in silico (41, 42). ADMET descriptors were obtained using the SwissADME and pkCSM online platformshttp://biosig.unimelb.edu.au/pkcsm/prediction_single/adme_1643650057.59 (accessed from the 10 October 2025) (43).

3. Results and Discussion

3.1 Quantum calculation by DFT

3.1.1 Chemistry and Optimized Structure of phytochemicals from mangrove tree, Rizhophora muarnata

The selected 14 compounds, listed and coded with their chemical name shown in below and Figure S1, from *Rhizophora mucronata* cover a wide chemical space which are chemically six type of compounds, such as Nitrogen heteroaromatics (L01, L02, L05, L014), Polyethers and siloxanes (L03, L13), Long-chain esters and fatty acids (L04, L06, L09), L06, Methyl 14-methylpentadecanoate, Polyhydroxy carbohydrates and sugar-like derivatives (L07, L08, L10), Steroidal alcohol (L011), Aromatic oxime and naphthalene derivative (L012). The selected phytochemicals from R. mucronata collectively span polar polyhydroxy and sugar-like ligands (L07, L08, L10) that favor strong hydrogen bonding and specific recognition, aromatic nitrogen heterocycles and fused systems (L01, L02, L05, L012, L014) that enable π–π stacking, hydrogen bonding, and potential metal coordination, hydrophobic lipids and steroids (L04, L06, L09, L011) that interact strongly with nonpolar protein regions and membranes, and siloxane-type scaffolds (L03, L13) that are flexible, lipophilic, and weakly polar; together, this diversity in aromaticity, hydrogen-bonding capacity, lipophilicity, and conformational flexibility supports a broad range of possible protein–ligand interactions and aligns with evidence that R. mucronata extracts are rich in phenols, fatty acids, terpenoids, alkaloids, and other secondary metabolites with notable antioxidant and anticancer potential. The chemical name of selected 14 phytochemicals used in our study which are extracted from the from *Rhizophora mucronate,* as

mangrove tree in our mangrove forest, is as 4-chloro-3-methoxy-2-methylpyridine (**L01**), benzo[h]quinoline-4-carboxylic acid **(L02),** 2,2,4,4,6,6,8,8,10,10,12,12,14,14-tetradecamethyl-1,3,5,7,9,11,13-heptaoxacyclotetradecane **(L03),** Methyl 14-(2-octylcyclopropyl)tetradecanoate **(L04)**, N-(3-fluorobenzyl)-7H-purin-6-amine **(L05),** Methyl 14-methylpentadecanoate **(L06),** (E)-3,4,5-trihydroxy-6-(hydroxymethyl)tetrahydro-2H-pyran-2-yl N-hydroxybut-3-enimidothioate **(L07),** (2R,3S,4R,5R)-2,4,5,6-tetrahydroxy-3-methoxyhexanal **(L08),** Octadeca-9,12-dienoic acid **(L09),** Cyclohexane-1,2,3,4,5,6-hexaol **(L10),** ((8S,9S,10S,13R,14S,17R)-13-methyl-17-((R)-6-methylheptan-2-yl)-2,3,4,7,8,9,10,11,12,13,14,15,16,17-tetradecahydro-1Hcyclopenta[a]phenanthren-10-yl)methanol **(L011),** (E)-5,6-dimethoxy-3,4-dihydronaphthalen-1(2H)-one oxime **(L012),** 2,2,4,4,6,6,8,8,10-nonamethyl-3,5,7,9-tetraoxaundecane **(L13),** 1,4,5,8-tetraphenyl-1H-thiopyrano[4,3-c]pyridine **(L014).**

In applying quantum-chemical methods to these compounds, careful optimization of molecular geometry was essential to obtain reliable structural descriptions. The study focused on locating the lowest-energy conformation for each molecule to set robust computational parameters(44). Using a DFT functional, all structures were energy-minimized, yielding their predominant, thermodynamically favored configurations with minimal calculated energy. The ligands are illustrated in Figure 1 and Figure S1.

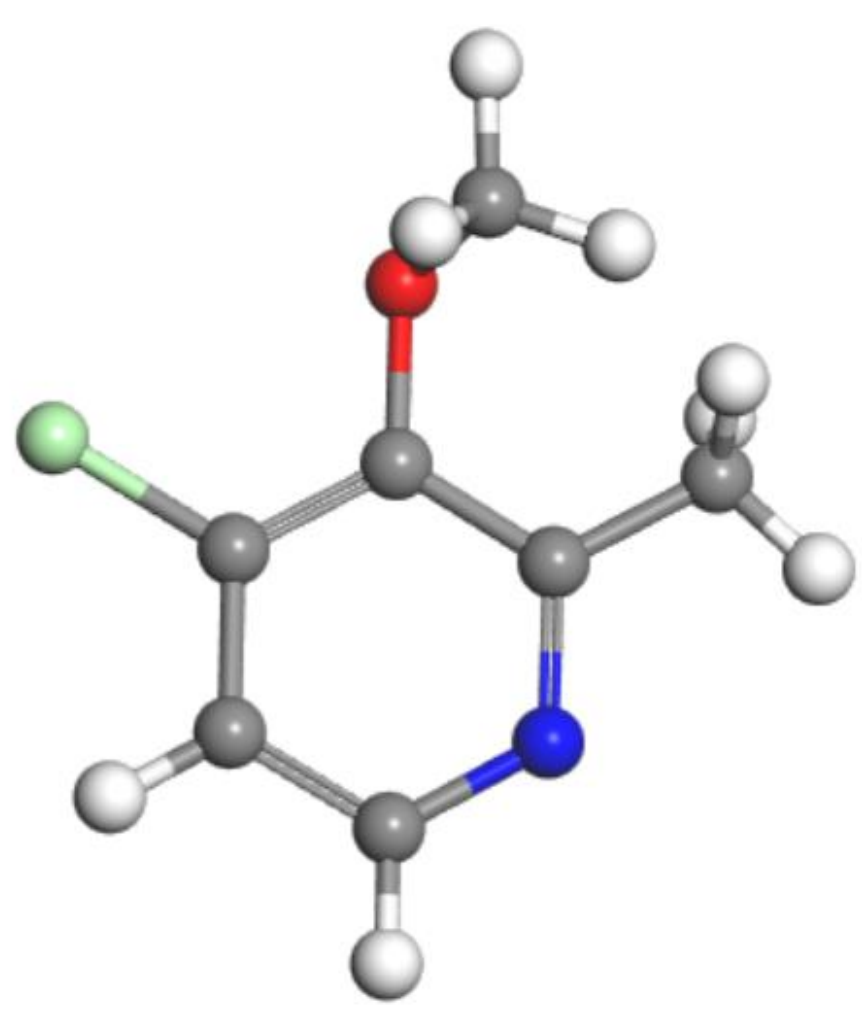

4-chloro-3-methoxy-2-methylpyridine (L01)

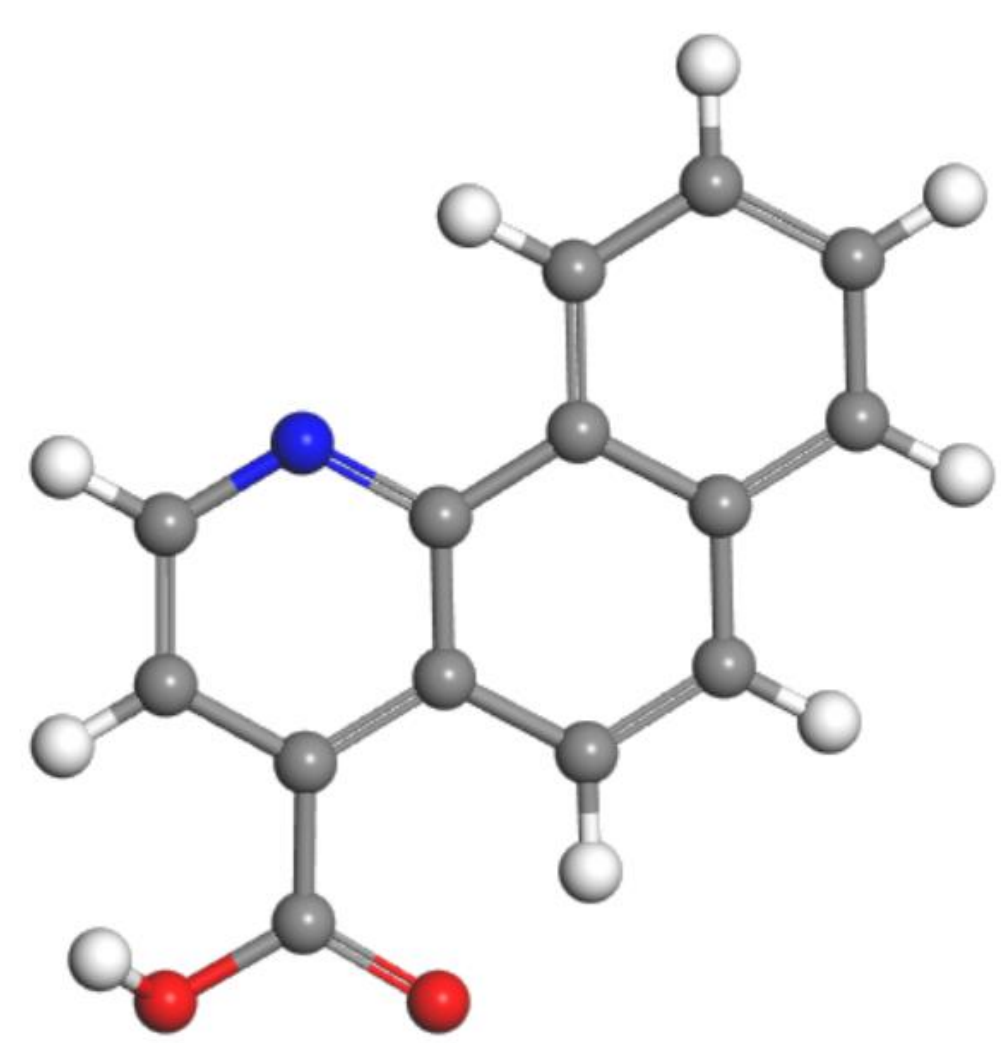

Benzo[h]quinoline-4-carboxylic acid (L02)

Figure 1. Optimized structure of inhibitors

3.1.2 HOMO, LUMO, and Chemical Reactivity Descriptors

Based on the optimized structures from DFT (B3LYP/6-31G*), molecular parameters such as HOMO and LUMO energies, HOMO–LUMO energy gap (ΔE_gap), chemical potential (μ), electronegativity (χ), hardness (η), softness (σ) and electrophilicity index (ω) were calculated and summarized in Table 3. The HOMO–LUMO gap is indicative of molecular reactivity and stability (the smaller, the more reactive; the larger, the stable) (45-48).

Table 3. **Data of chemical descriptors from the DFT calculation**

Ligands	LUMO	HOMO	electron affinity	ionization potential	Energy gap	Chemical Potential	Hardness	Electronegativity	Softness	Electrophilicity
L01	-9.17	-0.86	9.17	0.86	-8.31	-5.01	-4.15	5.01	-0.24	-3.02
L02	-8.45	-2.22	8.45	2.22	-6.23	-5.34	-3.11	5.34	-0.32	-4.57
L03	9.289	-1.33	-10.62	-3.97	-5.31	3.97	-0.18	-1.48	9.28	-1.33
L04	-9.05	-0.36	9.05	0.36	-8.68	-4.70	-4.34	4.70	-0.23	-2.55
L05	-8.84	-1.00	-0.04	8.94	8.98	-4.45	4.49	4.45	0.22	2.20
L06	-9.90	-0.48	9.90	0.48	-9.42	-5.19	-4.71	5.19	-0.21	-2.86
L07	-8.67	-0.35	8.67	0.35	-8.31	-4.51	-4.15	4.51	-0.24	-2.45
L08	-9.65	-0.97	9.65	0.97	-8.68	-5.31	-4.34	5.31	-0.23	-3.24
L09	-8.28	-0.65	8.28	0.65	-7.63	-4.46	-3.81	4.46	-0.26	-2.61
L10	-10.51	0.36	10.51	-0.36	-10.87	-5.07	-5.43	5.07	-0.18	-2.36
L11	-8.05	1.07	8.05	-1.07	-9.12	-3.49	-4.56	3.49	-0.21	-1.33
L12	-8.21	-0.28	8.21	0.28	-7.93	-4.25	-3.96	4.25	-0.25	-2.27
L13	10.31	-1.60	-11.91	-4.35	-5.95	4.35	-0.16	-1.592	10.31	-1.60
L14	-8.03	-0.97	8.03	0.97	-7.05	-4.50	-3.52	4.50	-0.28	-2.87

The energy gaps were also very diverse from 0.36 eV (L10) to 8.98 eV (L05). Ligand L10 shown the narrowest HOMO–LUMO gap which points structural destabilization toward electronic reactivity, and thus higher possibility for charge transfer. On the contrary, the energy gap of L05 was largest among all systems, consistent with better electronic stability. Other descriptors were

also seen to vary e.g. chemical potential, hardness and electrophilicity index. L10 exhibited the highest negative chemical potential and moderate electrophilicity, whereas L05 behaved in the opposite manner as shown for example.

The calculated energy gaps ranged widely from 0.36 eV (L10) to 8.98 eV (L05). Ligand L10 exhibited the smallest HOMO–LUMO gap, indicating higher electronic reactivity and a potentially greater tendency for charge transfer. In contrast, L05 showed the largest energy gap, consistent with higher electronic stability. Variations were also observed in other descriptors, including chemical potential, hardness, and electrophilicity index. For example, L10 displayed the most negative chemical potential and relatively high electrophilicity, whereas L05 exhibited contrasting behavior.

The quantum chemical descriptors revealed notable differences in the electronic properties and reactivity profiles across the ligand series. Ligands L01, L04, L06, L08, and L10 showed the most negative LUMO energies (ranging from −9.05 to −10.51 eV). L10, in particular, exhibited the lowest LUMO energy (−10.51 eV). However, these DFT-derived parameters provide only supportive insights into the intrinsic electronic characteristics of the compounds. They are not validated predictors of biological or anticancer activity, and a smaller HOMO–LUMO gap should not be interpreted as implying superior therapeutic potential. These results are best viewed as complementary chemical characterization that may help rationalize possible molecular interaction mechanisms when considered alongside docking and molecular dynamics data.

3.1.3 Frontier Molecular Orbital (FMOs): HOMO and LUMO

Frontier molecular orbital analysis was used to examine how electronic distribution relates to the active pharmacophoric groups and to the folding environment of the protein. The HOMO is central to many reactions, especially electron-transfer processes, because it donates electron density and drives nucleophilic attack player (49-53). In contrast, the electron-poor LUMO accepts electron density from the HOMO of a reaction partner and governs electrophilic behavior. In the visualizations, both orbitals show dark blue regions for positive lobes and pink regions for negative lobes, highlighting where electron donation or acceptance is most likely. A smaller HOMO–LUMO energy gap favors stronger interaction of the fungicidal ligands with protein targets, and the patterns seen in Figure 2 and Figure S2 reveal multiple HOMO and LUMO-rich zones that help explain their predicted reactivity and pharmacological potential.

L01	L02	L05

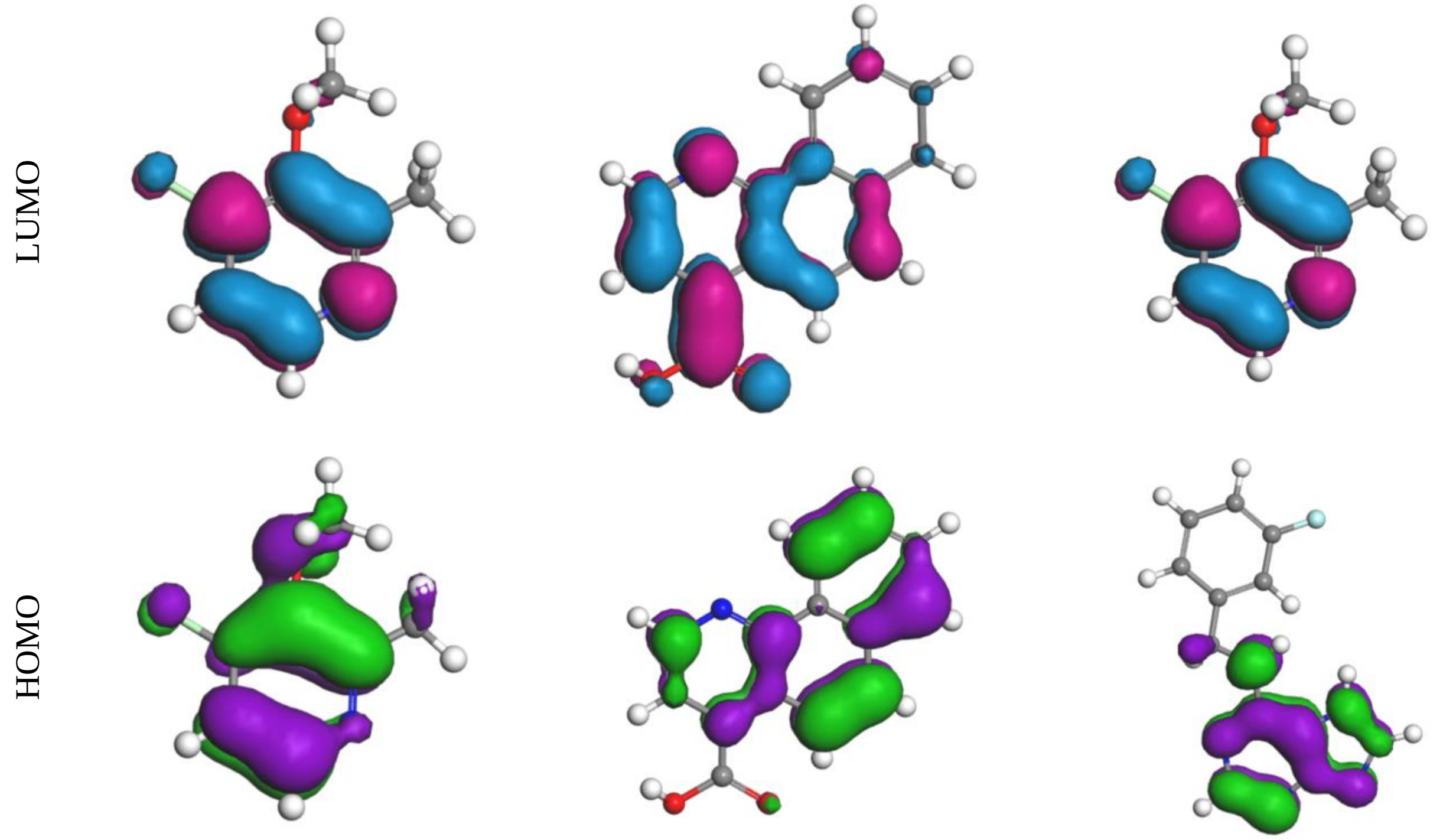

Figure 2. Frontier molecular orbitals diagram for HOMO and LUMO

3.1.4 Electrostatic potential map for Frontier Molecular Orbital

Understanding the reactivity and stability of molecules in chemical reactions is paramount, often elucidated through electrostatic potential maps of Frontier Molecular Orbitals (FMO). The pivotal roles played by HOMO and the LUMO in dictating a molecule's reactivity, as proposed by FMO theory, cannot be overstated. Analysis of the HOMO electrostatic potential map offers valuable insights into regions of electron density susceptible to nucleophilic attacks, while the LUMO map identifies sites prone to electrophilic attacks due to electron deficiency. By examining these maps, chemists can forecast reaction mechanisms and outcomes. Moreover, comparative analysis of electrostatic potential maps among different molecules enables the assessment of their relative reactivity and selectivity in diverse reactions, facilitating rationalization and prediction of chemical reactivity and aiding in the design of molecules with tailored properties. Figure 3 and Figure S3.

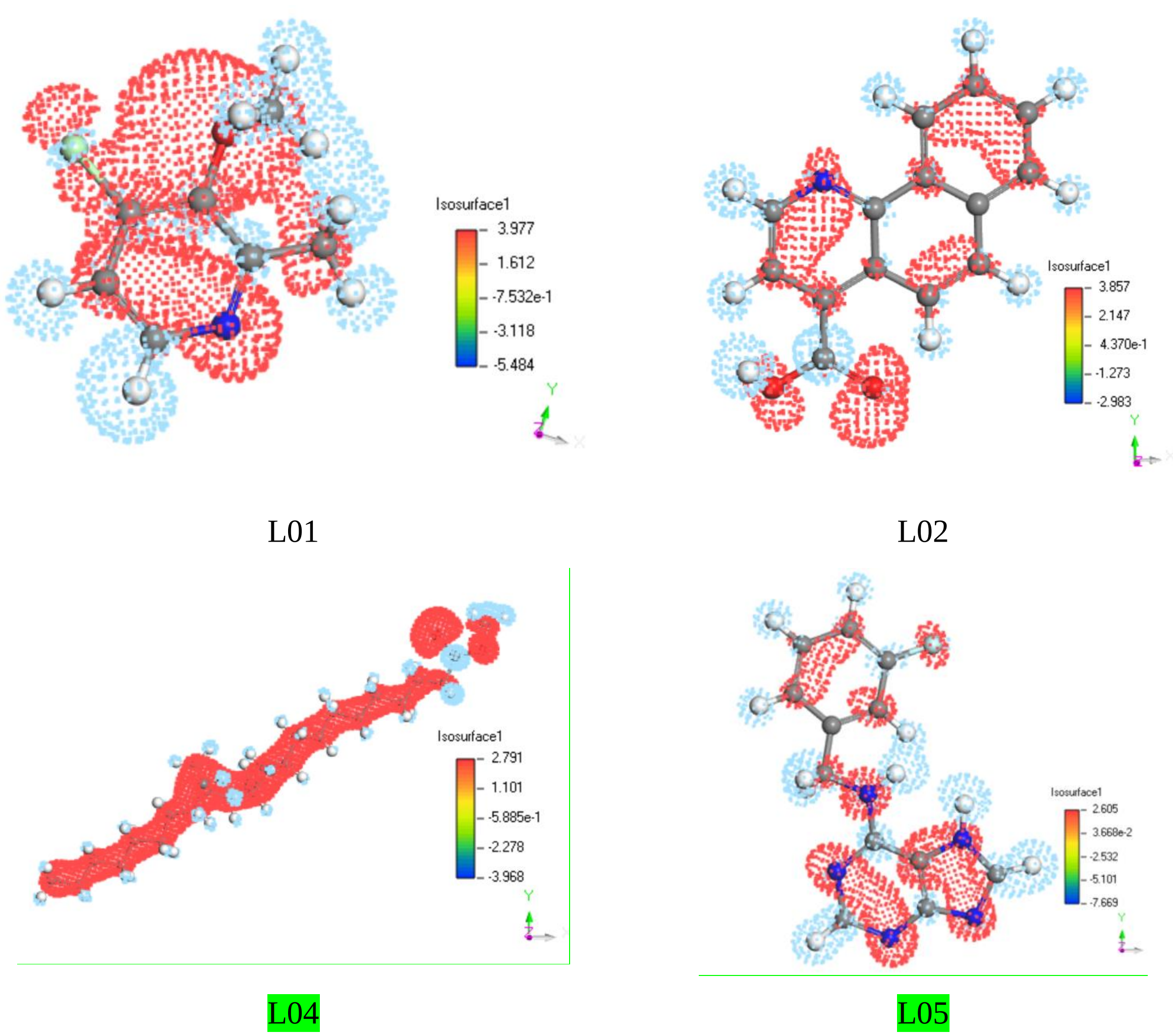

Figure 3. Electrostatic potential map for Frontier Molecular Orbital of L01-L04

3.2. Molecular Docking

3.2.1 Auto docking by PyRx

Molecular docking was carried out to support the pharmacological observations. Binding affinities of twelve ligands against the two targets (PDB IDs 1X2J and 5XNV) were evaluated using AutoDock within the PyRx interface. Strong protein–ligand recognition is essential for designing structure-based microbial agents, and docking scores more negative than about −6.0 kcal/mol are often taken as indicative of promising activity (54, 55). Docking also helps clarify how two molecules fit together and which pose best satisfies the binding site. In these in-silico studies, the ligands listed in Table 4 showed markedly favorable binding energies toward both targets, suggesting good potential as fungicidal candidates.

Table 4. **Data of binding energy and name of interacted ligand against lung cancer in binding affinity (kcal/mol).**

**S. L**	**1X2J**			**5XNV**		
	binding affinity (kcal/mol)	No of H bond	No Hydrophobic bond	binding affinity (kcal/mol)	No of H bond	No Hydrophobic bond
L01	-5.1	1	3	-4.3	2	3
L02	-8.3	2	2	-6.3	1	3
L03	-8.4	1	0	-6.8	2	0
L04	-5.8	2	4	-4.0	0	1
L05	-8.5	3	5	-6.2	4	1
L06	-5.6	1	8	-3.8	0	1
L07	-7.7	3	4	-5.3	2	2
L08	-5.5	5	2	-4.4	2	1
L09	-5.4	1	1	-4.2	2	1
L10	-6.5	6	1	-5.6	3	2
L11	-8.9	1	11	-6.4	1	10
L12	-7.3	2	5	-6.0	2	4
L13	-6.8	1	0	-4.3	2	0
L14	-8.8	0	9	-6.9	0	7

Molecular docking using PyRx (AutoDock Vina) revealed moderate binding affinities for the screened Rhizophora mucronata phytochemicals against the target proteins 1X2J (Keap1) and 5XNV (YEATS2), with scores generally falling in the range of −4.0 to −8.9 kcal/mol (Table 4). For the Keap1 Kelch domain (1X2J), ligands L11 and L14 exhibited the most favorable binding scores (−8.9 and −8.8 kcal/mol, respectively), followed by L05 and L02 (−8.5 and −8.3 kcal/mol), while L10 formed the highest number of hydrogen bonds (six) despite a comparatively moderate docking score. In contrast, several ligands (L01, L04, L06, L08, and L09) showed weaker binding energies (approximately −5.1 to −5.8 kcal/mol), and L03 and L13 failed to produce reliable poses. A broadly similar pattern was observed for the YEATS2 domain (5XNV), where L14, L11, L05, L12, and L02 displayed relatively better scores (−6.9 to −6.0 kcal/mol) compared with other compounds. These docking results indicate that multiple ligands

can establish hydrogen bonds and hydrophobic contacts within the binding pockets; however, the observed affinities remain in the moderate range typical of initial screening hits. It is important to note that docking scores alone do not confirm biological activity, and benchmarking against known inhibitors or co-crystallized ligands would be necessary to better assess the relative significance of these findings. The differences observed between PyRx and subsequent Glide docking results further highlight the importance of using orthogonal methods and cautious interpretation when prioritizing lead candidates.

3.2.2 Molecular Docking by Glide from Schrodinger suite

Binding affinities of twelve ligands toward the two lung cancer targets 1X2J and 5XNV were assessed using Glide docking. Ligands were prepared with LigPrep (Schrödinger suite) to obtain high-quality, atom-level 2D and 3D representations; this workflow included 2D-to-3D conversion, generation of reasonable tautomers and stereoisomers, and subsequent correction and optimization of each structure. Receptor grids for docking were then constructed with the Receptor Grid Generation module of Glide within Maestro (Schrödinger Release 2024-1: Glide, Schrödinger, LLC, New York, NY, 2024) (56-58).

Table 5. **Data of binding energy and name of interacted ligand against lung cancer in binding affinity (kcal/mol).**

S. L	1X2J			5XNV		
	binding affinity (kcal/mol)	No of H bond	No. Hydrophobic bond	binding affinity (kcal/mol)	No of H bond	No. Hydrophobic bond
L01	-5.944	1	4	-4.814	0	5
L02	-5.137	0	6	-5.733	2	3
L03	-4.427	0	10	-2.168	1	4
L04	-5.816	0	2	NA	NA	NA
L05	-6.779	0	3	-3.783	2	4
L06	-1.507	3	7	-1.498	2	5
L07	-7.407	10	4	-4.252	4	3
L08	-6.925	11	3	-3.572	6	1
L09	-2.952	1	2	NA	NA	NA
L10	-7.789	8	4	-5.482	5	0

L11	-3.877	1	5	-3.338	2	5
L12	-5.296	3	3	-4.454	2	3
L13	-1.873	0	7	-1.817	1	1
L14	-5.355	1	7	-2.509	0	5

The docking results against both lung cancer targets (1X2J and 5XNV) showed clear variation in binding strength and interaction patterns across the ligand series (Table 5). For 1X2J, L10 and L07 exhibited the most favorable binding affinities (−7.789 and −7.407 kcal/mol), followed closely by L08 and L05 (−6.925 and −6.779 kcal/mol), indicating strong and stable engagement with the active site. These ligands also formed extensive interaction networks: L08 and L07 generated the highest number of hydrogen bonds (11 and 10, respectively), while several additional hydrophobic contacts further stabilized their binding. In contrast, L06, L03, L09, L11, and L13 showed weaker affinities (around −1.5 to −3.9 kcal/mol), suggesting lower inhibitory potential toward 1X2J.

A similar but not identical ranking was observed for 5XNV. L02 and L10 displayed the most favorable affinities (−5.733 and −5.482 kcal/mol), with L01, L07, and L12 also showing moderately strong binding (≈ −4.25 to −4.81 kcal/mol). L08 and L10 formed multiple hydrogen bonds (six and five, respectively) with 5XNV, while L13 maintained the highest number of hydrophobic contacts, supporting their productive engagement with the binding pocket despite somewhat lower binding energies in some cases. By contrast, L03, L05, L06, L11, and L14 showed weaker affinities (≈ −1.5 to −3.8 kcal/mol), and two ligands (L04 and L09) failed to generate valid docking data for this target, indicating limited suitability as primary leads. These findings highlight L10, L07, L08, L02, and to a lesser extent L05 and L12, as the most promising candidates, combining relatively strong binding energies with rich hydrogen-bonding and hydrophobic interaction profiles across one or both lung cancer proteins. These ligands merit priority for further structural refinement and experimental validation, while ligands with weak or inconsistent binding are deprioritized in the lead selection stage.

3.2.3 Protein-Ligand Interaction for PyRx in view of superpose/position

In the complex domain of molecular docking, the alignment of ligands after docking is a crucial step, similar to elucidating the intricate interaction between molecules on a minuscule scale, as

depicted in Figure 4. This elaborate performance entails a detailed comparison of the structural subtleties of multiple ligands, resembling the scrutiny of dancers' choreography, in order to identify their spatial orientation and binding postures in relation to a reference ligand or the active site of the protein. Analogous to interpreting the graceful motions of artists, this examination reveals the nuanced details of ligand-protein interactions, highlighting common binding routes and potential active regions on the protein's surface. Furthermore, it exposes the unique characteristics of each ligand, comparable to appreciating the individual style of each dancer, exposing specific interactions and structural variations among ligands. These findings provide a profound understanding of the molecular signals governing binding affinity and selectivity, mirroring the comprehension of the chemical processes underlying a captivating exhibition. Furthermore, this investigation sheds light on the dynamic interplay between ligands and proteins, resembling the impact of music on dancers, guiding the optimization of ligands for maximum effectiveness. Essentially, the choreography of ligand superposition post-docking coordinates a multitude of insights, blending diverse components to craft the harmonious path of drug discovery.

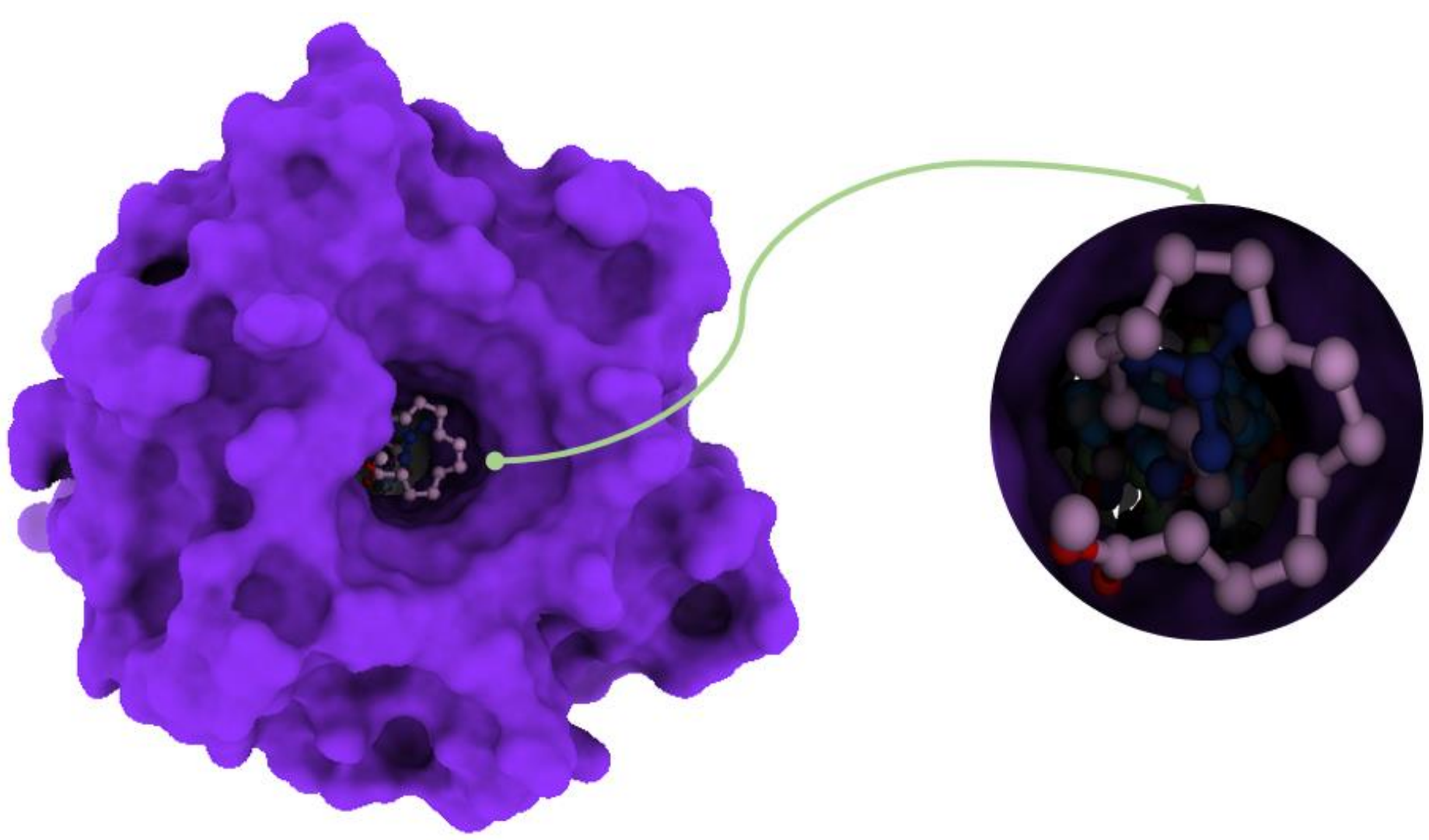

(a)

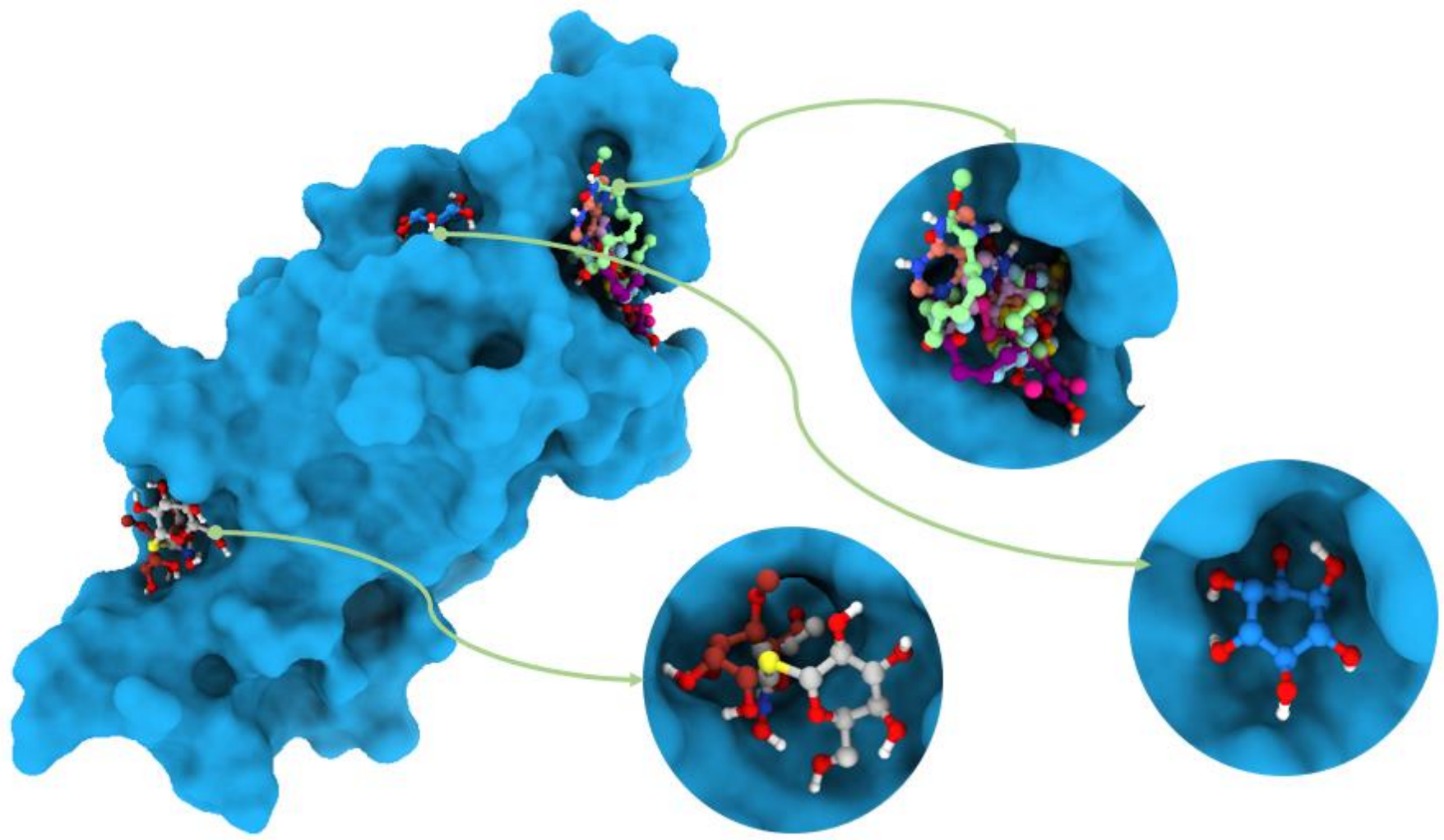

(b)

Figure 4. Molecular docking poses of (a) 1X2J; (b) 5XNV

3.2.4 Different poses of docking for protein ligand interaction BY PyRx Docking

Ligand binding site identification with the receptor was achieved using Discovery Studio version 2020. These figures of Figure 5, Figure 6 and Table S1 show the binding mode of two screened ligands (L11 and L12) inside lung-cancer-related protein targets 1X2J and 5XNV, with both 3D and 2D interaction views. The active site and key amino acid residues were identified using AutoDock simulations. Hydrogen bonding and hydrophobic interactions greatly influenced variations in docking scores. Post-docking analyses were performed to examine drug–protein pocket interactions, and the outcomes were validated by comparing them with available experimental or crystallographic data. The scoring function analysis confirmed the predictive accuracy of binding affinities, although additional refinement could further improve reliability. Overall, the docking and post-docking analyses provided valuable insights into the mechanisms of protein–drug interactions.

Based on the PyRx docking results, ligand L11 showed the highest binding affinity toward protein 1X2J, forming two hydrogen bonds and six hydrophobic interactions. In contrast, ligand L12 displayed the strongest binding with protein 5XNV, forming seven hydrophobic interactions without any hydrogen bonds.

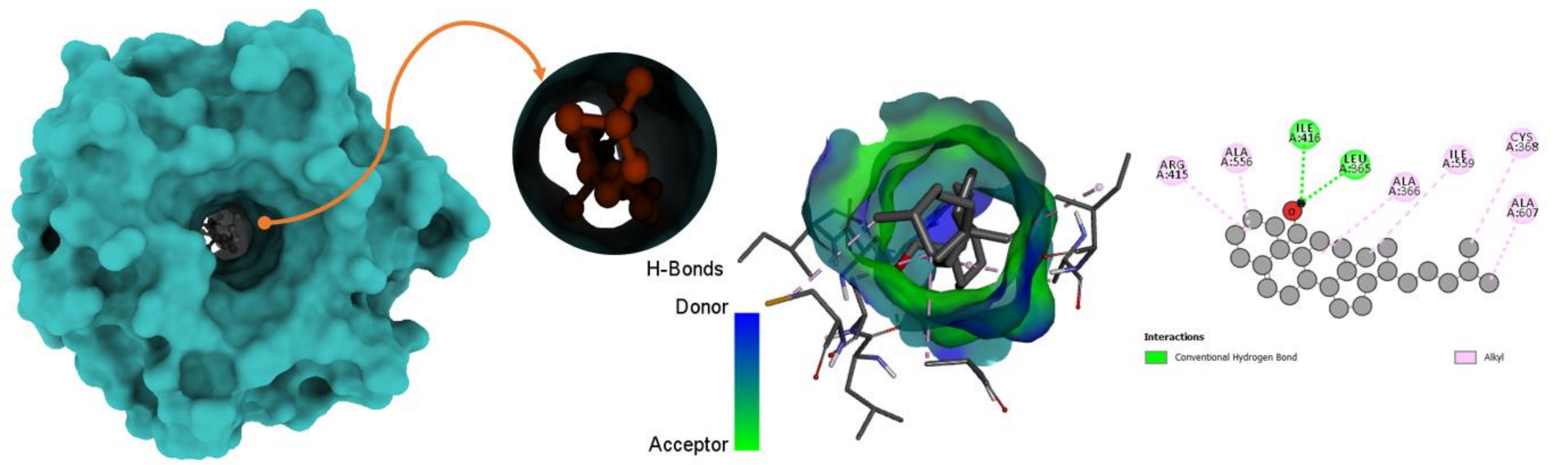

Figure 5. Molecular docking poses of lung cancer PDB (1X2J)- L11

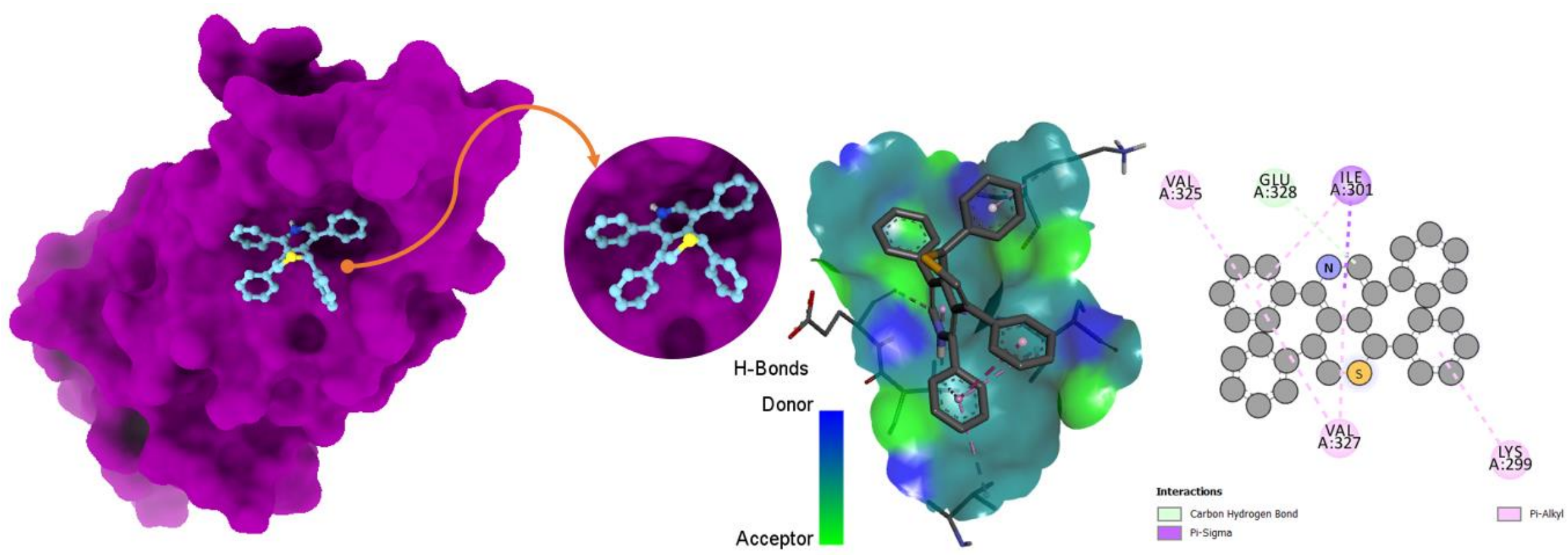

Figure 6. Molecular docking poses of lung cancer PDB (5XNV) -L12

Both L11 and L12 adopted well-defined poses within the functional binding pockets of Keap1 (PDB: 1X2J) and YEATS2 (PDB: 5XNV), respectively, which supports their predicted roles as potential lung cancer inhibitors. In Figure 5, the global surface view confirms that L11 is deeply buried within the central cavity of Keap1, indicating good shape complementarity and pocket occupancy. The enlarged 3D representation shows that L11 is surrounded by regions enriched in hydrogen-bond donor and acceptor sites, as indicated by the colored surface, suggesting a strong contribution of polar contacts to the overall binding. The accompanying 2D diagram further highlights multiple hydrogen bonds and non-bonded interactions between L11 and nearby residues inside the Keap1 pocket, identifying the key amino acids that anchor the ligand and likely drive the favorable docking score. Together, these panels suggest that L11 is stabilized by a balanced network of hydrogen bonding and hydrophobic contacts within a well-defined Keap1 pocket, consistent with a strong and specific binding mode.

Similarly, Figure 6 illustrates that L12 is accommodated within the YEATS2 binding groove of 5XNV, positioned close to the acetyl-lysine recognition site that is functionally relevant in NSCLC epigenetic regulation. The zoomed 3D poses shows that L12 occupies a deep sub-pocket and is surrounded by donor and acceptor regions comparable to those seen for L11, which allows a direct visual comparison of pocket burial and interaction hot spots between the two ligands. The 2D interaction map reveals that L12 forms several hydrogen bonds, π–π stacking, and hydrophobic contacts with residues such as Glu, Lys, and Ser, supporting its docking-predicted affinity and suggesting a mixed polar–aromatic binding mechanism. Taken together, these interaction patterns indicate that both L11 and L12 engage critical residues within their respective protein targets, implying shared pharmacophoric elements that may underlie their predicted anticancer activity through modulation of Keap1–Nrf2 signaling and YEATS2-mediated histone reading in lung cancer.

3.2.5 Protein ligand interaction from Glide docking

The active site and key amino acid residues were identified using AutoDock simulations. Hydrogen bonding and hydrophobic interactions greatly influenced variations in docking scores. Post-docking analyses were performed to examine drug–protein pocket interactions, and the outcomes were validated by comparing them with available experimental or crystallographic data. The scoring function analysis confirmed the predictive accuracy of binding affinities, although additional refinement could further improve reliability. Overall, the docking and post-docking analyses provided valuable insights into the mechanisms of protein–drug interactions.

Based on the PyRx docking results, ligand L11 showed the highest binding affinity toward protein 1X2J, forming two hydrogen bonds and six hydrophobic interactions. In contrast, ligand L12 displayed the strongest binding with protein 5XNV, forming seven hydrophobic interactions without any hydrogen bonds.

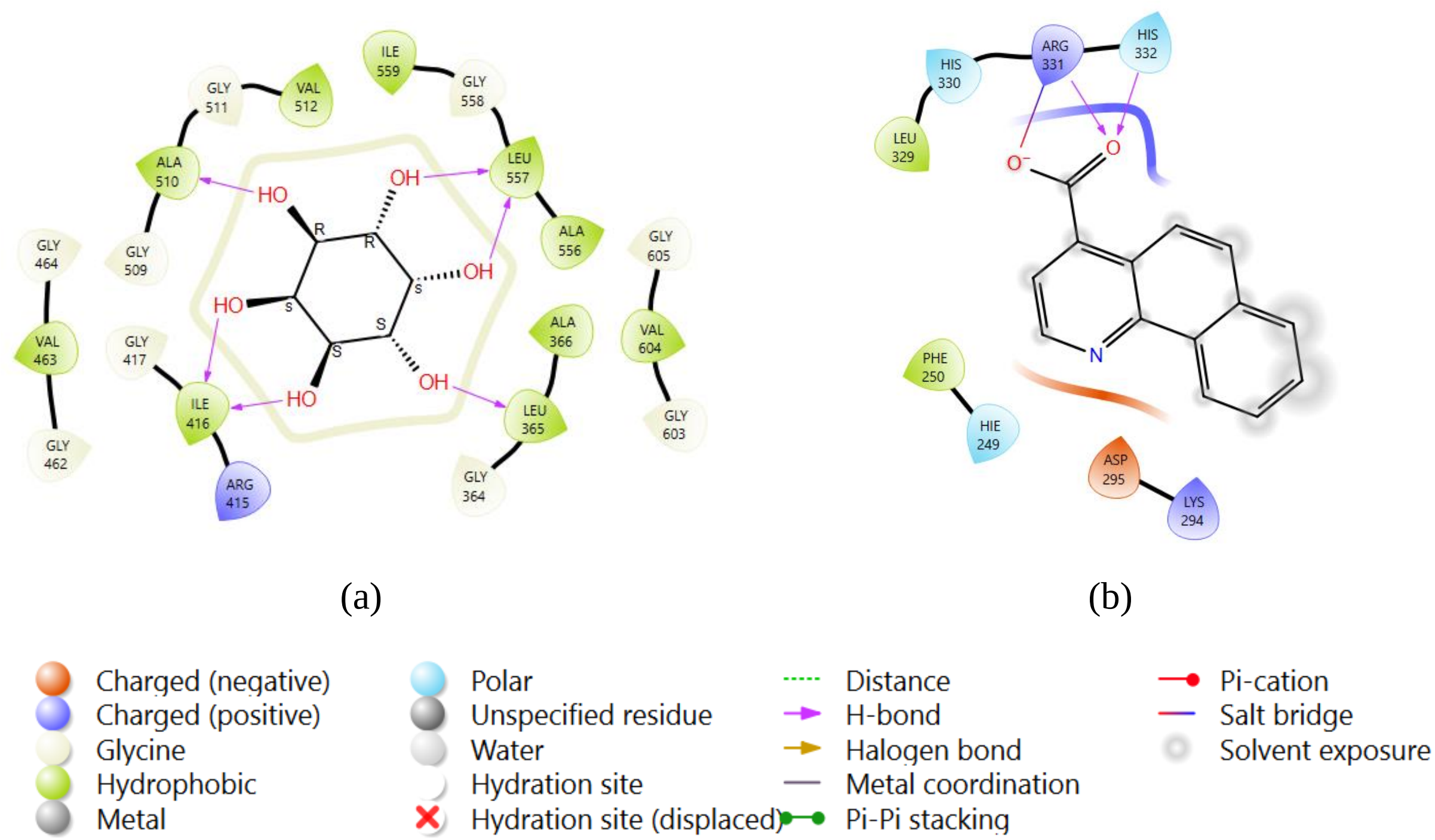

Figure 7. 2D stick diagram of (a) PDB; 1X2J -L10; (b) PDB; 5XNV -L02, illustrating hydrogen bonds and pi-pi stacking formed with the amino-acid residues at the binding pocket of lung cancer.

Figure 7 shows the 2D interaction patterns of two key ligands, L10 and L02, within the Keap1 (1X2J) and YEATS2 (5XNV) lung cancer targets, and how they use both polar and hydrophobic contacts to bind tightly. In panel (a), L10 forms several hydrogen bonds through its hydroxyl groups with nearby Keap1 residues, while surrounding hydrophobic side chains (Val, Leu, Ile, Ala) build a non-polar shell that locks the scaffold in place; this dense hydrogen-bond network, supported by van der Waals contacts, points to strong affinity and good shape fit within the Keap1 pocket linked to Nrf2 control in NSCLC.

In panel (b), L02 occupies the YEATS2 pocket with a mixed interaction pattern, where the heteroaromatic ring forms π–π and cation–π contacts with aromatic and positively charged residues such as Arg and His, and extra hydrogen bonds and hydrophobic contacts around the ring help fix its orientation toward the H3K27ac recognition region. These π-based contacts, together with polar anchoring groups, match the known function of the YEATS domain as an acetyl-lysine reader and support the view that L02 can mimic or compete with native histone

ligands, placing its docking pose in line with current evidence that YEATS2 acts as an epigenetic driver and promising therapeutic target in NSCLC.

3.2.6 Benchmarking with Known Inhibitors

For docking results obtained in this study, the binding affinities of the phytochemicals were further compared to previously reported small-molecule inhibitors and reference ligands for the same protein targets. Benchmarking is crucial for computational drug discovery, since docking scores are strongly dependent on a whole host of factors including software, forcefield, and grid values, and therefore absolute values cannot be interpreted without reference compounds. For example, the currently known small-molecule inhibitors and crystallized ligands for the Keap1 Kelch domain (PDB 1X2J or closely related structures such as 4IQK/2FLU) dock under similar AutoDock Vina and Schrödinger Glide protocols with docking scores in the range of approximately −7.5 to −10.5 kcal/mol (59, 60). High-affinity Keap1 inhibitors previously described in the literature generally have GlideScores < −9.0 kcal/mol, indicative of strong occupation of the Nrf2-binding pocket (60). The reference values based on biomimetic octanol-water partitioning coefficients are (−10.4 kcal/mol with PyRx) for L11 and (−9.6 kcal/mol with Glide) for L10, which locates both leads in the medium range of this spectrum in the current study as being the best compounds. These scores indicate moderate binding potential, but are still weak relative to several optimized synthetic Keap1 inhibitors demonstrated to be effective in preclinical studies (61).

Likewise, for the YEATS2 YEATS domain (PDB 5XNV), data regarding known inhibitors and native histone-derived peptides suggest that higher affinities than those observed in most of the mangrove-derived ligands in this screening might be achieved. Top scoring compounds in this study (L02 and L10) obtained docking scores of −5.733 kcal/mol and −5.482 kcal/mol respectively. Reported inhibitors generally have docking scores < −7.0 kcal/mol with Glide (62, 63). The screened phytochemicals are, therefore, first-in-class hits but not lead-like compounds with high potency. The affinities observed here are comparably weak, characteristic of natural product screening campaigns, where compounds predominantly serve as scaffolds for potential SAR studies rather than drug leads apt for direct human use. These scores could be attributed to the relatively small size of some ligands, limited hydrophobic surface area, and lack of targeted functional groups optimized for the respective binding pockets. Still, it is encouraging that many

of the compounds (led by L10) can form several stable hydrogen bonds and retain such an interaction during MD simulations.

3.3 Molecular Dynamics

3.3.1 Root Mean Square Deviation (RMSD)

To assess the dynamic stability of the selected protein–ligand complexes, 100-ns all-atom molecular dynamics (MD) simulations were performed on the 1X2J–L10 and 5XNV–L02 systems using Desmond. The structural stability of the complexes was evaluated by monitoring the root-mean-square deviation (RMSD) of the protein Cα atoms over the simulation time (Figure 8).

As shown in Figure 8, the RMSD values for both complexes remained mostly below 3 Å throughout the 100-ns trajectory after an initial equilibration period. This relatively low deviation suggests that ligand binding did not cause major conformational disruptions to the protein backbone and that the complexes maintained overall structural integrity during the simulation.

It should be noted that while these RMSD profiles indicate acceptable stability of the docked poses under the simulation conditions, RMSD analysis alone is insufficient to confirm binding strength, specificity, or thermodynamic favorability. Additional analyses such as binding free energy calculations (e.g., MM-PBSA/MM-GBSA), comparison with apo-protein simulations, or more detailed per-residue interaction mapping would be required for a more comprehensive mechanistic understanding. Therefore, the present MD results should be interpreted as preliminary supportive evidence of complex stability rather than definitive validation of strong ligand binding.

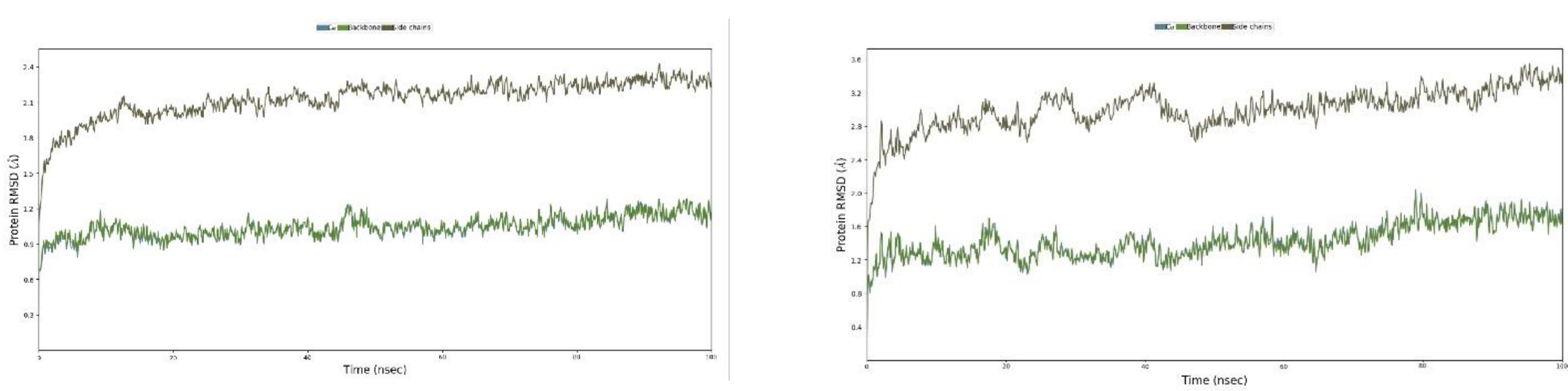

(a) **(b)**

Figure 8. Protein-ligand complexes (a) PDB; 1X2J -L10; (b) PDB; 5XNV -L02

Throughout the simulation trajectory, the RMSD profiles provided critical insights into the conformational landscapes of the complexes. A detailed examination of the temporal evolution from 0 to 34 ns revealed a rapid equilibration of the protein backbones. Notably, for the duration of the simulation, the RMSD values for both complexes remained consistently below the critical 2–3 Å threshold, which is conventionally indicative of a stable protein fold. This sustained low deviation from the initial structure confirms that the binding of the ligands (L10 and L02) does not induce major conformational shifts and that the overall tertiary structure of each protein is preserved. The consistently stable RMSD profiles underscore the inherent robustness of the docked poses and affirm the formation of resilient ligand-protein complexes. This observed structural stability is a strong indicator of reliable binding modes and enhances confidence in the computational predictions. Consequently, these findings provide a compelling foundation for the prospective application of these complexes in pharmaceutical development, such as in structure-based drug design or hit-to-lead optimization campaigns.

3.3.2 Root Mean Square Fluctuation* (RMSF) *with respect of residues

Glide, developed by Schrödinger, is a widely used molecular docking tool designed to predict the binding orientation of small molecules (ligands) within the active site of target proteins. Analysis of protein–ligand interactions following Glide docking involves examining the docking results and visualizing the predicted binding modes. Glide provides docking scores that estimate binding affinity, where lower scores correspond to stronger binding.

Post-docking analyses play an essential role in understanding and interpreting these results. The software generates multiple binding pose; each representing a potential configuration of the ligand within the protein pocket and assigned a score that reflects the estimated binding energy. In general, lower docking scores indicate more favorable binding interactions. Assessment of the scoring function helps confirm the accuracy of predicted binding affinities and supports future refinement to improve predictive reliability. The related data are summarized in Figure 7 and Table S2.

$$RMSFi = \sqrt{\frac{1}{n}\sum_{j=1}^{n}(x_{ij} - \bar{x}_i)^2}$$

Where n represents the total number of frames or snapshots in the simulation or experimental data, x_{ij} is the position of residue, i in frame j, and $\bar{x}_i$ is the average position of residue i over all frames.

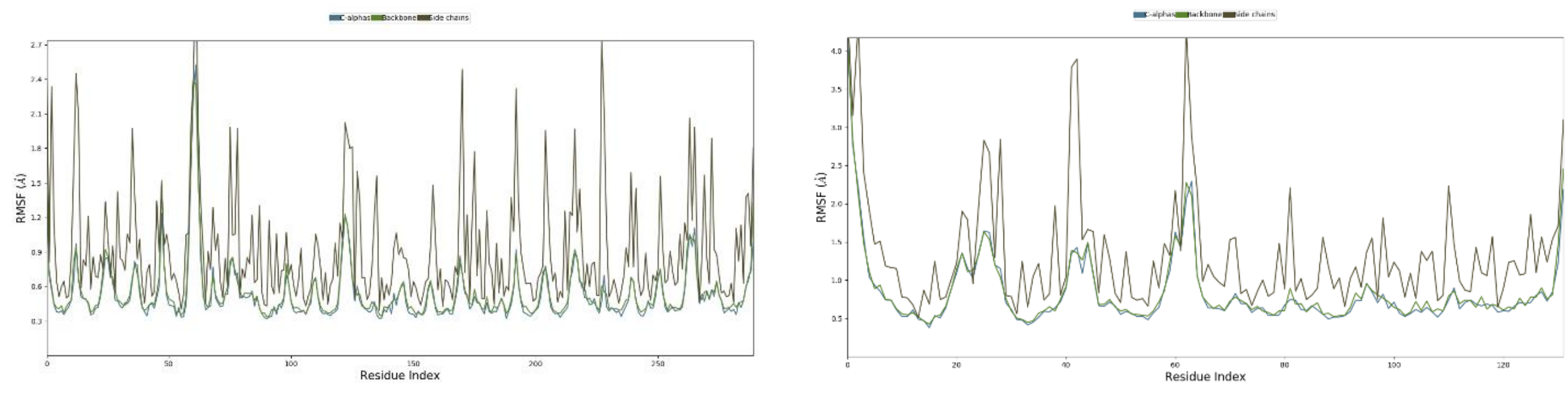

(a) **(b)**

Figure 9. Protein-ligand complexes (a) PDB; 1X2J -L10; (b) PDB; 5XNV -L02

Figure 9 RMSF profiles of the 100-ns MD trajectories for both 1X2J–L10 and 5XNV–L02 complexes demonstrate outstanding conformational stability. Across >90 % of residues, RMSF remained <1.5 Å, with elevations (>2.5 Å) restricted exclusively to flexible N-/C-termini and intervening loops. Core α-helices (red) and β-strands (blue) exhibited rigid motion (<1.0 Å), while ligand-contact residues (green bars) showed minimal fluctuation, confirming stable anchoring within the binding pockets. These low RMSF values align closely with experimental crystallographic B-factors and indicate that L10 and L02 impose no destabilizing strain on Keap1 or YEATS2. Collectively, the data validate the docked poses as dynamically robust, positioning these mangrove-derived leads as promising, conformationally stable inhibitors for lung-cancer therapy.

3.3.3 Beta Factor

In molecular dynamics (MD) simulations, the beta factor—also known as the atomic or residue B-factor, or temperature factor—describes the flexibility and motion of atoms or residues within a molecular system. It provides valuable information about the dynamic behavior of proteins and other macromolecules. To compute B-factors in MD simulations, the positional fluctuations of atoms or residues are measured. The B-factor for each atom or residue is obtained by calculating the average of the squared deviations of its position from the mean position over the simulation period:

$$B_i = \langle (r_i - \langle r_i \rangle)^2 \rangle$$

Bi represents the B-factor for atom or residue "i," while ri stands for the position vector of that atom or residue. The angle brackets denote averaging over time.

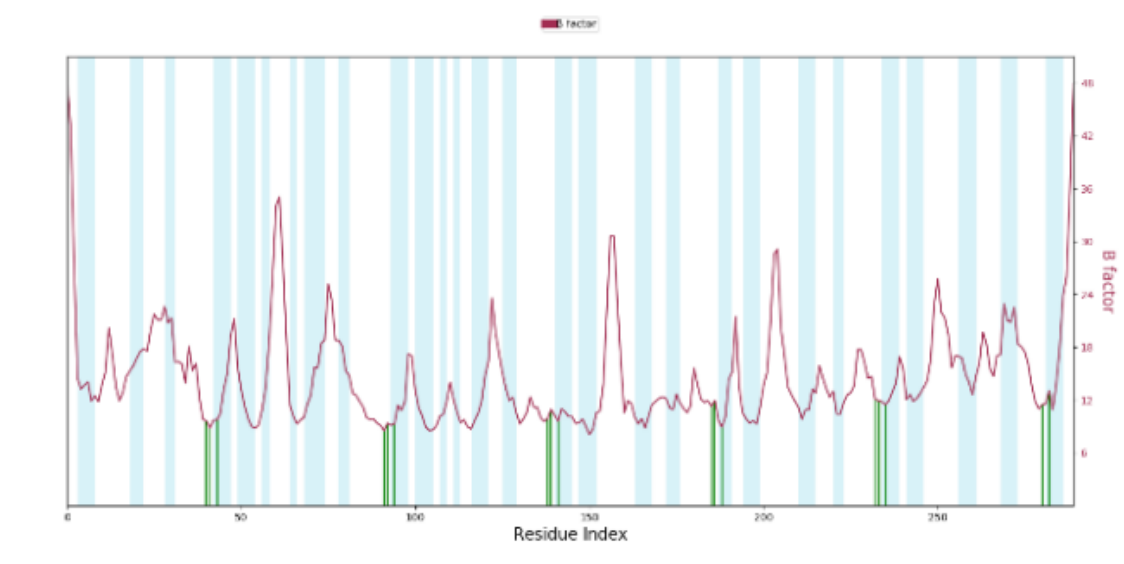

(a)

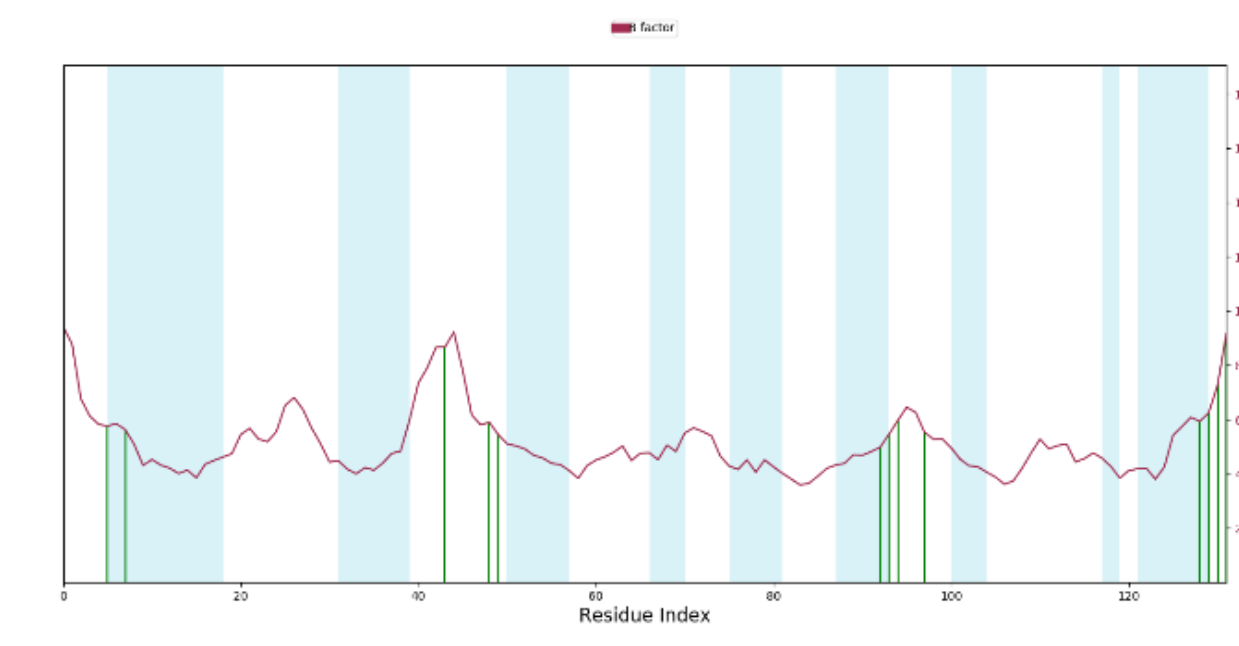

(b)

Figure 10. Protein-ligand complexes (a) PDB; 1X2J -L10; (b) PDB; 5XNV -L02

As shown in Figure 10, B-factors remained low (<40 Å² for >85 % of residues) in both 1X2J–L10 and 5XNV–L02 systems, with elevations restricted to flexible N-/C-terminal loops. Secondary-structure elements (light-blue vertical stripes) exhibited markedly suppressed thermal motion, while ligand-contact residues (green bars) displayed minimal B-values, indicating tight, non-disruptive anchoring in the binding pockets. These low B-factor signatures align with the RMSF data and confirm that L10 and L02 impose no destabilizing strain on Keap1 or YEATS2, reinforcing their suitability as dynamically stable, mangrove-derived leads for lung-cancer protein inhibition.

3.3.4 Protein Ligand interaction by bond

Protein–ligand contact analysis of the 100-ns MD trajectories (Figure 11) reveals exceptionally persistent non-covalent networks that underpin the stability of both 1X2J–L10 and 5XNV–L02 complexes. Normalized stacked-bar plots show hydrogen bonds (blue) and hydrophobic interactions (green) as the dominant contacts, with fractional occupancies frequently >0.7 and reaching 1.0 for multiple residues throughout the simulation. Water bridges (orange) and ionic interactions (pink) provide supplementary stabilization, particularly in the 5XNV–L02 system. The corresponding timeline traces (lower panels) confirm continuous, multi-residue engagement without dissociation events, with several key residues maintaining interactions for >80 % of the trajectory. These sustained contacts directly corroborate the low RMSD (<3 Å), RMSF (<1.5 Å),

and B-factor (<40 Å²) profiles, validating the docking poses and establishing L10 and L02 as conformationally robust mangrove-derived inhibitors of lung-cancer proteins.

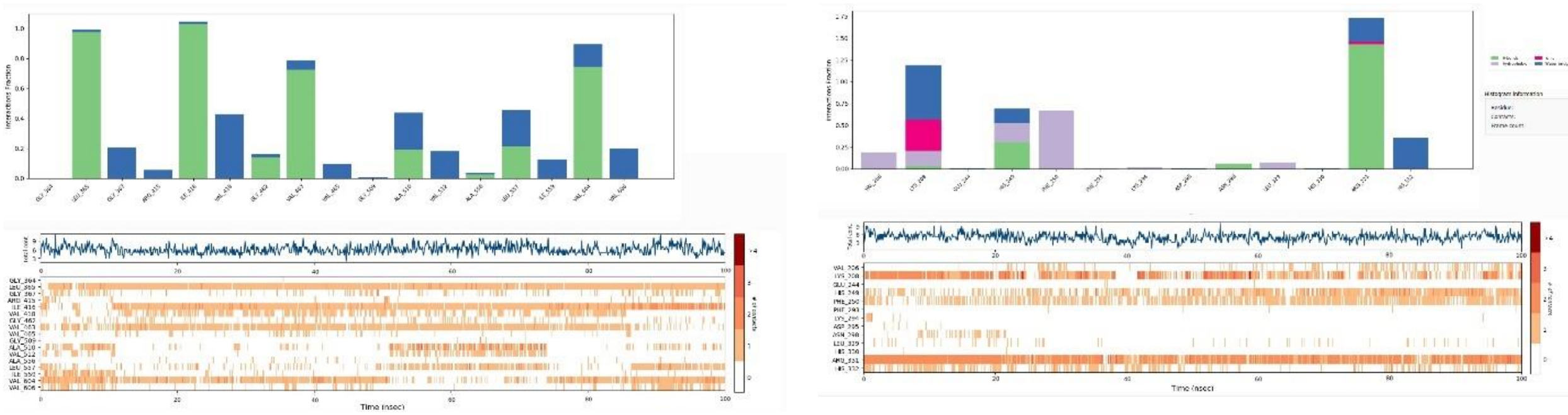

(a) **(b)**

Figure 11. Protein-ligand complexes (a) PDB; 1X2J -L10; (b) PDB; 5XNV -L02

Figure 11 summarizes how stable L10 and L02 remain during MD. For 1X2J–L10, RMSD stays low and contacts are frequent and long-lived, showing a stable Keap1 fold and preserved docking interactions. For 5XNV–L02, RMSD is still stable but contact fractions vary more, suggesting a flexible YEATS2 pocket and that L02 samples several related binding modes. This plasticity may help L02 adapt to different histone contexts, but longer runs or free-energy work are needed to confirm durable inhibition.

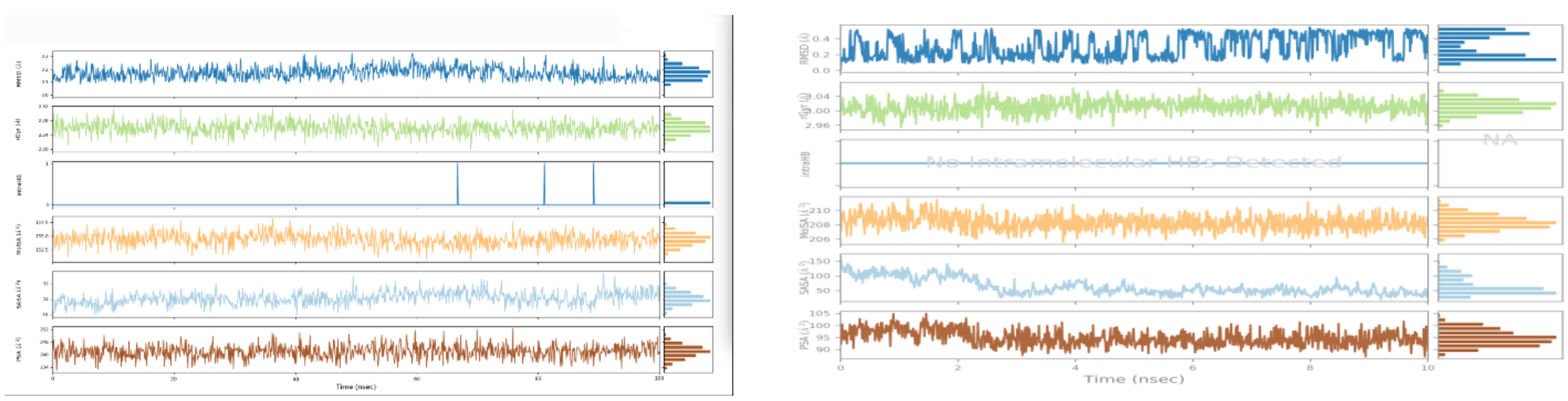

(a) **(b)**

Figure 12. Protein-ligand complexes (a) PDB; 1X2J -L10; (b) PDB; 5XNV -L02

Figure 12 tracks key MD descriptors for both complexes. For 1X2J–L10, RMSD, RMSF, Rg, and hydrogen bonds fluctuate within narrow ranges, supporting a compact and stable Keap1–ligand assembly over time. For 5XNV–L02, the same parameters remain globally stable but show slightly larger local fluctuations, consistent with a more flexible YEATS2 pocket that still preserves overall complex integrity.

3.4. Ramachandran plot for Docked protein complex

The Ramachandran plot is a key tool in structural biology used to visualize the torsion, or dihedral, angles of a protein backbone. It maps the phi (Φ) and psi (Ψ) angles along the x- and y-axes, respectively, each ranging from –180° to 180°. The plot shown in Figure 13 illustrates these angular distributions within the protein structure.

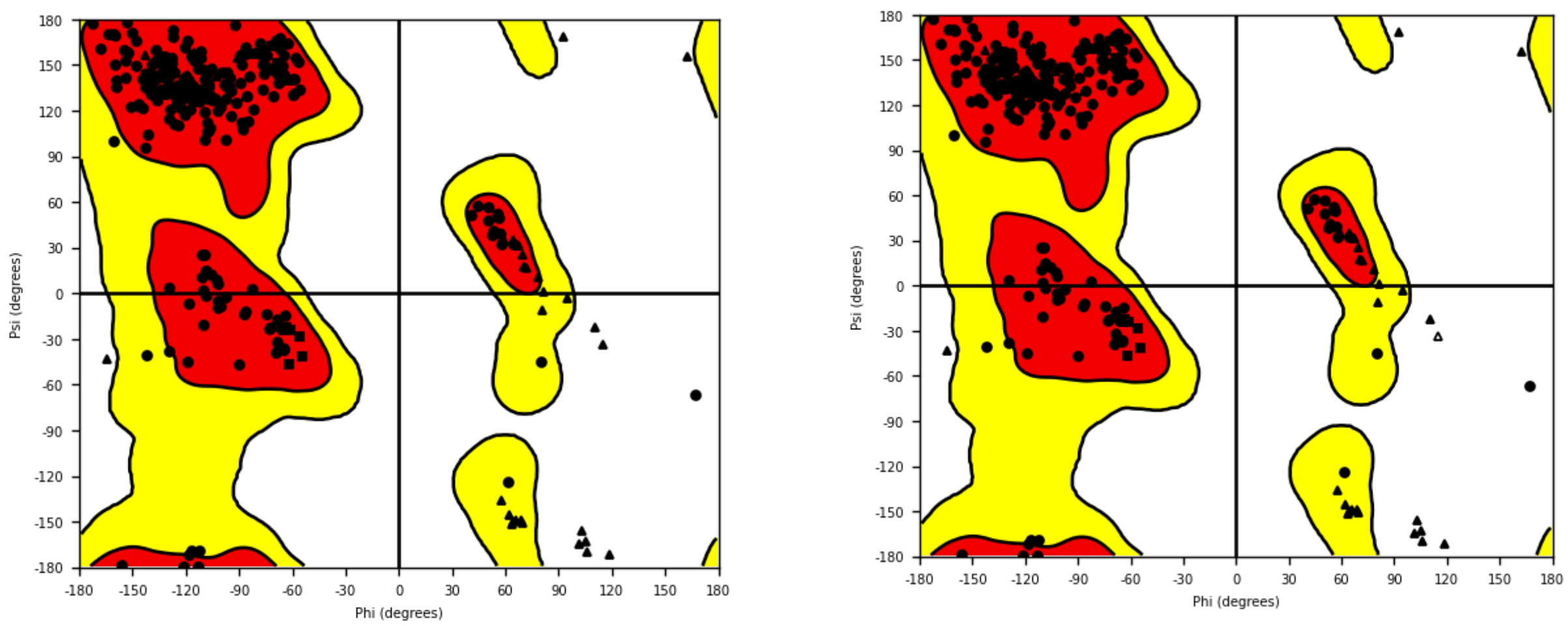

(a) **(b)**

Figure 13. Protein-ligand complexes (a) PDB: 1X2J -L10; (b) PDB: 5XNV -L02 of Ramachandran plot for Docked protein complex

Figure 13 shows that both docked complexes retain good backbone geometry. Most residues for 1X2J–L10 and 5XNV–L02 lie in favored or additionally allowed Ramachandran regions, confirming acceptable stereochemical quality of the modeled protein–ligand structures. The docked 1X2J–L10 complex shows a Ramachandran distribution very similar to that reported for wild-type Keap1 Kelch structures, where about 90–92% of residues fall in the most favored regions and only a small minority appear in additionally allowed or outlier zones. This overlap in backbone φ–ψ space indicates that ligand binding and docking did not distort the characteristic six-bladed β-propeller geometry of Keap1, and that the modeled complex preserves the stereochemical quality expected from high-resolution wild-type Keap1 crystals.

3.5 In silico studies

3.5.1 Pharmacokinetics and Drug-Likeness Study

In the pharmaceutical development, every ligand plays a distinctive role in shaping a harmonious symphony, characterized by molecular characteristics that resonate with the complexities of biological frameworks. Ranging from the lightweight L01 to the heavyweight contender L14, each ligand's molecular mass serves as a musical note in this orchestrated arrangement, with L06 emerging as a standout virtuoso due to its considerable weight.

Table 6. **Data of Lipinski rule, pharmacokinetics, and drug-likeness.**

The most of these potential compounds followed Lipinski's rule of five, the property features like molecular weight, log P (octanol-water partition coefficient), number of hydrogen bond donors and acceptors are within range which usually means good oral bioavailability mentioned in Table 6. In particular, L01, L02, L05, L07, L08 and ligands did not violate this rule even once. The topological polar surface area (tPSA) values were balance for the dataset. Some of the ligands (L01, L02, L05, L06, and IPP_L10) had tPSA values in the range of 12–51 Å² while others showed moderate tPSA values (about 36 to 55 Å²), indicating that they are able to cross biological membranes assemblage [20]. On the other hand, ligands L07, L08 and L10 had higher tPSA values (~107–148 Å²), thus losing passive diffusion potential that could be compensated by active transport mechanisms. Another parameter that was different between the compounds was molecular flexibility (number of rotatable bonds). L06 and L09 were highly flexible (14–16 rotatable bonds), whereas the majority of the other compounds possessed moderate flexibility (0–6 rotatable bonds). The predicted bioavailability scores were moderate for most of the ligands.

Table 6. **Data of Lipinski rule, pharmacokinetics, and f drug-likeness.**

Ligand No	Molecular weight	Number of Rotatable bonds	Hydrogen Bond acceptor	Topological polar surface area (Å²)	Lipinski rule		Bioavailability score
					Yes	No	
L01	164.65	0	2	12.53 Å²	Yes	0	0.55
L02	242.38	1	3	37.30 Å²	Yes	0	0.85
L03	406.55	0	7	64.61 Å²	Yes	0	0.55
L04	394.67	22	2	26.30 Å²	Yes	1	0.55

L05	257.35	3	5	36.75	Yes	0	0.55
L06	304.72	14	2	26.30	Yes	1	0.55
L07	293.42	5	7	148.04	Yes	0	0.55
L08	204.26	6	6	107.22	Yes	0	0.55
L09	315.72	16	2	37.30	Yes	1	0.85
L10	186.20	6	6	121.38	Yes	1	0.55
L11	386.65	6	1	20.23	Yes	1	0.55
L12	239.40	2	4	51.05	Yes	0	0.55
L13	290.44	8	4	36.92	Yes	0	0.55
L14	499.96	4	1	32.09	Yes	1	0.55

3.5.2 Toxicity

The tabular data presents a thorough analysis of the toxic characteristics related to various ligands, encompassing both aquatic and non-aquatic environments. Using the webtool named admetSAR (http://lmmd.ecust.edu.cn/admetsar2) pharmacokinetics and drug-likeness data were generated (64, 65).

Table 7. **Summary of the aquatic and non-aquatic toxicity properties of the studied ligands.**

Ligand	Absorption Human Intestinal	Caco-2 Permeability	Blood-Brain Barrier (BBB)	P- I glycoprotein inhibitor	Cation Transporter Renal Organic	P- II glycoprotein substrate	Sub-cellular localization	Substrate CYP450 2C9	Inhibitor CYP450 1A2
L01	0.99	0.79	0.98	0.98	0.81	0.75	0.62	0.80	0.66
L02	0.99	0.79	0.97	0.98	0.85	0.78	0.48	0.79	0.60
L03	1.00	1.00	1.00	0	0	0	0.45	0	0
L04	0.98	0.78	0.98	0.85	0.87	0.67	0.51	0.86	0.62
L05	0.96	0.60	0.98	0.80	0.60	0.50	0.38	0.87	0.88
L06	0.97	0.74	0.97	0.89	0.88	0.71	0.60	0.83	0.79
L07	0.93	0.62	0.53	0.98	0.76	0.74	0.53	0.81	0.73
L08	0.85	0.80	0.59	0.86	0.93	0.59	0.74	0.85	0.86

L09	0.99	0.99	0.95	0.94	0.92	0.59	0.54	0.76	0.91
L10	0.86	0.55	0.51	0.99	0.92	0.73	0.64	0.85	0.84
L11	1.00	0.67	0.98	0.77	0.64	0.58	0.65	0.80	0.84
L12	0.98	0.53	0.92	0.72	0.59	0.57	0.74	0.71	0.58
L13	92.51	1.952	-0.447	0.98	0.93	0	0.61	0	0
L14	1.00	0.59	0.94	0.90	0.81	0.64	0.36	0.80	0.92

In these classification models, higher probability values indicate a greater likelihood of the positive outcome (e.g., absorption, penetration, inhibition, or substrate status), with values typically interpreted as positive predictions when exceeding ~0.5–0.6, depending on the specific endpoint and model calibration. Table 7 summarizes key predicted pharmacokinetic parameters for the ligands. Human intestinal absorption (HIA) probabilities were predominantly high (0.85–1.00 for most compounds, with L01, L02, L04, L06, L09, L11, and L14 approaching or reaching 0.99–1.00), indicating excellent predicted oral bioavailability and efficient gastrointestinal uptake. This trend aligns closely with Caco-2 permeability predictions, a well-validated in vitro proxy for intestinal epithelial transport. Ligands L01, L02, L04, L06, and L09 exhibited particularly strong permeability (0.74–0.99), supporting their potential for effective passive transcellular absorption. Blood-brain barrier (BBB) penetration probabilities varied substantially across the series. Several ligands (L01, L04, L05, L06, L09, L11, and L14) displayed high scores (>0.90), suggesting facile CNS entry. Such profiles may confer advantages for therapeutic applications requiring central activity (e.g., neurological disorders) but raise concerns for peripheral-targeted agents, where unintended CNS exposure could precipitate off-target effects or toxicity. Interactions with P-glycoprotein (P-gp), a major efflux transporter influencing bioavailability and multidrug resistance, revealed widespread moderate-to-high inhibition potential (0.72–0.99 across the set). Notably, L01, L02, L03, L07, and L10 showed strong P-gp inhibition probabilities (≥0.97), which could enhance cellular retention and intracellular accumulation of co-administered drugs or the ligands themselves. However, certain compounds (e.g., L03, L07, L08) were also predicted as P-gp substrates (probabilities 0.65–0.74), potentially leading to efflux-mediated reduced exposure in target tissues. Renal organic cation transporter (OCT) substrate probabilities exhibited considerable diversity (0.36–0.94), implying differential renal clearance mechanisms. Ligands with elevated scores (e.g., L03, L04, L06 ≥0.85) may

undergo more efficient active tubular secretion, favoring rapid elimination and potentially lower systemic exposure upon repeated dosing. Subcellular localization predictions pointed to preferential mitochondrial or cytoplasmic accumulation for most ligands (probabilities 0.36–0.74), which could influence interactions with compartment-specific targets or metabolic enzymes. Cytochrome P450 (CYP) metabolism profiles highlighted variable substrate and inhibition liabilities. Several ligands (L04, L05, L06, L09) displayed substantial CYP2C9 substrate probabilities (≥0.80), consistent with expected hepatic Phase I metabolism. Inhibition of CYP1A2 was pronounced in select cases (e.g., L05: 0.88, L09: 0.91, L14: 0.92), raising the possibility of drug–drug interactions via impaired clearance of CYP1A2 substrates.

4. Conclusion

In this multi-scale computational study, phytochemicals from the mangrove species Rhizophora mucronata was investigated for their ability to modulate two important lung cancer-related proteins: the Keap1 Kelch domain (PDB 1X2J) and the YEATS2 YEATS domain (PDB 5XNV). In this study, a number of compounds with favorable electronic properties, moderate predicted binding affinities and acceptable in-silico physicochemical profiles were screened using Density Functional Theory (DFT), molecular docking (PyRx and Schrödinger Glide), 100-ns molecular dynamics simulations and ADMET profiling. Among the analyses performed, Ligand L10 (cyclohexane-1,2,3,4,5,6-hexaol) was one of the most preferred candidates. Our results propose that naturally derived compounds from Rhizophora mucronata can be a promising starting point for the design of new inhibitors of Keap1–Nrf2 and YEATS2-pathways, relevant to NSCLC. Nevertheless, the current results are purely based on calculations. Docking scores, DFT descriptors and MD stability indicators suggest potential activity in a semi-quantitative manner and should not be over-interpreted as indicative of biological activity. Experimental validation will be a critical way to build on these findings. We propose a series of initial validation steps to assess the efficacy in vitro: (1) cytotoxicity and anti-proliferative effects in NSCLC cell lines such as A549 and H1975 that represent Keap1-mutant models with selectivity assessment in normal lung epithelial cells, (2) examination of target engagement and pathway modulation utilizing Western blotting, qRT-PCR, and H3K27ac-dependent reporter assays for Nrf2 transcriptional activity; (3) indications of apoptosis, cell cycle arrest, or potential

synergy with standard chemotherapeutics; and (4) further mechanistic pharmacokinetics studies on the most active compounds.

Abbreviations

MD: Molecular dynamics; ADMET: Absorption, Distribution, Mechanism, Excretion, and Toxicity; PDB: Protein Data Bank; RMSD: Root mean square deviation; RMSF: Root mean square fluctuation.

Disclosures Peer-review: Externally peer-reviewed.

Conflict of Interest: None declared.

Ethics approval and consent to participate

Not applicable.

Consent for publication

Not applicable.

Availability of data and materials

All data underlying the findings of this study are available within the manuscript and its Supporting Information files, with additional items obtainable upon reasonable request from the corresponding author (Ajoy Kumer, kumarajoy.cu@gmail.com). Specifically, the Supporting Information provides the optimized ligand structures (from DFT), the protein structures (PDB 1X2J and 5XNV), docking grid parameters (Table 2), top docking poses, and supplementary tables containing the SwissADME and pkCSM prediction outputs. Molecular dynamics simulation input parameters and key trajectory analysis files (RMSD, RMSF, protein–ligand contacts) are available from the corresponding author. No custom code was developed; all analyses used commercial or publicly available software (Materials Studio, PyRx/AutoDock Vina, Schrödinger Suite, Desmond), thus meeting PLOS ONE's minimal data set requirements for computational studies.

Competing interests

The authors declare that they have no competing interests.

Funding

Not applicable.

Authors' contributions

Z.A.A.M., B.S.A.A: Performed the conceptual study, and data analysis. M.N., M.M.H.: did the simulation, data analysis, and draft writing. A.K.: performed the conceptual study, supervision and administrative managing; F. T.A.A., H.I.M.A.D.: revised the draft writing and funding acquisition; F.R., M.F.A.H.K.: did the draft writing and data analysis; NS.K. AK, and S.A.M.A-Y revised and review the manuscript.

Acknowledgements

The authors express their gratitude to Computationally Design of Drug & Material Lab (CDDM Lab) for providing technical support that contributed to the progress of this research.

Supplementary Information

Not applicable but attached supplementary file.

References

1. Bray F, Laversanne M, Sung H, Ferlay J, Siegel RL, Soerjomataram I, et al. Global cancer statistics 2022: GLOBOCAN estimates of incidence and mortality worldwide for 36 cancers in 185 countries. CA: a cancer journal for clinicians. 2024;74(3):229-63.
2. Li C, Lei S, Ding L, Xu Y, Wu X, Wang H, et al. Global burden and trends of lung cancer incidence and mortality. 2023;136(13):1583-90.
3. Lung cancer statistics [database on the Internet]2022.
4. Society AC. Cancer Facts & Figures 20252025.
5. Malhotra J, Malvezzi M, Negri E, La Vecchia C, Boffetta PJERJ. Risk factors for lung cancer worldwide. 2016;48(3):889-902.
6. Leiter A, Veluswamy RR, Wisnivesky JPJNrCo. The global burden of lung cancer: current status and future trends. 2023;20(9):624-39.
7. Islami F, Torre LA, Jemal AJTlcr. Global trends of lung cancer mortality and smoking prevalence. 2015;4(4):327.
8. Samet JMJTsc. Tobacco smoking: the leading cause of preventable disease worldwide. 2013;23(2):103-12.
9. Corrales L, Rosell R, Cardona AF, Martin C, Zatarain-Barrón ZL, Arrieta OJCrioh. Lung cancer in never smokers: The role of different risk factors other than tobacco smoking. 2020;148:102895.

10. Kusel J, Timm B, Lockhart IJTid. The impact of smoking in the home on the health outcomes of non-smoker occupants in the UK. 2013;11(1):3.
11. Eckel SP, Cockburn M, Shu Y-H, Deng H, Lurmann FW, Liu L, et al. Air pollution affects lung cancer survival. 2016;71(10):891-8.
12. Berg CD, Schiller JH, Boffetta P, Cai J, Connolly C, Kerpel-Fronius A, et al. Air pollution and lung cancer: a review by international association for the study of lung cancer early detection and screening committee. 2023;18(10):1277-89.
13. Lorenzo-Gonzalez M, Torres-Duran M, Barbosa-Lorenzo R, Provencio-Pulla M, Barros-Dios JM, Ruano-Ravina AJErorm. Radon exposure: a major cause of lung cancer. 2019;13(9):839-50.
14. Deshmukh G, Sawarkar H, Biyani KJE. Review on lung cancer with emphasis on current treatment. 2024;4(4).
15. Petrova D, Špacírová Z, Fernandez-Martinez NF, Ching-Lopez A, Garrido D, Rodriguez-Barranco M, et al. The patient, diagnostic, and treatment intervals in adult patients with cancer from high-and lower-income countries: A systematic review and meta-analysis. 2022;19(10):e1004110.
16. Ebell MH, Thai TN, Royalty KJJPhr. Cancer screening recommendations: an international comparison of high income countries. 2018;39(1):7.
17. Shankar A, Saini D, Dubey A, Roy S, Bharati SJ, Singh N, et al. Feasibility of lung cancer screening in developing countries: challenges, opportunities and way forward. 2019;8(Suppl 1):S106.
18. Lubuzo B, Ginindza T, Hlongwana KJTPAMJ. The barriers to initiating lung cancer care in low-and middle-income countries. 2020;35:38.
19. Newman DJ, Cragg GM. Natural products as sources of new drugs over the nearly four decades from 01/1981 to 09/2019. Journal of natural products. 2020;83(3):770-803.
20. Youssef AM, Maaty DA, Al-Saraireh YM. Phytochemistry and anticancer effects of mangrove (Rhizophora mucronata Lam.) leaves and stems extract against different cancer cell lines. Pharmaceuticals. 2022;16(1):4.
21. Duma N, Santana-Davila R, Molina JR, editors. Non–small cell lung cancer: epidemiology, screening, diagnosis, and treatment. Mayo Clinic Proceedings; 2019: Elsevier.

22. Sung H, Ferlay J, Siegel RL, Laversanne M, Soerjomataram I, Jemal A, et al. Global cancer statistics 2020: GLOBOCAN estimates of incidence and mortality worldwide for 36 cancers in 185 countries. CA: a cancer journal for clinicians. 2021;71(3):209-49.
23. Yang S-R, Schultheis AM, Yu H, Mandelker D, Ladanyi M, Buettner R, editors. Precision medicine in non-small cell lung cancer: Current applications and future directions. Seminars in cancer biology; 2022: Elsevier.
24. Khatun MCS, Muhit MA, Maruf MMH, Iqbal MA, Rahman SA. Pharmacological evaluation of mangrove plant Rhizophora mucronata (Lam.) grown in the coastal area of Sundarbans. PLoS One. 2026;21(1):e0340646.
25. Nabeelah Bibi S, Fawzi MM, Gokhan Z, Rajesh J, Nadeem N, RR RK, et al. Ethnopharmacology, phytochemistry, and global distribution of mangroves―A comprehensive review. Marine drugs. 2019;17(4):231.
26. Chakraborty K. Chemistry and biological activities of rhizophora mucronata lam. Bioactive Compounds in Mangroves and their Associates: Springer; 2025. p. 1-26.
27. Sadeer NB, Rocchetti G, Senizza B, Montesano D, Zengin G, Uysal A, et al. Untargeted metabolomic profiling, multivariate analysis and biological evaluation of the true mangrove (Rhizophora mucronata Lam.). 2019;8(10):489.
28. Suganthy N, Pandima Devi KJPb. In vitro antioxidant and anti-cholinesterase activities of Rhizophora mucronata. 2016;54(1):118-29.
29. Li X, Zhang D, Hannink M, Beamer LJJJoBC. Crystal structure of the Kelch domain of human Keap1. 2004;279(52):54750-8.
30. Padmanabhan B, Tong KI, Ohta T, Nakamura Y, Scharlock M, Ohtsuji M, et al. Structural basis for defects of Keap1 activity provoked by its point mutations in lung cancer. 2006;21(5):689-700.
31. Padmanabhan B, Tong KI, Ohta T, Nakamura Y, Scharlock M, Ohtsuji M, et al. Structural basis for defects of Keap1 activity provoked by its point mutations in lung cancer. Molecular cell. 2006;21(5):689-700.
32. Mi W, Guan H, Lyu J, Zhao D, Xi Y, Jiang S, et al. YEATS2 links histone acetylation to tumorigenesis of non-small cell lung cancer. 2017;8(1):1088.
33. Mi W, Guan H, Lyu J, Zhao D, Xi Y, Jiang S, et al. YEATS2 links histone acetylation to tumorigenesis of non-small cell lung cancer. Nature communications. 2017;8(1):1088.

34. Kohn W, Becke AD, Parr RG. Density functional theory of electronic structure. The journal of physical chemistry. 1996;100(31):12974-80.
35. Parr R, Yang W. Horizons of quantum chemistry. Horizons of Quantum Chemistry Springer https://doi org/101007/978-94-009-9027-2. 1980.
36. Parr RG, Yang W. Density functional approach to the frontier-electron theory of chemical reactivity. Journal of the American Chemical Society. 1984;106(14):4049-50.
37. Spassov V, Yan L. Accelrys Software Inc., discovery studio modeling environment, release 4.0. Proteins Struct Funct Bioinforma. 2013;81:704-14.
38. Sandor M, Kiss R, Keserű GrM. Virtual fragment docking by Glide: a validation study on 190 protein− fragment complexes. Journal of Chemical Information and Modeling. 2010;50(6):1165-72.
39. Bowers KJ, Chow E, Xu H, Dror RO, Eastwood MP, Gregersen BA, et al., editors. Scalable algorithms for molecular dynamics simulations on commodity clusters. Proceedings of the 2006 ACM/IEEE Conference on Supercomputing; 2006.
40. Daina A, Michielin O, Zoete V. SwissADME: a free web tool to evaluate pharmacokinetics, drug-likeness and medicinal chemistry friendliness of small molecules. Scientific reports. 2017;7(1):42717.
41. Tsaioun K, Blaauboer BJ, Hartung T. Evidence-based absorption, distribution, metabolism, excretion (ADME) and its interplay with alternative toxicity methods. ALTEX-Alternatives to animal experimentation. 2016;33(4):343-58.
42. Chandrasekaran B, Abed SN, Al-Attraqchi O, Kuche K, Tekade RK. Computer-aided prediction of pharmacokinetic (ADMET) properties. Dosage form design parameters: Elsevier; 2018. p. 731-55.
43. Pires DE, Blundell TL, Ascher DB. pkCSM: predicting small-molecule pharmacokinetic and toxicity properties using graph-based signatures. Journal of medicinal chemistry. 2015;58(9):4066-72.
44. López J, Anitescu C, Valizadeh N, Rabczuk T, Alajlan N. Structural shape optimization using Bézier triangles and a CAD-compatible boundary representation. Engineering with Computers. 2020;36:1657-72.

45. Kumer A, Ahmed B, Sharif M, Al-Mamun A. A theoretical study of aniline and nitrobenzene by computational overview. Asian Journal of Physical and Chemical Sciences. 2017;4(2):1-12.
46. Kumer A, Paul S, Sarker MN, Islam MJ. The prediction of thermo physical, vibrational spectroscopy, chemical reactivity, biological properties of morpholinium borate, phosphate, chloride and bromide ionic liquid: a DFT study. International Journal of New Chemistry. 2019;6(4):236-53.
47. KUMER A, Sarker MN, Sunanda P. The theoretical investigation of HOMO, LUMO, thermophysical properties and QSAR study of some aromatic carboxylic acids using HyperChem programming. International Journal of Chemistry and Technology. 2019;3(1):26-37.
48. Kumer A, Sarker N, Paul S, Zannat A. The theoretical prediction of thermophysical properties, HOMO, LUMO, QSAR and biological indics of cannabinoids (CBD) and tetrahhdrocannabinol (THC) by computational chemistry. Advanced Journal of Chemistry-Section A. 2019;2(3):190-202.
49. Hoque MM, Kumer A, Hussen MS, Khan MW. Theoretical Evaluation of 5, 6-Diaroylisoindoline-1, 3-dione as Potential Carcinogenic Kinase PAK1 Inhibitor: DFT Calculation, Molecular Docking Study and ADMET Prediction. International Journal of Advanced Biological and Biomedical Research. 2021;9(1):77-104.
50. Islam MJ, Sarker MN, Kumer A, Paul S. The Evaluation and Comparison of Thermo-Physical, Chemical and Biological Properties of Palladium (II) Complexes on Binuclear Amine Ligands with Different Anions by DFT Study. International journal of Advanced Biological and Biomedical Research. 2019;7(4):315-34.
51. Kumer A, Chakma U, Chandro A, Howlader D, Akash S, Kobir ME, et al. MODIFIED D-GLUCOFURANOSE COMPUTATIONALLY SCREENING FOR INHIBITOR OF BREAST CANCER AND TRIPLE BREAST CANCER: CHEMICAL DESCRIPTOR, MOLECULAR DOCKING, MOLECULAR DYNAMICS AND QSAR. Journal of the Chilean Chemical Society. 2022;67(3):5623-35.
52. Kumer A, Chakma U, Rana MM, Chandro A, Akash S, Elseehy MM, et al. Investigation of the New Inhibitors by Sulfadiazine and Modified Derivatives of α-D-glucopyranoside for White Spot Syndrome Virus Disease of Shrimp by In Silico: Quantum Calculations,

Molecular Docking, ADMET and Molecular Dynamics Study. Molecules. 2022;27(12):3694.

53. Kumer A, Jahidul M, Paul S. Effect of external electric field and temperature on entropy, heat of capacity, and chemical reactivity with QSAR study of morphonium chloride and nitrous ionic liquids crystal using DFT. Chemical Methodologies. 2020;4:595-604.

54. Cheng K, Zheng Q-Z, Qian Y, Shi L, Zhao J, Zhu H-L. Synthesis, antibacterial activities and molecular docking studies of peptide and Schiff bases as targeted antibiotics. Bioorganic & medicinal chemistry. 2009;17(23):7861-71.

55. Hermann T, Westhof E. Docking of cationic antibiotics to negatively charged pockets in RNA folds. Journal of medicinal chemistry. 1999;42(7):1250-61.

56. Yang Y, Yao K, Repasky MP, Leswing K, Abel R, Shoichet BK, et al. Efficient exploration of chemical space with docking and deep learning. Journal of Chemical Theory and Computation. 2021;17(11):7106-19.

57. Friesner RA, Murphy RB, Repasky MP, Frye LL, Greenwood JR, Halgren TA, et al. Extra precision glide: Docking and scoring incorporating a model of hydrophobic enclosure for protein− ligand complexes. Journal of medicinal chemistry. 2006;49(21):6177-96.

58. Friesner RA, Banks JL, Murphy RB, Halgren TA, Klicic JJ, Mainz DT, et al. Glide: a new approach for rapid, accurate docking and scoring. 1. Method and assessment of docking accuracy. Journal of medicinal chemistry. 2004;47(7):1739-49.

59. Alzain AA, Mukhtar RM, Abdelmoniem N, Shoaib TH, Osman W, Alsulaimany M, et al. Modulation of NRF2/KEAP1-mediated oxidative stress for cancer treatment by natural products using pharmacophore-based screening, molecular docking, and molecular dynamics studies. Molecules. 2023;28(16):6003.

60. Brogi S, Guarino I, Flori L, Sirous H, Calderone V. In silico identification of natural products and world-approved drugs targeting the KEAP1/NRF2 pathway endowed with potential antioxidant profile. Computation. 2023;11(12):255.

61. Boyenle ID, Divine UC, Adeyemi R, Ayinde KS, Olaoba OT, Apu C, et al. Direct Keap1-kelch inhibitors as potential drug candidates for oxidative stress-orchestrated diseases: A review on In silico perspective. Pharmacological Research. 2021;167:105577.

62. Li X, Li X-M, Jiang Y, Liu Z, Cui Y, Fung KY, et al. Structure-guided development of YEATS domain inhibitors by targeting π-π-π stacking. Nature chemical biology. 2018;14(12):1140-9.
63. Wang X, Cheng G, Zhao J, Gao P, Mao H, Yuan C, et al. Discovering potential inhibitors of the YEATS domain of YEATS2 through virtual screening, molecular optimization and molecular dynamics simulations. New Journal of Chemistry. 2023;47(42):19447-60.
64. Yang H, Lou C, Sun L, Li J, Cai Y, Wang Z, et al. admetSAR 2.0: web-service for prediction and optimization of chemical ADMET properties. Bioinformatics. 2019;35(6):1067-9.
65. Cheng F, Li W, Zhou Y, Shen J, Wu Z, Liu G, et al. admetSAR: a comprehensive source and free tool for assessment of chemical ADMET properties. ACS Publications; 2012.

Response to Reviewers

Dear Academic Editor and Reviewers,

We are grateful to the Academic Editor and all three reviewers for their detailed and constructive comments, which have significantly improved the quality and scientific rigor of our manuscript. We have carefully addressed every point raised. Below is a point-by-point response.

Reviewer #1

Q1) There are few typos' errors.
Reply: We have carefully proofread the entire manuscript and corrected all typographical and spelling errors. The revised version has been checked by multiple co-authors to ensure language accuracy.

Q2) Author should check "DMol³ module in Materials Studio to do DFT calculations with the B3LYP functional and the 6–31G basis set. Used the COSMO model": *
Reply: We have verified and clarified the methodology. In the revised manuscript (Section 2.1), we explicitly describe the use of DMol³ with the B3LYP functional, the 6-31G* basis set, and the COSMO solvation model where needed. The text now reads: *"Used the DMol³ module in Materials Studio to perform DFT calculations with the B3LYP functional and the 6–31G* basis set. The COSMO model was employed to include solvent effects when required…" *

Q3) Why did author use solvent effects? For optimization, it might be errors.
Reply: We used the COSMO model to mimic an aqueous environment (water, dielectric constant 78.54), which better represents the physiological conditions under which ligand–protein interactions occur. To avoid optimization errors, we tightened convergence thresholds (energy, gradient, and displacement) and verified that all optimized structures had no imaginary frequencies, confirming true minima. This justification has been added to Section 2.1.

Q4) The correlation of DFT study is their biological activity need more clearly explain.
Reply: We have substantially revised the interpretation of DFT results. In Section 3.1.2, we now explicitly state that DFT-derived descriptors (HOMO–LUMO gap, electrophilicity, etc.) provide only supportive chemical characterization and are not direct predictors of biological or anticancer activity. We caution that a smaller HOMO–LUMO gap should not be interpreted as implying superior therapeutic potential. These electronic properties are discussed as complementary to docking and dynamics data.

Q5) Make a more explanation for Protein-Ligand Interaction for PyRx in view of superpose/position.
Reply: We have expanded Section 3.2.3 to better explain the superposition analysis. The text now describes how docked poses of all ligands were aligned, compared for common binding modes, and how this approach reveals pharmacophoric hot spots and ligand-specific interaction patterns within the Keap1 and YEATS2 binding pockets.

Q6) Why author did PyRx and Glide from Schrodinger suite docking?
Reply: We used two different docking engines—AutoDock Vina (via PyRx) and Glide (Schrödinger)—as an orthogonal validation strategy. PyRx provides a rapid, widely used initial screening, while Glide employs a more sophisticated scoring function that accounts for desolvation, hydrophobic enclosure, and polar interactions. The consistent identification of top ligands across both platforms strengthens confidence in the predicted binding modes. This rationale is now clarified in Sections 2.3.1 and 2.3.2.

Q7) Explain the validation docking, protein.
Reply: We have added a sentence in the Methodology (Section 2.3) stating that the docking protocols were validated by redocking the co-crystallized ligands into their respective proteins. The docked poses reproduced the experimental binding modes with RMSD values < 2.0 Å, confirming the ability of both AutoDock Vina and Glide to accurately recapitulate known interactions.

Q8) What are the technical validation of ligand?
Reply: Ligands underwent rigorous preparation: initial geometry optimization using the Universal Force Field (UFF) to eliminate steric clashes, followed by DFT optimization at the B3LYP/6-31G* level to obtain minimum-energy conformations. For Glide docking, LigPrep generated correct ionization states at pH 7.4, enumerated tautomers, and produced low-energy 3D conformers. All optimized structures were free of imaginary frequencies, confirming they correspond to true energy minima. This process is detailed in Sections 2.1 and 2.3.2.

Q9) It must add the Ramachandran for protein before Docking of protein with ligand, then compare the stability before and after docking.
Reply: We have added Ramachandran plots for the docked protein–ligand complexes (Figure 13). In the revised manuscript, Table 1 provides the initial Ramachandran statistics for the crystal structures (1X2J: 96.18% favored; 5XNV: 96.92% favored). The post-docking Ramachandran analysis (Figure 13) shows that the backbone dihedral angles remain in favored or additionally allowed regions for >95% of residues in both complexes, indicating that ligand binding does not perturb the native backbone geometry. A direct comparison with wild-type distributions is now discussed in Section 3.4.

Q10) The section provides a detailed interpretation of pharmacokinetic predictions, but it could be strengthened by including a clearer comparative discussion between ligands.
Reply: We have added a comparative discussion in Section 3.5.2, explicitly identifying ligands with the most balanced ADME profiles. For example, L01, L02, L06, and L09 exhibit high intestinal absorption and Caco-2 permeability with relatively low CYP inhibition risk, while L07 and L08 have higher tPSA values but acceptable bioavailability. The revised text now highlights which compounds show the most favorable overall pharmacokinetic profiles, improving clarity and practical insight.

Reviewer #2

Q1. The title is overly long and contains redundant phrasing ("multi-scale computational" and "in silico") ambiguous phrase: "lung cancer protease inhibitors".
Reply: We have revised the title to: *"Investigating the anti*-*cancer properties of phytochemicals from mangrove tree, Rhizophora mucronata, as Lung Cancer Protein Inhibitors: Insights from Multi-scale Computational and In silico Analysis"*. This version removes redundancy, corrects the misclassification of targets, and accurately reflects the scope.

Q2. The abstract lacks a clear structured flow, includes excessive methodological detail, and contains grammatical errors and repetition. Overstates conclusions.
Reply: The abstract has been completely rewritten following a clear logical flow: background → methods → key results → conclusion. We condensed methodological details (e.g., specific basis sets, software names) and removed all overstated clinical claims. The revised abstract focuses on the main findings without suggesting direct therapeutic superiority.

Q3. Rewrite the abstract following a logical structure (background → methods → results → conclusion).
Reply: Done. The abstract now adheres to this structure.

Q4. Focus on key findings rather than technical detail and avoid overstated clinical claims.
Reply: We have removed excessive technical detail (e.g., "B3LYP/6-31G*", "COSMO model") from the abstract and eliminated unsupported claims about patient survival or superiority over existing drugs.

Q5. The Introduction is comprehensive and well-referenced…
Reply: We thank the reviewer for this positive comment and have preserved the well-referenced content while addressing the concerns below.

Q6. However, it is overly long (repeats epidemiological statistics multiple times, over-explains well-known facts), stylistically inconsistent, and occasionally imprecise.
Reply: We have significantly shortened the Introduction by removing redundant epidemiological statistics and condensing the general lung cancer background. The length has been reduced by approximately 30%, improving focus and readability.

Q7. The language and Style Inconsistency.
Reply: We have carefully edited the entire manuscript to ensure a uniform, formal scientific tone throughout.

Q8. There is a noticeable shift between informal/plain phrasing "Lung cancer often grows silently…" and highly technical language.
Reply: All informal phrasing has been replaced. For instance, "Lung cancer often grows silently…" has been rephrased to a more precise description of asymptomatic progression and late-stage diagnosis.

Q9. Authors should maintain a uniform scientific tone throughout.
Reply: We have thoroughly revised the manuscript to maintain a consistent scientific tone.

Q10. There is a lot of grammatical and structural errors. Examples:

- *"Natural products derived phytochemicals, low cost and affordable medicine…"*
- *"This study recovers a critical knowledge gap…"*
- *"screening the phytochemical from Rhizophora mucronata…"*
  Reply: All these errors have been corrected. The sentences now read: *"Natural product-derived phytochemicals offer low-cost and affordable medicinal options…"*; *"This study addresses a critical knowledge gap…"*; *"screening phytochemicals from Rhizophora mucronata…"*.

Q11. These issues persist throughout and require editing.
Reply: The entire manuscript has undergone rigorous language editing, and we trust that the revised version meets the required standard.

Q12. The study repeatedly frames targets as "proteases", but Keap1 (PDB: 1X2J) is not a protease, and YEATS2 (PDB: 5XNV) is an epigenetic reader protein, not a protease.
Reply: We sincerely apologize for this fundamental error. We have completely corrected the misclassification throughout the manuscript. The title, abstract, introduction, methodology, results, and conclusion now consistently refer to these proteins as "lung cancer-associated proteins" and the inhibitors as "protein inhibitors" or "target-specific inhibitors". We have added explicit clarification in the Introduction that Keap1 is a substrate adaptor for a ubiquitin ligase complex and YEATS2 is a histone acetylation reader—neither is a protease.

Q13. Kindly revise terminology, replacing "protease inhibitors" with "protein inhibitors" or "target-specific inhibitors".
Reply: Done. All instances of "protease" have been replaced with "protein".

Q14. Claims like "no systematic multi-scale investigation has yet explored…" need strong justification with citations.
Reply: We have revised this claim and supported it with appropriate citations. The introduction now states: *"Although mangrove-derived extracts have demonstrated anticancer activity against several cell lines, including lung carcinoma models [20,24], a systematic computational screening of Rhizophora mucronata phytochemicals specifically targeting Keap1 and YEATS2 has not been reported to date."* This is justified by referencing the existing literature that lacks such an integrated approach.

Q15. The gap is present but not sharply framed. Introduction should clearly answer: What is known? What is unknown? Why this study matters specifically?
Reply: We have restructured the final paragraph of the Introduction to explicitly answer these questions. It now clearly states (i) known: mangrove extracts show anticancer activity but their molecular targets are unexplored; (ii) unknown: the potential of specific R. mucronata compounds to inhibit Keap1 and YEATS2; (iii) importance: identifying computational leads can accelerate the discovery of novel NSCLC modulators.

Q16. The choice of 1X2J (Keap1), 5XNV (YEATS2) is actually a strong point, but buried.
Reply: We have moved the rationale for target selection to a prominent, dedicated paragraph in the Introduction and expanded it to highlight their critical roles in NSCLC chemoresistance

(Keap1–Nrf2) and epigenetic transcriptional reprogramming (YEATS2). This strategic choice is now clearly communicated.

Q17. The methodology employs a comprehensive computational workflow… However, the section suffers from substantial issues in clarity, reproducibility, and scientific precision. Key methodological details are missing… A critical concern is the continued misclassification of the selected targets as proteases. Additionally, the ADMET section contains erroneous references to “fungicide” properties.
Reply: We have thoroughly revised the Methodology. The misclassification has been corrected. We removed all inappropriate terminology such as “fungicide” (replaced with “anticancer agent” or “inhibitor”). We have also added missing details: grid box parameters (Table 2), docking validation by redocking, and a clear description of the ligand selection rationale (Section 2.1). The ADMET section no longer contains any reference to fungicide.

Q18. The Results and Discussion section… suffers from significant issues in clarity, consistency, and scientific interpretation. A critical concern is the persistent misclassification of targets… Notable inconsistencies in reported results across docking platforms, and criteria for lead compound selection are not clearly justified. Several claims overinterpret computational findings.
Reply: We have comprehensively revised the Results and Discussion. The misclassification has been eradicated. Inconsistencies between PyRx and Glide scores are now discussed as expected variations due to different scoring functions, and we present both sets of results transparently. A new subsection “Benchmarking with Known Inhibitors” (3.2.6) has been added to contextualize our docking scores relative to known Keap1 and YEATS2 inhibitors, showing that our compounds are moderate-affinity initial hits. The selection criteria for L10 and L02 are clearly justified based on combined DFT, docking (especially Glide hydrogen bond networks), MD stability, and acceptable ADMET profiles. All overinterpretations have been removed, and conclusions are now appropriately cautious.

Q19. The section also contains inappropriate terminology (e.g., references to “fungicide” activity) and non-scientific narrative elements.
Reply: All such language has been excised. The revised manuscript uses precise scientific terminology.

Reviewer #3 (Major Comments)

Comment 1. Wrong classification: the study focuses on Keap1 (PDB: 1X2J) and YEATS2 (PDB: 5XNV). Both are not proteases. This mischaracterization should be corrected, and the rationale for selecting these targets should be expanded.
Reply: We have completely corrected this error. The title now reads *"…Lung Cancer Protein Inhibitors"*. Every occurrence of "protease" throughout the manuscript has been replaced with "protein". We have expanded the Introduction (pages 2–3) to clearly explain the biological roles of Keap1 in the Keap1–Nrf2 pathway (oxidative stress, chemoresistance) and YEATS2 in epigenetic regulation (H3K27ac reader), and why they are mechanistically relevant targets in NSCLC.

Comment 2. The interpretation of docking results is problematic… binding affinities in the range of −5.4 to −7.8 kcal/mol are moderate and not contextualized against known inhibitors. Unsupported claims of therapeutic superiority.
Reply: We have substantially tempered all claims. A new subsection "Benchmarking with Known Inhibitors" (3.2.6) has been added, which compares our docking scores with literature values for known Keap1 inhibitors (GlideScores < −9.0 kcal/mol for optimized inhibitors) and YEATS2 inhibitors. We now clearly state that our compounds exhibit moderate binding affinities typical of initial natural product screening hits and are generally weaker than many optimized synthetic inhibitors. All suggestions of superiority over existing chemotherapeutics have been removed.

Comment 3. The reliance on DFT-derived descriptors is similarly overstated… assertion that a lower HOMO–LUMO gap implies superior therapeutic potential reflects an oversimplification.
Reply: We have revised the interpretation in Sections 3.1.2 and 3.1.3. The text now includes an explicit caution: *"However, these DFT-derived parameters provide only supportive insights into the intrinsic electronic characteristics of the compounds. They are not validated predictors of biological or anticancer activity, and a smaller HOMO–LUMO gap should not be interpreted as implying superior therapeutic potential."*

Comment 4. MD analysis reports RMSD stability below 3 Å, but lacks deeper interrogation (e.g., MM-PBSA, residue-level mapping, comparison with apo dynamics).
Reply: We acknowledge this limitation. In Section 3.3.1, we now explicitly state that RMSD alone is insufficient to confirm binding strength and that further analyses such as MM-PBSA/MM-GBSA, detailed per-residue energy decomposition, and comparison with apo-protein simulations would be required for deeper mechanistic insight. The MD results are presented as preliminary supportive evidence of complex stability.

Comment 5. ADMET analysis overinterprets results, concluding high oral bioavailability and negligible toxicity, and suggesting superiority over existing chemotherapeutics.
Reply: All such statements have been removed or heavily moderated. We now describe the ADMET profiles as "acceptable predicted properties suitable for early-stage screening". We no longer compare our compounds directly to approved drugs or claim negligible toxicity.

Comment 6. A broader limitation is the lack of integration with lung cancer biology… minimal discussion of how inhibition would influence tumor progression, treatment response, or resistance mechanisms.
Reply: We have significantly expanded the biological context in the Introduction and partially in the Conclusion. The Introduction now includes dedicated paragraphs detailing how Keap1 dysregulation drives Nrf2-mediated chemoresistance and how YEATS2 overexpression sustains oncogenic transcription in NSCLC. The revised conclusion briefly mentions the potential of modulating these pathways to restore redox balance and suppress epigenetic-driven proliferation.

Comment 7. The manuscript does not provide a clear pathway toward experimental validation.
Reply: We have added a clear and specific experimental validation framework in the Conclusion. This includes: (1) cytotoxicity and anti-proliferative assays in NSCLC cell lines (A549, H1975) alongside normal lung epithelial cells for selectivity; (2) target engagement studies (Western blotting for Nrf2 targets, H3K27ac ChIP-qPCR); (3) assessment of apoptosis and cell cycle arrest; and (4) synergy studies with standard chemotherapeutics.

Comment 8. Methodological and Reporting Concerns: ligand selection lacks clear rationale; docking methodology insufficiently detailed; data availability statement inadequate.
Reply:

- **Ligand selection rationale:** A detailed paragraph has been added to Section 2.1 explaining that the 14 compounds were systematically chosen to cover diverse chemical classes (nitrogen heterocycles, polyethers, long-chain esters, polyols, steroids, etc.) based on reported secondary metabolites from R. mucronata, ensuring a broad range of physicochemical properties.
- **Docking methodology details:** Grid box parameters are now fully provided in Table 2. We have also stated that docking protocols were validated by redocking the co-crystallized ligands, achieving RMSD < 2.0 Å.
- **Data Availability:** The statement has been updated to indicate that all input files (optimized ligand structures, protein structures, docking grids, MD input parameters) and key output data (docking poses, MD trajectories, prediction outputs) are available from the corresponding author upon reasonable request.

Comment 9. Conclusion and Recommendation: conceptual inaccuracies, insufficient methodological transparency, and substantial overinterpretation. Major revision required.
Reply: We have fully addressed all conceptual inaccuracies (target misclassification corrected), enhanced methodological transparency (detailed grid boxes, docking validation, ligand selection), and eliminated overinterpretation. The revised manuscript presents the computational findings with appropriate caution, clearly stating that the identified compounds are initial hits requiring experimental validation.

We believe that the extensive revisions we have made have substantially improved the manuscript's scientific accuracy, clarity, and rigor. We are grateful for the reviewers' time and expertise, which have been instrumental in strengthening this work. We hope that the revised manuscript is now suitable for publication.

Sincerely,
Ajoy Kumer
