# Supplementary material for: Investigating the anticancer potential of phytochemicals from the mangrove Rhizophora mucronata as inhibitors of lung cancer-associated proteins: Insights from multiscale computational and in silico analyses
Source: PLoS One. 2026 Oct 7;21(10):e0359525. doi: 10.1371/journal.pone.0359525 (PMC13645060; doi:10.1371/journal.pone.0359525)
Supplement: Supplementary file 4 [file pone.0359525.s001.docx]

|  |  |  |
| --- | --- | --- |
| 4-chloro-3-methoxy-2-methylpyridine (L01) | benzo[h]quinoline-4-carboxylic acid (L02) | 2,2,4,4,6,6,8,8,10,10,12,12,14,14-tetradecamethyl-1,3,5,7,9,11,13-heptaoxacyclotetradecane (L03) |
|  |  |  |
| Methyl 14-(2-octylcyclopropyl)tetradecanoate (L04) | N-(3-fluorobenzyl)-7H-purin-6-amine (L05) | Methyl 14-methylpentadecanoate (L06) |
|  |  |  |
| (E)-3,4,5-trihydroxy-6-(hydroxymethyl)tetrahydro-2H-pyran-2-yl N-hydroxybut-3-enimidothioate (L07) | (2R,3S,4R,5R)-2,4,5,6-tetrahydroxy-3-methoxyhexanal (L08) | Octadeca-9,12-dienoic acid (L09) |
|  |  |  |
| Cyclohexane-1,2,3,4,5,6-hexaol (L10) | ((8S,9S,10S,13R,14S,17R)-13-methyl-17-((R)-6-methylheptan-2-yl)-2,3,4,7,8,9,10,11,12,13,14,15,16,17-tetradecahydro-1H-cyclopenta[a]phenanthren-10-yl)methanol (L011) | (E)-5,6-dimethoxy-3,4-dihydronaphthalen-1(2H)-one oxime (L012) |
|  |  | |
| 2,2,4,4,6,6,8,8,10-nonamethyl-3,5,7,9-tetraoxaundecane (L13) | 1,4,5,8-tetraphenyl-1H-thiopyrano[4,3-c]pyridine (L014) | |

**Figure S1. Optimized structure of tested compounds**
