# Supplementary material for: Investigating the anticancer potential of phytochemicals from the mangrove Rhizophora mucronata as inhibitors of lung cancer-associated proteins: Insights from multiscale computational and in silico analyses
Source: PLoS One. 2026 Oct 7;21(10):e0359525. doi: 10.1371/journal.pone.0359525 (PMC13645060; doi:10.1371/journal.pone.0359525)
Supplement: Supplementary file 5 [file pone.0359525.s002.docx]

|  | HOMO | LUMO |
| --- | --- | --- |
| L01 |  |  |
| L02 |  |  |
| L03 |  |  |
| L04 |  |  |
| L05 |  |  |
| L06 |  |  |
| L07 |  |  |
| L08 |  |  |
| L09 |  |  |
| L10 |  |  |
| L11 |  |  |
| L12 |  |  |
| L13 |  |  |
| L14 |  |  |

**Figure S2. Frontier Molecular orbital: HOMO LUMO**
