# Supplementary material for: Investigating the anticancer potential of phytochemicals from the mangrove Rhizophora mucronata as inhibitors of lung cancer-associated proteins: Insights from multiscale computational and in silico analyses
Source: PLoS One. 2026 Oct 7;21(10):e0359525. doi: 10.1371/journal.pone.0359525 (PMC13645060; doi:10.1371/journal.pone.0359525)
Supplement: Supplementary file 7 [file pone.0359525.s004.docx]

**Table S1.** Ligands Interaction with amino acid residues and their bond distance (PDB ID 1x2j)

| Ligands Interaction with amino acid residues and their bond distance (PDB ID 1x2j) | | | | | |
| --- | --- | --- | --- | --- | --- |
| Active sites |  | | | | |
| Ligand No | Hydrogen bond | | Hydrophobic bond | | Van der Waals bond |
|  | The interacting residue of amino acid | Distance, A֯ | The interacting residue of amino acid | Distance, A֯ |  |
| L01 | ALA A:510 | 2.31 | LEU A:557  VAL A:604  LEU A:365 | 3.76  3.62  3.42 | Absent |
| L02 | VAL A:463  GLY A:603 | 2.38  2.76 | ALA A:366  ALA A:366 | 3.86  4.46 | Absent |
| L03 | 0 | 0 | 0 | 0 | VAL A:324  VAL A:561  THR A:560  VAL A:369  CYS A:368  VAL A:608  ALA A:607  VAL A:606  GLY A: 367  VAL A:420  VAL A:512  CYS A:513  ALA A:466  VAL A:467  GLY A:419  VAL A:418  VAL A:514 |
| L04 | GLY A:367  ILE A:559 | 2.16  2.41 | VAL A:369  VAL A:420  VAL A:561  VAL A:514 | 4.75  3.97  3.99  4.04 | Absent |
| L05 | LEU A:365  ILE A:416  VAL A:463 | 2.27  2.09  2.56 | VAL A:604  ALA A:366  GLY A:367  VAL A:418  ALA A:510 | 3.43  3.64  3.07  3.08  3.36 | Absent |
| L06 | VAL A:465 | 2.85 | ALA A:510  GLY A:417  GLY A:464  VAL A:420  VAL A:420  VAL A:369  VAL A:369  ALA A:607 | 3.55  3.62  3.64  5.30  4.35  4.66  4.96  607 | Absent |
| L07 | LEU A:365  ILE A:416  VAL A:463 | 1.95  2.34  2.25 | GLY A:605  LEU A:557  LEU A:557  ILE A:416 | 3.58  3.50  3.75  3.67 | Absent |
| L08 | VAL A:604  LEU A:365  ILE A:416  ILE A:416  ILE A:416 | 2.58  2.39  2.51  1.97  1.94 | GLY A:558  LEU A:557 | 3.73  3.70 | Absent |
| L09 | ILE A:559 | 1.94 | VAL A:420 | 5.10 | Absent |
| L10 | GLY A:367  ILE A:559  VAL A:465  VAL A:465  VAL A:463  ILE A:416 | 2.97  1.84  2.18  1.74  2.63  2.70 | GLY A:558 | 3.46 | Absent |
| L11 | VAL A:512 | 2.74 | VAL A:418  ALA A:366  ALA A:607  VAL A:561  VAL A:561  VAL A:514  VAL A:514  VAL A:608  VAL A:369  VAL A:369  VAL A:420 | 5.26  4.08  4.60  4.01  3.95  3.87  3.91  4.09  4.17  4.98  4.94 | Absent |
| L12 | VAL A:606  VAL A:606 | 2.56  2.23 | ALA A:510  LEU A:557  VAL A:604  ALA A:366  ALA A:366 | 3.41  3.41  3.45  3.70  4.91 | Absent |
| L13 |  |  | ALA A:366 | 3.37 | GLY A:558  LEU A:557  VAL A:604  GLY A:417  ILE A:416  LEU A:365 |
| L14 |  |  | VAL A:420  VAL A:608  VAL A:369  VAL A:324  VAL A:324  VAL A:514  VAL A:514  VAL A:561  VAL A:561 | 3.80  4.97  3.48  2.91  3.05  3.52  4.53  5.29  4.41 |  |
