# Supplementary material for: Investigating the anticancer potential of phytochemicals from the mangrove Rhizophora mucronata as inhibitors of lung cancer-associated proteins: Insights from multiscale computational and in silico analyses
Source: PLoS One. 2026 Oct 7;21(10):e0359525. doi: 10.1371/journal.pone.0359525 (PMC13645060; doi:10.1371/journal.pone.0359525)
Supplement: Supplementary file 8 [file pone.0359525.s005.docx]

**Table S2.** Ligands Interaction with amino acid residues and their bond distance (PDB ID 5xnv)

| Ligands Interaction with amino acid residues and their bond distance (PDB ID 5xnv) | | | | | | |
| --- | --- | --- | --- | --- | --- | --- |
| Active sites | |  | | | | |
| Ligand No | Hydrogen bond | | | Hydrophobic bond | | Van der Waals bond |
|  | The interacting residue of amino acid | | Distance, A֯ | The interacting residue of amino acid | Distance, A֯ |  |
| L01 | VAL A:238  ILE A:247 | | 2.82  2.79 | HIS A:276  ARG A:239  PHE A:175 | 3.39  4.43  3.63 | Absent |
| L02 | PRO A:274 | | 2.98 | HIS A:276  VAL A:270  VAL A:270 | 4.20  3.54  4.21 | Present |
| L03 | SER A:230  ASN A:265 | | 2.94  2.89 | 0 | 0 | GLY A:281  ASP A:228  GLN A:229  TRP A:282  ARG A: 280  ARG A:279  TYR A:262  LYS A:263  PRO A:264 |
| L04 |  | |  | PHE A:257 | 5.30 | Absent |
| L05 | ARG A:300  LYS A:299  GLU A:328  ASN A:298 | | 1.94  2.49  2.13  2.51 | PHE A:205 | 4.84 | Absent |
| L06 |  | |  | SER A:261 | 2.71 | Absent |
| L07 | LYS A:299  PHE A:293 | | 2.08  2.79 | LYS A:299  HIS A:292 | 3.74  4.57 | Absent |
| L08 | PHE A:275  ARG A:239 | | 2.29  4.75 | ARG A:239 | 1.17 | Absent |
| L09 | PHE A:257  LEU A:258 | | 2.80  1.93 | PRO A:260 | 3.56 | Absent |
| L10 | LYS A:252  PHE A:293  ASN A:298 | | 2.07  2.30  2.22 | PHE A:293  SER A:296 | 1.44  3.43 | Absent |
| L11 | ASP A:266 | | 3.06 | PHE A:257  PHE A:257  PHE A:257  LEU A:267  ARG A:288  PRO A:260  ILE A:304  ILE A:304  PRO A:286  PRO A:286 | 5.14  4.69  4.47  4.27  3.92  4.27  4.19  3.90  4.55  4.65 | Absent |
| L12 | VAL A:238  HIS A:276 | | 2.31  2.82 | PRO A:273  ILE A:247  SER A:246  ARG A:239 | 4.99  5.15  3.59  4.92 | Absent |
| L13 | TRY A: 262 | | 3.82 | 0 | 0 | Absent |
| L14 |  | |  | VAL A:325  ILE A:301  ILE A:301  ILE A:301  ASP A:326  GLU A:328  VAL A:327 | 4.42  5.41  5.47  3.43  3.39  4.84  5.16 | Absent |
