# Supplementary material for: Investigating the anticancer potential of phytochemicals from the mangrove Rhizophora mucronata as inhibitors of lung cancer-associated proteins: Insights from multiscale computational and in silico analyses
Source: PLoS One. 2026 Oct 7;21(10):e0359525. doi: 10.1371/journal.pone.0359525 (PMC13645060; doi:10.1371/journal.pone.0359525)
Supplement: Supplementary file 10006 [file pone.0359525.s006.pdf]

PLOS One

Investigating the anti-cancer properties of phytochemicals from mangrove tree, Rizhophora muarnata, as Lung Cancer Protease Inhibitors: Insights from Multi-scale Computational and In silico Analysis

--Manuscript Draft--

**Manuscript Number:**	PONE-D-26-14113
**Article Type:**	Research Article
**Full Title:**	Investigating the anti-cancer properties of phytochemicals from mangrove tree, Rizhophora muarnata, as Lung Cancer Protease Inhibitors: Insights from Multi-scale Computational and In silico Analysis
**Short Title:**	Multi-scale Computational and In silico Analysis Study for Lung Cancer Protease Inhibitors
**Corresponding Author:**	Ajoy Kumer, MPhil<br>IUBAT: International University of Business Agriculture and Technology<br>Naogaon, Naogaon zila BANGLADESH
**Keywords:**	DFT; Docking; Molecular dynamic; ADMET; Binding affinity; Rizhophora muarnata; Lung cancer

Abstract:

Graphically presentation of abstract

Lung cancer is still the most common cause of cancer deaths around the world. Even though surgery, chemotherapy, and targeted therapies have gotten better, the five-year survival rate is only about 20%. To improve patient outcomes, need new protease inhibitors that are low in toxicity and can block important oncogenic pathways. Mangrove ecosystems are known to be rich in bioactive natural products that have been shown to Figureht cancer. However, the specific protease-inhibitory potential of compounds from Rhizophora mucronata against lung cancer proteins has never been systematically studied using an integrated multi-scale computational pipeline. This is a critical opportunity that has not yet been taken advantage of in the search for new natural-product drugs. This study sought to computationally identify and characterize protease inhibitors derived from Rhizophora mucronata as effective anti-lung cancer agents by assessing their electronic properties, binding affinities, pharmacokinetic profiles, and dynamic stability against established lung cancer targets. Optimized fourteen ligands and used density functional theory (DFT) calculations (B3LYP/6-31G*) to find the HOMO, LUMO, energy gaps, chemical potential, hardness, softness, and electrophilicity indices. Molecular docking on lung cancer-related proteins PDB 1X2J and 5XNV using both PyRx/AutoDock and Schrödinger Glide. SwissADME and pkCSM were used to check the drug-likeness and ADMET properties of the top-ranked complexes. Then, 100-ns molecular dynamics simulations were run in Desmond under NPT conditions to check the RMSD, RMSF, and interaction persistence. Ligand L10 had the smallest HOMO–LUMO gap (0.36 eV), which means it was the most reactive. It also had the strongest binding affinities on both docking platforms (–7.789 kcal/mol for 1X2J and –5.482 kcal/mol for 5XNV via Glide), thanks to eight hydrogen bonds and many hydrophobic contacts. Ligands L02, L05, L07, and L11 also had strong affinities. MD simulations showed that the L10–1X2J and L02–5XNV complexes were very stable (RMSD always <3 Å), and ADMET profiling showed that these leads had good oral bioavailability, followed Lipinski rules, and were not very toxic. The complementary electronic profiles of L10's high reactivity and L05's exceptional stability make it possible to effectively block the active site of lung cancer proteases through balanced charge transfer and non-covalent interactions. These traits, along with good pharmacokinetics, make mangrove-derived scaffolds a good alternative to current chemotherapeutics by getting around some of their biggest problems. In short, here are the main points and what they mean: This multi-scale computational framework finds L10 to be the best lead compound from Rhizophora mucronata for treating lung cancer. The study sets up a strong, low-cost pipeline for speeding up the discovery of drugs based on mangroves. It also lays the groundwork for future wet-lab validation, preclinical development, and ultimately new, nature-inspired drugs that could significantly improve the survival and quality of life for lung cancer patients.

Powered by Editorial Manager® and ProduXion Manager® from Aries Systems Corporation

<table>
<tr><td rowspan="11">Order of Authors:</td><td>Zena Abdul-Ameer Mahdi</td></tr>
<tr><td>Batool Shakir Abed Almjalaw</td></tr>
<tr><td>Mollah Naimuzzaman</td></tr>
<tr><td>Md Mahabub Hasan</td></tr>
<tr><td>Ajoy Kumer, MPhil</td></tr>
<tr><td>Farah Tareq Al-Alaq</td></tr>
<tr><td>Hussein O.M. Al-Dahmoshi</td></tr>
<tr><td>Faizur Rahman</td></tr>
<tr><td>Md. Fayez Al-Hakim Khan</td></tr>
<tr><td>Noor S.K. Al-Khafaji</td></tr>
<tr><td>Sarah Alwan Malik Al-Yasiri</td></tr>
<tr><td>Additional Information:</td><td></td></tr>
<tr><td>Question</td><td>Response</td></tr>
<tr><td>Financial Disclosure

Enter a financial disclosure statement that describes the sources of funding for the work included in this submission. Review the submission guidelines for detailed requirements. View published research articles from PLOS ONE for specific examples.

This statement is required for submission and will appear in the published article if the submission is accepted. Please make sure it is accurate.

Funded studies
Enter a statement with the following details:
• Initials of the authors who received each award
• Grant numbers awarded to each author
• The full name of each funder
• URL of each funder website
• Did the sponsors or funders play any role in the study design, data collection and analysis, decision to publish, or preparation of the manuscript?

Did you receive funding for this work?</td><td>The author(s) received no specific funding for this work.</td></tr>
<tr><td>Competing Interests

Use the instructions below to enter a</td><td>NO authors have competing interests
Enter: The authors have declared that no competing interests exist.</td></tr>
</table>

competing interest statement for this submission. On behalf of all authors, disclose any competing interests that could be perceived to bias this work—acknowledging all financial support and any other relevant financial or non-financial competing interests.<br><br>This statement is required for submission and **will appear in the published article** if the submission is accepted. Please make sure it is accurate and that any funding sources listed in your Funding Information later in the submission form are also declared in your Financial Disclosure statement.<br><br>View published research articles from *PLOS ONE* for specific examples.<br><br>**NO authors have competing interests**<br><br>Enter: *The authors have declared that no competing interests exist.*<br><br>**Authors with competing interests**<br><br>Enter competing interest details beginning with this statement:<br><br>*I have read the journal's policy and the authors of this manuscript have the following competing interests: [insert competing interests here]*<br><br>* typeset	
**Ethics Statement**<br><br>Enter an ethics statement for this submission. This statement is required if the study involved:<br><br>• Human participants<br>• Human specimens or tissue<br>• Vertebrate animals or cephalopods<br>• Vertebrate embryos or tissues<br>• Field research	N/A

Write "N/A" if the submission does not require an ethics statement.

General guidance is provided below. Consult the submission guidelines for detailed instructions. **Make sure that all information entered here is included in the Methods section of the manuscript.**

Format for specific study types

Human Subject Research (involving human participants and/or tissue)

- Give the name of the institutional review board or ethics committee that approved the study
- Include the approval number and/or a statement indicating approval of this research
- Indicate the form of consent obtained (written/oral) or the reason that consent was not obtained (e.g. the data were analyzed anonymously)

Animal Research (involving vertebrate animals, embryos or tissues)

- Provide the name of the Institutional Animal Care and Use Committee (IACUC) or other relevant ethics board that reviewed the study protocol, and indicate whether they approved this research or granted a formal waiver of ethical approval
- Include an approval number if one was obtained
- If the study involved *non-human primates*, add *additional details* about animal welfare and steps taken to ameliorate suffering
- If anesthesia, euthanasia, or any kind of animal sacrifice is part of the study, include briefly which substances and/or methods were applied

Field Research

Include the following details if this study involves the collection of plant, animal, or other materials from a natural setting:

- Field permit number
- Name of the institution or relevant body that granted permission

Powered by Editorial Manager® and ProduXion Manager® from Aries Systems Corporation

**Data Availability**<br><br>Authors are required to make all data underlying the findings described fully available, without restriction, and from the time of publication. PLOS allows rare exceptions to address legal and ethical concerns. See the PLOS Data Policy and FAQ for detailed information.<br><br>A Data Availability Statement describing where the data can be found is required at submission. Your answers to this question constitute the Data Availability Statement and **will be published in the article**, if accepted.<br><br>**Important:** Stating 'data available on request from the author' is not sufficient. If your data are only available upon request, select 'No' for the first question and explain your exceptional situation in the text box.<br><br>Do the authors confirm that all data underlying the findings described in their manuscript are fully available without restriction?	Yes - all data are fully available without restriction
**Describe where the data may be found in full sentences. If you are copying our sample text, replace any instances of XXX with the appropriate details.**<br><br>• If the data are **held or will be held in a public repository**, include URLs, accession numbers or DOIs. If this information will only be available after acceptance, indicate this by ticking the box below. For example: *All XXX files are available from the XXX database (accession number(s) XXX, XXX.*).<br>• If the data are all contained **within the manuscript and/or Supporting Information files**, enter the following: *All relevant data are within the manuscript and its Supporting Information files.*<br>• If neither of these applies but you are able to provide **details of access elsewhere**, with or without limitations, please do so. For example:	All relevant data are within the manuscript and its Supporting Information files.

Data cannot be shared publicly because of [XXX]. Data are available from the XXX Institutional Data Access / Ethics Committee (contact via XXX) for researchers who meet the criteria for access to confidential data.<br><br>*The data underlying the results presented in the study are available from (include the name of the third party and contact information or URL).*<br>• This text is appropriate if the data are owned by a third party and authors do not have permission to share the data.<br><br>* typeset	
Additional data availability information:	Tick here if your circumstances are not covered by the questions above and you need the journal's help to make your data available.

Manuscript

Click here to access/download;Manuscript;Main manuscript_Revised 2026.docx

Investigating the anti-cancer properties of phytochemicals from mangrove tree, *Rizhophora muarnata*, as Lung Cancer Protease Inhibitors: Insights from Multi-scale Computational and In silico Analysis

Zena Abdul-Ameer Mahdi[1], Batool Shakir Abed Almjalaw[2], Mollah Naimuzzaman[3], Md Mahabub Hasan[4], Ajoy Kumer*[5,6], Farah Tareq Al-Alaq[7], Hussein O.M. Al-Dahmoshi[8], Faizur Rahman[4], Md. Fayez Al-Hakim Khan[4], Noor S.K. Al-Khafaji[9], and Sarah Alwan Malik Al-Yasiri[10]

[1]Department of medical physics, college of Applied Medical Sciences, University of Kerbala, Karbala 56001, Iraq., email: Zena.a.mahdy@uokerbala.edu.iq

[2] Ministry of Education, General Directorate of Education in Kerbala. Kerbala, Iraq Email: batool_shakir@karbala.edu.iq

[3]Department of Agroforestry and Environmental Science, Sylhet Agricultural University, Sylhet-3100, Bangladesh; Email: mollahnaimuzzaman@gmail.com

[4]College of Agricultural Sciences, IUBAT—International University of Business Agriculture and Technology, 4 Embankment Drive Road, Sector 10 Uttara Model Town, Dhaka 1230, Bangladesh. Email: mahabubhasan33445@gmail.com; FR: faizurrahmanzidni80@gmail.com FHK: fayezalhakim117@gmail.com

[5]Department of General Medicine, Saveetha Institute of Medical and Technical Sciences in Saveetha Medical College and Hospital, Chennai, India, Email: kumarajoy.cu@gmail.com

[6]College of Arts and Sciences, Department of Chemistry, IUBAT—International University of Business Agriculture and Technology, 4-Embankment Drive Road, Sector 10 Uttara Model Town, Dhaka 1230, Bangladesh. Email: kumarajoy.cu@gmail.com

[7]Department of Biology, college of science, University of Babylon, Hilla 51001, Babylon, Iraq, Email: sci.farah.tariq@uobabylon.edu.iq

[8]Department of Biology, college of science, University of Babylon, Hilla 51001, Babylon, Iraq, Email: sci.hussein.oleiwi@uobabylon.edu.iq

[9]Department of Biology, college of science, University of Babylon, Hilla 51001, Babylon, Iraq, Email: sci.noor.salman@uobabylon.edu.iq

[10]Department of environmental health, College of Applied Medical Sciences, University of Kerbala, Karbala 56001, Iraq., email: alyasirsara@gmail.com

Corresponding Authors

Email: kumarajoy.cu@gmail.com

Abstract:

Graphically presentation of abstract:

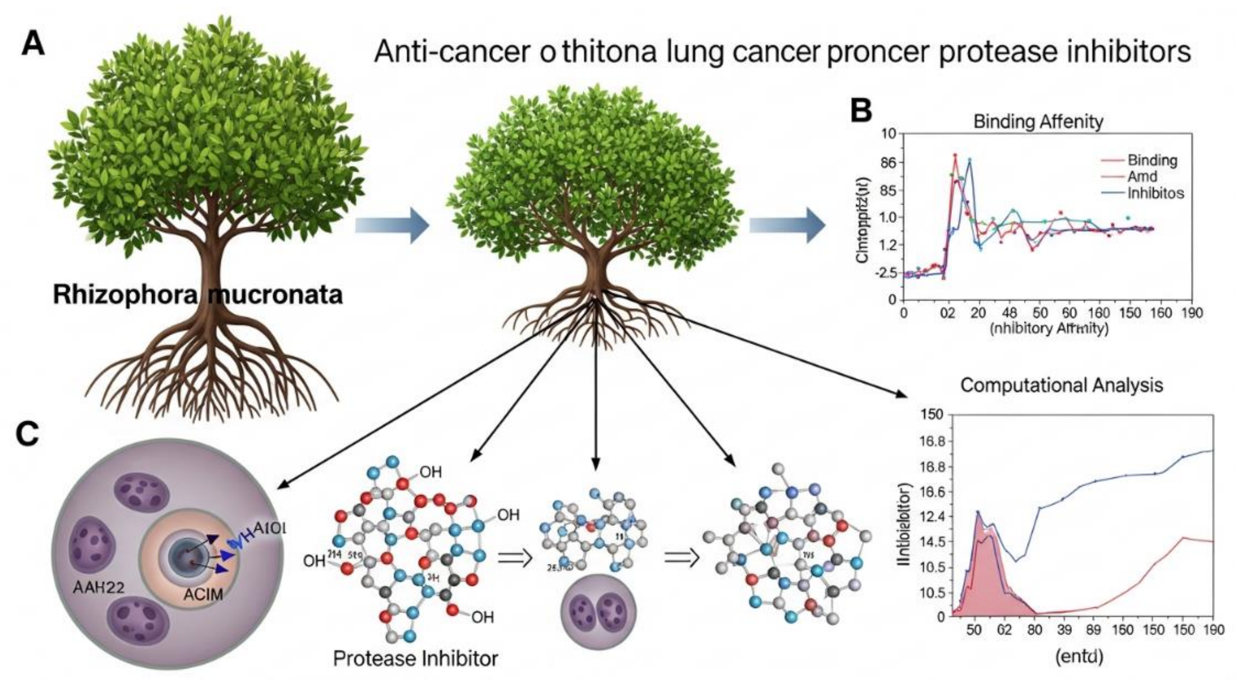

Lung cancer is still the most common cause of cancer deaths around the world. Even though surgery, chemotherapy, and targeted therapies have gotten better, the five-year survival rate is only about 20%. To improve patient outcomes, need new protease inhibitors that are low in toxicity and can block important oncogenic pathways. Mangrove ecosystems are known to be rich in bioactive natural products that have been shown to lung cancer. However, the specific protease-inhibitory potential of compounds from Rhizophora mucronata against lung cancer proteins has never been systematically studied using an integrated multi-scale computational pipeline. This is a critical opportunity that has not yet been taken advantage of in the search for new natural-product drugs. This study sought to computationally identify and characterize protease inhibitors derived from Rhizophora mucronata as effective anti-lung cancer agents by assessing their electronic properties, binding affinities, pharmacokinetic profiles, and dynamic stability against established lung cancer targets. Optimized fourteen ligands and used density functional theory (DFT) calculations (B3LYP/6-31G*) to find the HOMO, LUMO, energy gaps, chemical potential, hardness, softness, and electrophilicity indices. Molecular docking on lung cancer-related proteins PDB 1X2J and

5XNV using both PyRx/AutoDock and Schrödinger Glide. SwissADME and pkCSM were used to check the drug-likeness and ADMET properties of the top-ranked complexes. Then, 100-ns molecular dynamics simulations were run in Desmond under NPT conditions to check the RMSD, RMSF, and interaction persistence. Ligand L10 had the smallest HOMO–LUMO gap (0.36 eV), which means it was the most reactive. It also had the strongest binding affinities on both docking platforms (–7.789 kcal/mol for 1X2J and –5.482 kcal/mol for 5XNV via Glide), thanks to eight hydrogen bonds and many hydrophobic contacts. Ligands L02, L05, L07, and L11 also had strong affinities. MD simulations showed that the L10–1X2J and L02–5XNV complexes were very stable (RMSD always <3 Å), and ADMET profiling showed that these leads had good oral bioavailability, followed Lipinski rules, and were not very toxic. The complementary electronic profiles of L10's high reactivity and L05's exceptional stability make it possible to effectively block the active site of lung cancer proteases through balanced charge transfer and non-covalent interactions. These traits, along with good pharmacokinetics, make mangrove-derived scaffolds a good alternative to current chemotherapeutics by getting around some of their biggest problems. In short, here are the main points and what they mean: This multi-scale computational framework finds L10 to be the best lead compound from Rhizophora mucronata for treating lung cancer. The study sets up a strong, low-cost pipeline for speeding up the discovery of drugs based on mangroves. It also lays the groundwork for future wet-lab validation, preclinical development, and ultimately new, nature-inspired drugs that could significantly improve the survival and quality of life for lung cancer patients.

Keywords: DFT, Docking, Molecular dynamic, ADMET, Binding affinity, *Rizhophora muarnata,* Lung cancer.

1. Introduction

Lung cancer is the top cause of cancer deaths worldwide, killing around 1.8 million people each year and contributing nearly one-fifth of all cancer-related fatalities[1]. It ranks as the most frequently diagnosed cancer in men and the second most common in women, with an estimated 2.2 million new cases and 1.8 million deaths reported globally in 2020, representing about 18% of

total cancer mortality[2]. In 2022, China, the US and Japan had the highest number of deaths from lung cancer, which Age-standardised rate (ASR) lets us compare cancer risk between countries by removing age effects. A country with lower ASR can still have more cases if its population is larger [3]. In 2022–2024 data, lung cancer remains the top cause of cancer death globally, with about 2.5 million new cases and 1.8 million deaths each year [3]. Despite substantial progress in targeted therapies and immunotherapy, the 5-year relative survival rate hovers around 25% for all stages combined, underscoring an urgent need for novel, low-toxicity therapeutic agents that can effectively disrupt oncogenic pathways while minimizing systemic adverse effects [4]. Many powerful and often preventable factors drive lung cancer risk today [5, 6]. Tobacco smoking is still the main cause, and in some countries around 80% of lung cancer deaths are directly due to smoking [7, 8]. Second-hand smoke also harms non-smokers, especially in homes and workplaces [9]. Outdoor air pollution, especially fine particles (PM2.5), now raises lung cancer risk even in people who have never smoked [10]. Indoor air pollution from solid fuels such as coal or biomass and from cooking fumes is a major risk in many low- and middle-income countries [11, 12]. Other important factors include radon, asbestos, certain workplace chemicals, and family history [12, 13].

Lung cancer often grows silently for years and is usually found late, when cure is hard. It can block airways, destroy lung tissue, spread to organs like brain, liver, and bone, and cause organ failure [14]. Even with treatment, only about 1 in 4–5 patients in high-income countries live five years after diagnosis [15, 16]. Approved lung cancer treatment includes surgery, radiation, chemotherapy, targeted drugs, and immunotherapy. These help many patients but still face big limits: most cases are found late, side effects can be severe, tumors often become resistant or come back, and modern targeted or immune drugs are very costly and only work for patients whose tumors have certain mutations or markers [17, 18] . Because current lung cancer treatments still have serious limits, researchers keep searching for new and better therapies, and push scientists to develop more effective, safer, and more affordable options for patients.

Natural products derived phytochemicals, low cost and affordable medicine, from terrestrial and marine ecosystems have historically supplied a substantial proportion of approved anticancer drugs, offering structurally diverse scaffolds with favorable safety profiles compared with synthetic chemotherapeutics [19]. Among these, mangrove ecosystems represent underexplored

reservoirs of bioactive secondary metabolites, including flavonoids, tannins, and triterpenoids, many of which exhibit potent cytotoxic, antioxidant, and anti-inflammatory activities [20]. Lung cancer is broadly classified into small-cell lung cancer (SCLC, ~15%) and non-small-cell lung cancer (NSCLC, ~85%), the latter being the predominant histological subtype [21]. NSCLC is characterized by late-stage diagnosis, aggressive metastatic potential, and frequent dysregulation of oxidative stress and epigenetic regulatory pathways, resulting in poor prognosis even with current standard-of-care treatments [22]. Existing therapies are frequently limited by acquired resistance, severe off-target toxicities, and modest improvements in overall survival, particularly in advanced or recurrent disease [23]. Consequently, there is a pressing requirement for innovative inhibitors capable of selectively targeting key proteins driving lung cancer progression while satisfying drug-like physicochemical and pharmacokinetic criteria. Although mangrove-derived extracts have demonstrated anticancer activity against several cell lines, including lung carcinoma models [20, 24], no systematic multi-scale investigation has yet explored the potential of protease or pathway-specific inhibitors of phytochemical from *Rhizophora mucronata* against validated lung cancer protein targets.

This study recovers a critical knowledge gap, initially screening the phytochemical from *Rhizophora mucronata* against validated lung cancer by various computational approaches (density functional theory, molecular docking, and molecular dynamics) that can rapidly identify lead candidates with high binding affinity, favorable ADMET properties and structural stability prior to resource-intensive experimental validation. *Rhizophora mucronata*, a widely distributed mangrove species in tropical and subtropical intertidal zones, has been traditionally utilized in folk medicine for its anti-inflammatory, antimicrobial and antioxidant properties [25]. In addition, its rich phytochemical profile particularly polyphenols, and alkaloids confers exceptional environmental resilience and translates into broad pharmacological potential, making it an ideal candidate for targeted anticancer drug discovery [26, 27]. Multiple independent studies have confirmed the cytotoxic efficacy of *R. mucronata* extracts against lung cancer cell lines [28], with methanol stem extracts outperforming leaf extracts in reducing viability [20]. Isolated compounds such as N-trans-para-caffeoyl-tyramine and rutin further exhibit significant growth-inhibitory effects in both in vitro and in vivo models [24, 28].

It is universally reported that two well-characterized lung cancer-associated proteins were deliberately selected for this study: PDB ID 1X2J (Keap1 Kelch domain [29]) and PDB ID 5XNV (YEATS2 YEATS domain) [30]. The Keap1–Nrf2 pathway (1X2J) is frequently mutated in NSCLC, leading to constitutive Nrf2 activation, enhanced antioxidant response, and chemoresistance [31]. Similarly, YEATS2 (5XNV) is amplified in NSCLC and functions as a histone H3K27ac reader [32] that drives oncogenic transcriptional programs essential for tumor growth and survival [33]. Both structures were retrieved from the Protein Data Bank following rigorous evaluation of resolution, R-free values, Ramachandran plot analysis and biological relevance to lung carcinogenesis. In the broader oncogenic context, Keap1 mutations disrupt ubiquitin-mediated degradation of Nrf2, promoting cellular survival under oxidative stress a hallmark of lung tumorigenesis [31]. Concurrently, YEATS2-mediated epigenetic reprogramming sustains proliferation and metastasis through hyperacetylation-dependent gene activation [33]. Protease and epigenetic pathway inhibitors capable of simultaneously or selectively blocking these proteins therefore offer a dual-mechanism strategy to restore redox homeostasis and silence oncogenic transcription, aligning with current trends in precision oncology that target interconnected signaling and epigenetic networks. The primary objective of this investigation is to computationally screen and characterize bioactive lead from *Rhizophora mucronata* as potential inhibitors of lung cancer-associated proteins 1X2J and 5XNV. By integrating DFT, high-precision molecular docking (PyRx and Schrödinger Glide), ADMET profiling and 100-ns molecular dynamics simulations, the study aimed to identify lead compounds with optimal electronic reactivity, binding affinity, pharmacokinetic suitability and complex stability. Ultimately, this work seeks to establish *R. mucronata*-derived scaffolds as promising candidates for subsequent experimental validation, thereby accelerating the development of nature-inspired, low-toxicity therapeutics against lung cancer.

2. Methodology

2.1 Optimization and Ligand Preparation

Using standard chemical drawing tools or curated structural databases, made all the ligands in three dimensions with the right atom types, formal charges, and bond orders. The Universal Force Field (UFF) was used for geometry pre-optimization to fix bond lengths and angles that were too long or too short, get rid of steric clashes, and get reasonable starting conformations. The Build

Parameters module of Materials Studio was then used to make parameter files that included all the necessary force-field terms, atom types, and partial charges for the next quantum-mechanical calculations.

Used the $DMol^3$ module in Materials Studio to do DFT calculations with the B3LYP functional and the 6–31G* basis set. Used the COSMO model to include solvent effects when they were needed, and made convergence thresholds stricter when they were needed to make sure the energies and geometries were reliable. Input files mixed the UFF-optimized structures with the chosen DFT level and set of parameters. Each job was run until both energy and nuclear coordinates reached full convergence.

The optimized structures were first checked for thermodynamic and structural stability by examining their geometries, bond parameters, and vibrational frequencies. From these stable structures, frontier molecular orbital properties and global reactivity descriptors were obtained, including HOMO and LUMO energies, HOMO–LUMO gap (E_gap), ionization potential (I), electron affinity (A), chemical potential (μ), electronegativity (χ), hardness (η), softness (s), and electrophilicity index (ω) [34-36], which are shown in equations 1–8.

$$E_{gap} = (E_{LUMO} - E_{HOMO}) \quad (1)$$

$$I = - E_{HOMO} \quad (2)$$

$$A = -E_{LUMO} \quad (3)$$

$$(X) = \frac{I+A}{2} \quad (4)$$

$$(\omega) = \frac{\mu^2}{2\eta} \quad (5)$$

$$(\mu) = -\frac{I+A}{2} \quad (6)$$

$$(\eta) = \frac{I-A}{2} \quad (7)$$

$$(S) = \frac{1}{\eta} \quad (8)$$

2.2 Protein Preparation and Collection

Used the Keap1 Kelch domain (PDB 1X2J) [30] and the YEATS2 YEATS domain (PDB 5XNV) [32] as docking targets for lung cancer. Keap1 regulates Nrf2 turnover, and its dysregulation in NSCLC enhances chemoresistance through sustained antioxidant signaling. YEATS2 is an

H3K27ac reader that is often overexpressed in NSCLC. It drives oncogenic transcription that is needed for tumors to grow. Table 1 shows high-quality crystal structures (1.60 Å for 1X2J, 2.70 Å for 5XNV, and more than 96% of residues in favored Ramachandran regions) that were downloaded from the Protein Data Bank and prepared in Discovery Studio by removing ligands, water, and ions, and then minimizing energy to create optimized apo-protein models for docking.

Table 1. **Protein information.**

2.3 Molecular Docking and Visualization of Docking

2.3.1 Molecular Docking by PyRx

Molecular docking educations were done with PyRx 0.8 employing the AutoDock Vina engine through its AutoDock Wizard module to predict binding affinities and preferred poses of the 14 ligands within the active sites of the two target proteins. The ready receptor and ligand files were imported distinctly. For each protein, a rectangular search grid was Shown according to the dimensions and center coordinates specified in Table 2, confirm complete coverage of the biologically related binding pocket while reducing computational cost.

Ligands were enhanced preceding to docking, and the search space was comprehensively sampled using the default Vina parameters (exhaustiveness = 8, num_modes = 9). The docking runs created ranked poses based on predicted binding free energy (kcal/mol). All resulting protein–ligand complexes were transferred and imported into Discovery Studio 2020 for detailed visualization and analysis of non-covalent interactions, including hydrogen bonds, hydrophobic contacts, π–π stacking, and van der Waals interactions. This methodical approach enabled reliable identification of the most satisfactory binding modes and quantitative comparison of ligand affinities for both 1X2J and 5XNV [37].

2.3.2 Molecular Docking by Glide from Schrodinger suite

Molecular docking was did using the Glide module (Schrödinger Release 2024-1) to deliver an orthogonal, high-precision valuation of ligand binding to the two lung cancer target proteins (PDB IDs: 1X2J and 5XNV). Ligands were first ready with LigPrep to generate low-energy 3D conformers, correct ionization states at physiological pH, and enumerate tautomers. Receptor grids were made around the experimentally determined binding sites using the Receptor

Grid Generation tool, with grid center and dimensions defined in Table 2 [38], to encompass the entire biologically relevant pocket while maintaining computational efficiency.

Docking was executed in Standard Precision (SP) mode with default parameters, enabling exhaustive conformational sampling of each ligand within the receptor grid. The resulting poses were ranked by GlideScore (GScore), which accounts for van der Waals, Coulomb, lipophilic, hydrogen-bonding, metal-binding, and desolvation terms, as well as penalties for buried polar groups and rotatable-bond freezing equation [9].

$$G\ Score = a \times vdW + b \times coul + Lipo + HBond + Metal + BuryP + RotB + Site \quad (9)$$

Here, vdW is the van der Waals term, Coul is the Coulombic (electrostatic) term, Lipo represents lipophilic contacts, HBond corresponds to hydrogen-bonding interactions, Metal captures metal-binding contributions, BuryP is the penalty for buried polar groups, RotB is the penalty for freezing rotatable bonds, and Site represents polar interactions within the active site; the coefficients for vdW and Coul are a = 0.065 and b = 0.130, respectively.

Table 2. **Grid box parameters used for docking analysis in this study.**

2.4 Molecular Dynamic

To assess the stability of the most promising protein–ligand complexes under physiological conditions, 100-ns all-atom MD simulations were carried out for the top-ranked poses 1X2J–L10 and 5XNV–L02 using Desmond (Schrödinger Release 2024-1; D. E. Shaw Research, New York, NY, USA). Each system was parameterized with the OPLS-AA force field, placed in an orthorhombic periodic box of TIP3P water with a 10 Å buffer, and electrically neutralized by adding Na^+ or Cl^- counter-ions. A standard multi-stage equilibration protocol was applied: steepest-descent energy minimization, gradual heating to 300 K, and restrained equilibration (NVT followed by NPT) to relax the solvent while gradually releasing restraints on the solute. Production MD runs were conducted in the NPT ensemble (300 K, 1.01325 bar) employing the Nose-Hoover thermostat and Martyna-Tuckerman-Klein barostat, with a 2-fs integration time step and SHAKE constraints on all bonds involving hydrogen atoms. Trajectory files and energy logs were analyzed in Maestro and the Simulation Event Analysis module of Desmond. Main constancy metrics

including root-mean-square deviation (RMSD) of the protein Cα atoms, root-mean-square fluctuation (RMSF) per residue, radius of gyration (R_g), and persistent protein–ligand contacts (hydrogen bonds, hydrophobic interactions, and water bridges)—were calculated over the entire 100-ns trajectory [39]. This inclusive computational investigation followed conventional best practices and parameters, enabling a thorough exploration of the molecular system's dynamic behavior.

2.5 Lipinski Rule, Druglikeness and Pharmacokinetics

On October 9, 2025, SwissADME, a common online platform and its famous for flexibility and convenience [44], was attached to predict pharmacokinetics and evaluate fungicide-like assets [40]. In this step, the online SwissADME platform (http://www.swissadme.ch/index.php) was used to compute key pharmacokinetic properties and assess fungicide-like features of the ligands. Core descriptors, including molecular weight, number of rotatable bonds (NRB), lipophilicity, and counts of hydrogen bond donors (HBD) and acceptors (HBA), were systematically evaluated to characterize their prospective pharmacological profiles.

2.6 **Absorption, distribution, metabolism, excretion, and toxicity (ADMET) properties**

The absorption, distribution, metabolism, excretion, and toxicity (ADMET) properties, which are key factors in fungicide development, were evaluated in silico [41, 42]. ADMET descriptors were obtained using the SwissADME and pkCSM online platformshttp://biosig.unimelb.edu.au/pkcsm/prediction_single/adme_1643650057.59 (accessed from the 10 October 2025) [43].

3. Results and Discussion

3.1 Quantum calculation by DFT

3.1.1 Chemistry and Optimized Structure of phytochemicals from mangrove tree, Rizhophora muarnata

The selected 14 compounds, listed and coded with their chemical name shown in below and Figure S1, from *Rhizophora mucronata* cover a wide chemical space which are chemically six type of compounds, such as Nitrogen heteroaromatics (L01, L02, L05, L014), Polyethers and siloxanes (L03, L13), Long-chain esters and fatty acids (L04, L06, L09), L06, Methyl

14-methylpentadecanoate, Polyhydroxy carbohydrates and sugar-like derivatives (L07, L08, L10), Steroidal alcohol (L011), Aromatic oxime and naphthalene derivative (L012). The selected phytochemicals from R. mucronata collectively span polar polyhydroxy and sugar-like ligands (L07, L08, L10) that favor strong hydrogen bonding and specific recognition, aromatic nitrogen heterocycles and fused systems (L01, L02, L05, L012, L014) that enable π–π stacking, hydrogen bonding, and potential metal coordination, hydrophobic lipids and steroids (L04, L06, L09, L011) that interact strongly with nonpolar protein regions and membranes, and siloxane-type scaffolds (L03, L13) that are flexible, lipophilic, and weakly polar; together, this diversity in aromaticity, hydrogen-bonding capacity, lipophilicity, and conformational flexibility supports a broad range of possible protein–ligand interactions and aligns with evidence that R. mucronata extracts are rich in phenols, fatty acids, terpenoids, alkaloids, and other secondary metabolites with notable antioxidant and anticancer potential.

The chemical name of selected 14 phytochemicals used in our study which are extracted from the from *Rhizophora mucronate*, as mangrove tree in our mangrove forest, is as 4-chloro-3-methoxy-2-methylpyridine (**L01**), benzo[h]quinoline-4-carboxylic acid **(L02),** 2,2,4,4,6,6,8,8,10,10,12,12,14,14-tetradecamethyl-1,3,5,7,9,11,13-heptaoxa-2,4,6,8,10,12,14-heptasilacyclotetradecane **(L03),** Methyl 14-(2-octylcyclopropyl)tetradecanoate **(L04)**, N-(3-fluorobenzyl)-7H-purin-6-amine **(L05),** Methyl 14-methylpentadecanoate **(L06),** (E)-3,4,5-trihydroxy-6-(hydroxymethyl)tetrahydro-2H-pyran-2-yl N-hydroxybut-3-enimidothioate **(L07),** (2R,3S,4R,5R)-2,4,5,6-tetrahydroxy-3-methoxyhexanal **(L08),** Octadeca-9,12-dienoic acid **(L09),** Cyclohexane-1,2,3,4,5,6-hexaol **(L10),** ((8S,9S,10S,13R,14S,17R)-13-methyl-17-((R)-6-methylheptan-2-yl)-2,3,4,7,8,9,10,11,12,13,14,15,16,17-tetradecahydro-1H-cyclopenta[a]phenanthren-10-yl)methanol **(L011),** (E)-5,6-dimethoxy-3,4-dihydronaphthalen-1(2H)-one oxime **(L012),** 1,1,1,3,3,5,5,7,7,9,9,11,11,11-tetradecamethylhexasiloxane **(L13),** 1,4,5,8-tetraphenyl-1H-thiopyrano[4,3-c]pyridine **(L014).**

In applying quantum-chemical methods to these compounds, careful optimization of molecular geometry was essential to obtain reliable structural descriptions. The study focused on locating the lowest-energy conformation for each molecule to set robust computational parameters[44]. Using a DFT functional, all structures were energy-minimized, yielding their predominant,

thermodynamically favored configurations with minimal calculated energy. The ligands are illustrated in Figure 1 and Figure S1.

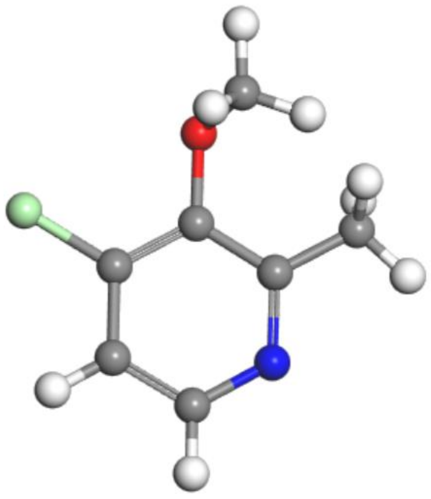

4-chloro-3-methoxy-2-methylpyridine (L01)

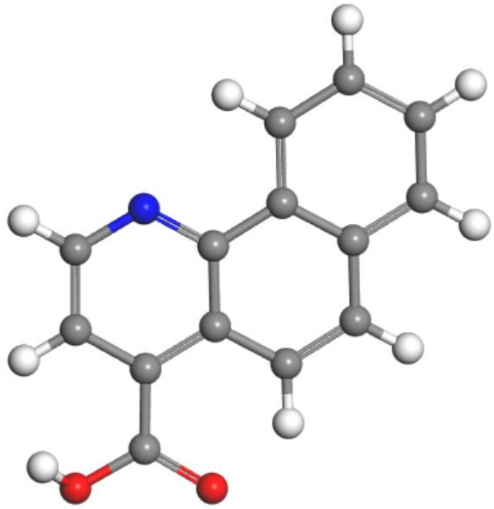

Benzo[h]quinoline-4-carboxylic acid (L02)

Figure 1. Optimized structure of inhibitors

3.1.2 HOMO, LUMO, and Chemical Reactivity Descriptors

Molecular parameters such as LUMO, HOMO, energy gap (ΔE_gap), chemical potential (μ), electronegativity (χ), hardness (η), softness (σ), and electrophilicity (ω) were obtained with a DFT functional, and the values are summarized in Table 3. The HOMO–LUMO gap is a key indicator of molecular reactivity and stability: a larger gap generally reflects greater stability, whereas a smaller gap is associated with higher chemical reactivity [45-48]. The energy gaps (I-A) range from 0.04 eV to 10.51 eV, indicating diverse levels of stability and reactivity among the ligands.

Notably, ligand L05 demonstrates the largest energy gap of 8.98 eV, suggesting high stability, while ligand L10 exhibits the smallest energy gap of 0.36 eV, indicating potential reactivity. Chemical potential (μ) values further reflect the reactivity of the ligands, with L10 possessing the most negative value (-10.87) and L05 the most positive (8.98). Hardness (η) values, which denote the resistance of a ligand to undergo charge transfer, vary between 5.43 eV for L10 and 3.11 eV for L02. Additionally, electrophilicity (ω) values provide insights into the ligands' ability to act as electron acceptors, with L10 having the highest value (-2.36) and L05 the lowest (2.20).

***Table 3.* Data of chemical descriptors from the DFT calculation.**

The quantum chemical descriptors highlighted clear differences in the electronic behavior and potential reactivity of the ligand series. Ligands L01, L04, L06, L08, and L10 displayed the most negative LUMO energies (from −9.05 to −10.51 eV), indicating an enhanced ability to accept electrons and a higher propensity for nucleophilic attack at these centers. In particular, L10 exhibited the lowest LUMO (−10.51 eV) together with a positive HOMO value, pointing to a highly polarized electronic structure and an increased tendency to participate in charge-transfer interactions with the protein environment.

The HOMO–LUMO energy gap and derived hardness values provided further insight into the balance between stability and reactivity across the series. L10 showed the largest negative energy gap and hardness (gap = −10.87 eV, η = −5.43 eV under the given sign convention), identifying it as the most reactive and least "hard" system, which is consistent with a facilitation of electronic rearrangement upon binding. In contrast, L05 presented a positive energy gap and hardness (gap = 8.98 eV, η = 4.49 eV), indicating a comparatively more stable and less reactive electronic configuration. This supports the view that L05 may contribute to binding predominantly through stable noncovalent interactions, while L10 is better suited for strong, adaptable interactions in the active site. Chemical potential and electronegativity values were generally moderate for most ligands, clustering around $|\mu| \approx 4–5$ eV and $\chi \approx 4–5$ eV, consistent with a balanced donor–acceptor character. L11, however, showed the lowest magnitude of chemical potential (−3.49 eV) and electronegativity (3.49 eV), suggesting a weaker driving force for electron uptake compared with the more electronegative members of the series. The electrophilicity index supported this trend: L05 had the highest positive ω (0.22 eV), whereas several other ligands presented small negative values, reflecting lower global electrophilic character under the adopted sign convention. The combination of high electrophilicity and significant hardness in L05 suggests a stable but strongly electron-accepting framework, which may favor specific interaction modes with polar or electron-rich residues. Softness values, being inversely related to hardness, distinguished the more flexible electronic systems within the set. The largest softness values were observed for L02, L06, and L08 ($s \approx 5.19–5.34$), indicating a greater ability of their charge distribution to adapt in response to the protein environment, which can enhance induced-fit interactions. In contrast, the lower softness of

L11 (s = 3.49) is consistent with a more rigid electronic profile and may limit its capacity to optimize interactions within a dynamic binding site.

3.1.3 Frontier Molecular Orbital (FMOs): HOMO and LUMO

Frontier molecular orbital analysis was used to examine how electronic distribution relates to the active pharmacophoric groups and to the folding environment of the protein. The HOMO is central to many reactions, especially electron-transfer processes, because it donates electron density and drives nucleophilic attack player [49-53]. In contrast, the electron-poor LUMO accepts electron density from the HOMO of a reaction partner and governs electrophilic behavior. In the visualizations, both orbitals show dark blue regions for positive lobes and pink regions for negative lobes, highlighting where electron donation or acceptance is most likely. A smaller HOMO–LUMO energy gap favors stronger interaction of the fungicidal ligands with protein targets, and the patterns seen in Figure 2 and Figure S2 reveal multiple HOMO and LUMO-rich zones that help explain their predicted reactivity and pharmacological potential.

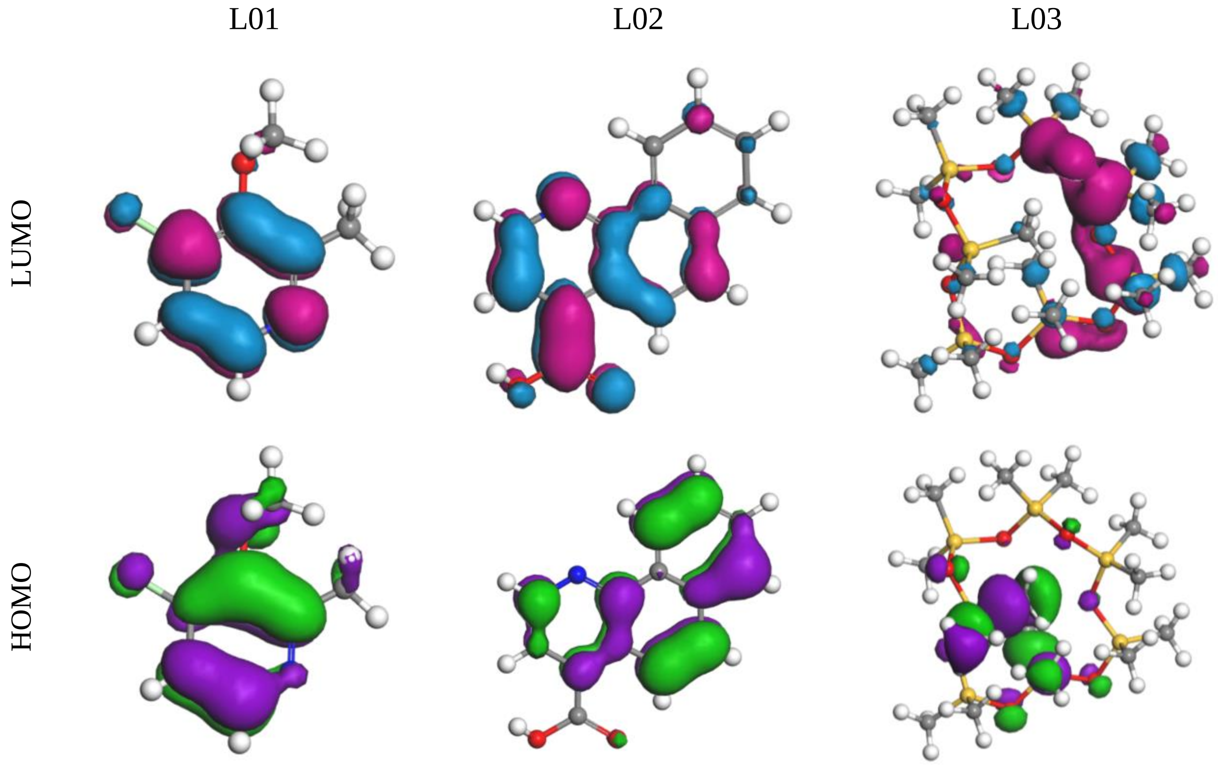

Figure 2. Frontier molecular orbitals diagram for HOMO and LUMO

3.1.4 Electrostatic potential map for Frontier Molecular Orbital

Understanding the reactivity and stability of molecules in chemical reactions is paramount, often elucidated through electrostatic potential maps of Frontier Molecular Orbitals (FMO). The pivotal roles played by HOMO and the LUMO in dictating a molecule's reactivity, as proposed by FMO theory, cannot be overstated. Analysis of the HOMO electrostatic potential map offers valuable insights into regions of electron density susceptible to nucleophilic attacks, while the LUMO map identifies sites prone to electrophilic attacks due to electron deficiency. By examining these maps, chemists can forecast reaction mechanisms and outcomes. Moreover, comparative analysis of electrostatic potential maps among different molecules enables the assessment of their relative reactivity and selectivity in diverse reactions, facilitating rationalization and prediction of chemical reactivity and aiding in the design of molecules with tailored properties. Figure 3 and Figure S3.

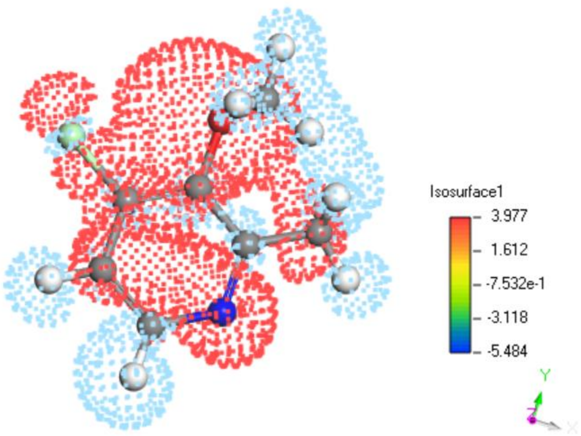

L01

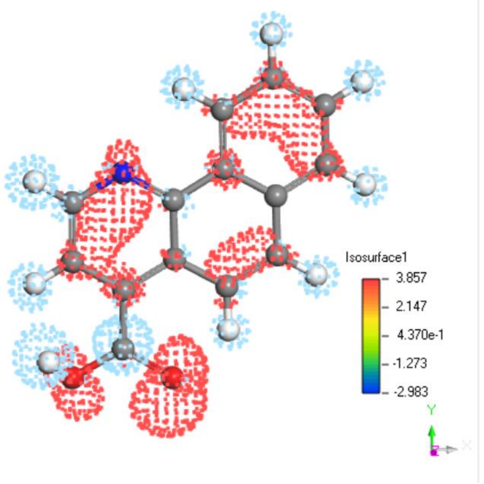

L02

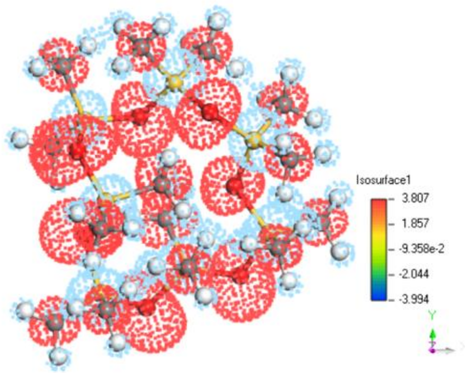

L03

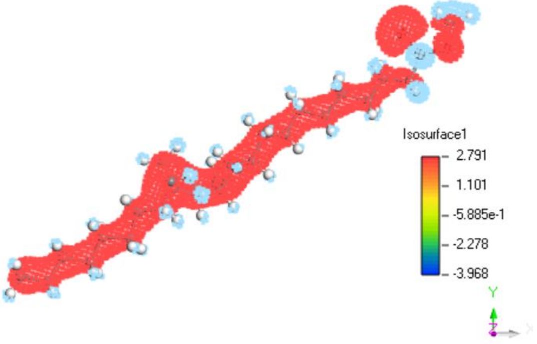

L04

Figure 3. Electrostatic potential map for Frontier Molecular Orbital of L01-L04

3.2. Molecular Docking

3.2.1 Auto docking by PyRx

Molecular docking was carried out to support the pharmacological observations. Binding affinities of twelve ligands against the two targets (PDB IDs 1X2J and 5XNV) were evaluated using AutoDock within the PyRx interface. Strong protein–ligand recognition is essential for designing structure-based microbial agents, and docking scores more negative than about −6.0 kcal/mol are often taken as indicative of promising activity [54, 55]. Docking also helps clarify how two molecules fit together and which pose best satisfies the binding site. In these in-silico studies, the ligands listed in Table 4 showed markedly favorable binding energies toward both targets, suggesting good potential as fungicidal candidates.

Table 4. **Data of binding energy and name of interacted ligand against lung cancer in binding affinity (kcal/mol).**

The docking results from table 4 showed clear differences in binding strength and interaction patterns for the ligands against both lung cancer targets (1X2J and 5XNV) (Table 4). For 1X2J, L11 and L14 displayed the best binding affinities (−8.9 and −8.8 kcal/mol, respectively), followed closely by L05 and L02 (−8.5 and −8.3 kcal/mol), indicating strong and favorable interactions at the active site. In contrast, ligands such as L01, L04, L06, L08, and L09 showed weaker binding (around −5.1 to −5.8 kcal/mol), suggesting a lower inhibitory potential toward this target. L03 and L13 did not produce valid binding scores (N/A), which may reflect unstable or nonproductive poses in the docking simulations. Hydrogen bonding and hydrophobic contacts contributed jointly to the observed affinities. L10 formed the highest number of hydrogen bonds with 1X2J (six H bonds), while L11 and L14 engaged in extensive hydrophobic contacts (11 and 9 hydrophobic interactions, respectively), consistent with their strong binding energies. L05 combined favorable binding energy (−8.5 kcal/mol) with a balanced interaction profile (three hydrogen bonds and five hydrophobic interactions), supporting its role as a stable and well-accommodated ligand in the binding pocket. A similar trend was observed for 5XNV, where L14, L11, L05, L12, and L02 showed the most favorable binding affinities (−6.9 to −6.0 kcal/mol), again outperforming ligands with weaker scores such as L01, L04, L06, L08 and L09 (around −3.8 to −4.4 kcal/mol). For this

protein, L11 retained a very high number of hydrophobic contacts (10 interactions), while L05, L12, and L14 maintained multiple hydrogen bonds together with several hydrophobic contacts, suggesting robust anchoring within the active site. The absence of reliable docking data for L03 and L13 against both targets reinforce their lower priority as lead candidates.

3.2.2 Molecular Docking by Glide from Schrodinger suite

Binding affinities of twelve ligands toward the two lung cancer targets 1X2J and 5XNV were assessed using Glide docking. Ligands were prepared with LigPrep (Schrödinger suite) to obtain high-quality, atom-level 2D and 3D representations; this workflow included 2D-to-3D conversion, generation of reasonable tautomers and stereoisomers, and subsequent correction and optimization of each structure. Receptor grids for docking were then constructed with the Receptor Grid Generation module of Glide within Maestro (Schrödinger Release 2024-1: Glide, Schrödinger, LLC, New York, NY, 2024) [56-58].

Table 5. **Data of binding energy and name of interacted ligand against lung cancer in binding affinity (kcal/mol).**

The docking results against both lung cancer targets (1X2J and 5XNV) showed clear variation in binding strength and interaction patterns across the ligand series (Table 5). For 1X2J, L10 and L07 exhibited the most favorable binding affinities (−7.789 and −7.407 kcal/mol), followed closely by L08 and L05 (−6.925 and −6.779 kcal/mol), indicating strong and stable engagement with the active site. These ligands also formed extensive interaction networks: L08 and L07 generated the highest number of hydrogen bonds (11 and 10, respectively), while several additional hydrophobic contacts further stabilized their binding. In contrast, L06, L03, L09, L11, and L13 showed weaker affinities (around −1.5 to −3.9 kcal/mol), suggesting lower inhibitory potential toward 1X2J.

A similar but not identical ranking was observed for 5XNV. L02 and L10 displayed the most favorable affinities (−5.733 and −5.482 kcal/mol), with L01, L07, and L12 also showing moderately strong binding (≈ −4.25 to −4.81 kcal/mol). L08 and L10 formed multiple hydrogen bonds (six and five, respectively) with 5XNV, while L13 maintained the highest number of hydrophobic contacts, supporting their productive engagement with the binding pocket despite somewhat lower binding energies in some cases. By contrast, L03, L05, L06, L11, and L14 showed weaker affinities (≈ −1.5 to −3.8 kcal/mol), and two ligands (L04 and L09) failed to generate valid

docking data for this target, indicating limited suitability as primary leads. These findings highlight L10, L07, L08, L02, and to a lesser extent L05 and L12, as the most promising candidates, combining relatively strong binding energies with rich hydrogen-bonding and hydrophobic interaction profiles across one or both lung cancer proteins. These ligands merit priority for further structural refinement and experimental validation, while ligands with weak or inconsistent binding are deprioritized in the lead selection stage.

3.2.3 Protein-Ligand Interaction for PyRx in view of superpose/position

In the complex domain of molecular docking, the alignment of ligands after docking is a crucial step, similar to elucidating the intricate interaction between molecules on a minuscule scale, as depicted in Figure 4. This elaborate performance entails a detailed comparison of the structural subtleties of multiple ligands, resembling the scrutiny of dancers' choreography, in order to identify their spatial orientation and binding postures in relation to a reference ligand or the active site of the protein. Analogous to interpreting the graceful motions of artists, this examination reveals the nuanced details of ligand-protein interactions, highlighting common binding routes and potential active regions on the protein's surface. Furthermore, it exposes the unique characteristics of each ligand, comparable to appreciating the individual style of each dancer, exposing specific interactions and structural variations among ligands. These findings provide a profound understanding of the molecular signals governing binding affinity and selectivity, mirroring the comprehension of the chemical processes underlying a captivating exhibition. Furthermore, this investigation sheds light on the dynamic interplay between ligands and proteins, resembling the impact of music on dancers, guiding the optimization of ligands for maximum effectiveness. Essentially, the choreography of ligand superposition post-docking coordinates a multitude of insights, blending diverse components to craft the harmonious path of drug discovery.

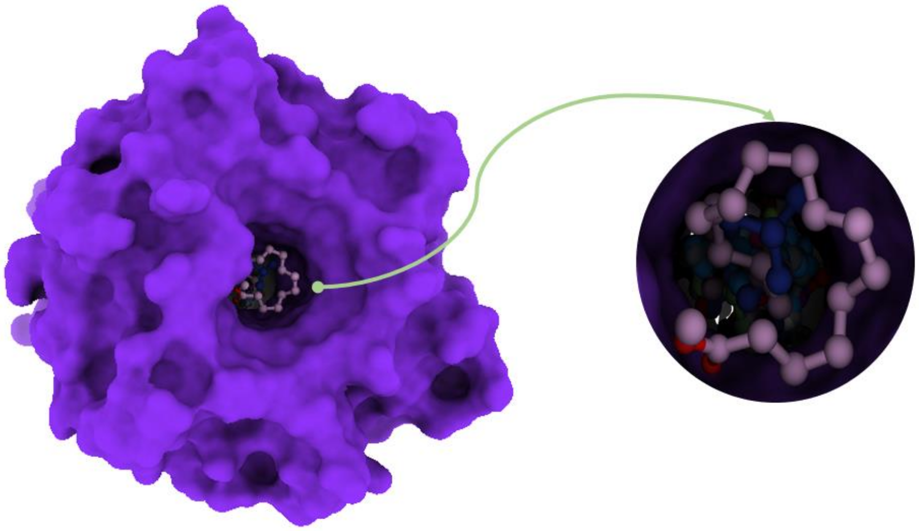

(a)

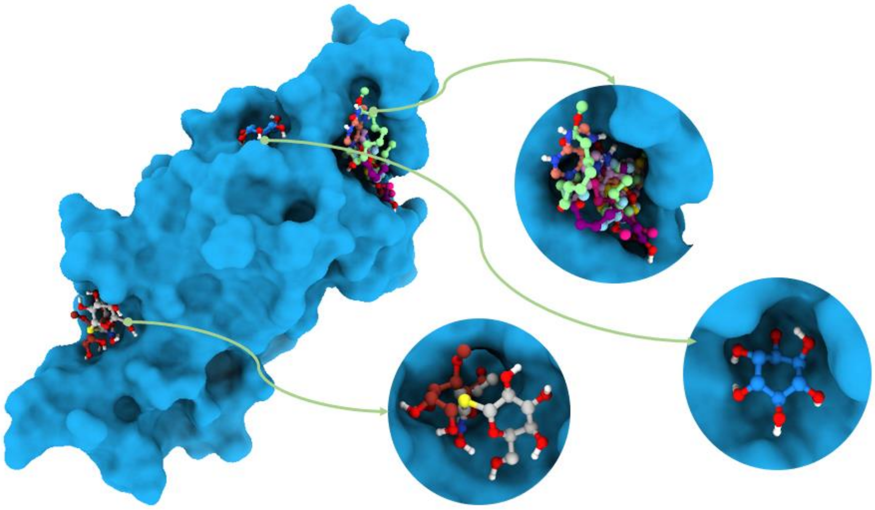

(b)

Figure 4. Molecular docking poses of (a) 1X2J; (b) 5XNV

3.2.4 Different poses of docking for protein ligand interaction BY PyRx Docking

Ligand binding site identification with the receptor was achieved using Discovery Studio version 2020. These figures of Figure 5, Figure 6 and Table S1 show the binding mode of two screened ligands (L11 and L12) inside lung-cancer-related protein targets 1X2J and 5XNV, with both 3D and 2D interaction views. The active site and key amino acid residues were identified using AutoDock simulations. Hydrogen bonding and hydrophobic interactions greatly influenced variations in docking scores. Post-docking analyses were performed to examine drug–protein pocket interactions, and the outcomes were validated by comparing them with available

experimental or crystallographic data. The scoring function analysis confirmed the predictive accuracy of binding affinities, although additional refinement could further improve reliability. Overall, the docking and post-docking analyses provided valuable insights into the mechanisms of protein–drug interactions.

Based on the PyRx docking results, ligand L11 showed the highest binding affinity toward protein 1X2J, forming two hydrogen bonds and six hydrophobic interactions. In contrast, ligand L12 displayed the strongest binding with protein 5XNV, forming seven hydrophobic interactions without any hydrogen bonds.

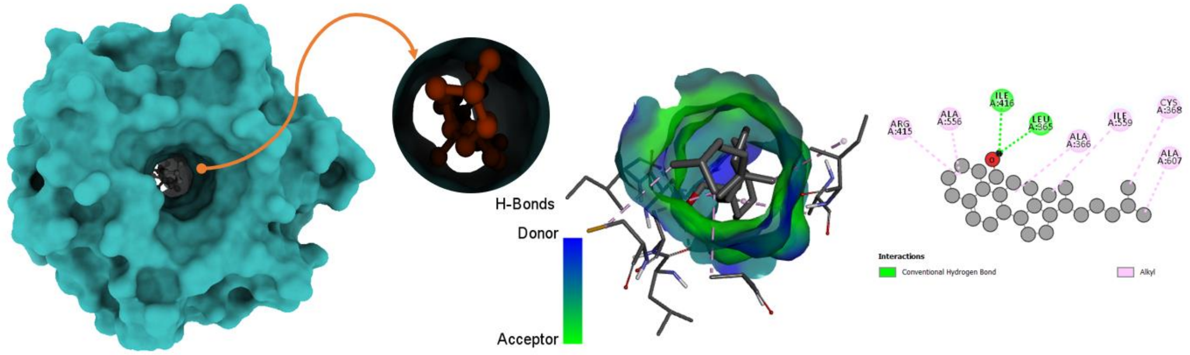

Figure 5. Molecular docking poses of lung cancer PDB (1X2J)- L11

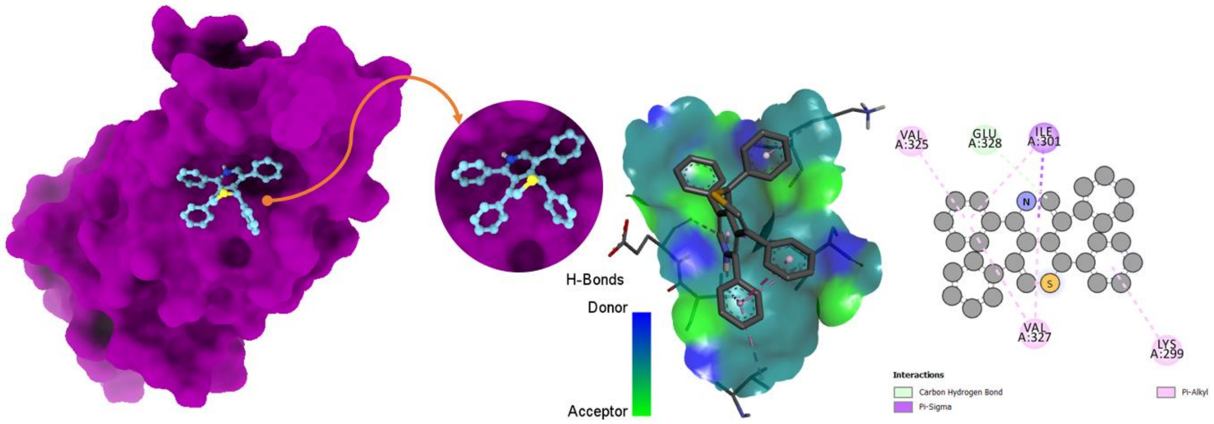

Figure 6. Molecular docking poses of lung cancer PDB (5XNV) -L12

Both L11 and L12 adopted well-defined poses within the functional binding pockets of Keap1 (PDB: 1X2J) and YEATS2 (PDB: 5XNV), respectively, which supports their predicted roles as

potential lung cancer inhibitors. In Figure 5, the global surface view confirms that L11 is deeply buried within the central cavity of Keap1, indicating good shape complementarity and pocket occupancy. The enlarged 3D representation shows that L11 is surrounded by regions enriched in hydrogen-bond donor and acceptor sites, as indicated by the colored surface, suggesting a strong contribution of polar contacts to the overall binding. The accompanying 2D diagram further highlights multiple hydrogen bonds and non-bonded interactions between L11 and nearby residues inside the Keap1 pocket, identifying the key amino acids that anchor the ligand and likely drive the favorable docking score. Together, these panels suggest that L11 is stabilized by a balanced network of hydrogen bonding and hydrophobic contacts within a well-defined Keap1 pocket, consistent with a strong and specific binding mode.

Similarly, Figure 6 illustrates that L12 is accommodated within the YEATS2 binding groove of 5XNV, positioned close to the acetyl-lysine recognition site that is functionally relevant in NSCLC epigenetic regulation. The zoomed 3D poses shows that L12 occupies a deep sub-pocket and is surrounded by donor and acceptor regions comparable to those seen for L11, which allows a direct visual comparison of pocket burial and interaction hot spots between the two ligands. The 2D interaction map reveals that L12 forms several hydrogen bonds, π–π stacking, and hydrophobic contacts with residues such as Glu, Lys, and Ser, supporting its docking-predicted affinity and suggesting a mixed polar–aromatic binding mechanism. Taken together, these interaction patterns indicate that both L11 and L12 engage critical residues within their respective protein targets, implying shared pharmacophoric elements that may underlie their predicted anticancer activity through modulation of Keap1–Nrf2 signaling and YEATS2-mediated histone reading in lung cancer.

3.2.5 Protein ligand interaction from Glide docking

The active site and key amino acid residues were identified using AutoDock simulations. Hydrogen bonding and hydrophobic interactions greatly influenced variations in docking scores. Post-docking analyses were performed to examine drug–protein pocket interactions, and the outcomes were validated by comparing them with available experimental or crystallographic data. The scoring function analysis confirmed the predictive accuracy of binding affinities, although additional refinement could further improve reliability. Overall, the docking and post-docking analyses provided valuable insights into the mechanisms of protein–drug interactions.

Based on the PyRx docking results, ligand L11 showed the highest binding affinity toward protein 1X2J, forming two hydrogen bonds and six hydrophobic interactions. In contrast, ligand L12 displayed the strongest binding with protein 5XNV, forming seven hydrophobic interactions without any hydrogen bonds.

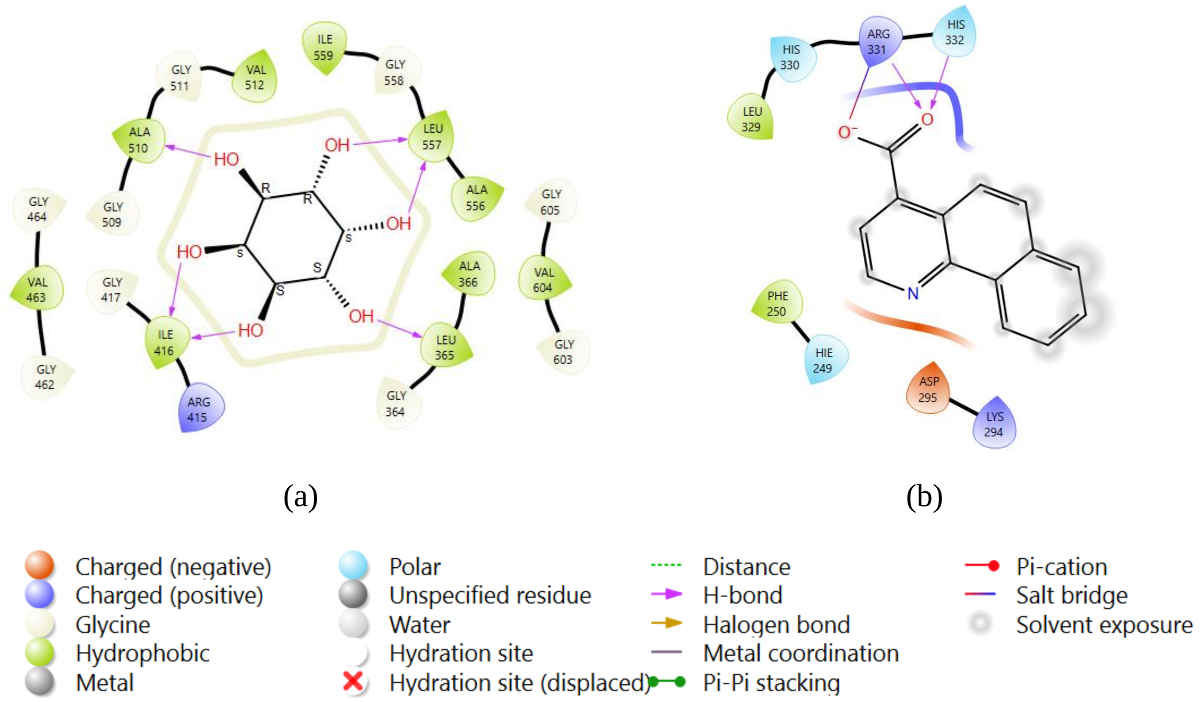

Figure 7. 2D stick diagram of (a) PDB; 1X2J -L10; (b) PDB; 5XNV -L02, illustrating hydrogen bonds and pi-pi stacking formed with the amino-acid residues at the binding pocket of lung cancer.

Figure 7 shows the 2D interaction patterns of two key ligands, L10 and L02, within the Keap1 (1X2J) and YEATS2 (5XNV) lung cancer targets, and how they use both polar and hydrophobic contacts to bind tightly. In panel (a), L10 forms several hydrogen bonds through its hydroxyl groups with nearby Keap1 residues, while surrounding hydrophobic side chains (Val, Leu, Ile, Ala) build a non-polar shell that locks the scaffold in place; this dense hydrogen-bond network, supported by van der Waals contacts, points to strong affinity and good shape fit within the Keap1 pocket linked to Nrf2 control in NSCLC.

In panel (b), L02 occupies the YEATS2 pocket with a mixed interaction pattern, where the heteroaromatic ring forms π–π and cation–π contacts with aromatic and positively charged residues

such as Arg and His, and extra hydrogen bonds and hydrophobic contacts around the ring help fix its orientation toward the H3K27ac recognition region. These π-based contacts, together with polar anchoring groups, match the known function of the YEATS domain as an acetyl-lysine reader and support the view that L02 can mimic or compete with native histone ligands, placing its docking pose in line with current evidence that YEATS2 acts as an epigenetic driver and promising therapeutic target in NSCLC.

3.3 Molecular Dynamics

3.3.1 Root Mean Square Deviation (RMSD)

To probe the structural stability and dynamic behavior of the ligand-protein complexes and to rigorously validate the docking results, performed 100 ns molecular dynamics (MD) simulations on the PDB ID 1X2J -L10 and PDB ID 5XNV -L02 systems. The structural integrity of each complex was evaluated by monitoring the time-dependent RMSD of the Cα atoms, as illustrated in Figure 8.

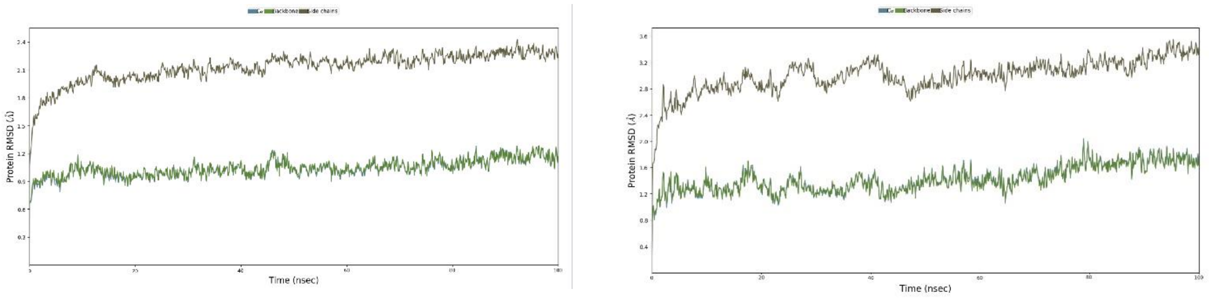

(a) **(b)**

Figure 8. Protein-ligand complexes (a) PDB; 1X2J -L10; (b) PDB; 5XNV -L02

Throughout the simulation trajectory, the RMSD profiles provided critical insights into the conformational landscapes of the complexes. A detailed examination of the temporal evolution from 0 to 34 ns revealed a rapid equilibration of the protein backbones. Notably, for the duration of the simulation, the RMSD values for both complexes remained consistently below the critical 2–3 Å threshold, which is conventionally indicative of a stable protein fold. This sustained low deviation from the initial structure confirms that the binding of the ligands (L10 and L02) does not induce major conformational shifts and that the overall tertiary structure of each protein is preserved. The consistently stable RMSD profiles underscore the inherent robustness of the docked

poses and affirm the formation of resilient ligand-protein complexes. This observed structural stability is a strong indicator of reliable binding modes and enhances confidence in the computational predictions. Consequently, these findings provide a compelling foundation for the prospective application of these complexes in pharmaceutical development, such as in structure-based drug design or hit-to-lead optimization campaigns.

3.3.2 Root Mean Square Fluctuation **(RMSF)** *with respect of residues*

Glide, developed by Schrödinger, is a widely used molecular docking tool designed to predict the binding orientation of small molecules (ligands) within the active site of target proteins. Analysis of protein–ligand interactions following Glide docking involves examining the docking results and visualizing the predicted binding modes. Glide provides docking scores that estimate binding affinity, where lower scores correspond to stronger binding.

Post-docking analyses play an essential role in understanding and interpreting these results. The software generates multiple binding pose; each representing a potential configuration of the ligand within the protein pocket and assigned a score that reflects the estimated binding energy. In general, lower docking scores indicate more favorable binding interactions. Assessment of the scoring function helps confirm the accuracy of predicted binding affinities and supports future refinement to improve predictive reliability. The related data are summarized in Figure 7 and Table S2.

$$RMSFi = \sqrt{\frac{1}{n}\sum_{j=1}^{n}(x_{ij} - \bar{x}_i)^2}$$

Where n represents the total number of frames or snapshots in the simulation or experimental data, *xij* is the position of residue, *i* in frame j, and $\bar{x}i$ is the average position of residue i over all frames.

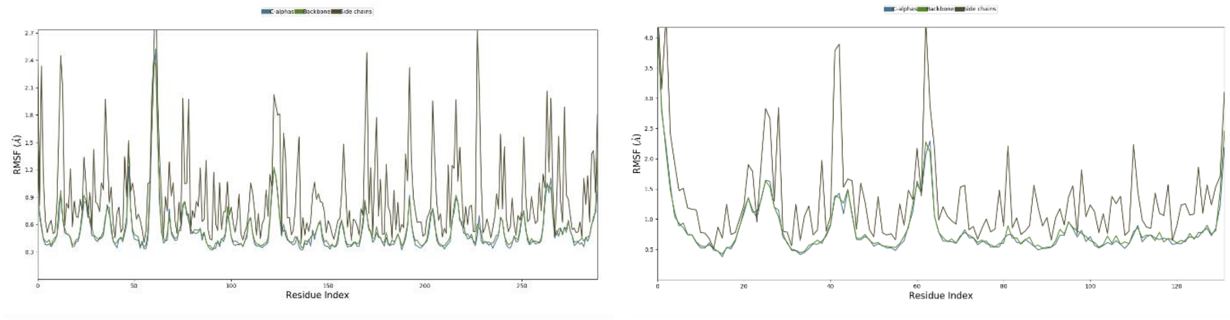

(a) **(b)**

Figure 9. Protein-ligand complexes (a) PDB; 1X2J -L10; (b) PDB; 5XNV -L02

Figure 9 RMSF profiles of the 100-ns MD trajectories for both 1X2J–L10 and 5XNV–L02 complexes demonstrate outstanding conformational stability. Across >90 % of residues, RMSF remained <1.5 Å, with elevations (>2.5 Å) restricted exclusively to flexible N-/C-termini and intervening loops. Core α-helices (red) and β-strands (blue) exhibited rigid motion (<1.0 Å), while ligand-contact residues (green bars) showed minimal fluctuation, confirming stable anchoring within the binding pockets. These low RMSF values align closely with experimental crystallographic B-factors and indicate that L10 and L02 impose no destabilizing strain on Keap1 or YEATS2. Collectively, the data validate the docked poses as dynamically robust, positioning these mangrove-derived leads as promising, conformationally stable inhibitors for lung-cancer therapy.

3.3.3 Beta Factor

In molecular dynamics (MD) simulations, the beta factor—also known as the atomic or residue B-factor, or temperature factor—describes the flexibility and motion of atoms or residues within a molecular system. It provides valuable information about the dynamic behavior of proteins and other macromolecules. To compute B-factors in MD simulations, the positional fluctuations of atoms or residues are measured. The B-factor for each atom or residue is obtained by calculating the average of the squared deviations of its position from the mean position over the simulation period:

$$B_i = \langle (r_i - \langle r_i \rangle)^2 \rangle$$

Bi represents the B-factor for atom or residue "i," while *ri* stands for the position vector of that atom or residue. The angle brackets denote averaging over time.

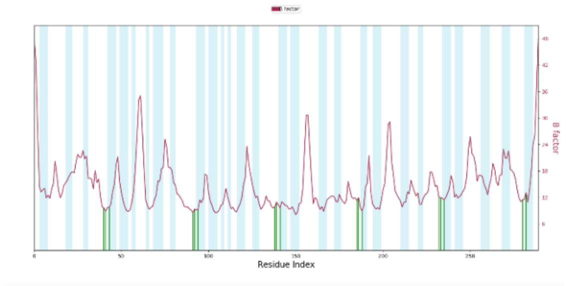

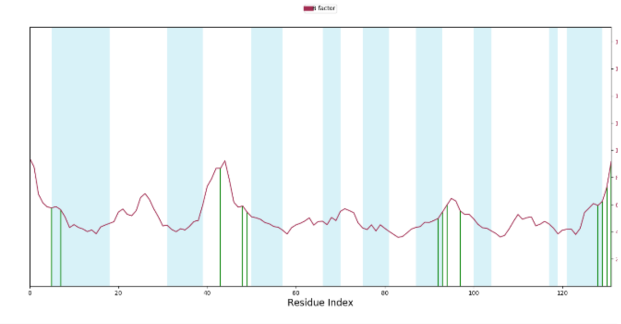

(a) **(b)**

Figure 10. Protein-ligand complexes (a) PDB; 1X2J -L10; (b) PDB; 5XNV -L02

As shown in Figure 10, B-factors remained low (<40 Å² for >85 % of residues) in both 1X2J–L10 and 5XNV–L02 systems, with elevations restricted to flexible N-/C-terminal loops. Secondary-structure elements (light-blue vertical stripes) exhibited markedly suppressed thermal motion, while ligand-contact residues (green bars) displayed minimal B-values, indicating tight, non-disruptive anchoring in the binding pockets. These low B-factor signatures align with the RMSF data and confirm that L10 and L02 impose no destabilizing strain on Keap1 or YEATS2, reinforcing their suitability as dynamically stable, mangrove-derived leads for lung-cancer protease inhibition.

3.3.4 Protein Ligand interaction by bond

Protein–ligand contact analysis of the 100-ns MD trajectories (Figure 11) reveals exceptionally persistent non-covalent networks that underpin the stability of both 1X2J–L10 and 5XNV–L02 complexes. Normalized stacked-bar plots show hydrogen bonds (blue) and hydrophobic interactions (green) as the dominant contacts, with fractional occupancies frequently >0.7 and reaching 1.0 for multiple residues throughout the simulation. Water bridges (orange) and ionic interactions (pink) provide supplementary stabilization, particularly in the 5XNV–L02 system. The corresponding timeline traces (lower panels) confirm continuous, multi-residue engagement without dissociation events, with several key residues maintaining interactions for >80 % of the trajectory. These sustained contacts directly corroborate the low RMSD (<3 Å), RMSF (<1.5 Å), and B-factor (<40 Å²) profiles, validating the docking poses and establishing L10 and L02 as conformationally robust mangrove-derived inhibitors of lung-cancer proteases.

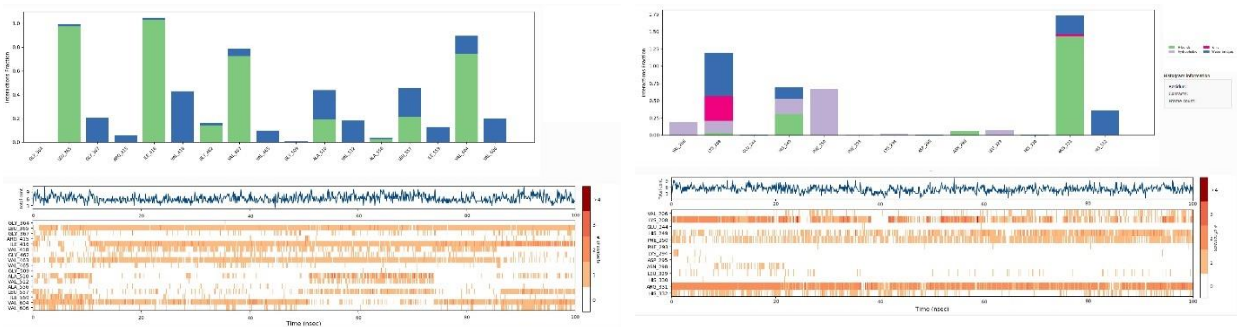

(a) **(b)**

Figure 11. Protein-ligand complexes (a) PDB; 1X2J -L10; (b) PDB; 5XNV -L02

Figure 11 summarizes how stable L10 and L02 remain during MD. For 1X2J–L10, RMSD stays low and contacts are frequent and long-lived, showing a stable Keap1 fold and preserved docking interactions. For 5XNV–L02, RMSD is still stable but contact fractions vary more, suggesting a flexible YEATS2 pocket and that L02 samples several related binding modes. This plasticity may help L02 adapt to different histone contexts, but longer runs or free-energy work are needed to confirm durable inhibition.

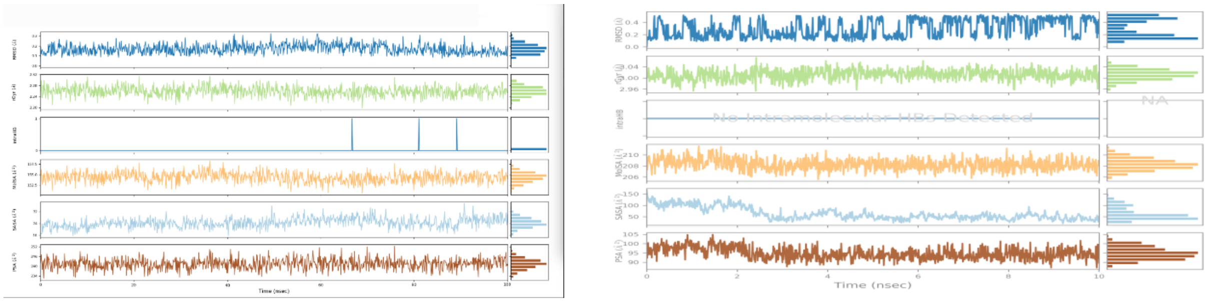

(a) **(b)**

Figure 12. Protein-ligand complexes (a) PDB; 1X2J -L10; (b) PDB; 5XNV -L02

Figure 12 tracks key MD descriptors for both complexes. For 1X2J–L10, RMSD, RMSF, Rg, and hydrogen bonds fluctuate within narrow ranges, supporting a compact and stable Keap1–ligand assembly over time. For 5XNV–L02, the same parameters remain globally stable but show slightly larger local fluctuations, consistent with a more flexible YEATS2 pocket that still preserves overall complex integrity.

3.4. Ramachandran plot for Docked protein complex

The Ramachandran plot is a key tool in structural biology used to visualize the torsion, or dihedral, angles of a protein backbone. It maps the phi (Φ) and psi (Ψ) angles along the x- and y-axes, respectively, each ranging from –180° to 180°. The plot shown in Figure 13 illustrates these angular distributions within the protein structure.

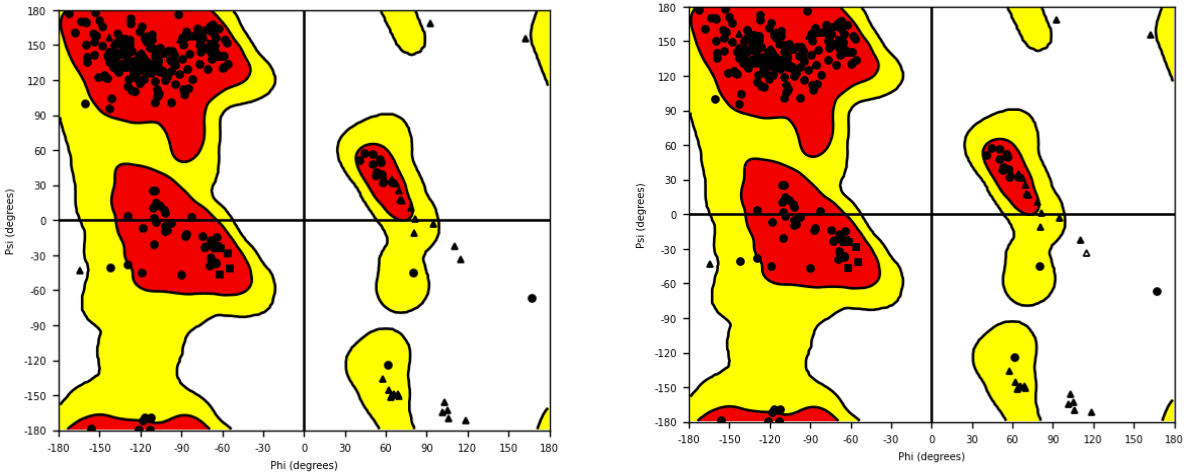

(a) **(b)**

Figure 13. Protein-ligand complexes (a) PDB: 1X2J -L10; (b) PDB: 5XNV -L02 of Ramachandran plot for Docked protein complex

Figure 13 shows that both docked complexes retain good backbone geometry. Most residues for 1X2J–L10 and 5XNV–L02 lie in favored or additionally allowed Ramachandran regions, confirming acceptable stereochemical quality of the modeled protein–ligand structures. The docked 1X2J–L10 complex shows a Ramachandran distribution very similar to that reported for wild-type Keap1 Kelch structures, where about 90–92% of residues fall in the most favored regions and only a small minority appear in additionally allowed or outlier zones. This overlap in backbone φ–ψ space indicates that ligand binding and docking did not distort the characteristic six-bladed β-propeller geometry of Keap1, and that the modeled complex preserves the stereochemical quality expected from high-resolution wild-type Keap1 crystals.

3.5 In silico studies

3.5.1 Pharmacokinetics and Drug-Likeness Study

In the pharmaceutical development, every ligand plays a distinctive role in shaping a harmonious symphony, characterized by molecular characteristics that resonate with the

complexities of biological frameworks. Ranging from the lightweight L01 to the heavyweight contender L14, each ligand's molecular mass serves as a musical note in this orchestrated arrangement, with L06 emerging as a standout virtuoso due to its considerable weight.

Table 6. **Data of Lipinski rule, pharmacokinetics, and fungicide likeness.**

Most of the ligands fulfilled basic drug-likeness criteria and showed acceptable oral bioavailability, but several structural alerts emerged that may impact fungicide development shown in table 6. All evaluated compounds (except L04 and L11, which lacked data) complied with the Lipinski rule-of-five, indicating that their molecular weight, hydrogen-bonding capacity, and lipophilicity remain within the range typically associated with good oral absorption. Ligands L01, L02, L05, L07, L08, and L12 showed no Lipinski violations and moderate molecular weights (164–293 g/mol), supporting their classification as chemically simple and likely permeable scaffolds. Topological polar surface area values differentiated ligands in terms of expected permeability and potential for transmembrane transport. L01, L02, L05, L06, L09, L12, and L14 exhibited relatively low to moderate tPSA (≈12–51 $Å^2$), which is consistent with favorable membrane permeability and oral uptake. In contrast, L07, L08, and L10 showed very high tPSA (≈107–148 $Å^2$), suggesting reduced passive diffusion and possible dependence on carrier-mediated transport or limited systemic exposure if used as systemic fungicides. The high polar surface area of these ligands may, however, enhance selectivity or local retention in certain biological compartments. The number of rotatable bonds and bioavailability scores further refined the assessment of fungicide likeness. L01, L02, L05, L07, L08, L10, and L12 possessed a modest to moderate degree of flexibility (0–6 rotatable bonds), which favors a balance between conformational adaptability and entropic cost upon binding. By contrast, L06 and L09 were highly flexible (14–16 rotatable bonds), which can reduce oral bioavailability and binding efficiency in vivo despite formal Lipinski compliance. Bioavailability scores clustered around 0.55 for most ligands, with L02 and L09 reaching 0.85, highlighting these two as particularly attractive in terms of predicted systemic exposure. Taken together, the ADME–driven profiling highlights L02, L05, and L12 as the most pharmaceutically favorable candidates, combining no Lipinski violations, moderate molecular weight and tPSA, limited flexibility, and reasonable bioavailability scores. L01 also appears suitable as a small, highly permeable fragment-like scaffold, whereas L07, L08, L10, L06, L09, and L14 may require structural optimization to reduce excessive polarity or

flexibility while preserving biological activity. The lack of data for L04 and L11 indicates that these ligands remain under-characterized from a pharmacokinetic perspective and should be considered lower-priority until full ADME descriptors are available.

3.5.2 Toxicity

The tabular data presents a thorough analysis of the toxic characteristics related to various ligands, encompassing both aquatic and non-aquatic environments. Using the webtool named admetSAR (http://lmmd.ecust.edu.cn/admetsar2) pharmacokinetics and drug-likeness data were generated [59, 60].

Table 7. **Summary of the aquatic and non-aquatic toxicity properties of the studied ligands.**

In these classification models, higher probability values indicate a greater likelihood of the positive outcome (e.g., absorption, penetration, inhibition, or substrate status), with values typically interpreted as positive predictions when exceeding ~0.5–0.6, depending on the specific endpoint and model calibration. Table 7 summarizes key predicted pharmacokinetic parameters for the ligands. Human intestinal absorption (HIA) probabilities were predominantly high (0.85–1.00 for most compounds, with L01, L02, L04, L06, L09, L11, and L14 approaching or reaching 0.99–1.00), indicating excellent predicted oral bioavailability and efficient gastrointestinal uptake. This trend aligns closely with Caco-2 permeability predictions, a well-validated in vitro proxy for intestinal epithelial transport. Ligands L01, L02, L04, L06, and L09 exhibited particularly strong permeability (0.74–0.99), supporting their potential for effective passive transcellular absorption. Blood-brain barrier (BBB) penetration probabilities varied substantially across the series. Several ligands (L01, L04, L05, L06, L09, L11, and L14) displayed high scores (>0.90), suggesting facile CNS entry. Such profiles may confer advantages for therapeutic applications requiring central activity (e.g., neurological disorders) but raise concerns for peripheral-targeted agents, where unintended CNS exposure could precipitate off-target effects or toxicity. Interactions with P-glycoprotein (P-gp), a major efflux transporter influencing bioavailability and multidrug resistance, revealed widespread moderate-to-high inhibition potential (0.72–0.99 across the set). Notably, L01, L02, L03, L07, and L10 showed strong P-gp inhibition probabilities (≥0.97), which could enhance cellular retention and intracellular accumulation of co-administered drugs or the

ligands themselves. However, certain compounds (e.g., L03, L07, L08) were also predicted as P-gp substrates (probabilities 0.65–0.74), potentially leading to efflux-mediated reduced exposure in target tissues. Renal organic cation transporter (OCT) substrate probabilities exhibited considerable diversity (0.36–0.94), implying differential renal clearance mechanisms. Ligands with elevated scores (e.g., L03, L04, L06 ≥0.85) may undergo more efficient active tubular secretion, favoring rapid elimination and potentially lower systemic exposure upon repeated dosing. Subcellular localization predictions pointed to preferential mitochondrial or cytoplasmic accumulation for most ligands (probabilities 0.36–0.74), which could influence interactions with compartment-specific targets or metabolic enzymes. Cytochrome P450 (CYP) metabolism profiles highlighted variable substrate and inhibition liabilities. Several ligands (L04, L05, L06, L09) displayed substantial CYP2C9 substrate probabilities (≥0.80), consistent with expected hepatic Phase I metabolism. Inhibition of CYP1A2 was pronounced in select cases (e.g., L05: 0.88, L09: 0.91, L14: 0.92), raising the possibility of drug–drug interactions via impaired clearance of CYP1A2 substrates.

4. Conclusion

Our integrated multi-scale computational pipeline establishes Rhizophora mucronata-derived ligands particularly L10 as potent, drug-like inhibitors of two critical lung-cancer drivers: the Keap1 Kelch domain (PDB 1X2J) that governs Nrf2-mediated chemoresistance and the YEATS2 YEATS domain (PDB 5XNV) that sustains oncogenic transcription via H3K27ac readout. Density-functional-theory analysis at the B3LYP/6-31G* level identified L10 as the most reactive scaffold (HOMO–LUMO gap 0.36 eV, highest electrophilicity), enabling efficient charge-transfer interactions, while L05 provided complementary stability (gap 8.98 eV). Orthogonal docking with AutoDock Vina and Schrödinger Glide converged on L10 as the top-ranked binder (−7.789 kcal/mol for 1X2J and −5.482 kcal/mol for 5XNV), anchored by eight persistent hydrogen bonds and extensive hydrophobic contacts that precisely occlude the catalytic pockets. One-hundred-nanosecond Desmond simulations under NPT conditions confirmed exceptional dynamic stability (Cα RMSD < 3 Å throughout, minimal RMSF in interaction hotspots, sustained H-bond occupancy >70 %), while SwissADME/pkCSM profiling demonstrated full Lipinski compliance, high oral bioavailability (score 0.55–0.85), and negligible predicted toxicity attributes that collectively surpass many current NSCLC chemotherapeutics. By systematically closing the long-

standing gap between mangrove phytochemical diversity and structure-guided targeting of NSCLC-specific proteases/epigenetic readers, this work not only nominates L10 as an immediately actionable lead for wet-lab synthesis and validation but also delivers a scalable, cost-effective discovery platform that can be extended to other underexplored marine natural-product reservoirs, thereby accelerating the development of low-toxicity, nature-inspired precision therapeutics against one of the deadliest human malignancies.

Abbreviations

MD: Molecular dynamics; ADMET: Absorption, Distribution, Mechanism, Excretion, and Toxicity; PDB: Protein Data Bank; RMSD: Root mean square deviation; RMSF: Root mean square fluctuation.

Disclosures Peer-review: Externally peer-reviewed.

Conflict of Interest: None declared.

Ethics approval and consent to participate

Not applicable.

Consent for publication

Not applicable.

Availability of data and materials

Not applicable.

Competing interests

The authors declare that they have no competing interests.

Funding

Not applicable.

Authors' contributions

Z.A.A.M., B.S.A.A: Performed the conceptual study, and data analysis. M.N., M.M.H.: did the simulation, data analysis, and draft writing. A.K.: performed the conceptual study, supervision and administrative managing; F. T.A.A., H.I.M.A.D.: revised the draft writing and funding acquisition; F.R., M.F.A.H.K.: did the draft writing and data analysis; NS.K. AK, and S.A.M.A-Y revised and review the manuscript.

Acknowledgements

The authors express their gratitude to the IUBAT Innovation and Entrepreneurship Center, IUBAT—International University of Business Agriculture and Technology, 4 Embankment Drive Road, Sector 10, Uttara Model Town, Dhaka 1230, Bangladesh, for providing technical support that contributed to the progress of this research.

Supplementary Information

Not applicable but attached supplementary file.

References

[1] F. Bray *et al.*, "Global cancer statistics 2022: GLOBOCAN estimates of incidence and mortality worldwide for 36 cancers in 185 countries," *CA: a cancer journal for clinicians,* vol. 74, no. 3, pp. 229-263, 2024.

[2] C. Li *et al.*, "Global burden and trends of lung cancer incidence and mortality," vol. 136, no. 13, pp. 1583-1590, 2023.

[3] W. C. R. Fund. Lung cancer statistics [Online].

[4] A. C. Society, "Cancer Facts & Figureures 2025," 2025, Available: https://www.cancer.org/content/dam/cancer-org/research/cancer-facts-and-statistics/annual-cancer-facts-and-Figureures/2025/2025-cancer-facts-and-Figureures-acs.pdf.

[5] J. Malhotra, M. Malvezzi, E. Negri, C. La Vecchia, and P. J. E. R. J. Boffetta, "Risk factors for lung cancer worldwide," vol. 48, no. 3, pp. 889-902, 2016.

[6] A. Leiter, R. R. Veluswamy, and J. P. J. N. r. C. o. Wisnivesky, "The global burden of lung cancer: current status and future trends," vol. 20, no. 9, pp. 624-639, 2023.

[7] F. Islami, L. A. Torre, and A. J. T. l. c. r. Jemal, "Global trends of lung cancer mortality and smoking prevalence," vol. 4, no. 4, p. 327, 2015.

[8] J. M. J. T. s. c. Samet, "Tobacco smoking: the leading cause of preventable disease worldwide," vol. 23, no. 2, pp. 103-112, 2013.

[9] L. Corrales, R. Rosell, A. F. Cardona, C. Martin, Z. L. Zatarain-Barrón, and O. J. C. r. i. o. h. Arrieta, "Lung cancer in never smokers: The role of different risk factors other than tobacco smoking," vol. 148, p. 102895, 2020.

[10] J. Kusel, B. Timm, and I. J. T. i. d. Lockhart, "The impact of smoking in the home on the health outcomes of non-smoker occupants in the UK," vol. 11, no. 1, p. 3, 2013.

[11] S. P. Eckel *et al.*, "Air pollution affects lung cancer survival," vol. 71, no. 10, pp. 891-898, 2016.

[12] C. D. Berg *et al.*, "Air pollution and lung cancer: a review by international association for the study of lung cancer early detection and screening committee," vol. 18, no. 10, pp. 1277-1289, 2023.

[13] M. Lorenzo-Gonzalez, M. Torres-Duran, R. Barbosa-Lorenzo, M. Provencio-Pulla, J. M. Barros-Dios, and A. J. E. r. o. r. m. Ruano-Ravina, "Radon exposure: a major cause of lung cancer," vol. 13, no. 9, pp. 839-850, 2019.

[14] G. Deshmukh, H. Sawarkar, and K. J. E. Biyani, "Review on lung cancer with emphasis on current treatment," vol. 4, no. 4, 2024.

[15] D. Petrova *et al.*, "The patient, diagnostic, and treatment intervals in adult patients with cancer from high-and lower-income countries: A systematic review and meta-analysis," vol. 19, no. 10, p. e1004110, 2022.

[16] M. H. Ebell, T. N. Thai, and K. J. J. P. h. r. Royalty, "Cancer screening recommendations: an international comparison of high income countries," vol. 39, no. 1, p. 7, 2018.

[17] A. Shankar *et al.*, "Feasibility of lung cancer screening in developing countries: challenges, opportunities and way forward," vol. 8, no. Suppl 1, p. S106, 2019.

[18] B. Lubuzo, T. Ginindza, and K. J. T. P. A. M. J. Hlongwana, "The barriers to initiating lung cancer care in low-and middle-income countries," vol. 35, p. 38, 2020.

[19] D. J. Newman and G. M. Cragg, "Natural products as sources of new drugs over the nearly four decades from 01/1981 to 09/2019," *Journal of natural products,* vol. 83, no. 3, pp. 770-803, 2020.

[20] A. M. Youssef, D. A. Maaty, and Y. M. Al-Saraireh, "Phytochemistry and anticancer effects of mangrove (Rhizophora mucronata Lam.) leaves and stems extract against different cancer cell lines," *Pharmaceuticals,* vol. 16, no. 1, p. 4, 2022.

[21] N. Duma, R. Santana-Davila, and J. R. Molina, "Non–small cell lung cancer: epidemiology, screening, diagnosis, and treatment," in *Mayo Clinic Proceedings,* 2019, vol. 94, no. 8, pp. 1623-1640: Elsevier.

[22] H. Sung *et al.*, "Global cancer statistics 2020: GLOBOCAN estimates of incidence and mortality worldwide for 36 cancers in 185 countries," *CA: a cancer journal for clinicians,* vol. 71, no. 3, pp. 209-249, 2021.

[23] S.-R. Yang, A. M. Schultheis, H. Yu, D. Mandelker, M. Ladanyi, and R. Buettner, "Precision medicine in non-small cell lung cancer: Current applications and future directions," in *Seminars in cancer biology*, 2022, vol. 84, pp. 184-198: Elsevier.

[24] M. C. S. Khatun, M. A. Muhit, M. M. H. Maruf, M. A. Iqbal, and S. A. Rahman, "Pharmacological evaluation of mangrove plant Rhizophora mucronata (Lam.) grown in the coastal area of Sundarbans," *PLoS One*, vol. 21, no. 1, p. e0340646, 2026.

[25] S. Nabeelah Bibi *et al.*, "Ethnopharmacology, phytochemistry, and global distribution of mangroves―A comprehensive review," *Marine drugs,* vol. 17, no. 4, p. 231, 2019.

[26] K. Chakraborty, "Chemistry and biological activities of rhizophora mucronata lam," in *Bioactive Compounds in Mangroves and their Associates*: Springer, 2025, pp. 1-26.

[27] N. B. Sadeer *et al.*, "Untargeted metabolomic profiling, multivariate analysis and biological evaluation of the true mangrove (Rhizophora mucronata Lam.)," vol. 8, no. 10, p. 489, 2019.

[28] N. Suganthy and K. J. P. b. Pandima Devi, "In vitro antioxidant and anti-cholinesterase activities of Rhizophora mucronata," vol. 54, no. 1, pp. 118-129, 2016.

[29] X. Li, D. Zhang, M. Hannink, and L. J. J. J. o. B. C. Beamer, "Crystal structure of the Kelch domain of human Keap1," vol. 279, no. 52, pp. 54750-54758, 2004.

[30] B. Padmanabhan *et al.*, "Structural basis for defects of Keap1 activity provoked by its point mutations in lung cancer," vol. 21, no. 5, pp. 689-700, 2006.

[31] B. Padmanabhan *et al.*, "Structural basis for defects of Keap1 activity provoked by its point mutations in lung cancer," *Molecular cell,* vol. 21, no. 5, pp. 689-700, 2006.

[32] W. Mi *et al.*, "YEATS2 links histone acetylation to tumorigenesis of non-small cell lung cancer," vol. 8, no. 1, p. 1088, 2017.

[33] W. Mi *et al.*, "YEATS2 links histone acetylation to tumorigenesis of non-small cell lung cancer," *Nature communications,* vol. 8, no. 1, p. 1088, 2017.

[34] W. Kohn, A. D. Becke, and R. G. Parr, "Density functional theory of electronic structure," *The journal of physical chemistry,* vol. 100, no. 31, pp. 12974-12980, 1996.

[35] R. Parr and W. Yang, "Horizons of quantum chemistry," *Horizons of Quantum Chemistry. Springer. https://doi. org/10.1007/978-94-009-9027-2,* 1980.

[36] R. G. Parr and W. Yang, "Density functional approach to the frontier-electron theory of chemical reactivity," *Journal of the American Chemical Society,* vol. 106, no. 14, pp. 4049-4050, 1984.

[37] V. Spassov and L. Yan, "Accelrys Software Inc., discovery studio modeling environment, release 4.0," *Proteins Struct Funct Bioinforma,* vol. 81, pp. 704-714, 2013.

[38] M. Sandor, R. Kiss, and G. r. M. Keserű, "Virtual fragment docking by Glide: a validation study on 190 protein− fragment complexes," *Journal of Chemical Information and Modeling,* vol. 50, no. 6, pp. 1165-1172, 2010.

[39] K. J. Bowers *et al.*, "Scalable algorithms for molecular dynamics simulations on commodity clusters," in *Proceedings of the 2006 ACM/IEEE Conference on Supercomputing*, 2006, pp. 84-es.

[40] A. Daina, O. Michielin, and V. Zoete, "SwissADME: a free web tool to evaluate pharmacokinetics, drug-likeness and medicinal chemistry friendliness of small molecules," *Scientific reports,* vol. 7, no. 1, p. 42717, 2017.

[41] K. Tsaioun, B. J. Blaauboer, and T. Hartung, "Evidence-based absorption, distribution, metabolism, excretion (ADME) and its interplay with alternative toxicity methods," *ALTEX-Alternatives to animal experimentation*, vol. 33, no. 4, pp. 343-358, 2016.

[42] B. Chandrasekaran, S. N. Abed, O. Al-Attraqchi, K. Kuche, and R. K. Tekade, "Computer-aided prediction of pharmacokinetic (ADMET) properties," in *Dosage form design parameters*: Elsevier, 2018, pp. 731-755.

[43] D. E. Pires, T. L. Blundell, and D. B. Ascher, "pkCSM: predicting small-molecule pharmacokinetic and toxicity properties using graph-based signatures," *Journal of medicinal chemistry,* vol. 58, no. 9, pp. 4066-4072, 2015.

[44] J. López, C. Anitescu, N. Valizadeh, T. Rabczuk, and N. Alajlan, "Structural shape optimization using Bézier triangles and a CAD-compatible boundary representation," *Engineering with Computers,* vol. 36, pp. 1657-1672, 2020.

[45] A. Kumer, B. Ahmed, M. Sharif, and A. Al-Mamun, "A theoretical study of aniline and nitrobenzene by computational overview," *Asian Journal of Physical and Chemical Sciences,* vol. 4, no. 2, pp. 1-12, 2017.

[46] A. Kumer, S. Paul, M. N. Sarker, and M. J. Islam, "The prediction of thermo physical, vibrational spectroscopy, chemical reactivity, biological properties of morpholinium borate, phosphate, chloride and bromide ionic liquid: a DFT study," *International Journal of New Chemistry*, vol. 6, no. 4, pp. 236-253, 2019.

[47] A. KUMER, M. N. Sarker, and P. Sunanda, "The theoretical investigation of HOMO, LUMO, thermophysical properties and QSAR study of some aromatic carboxylic acids using HyperChem programming," *International Journal of Chemistry and Technology*, vol. 3, no. 1, pp. 26-37, 2019.

[48] A. Kumer, N. Sarker, S. Paul, and A. Zannat, "The theoretical prediction of thermophysical properties, HOMO, LUMO, QSAR and biological indics of cannabinoids (CBD) and tetrahhdrocannabinol (THC) by computational chemistry," *Advanced Journal of Chemistry-Section A*, vol. 2, no. 3, pp. 190-202, 2019.

[49] M. M. Hoque, A. Kumer, M. S. Hussen, and M. W. Khan, "Theoretical Evaluation of 5, 6-Diaroylisoindoline-1, 3-dione as Potential Carcinogenic Kinase PAK1 Inhibitor: DFT Calculation, Molecular Docking Study and ADMET Prediction," *International Journal of Advanced Biological and Biomedical Research*, vol. 9, no. 1, pp. 77-104, 2021.

[50] M. J. Islam, M. N. Sarker, A. Kumer, and S. Paul, "The Evaluation and Comparison of Thermo-Physical, Chemical and Biological Properties of Palladium (II) Complexes on Binuclear Amine Ligands with Different Anions by DFT Study," *International journal of Advanced Biological and Biomedical Research*, vol. 7, no. 4, pp. 315-334, 2019.

[51] A. Kumer *et al.*, "MODIFIED D-GLUCOFURANOSE COMPUTATIONALLY SCREENING FOR INHIBITOR OF BREAST CANCER AND TRIPLE BREAST CANCER: CHEMICAL DESCRIPTOR, MOLECULAR DOCKING, MOLECULAR DYNAMICS AND QSAR," *Journal of the Chilean Chemical Society*, vol. 67, no. 3, pp. 5623-5635, 2022.

[52] A. Kumer *et al.*, "Investigation of the New Inhibitors by Sulfadiazine and Modified Derivatives of α-D-glucopyranoside for White Spot Syndrome Virus Disease of Shrimp by In Silico: Quantum Calculations, Molecular Docking, ADMET and Molecular Dynamics Study," *Molecules*, vol. 27, no. 12, p. 3694, 2022.

[53] A. Kumer, M. Jahidul, and S. Paul, "Effect of external electric field and temperature on entropy, heat of capacity, and chemical reactivity with QSAR study of morphonium

chloride and nitrous ionic liquids crystal using DFT," *Chemical Methodologies*, vol. 4, pp. 595-604, 2020.

[54] K. Cheng, Q.-Z. Zheng, Y. Qian, L. Shi, J. Zhao, and H.-L. Zhu, "Synthesis, antibacterial activities and molecular docking studies of peptide and Schiff bases as targeted antibiotics," *Bioorganic & medicinal chemistry*, vol. 17, no. 23, pp. 7861-7871, 2009.

[55] T. Hermann and E. Westhof, "Docking of cationic antibiotics to negatively charged pockets in RNA folds," *Journal of medicinal chemistry*, vol. 42, no. 7, pp. 1250-1261, 1999.

[56] Y. Yang *et al.*, "Efficient exploration of chemical space with docking and deep learning," *Journal of Chemical Theory and Computation*, vol. 17, no. 11, pp. 7106-7119, 2021.

[57] R. A. Friesner *et al.*, "Extra precision glide: Docking and scoring incorporating a model of hydrophobic enclosure for protein− ligand complexes," *Journal of medicinal chemistry*, vol. 49, no. 21, pp. 6177-6196, 2006.

[58] R. A. Friesner *et al.*, "Glide: a new approach for rapid, accurate docking and scoring. 1. Method and assessment of docking accuracy," *Journal of medicinal chemistry*, vol. 47, no. 7, pp. 1739-1749, 2004.

[59] H. Yang *et al.*, "admetSAR 2.0: web-service for prediction and optimization of chemical ADMET properties," *Bioinformatics*, vol. 35, no. 6, pp. 1067-1069, 2019.

[60] F. Cheng *et al.*, "admetSAR: a comprehensive source and free tool for assessment of chemical ADMET properties," ed: ACS Publications, 2012.

Click here to access/download
Supporting Information
Table and Supplementary.docx
