# Supplementary material for: Investigating the anticancer potential of phytochemicals from the mangrove Rhizophora mucronata as inhibitors of lung cancer-associated proteins: Insights from multiscale computational and in silico analyses
Source: PLoS One. 2026 Oct 7;21(10):e0359525. doi: 10.1371/journal.pone.0359525 (PMC13645060; doi:10.1371/journal.pone.0359525)
Supplement: Supplementary file 10007 [file pone.0359525.s011.pdf]

PLOS One

Investigating the Anticancer Potential of Phytochemicals from the Mangrove Rhizophora mucronata as Inhibitors of Lung Cancer-Associated Proteins: Insights from Multiscale Computational and In Silico Analyses

--Manuscript Draft--

**Manuscript Number:**	PONE-D-26-14113R2
**Article Type:**	Research Article
**Full Title:**	Investigating the Anticancer Potential of Phytochemicals from the Mangrove Rhizophora mucronata as Inhibitors of Lung Cancer-Associated Proteins: Insights from Multiscale Computational and In Silico Analyses
**Short Title:**	Multi-scale Computational and In silico Analysis Study for Lung Cancer Protein Inhibitors
**Corresponding Author:**	Ajoy Kumer, MPhil<br>IUBAT: International University of Business Agriculture and Technology<br>Naogaon, Naogaon zila BANGLADESH
**Keywords:**	DFT; Docking; Molecular Dynamic; ADMET; Binding affinity; Rizhophora muarnata; Lung cancer
**Abstract:**	Lung cancer remains the leading cause of cancer-related mortality worldwide, with a five-year survival rate of approximately 20–25% despite advances in targeted therapy and immunotherapy. There is a continuous need for novel, low-toxicity compounds capable of modulating key oncogenic pathways in non-small cell lung cancer (NSCLC). Mangrove ecosystems are rich reservoirs of bioactive phytochemicals, yet the inhibitory potential of compounds from Rhizophora mucronata against validated lung cancer-associated proteins has not been systematically explored using multi-scale computational approaches. This study employed an integrated computational pipeline to evaluate 14 phytochemicals from R. mucronata. Density Functional Theory (DFT) calculations at the B3LYP/6-31G* level were performed to characterize electronic properties of the ligands. Molecular docking studies were conducted using both AutoDock Vina (PyRx) and Schrödinger Glide against the Keap1 Kelch domain (PDB 1X2J) and the YEATS2 YEATS domain (PDB 5XNV). Top-ranked complexes were subjected to 100-ns molecular dynamics (MD) simulations in Desmond, and pharmacokinetic and toxicity profiles were predicted using SwissADME and pkCSM. Among the screened compounds, ligand L10 (cyclohexane-1,2,3,4,5,6-hexaol) exhibited the smallest HOMO–LUMO energy gap (0.36 eV), favorable binding affinities (Glide score of –7.789 kcal/mol against 1X2J), multiple hydrogen-bonding interactions, and stable complex behavior during MD simulations (Cα RMSD < 3 Å). Several other ligands (L02, L05, and L07) also displayed promising profiles. Most compounds complied with Lipinski's rule of five and showed acceptable predicted ADMET properties. These preliminary findings suggest that Rhizophora mucronate -derived phytochemicals, particularly L10, are promising candidates for further experimental evaluation as modulators of Keap1 and YEATS2 in lung cancer. This study provides a computational framework for prioritizing natural product leads from mangrove species for subsequent in vitro and in vivo validation.
**Order of Authors:**	Zena Abdul-Ameer Mahdi
	Batool Shakir Abed Almjalaw
	Mollah Naimuzzaman
	Md Mahabub Hasan
	Ajoy Kumer, MPhil
	Farah Tareq Al-Alaq
	Hussein O.M. Al-Dahmoshi
	Faizur Rahman
	Md. Fayez Al-Hakim Khan

Powered by Editorial Manager® and ProduXion Manager® from Aries Systems Corporation

	Noor S.K. Al-Khafaji
	Sarah Alwan Malik Al-Yasiri
**Response to Reviewers:**	Response to the Academic Editor and Reviewers<br>Manuscript ID: PONE-D-26-14113R1<br>Title: Investigating the Anticancer Potential of Phytochemicals from the Mangrove Rhizophora mucronata as Inhibitors of Lung Cancer-Associated Proteins: Insights from Multiscale Computational and In Silico Analyses<br>Dear Academic Editor,<br>We sincerely thank you and the reviewers for the careful evaluation of our revised manuscript and for the constructive comments. We greatly appreciate the time and effort devoted to assessing our work. The comments were valuable in identifying several remaining issues related to manuscript organization, scientific terminology, methodological clarity, data presentation, and interpretation.<br>We have carefully revised the manuscript accordingly. In particular, we consolidated the Introduction, improved the academic tone, standardized the biological nomenclature, clarified the independent AutoDock Vina and Schrödinger Glide workflows, completed the molecular dynamics methodology, corrected the DFT equations and descriptors, removed duplicated and inappropriate text, reconciled the docking results obtained from the two docking platforms, corrected the protein–ligand interaction discussion, and revised the benchmarking section to avoid inappropriate comparison of docking scores with logP.<br>Our point-by-point responses are provided below. The relevant section of the revised manuscript is identified after each response.<br>Reviewer #1<br>We sincerely thank Reviewer #1 for confirming that the previous concerns were adequately addressed and for recommending the manuscript for publication. We greatly appreciate the reviewer's positive assessment of the revised work.<br>Reviewer #2<br>Comment: Redundancy in the Introduction<br>The reviewer noted that epidemiological information, survival statistics, and the limitations of existing therapies were repeated across the opening paragraphs.<br>Response:<br>Thank you for this observation. We agree that the earlier version contained unnecessary repetition. The Introduction has been substantially condensed and reorganized. Repeated information regarding global lung cancer mortality, five-year survival, major risk factors, and limitations of existing treatments has been consolidated. The revised Introduction now progresses more directly from the clinical burden of lung cancer to the rationale for investigating natural-product-derived compounds and finally to the selection of Keap1 and YEATS2.<br>Location of correction: Section 1, Introduction, opening paragraphs.<br>Comment: Colloquial and non-academic expressions<br>The reviewer identified several expressions that were too conversational for a scientific manuscript.<br>Response:<br>We agree and have revised the manuscript extensively to improve its academic tone. Conversational expressions and informal analogies were removed and replaced with concise scientific language. Statements such as "lets us compare," "grows silently," and other informal descriptions were rewritten using formal biomedical terminology. Similar revisions were made throughout the Methods, Results and Discussion sections.<br>Location of correction: Throughout the manuscript, particularly Sections 1, 2 and 3.<br>Comment: Biological nomenclature of Rhizophora mucronata<br>The reviewer requested that the genus and species name be italicized throughout.<br>Response:<br>Thank you. The scientific name has been standardized throughout the revised manuscript. The full name Rhizophora mucronata is used at first mention where appropriate, followed by the abbreviated form R. mucronata. Both forms are italicized consistently.<br>Location of correction: Title, Abstract, Keywords, Introduction, Methods, Results and Discussion, Conclusion, and relevant figure/table captions.<br>Comment: Semantic error in the phrase "This study recovers a critical knowledge gap"<br>Response:<br>We agree. The inappropriate expression has been removed. The revised manuscript now states that the study "addresses a critical knowledge gap" rather than "recovers" a

gap.
Location of correction: Section 1, Introduction, final part of the study-rationale paragraph.
Comment: Grammatical sentence fragments in the methodology
The reviewer identified incomplete expressions such as “Used the DMol³ module…,” “Used the Keap1…,” and “Molecular docking was did…”.
Response:
Thank you. These sentence fragments have been rewritten using complete formal scientific sentences and appropriate passive constructions. The DFT, receptor-preparation, AutoDock Vina, and Glide procedures are now described as complete methodological workflows.
Location of correction: Sections 2.1, Optimization and Ligand Preparation; 2.2, Protein Preparation and Collection; and 2.3, Molecular Docking and Visualization.
Comment: Contradiction concerning Discovery Studio and Schrödinger receptor preparation
The reviewer questioned whether receptors prepared only in Discovery Studio were directly used for Glide docking.
Response:
We thank the reviewer for highlighting this important methodological point. The receptors for AutoDock Vina and Glide were prepared independently using platform-specific workflows.
For AutoDock Vina, receptor preparation was performed using Discovery Studio, including removal of non-essential crystallographic molecules and energy minimization.
For Glide docking, the receptor structures were independently processed in Schrödinger Maestro using the Protein Preparation Wizard. This workflow included assignment of bond orders, addition of hydrogen atoms, optimization of the hydrogen-bonding network, removal of unnecessary water molecules, and restrained energy minimization. The resulting structures were subsequently used for receptor-grid generation and Glide Standard Precision docking.
This distinction is now explicitly reported in the manuscript.
Location of correction: Section 2.2, Protein Preparation and Collection, and Section 2.3.2, Molecular Docking by Glide from Schrödinger Suite.
Comment: Duplicate Table 2 heading and incomplete Molecular Dynamics methodology
Response:
Thank you. The duplicated Table 2 heading has been removed.
The Molecular Dynamics methodology has also been completed and expanded. The revised section now specifies the selected complexes, 100-ns simulation duration, Desmond version, OPLS-AA force field, TIP3P solvent model, orthorhombic periodic box with a 10 Å buffer, system neutralization, energy minimization, NVT and NPT equilibration, simulation temperature and pressure, thermostat and barostat, integration time step, SHAKE constraints, and the trajectory parameters evaluated.
Location of correction: Table 2 and Section 2.4, Molecular Dynamics.
Comment: Poor formatting of Equations 4–8
Response:
We agree. Equations 1–8 have been reformatted and standardized. The notation for HOMO–LUMO energy gap, ionization potential, electron affinity, electronegativity, chemical potential, hardness, softness, and electrophilicity has been corrected and presented consistently.
The equations are now defined as:
$\Delta Egap = ELUMO - EHOMO$
$I = -EHOMO$
$A = -ELUMO$
$\chi = (I + A)/2$
$\mu = -(I + A)/2$
$\eta = (I - A)/2$
$S = 1/\eta$
$\omega = \mu^2/(2\eta)$
Location of correction: Section 2.1, Optimization and Ligand Preparation, Equations 1–8.
Comment: Sign and variable inconsistencies in the DFT descriptors
The reviewer noted inconsistencies involving ionization potential, electron affinity, hardness, softness, electrophilicity, and the HOMO/LUMO values.
Response:

Powered by Editorial Manager® and ProduXion Manager® from Aries Systems Corporation

We appreciate this important observation. The DFT-derived descriptors were rechecked against the original HOMO and LUMO outputs and recalculated using the equations defined in the Methods. Table 3 has been revised accordingly so that the reported ionization potential, electron affinity, hardness, softness, chemical potential, electronegativity, and electrophilicity values are internally consistent with the underlying frontier orbital energies.
We also revised the accompanying discussion to avoid interpreting the HOMO–LUMO gap or related DFT descriptors as direct indicators of anticancer activity. These quantities are now described as supportive physicochemical descriptors of intrinsic electronic properties.
Location of correction: Section 3.1.2, HOMO, LUMO, and Chemical Reactivity Descriptors, and Table 3.
Comment: Duplicate paragraphs in the DFT section
Response:
Thank you. The duplicated text has been removed. The DFT discussion has been consolidated into a single concise interpretation emphasizing electronic stability and reactivity while avoiding overinterpretation of these descriptors as predictors of biological activity.
Location of correction: Section 3.1.2, immediately following Table 3.
Comment: Use of the term “fungicidal ligands”
Response:
We agree that this was an inappropriate copy-paste artifact unrelated to the present anticancer study. All references to “fungicidal ligands,” “fungicidal candidates,” and related terminology have been removed. The compounds are now consistently described as phytochemicals, screened ligands, or candidate anticancer compounds as appropriate.
Location of correction: Sections 3.1.3 and 3.2.1.
Comment: Section 3.1.4 was truncated
Response:
Thank you. Section 3.1.4 has been completed and revised so that the discussion of the electrostatic potential/FMO maps is presented as a complete paragraph and appropriately linked to the corresponding figures.
Location of correction: Section 3.1.4, Electrostatic Potential Map for Frontier Molecular Orbital.
Comment: Glide discussion duplicated the AutoDock Vina discussion
Response:
We agree. The duplicated AutoDock Vina material has been removed from the Glide subsection. Section 3.2.5 now discusses only the Glide-selected 1X2J–L10 and 5XNV–L02 complexes and their corresponding interaction patterns.
Location of correction: Section 3.2.5, Protein–Ligand Interaction from Glide Docking.
Comment: Different lead ligands were identified by PyRx and Glide without explanation
Response:
Thank you for raising this important issue. We have now explicitly explained why different ligand rankings were obtained from the two docking platforms.
AutoDock Vina identified L11 and L14 as the highest-ranked ligands against 1X2J and 5XNV, respectively, whereas Glide identified L10 and L02 as the highest-ranked ligands against the corresponding targets. These differences are expected because AutoDock Vina and Glide use different receptor-preparation procedures, conformational-search algorithms, scoring functions, and treatments of intermolecular interactions.
The two docking approaches are therefore interpreted as complementary rather than numerically interchangeable. To maintain methodological consistency, the Glide-ranked complexes 1X2J–L10 and 5XNV–L02 were selected for subsequent 100-ns molecular dynamics simulations.
Location of correction: Section 3.2.2, immediately following Table 5, and Section 3.3.1, RMSD.
Comment: Contradictory description of L12 hydrogen bonding
The reviewer noted that L12 was initially described as forming no hydrogen bonds but was subsequently described as forming several hydrogen bonds.
Response:
We agree and have corrected this inconsistency. According to the interaction data reported in Table 4, L12 against 5XNV forms two hydrogen bonds and four hydrophobic contacts, with a PyRx docking score of −6.0 kcal/mol. L12 is therefore now described consistently using these interaction values.

Powered by Editorial Manager® and ProduXion Manager® from Aries Systems Corporation

	We also clarify that L12 is shown as a representative YEATS2-binding pose and was not the highest-ranked PyRx ligand for 5XNV; L14 was the highest-ranked ligand for this target in the AutoDock Vina analysis.<br>Location of correction: Section 3.2.4, Protein–Ligand Interaction by PyRx Docking, and Figure 6 discussion.<br>Comment: Conceptual error involving docking energy and octanol–water partition coefficient<br>Response:<br>We fully agree with the reviewer. The previous wording incorrectly associated docking energies expressed in kcal/mol with the octanol–water partition coefficient.<br>The benchmarking section has been revised to clearly distinguish docking scores from logP. Docking scores are software-dependent scoring quantities used for ranking predicted binding poses, whereas logP is a dimensionless measure of molecular lipophilicity. No direct equivalence between these quantities is now implied.<br>Reported Keap1 and YEATS-domain inhibitors are used primarily as qualitative structural and pharmacological references, and we explicitly state that docking scores obtained using different protocols should not be interpreted as experimentally measured binding free energies.<br>Location of correction: Section 3.2.6, Benchmarking with Known Inhibitors.<br>Reviewer #3<br>Comment 1: The rationale for selecting the protein targets was insufficiently summarized<br>Response:<br>We thank the reviewer for this important suggestion. The Introduction has been expanded to provide a concise mechanistic rationale for selecting Keap1 and YEATS2.<br>Keap1 was selected because it is a principal negative regulator of Nrf2, and disruption of the Keap1–Nrf2 pathway is frequently associated with NSCLC, enhanced antioxidant defense, metabolic adaptation, cell survival, and treatment resistance.<br>YEATS2 was selected because it functions as an epigenetic reader of H3K27 acetylation and has been associated with oncogenic transcriptional regulation, proliferation, and survival in NSCLC.<br>The revised manuscript therefore presents Keap1 and YEATS2 as complementary targets representing redox-regulatory and epigenetic mechanisms relevant to NSCLC pathogenesis.<br>Location of correction: Section 1, Introduction, final two paragraphs.<br>Comment 9: The locations of the revisions were not specified<br>Response:<br>We apologize for this omission in the previous response. In the present rebuttal, the location of each substantive revision has been explicitly identified by manuscript section and subsection. Major revisions can be found in:<br>Section 1 – Introduction: consolidation of epidemiological information, formalization of academic language, and expanded rationale for Keap1 and YEATS2.<br>Section 2.1 – Optimization and Ligand Preparation: corrected DFT methodology and equations.<br>Section 2.2 – Protein Preparation and Collection: clarified independent AutoDock Vina and Schrödinger Protein Preparation Wizard workflows.<br>Section 2.3 – Molecular Docking: clarified AutoDock Vina and Glide protocols.<br>Section 2.4 – Molecular Dynamics: completed simulation methodology and parameters.<br>Section 3.1.2 – HOMO, LUMO, and Chemical Reactivity Descriptors: corrected DFT descriptors and revised interpretation.<br>Section 3.1.3–3.1.4: removed inappropriate terminology and completed FMO/electrostatic-potential discussion.<br>Sections 3.2.1–3.2.6 – Molecular Docking: reconciled AutoDock Vina and Glide results, corrected interaction counts, clarified ligand prioritization, and revised benchmarking.<br>Section 3.3 – Molecular Dynamics: clarified the rationale for selecting 1X2J–L10 and 5XNV–L02 for simulation.<br>We have also provided both a marked-up manuscript showing all revisions and a clean version with the revisions incorporated.<br>Closing Response<br>We sincerely thank the Academic Editor and all reviewers for their constructive and detailed comments. Their suggestions have substantially improved the clarity, methodological transparency, internal consistency, and scientific interpretation of the manuscript.

Powered by Editorial Manager® and ProduXion Manager® from Aries Systems Corporation

	We have carefully revised the manuscript to ensure that computational predictions are presented conservatively and are not interpreted as direct evidence of biological efficacy. We believe that the revised manuscript now provides a clearer distinction between DFT-derived electronic descriptors, docking scores, molecular dynamics stability, and experimentally measurable biological activity.<br>We hope that the revised manuscript and detailed responses satisfactorily address all remaining concerns, and we respectfully submit the manuscript for further consideration for publication in PLOS ONE.<br>Sincerely,<br>Ajoy Kumer<br>Corresponding Author<br>on behalf of all authors
**Additional Information:**	
**Question**	**Response**
**Competing Interests**<br><br>Use the instructions below to enter a competing interest statement for this submission. On behalf of all authors, disclose any competing interests that could be perceived to bias this work—acknowledging all financial support and any other relevant financial or non-financial competing interests.<br><br>This statement is required for submission and **will appear in the published article** if the submission is accepted. Please make sure it is accurate and that any funding sources listed in your Funding Information later in the submission form are also declared in your Financial Disclosure statement.<br><br>View published research articles from *PLOS ONE* for specific examples.<br><br>**NO authors have competing interests**<br><br>Enter: *The authors have declared that no competing interests exist.*<br><br>**Authors with competing interests**<br><br>Enter competing interest details beginning with this statement:<br><br>*I have read the journal's policy and the authors of this manuscript have the following competing interests: [insert competing interests here]*	The authors have declared that no competing interests exist.

Powered by Editorial Manager® and ProduXion Manager® from Aries Systems Corporation

* typeset	
**Data Availability**<br><br>Authors are required to make all data underlying the findings described fully available, without restriction, and from the time of publication. PLOS allows rare exceptions to address legal and ethical concerns. See the PLOS Data Policy and FAQ for detailed information.<br><br>A Data Availability Statement describing where the data can be found is required at submission. Your answers to this question constitute the Data Availability Statement and **will be published in the article**, if accepted.<br><br>**Important:** Stating 'data available on request from the author' is not sufficient. If your data are only available upon request, select 'No' for the first question and explain your exceptional situation in the text box.<br><br>Do the authors confirm that all data underlying the findings described in their manuscript are fully available without restriction?	Yes - all data are fully available without restriction
Additional data availability information:	Tick here if your circumstances are not covered by the questions above and you need the journal's help to make your data available.
**Financial Disclosure**<br><br>Enter a financial disclosure statement that describes the sources of funding for the work included in this submission. Review the submission guidelines for detailed requirements. View published research articles from *PLOS ONE* for specific examples.<br><br>This statement is required for submission and **will appear in the published article** if the submission is accepted. Please make sure it is accurate.	The author(s) received no specific funding for this work.

**Funded studies**<br>Enter a statement with the following details:<br>• Initials of the authors who received each award<br>• Grant numbers awarded to each author<br>• The full name of each funder<br>• URL of each funder website<br>• Did the sponsors or funders play any role in the study design, data collection and analysis, decision to publish, or preparation of the manuscript?<br><br>Did you receive funding for this work?	
**Ethics Statement**<br><br>Enter an ethics statement for this submission. This statement is required if the study involved:<br><br>• Human participants<br>• Human specimens or tissue<br>• Vertebrate animals or cephalopods<br>• Vertebrate embryos or tissues<br>• Field research<br><br>Write "N/A" if the submission does not require an ethics statement.<br><br>General guidance is provided below. Consult the submission guidelines for detailed instructions. **Make sure that all information entered here is included in the Methods section of the manuscript.**	N/A

**Format for specific study types**<br><br>**Human Subject Research (involving human participants and/or tissue)**<br>• Give the name of the institutional review board or ethics committee that approved the study<br>• Include the approval number and/or a statement indicating approval of this research<br>• Indicate the form of consent obtained (written/oral) or the reason that consent was not obtained (e.g. the data were analyzed anonymously)<br><br>**Animal Research (involving vertebrate animals, embryos or tissues)**<br>• Provide the name of the Institutional Animal Care and Use Committee (IACUC) or other relevant ethics board that reviewed the study protocol, and indicate whether they approved this research or granted a formal waiver of ethical approval<br>• Include an approval number if one was obtained<br>• If the study involved *non-human primates*, add *additional details* about animal welfare and steps taken to ameliorate suffering<br>• If anesthesia, euthanasia, or any kind of animal sacrifice is part of the study, include briefly which substances and/or methods were applied<br><br>**Field Research**<br><br>Include the following details if this study involves the collection of plant, animal, or other materials from a natural setting:<br>• Field permit number<br>• Name of the institution or relevant body that granted permission	
**Data Availability**<br><br>Before publication, Authors are required to make fully available and without restriction all data underlying their findings. Sharing the data underlying your findings increases the visibility, credibility, and impact of your work, while supporting transparency and future scientific discovery. Please see our PLOS Data	All relevant data are within the manuscript and its Supporting Information files.

Powered by Editorial Manager® and ProduXion Manager® from Aries Systems Corporation

Policy page for detailed information on this policy.<br><br>A **Data Availability Statement**, detailing where the data can be accessed, is required at first submission. Insert your Data Availability Statement in the box below.<br><br>Please see the data reporting section of our submission guidelines for instructions on what you need to include in your Data Availability Statement. Examples of acceptable Data Availability Statements are included here for reference:<br>1. The minimal data set [and accompanying code, where generated] is available at [repository name] via [DOI hyperlink]<br>2. No data was generated by this study. The following existing data sources were used: [dataset name] from [dataset location] available via [web address or DOI hyperlink].<br>3. For Study Protocols: No datasets were generated or analysed during the current study. All relevant data from this study will be made available upon study completion.<br><br>*PLOS allows rare exemptions to address legal and ethical concerns. If you have legal or ethical restrictions, please use the box below to detail these in full sentences for the Journal team to consider.*<br><br>This statement will be typeset if the manuscript is accepted for publication.	

Powered by Editorial Manager® and ProduXion Manager® from Aries Systems Corporation

Manuscript

Click here to access/download;Manuscript;Main manuscript_Revised R3.docx

Investigating the Anticancer Potential of Phytochemicals from the Mangrove *Rhizophora mucronata* as Inhibitors of Lung Cancer-Associated Proteins: Insights from Multiscale Computational and In Silico Analyses

Zena Abdul-Ameer Mahdi[1], Batool Shakir Abed Almjalaw[2], Mollah Naimuzzaman[3], Md Mahabub Hasan[4], Ajoy Kumer*[5,6], Farah Tareq Al-Alaq[7], Hussein O.M. Al-Dahmoshi[8], Faizur Rahman[4], Md. Fayez Al-Hakim Khan[4], Noor S.K. Al-Khafaji[9], and Sarah Alwan Malik Al-Yasiri[10]

[1]Department of medical physics, college of Applied Medical Sciences, University of Kerbala, Karbala 56001, Iraq., email: Zena.a.mahdy@uokerbala.edu.iq

[2] Ministry of Education, General Directorate of Education in Kerbala. Kerbala, Iraq Email: batool_shakir@karbala.edu.iq

[3]Department of Agroforestry and Environmental Science, Sylhet Agricultural University, Sylhet-3100, Bangladesh; Email: mollahnaimuzzaman@gmail.com

[4]College of Agricultural Sciences, IUBAT—International University of Business Agriculture and Technology, 4 Embankment Drive Road, Sector 10 Uttara Model Town, Dhaka 1230, Bangladesh. Email: mahabubhasan33445@gmail.com; FR: faizurrahmanzidni80@gmail.com FHK: fayezalhakim117@gmail.com

[5]Department of General Medicine, Saveetha Institute of Medical and Technical Sciences in Saveetha Medical College and Hospital, Chennai, India, Email: kumarajoy.cu@gmail.com

[6]College of Arts and Sciences, Department of Chemistry, IUBAT—International University of Business Agriculture and Technology, 4-Embankment Drive Road, Sector 10 Uttara Model Town, Dhaka 1230, Bangladesh. Email: kumarajoy.cu@gmail.com

[7]Department of Biology, college of science, University of Babylon, Hilla 51001, Babylon, Iraq, Email: sci.farah.tariq@uobabylon.edu.iq

[8]Department of Biology, college of science, University of Babylon, Hilla 51001, Babylon, Iraq, Email: sci.hussein.oleiwi@uobabylon.edu.iq

[9]Department of Biology, college of science, University of Babylon, Hilla 51001, Babylon, Iraq, Email: sci.noor.salman@uobabylon.edu.iq

[10]Department of environmental health, College of Applied Medical Sciences, University of Kerbala, Karbala 56001, Iraq., email: alyasirsara@gmail.com

Corresponding Authors

Email: kumarajoy.cu@gmail.com

Abstract:

Graphically presentation of abstract:

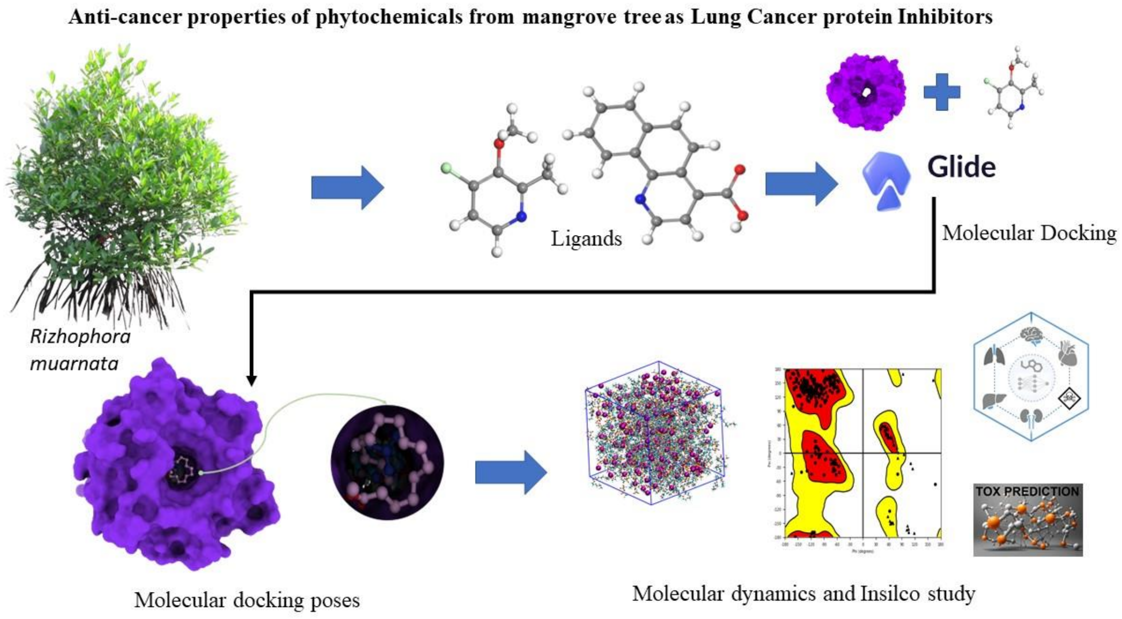

Lung cancer remains the leading cause of cancer-related mortality worldwide, with a five-year survival rate of approximately 20–25% despite advances in targeted therapy and immunotherapy. There is a continuous need for novel, low-toxicity compounds capable of modulating key oncogenic pathways in non-small cell lung cancer (NSCLC). Mangrove ecosystems are rich reservoirs of bioactive phytochemicals, yet the inhibitory potential of compounds from *Rhizophora mucronata* against validated lung cancer-associated proteins has not been systematically explored using multi-scale computational approaches. This study employed an integrated computational pipeline to evaluate 14 phytochemicals from R. mucronata. Density Functional Theory (DFT) calculations at the B3LYP/6-31G* level were performed to characterize electronic properties of the ligands. Molecular docking studies were conducted using both AutoDock Vina (PyRx) and Schrödinger Glide against the Keap1 Kelch domain (PDB 1X2J) and the YEATS2 YEATS domain (PDB 5XNV). Top-ranked complexes were subjected to 100-ns molecular dynamics (MD) simulations in Desmond, and pharmacokinetic and toxicity profiles were predicted using SwissADME and pkCSM. Among the screened compounds, ligand L10 (cyclohexane-1,2,3,4,5,6-hexaol) exhibited the smallest HOMO–LUMO energy gap (0.36 eV), favorable binding affinities

(Glide score of –7.789 kcal/mol against 1X2J), multiple hydrogen-bonding interactions, and stable complex behavior during MD simulations (Cα RMSD < 3 Å). Several other ligands (L02, L05, and L07) also displayed promising profiles. Most compounds complied with Lipinski's rule of five and showed acceptable predicted ADMET properties. These preliminary findings suggest that *Rhizophora mucronate* -derived phytochemicals, particularly L10, are promising candidates for further experimental evaluation as modulators of Keap1 and YEATS2 in lung cancer. This study provides a computational framework for prioritizing natural product leads from mangrove species for subsequent in vitro and in vivo validation.

Keywords: *Rhizophora mucronata*, DFT, molecular docking, molecular dynamics, ADMET, Keap1, YEATS2, NSCLC, natural products, in silico screening

1. Introduction

Lung cancer is one of the top 3 causes of cancer mortality globally, causing nearly 1.8 million deaths a year and representing a significant burden on global health (1). Even with significant progress in targeted therapy and immunotherapy, the overall five-year survival is still modest, mainly due to patients being diagnosed at advanced stages (2). The five-year relative survival rate is still only ~25% for all stages, highlighting a continued unmet need in finding safe and effective therapeutic agents (3). The pathogenesis of lung cancer is affected by diverse modifiable and environmental risk factors (4, 5). Tobacco smoking persists the leading aetiological factor, with passive smoking also playing an important role in disease causation (6-8). These include environmental exposures such as ambient air pollution, household air pollution, and radon, as well as occupational carcinogens and genetic susceptibility (9-11).

Lung cancer is asymptomatic in most cases during the early stages and hence detected at advanced disease state when curative treatment options become scarce (12). Management for current treatment approaches consists of surgical options, radiotherapy, chemotherapy, molecularly-targeted therapy and immunotherapy. While these strategies have improved clinical outcomes, their utility can be limited by treatment-induced toxicity, pan-tumor drug resistance and

recurrence, biomarker-restrictive accessibility as well as high costs (13-16). These drawbacks are driving the identification of less toxic and more efficacious therapeutic candidates.

Natural-product-based phytochemicals have been used as the primary source of structurally diverse scaffolds for anticancer compound discovery (17). Ecosystems of mangroves are notably significant because they constitute, as yet underexplored, a true reservoir of bioactive secondary metabolites: viz. flavonoids, tannins, terpenoids and alkaloids with reported antioxidant, anti-inflammatory and cytotoxic activity (18). Lung cancer is generally divided into small-cell lung cancer and non-small-cell lung cancer (NSCLC). Non-small-lung- cell carcinoma accounts for about 85% of these cases (19).

Traditional medicinal practice uses mangrove species, which are known to contain phytochemicals with a wide range of biological activities including anticancer (20), antibacterial properties (p-glyoproteins in *Rhizophora mucronata* and plant extracts based natural product) antioxidant, anti-inflammatory property. It contains many polyphenols, alkaloids, and other secondary metabolites that may have a pharmacological importance (21, 22). Antitumor activity of *R. mucronata* extracts was found in various cancer-cell models, such as lung carcinoma cells (18, 23). Thus, *R. mucronata* appears not only as a source of active cysteine proteinase inhibitors, but other biological activities have also been reported from the extracts and from isolated constituents that warrant future investigation of this species for potential anticancer compounds (24). Nevertheless, no systematic all-in-one identification of R. mucronata phytochemicals acting as protein-selective modulators against lung cancer-related targets has been illustrated through an integrated method in silico studies till date.

Thus, we selected two mechanistically relevant NSCLC-associated proteins as molecular targets: the Kelch domain of Keap1 (PDB ID: 1X2J) and the YEATS domain of YEATS2 (PDB ID: 5XNV). Keap1 is a major negative regulator of Nrf2 and the Keap1–Nrf2 pathway is often abrogated in NSCLC. Mutations of Keap1 induce persistent Nrf2 activation, which can lead to antioxidant protection, metabolic adaptation, cell survival and more importantly anticancer treatment resistance (25, 26). Thus, targeting Keap1–Nrf2 offers a biologically relevant approach for probing redox-dependent mechanisms of NSCLC progression. Histone acetylation is associated with transcriptional activation. In lung cancer, amplification or overexpression of

YEATS2 is linked to the progression of NSCLC via oncogenic transcriptional programs associated with tumor-cell proliferation and survival (27). Hence, targeting the YEATS2 YEATS domain may allow a strategy to modulate aberrant acetylation-dependent transcriptional signaling. Thus, Keap1 and YEATS2 were chosen as composite molecular targets indicative of redox-regulatory and epigenetic mechanisms associated with NSCLC pathogenesis.

Here, we provide a systematic evaluation of phytochemicals from *R. mucronata* against Keap1 and YEATS2 to test our hypothesis that they could act as modulators of these proteins, representing an innovation in overcoming this identified knowledge gap via an integrated computational workflow. We used Density Functional Theory (DFT), molecular docking through AutoDock Vina/PyRx and Schrödinger Glide, ADMET profiling and 100-ns molecular dynamics simulations to provide the electronic properties, predicted binding interactions, pharmacokinetic behavior and complex stability. This multiscale approach was therefore carried out to rank potential phytochemicals for further in vitro validation instead of deriving conclusive biological activity directly from hundreds of thousands of predicted computationally.

2. Methodology

2.1 Optimization and Ligand Preparation

The 14 phytochemicals from *R. mucronata* were systematically selected based on a comprehensive literature review of its reported secondary metabolites, prioritizing chemical diversity across multiple classes including nitrogen-containing heteroaromatics (L01, L02, L05, L014), polyethers/siloxanes (L03, L13), long-chain esters/fatty acids (L04, L06, L09), polyhydroxy carbohydrates/sugar derivatives (L07, L08, L10), steroidal alcohols (L011), and aromatic oximes/naphthalene derivatives (L012) to (L014) cover a broad range of polarity, aromaticity, hydrogen-bonding capacity, and lipophilicity that could enable diverse interactions with target proteins. The Universal Force Field (UFF) was used for geometry pre-optimization to fix bond lengths and angles that were too long or too short, get rid of steric clashes, and get reasonable starting conformations. The Build Parameters module of Materials Studio was then used to make parameter files that included all the necessary force-field terms, atom types, and partial charges for the next quantum-mechanical calculations.

The DMol³ module in Materials Studio was employed to perform the density functional theory calculations using the computational level specified for this study. The molecular structures were initially subjected to geometry pre-optimization using the Universal Force Field (UFF) to remove unfavorable geometries and steric clashes. Solvent effects were incorporated using the COSMO model where applicable. Geometry optimization was continued until the specified convergence criteria for total energy and nuclear coordinates were satisfied. The optimized structures were subsequently used to calculate the frontier molecular orbital energies and global chemical-reactivity descriptors, including the HOMO–LUMO energy gap, ionization potential, electron affinity, chemical potential, electronegativity, hardness, softness, and electrophilicity index. The optimized structures were first checked for thermodynamic and structural stability by examining their geometries, bond parameters, and vibrational frequencies. From these stable structures, frontier molecular orbital properties and global reactivity descriptors were obtained, including HOMO and LUMO energies, HOMO–LUMO gap (E_gap), ionization potential (I), electron affinity (A), chemical potential (μ), electronegativity (χ), hardness (η), softness (s), and electrophilicity index (ω) (28-30), which are shown in equations 1–8.

$$E_{gap} = (E_{LUMO} - E_{HOMO}) \quad (1)$$

$$I = -E_{HOMO} \quad (2)$$

$$A = -E_{LUMO} \quad (3)$$

$$(X) = \frac{I+A}{2} \quad (4)$$

$$(\omega) = \frac{\mu^2}{2\eta} \quad (5)$$

$$(\mu) = -\frac{I+A}{2} \quad (6)$$

$$(\eta) = \frac{I-A}{2} \quad (7)$$

$$(S) = \frac{1}{\eta} \quad (8)$$

2.2 Protein Preparation and Collection

Used the Keap1 Kelch domain (PDB 1X2J) (31) and the YEATS2 YEATS domain (PDB 5XNV) (32) were selected as molecular docking targets because of their biological relevance to non-small cell lung cancer (NSCLC). Keap1 regulates Nrf2 turnover, and dysregulation of the Keap1–Nrf2 pathway contributes to persistent antioxidant signaling, metabolic adaptation, and chemoresistance in NSCLC. YEATS2 functions as an epigenetic reader of histone H3K27 acetylation (H3K27ac)

and is frequently amplified or overexpressed in NSCLC, where it promotes oncogenic transcriptional programs associated with tumor progression.

The crystal structures were retrieved from the Protein Data Bank. The selected structures had resolutions of 1.60 Å for 1X2J and 2.70 Å for 5XNV, with more than 96% of residues located in favored regions of their respective Ramachandran plots (Table 1). Receptor preparation was performed independently for the AutoDock Vina and Glide workflows. For AutoDock Vina docking, the structures were prepared using Discovery Studio by removing non-essential crystallographic ligands, water molecules, and ions, followed by energy minimization.

For Glide docking, the receptor structures were independently processed using the Protein Preparation Wizard in Schrödinger Maestro. The preparation workflow included assignment of bond orders, addition of hydrogen atoms, optimization of the hydrogen-bonding network, removal of unnecessary water molecules, and restrained energy minimization. The resulting prepared receptor structures were subsequently used for receptor-grid generation and Glide Standard Precision (SP) docking.

Table 1. **Protein information.**

**Title**	**PDB ID: 1X2J**	**PDB ID:** 5XNV
Organism	*Homo sapiens*	*Homo sapiens*
Resolution	1.60 Å	2.70 Å
R-Value Free	0.200	0.266
Ramachandran plot, %	96.18%	96.92%
References	(31)	(32)

2.3 Molecular Docking and Visualization of Docking

2.3.1 Molecular Docking by PyRx

Molecular docking was performed using PyRx 0.8 with the AutoDock Vina engine to predict the binding affinities and preferred binding poses of the 14 phytochemicals against the two target proteins. Prepared receptor and ligand structures were imported separately into PyRx. For each

receptor, a rectangular docking grid was defined using the center coordinates and dimensions reported in Table 2 to encompass the biologically relevant binding region. The conformational search was conducted using an exhaustiveness value of 8 and nine output binding modes. The resulting poses were ranked according to the AutoDock Vina docking score, expressed in kcal/mol. Protein–ligand complexes were subsequently visualized and analyzed in Discovery Studio for hydrogen bonding, hydrophobic interactions, π interactions, and other non-covalent contacts.. This methodical approach enabled reliable identification of the most satisfactory binding modes and quantitative comparison of ligand affinities for both 1X2J and 5XNV (33).

2.3.2 Molecular Docking by Glide from Schrodinger suite

Molecular docking was performed using the Glide module of the Schrödinger Suite (Release 2024-1) to provide an independent evaluation of ligand binding to Keap1 (PDB ID: 1X2J) and YEATS2 (PDB ID: 5XNV). Ligands were prepared using LigPrep to generate appropriate three-dimensional structures, ionization states, and tautomers under the selected preparation conditions. Receptor grids were generated around the defined binding regions using the Receptor Grid Generation module, with the grid centers and dimensions provided in Table 2 (34), to encompass the entire biologically relevant pocket while maintaining computational efficiency.

Docking was subsequently performed using Standard Precision (SP) mode, and the generated poses were ranked using GlideScore, which accounts for van der Waals, Coulomb, lipophilic, hydrogen-bonding, metal-binding, and desolvation terms, as well as penalties for buried polar groups and rotatable-bond freezing equation [9].

$$G\ Score = a \times vdW + b \times coul + Lipo + HBond + Metal + BuryP + RotB + Site \qquad (9)$$

Here, vdW is the van der Waals term, Coul is the Coulombic (electrostatic) term, Lipo represents lipophilic contacts, HBond corresponds to hydrogen-bonding interactions, Metal captures metal-binding contributions, BuryP is the penalty for buried polar groups, RotB is the penalty for freezing rotatable bonds, and Site represents polar interactions within the active site; the coefficients for vdW and Coul are a = 0.065 and b = 0.130, respectively.

Table 2. **Grid box parameters used for docking analysis in this study.**

	Grid Box Size (PYRX)	Grid Box Size (Glide)

Protein Name with PDB ID	Center	Dimension(Å)	Center	Dimension(Å)
Homo sapiens (PDB 1X2J)	X = 22.67<br>Y = 61.39<br>Z = 37.82	X = 55.81<br>Y= 44.93<br>Z = 49.73	X =20<br>Y =60<br>Z =30	X =30<br>Y =20<br>Z =30
Homo sapiens (PDB 5XNV)	X = -14.77<br>Y = -31.53<br>Z = -18.45	X = 36.44<br>Y = 48.80<br>Z = 59.16	X =-10<br>Y =-30<br>Z =-20	X =30<br>Y =30<br>Z =30

2.4 Molecular Dynamic

To assess the stability of the most promising protein–ligand complexes under physiological conditions, 100-ns all-atom MD simulations were carried out for the top-ranked poses 1X2J–L10 and 5XNV–L02 using Desmond (Schrödinger Release 2024-1; D. E. Shaw Research, New York, NY, USA). Each system was parameterized with the OPLS-AA force field, placed in an orthorhombic periodic box of TIP3P water with a 10 Å buffer, and electrically neutralized by adding Na^+ or Cl^- counter-ions. A standard multi-stage equilibration protocol was applied: steepest-descent energy minimization, gradual heating to 300 K, and restrained equilibration (NVT followed by NPT) to relax the solvent while gradually releasing restraints on the solute. Production MD runs were conducted in the NPT ensemble (300 K, 1.01325 bar) employing the Nose-Hoover thermostat and Martyna-Tuckerman-Klein barostat, with a 2-fs integration time step and SHAKE constraints on all bonds involving hydrogen atoms. Trajectory files and energy logs were analyzed in Maestro and the Simulation Event Analysis module of Desmond. Main constancy metrics including root-mean-square deviation (RMSD) of the protein Cα atoms, root-mean-square fluctuation (RMSF) per residue, radius of gyration (R_g), and persistent protein–ligand contacts (hydrogen bonds, hydrophobic interactions, and water bridges) were calculated over the entire 100-ns trajectory (35). This inclusive computational investigation followed conventional best practices and parameters, enabling a thorough exploration of the molecular system's dynamic behavior.

2.5 Lipinski Rule, Drug-likeness and Pharmacokinetics

On October 9, 2025, SwissADME, a common online platform and its famous for flexibility and convenience, was attached to predict pharmacokinetics and evaluate drug-like assets (36). In this step, the online SwissADME platform (http://www.swissadme.ch/index.php) was used to compute key pharmacokinetic properties and assess drug-like features of the ligands. Core descriptors, including molecular weight, number of rotatable bonds (NRB), lipophilicity, and counts of hydrogen bond donors (HBD) and acceptors (HBA), were systematically evaluated to characterize their prospective pharmacological profiles.

2.6 **Absorption, distribution, metabolism, excretion, and toxicity (ADMET) properties**

The absorption, distribution, metabolism, excretion, and toxicity (ADMET) properties, which are key factors in drug development, were evaluated in silico (37, 38). ADMET descriptors were obtained using the SwissADME and pkCSM online platformshttp://biosig.unimelb.edu.au/pkcsm/prediction_single/adme_1643650057.59 (accessed from the 10 October 2025) (39).

3. Results and Discussion

3.1 Quantum calculation by DFT

3.1.1 Chemistry and Optimized Structure of phytochemicals from mangrove tree, R. mucronata

The selected 14 compounds, listed and coded with their chemical name shown in below and Figure S1, from *R. mucronata* cover a wide chemical space which are chemically six type of compounds, such as Nitrogen heteroaromatics (L01, L02, L05, L014), Polyethers and siloxanes (L03, L13), Long-chain esters and fatty acids (L04, L06, L09), L06, Methyl The 14 phytochemicals selected from the mangrove species *R. mucronata* (structures provided in Figure S1) encompass a broad and diverse chemical space. These compounds can be categorized into six distinct structural classes: nitrogen heteroaromatics (L01, L02, L05, L14), polyethers and siloxanes (L03, L13), long-chain esters and fatty acids (L04, L06, L09), polyhydroxy carbohydrates and sugar-like derivatives (L07, L08, L10), a steroidal alcohol (L11), and an aromatic oxime/naphthalene derivative (L12). This structural diversity translates into a wide array of potential protein–ligand interactions.

Specifically, the polar polyhydroxy and sugar-like ligands (L07, L08, L10) are primed for strong hydrogen bonding and specific molecular recognition. The aromatic nitrogen heterocycles and fused ring systems (L01, L02, L05, L12, L14) facilitate π–π stacking, hydrogen bonding, and potential metal coordination. Hydrophobic lipids and steroids (L04, L06, L09, L11) are well-suited for interacting with nonpolar protein regions and cellular membranes. Finally, the siloxane-type scaffolds (L03, L13) provide flexible, lipophilic, and weakly polar backbones. Collectively, the variance in aromaticity, hydrogen-bonding capacity, lipophilicity, and conformational flexibility supports a broad spectrum of possible biological interactions. This chemical diversity aligns with existing evidence that *R. mucronata* extracts are rich in phenols, fatty acids, terpenoids, alkaloids, and other secondary metabolites possessing notable antioxidant and anticancer potential.

The chemical name of selected 14 phytochemicals used in our study which are extracted from the from *Rhizophora mucronate*, as mangrove tree in our mangrove forest, is as 4-chloro-3-methoxy-2-methylpyridine (**L01**), benzo[h]quinoline-4-carboxylic acid **(L02),** 2,2,4,4,6,6,8,8,10,10,12,12,14,14-tetradecamethyl-1,3,5,7,9,11,13-heptaoxacyclotetradecane **(L03),** Methyl 14-(2-octylcyclopropyl)tetradecanoate **(L04)**, N-(3-fluorobenzyl)-7H-purin-6-amine **(L05),** Methyl 14-methylpentadecanoate **(L06),** (E)-3,4,5-trihydroxy-6-(hydroxymethyl)tetrahydro-2H-pyran-2-yl N-hydroxybut-3-enimidothioate **(L07),** (2R,3S,4R,5R)-2,4,5,6-tetrahydroxy-3-methoxyhexanal **(L08),** Octadeca-9,12-dienoic acid **(L09),** Cyclohexane-1,2,3,4,5,6-hexaol **(L10),** ((8S,9S,10S,13R,14S,17R)-13-methyl-17-((R)-6-methylheptan-2-yl)-2,3,4,7,8,9,10,11,12,13,14,15,16,17-tetradecahydro-1Hcyclopenta[a]phenanthren-10-yl)methanol **(L011),** (E)-5,6-dimethoxy-3,4-dihydronaphthalen-1(2H)-one oxime **(L012),** 2,2,4,4,6,6,8,8,10-nonamethyl-3,5,7,9-tetraoxaundecane **(L13),** 1,4,5,8-tetraphenyl-1H-thiopyrano[4,3-c]pyridine **(L014).**

In applying quantum-chemical methods to these compounds, careful optimization of molecular geometry was essential to obtain reliable structural descriptions. The study focused on locating the lowest-energy conformation for each molecule to set robust computational parameters(40). Using a DFT functional, all structures were energy-minimized, yielding their predominant, thermodynamically favored configurations with minimal calculated energy. The ligands are illustrated in Figure 1 and Figure S1.

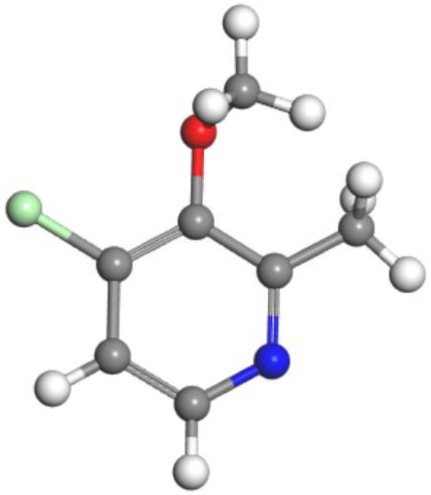

4-chloro-3-methoxy-2-methylpyridine (L01)

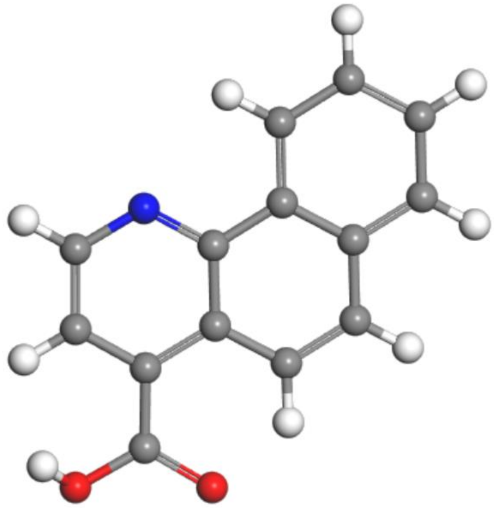

Benzo[h]quinoline-4-carboxylic acid (L02)

Figure 1. Optimized structure of inhibitors

3.1.2 HOMO, LUMO, and Chemical Reactivity Descriptors

Based on the optimized structures from DFT (B3LYP/6-31G*), molecular parameters such as HOMO and LUMO energies, HOMO–LUMO energy gap (ΔE_gap), chemical potential (μ), electronegativity (χ), hardness (η), softness (σ) and electrophilicity index (ω) were calculated and summarized in Table 3. The HOMO–LUMO gap is indicative of molecular reactivity and stability (the smaller, the more reactive; the larger, the stable) (41-44).

Table 3. **Data of chemical descriptors from the DFT calculation**

Ligands	LUMO	HOMO	electron affinity (A)	Ionization potential (I)	Energy gap	chemical potential (μ)	chemical hardness (η)	electronegativity (χ)	softness (S)	electrophilicity index (ω)
L01	-9.17	-0.86	9.17	0.86	-8.31	-5.01	-4.15	5.01	-0.24	-3.02
L02	-8.45	-2.22	8.45	2.22	-6.23	-5.34	-3.11	5.34	-0.32	-4.57
L03	9.289	-1.33	-10.62	-3.97	-5.31	3.97	-0.18	-1.48	9.28	-1.33
L04	-9.05	-0.36	9.05	0.36	-8.68	-4.70	-4.34	4.70	-0.23	-2.55

L05	-8.84	-1.00	-0.04	8.94	8.98	-4.45	4.49	4.45	0.22	2.20
L06	-9.90	-0.48	9.90	0.48	-9.42	-5.19	-4.71	5.19	-0.21	-2.86
L07	-8.67	-0.35	8.67	0.35	-8.31	-4.51	-4.15	4.51	-0.24	-2.45
L08	-9.65	-0.97	9.65	0.97	-8.68	-5.31	-4.34	5.31	-0.23	-3.24
L09	-8.28	-0.65	8.28	0.65	-7.63	-4.46	-3.81	4.46	-0.26	-2.61
L10	-10.51	0.36	10.51	-0.36	-10.87	-5.07	-5.43	5.07	-0.18	-2.36
L11	-8.05	1.07	8.05	-1.07	-9.12	-3.49	-4.56	3.49	-0.21	-1.33
L12	-8.21	-0.28	8.21	0.28	-7.93	-4.25	-3.96	4.25	-0.25	-2.27
L13	10.31	-1.60	-11.91	-4.35	-5.95	4.35	-0.16	-1.592	10.31	-1.60
L14	-8.03	-0.97	8.03	0.97	-7.05	-4.50	-3.52	4.50	-0.28	-2.87

The calculated quantum-chemical descriptors demonstrated differences in electronic stability and reactivity among the investigated phytochemicals. Compounds with smaller HOMO–LUMO energy gaps are generally considered more electronically polarizable and reactive, whereas larger energy gaps are associated with greater electronic stability. However, these descriptors characterize intrinsic electronic properties and should not be interpreted as direct predictors of anticancer activity or protein-binding affinity. Their principal value in the present study is to complement the molecular docking and molecular dynamics analyses by providing additional physicochemical information on the screened compounds.

The calculated energy gaps ranged widely from 0.36 eV (L10) to 8.98 eV (L05). Ligand L10 exhibited the smallest HOMO–LUMO gap, indicating higher electronic reactivity and a potentially greater tendency for charge transfer. In contrast, L05 showed the largest energy gap, consistent with higher electronic stability. Variations were also observed in other descriptors, including chemical potential, hardness, and electrophilicity index. For example, L10 displayed the most negative chemical potential and relatively high electrophilicity, whereas L05 exhibited contrasting behavior.

The quantum chemical descriptors revealed notable differences in the electronic properties and reactivity profiles across the ligand series. Ligands L01, L04, L06, L08, and L10 showed the most negative LUMO energies (ranging from −9.05 to −10.51 eV). L10, in particular, exhibited the lowest LUMO energy (−10.51 eV). However, these DFT-derived parameters provide only supportive insights into the intrinsic electronic characteristics of the compounds. They are not

validated predictors of biological or anticancer activity, and a smaller HOMO–LUMO gap should not be interpreted as implying superior therapeutic potential. These results are best viewed as complementary chemical characterization that may help rationalize possible molecular interaction mechanisms when considered alongside docking and molecular dynamics data.

3.1.3 Frontier Molecular Orbital (FMOs): HOMO and LUMO

Frontier molecular orbital analysis was employed to examine the electronic distribution of the screened phytochemicals. The HOMO identifies regions with relatively greater electron-donating character, whereas the LUMO indicates regions capable of accepting electron density. The HOMO–LUMO energy gap was considered an electronic-reactivity descriptor and was not interpreted as a direct measure of protein-binding affinity or anticancer activity (45-49). A smaller HOMO–LUMO energy gap favors stronger interaction of the ligands with protein targets, and the patterns seen in Figure 2 and Figure S2 reveal multiple HOMO and LUMO-rich zones that help explain their predicted reactivity and pharmacological potential.

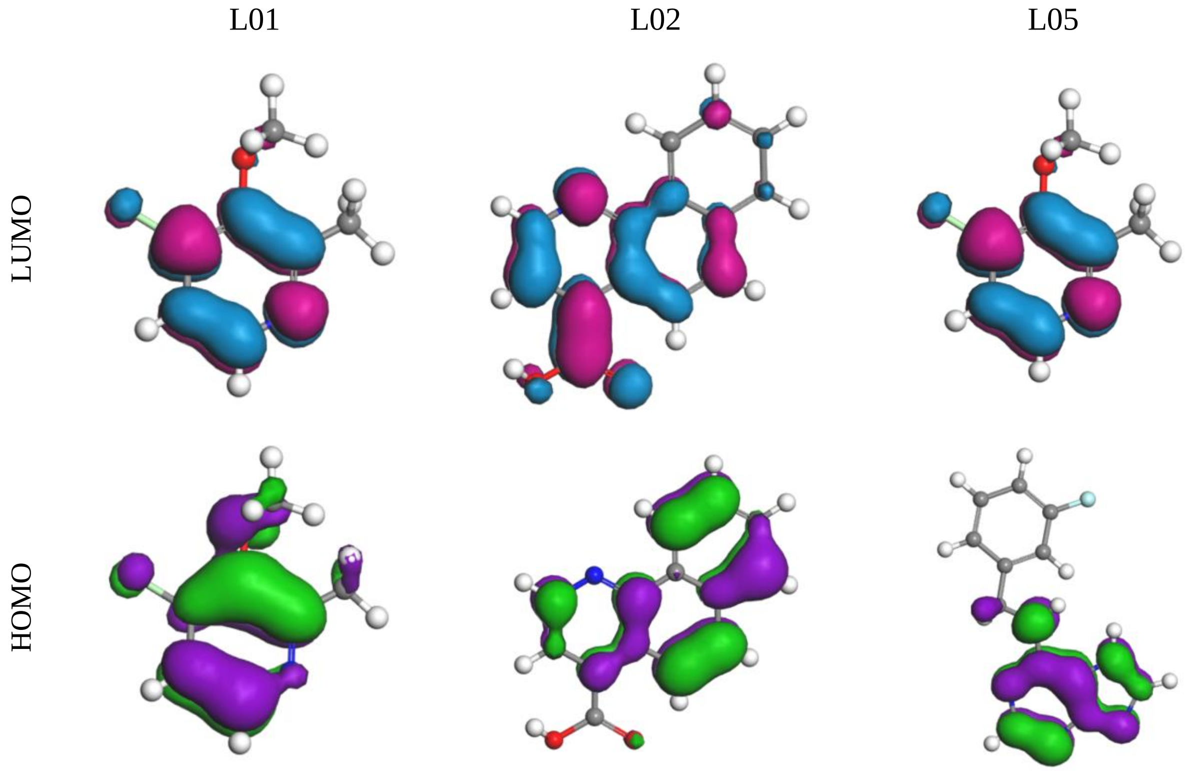

Figure 2. Frontier molecular orbitals diagram for HOMO and LUMO

3.1.4 Electrostatic potential map for Frontier Molecular Orbital

Understanding the reactivity and stability of molecules in chemical reactions is paramount, often elucidated through electrostatic potential maps of Frontier Molecular Orbitals (FMO). The pivotal roles played by HOMO and the LUMO in dictating a molecule's reactivity, as proposed by FMO theory, cannot be overstated. Analysis of the HOMO electrostatic potential map offers valuable insights into regions of electron density susceptible to nucleophilic attacks, while the LUMO map identifies sites prone to electrophilic attacks due to electron deficiency. By examining these maps, chemists can forecast reaction mechanisms and outcomes. Moreover, comparative analysis of electrostatic potential maps among different molecules enables the assessment of their relative reactivity and selectivity in diverse reactions, facilitating rationalization and prediction of chemical reactivity and aiding in the design of molecules with tailored properties. Figure 3 and Figure S3.

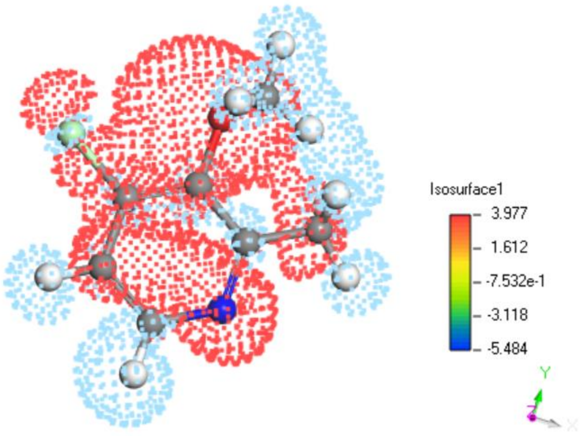

L01

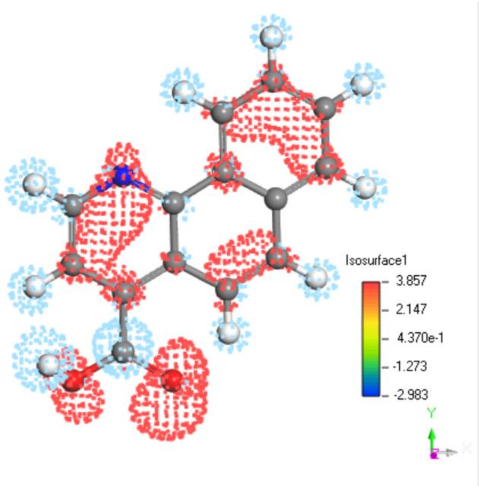

L02

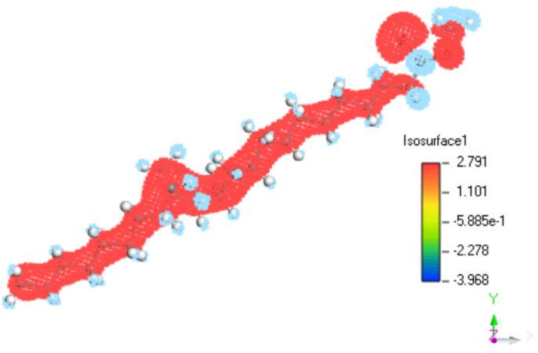

L04

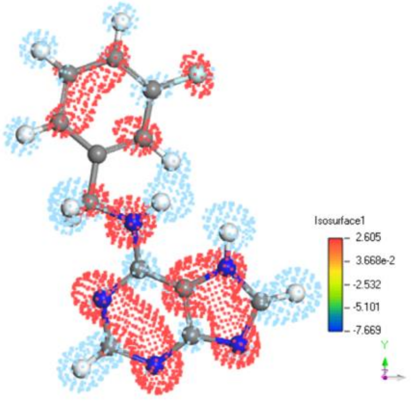

L05

Figure 3. Electrostatic potential map for Frontier Molecular Orbital of L01-L04

3.2. Molecular Docking

3.2.1 Auto docking by PyRx

Molecular docking was carried out to support the pharmacological observations. Binding affinities of twelve ligands against the two targets (PDB IDs 1X2J and 5XNV) were evaluated using AutoDock within the PyRx interface. Strong protein–ligand recognition is essential for designing structure-based microbial agents, and docking scores more negative than about −6.0 kcal/mol are often taken as indicative of promising activity (50, 51). Docking also helps clarify how two molecules fit together and which pose best satisfies the binding site. In these in-silico studies, the ligands listed in Table 4 showed markedly favorable binding energies toward both targets, suggesting good potential as anticancer candidates.

Table 4. **Data of binding energy and name of interacted ligand against lung cancer in binding affinity (kcal/mol).**

**S. L**	**1X2J**			**5XNV**		
	binding affinity (kcal/mol)	No of H bond	No Hydrophobic bond	binding affinity (kcal/mol)	No of H bond	No Hydrophobic bond
L01	-5.1	1	3	-4.3	2	3
L02	-8.3	2	2	-6.3	1	3
L03	-8.4	1	0	-6.8	2	0
L04	-5.8	2	4	-4.0	0	1
L05	-8.5	3	5	-6.2	4	1
L06	-5.6	1	8	-3.8	0	1
L07	-7.7	3	4	-5.3	2	2
L08	-5.5	5	2	-4.4	2	1
L09	-5.4	1	1	-4.2	2	1
L10	-6.5	6	1	-5.6	3	2
L11	-8.9	1	11	-6.4	1	10
L12	-7.3	2	5	-6.0	2	4
L13	-6.8	1	0	-4.3	2	0
L14	-8.8	0	9	-6.9	0	7

Molecular docking using PyRx (AutoDock Vina) revealed moderate binding affinities for the screened *R. mucronata* phytochemicals against the target proteins 1X2J (Keap1) and 5XNV (YEATS2), with scores generally falling in the range of −4.0 to −8.9 kcal/mol (Table 4). For the Keap1 Kelch domain (1X2J), ligands L11 and L14 exhibited the most favorable binding scores (−8.9 and −8.8 kcal/mol, respectively), followed by L05 and L02 (−8.5 and −8.3 kcal/mol), while L10 formed the highest number of hydrogen bonds (six) despite a comparatively moderate docking score. In contrast, several ligands (L01, L04, L06, L08, and L09) showed weaker binding energies (approximately −5.1 to −5.8 kcal/mol), and L03 and L13 failed to produce reliable poses. A broadly similar pattern was observed for the YEATS2 domain (5XNV), where L14, L11, L05, L12, and L02 displayed relatively better scores (−6.9 to −6.0 kcal/mol) compared with other compounds. These docking results indicate that multiple ligands can establish hydrogen bonds and hydrophobic contacts within the binding pockets; however, the observed affinities remain in the moderate range typical of initial screening hits. It is important to note that docking scores alone do not confirm biological activity, and benchmarking against known inhibitors or co-crystallized ligands would be necessary to better assess the relative significance of these findings. The differences observed between PyRx and subsequent Glide docking results further highlight the importance of using orthogonal methods and cautious interpretation when prioritizing lead candidates.

3.2.2 Molecular Docking by Glide from Schrodinger suite

Binding affinities of twelve ligands toward the two lung cancer targets 1X2J and 5XNV were assessed using Glide docking. Ligands were prepared with LigPrep (Schrödinger suite) to obtain high-quality, atom-level 2D and 3D representations; this workflow included 2D-to-3D conversion, generation of reasonable tautomers and stereoisomers, and subsequent correction and optimization of each structure. Receptor grids for docking were then constructed with the Receptor Grid Generation module of Glide within Maestro (Schrödinger Release 2024-1: Glide, Schrödinger, LLC, New York, NY, 2024) (52-54).

Table 5. **Data of binding energy and name of interacted ligand against lung cancer in binding affinity (kcal/mol).**

S. L	1X2J	5XNV

	binding affinity (kcal/mol)	No of H bond	No. Hydrophob ic bond	binding affinity (kcal/mol)	No of H bond	No. Hydrophob ic bond
L01	-5.944	1	4	-4.814	0	5
L02	-5.137	0	6	-5.733	2	3
L03	-4.427	0	10	-2.168	1	4
L04	-5.816	0	2	NA	NA	NA
L05	-6.779	0	3	-3.783	2	4
L06	-1.507	3	7	-1.498	2	5
L07	-7.407	10	4	-4.252	4	3
L08	-6.925	11	3	-3.572	6	1
L09	-2.952	1	2	NA	NA	NA
L10	-7.789	8	4	-5.482	5	0
L11	-3.877	1	5	-3.338	2	5
L12	-5.296	3	3	-4.454	2	3
L13	-1.873	0	7	-1.817	1	1
L14	-5.355	1	7	-2.509	0	5

The rank ordering of the ligands differed between AutoDock Vina and Glide. In the AutoDock Vina analysis, L11 and L14 showed the most favorable scores against 1X2J and 5XNV, respectively, whereas Glide identified L10 as the highest-ranked ligand for 1X2J and L02 as the highest-ranked ligand for 5XNV. These differences are not unexpected because AutoDock Vina and Glide employ different receptor-preparation procedures, conformational-search algorithms, scoring functions, and treatment of intermolecular interactions. Therefore, the results from the two platforms were interpreted as complementary rather than directly interchangeable. The Glide-ranked 1X2J–L10 and 5XNV–L02 complexes were subsequently selected for molecular dynamics simulations to maintain methodological consistency between Glide ranking, interaction analysis, and MD lead selection.

3.2.3 Protein-Ligand Interaction for PyRx in view of superpose/position

In the complex domain of molecular docking, the alignment of ligands after docking is a crucial step, similar to elucidating the intricate interaction between molecules on a minuscule scale, as

depicted in Figure 4. Superposition of the docked ligand poses was used to compare their spatial orientations within the receptor-binding pockets. Differences in ligand orientation, pocket occupancy and interactions with key amino-acid residues were examined to identify common binding features and ligand-specific interaction patterns. This analysis provided complementary structural information for interpreting the docking scores and identifying potentially favorable binding modes.

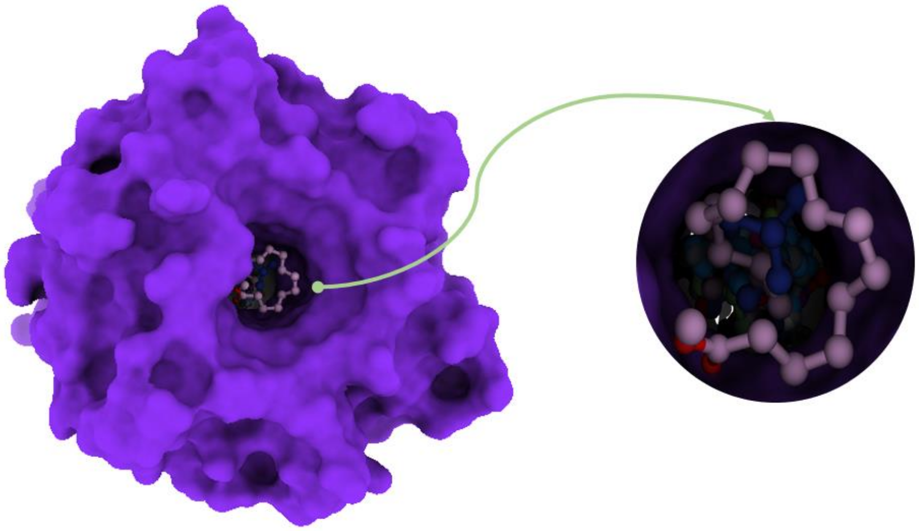

(a)

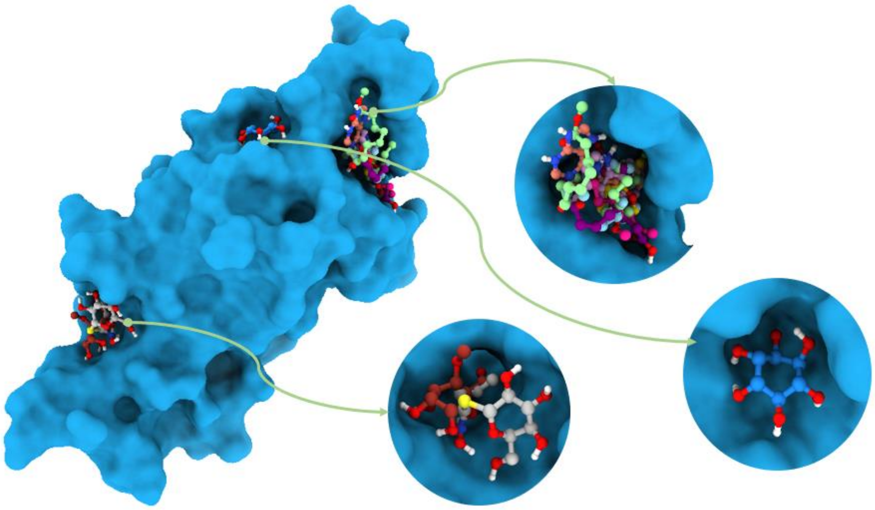

(b)

Figure 4. Molecular docking poses of (a) 1X2J; (b) 5XNV

3.2.4 Different poses of docking for protein ligand interaction BY PyRx Docking

Post-docking interaction analysis was performed using Discovery Studio to characterize the binding poses generated by AutoDock Vina. According to the PyRx results, L11 exhibited the most favorable binding affinity toward Keap1 (1X2J), with a docking score of −8.9 kcal/mol, one hydrogen bond, and eleven hydrophobic contacts. For YEATS2 (5XNV), L14 showed the most favorable PyRx docking score of −6.9 kcal/mol and formed seven hydrophobic contacts without a conventional hydrogen bond. These findings identify L11 and L14 as the highest-ranked PyRx ligands for 1X2J and 5XNV, respectively.

L12, which is illustrated in Figure 6 as a representative YEATS2-binding pose, showed a docking score of −6.0 kcal/mol against 5XNV and formed two hydrogen bonds and four hydrophobic contacts according to Table 4. Therefore, L12 should not be described as the highest-ranked PyRx ligand for 5XNV. Its interaction map is presented only to illustrate a representative mixed polar and hydrophobic binding mode within the YEATS2 pocket.

Based on the PyRx docking results, ligand L11 showed the highest binding affinity toward protein 1X2J, forming two hydrogen bonds and six hydrophobic interactions. In contrast, ligand L12 displayed the strongest binding with protein 5XNV, forming seven hydrophobic interactions without any hydrogen bonds.

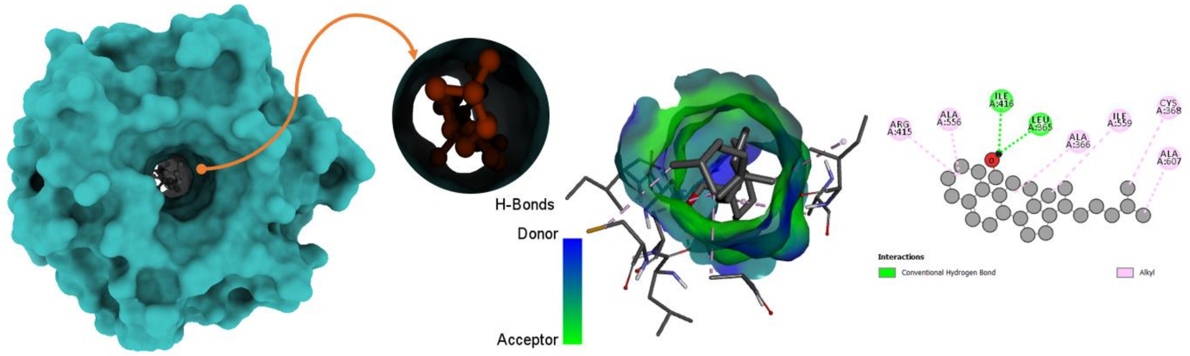

Figure 5. Molecular docking poses of lung cancer PDB (1X2J)- L11

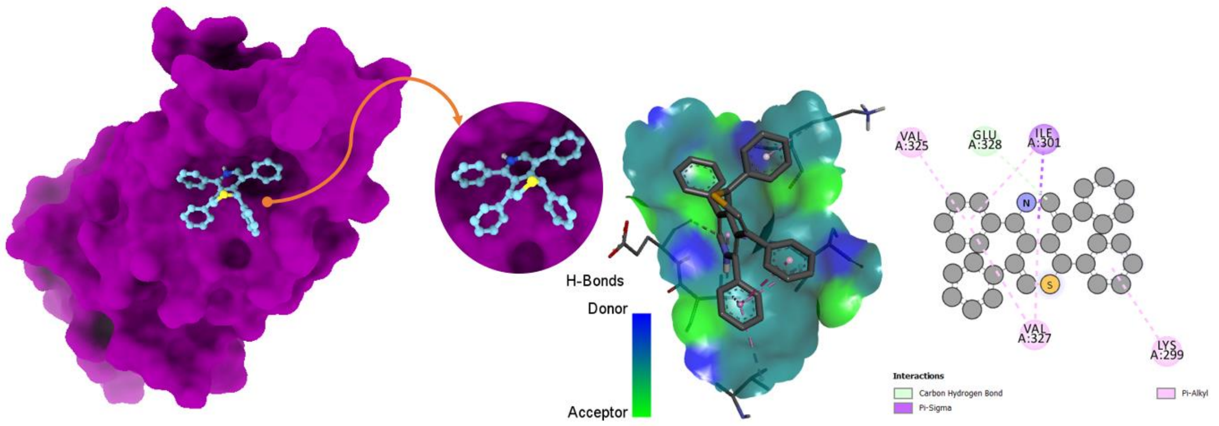

Figure 6. Molecular docking poses of lung cancer PDB (5XNV) -L12

Both L11 and L12 adopted well-defined poses within the functional binding pockets of Keap1 (PDB: 1X2J) and YEATS2 (PDB: 5XNV), respectively, which supports their predicted roles as potential lung cancer inhibitors. In Figure 5, the global surface view confirms that L11 is deeply buried within the central cavity of Keap1, indicating good shape complementarity and pocket occupancy. The enlarged 3D representation shows that L11 is surrounded by regions enriched in hydrogen-bond donor and acceptor sites, as indicated by the colored surface, suggesting a strong contribution of polar contacts to the overall binding. The accompanying 2D diagram further highlights multiple hydrogen bonds and non-bonded interactions between L11 and nearby residues inside the Keap1 pocket, identifying the key amino acids that anchor the ligand and likely drive the favorable docking score. Together, these panels suggest that L11 is stabilized by a balanced network of hydrogen bonding and hydrophobic contacts within a well-defined Keap1 pocket, consistent with a strong and specific binding mode.

Similarly, Figure 6 illustrates the representative binding orientation of L12 within the YEATS2 binding pocket. The interaction analysis indicates two hydrogen bonds together with four hydrophobic contacts, consistent with the interaction data reported in Table 4. These contacts support stabilization of the predicted docking pose; however, L12 was not the highest-ranked PyRx ligand for 5XNV. The zoomed 3D poses shows that L12 occupies a deep sub-pocket and is surrounded by donor and acceptor regions comparable to those seen for L11, which allows a direct visual comparison of pocket burial and interaction hot spots between the two ligands. The 2D interaction map reveals that L12 forms several hydrogen bonds, π–π stacking, and hydrophobic

contacts with residues such as Glu, Lys, and Ser, supporting its docking-predicted affinity and suggesting a mixed polar–aromatic binding mechanism. Taken together, these interaction patterns indicate that both L11 and L12 engage critical residues within their respective protein targets, implying shared pharmacophoric elements that may underlie their predicted anticancer activity through modulation of Keap1–Nrf2 signaling and YEATS2-mediated histone reading in lung cancer.

3.2.5 Protein ligand interaction from Glide docking

Protein–ligand interaction analysis of the Glide-ranked complexes was performed for 1X2J–L10 and 5XNV–L02. For 1X2J, L10 exhibited the most favorable GlideScore (−7.789 kcal/mol) and established an extensive polar interaction network within the binding pocket. For 5XNV, L02 showed the most favorable GlideScore (−5.733 kcal/mol) and formed a combination of hydrogen-bonding, aromatic and hydrophobic interactions within the YEATS domain. Figure 7 illustrates the corresponding two-dimensional interaction patterns of the two Glide-selected complexes. These interaction maps were used to characterize the predicted binding modes and to support selection of the complexes for subsequent molecular dynamics simulations.

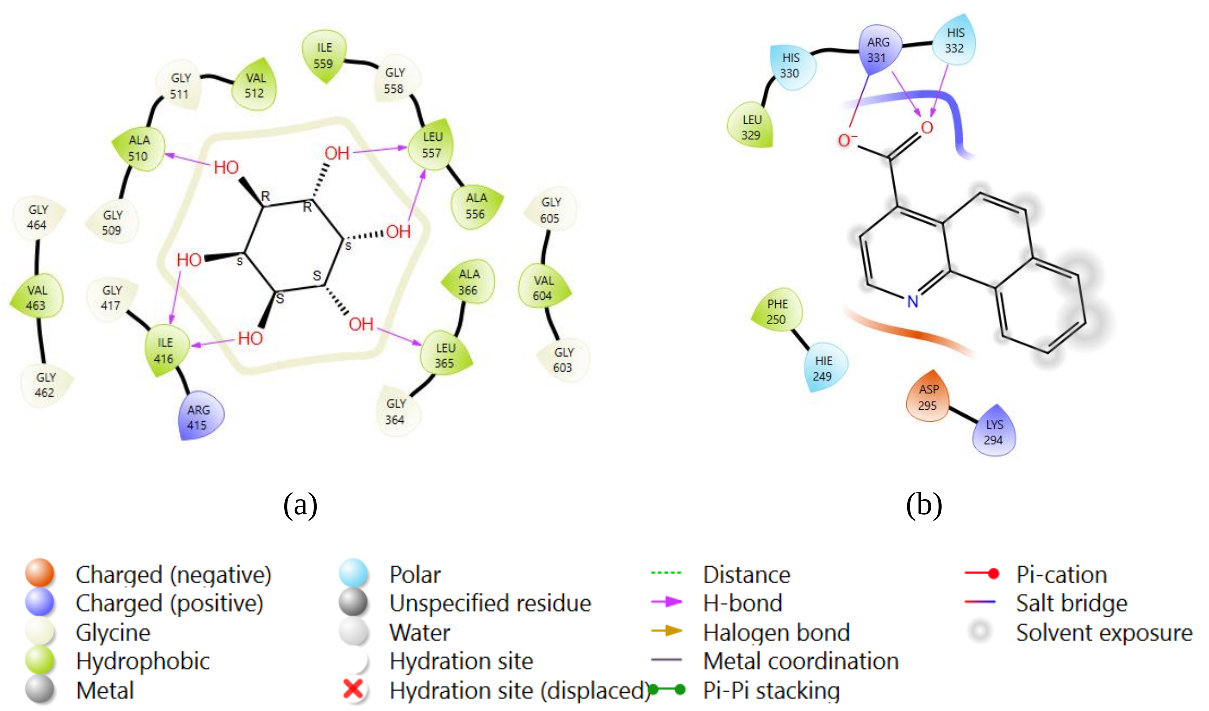

Figure 7. 2D stick diagram of (a) PDB; 1X2J -L10; (b) PDB; 5XNV -L02, illustrating hydrogen bonds and pi-pi stacking formed with the amino-acid residues at the binding pocket of lung cancer.

Figure 7 shows the 2D interaction patterns of two key ligands, L10 and L02, within the Keap1 (1X2J) and YEATS2 (5XNV) lung cancer targets, and how they use both polar and hydrophobic contacts to bind tightly. In panel (a), L10 forms several hydrogen bonds through its hydroxyl groups with nearby Keap1 residues, while surrounding hydrophobic side chains (Val, Leu, Ile, Ala) build a non-polar shell that locks the scaffold in place; this dense hydrogen-bond network, supported by van der Waals contacts, points to strong affinity and good shape fit within the Keap1 pocket linked to Nrf2 control in NSCLC.

In panel (b), L02 occupies the YEATS2 pocket with a mixed interaction pattern, where the heteroaromatic ring forms π–π and cation–π contacts with aromatic and positively charged residues such as Arg and His, and extra hydrogen bonds and hydrophobic contacts around the ring help fix its orientation toward the H3K27ac recognition region. These π-based contacts, together with polar anchoring groups, match the known function of the YEATS domain as an acetyl-lysine reader and support the view that L02 can mimic or compete with native histone ligands, placing its docking pose in line with current evidence that YEATS2 acts as an epigenetic driver and promising therapeutic target in NSCLC.

3.2.6 Benchmarking with Known Inhibitors

The docking results obtained in this study were interpreted in the context of previously reported inhibitors and structural studies involving the Keap1 Kelch and YEATS2 YEATS domains(55, 56). Docking scores are dependent on the docking software, receptor-preparation protocol, scoring function, grid definition, and ligand-preparation procedure; therefore, absolute docking scores obtained from different studies cannot be directly interpreted as experimentally measured binding free energies (57). For Keap1 (1X2J), AutoDock Vina identified L11 as the highest-ranked ligand, with a docking score of −8.9 kcal/mol, whereas Glide identified L10 as the highest-ranked ligand, with a GlideScore of −7.789 kcal/mol. For YEATS2 (5XNV), L14 showed the most favorable AutoDock Vina score (−6.9 kcal/mol), while L02 showed the most favorable GlideScore (−5.733 kcal/mol). These values indicate favorable predicted interactions within the respective docking

models but should not be interpreted as experimentally determined binding affinities or thermodynamic binding free energies (58, 59). Reported Keap1 and YEATS-domain inhibitors were therefore used primarily as qualitative structural and pharmacological references rather than as directly comparable numerical standards. In particular, docking scores expressed in kcal/mol are distinct from the octanol–water partition coefficient (logP), which describes molecular lipophilicity and is dimensionless. Accordingly, no direct relationship between logP and docking energy is assumed in the present analysis. The screened phytochemicals should be regarded as preliminary computational hits requiring experimental validation and further structure–activity optimization before conclusions regarding inhibitory potency can be established

3.3 Molecular Dynamics

3.3.1 Root Mean Square Deviation (RMSD)

For subsequent molecular dynamics simulations, lead complexes were selected according to the Glide ranking and interaction profiles. L10 exhibited the most favorable GlideScore against 1X2J (−7.789 kcal/mol), whereas L02 showed the most favorable GlideScore against 5XNV (−5.733 kcal/mol). Consequently, the 1X2J–L10 and 5XNV–L02 complexes were selected for 100-ns MD simulations. The structural stability of the complexes was evaluated by monitoring the root-mean-square deviation (RMSD) of the protein Cα atoms over the simulation time (Figure 8).

As shown in Figure 8, the RMSD values for both complexes remained mostly below 3 Å throughout the 100-ns trajectory after an initial equilibration period. This relatively low deviation suggests that ligand binding did not cause major conformational disruptions to the protein backbone and that the complexes maintained overall structural integrity during the simulation.

It should be noted that while these RMSD profiles indicate acceptable stability of the docked poses under the simulation conditions, RMSD analysis alone is insufficient to confirm binding strength, specificity, or thermodynamic favorability. Additional analyses such as binding free energy calculations (e.g., MM-PBSA/MM-GBSA), comparison with apo-protein simulations, or more detailed per-residue interaction mapping would be required for a more comprehensive mechanistic understanding. Therefore, the present MD results should be interpreted as preliminary supportive evidence of complex stability rather than definitive validation of strong ligand binding.

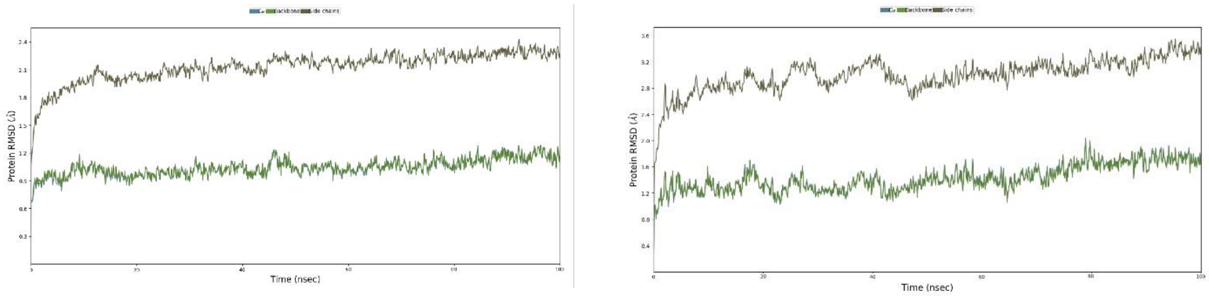

(a) **(b)**

Figure 8. Protein-ligand complexes (a) PDB; 1X2J -L10; (b) PDB; 5XNV -L02

Throughout the simulation trajectory, the RMSD profiles provided critical insights into the conformational landscapes of the complexes. A detailed examination of the temporal evolution from 0 to 34 ns revealed a rapid equilibration of the protein backbones. Notably, for the duration of the simulation, the RMSD values for both complexes remained consistently below the critical 2–3 Å threshold, which is conventionally indicative of a stable protein fold. This sustained low deviation from the initial structure confirms that the binding of the ligands (L10 and L02) does not induce major conformational shifts and that the overall tertiary structure of each protein is preserved. The consistently stable RMSD profiles underscore the inherent robustness of the docked poses and affirm the formation of resilient ligand-protein complexes. This observed structural stability is a strong indicator of reliable binding modes and enhances confidence in the computational predictions. Consequently, these findings provide a compelling foundation for the prospective application of these complexes in pharmaceutical development, such as in structure-based drug design or hit-to-lead optimization campaigns.

3.3.2 Root Mean Square Fluctuation **(RMSF)** *with respect of residues*

Glide, developed by Schrödinger, is a widely used molecular docking tool designed to predict the binding orientation of small molecules (ligands) within the active site of target proteins. Analysis of protein–ligand interactions following Glide docking involves examining the docking results and visualizing the predicted binding modes. Glide provides docking scores that estimate binding affinity, where lower scores correspond to stronger binding.

Post-docking analyses play an essential role in understanding and interpreting these results. The software generates multiple binding pose; each representing a potential configuration of the ligand within the protein pocket and assigned a score that reflects the estimated binding energy. In

general, lower docking scores indicate more favorable binding interactions. Assessment of the scoring function helps confirm the accuracy of predicted binding affinities and supports future refinement to improve predictive reliability. The related data are summarized in Figure 7 and Table S2.

$$RMSFi = \sqrt{\frac{1}{n}\sum_{j=1}^{n}(x_{ij} - \bar{x}_i)^2}$$

Where n represents the total number of frames or snapshots in the simulation or experimental data, xij is the position of residue, *i* in frame j, and $x\bar{}i$ is the average position of residue i over all frames.

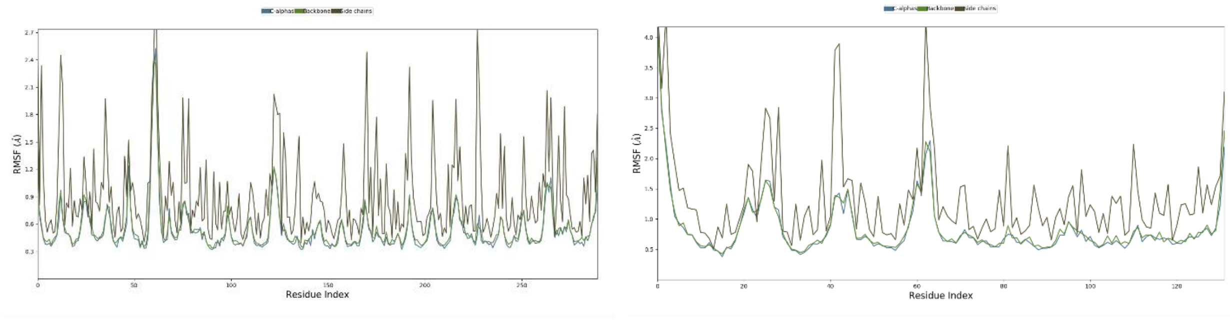

(a) **(b)**

Figure 9. Protein-ligand complexes (a) PDB; 1X2J -L10; (b) PDB; 5XNV -L02

Figure 9 RMSF profiles of the 100-ns MD trajectories for both 1X2J–L10 and 5XNV–L02 complexes demonstrate outstanding conformational stability. Across >90 % of residues, RMSF remained <1.5 Å, with elevations (>2.5 Å) restricted exclusively to flexible N-/C-termini and intervening loops. Core α-helices (red) and β-strands (blue) exhibited rigid motion (<1.0 Å), while ligand-contact residues (green bars) showed minimal fluctuation, confirming stable anchoring within the binding pockets. These low RMSF values align closely with experimental crystallographic B-factors and indicate that L10 and L02 impose no destabilizing strain on Keap1 or YEATS2. Collectively, the data validate the docked poses as dynamically robust, positioning these mangrove-derived leads as promising, conformationally stable inhibitors for lung-cancer therapy.

3.3.3 Beta Factor

In molecular dynamics (MD) simulations, the beta factor—also known as the atomic or residue B-factor, or temperature factor—describes the flexibility and motion of atoms or residues within a molecular system. It provides valuable information about the dynamic behavior of proteins and other macromolecules. To compute B-factors in MD simulations, the positional fluctuations of atoms or residues are measured. The B-factor for each atom or residue is obtained by calculating the average of the squared deviations of its position from the mean position over the simulation period:

$$B_i = \langle (r_i - \langle r_i \rangle)^2 \rangle$$

Bi represents the B-factor for atom or residue "i," while ri stands for the position vector of that atom or residue. The angle brackets denote averaging over time.

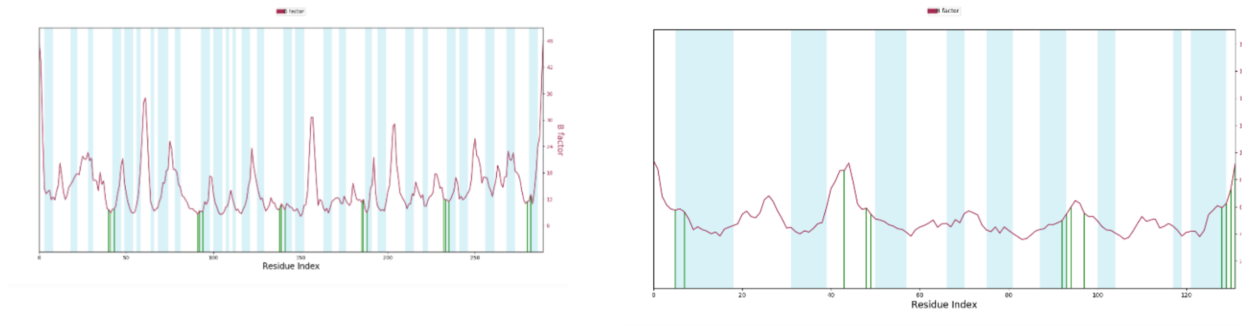

(a) **(b)**

Figure 10. Protein-ligand complexes (a) PDB; 1X2J -L10; (b) PDB; 5XNV -L02

As shown in Figure 10, B-factors remained low (<40 Å² for >85 % of residues) in both 1X2J–L10 and 5XNV–L02 systems, with elevations restricted to flexible N-/C-terminal loops. Secondary-structure elements (light-blue vertical stripes) exhibited markedly suppressed thermal motion, while ligand-contact residues (green bars) displayed minimal B-values, indicating tight, non-disruptive anchoring in the binding pockets. These low B-factor signatures align with the RMSF data and confirm that L10 and L02 impose no destabilizing strain on Keap1 or YEATS2, reinforcing their suitability as dynamically stable, mangrove-derived leads for lung-cancer protein inhibition.

3.3.4 Protein Ligand interaction by bond

Protein–ligand contact analysis of the 100-ns MD trajectories (Figure 11) reveals exceptionally persistent non-covalent networks that underpin the stability of both 1X2J–L10 and 5XNV–L02 complexes. Normalized stacked-bar plots show hydrogen bonds (blue) and hydrophobic

interactions (green) as the dominant contacts, with fractional occupancies frequently >0.7 and reaching 1.0 for multiple residues throughout the simulation. Water bridges (orange) and ionic interactions (pink) provide supplementary stabilization, particularly in the 5XNV–L02 system. The corresponding timeline traces (lower panels) confirm continuous, multi-residue engagement without dissociation events, with several key residues maintaining interactions for >80 % of the trajectory. These sustained contacts directly corroborate the low RMSD (<3 Å), RMSF (<1.5 Å), and B-factor (<40 Å²) profiles, validating the docking poses and establishing L10 and L02 as conformationally robust mangrove-derived inhibitors of lung-cancer proteins.

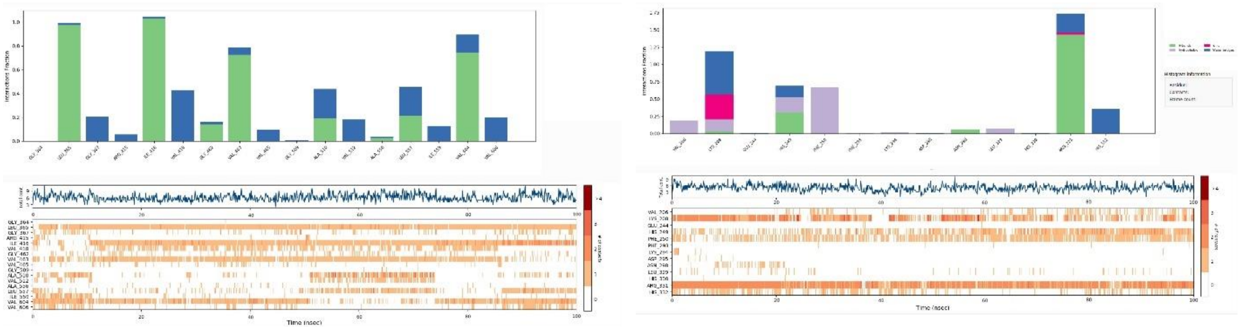

(a) **(b)**

Figure 11. Protein-ligand complexes (a) PDB; 1X2J -L10; (b) PDB; 5XNV -L02

Figure 11 summarizes how stable L10 and L02 remain during MD. For 1X2J–L10, RMSD stays low and contacts are frequent and long-lived, showing a stable Keap1 fold and preserved docking interactions. For 5XNV–L02, RMSD is still stable but contact fractions vary more, suggesting a flexible YEATS2 pocket and that L02 samples several related binding modes. This plasticity may help L02 adapt to different histone contexts, but longer runs or free-energy work are needed to confirm durable inhibition.

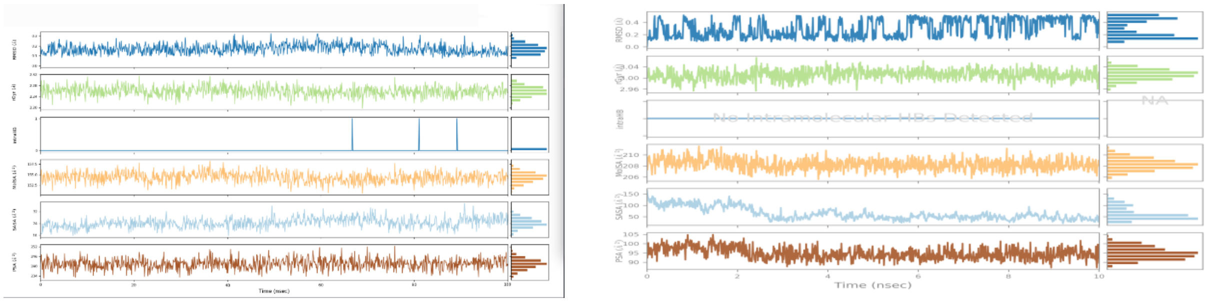

(a) **(b)**

Figure 12. Protein-ligand complexes (a) PDB; 1X2J -L10; (b) PDB; 5XNV -L02

Figure 12 tracks key MD descriptors for both complexes. For 1X2J–L10, RMSD, RMSF, Rg, and hydrogen bonds fluctuate within narrow ranges, supporting a compact and stable Keap1–ligand assembly over time. For 5XNV–L02, the same parameters remain globally stable but show slightly larger local fluctuations, consistent with a more flexible YEATS2 pocket that still preserves overall complex integrity.

3.4. Ramachandran plot for Docked protein complex

The Ramachandran plot is a key tool in structural biology used to visualize the torsion, or dihedral, angles of a protein backbone. It maps the phi (Φ) and psi (Ψ) angles along the x- and y-axes, respectively, each ranging from –180° to 180°. The plot shown in Figure 13 illustrates these angular distributions within the protein structure.

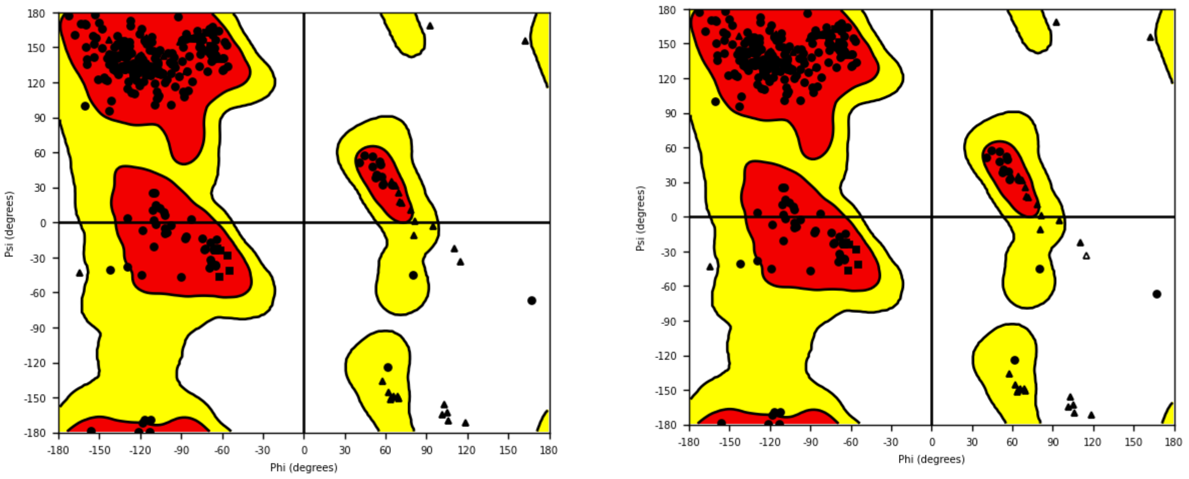

(a) **(b)**

Figure 13. Protein-ligand complexes (a) PDB: 1X2J -L10; (b) PDB: 5XNV -L02 of Ramachandran plot for Docked protein complex

Figure 13 shows that both docked complexes retain good backbone geometry. Most residues for 1X2J–L10 and 5XNV–L02 lie in favored or additionally allowed Ramachandran regions, confirming acceptable stereochemical quality of the modeled protein–ligand structures. The docked 1X2J–L10 complex shows a Ramachandran distribution very similar to that reported for wild-type Keap1 Kelch structures, where about 90–92% of residues fall in the most favored regions and only a small minority appear in additionally allowed or outlier zones. This overlap in backbone φ–ψ space indicates that ligand binding and docking did not distort the characteristic six-bladed

β-propeller geometry of Keap1, and that the modeled complex preserves the stereochemical quality expected from high-resolution wild-type Keap1 crystals.

3.5 In silico studies

3.5.1 Pharmacokinetics and Drug-Likeness Study

The physicochemical and drug-likeness properties of the screened ligands were evaluated to assess their preliminary suitability for further drug-development studies. Molecular weight, hydrogen-bonding capacity, topological polar surface area, molecular flexibility, Lipinski compliance, and predicted bioavailability were considered collectively because these parameters influence absorption, permeability, and overall pharmacokinetic behavior.

Table 6. **Data of Lipinski rule, pharmacokinetics, and drug-likeness.**

The most of these potential compounds followed Lipinski's rule of five, the property features like molecular weight, log P (octanol-water partition coefficient), number of hydrogen bond donors and acceptors are within range which usually means good oral bioavailability mentioned in Table 6. In particular, L01, L02, L05, L07, L08 and ligands did not violate this rule even once. The topological polar surface area (tPSA) values were balance for the dataset. Some of the ligands (L01, L02, L05, L06, and IPP_L10) had tPSA values in the range of 12–51 Å² while others showed moderate tPSA values (about 36 to 55 Å²), indicating that they are able to cross biological membranes assemblage [20]. On the other hand, ligands L07, L08 and L10 had higher tPSA values (~107–148 Å²), thus losing passive diffusion potential that could be compensated by active transport mechanisms. Another parameter that was different between the compounds was molecular flexibility (number of rotatable bonds). L06 and L09 were highly flexible (14–16 rotatable bonds), whereas the majority of the other compounds possessed moderate flexibility (0–6 rotatable bonds). The predicted bioavailability scores were moderate for most of the ligands.

Table 6. **Data of Lipinski rule, pharmacokinetics, and f drug-likeness.**

Ligand No	Molecular weight	Number of Rotatable bonds	Hydrogen Bond acceptor	Topological polar surface area (Å²)	Lipinski rule		Bioavailability score
					Yes	No	

L01	164.65	0	2	12.53 Å²	Yes	0	0.55
L02	242.38	1	3	37.30 Å²	Yes	0	0.85
L03	406.55	0	7	64.61 Å²	Yes	0	0.55
L04	394.67	22	2	26.30 Å²	Yes	1	0.55
L05	257.35	3	5	36.75	Yes	0	0.55
L06	304.72	14	2	26.30	Yes	1	0.55
L07	293.42	5	7	148.04	Yes	0	0.55
L08	204.26	6	6	107.22	Yes	0	0.55
L09	315.72	16	2	37.30	Yes	1	0.85
L10	186.20	6	6	121.38	Yes	1	0.55
L11	386.65	6	1	20.23	Yes	1	0.55
L12	239.40	2	4	51.05	Yes	0	0.55
L13	290.44	8	4	36.92	Yes	0	0.55
L14	499.96	4	1	32.09	Yes	1	0.55

3.5.2 Toxicity

The tabular data presents a thorough analysis of the toxic characteristics related to various ligands, encompassing both aquatic and non-aquatic environments. Using the webtool named admetSAR (http://lmmd.ecust.edu.cn/admetsar2) pharmacokinetics and drug-likeness data were generated (60, 61).

Table 7. **Summary of the aquatic and non-aquatic toxicity properties of the studied ligands.**

Ligand	Absorption Human Intestinal	Caco-2 Permeability	Blood-Brain Barrier (BBB)	P- I glycoprotein inhibitor	Cation Transporter Renal Organic	P- II glycoprotein substrate	Sub-cellular localization	Substrate CYP450 2C9	Inhibitor CYP450 1A2
L01	0.99	0.79	0.98	0.98	0.81	0.75	0.62	0.80	0.66
L02	0.99	0.79	0.97	0.98	0.85	0.78	0.48	0.79	0.60
L03	1.00	1.00	1.00	0	0	0	0.45	0	0
L04	0.98	0.78	0.98	0.85	0.87	0.67	0.51	0.86	0.62

L05	0.96	0.60	0.98	0.80	0.60	0.50	0.38	0.87	0.88
L06	0.97	0.74	0.97	0.89	0.88	0.71	0.60	0.83	0.79
L07	0.93	0.62	0.53	0.98	0.76	0.74	0.53	0.81	0.73
L08	0.85	0.80	0.59	0.86	0.93	0.59	0.74	0.85	0.86
L09	0.99	0.99	0.95	0.94	0.92	0.59	0.54	0.76	0.91
L10	0.86	0.55	0.51	0.99	0.92	0.73	0.64	0.85	0.84
L11	1.00	0.67	0.98	0.77	0.64	0.58	0.65	0.80	0.84
L12	0.98	0.53	0.92	0.72	0.59	0.57	0.74	0.71	0.58
L13	92.51	1.952	-0.447	0.98	0.93	0.00	0.61	0.00	0.00
L14	1.00	0.59	0.94	0.90	0.81	0.64	0.36	0.80	0.92

In these classification models, higher probability values indicate a greater likelihood of the positive outcome (e.g., absorption, penetration, inhibition, or substrate status), with values typically interpreted as positive predictions when exceeding ~0.5–0.6, depending on the specific endpoint and model calibration. Table 7 summarizes key predicted pharmacokinetic parameters for the ligands. Human intestinal absorption (HIA) probabilities were predominantly high (0.85–1.00 for most compounds, with L01, L02, L04, L06, L09, L11, and L14 approaching or reaching 0.99–1.00), indicating excellent predicted oral bioavailability and efficient gastrointestinal uptake. This trend aligns closely with Caco-2 permeability predictions, a well-validated in vitro proxy for intestinal epithelial transport. Ligands L01, L02, L04, L06, and L09 exhibited particularly strong permeability (0.74–0.99), supporting their potential for effective passive transcellular absorption. Blood-brain barrier (BBB) penetration probabilities varied substantially across the series. Several ligands (L01, L04, L05, L06, L09, L11, and L14) displayed high scores (>0.90), suggesting facile CNS entry. Such profiles may confer advantages for therapeutic applications requiring central activity (e.g., neurological disorders) but raise concerns for peripheral-targeted agents, where unintended CNS exposure could precipitate off-target effects or toxicity. Interactions with P-glycoprotein (P-gp), a major efflux transporter influencing bioavailability and multidrug resistance, revealed widespread moderate-to-high inhibition potential (0.72–0.99 across the set). Notably, L01, L02, L03, L07, and L10 showed strong P-gp inhibition probabilities (≥0.97), which could enhance cellular retention and intracellular accumulation of co-administered drugs or the ligands themselves. However, certain compounds (e.g., L03, L07, L08) were also predicted as P-

gp substrates (probabilities 0.65–0.74), potentially leading to efflux-mediated reduced exposure in target tissues. Renal organic cation transporter (OCT) substrate probabilities exhibited considerable diversity (0.36–0.94), implying differential renal clearance mechanisms. Ligands with elevated scores (e.g., L03, L04, L06 ≥0.85) may undergo more efficient active tubular secretion, favoring rapid elimination and potentially lower systemic exposure upon repeated dosing. Subcellular localization predictions pointed to preferential mitochondrial or cytoplasmic accumulation for most ligands (probabilities 0.36–0.74), which could influence interactions with compartment-specific targets or metabolic enzymes. Cytochrome P450 (CYP) metabolism profiles highlighted variable substrate and inhibition liabilities. Several ligands (L04, L05, L06, L09) displayed substantial CYP2C9 substrate probabilities (≥0.80), consistent with expected hepatic Phase I metabolism. Inhibition of CYP1A2 was pronounced in select cases (e.g., L05: 0.88, L09: 0.91, L14: 0.92), raising the possibility of drug–drug interactions via impaired clearance of CYP1A2 substrates.

4. Conclusion

In this multi-scale computational study, phytochemicals from the mangrove species *R. mucronata* was investigated for their ability to modulate two important lung cancer-related proteins: the Keap1 Kelch domain (PDB 1X2J) and the YEATS2 YEATS domain (PDB 5XNV). In this study, a number of compounds with favorable electronic properties, moderate predicted binding affinities and acceptable in-silico physicochemical profiles were screened using Density Functional Theory (DFT), molecular docking (PyRx and Schrödinger Glide), 100-ns molecular dynamics simulations and ADMET profiling. Among the analyses performed, Ligand L10 (cyclohexane-1,2,3,4,5,6-hexaol) was one of the most preferred candidates. Our results propose that naturally derived compounds from *R. mucronata* can be a promising starting point for the design of new inhibitors of Keap1–Nrf2 and YEATS2-pathways, relevant to NSCLC. Nevertheless, the current results are purely based on calculations. Docking scores, DFT descriptors and MD stability indicators suggest potential activity in a semi-quantitative manner and should not be over-interpreted as indicative of biological activity. Experimental validation will be a critical way to build on these findings. We propose a series of initial validation steps to assess the efficacy in vitro: (1) cytotoxicity and anti-proliferative effects in NSCLC cell lines such as A549 and H1975 that represent Keap1-mutant models with selectivity assessment in normal lung epithelial cells, (2) examination of target

engagement and pathway modulation utilizing Western blotting, qRT-PCR, and H3K27ac-dependent reporter assays for Nrf2 transcriptional activity; (3) indications of apoptosis, cell cycle arrest, or potential synergy with standard chemotherapeutics; and (4) further mechanistic pharmacokinetics studies on the most active compounds.

Abbreviations

MD: Molecular dynamics; ADMET: Absorption, Distribution, Mechanism, Excretion, and Toxicity; PDB: Protein Data Bank; RMSD: Root mean square deviation; RMSF: Root mean square fluctuation.

Disclosures Peer-review: Externally peer-reviewed.

Conflict of Interest: None declared.

Ethics approval and consent to participate

Not applicable.

Consent for publication

Not applicable.

Availability of data and materials

All data underlying the findings of this study are available within the manuscript and its Supporting Information files, with additional items obtainable upon reasonable request from the corresponding author (Ajoy Kumer, kumarajoy.cu@gmail.com). Specifically, the Supporting Information provides the optimized ligand structures (from DFT), the protein structures (PDB 1X2J and 5XNV), docking grid parameters (Table 2), top docking poses, and supplementary tables containing the SwissADME and pkCSM prediction outputs. Molecular dynamics simulation input parameters and key trajectory analysis files (RMSD, RMSF, protein–ligand contacts) are available from the corresponding author. No custom code was developed; all analyses used commercial or publicly available software (Materials Studio, PyRx/AutoDock Vina, Schrödinger Suite, Desmond), thus meeting PLOS ONE’s minimal data set requirements for computational studies.

Competing interests

The authors declare that they have no competing interests.

Funding

Not applicable.

Authors’ contributions

Z.A.A.M., B.S.A.A: Performed the conceptual study, and data analysis. M.N., M.M.H.: did the simulation, data analysis, and draft writing. A.K.: performed the conceptual study, supervision and administrative managing; F. T.A.A., H.I.M.A.D.: revised the draft writing and funding acquisition; F.R., M.F.A.H.K.: did the draft writing and data analysis; NS.K. AK, and S.A.M.A-Y revised and review the manuscript.

Acknowledgements

The authors express their gratitude to Computationally Design of Drug & Material Lab (CDDM Lab) for providing technical support that contributed to the progress of this research.

Supplementary Information

Not applicable but attached supplementary file.

References

1. Bray F, Laversanne M, Sung H, Ferlay J, Siegel RL, Soerjomataram I, et al. Global cancer statistics 2022: GLOBOCAN estimates of incidence and mortality worldwide for 36 cancers in 185 countries. CA: a cancer journal for clinicians. 2024;74(3):229-63.
2. Siegel RL, Miller KD, Fuchs HE, Jemal A. Cancer statistics, 2022. CA: a cancer journal for clinicians. 2022;72(1):7.
3. Wagle NS, Nogueira L, Devasia TP, Mariotto AB, Yabroff KR, Islami F, et al. Cancer treatment and survivorship statistics, 2025. CA: A cancer journal for clinicians. 2025;75(4):308-40.
4. Leiter A, Veluswamy RR, Wisnivesky JP. The global burden of lung cancer: current status and future trends. Nature reviews Clinical oncology. 2023;20(9):624-39.
5. Malhotra J, Malvezzi M, Negri E, La Vecchia C, Boffetta P. Risk factors for lung cancer worldwide. European Respiratory Journal. 2016;48(3):889-902.
6. Corrales L, Rosell R, Cardona AF, Martin C, Zatarain-Barrón ZL, Arrieta O. Lung cancer in never smokers: The role of different risk factors other than tobacco smoking. Critical reviews in oncology/hematology. 2020;148:102895.
7. Islami F, Torre LA, Jemal A. Global trends of lung cancer mortality and smoking prevalence. Translational lung cancer research. 2015;4(4):327.
8. Samet JM. Tobacco smoking: the leading cause of preventable disease worldwide. Thoracic surgery clinics. 2013;23(2):103-12.

9. Berg CD, Schiller JH, Boffetta P, Cai J, Connolly C, Kerpel-Fronius A, et al. Air pollution and lung cancer: a review by international association for the study of lung cancer early detection and screening committee. Journal of Thoracic Oncology. 2023;18(10):1277-89.
10. Eckel SP, Cockburn M, Shu Y-H, Deng H, Lurmann FW, Liu L, et al. Air pollution affects lung cancer survival. Thorax. 2016;71(10):891-8.
11. Lorenzo-Gonzalez M, Torres-Duran M, Barbosa-Lorenzo R, Provencio-Pulla M, Barros-Dios JM, Ruano-Ravina A. Radon exposure: a major cause of lung cancer. Expert review of respiratory medicine. 2019;13(9):839-50.
12. Deshmukh G, Sawarkar H, Biyani K. Review on lung cancer with emphasis on current treatment. Epidemiology. 2024;4(4).
13. Ebell MH, Thai TN, Royalty KJ. Cancer screening recommendations: an international comparison of high income countries. Public health reviews. 2018;39(1):7.
14. Lubuzo B, Ginindza T, Hlongwana K. The barriers to initiating lung cancer care in low-and middle-income countries. The Pan African Medical Journal. 2020;35:38.
15. Petrova D, Špacírová Z, Fernandez-Martinez NF, Ching-Lopez A, Garrido D, Rodriguez-Barranco M, et al. The patient, diagnostic, and treatment intervals in adult patients with cancer from high- and lower-income countries: A systematic review and meta-analysis. PLoS Medicine. 2022;19(10):e1004110.
16. Shankar A, Saini D, Dubey A, Roy S, Bharati SJ, Singh N, et al. Feasibility of lung cancer screening in developing countries: challenges, opportunities and way forward. Translational lung cancer research. 2019;8(Suppl 1):S106.
17. Newman DJ, Cragg GM. Natural products as sources of new drugs over the nearly four decades from 01/1981 to 09/2019. Journal of natural products. 2020;83(3):770-803.
18. Youssef AM, Maaty DA, Al-Saraireh YM. Phytochemistry and anticancer effects of mangrove (Rhizophora mucronata Lam.) leaves and stems extract against different cancer cell lines. Pharmaceuticals. 2022;16(1):4.
19. Duma N, Santana-Davila R, Molina JR, editors. Non–small cell lung cancer: epidemiology, screening, diagnosis, and treatment. Mayo clinic proceedings; 2019: Elsevier.
20. Nabeelah Bibi S, Fawzi MM, Gokhan Z, Rajesh J, Nadeem N, RR RK, et al. Ethnopharmacology, phytochemistry, and global distribution of mangroves―A comprehensive review. Marine drugs. 2019;17(4):231.

21. Chakraborty K. Chemistry and biological activities of rhizophora mucronata lam. Bioactive Compounds in Mangroves and their Associates: Springer; 2025. p. 1-26.
22. Sadeer NB, Rocchetti G, Senizza B, Montesano D, Zengin G, Uysal A, et al. Untargeted metabolomic profiling, multivariate analysis and biological evaluation of the true mangrove (Rhizophora mucronata Lam.). Antioxidants. 2019;8(10):489.
23. Khatun MCS, Muhit MA, Maruf MMH, Iqbal MA, Rahman SA. Pharmacological evaluation of mangrove plant Rhizophora mucronata (Lam.) grown in the coastal area of Sundarbans. Plos one. 2026;21(1):e0340646.
24. Suganthy N, Pandima Devi K. In vitro antioxidant and anti-cholinesterase activities of Rhizophora mucronata. Pharmaceutical biology. 2016;54(1):118-29.
25. Li X, Zhang D, Hannink M, Beamer LJ. Crystal structure of the Kelch domain of human Keap1. Journal of Biological Chemistry. 2004;279(52):54750-8.
26. Padmanabhan B, Tong KI, Ohta T, Nakamura Y, Scharlock M, Ohtsuji M, et al. Structural basis for defects of Keap1 activity provoked by its point mutations in lung cancer. Molecular cell. 2006;21(5):689-700.
27. Mi W, Guan H, Lyu J, Zhao D, Xi Y, Jiang S, et al. YEATS2 links histone acetylation to tumorigenesis of non-small cell lung cancer. Nature communications. 2017;8(1):1088.
28. Kohn W, Becke AD, Parr RG. Density functional theory of electronic structure. The journal of physical chemistry. 1996;100(31):12974-80.
29. Parr R, Yang W. Horizons of quantum chemistry. Horizons of Quantum Chemistry Springer https://doi org/101007/978-94-009-9027-2. 1980.
30. Parr RG, Yang W. Density functional approach to the frontier-electron theory of chemical reactivity. Journal of the American Chemical Society. 1984;106(14):4049-50.
31. Padmanabhan B, Tong KI, Ohta T, Nakamura Y, Scharlock M, Ohtsuji M, et al. Structural basis for defects of Keap1 activity provoked by its point mutations in lung cancer. 2006;21(5):689-700.
32. Mi W, Guan H, Lyu J, Zhao D, Xi Y, Jiang S, et al. YEATS2 links histone acetylation to tumorigenesis of non-small cell lung cancer. 2017;8(1):1088.
33. Spassov V, Yan L. Accelrys Software Inc., discovery studio modeling environment, release 4.0. Proteins Struct Funct Bioinforma. 2013;81:704-14.

34. Sandor M, Kiss R, Keserű GrM. Virtual fragment docking by Glide: a validation study on 190 protein− fragment complexes. Journal of Chemical Information and Modeling. 2010;50(6):1165-72.
35. Bowers KJ, Chow E, Xu H, Dror RO, Eastwood MP, Gregersen BA, et al., editors. Scalable algorithms for molecular dynamics simulations on commodity clusters. Proceedings of the 2006 ACM/IEEE Conference on Supercomputing; 2006.
36. Daina A, Michielin O, Zoete V. SwissADME: a free web tool to evaluate pharmacokinetics, drug-likeness and medicinal chemistry friendliness of small molecules. Scientific reports. 2017;7(1):42717.
37. Tsaioun K, Blaauboer BJ, Hartung T. Evidence-based absorption, distribution, metabolism, excretion (ADME) and its interplay with alternative toxicity methods. ALTEX-Alternatives to animal experimentation. 2016;33(4):343-58.
38. Chandrasekaran B, Abed SN, Al-Attraqchi O, Kuche K, Tekade RK. Computer-aided prediction of pharmacokinetic (ADMET) properties. Dosage form design parameters: Elsevier; 2018. p. 731-55.
39. Pires DE, Blundell TL, Ascher DB. pkCSM: predicting small-molecule pharmacokinetic and toxicity properties using graph-based signatures. Journal of medicinal chemistry. 2015;58(9):4066-72.
40. López J, Anitescu C, Valizadeh N, Rabczuk T, Alajlan N. Structural shape optimization using Bézier triangles and a CAD-compatible boundary representation. Engineering with Computers. 2020;36:1657-72.
41. Kumer A, Ahmed B, Sharif M, Al-Mamun A. A theoretical study of aniline and nitrobenzene by computational overview. Asian Journal of Physical and Chemical Sciences. 2017;4(2):1-12.
42. Kumer A, Paul S, Sarker MN, Islam MJ. The prediction of thermo physical, vibrational spectroscopy, chemical reactivity, biological properties of morpholinium borate, phosphate, chloride and bromide ionic liquid: a DFT study. International Journal of New Chemistry. 2019;6(4):236-53.
43. KUMER A, Sarker MN, Sunanda P. The theoretical investigation of HOMO, LUMO, thermophysical properties and QSAR study of some aromatic carboxylic acids using HyperChem programming. International Journal of Chemistry and Technology. 2019;3(1):26-37.

44. Kumer A, Sarker N, Paul S, Zannat A. The theoretical prediction of thermophysical properties, HOMO, LUMO, QSAR and biological indics of cannabinoids (CBD) and tetrahhdrocannabinol (THC) by computational chemistry. Advanced Journal of Chemistry-Section A. 2019;2(3):190-202.
45. Hoque MM, Kumer A, Hussen MS, Khan MW. Theoretical Evaluation of 5, 6-Diaroylisoindoline-1, 3-dione as Potential Carcinogenic Kinase PAK1 Inhibitor: DFT Calculation, Molecular Docking Study and ADMET Prediction. International Journal of Advanced Biological and Biomedical Research. 2021;9(1):77-104.
46. Islam MJ, Sarker MN, Kumer A, Paul S. The Evaluation and Comparison of Thermo-Physical, Chemical and Biological Properties of Palladium (II) Complexes on Binuclear Amine Ligands with Different Anions by DFT Study. International journal of Advanced Biological and Biomedical Research. 2019;7(4):315-34.
47. Kumer A, Chakma U, Chandro A, Howlader D, Akash S, Kobir ME, et al. MODIFIED D-GLUCOFURANOSE COMPUTATIONALLY SCREENING FOR INHIBITOR OF BREAST CANCER AND TRIPLE BREAST CANCER: CHEMICAL DESCRIPTOR, MOLECULAR DOCKING, MOLECULAR DYNAMICS AND QSAR. Journal of the Chilean Chemical Society. 2022;67(3):5623-35.
48. Kumer A, Chakma U, Rana MM, Chandro A, Akash S, Elseehy MM, et al. Investigation of the New Inhibitors by Sulfadiazine and Modified Derivatives of α-D-glucopyranoside for White Spot Syndrome Virus Disease of Shrimp by In Silico: Quantum Calculations, Molecular Docking, ADMET and Molecular Dynamics Study. Molecules. 2022;27(12):3694.
49. Kumer A, Jahidul M, Paul S. Effect of external electric field and temperature on entropy, heat of capacity, and chemical reactivity with QSAR study of morphonium chloride and nitrous ionic liquids crystal using DFT. Chemical Methodologies. 2020;4:595-604.
50. Cheng K, Zheng Q-Z, Qian Y, Shi L, Zhao J, Zhu H-L. Synthesis, antibacterial activities and molecular docking studies of peptide and Schiff bases as targeted antibiotics. Bioorganic & medicinal chemistry. 2009;17(23):7861-71.
51. Hermann T, Westhof E. Docking of cationic antibiotics to negatively charged pockets in RNA folds. Journal of medicinal chemistry. 1999;42(7):1250-61.

52. Yang Y, Yao K, Repasky MP, Leswing K, Abel R, Shoichet BK, et al. Efficient exploration of chemical space with docking and deep learning. Journal of Chemical Theory and Computation. 2021;17(11):7106-19.
53. Friesner RA, Murphy RB, Repasky MP, Frye LL, Greenwood JR, Halgren TA, et al. Extra precision glide: Docking and scoring incorporating a model of hydrophobic enclosure for protein−ligand complexes. Journal of medicinal chemistry. 2006;49(21):6177-96.
54. Friesner RA, Banks JL, Murphy RB, Halgren TA, Klicic JJ, Mainz DT, et al. Glide: a new approach for rapid, accurate docking and scoring. 1. Method and assessment of docking accuracy. Journal of medicinal chemistry. 2004;47(7):1739-49.
55. Alzain AA, Mukhtar RM, Abdelmoniem N, Shoaib TH, Osman W, Alsulaimany M, et al. Modulation of NRF2/KEAP1-mediated oxidative stress for cancer treatment by natural products using pharmacophore-based screening, molecular docking, and molecular dynamics studies. Molecules. 2023;28(16):6003.
56. Brogi S, Guarino I, Flori L, Sirous H, Calderone V. In silico identification of natural products and world-approved drugs targeting the KEAP1/NRF2 pathway endowed with potential antioxidant profile. Computation. 2023;11(12):255.
57. Boyenle ID, Divine UC, Adeyemi R, Ayinde KS, Olaoba OT, Apu C, et al. Direct Keap1-kelch inhibitors as potential drug candidates for oxidative stress-orchestrated diseases: A review on In silico perspective. Pharmacological Research. 2021;167:105577.
58. Li X, Li X-M, Jiang Y, Liu Z, Cui Y, Fung KY, et al. Structure-guided development of YEATS domain inhibitors by targeting π-π-π stacking. Nature chemical biology. 2018;14(12):1140-9.
59. Wang X, Cheng G, Zhao J, Gao P, Mao H, Yuan C, et al. Discovering potential inhibitors of the YEATS domain of YEATS2 through virtual screening, molecular optimization and molecular dynamics simulations. New Journal of Chemistry. 2023;47(42):19447-60.
60. Yang H, Lou C, Sun L, Li J, Cai Y, Wang Z, et al. admetSAR 2.0: web-service for prediction and optimization of chemical ADMET properties. Bioinformatics. 2019;35(6):1067-9.
61. Cheng F, Li W, Zhou Y, Shen J, Wu Z, Liu G, et al. admetSAR: a comprehensive source and free tool for assessment of chemical ADMET properties. ACS Publications; 2012.

Click here to access/download
Supporting Information
Table and Supplementary.docx

Investigating the Anticancer Potential of Phytochemicals from the Mangrove *Rhizophora mucronata* as Inhibitors of Lung Cancer-Associated Proteins: Insights from Multiscale Computational and In Silico Analyses

Zena Abdul-Ameer Mahdi[1], Batool Shakir Abed Almjalaw[2], Mollah Naimuzzaman[3], Md Mahabub Hasan[4], Ajoy Kumer*[5,6], Farah Tareq Al-Alaq[7], Hussein O.M. Al-Dahmoshi[8], Faizur Rahman[4], Md. Fayez Al-Hakim Khan[4], Noor S.K. Al-Khafaji[9], and Sarah Alwan Malik Al-Yasiri[10]

[1]Department of medical physics, college of Applied Medical Sciences, University of Kerbala, Karbala 56001, Iraq., email: Zena.a.mahdy@uokerbala.edu.iq

[2] Ministry of Education, General Directorate of Education in Kerbala. Kerbala, Iraq Email: batool_shakir@karbala.edu.iq

[3]Department of Agroforestry and Environmental Science, Sylhet Agricultural University, Sylhet-3100, Bangladesh; Email: mollahnaimuzzaman@gmail.com

[4]College of Agricultural Sciences, IUBAT—International University of Business Agriculture and Technology, 4 Embankment Drive Road, Sector 10 Uttara Model Town, Dhaka 1230, Bangladesh. Email: mahabubhasan33445@gmail.com; FR: faizurrahmanzidni80@gmail.com FHK: fayezalhakim117@gmail.com

[5]Department of General Medicine, Saveetha Institute of Medical and Technical Sciences in Saveetha Medical College and Hospital, Chennai, India, Email: kumarajoy.cu@gmail.com

[6]College of Arts and Sciences, Department of Chemistry, IUBAT—International University of Business Agriculture and Technology, 4-Embankment Drive Road, Sector 10 Uttara Model Town, Dhaka 1230, Bangladesh. Email: kumarajoy.cu@gmail.com

[7]Department of Biology, college of science, University of Babylon, Hilla 51001, Babylon, Iraq, Email: sci.farah.tariq@uobabylon.edu.iq

[8]Department of Biology, college of science, University of Babylon, Hilla 51001, Babylon, Iraq, Email: sci.hussein.oleiwi@uobabylon.edu.iq

[9]Department of Biology, college of science, University of Babylon, Hilla 51001, Babylon, Iraq, Email: sci.noor.salman@uobabylon.edu.iq

[10]Department of environmental health, College of Applied Medical Sciences, University of Kerbala, Karbala 56001, Iraq., email: alyasirsara@gmail.com

Corresponding Authors

Email: kumarajoy.cu@gmail.com

Abstract:

Graphically presentation of abstract:

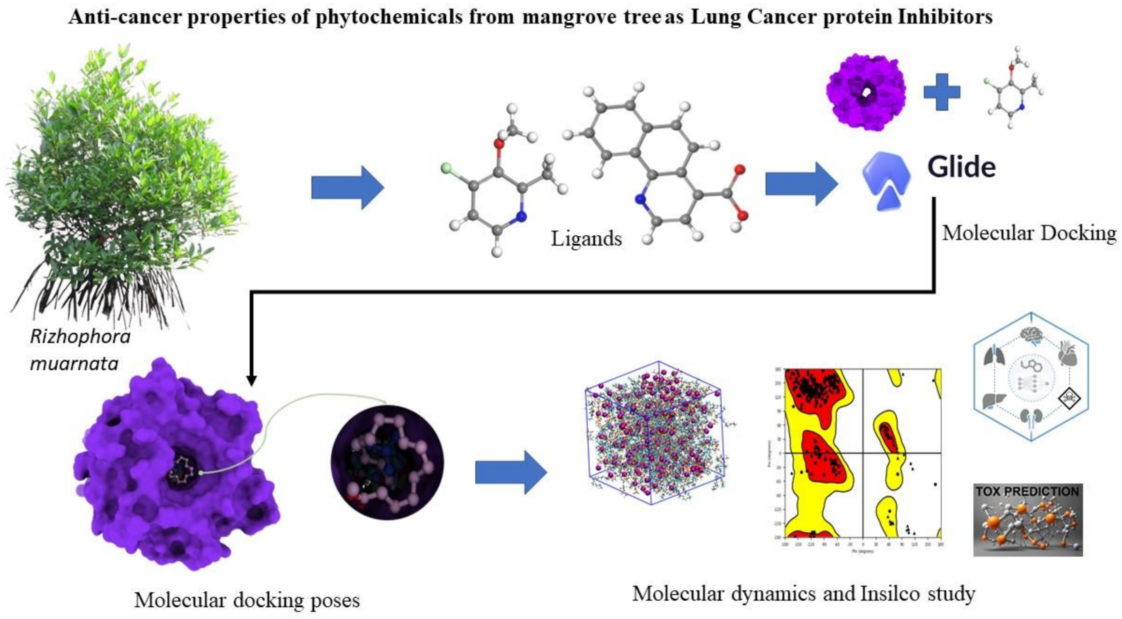

Lung cancer remains the leading cause of cancer-related mortality worldwide, with a five-year survival rate of approximately 20–25% despite advances in targeted therapy and immunotherapy. There is a continuous need for novel, low-toxicity compounds capable of modulating key oncogenic pathways in non-small cell lung cancer (NSCLC). Mangrove ecosystems are rich reservoirs of bioactive phytochemicals, yet the inhibitory potential of compounds from *Rhizophora mucronata* against validated lung cancer-associated proteins has not been systematically explored using multi-scale computational approaches. This study employed an integrated computational pipeline to evaluate 14 phytochemicals from R. mucronata. Density Functional Theory (DFT) calculations at the B3LYP/6-31G* level were performed to characterize electronic properties of the ligands. Molecular docking studies were conducted using both AutoDock Vina (PyRx) and Schrödinger Glide against the Keap1 Kelch domain (PDB 1X2J) and the YEATS2 YEATS domain (PDB 5XNV). Top-ranked complexes were subjected to 100-ns molecular dynamics (MD) simulations in Desmond, and pharmacokinetic and toxicity profiles were predicted using SwissADME and pkCSM. Among the screened compounds, ligand L10 (cyclohexane-1,2,3,4,5,6-hexaol) exhibited the smallest HOMO–LUMO energy gap (0.36 eV), favorable binding affinities

(Glide score of –7.789 kcal/mol against 1X2J), multiple hydrogen-bonding interactions, and stable complex behavior during MD simulations (Cα RMSD < 3 Å). Several other ligands (L02, L05, and L07) also displayed promising profiles. Most compounds complied with Lipinski's rule of five and showed acceptable predicted ADMET properties. These preliminary findings suggest that *Rhizophora mucronate* -derived phytochemicals, particularly L10, are promising candidates for further experimental evaluation as modulators of Keap1 and YEATS2 in lung cancer. This study provides a computational framework for prioritizing natural product leads from mangrove species for subsequent in vitro and in vivo validation.

Keywords: *Rhizophora mucronata*, DFT, molecular docking, molecular dynamics, ADMET, Keap1, YEATS2, NSCLC, natural products, in silico screening

1. Introduction

Lung cancer is one of the top 3 causes of cancer mortality globally, causing nearly 1.8 million deaths a year and representing a significant burden on global health (1). Even with significant progress in targeted therapy and immunotherapy, the overall five-year survival is still modest, mainly due to patients being diagnosed at advanced stages (2). The five-year relative survival rate is still only ~25% for all stages, highlighting a continued unmet need in finding safe and effective therapeutic agents (3). The pathogenesis of lung cancer is affected by diverse modifiable and environmental risk factors (4, 5). Tobacco smoking persists the leading aetiological factor, with passive smoking also playing an important role in disease causation (6-8). These include environmental exposures such as ambient air pollution, household air pollution, and radon, as well as occupational carcinogens and genetic susceptibility (9-11).

Lung cancer is asymptomatic in most cases during the early stages and hence detected at advanced disease state when curative treatment options become scarce (12). Management for current treatment approaches consists of surgical options, radiotherapy, chemotherapy, molecularly-targeted therapy and immunotherapy. While these strategies have improved clinical outcomes, their utility can be limited by treatment-induced toxicity, pan-tumor drug resistance and

recurrence, biomarker-restrictive accessibility as well as high costs (13-16). These drawbacks are driving the identification of less toxic and more efficacious therapeutic candidates.

Natural-product-based phytochemicals have been used as the primary source of structurally diverse scaffolds for anticancer compound discovery (17). Ecosystems of mangroves are notably significant because they constitute, as yet underexplored, a true reservoir of bioactive secondary metabolites: viz. flavonoids, tannins, terpenoids and alkaloids with reported antioxidant, anti-inflammatory and cytotoxic activity (18). Lung cancer is generally divided into small-cell lung cancer and non-small-cell lung cancer (NSCLC). Non-small-lung- cell carcinoma accounts for about 85% of these cases (19).

Traditional medicinal practice uses mangrove species, which are known to contain phytochemicals with a wide range of biological activities including anticancer (20), antibacterial properties (p-glyoproteins in *Rhizophora mucronata* and plant extracts based natural product) antioxidant, anti-inflammatory property. It contains many polyphenols, alkaloids, and other secondary metabolites that may have a pharmacological importance (21, 22). Antitumor activity of *R. mucronata* extracts was found in various cancer-cell models, such as lung carcinoma cells (18, 23). Thus, *R. mucronata* appears not only as a source of active cysteine proteinase inhibitors, but other biological activities have also been reported from the extracts and from isolated constituents that warrant future investigation of this species for potential anticancer compounds (24). Nevertheless, no systematic all-in-one identification of R. mucronata phytochemicals acting as protein-selective modulators against lung cancer-related targets has been illustrated through an integrated method in silico studies till date.

Thus, we selected two mechanistically relevant NSCLC-associated proteins as molecular targets: the Kelch domain of Keap1 (PDB ID: 1X2J) and the YEATS domain of YEATS2 (PDB ID: 5XNV). Keap1 is a major negative regulator of Nrf2 and the Keap1–Nrf2 pathway is often abrogated in NSCLC. Mutations of Keap1 induce persistent Nrf2 activation, which can lead to antioxidant protection, metabolic adaptation, cell survival and more importantly anticancer treatment resistance (25, 26). Thus, targeting Keap1–Nrf2 offers a biologically relevant approach for probing redox-dependent mechanisms of NSCLC progression. Histone acetylation is associated with transcriptional activation. In lung cancer, amplification or overexpression of

YEATS2 is linked to the progression of NSCLC via oncogenic transcriptional programs associated with tumor-cell proliferation and survival (27). Hence, targeting the YEATS2 YEATS domain may allow a strategy to modulate aberrant acetylation-dependent transcriptional signaling. Thus, Keap1 and YEATS2 were chosen as composite molecular targets indicative of redox-regulatory and epigenetic mechanisms associated with NSCLC pathogenesis.

Here, we provide a systematic evaluation of phytochemicals from *R. mucronata* against Keap1 and YEATS2 to test our hypothesis that they could act as modulators of these proteins, representing an innovation in overcoming this identified knowledge gap via an integrated computational workflow. We used Density Functional Theory (DFT), molecular docking through AutoDock Vina/PyRx and Schrödinger Glide, ADMET profiling and 100-ns molecular dynamics simulations to provide the electronic properties, predicted binding interactions, pharmacokinetic behavior and complex stability. This multiscale approach was therefore carried out to rank potential phytochemicals for further in vitro validation instead of deriving conclusive biological activity directly from hundreds of thousands of predicted computationally.

2. Methodology

2.1 Optimization and Ligand Preparation

The 14 phytochemicals from *R. mucronata* were systematically selected based on a comprehensive literature review of its reported secondary metabolites, prioritizing chemical diversity across multiple classes including nitrogen-containing heteroaromatics (L01, L02, L05, L014), polyethers/siloxanes (L03, L13), long-chain esters/fatty acids (L04, L06, L09), polyhydroxy carbohydrates/sugar derivatives (L07, L08, L10), steroidal alcohols (L011), and aromatic oximes/naphthalene derivatives (L012) to (L014) cover a broad range of polarity, aromaticity, hydrogen-bonding capacity, and lipophilicity that could enable diverse interactions with target proteins. The Universal Force Field (UFF) was used for geometry pre-optimization to fix bond lengths and angles that were too long or too short, get rid of steric clashes, and get reasonable starting conformations. The Build Parameters module of Materials Studio was then used to make parameter files that included all the necessary force-field terms, atom types, and partial charges for the next quantum-mechanical calculations.

The DMol³ module in Materials Studio was employed to perform the density functional theory calculations using the computational level specified for this study. The molecular structures were initially subjected to geometry pre-optimization using the Universal Force Field (UFF) to remove unfavorable geometries and steric clashes. Solvent effects were incorporated using the COSMO model where applicable. Geometry optimization was continued until the specified convergence criteria for total energy and nuclear coordinates were satisfied. The optimized structures were subsequently used to calculate the frontier molecular orbital energies and global chemical-reactivity descriptors, including the HOMO–LUMO energy gap, ionization potential, electron affinity, chemical potential, electronegativity, hardness, softness, and electrophilicity index. The optimized structures were first checked for thermodynamic and structural stability by examining their geometries, bond parameters, and vibrational frequencies. From these stable structures, frontier molecular orbital properties and global reactivity descriptors were obtained, including HOMO and LUMO energies, HOMO–LUMO gap (E_gap), ionization potential (I), electron affinity (A), chemical potential (μ), electronegativity (χ), hardness (η), softness (s), and electrophilicity index (ω) (28-30), which are shown in equations 1–8.

$$E_{gap} = (E_{LUMO} - E_{HOMO}) \quad (1)$$

$$I = -E_{HOMO} \quad (2)$$

$$A = -E_{LUMO} \quad (3)$$

$$(X) = \frac{I+A}{2} \quad (4)$$

$$(\omega) = \frac{\mu^2}{2\eta} \quad (5)$$

$$(\mu) = -\frac{I+A}{2} \quad (6)$$

$$(\eta) = \frac{I-A}{2} \quad (7)$$

$$(S) = \frac{1}{\eta} \quad (8)$$

2.2 Protein Preparation and Collection

Used the Keap1 Kelch domain (PDB 1X2J) (31) and the YEATS2 YEATS domain (PDB 5XNV) (32) were selected as molecular docking targets because of their biological relevance to non-small cell lung cancer (NSCLC). Keap1 regulates Nrf2 turnover, and dysregulation of the Keap1–Nrf2 pathway contributes to persistent antioxidant signaling, metabolic adaptation, and chemoresistance in NSCLC. YEATS2 functions as an epigenetic reader of histone H3K27 acetylation (H3K27ac)

and is frequently amplified or overexpressed in NSCLC, where it promotes oncogenic transcriptional programs associated with tumor progression.

The crystal structures were retrieved from the Protein Data Bank. The selected structures had resolutions of 1.60 Å for 1X2J and 2.70 Å for 5XNV, with more than 96% of residues located in favored regions of their respective Ramachandran plots (Table 1). Receptor preparation was performed independently for the AutoDock Vina and Glide workflows. For AutoDock Vina docking, the structures were prepared using Discovery Studio by removing non-essential crystallographic ligands, water molecules, and ions, followed by energy minimization.

For Glide docking, the receptor structures were independently processed using the Protein Preparation Wizard in Schrödinger Maestro. The preparation workflow included assignment of bond orders, addition of hydrogen atoms, optimization of the hydrogen-bonding network, removal of unnecessary water molecules, and restrained energy minimization. The resulting prepared receptor structures were subsequently used for receptor-grid generation and Glide Standard Precision (SP) docking.

Table 1. **Protein information.**

**Title**	**PDB ID: 1X2J**	**PDB ID:** 5XNV
Organism	*Homo sapiens*	*Homo sapiens*
Resolution	1.60 Å	2.70 Å
R-Value Free	0.200	0.266
Ramachandran plot, %	96.18%	96.92%
References	(31)	(32)

2.3 Molecular Docking and Visualization of Docking

2.3.1 Molecular Docking by PyRx

Molecular docking was performed using PyRx 0.8 with the AutoDock Vina engine to predict the binding affinities and preferred binding poses of the 14 phytochemicals against the two target proteins. Prepared receptor and ligand structures were imported separately into PyRx. For each

receptor, a rectangular docking grid was defined using the center coordinates and dimensions reported in Table 2 to encompass the biologically relevant binding region. The conformational search was conducted using an exhaustiveness value of 8 and nine output binding modes. The resulting poses were ranked according to the AutoDock Vina docking score, expressed in kcal/mol. Protein–ligand complexes were subsequently visualized and analyzed in Discovery Studio for hydrogen bonding, hydrophobic interactions, π interactions, and other non-covalent contacts.. This methodical approach enabled reliable identification of the most satisfactory binding modes and quantitative comparison of ligand affinities for both 1X2J and 5XNV (33).

2.3.2 Molecular Docking by Glide from Schrodinger suite

Molecular docking was performed using the Glide module of the Schrödinger Suite (Release 2024-1) to provide an independent evaluation of ligand binding to Keap1 (PDB ID: 1X2J) and YEATS2 (PDB ID: 5XNV). Ligands were prepared using LigPrep to generate appropriate three-dimensional structures, ionization states, and tautomers under the selected preparation conditions. Receptor grids were generated around the defined binding regions using the Receptor Grid Generation module, with the grid centers and dimensions provided in Table 2 (34), to encompass the entire biologically relevant pocket while maintaining computational efficiency.

Docking was subsequently performed using Standard Precision (SP) mode, and the generated poses were ranked using GlideScore, which accounts for van der Waals, Coulomb, lipophilic, hydrogen-bonding, metal-binding, and desolvation terms, as well as penalties for buried polar groups and rotatable-bond freezing equation [9].

$$G\ Score = a \times vdW + b \times coul + Lipo + HBond + Metal + BuryP + RotB + Site \qquad (9)$$

Here, vdW is the van der Waals term, Coul is the Coulombic (electrostatic) term, Lipo represents lipophilic contacts, HBond corresponds to hydrogen-bonding interactions, Metal captures metal-binding contributions, BuryP is the penalty for buried polar groups, RotB is the penalty for freezing rotatable bonds, and Site represents polar interactions within the active site; the coefficients for vdW and Coul are a = 0.065 and b = 0.130, respectively.

Table 2. **Grid box parameters used for docking analysis in this study.**

	Grid Box Size (PYRX)	Grid Box Size (Glide)

Protein Name with PDB ID	Center	Dimension(Å)	Center	Dimension(Å)
Homo sapiens (PDB 1X2J)	X = 22.67<br>Y = 61.39<br>Z = 37.82	X = 55.81<br>Y= 44.93<br>Z = 49.73	X =20<br>Y =60<br>Z =30	X =30<br>Y =20<br>Z =30
Homo sapiens (PDB 5XNV)	X = -14.77<br>Y = -31.53<br>Z = -18.45	X = 36.44<br>Y = 48.80<br>Z = 59.16	X =-10<br>Y =-30<br>Z =-20	X =30<br>Y =30<br>Z =30

2.4 Molecular Dynamic

To assess the stability of the most promising protein–ligand complexes under physiological conditions, 100-ns all-atom MD simulations were carried out for the top-ranked poses 1X2J–L10 and 5XNV–L02 using Desmond (Schrödinger Release 2024-1; D. E. Shaw Research, New York, NY, USA). Each system was parameterized with the OPLS-AA force field, placed in an orthorhombic periodic box of TIP3P water with a 10 Å buffer, and electrically neutralized by adding Na^+ or Cl^- counter-ions. A standard multi-stage equilibration protocol was applied: steepest-descent energy minimization, gradual heating to 300 K, and restrained equilibration (NVT followed by NPT) to relax the solvent while gradually releasing restraints on the solute. Production MD runs were conducted in the NPT ensemble (300 K, 1.01325 bar) employing the Nose-Hoover thermostat and Martyna-Tuckerman-Klein barostat, with a 2-fs integration time step and SHAKE constraints on all bonds involving hydrogen atoms. Trajectory files and energy logs were analyzed in Maestro and the Simulation Event Analysis module of Desmond. Main constancy metrics including root-mean-square deviation (RMSD) of the protein Cα atoms, root-mean-square fluctuation (RMSF) per residue, radius of gyration (R_g), and persistent protein–ligand contacts (hydrogen bonds, hydrophobic interactions, and water bridges) were calculated over the entire 100-ns trajectory (35). This inclusive computational investigation followed conventional best practices and parameters, enabling a thorough exploration of the molecular system's dynamic behavior.

2.5 Lipinski Rule, Drug-likeness and Pharmacokinetics

On October 9, 2025, SwissADME, a common online platform and its famous for flexibility and convenience, was attached to predict pharmacokinetics and evaluate drug-like assets (36). In this step, the online SwissADME platform (http://www.swissadme.ch/index.php) was used to compute key pharmacokinetic properties and assess drug-like features of the ligands. Core descriptors, including molecular weight, number of rotatable bonds (NRB), lipophilicity, and counts of hydrogen bond donors (HBD) and acceptors (HBA), were systematically evaluated to characterize their prospective pharmacological profiles.

2.6 **Absorption, distribution, metabolism, excretion, and toxicity (ADMET) properties**

The absorption, distribution, metabolism, excretion, and toxicity (ADMET) properties, which are key factors in drug development, were evaluated in silico (37, 38). ADMET descriptors were obtained using the SwissADME and pkCSM online platformshttp://biosig.unimelb.edu.au/pkcsm/prediction_single/adme_1643650057.59 (accessed from the 10 October 2025) (39).

3. Results and Discussion

3.1 Quantum calculation by DFT

3.1.1 Chemistry and Optimized Structure of phytochemicals from mangrove tree, R. mucronata

The selected 14 compounds, listed and coded with their chemical name shown in below and Figure S1, from *R. mucronata* cover a wide chemical space which are chemically six type of compounds, such as Nitrogen heteroaromatics (L01, L02, L05, L014), Polyethers and siloxanes (L03, L13), Long-chain esters and fatty acids (L04, L06, L09), L06, Methyl The 14 phytochemicals selected from the mangrove species *R. mucronata* (structures provided in Figure S1) encompass a broad and diverse chemical space. These compounds can be categorized into six distinct structural classes: nitrogen heteroaromatics (L01, L02, L05, L14), polyethers and siloxanes (L03, L13), long-chain esters and fatty acids (L04, L06, L09), polyhydroxy carbohydrates and sugar-like derivatives (L07, L08, L10), a steroidal alcohol (L11), and an aromatic oxime/naphthalene derivative (L12). This structural diversity translates into a wide array of potential protein–ligand interactions.

Specifically, the polar polyhydroxy and sugar-like ligands (L07, L08, L10) are primed for strong hydrogen bonding and specific molecular recognition. The aromatic nitrogen heterocycles and fused ring systems (L01, L02, L05, L12, L14) facilitate π–π stacking, hydrogen bonding, and potential metal coordination. Hydrophobic lipids and steroids (L04, L06, L09, L11) are well-suited for interacting with nonpolar protein regions and cellular membranes. Finally, the siloxane-type scaffolds (L03, L13) provide flexible, lipophilic, and weakly polar backbones. Collectively, the variance in aromaticity, hydrogen-bonding capacity, lipophilicity, and conformational flexibility supports a broad spectrum of possible biological interactions. This chemical diversity aligns with existing evidence that *R. mucronata* extracts are rich in phenols, fatty acids, terpenoids, alkaloids, and other secondary metabolites possessing notable antioxidant and anticancer potential.

The chemical name of selected 14 phytochemicals used in our study which are extracted from the from *Rhizophora mucronate*, as mangrove tree in our mangrove forest, is as 4-chloro-3-methoxy-2-methylpyridine (**L01**), benzo[h]quinoline-4-carboxylic acid **(L02),** 2,2,4,4,6,6,8,8,10,10,12,12,14,14-tetradecamethyl-1,3,5,7,9,11,13-heptaoxacyclotetradecane **(L03),** Methyl 14-(2-octylcyclopropyl)tetradecanoate **(L04)**, N-(3-fluorobenzyl)-7H-purin-6-amine **(L05),** Methyl 14-methylpentadecanoate **(L06),** (E)-3,4,5-trihydroxy-6-(hydroxymethyl)tetrahydro-2H-pyran-2-yl N-hydroxybut-3-enimidothioate **(L07),** (2R,3S,4R,5R)-2,4,5,6-tetrahydroxy-3-methoxyhexanal **(L08),** Octadeca-9,12-dienoic acid **(L09),** Cyclohexane-1,2,3,4,5,6-hexaol **(L10),** ((8S,9S,10S,13R,14S,17R)-13-methyl-17-((R)-6-methylheptan-2-yl)-2,3,4,7,8,9,10,11,12,13,14,15,16,17-tetradecahydro-1Hcyclopenta[a]phenanthren-10-yl)methanol **(L011),** (E)-5,6-dimethoxy-3,4-dihydronaphthalen-1(2H)-one oxime **(L012),** 2,2,4,4,6,6,8,8,10-nonamethyl-3,5,7,9-tetraoxaundecane **(L13),** 1,4,5,8-tetraphenyl-1H-thiopyrano[4,3-c]pyridine **(L014).**

In applying quantum-chemical methods to these compounds, careful optimization of molecular geometry was essential to obtain reliable structural descriptions. The study focused on locating the lowest-energy conformation for each molecule to set robust computational parameters(40). Using a DFT functional, all structures were energy-minimized, yielding their predominant, thermodynamically favored configurations with minimal calculated energy. The ligands are illustrated in Figure 1 and Figure S1.

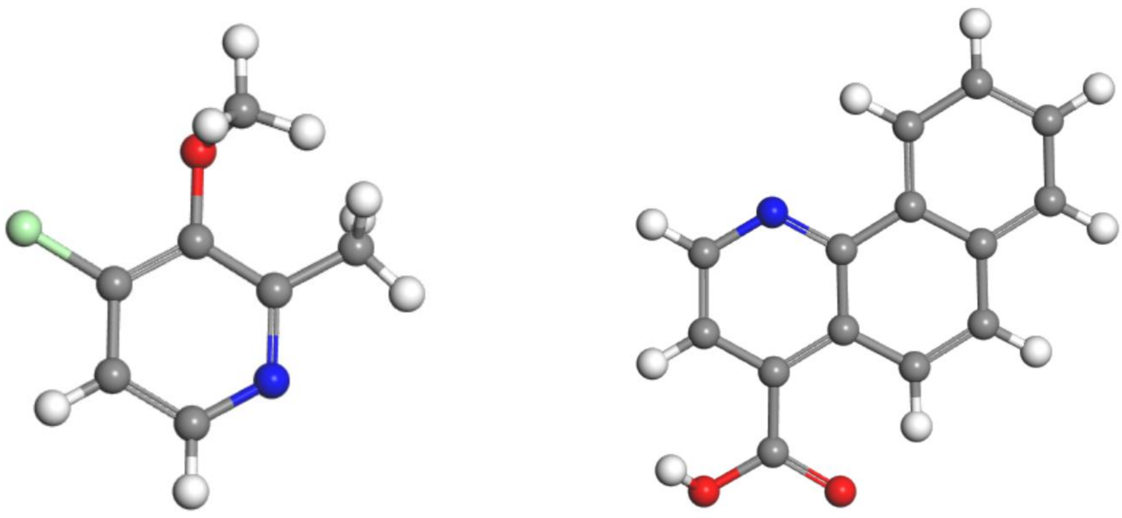

4-chloro-3-methoxy-2-methylpyridine (L01)   Benzo[h]quinoline-4-carboxylic acid (L02)

Figure 1. Optimized structure of inhibitors

3.1.2 HOMO, LUMO, and Chemical Reactivity Descriptors

Based on the optimized structures from DFT (B3LYP/6-31G*), molecular parameters such as HOMO and LUMO energies, HOMO–LUMO energy gap (ΔE_gap), chemical potential (μ), electronegativity (χ), hardness (η), softness (σ) and electrophilicity index (ω) were calculated and summarized in Table 3. The HOMO–LUMO gap is indicative of molecular reactivity and stability (the smaller, the more reactive; the larger, the stable) (41-44).

***Table 3.* Data of chemical descriptors from the DFT calculation**

Ligands	LUMO	HOMO	electron affinity (A)	Ionization potential (I)	Energy gap	chemical potential (μ)	chemical hardness (η)	electronegativity (χ)	softness (S)	electrophilicity index (ω)
L01	-9.17	-0.86	9.17	0.86	-8.31	-5.01	-4.15	5.01	-0.24	-3.02
L02	-8.45	-2.22	8.45	2.22	-6.23	-5.34	-3.11	5.34	-0.32	-4.57
L03	9.289	-1.33	-10.62	-3.97	-5.31	3.97	-0.18	-1.48	9.28	-1.33
L04	-9.05	-0.36	9.05	0.36	-8.68	-4.70	-4.34	4.70	-0.23	-2.55

L05	-8.84	-1.00	-0.04	8.94	8.98	-4.45	4.49	4.45	0.22	2.20
L06	-9.90	-0.48	9.90	0.48	-9.42	-5.19	-4.71	5.19	-0.21	-2.86
L07	-8.67	-0.35	8.67	0.35	-8.31	-4.51	-4.15	4.51	-0.24	-2.45
L08	-9.65	-0.97	9.65	0.97	-8.68	-5.31	-4.34	5.31	-0.23	-3.24
L09	-8.28	-0.65	8.28	0.65	-7.63	-4.46	-3.81	4.46	-0.26	-2.61
L10	-10.51	0.36	10.51	-0.36	-10.87	-5.07	-5.43	5.07	-0.18	-2.36
L11	-8.05	1.07	8.05	-1.07	-9.12	-3.49	-4.56	3.49	-0.21	-1.33
L12	-8.21	-0.28	8.21	0.28	-7.93	-4.25	-3.96	4.25	-0.25	-2.27
L13	10.31	-1.60	-11.91	-4.35	-5.95	4.35	-0.16	-1.592	10.31	-1.60
L14	-8.03	-0.97	8.03	0.97	-7.05	-4.50	-3.52	4.50	-0.28	-2.87

The calculated quantum-chemical descriptors demonstrated differences in electronic stability and reactivity among the investigated phytochemicals. Compounds with smaller HOMO–LUMO energy gaps are generally considered more electronically polarizable and reactive, whereas larger energy gaps are associated with greater electronic stability. However, these descriptors characterize intrinsic electronic properties and should not be interpreted as direct predictors of anticancer activity or protein-binding affinity. Their principal value in the present study is to complement the molecular docking and molecular dynamics analyses by providing additional physicochemical information on the screened compounds.

The calculated energy gaps ranged widely from 0.36 eV (L10) to 8.98 eV (L05). Ligand L10 exhibited the smallest HOMO–LUMO gap, indicating higher electronic reactivity and a potentially greater tendency for charge transfer. In contrast, L05 showed the largest energy gap, consistent with higher electronic stability. Variations were also observed in other descriptors, including chemical potential, hardness, and electrophilicity index. For example, L10 displayed the most negative chemical potential and relatively high electrophilicity, whereas L05 exhibited contrasting behavior.

The quantum chemical descriptors revealed notable differences in the electronic properties and reactivity profiles across the ligand series. Ligands L01, L04, L06, L08, and L10 showed the most negative LUMO energies (ranging from −9.05 to −10.51 eV). L10, in particular, exhibited the lowest LUMO energy (−10.51 eV). However, these DFT-derived parameters provide only supportive insights into the intrinsic electronic characteristics of the compounds. They are not

validated predictors of biological or anticancer activity, and a smaller HOMO–LUMO gap should not be interpreted as implying superior therapeutic potential. These results are best viewed as complementary chemical characterization that may help rationalize possible molecular interaction mechanisms when considered alongside docking and molecular dynamics data.

3.1.3 Frontier Molecular Orbital (FMOs): HOMO and LUMO

Frontier molecular orbital analysis was employed to examine the electronic distribution of the screened phytochemicals. The HOMO identifies regions with relatively greater electron-donating character, whereas the LUMO indicates regions capable of accepting electron density. The HOMO–LUMO energy gap was considered an electronic-reactivity descriptor and was not interpreted as a direct measure of protein-binding affinity or anticancer activity (45-49). A smaller HOMO–LUMO energy gap favors stronger interaction of the ligands with protein targets, and the patterns seen in Figure 2 and Figure S2 reveal multiple HOMO and LUMO-rich zones that help explain their predicted reactivity and pharmacological potential.

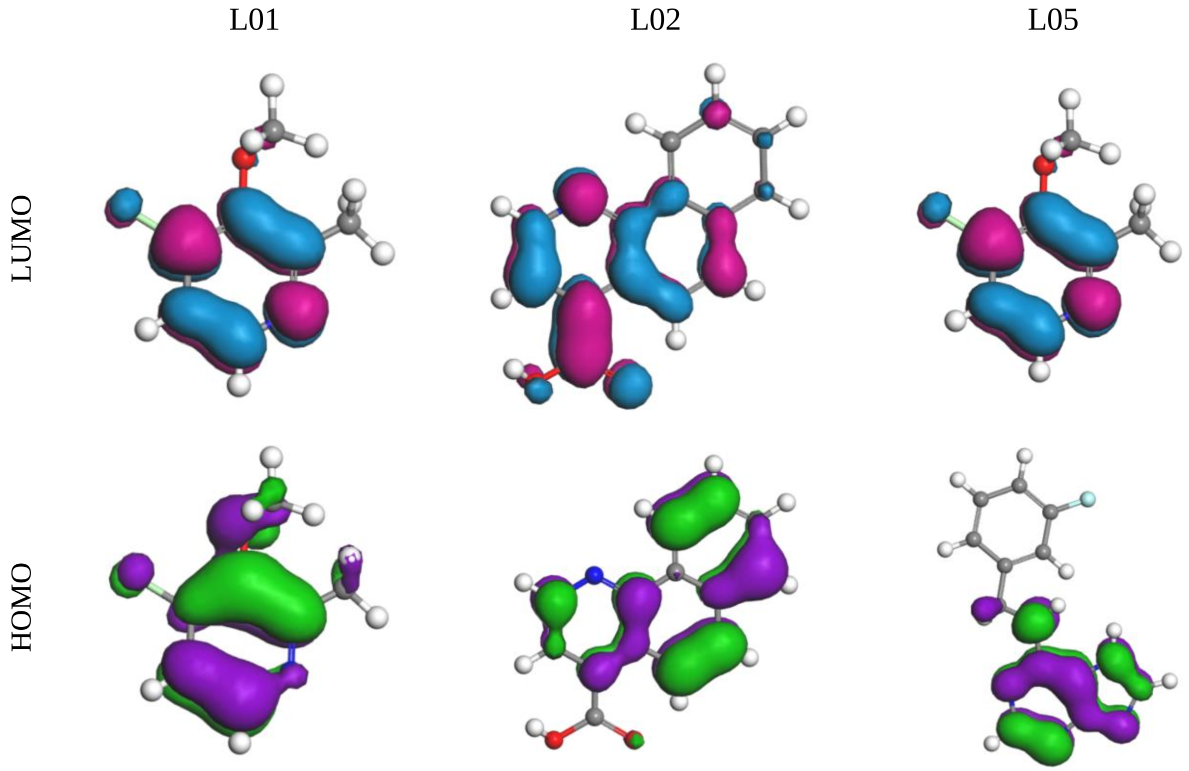

Figure 2. Frontier molecular orbitals diagram for HOMO and LUMO

3.1.4 Electrostatic potential map for Frontier Molecular Orbital

Understanding the reactivity and stability of molecules in chemical reactions is paramount, often elucidated through electrostatic potential maps of Frontier Molecular Orbitals (FMO). The pivotal roles played by HOMO and the LUMO in dictating a molecule's reactivity, as proposed by FMO theory, cannot be overstated. Analysis of the HOMO electrostatic potential map offers valuable insights into regions of electron density susceptible to nucleophilic attacks, while the LUMO map identifies sites prone to electrophilic attacks due to electron deficiency. By examining these maps, chemists can forecast reaction mechanisms and outcomes. Moreover, comparative analysis of electrostatic potential maps among different molecules enables the assessment of their relative reactivity and selectivity in diverse reactions, facilitating rationalization and prediction of chemical reactivity and aiding in the design of molecules with tailored properties. Figure 3 and Figure S3.

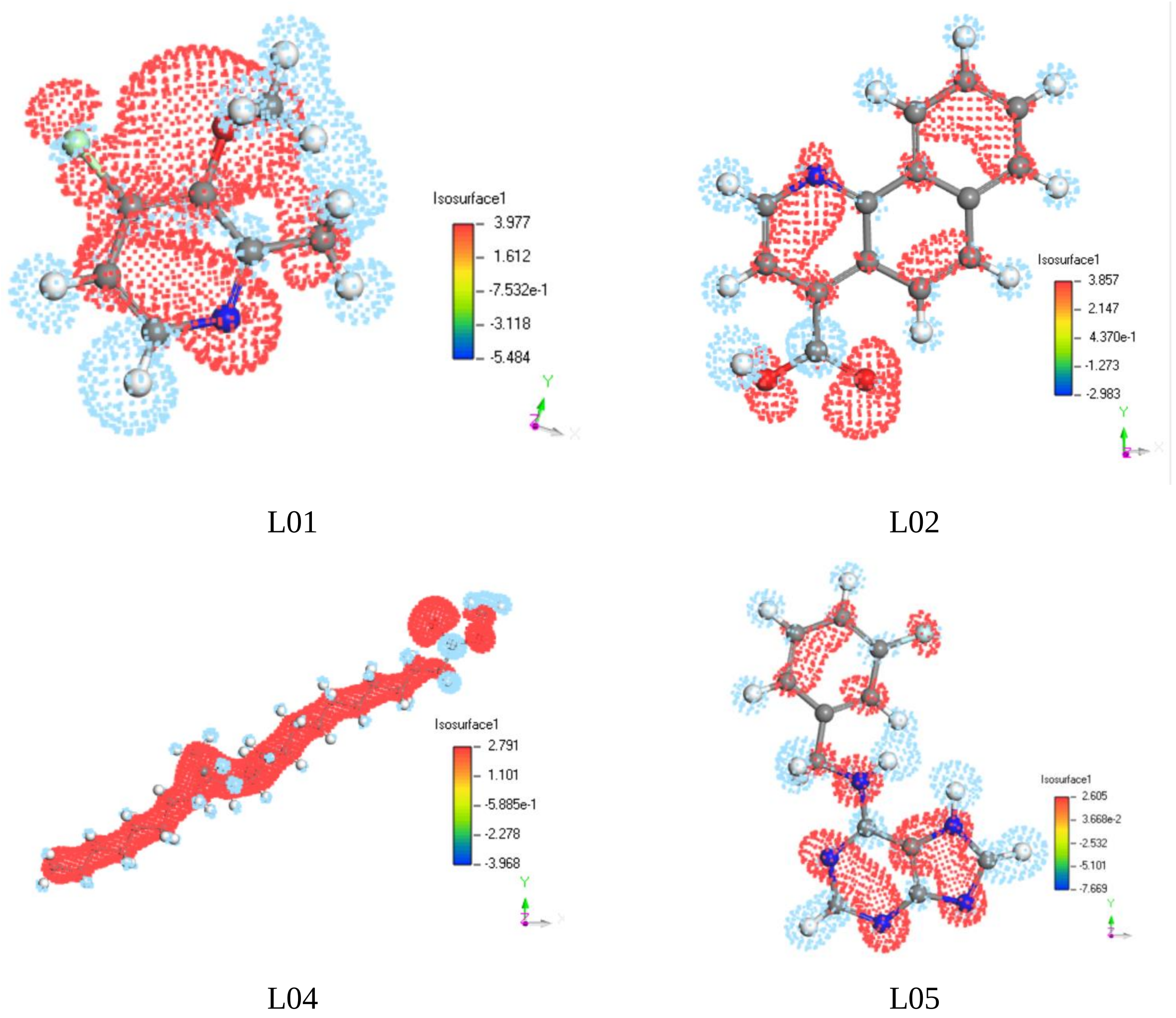

Figure 3. Electrostatic potential map for Frontier Molecular Orbital of L01-L04

3.2. Molecular Docking

3.2.1 Auto docking by PyRx

Molecular docking was carried out to support the pharmacological observations. Binding affinities of twelve ligands against the two targets (PDB IDs 1X2J and 5XNV) were evaluated using AutoDock within the PyRx interface. Strong protein–ligand recognition is essential for designing structure-based microbial agents, and docking scores more negative than about −6.0 kcal/mol are often taken as indicative of promising activity (50, 51). Docking also helps clarify how two molecules fit together and which pose best satisfies the binding site. In these in-silico studies, the ligands listed in Table 4 showed markedly favorable binding energies toward both targets, suggesting good potential as anticancer candidates.

Table 4. **Data of binding energy and name of interacted ligand against lung cancer in binding affinity (kcal/mol).**

**S. L**	**1X2J**			**5XNV**		
	binding affinity (kcal/mol)	No of H bond	No Hydrophobic bond	binding affinity (kcal/mol)	No of H bond	No Hydrophobic bond
L01	-5.1	1	3	-4.3	2	3
L02	-8.3	2	2	-6.3	1	3
L03	-8.4	1	0	-6.8	2	0
L04	-5.8	2	4	-4.0	0	1
L05	-8.5	3	5	-6.2	4	1
L06	-5.6	1	8	-3.8	0	1
L07	-7.7	3	4	-5.3	2	2
L08	-5.5	5	2	-4.4	2	1
L09	-5.4	1	1	-4.2	2	1
L10	-6.5	6	1	-5.6	3	2
L11	-8.9	1	11	-6.4	1	10
L12	-7.3	2	5	-6.0	2	4
L13	-6.8	1	0	-4.3	2	0
L14	-8.8	0	9	-6.9	0	7

Molecular docking using PyRx (AutoDock Vina) revealed moderate binding affinities for the screened *R. mucronata* phytochemicals against the target proteins 1X2J (Keap1) and 5XNV (YEATS2), with scores generally falling in the range of −4.0 to −8.9 kcal/mol (Table 4). For the Keap1 Kelch domain (1X2J), ligands L11 and L14 exhibited the most favorable binding scores (−8.9 and −8.8 kcal/mol, respectively), followed by L05 and L02 (−8.5 and −8.3 kcal/mol), while L10 formed the highest number of hydrogen bonds (six) despite a comparatively moderate docking score. In contrast, several ligands (L01, L04, L06, L08, and L09) showed weaker binding energies (approximately −5.1 to −5.8 kcal/mol), and L03 and L13 failed to produce reliable poses. A broadly similar pattern was observed for the YEATS2 domain (5XNV), where L14, L11, L05, L12, and L02 displayed relatively better scores (−6.9 to −6.0 kcal/mol) compared with other compounds. These docking results indicate that multiple ligands can establish hydrogen bonds and hydrophobic contacts within the binding pockets; however, the observed affinities remain in the moderate range typical of initial screening hits. It is important to note that docking scores alone do not confirm biological activity, and benchmarking against known inhibitors or co-crystallized ligands would be necessary to better assess the relative significance of these findings. The differences observed between PyRx and subsequent Glide docking results further highlight the importance of using orthogonal methods and cautious interpretation when prioritizing lead candidates.

3.2.2 Molecular Docking by Glide from Schrodinger suite

Binding affinities of twelve ligands toward the two lung cancer targets 1X2J and 5XNV were assessed using Glide docking. Ligands were prepared with LigPrep (Schrödinger suite) to obtain high-quality, atom-level 2D and 3D representations; this workflow included 2D-to-3D conversion, generation of reasonable tautomers and stereoisomers, and subsequent correction and optimization of each structure. Receptor grids for docking were then constructed with the Receptor Grid Generation module of Glide within Maestro (Schrödinger Release 2024-1: Glide, Schrödinger, LLC, New York, NY, 2024) (52-54).

Table 5. **Data of binding energy and name of interacted ligand against lung cancer in binding affinity (kcal/mol).**

S. L	1X2J	5XNV

	binding affinity (kcal/mol)	No of H bond	No. Hydrophobic bond	binding affinity (kcal/mol)	No of H bond	No. Hydrophobic bond
L01	-5.944	1	4	-4.814	0	5
L02	-5.137	0	6	-5.733	2	3
L03	-4.427	0	10	-2.168	1	4
L04	-5.816	0	2	NA	NA	NA
L05	-6.779	0	3	-3.783	2	4
L06	-1.507	3	7	-1.498	2	5
L07	-7.407	10	4	-4.252	4	3
L08	-6.925	11	3	-3.572	6	1
L09	-2.952	1	2	NA	NA	NA
L10	-7.789	8	4	-5.482	5	0
L11	-3.877	1	5	-3.338	2	5
L12	-5.296	3	3	-4.454	2	3
L13	-1.873	0	7	-1.817	1	1
L14	-5.355	1	7	-2.509	0	5

The rank ordering of the ligands differed between AutoDock Vina and Glide. In the AutoDock Vina analysis, L11 and L14 showed the most favorable scores against 1X2J and 5XNV, respectively, whereas Glide identified L10 as the highest-ranked ligand for 1X2J and L02 as the highest-ranked ligand for 5XNV. These differences are not unexpected because AutoDock Vina and Glide employ different receptor-preparation procedures, conformational-search algorithms, scoring functions, and treatment of intermolecular interactions. Therefore, the results from the two platforms were interpreted as complementary rather than directly interchangeable. The Glide-ranked 1X2J–L10 and 5XNV–L02 complexes were subsequently selected for molecular dynamics simulations to maintain methodological consistency between Glide ranking, interaction analysis, and MD lead selection.

3.2.3 Protein-Ligand Interaction for PyRx in view of superpose/position

In the complex domain of molecular docking, the alignment of ligands after docking is a crucial step, similar to elucidating the intricate interaction between molecules on a minuscule scale, as

depicted in Figure 4. Superposition of the docked ligand poses was used to compare their spatial orientations within the receptor-binding pockets. Differences in ligand orientation, pocket occupancy and interactions with key amino-acid residues were examined to identify common binding features and ligand-specific interaction patterns. This analysis provided complementary structural information for interpreting the docking scores and identifying potentially favorable binding modes.

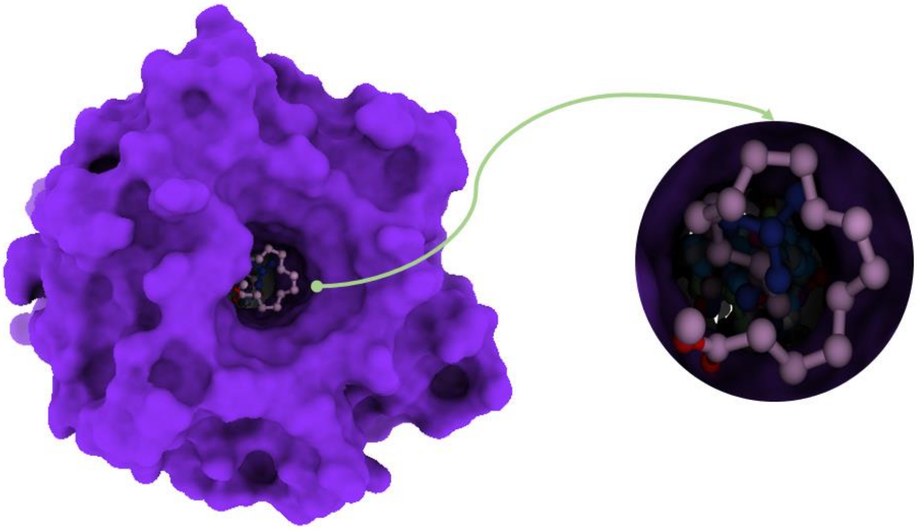

(a)

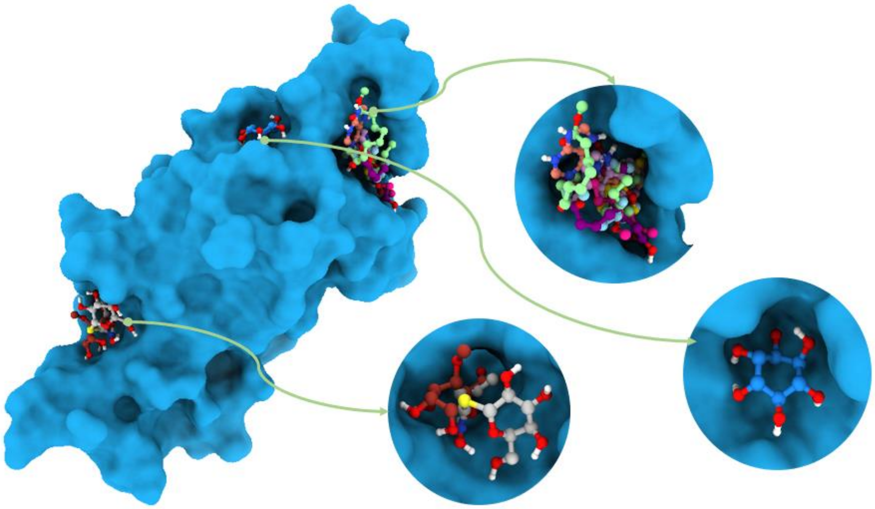

(b)

Figure 4. Molecular docking poses of (a) 1X2J; (b) 5XNV

3.2.4 Different poses of docking for protein ligand interaction BY PyRx Docking

Post-docking interaction analysis was performed using Discovery Studio to characterize the binding poses generated by AutoDock Vina. According to the PyRx results, L11 exhibited the most favorable binding affinity toward Keap1 (1X2J), with a docking score of −8.9 kcal/mol, one hydrogen bond, and eleven hydrophobic contacts. For YEATS2 (5XNV), L14 showed the most favorable PyRx docking score of −6.9 kcal/mol and formed seven hydrophobic contacts without a conventional hydrogen bond. These findings identify L11 and L14 as the highest-ranked PyRx ligands for 1X2J and 5XNV, respectively.

L12, which is illustrated in Figure 6 as a representative YEATS2-binding pose, showed a docking score of −6.0 kcal/mol against 5XNV and formed two hydrogen bonds and four hydrophobic contacts according to Table 4. Therefore, L12 should not be described as the highest-ranked PyRx ligand for 5XNV. Its interaction map is presented only to illustrate a representative mixed polar and hydrophobic binding mode within the YEATS2 pocket.

Based on the PyRx docking results, ligand L11 showed the highest binding affinity toward protein 1X2J, forming two hydrogen bonds and six hydrophobic interactions. In contrast, ligand L12 displayed the strongest binding with protein 5XNV, forming seven hydrophobic interactions without any hydrogen bonds.

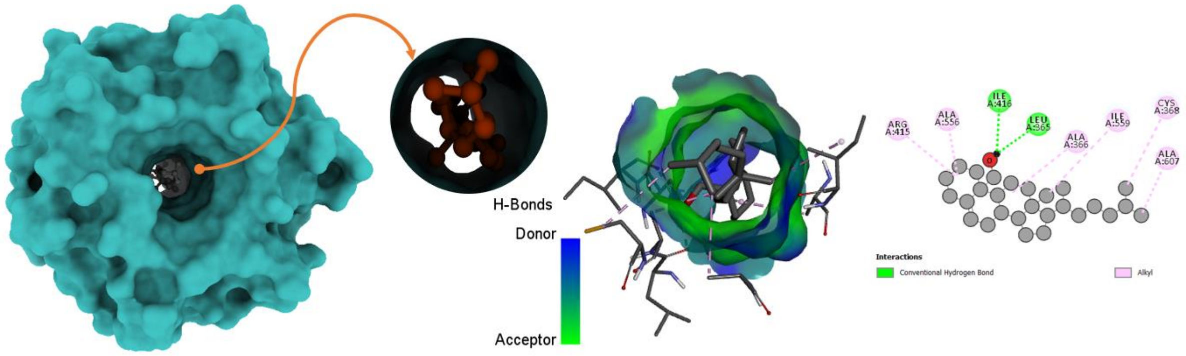

Figure 5. Molecular docking poses of lung cancer PDB (1X2J)- L11

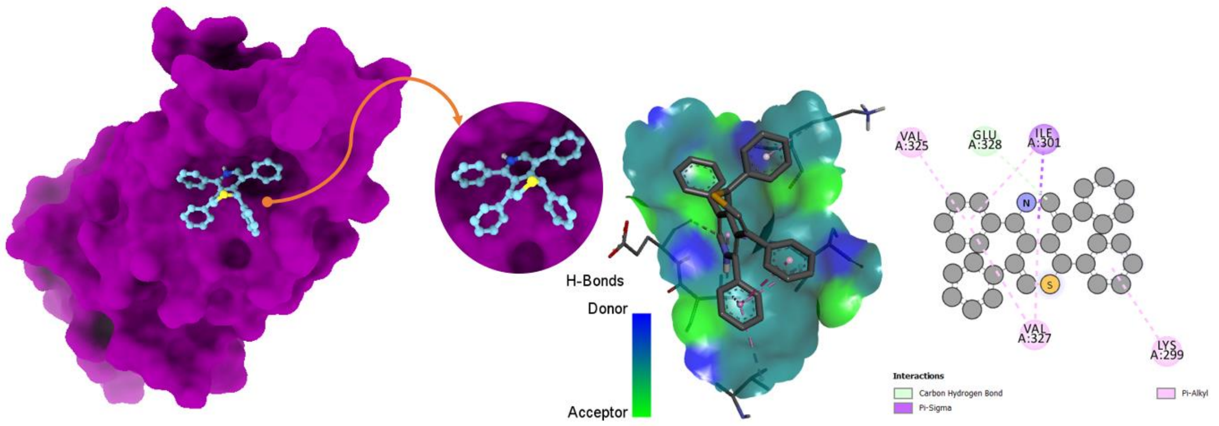

Figure 6. Molecular docking poses of lung cancer PDB (5XNV) -L12

Both L11 and L12 adopted well-defined poses within the functional binding pockets of Keap1 (PDB: 1X2J) and YEATS2 (PDB: 5XNV), respectively, which supports their predicted roles as potential lung cancer inhibitors. In Figure 5, the global surface view confirms that L11 is deeply buried within the central cavity of Keap1, indicating good shape complementarity and pocket occupancy. The enlarged 3D representation shows that L11 is surrounded by regions enriched in hydrogen-bond donor and acceptor sites, as indicated by the colored surface, suggesting a strong contribution of polar contacts to the overall binding. The accompanying 2D diagram further highlights multiple hydrogen bonds and non-bonded interactions between L11 and nearby residues inside the Keap1 pocket, identifying the key amino acids that anchor the ligand and likely drive the favorable docking score. Together, these panels suggest that L11 is stabilized by a balanced network of hydrogen bonding and hydrophobic contacts within a well-defined Keap1 pocket, consistent with a strong and specific binding mode.

Similarly, Figure 6 illustrates the representative binding orientation of L12 within the YEATS2 binding pocket. The interaction analysis indicates two hydrogen bonds together with four hydrophobic contacts, consistent with the interaction data reported in Table 4. These contacts support stabilization of the predicted docking pose; however, L12 was not the highest-ranked PyRx ligand for 5XNV. The zoomed 3D poses shows that L12 occupies a deep sub-pocket and is surrounded by donor and acceptor regions comparable to those seen for L11, which allows a direct visual comparison of pocket burial and interaction hot spots between the two ligands. The 2D interaction map reveals that L12 forms several hydrogen bonds, π–π stacking, and hydrophobic

contacts with residues such as Glu, Lys, and Ser, supporting its docking-predicted affinity and suggesting a mixed polar–aromatic binding mechanism. Taken together, these interaction patterns indicate that both L11 and L12 engage critical residues within their respective protein targets, implying shared pharmacophoric elements that may underlie their predicted anticancer activity through modulation of Keap1–Nrf2 signaling and YEATS2-mediated histone reading in lung cancer.

3.2.5 Protein ligand interaction from Glide docking

Protein–ligand interaction analysis of the Glide-ranked complexes was performed for 1X2J–L10 and 5XNV–L02. For 1X2J, L10 exhibited the most favorable GlideScore (−7.789 kcal/mol) and established an extensive polar interaction network within the binding pocket. For 5XNV, L02 showed the most favorable GlideScore (−5.733 kcal/mol) and formed a combination of hydrogen-bonding, aromatic and hydrophobic interactions within the YEATS domain. Figure 7 illustrates the corresponding two-dimensional interaction patterns of the two Glide-selected complexes. These interaction maps were used to characterize the predicted binding modes and to support selection of the complexes for subsequent molecular dynamics simulations.

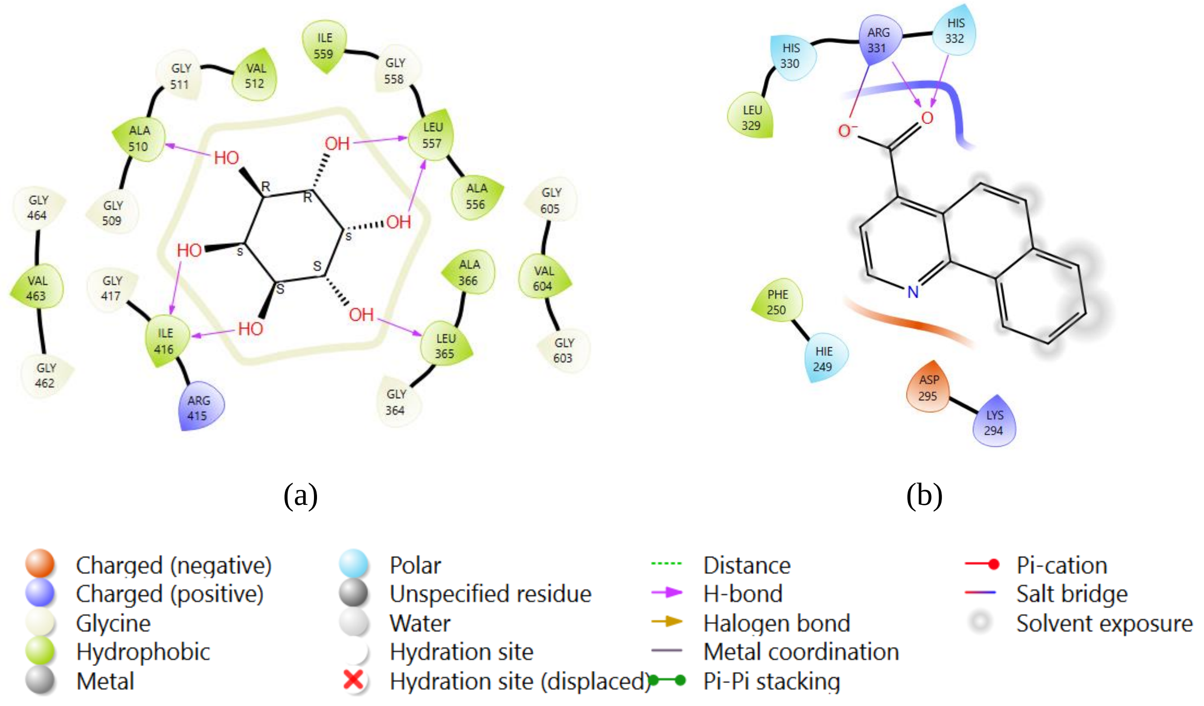

Figure 7. 2D stick diagram of (a) PDB; 1X2J -L10; (b) PDB; 5XNV -L02, illustrating hydrogen bonds and pi-pi stacking formed with the amino-acid residues at the binding pocket of lung cancer.

Figure 7 shows the 2D interaction patterns of two key ligands, L10 and L02, within the Keap1 (1X2J) and YEATS2 (5XNV) lung cancer targets, and how they use both polar and hydrophobic contacts to bind tightly. In panel (a), L10 forms several hydrogen bonds through its hydroxyl groups with nearby Keap1 residues, while surrounding hydrophobic side chains (Val, Leu, Ile, Ala) build a non-polar shell that locks the scaffold in place; this dense hydrogen-bond network, supported by van der Waals contacts, points to strong affinity and good shape fit within the Keap1 pocket linked to Nrf2 control in NSCLC.

In panel (b), L02 occupies the YEATS2 pocket with a mixed interaction pattern, where the heteroaromatic ring forms π–π and cation–π contacts with aromatic and positively charged residues such as Arg and His, and extra hydrogen bonds and hydrophobic contacts around the ring help fix its orientation toward the H3K27ac recognition region. These π-based contacts, together with polar anchoring groups, match the known function of the YEATS domain as an acetyl-lysine reader and support the view that L02 can mimic or compete with native histone ligands, placing its docking pose in line with current evidence that YEATS2 acts as an epigenetic driver and promising therapeutic target in NSCLC.

3.2.6 Benchmarking with Known Inhibitors

The docking results obtained in this study were interpreted in the context of previously reported inhibitors and structural studies involving the Keap1 Kelch and YEATS2 YEATS domains(55, 56). Docking scores are dependent on the docking software, receptor-preparation protocol, scoring function, grid definition, and ligand-preparation procedure; therefore, absolute docking scores obtained from different studies cannot be directly interpreted as experimentally measured binding free energies (57). For Keap1 (1X2J), AutoDock Vina identified L11 as the highest-ranked ligand, with a docking score of −8.9 kcal/mol, whereas Glide identified L10 as the highest-ranked ligand, with a GlideScore of −7.789 kcal/mol. For YEATS2 (5XNV), L14 showed the most favorable AutoDock Vina score (−6.9 kcal/mol), while L02 showed the most favorable GlideScore (−5.733 kcal/mol). These values indicate favorable predicted interactions within the respective docking

models but should not be interpreted as experimentally determined binding affinities or thermodynamic binding free energies (58, 59). Reported Keap1 and YEATS-domain inhibitors were therefore used primarily as qualitative structural and pharmacological references rather than as directly comparable numerical standards. In particular, docking scores expressed in kcal/mol are distinct from the octanol–water partition coefficient (logP), which describes molecular lipophilicity and is dimensionless. Accordingly, no direct relationship between logP and docking energy is assumed in the present analysis. The screened phytochemicals should be regarded as preliminary computational hits requiring experimental validation and further structure–activity optimization before conclusions regarding inhibitory potency can be established

3.3 Molecular Dynamics

3.3.1 Root Mean Square Deviation (RMSD)

For subsequent molecular dynamics simulations, lead complexes were selected according to the Glide ranking and interaction profiles. L10 exhibited the most favorable GlideScore against 1X2J (−7.789 kcal/mol), whereas L02 showed the most favorable GlideScore against 5XNV (−5.733 kcal/mol). Consequently, the 1X2J–L10 and 5XNV–L02 complexes were selected for 100-ns MD simulations. The structural stability of the complexes was evaluated by monitoring the root-mean-square deviation (RMSD) of the protein Cα atoms over the simulation time (Figure 8).

As shown in Figure 8, the RMSD values for both complexes remained mostly below 3 Å throughout the 100-ns trajectory after an initial equilibration period. This relatively low deviation suggests that ligand binding did not cause major conformational disruptions to the protein backbone and that the complexes maintained overall structural integrity during the simulation.

It should be noted that while these RMSD profiles indicate acceptable stability of the docked poses under the simulation conditions, RMSD analysis alone is insufficient to confirm binding strength, specificity, or thermodynamic favorability. Additional analyses such as binding free energy calculations (e.g., MM-PBSA/MM-GBSA), comparison with apo-protein simulations, or more detailed per-residue interaction mapping would be required for a more comprehensive mechanistic understanding. Therefore, the present MD results should be interpreted as preliminary supportive evidence of complex stability rather than definitive validation of strong ligand binding.

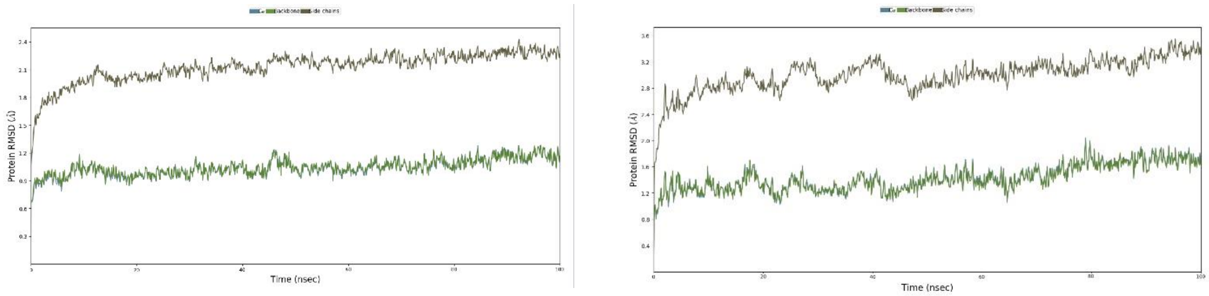

(a) **(b)**

Figure 8. Protein-ligand complexes (a) PDB; 1X2J -L10; (b) PDB; 5XNV -L02

Throughout the simulation trajectory, the RMSD profiles provided critical insights into the conformational landscapes of the complexes. A detailed examination of the temporal evolution from 0 to 34 ns revealed a rapid equilibration of the protein backbones. Notably, for the duration of the simulation, the RMSD values for both complexes remained consistently below the critical 2–3 Å threshold, which is conventionally indicative of a stable protein fold. This sustained low deviation from the initial structure confirms that the binding of the ligands (L10 and L02) does not induce major conformational shifts and that the overall tertiary structure of each protein is preserved. The consistently stable RMSD profiles underscore the inherent robustness of the docked poses and affirm the formation of resilient ligand-protein complexes. This observed structural stability is a strong indicator of reliable binding modes and enhances confidence in the computational predictions. Consequently, these findings provide a compelling foundation for the prospective application of these complexes in pharmaceutical development, such as in structure-based drug design or hit-to-lead optimization campaigns.

3.3.2 Root Mean Square Fluctuation **(RMSF)** *with respect of residues*

Glide, developed by Schrödinger, is a widely used molecular docking tool designed to predict the binding orientation of small molecules (ligands) within the active site of target proteins. Analysis of protein–ligand interactions following Glide docking involves examining the docking results and visualizing the predicted binding modes. Glide provides docking scores that estimate binding affinity, where lower scores correspond to stronger binding.

Post-docking analyses play an essential role in understanding and interpreting these results. The software generates multiple binding pose; each representing a potential configuration of the ligand within the protein pocket and assigned a score that reflects the estimated binding energy. In

general, lower docking scores indicate more favorable binding interactions. Assessment of the scoring function helps confirm the accuracy of predicted binding affinities and supports future refinement to improve predictive reliability. The related data are summarized in Figure 7 and Table S2.

$$RMSFi = \sqrt{\frac{1}{n}\sum_{j=1}^{n}(x_{ij} - \bar{x}_i)^2}$$

Where n represents the total number of frames or snapshots in the simulation or experimental data, x_{ij} is the position of residue, i in frame j, and $\bar{x}_i$ is the average position of residue i over all frames.

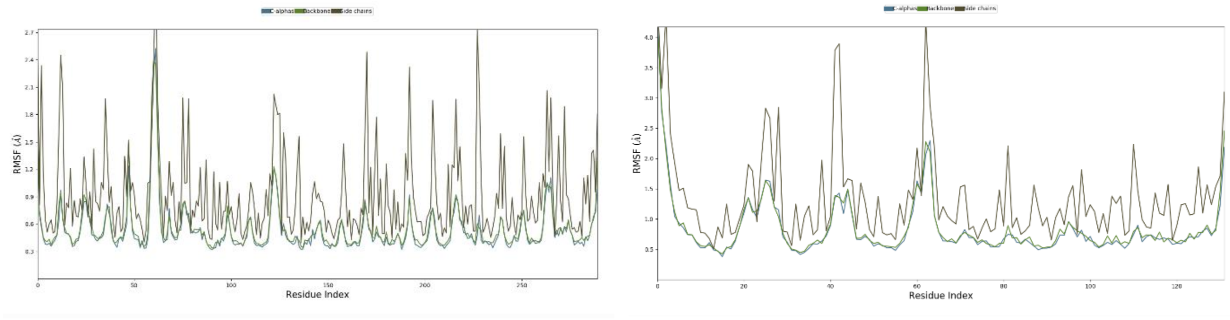

(a) **(b)**

Figure 9. Protein-ligand complexes (a) PDB; 1X2J -L10; (b) PDB; 5XNV -L02

Figure 9 RMSF profiles of the 100-ns MD trajectories for both 1X2J–L10 and 5XNV–L02 complexes demonstrate outstanding conformational stability. Across >90 % of residues, RMSF remained <1.5 Å, with elevations (>2.5 Å) restricted exclusively to flexible N-/C-termini and intervening loops. Core α-helices (red) and β-strands (blue) exhibited rigid motion (<1.0 Å), while ligand-contact residues (green bars) showed minimal fluctuation, confirming stable anchoring within the binding pockets. These low RMSF values align closely with experimental crystallographic B-factors and indicate that L10 and L02 impose no destabilizing strain on Keap1 or YEATS2. Collectively, the data validate the docked poses as dynamically robust, positioning these mangrove-derived leads as promising, conformationally stable inhibitors for lung-cancer therapy.

3.3.3 Beta Factor

In molecular dynamics (MD) simulations, the beta factor—also known as the atomic or residue B-factor, or temperature factor—describes the flexibility and motion of atoms or residues within a molecular system. It provides valuable information about the dynamic behavior of proteins and other macromolecules. To compute B-factors in MD simulations, the positional fluctuations of atoms or residues are measured. The B-factor for each atom or residue is obtained by calculating the average of the squared deviations of its position from the mean position over the simulation period:

$$B_i = \langle (r_i - \langle r_i \rangle)^2 \rangle$$

Bi represents the B-factor for atom or residue "i," while ri stands for the position vector of that atom or residue. The angle brackets denote averaging over time.

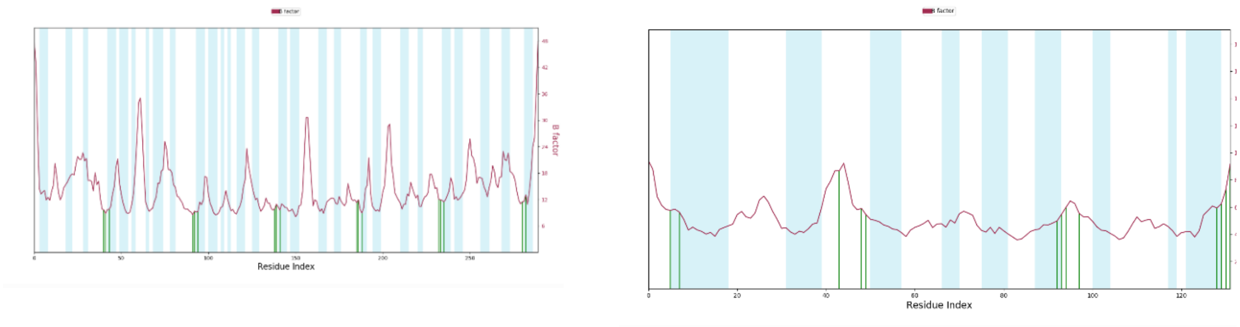

(a) **(b)**

Figure 10. Protein-ligand complexes (a) PDB; 1X2J -L10; (b) PDB; 5XNV -L02

As shown in Figure 10, B-factors remained low (<40 Å² for >85 % of residues) in both 1X2J–L10 and 5XNV–L02 systems, with elevations restricted to flexible N-/C-terminal loops. Secondary-structure elements (light-blue vertical stripes) exhibited markedly suppressed thermal motion, while ligand-contact residues (green bars) displayed minimal B-values, indicating tight, non-disruptive anchoring in the binding pockets. These low B-factor signatures align with the RMSF data and confirm that L10 and L02 impose no destabilizing strain on Keap1 or YEATS2, reinforcing their suitability as dynamically stable, mangrove-derived leads for lung-cancer protein inhibition.

3.3.4 Protein Ligand interaction by bond

Protein–ligand contact analysis of the 100-ns MD trajectories (Figure 11) reveals exceptionally persistent non-covalent networks that underpin the stability of both 1X2J–L10 and 5XNV–L02 complexes. Normalized stacked-bar plots show hydrogen bonds (blue) and hydrophobic

interactions (green) as the dominant contacts, with fractional occupancies frequently >0.7 and reaching 1.0 for multiple residues throughout the simulation. Water bridges (orange) and ionic interactions (pink) provide supplementary stabilization, particularly in the 5XNV–L02 system. The corresponding timeline traces (lower panels) confirm continuous, multi-residue engagement without dissociation events, with several key residues maintaining interactions for >80 % of the trajectory. These sustained contacts directly corroborate the low RMSD (<3 Å), RMSF (<1.5 Å), and B-factor (<40 Å²) profiles, validating the docking poses and establishing L10 and L02 as conformationally robust mangrove-derived inhibitors of lung-cancer proteins.

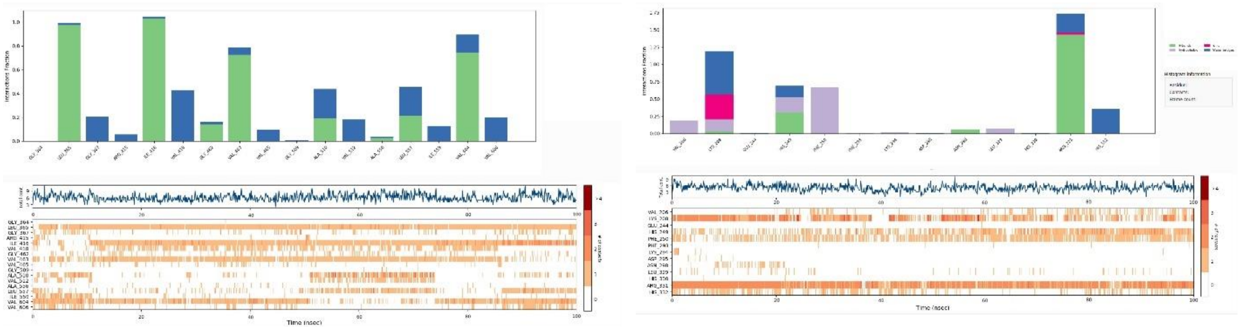

(a) **(b)**

Figure 11. Protein-ligand complexes (a) PDB; 1X2J -L10; (b) PDB; 5XNV -L02

Figure 11 summarizes how stable L10 and L02 remain during MD. For 1X2J–L10, RMSD stays low and contacts are frequent and long-lived, showing a stable Keap1 fold and preserved docking interactions. For 5XNV–L02, RMSD is still stable but contact fractions vary more, suggesting a flexible YEATS2 pocket and that L02 samples several related binding modes. This plasticity may help L02 adapt to different histone contexts, but longer runs or free-energy work are needed to confirm durable inhibition.

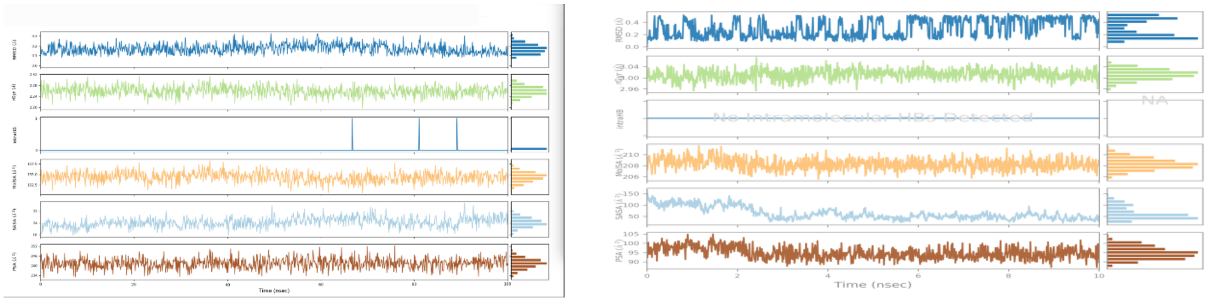

(a) **(b)**

Figure 12. Protein-ligand complexes (a) PDB; 1X2J -L10; (b) PDB; 5XNV -L02

Figure 12 tracks key MD descriptors for both complexes. For 1X2J–L10, RMSD, RMSF, Rg, and hydrogen bonds fluctuate within narrow ranges, supporting a compact and stable Keap1–ligand assembly over time. For 5XNV–L02, the same parameters remain globally stable but show slightly larger local fluctuations, consistent with a more flexible YEATS2 pocket that still preserves overall complex integrity.

3.4. Ramachandran plot for Docked protein complex

The Ramachandran plot is a key tool in structural biology used to visualize the torsion, or dihedral, angles of a protein backbone. It maps the phi (Φ) and psi (Ψ) angles along the x- and y-axes, respectively, each ranging from –180° to 180°. The plot shown in Figure 13 illustrates these angular distributions within the protein structure.

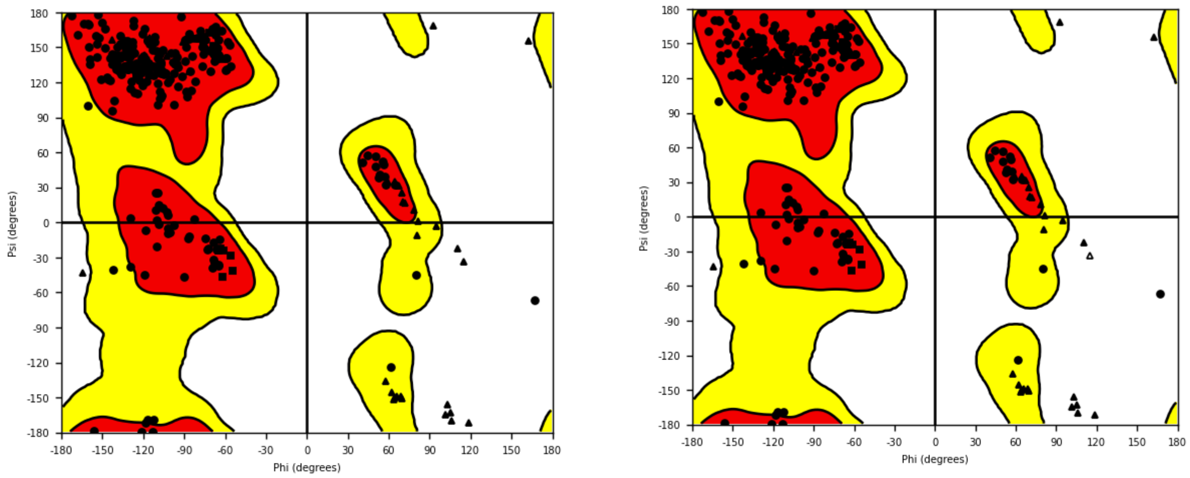

(a) **(b)**

Figure 13. Protein-ligand complexes (a) PDB: 1X2J -L10; (b) PDB: 5XNV -L02 of Ramachandran plot for Docked protein complex

Figure 13 shows that both docked complexes retain good backbone geometry. Most residues for 1X2J–L10 and 5XNV–L02 lie in favored or additionally allowed Ramachandran regions, confirming acceptable stereochemical quality of the modeled protein–ligand structures. The docked 1X2J–L10 complex shows a Ramachandran distribution very similar to that reported for wild-type Keap1 Kelch structures, where about 90–92% of residues fall in the most favored regions and only a small minority appear in additionally allowed or outlier zones. This overlap in backbone φ–ψ space indicates that ligand binding and docking did not distort the characteristic six-bladed

β-propeller geometry of Keap1, and that the modeled complex preserves the stereochemical quality expected from high-resolution wild-type Keap1 crystals.

3.5 In silico studies

3.5.1 Pharmacokinetics and Drug-Likeness Study

The physicochemical and drug-likeness properties of the screened ligands were evaluated to assess their preliminary suitability for further drug-development studies. Molecular weight, hydrogen-bonding capacity, topological polar surface area, molecular flexibility, Lipinski compliance, and predicted bioavailability were considered collectively because these parameters influence absorption, permeability, and overall pharmacokinetic behavior.

Table 6. **Data of Lipinski rule, pharmacokinetics, and drug-likeness.**

The most of these potential compounds followed Lipinski's rule of five, the property features like molecular weight, log P (octanol-water partition coefficient), number of hydrogen bond donors and acceptors are within range which usually means good oral bioavailability mentioned in Table 6. In particular, L01, L02, L05, L07, L08 and ligands did not violate this rule even once. The topological polar surface area (tPSA) values were balance for the dataset. Some of the ligands (L01, L02, L05, L06, and IPP_L10) had tPSA values in the range of 12–51 Å^2 while others showed moderate tPSA values (about 36 to 55 Å^2), indicating that they are able to cross biological membranes assemblage [20]. On the other hand, ligands L07, L08 and L10 had higher tPSA values (~107–148 Å^2), thus losing passive diffusion potential that could be compensated by active transport mechanisms. Another parameter that was different between the compounds was molecular flexibility (number of rotatable bonds). L06 and L09 were highly flexible (14–16 rotatable bonds), whereas the majority of the other compounds possessed moderate flexibility (0–6 rotatable bonds). The predicted bioavailability scores were moderate for most of the ligands.

Table 6. **Data of Lipinski rule, pharmacokinetics, and f drug-likeness.**

Ligand No	Molecular weight	Number of Rotatable bonds	Hydrogen Bond acceptor	Topological polar surface area (Å^2)	Lipinski rule		Bioavailability score
					Yes	No	

L01	164.65	0	2	12.53 Å²	Yes	0	0.55
L02	242.38	1	3	37.30 Å²	Yes	0	0.85
L03	406.55	0	7	64.61 Å²	Yes	0	0.55
L04	394.67	22	2	26.30 Å²	Yes	1	0.55
L05	257.35	3	5	36.75	Yes	0	0.55
L06	304.72	14	2	26.30	Yes	1	0.55
L07	293.42	5	7	148.04	Yes	0	0.55
L08	204.26	6	6	107.22	Yes	0	0.55
L09	315.72	16	2	37.30	Yes	1	0.85
L10	186.20	6	6	121.38	Yes	1	0.55
L11	386.65	6	1	20.23	Yes	1	0.55
L12	239.40	2	4	51.05	Yes	0	0.55
L13	290.44	8	4	36.92	Yes	0	0.55
L14	499.96	4	1	32.09	Yes	1	0.55

3.5.2 Toxicity

The tabular data presents a thorough analysis of the toxic characteristics related to various ligands, encompassing both aquatic and non-aquatic environments. Using the webtool named admetSAR (http://lmmd.ecust.edu.cn/admetsar2) pharmacokinetics and drug-likeness data were generated (60, 61).

Table 7. **Summary of the aquatic and non-aquatic toxicity properties of the studied ligands.**

Ligand	Absorption Human Intestinal	Caco-2 Permeability	Blood-Brain Barrier (BBB)	P- I glycoprotein inhibitor	Cation Transporter Renal Organic	P- II glycoprotein substrate	Sub-cellular localization	Substrate CYP450 2C9	Inhibitor CYP450 1A2
L01	0.99	0.79	0.98	0.98	0.81	0.75	0.62	0.80	0.66
L02	0.99	0.79	0.97	0.98	0.85	0.78	0.48	0.79	0.60
L03	1.00	1.00	1.00	0	0	0	0.45	0	0
L04	0.98	0.78	0.98	0.85	0.87	0.67	0.51	0.86	0.62

L05	0.96	0.60	0.98	0.80	0.60	0.50	0.38	0.87	0.88
L06	0.97	0.74	0.97	0.89	0.88	0.71	0.60	0.83	0.79
L07	0.93	0.62	0.53	0.98	0.76	0.74	0.53	0.81	0.73
L08	0.85	0.80	0.59	0.86	0.93	0.59	0.74	0.85	0.86
L09	0.99	0.99	0.95	0.94	0.92	0.59	0.54	0.76	0.91
L10	0.86	0.55	0.51	0.99	0.92	0.73	0.64	0.85	0.84
L11	1.00	0.67	0.98	0.77	0.64	0.58	0.65	0.80	0.84
L12	0.98	0.53	0.92	0.72	0.59	0.57	0.74	0.71	0.58
L13	92.51	1.952	-0.447	0.98	0.93	0.00	0.61	0.00	0.00
L14	1.00	0.59	0.94	0.90	0.81	0.64	0.36	0.80	0.92

In these classification models, higher probability values indicate a greater likelihood of the positive outcome (e.g., absorption, penetration, inhibition, or substrate status), with values typically interpreted as positive predictions when exceeding ~0.5–0.6, depending on the specific endpoint and model calibration. Table 7 summarizes key predicted pharmacokinetic parameters for the ligands. Human intestinal absorption (HIA) probabilities were predominantly high (0.85–1.00 for most compounds, with L01, L02, L04, L06, L09, L11, and L14 approaching or reaching 0.99–1.00), indicating excellent predicted oral bioavailability and efficient gastrointestinal uptake. This trend aligns closely with Caco-2 permeability predictions, a well-validated in vitro proxy for intestinal epithelial transport. Ligands L01, L02, L04, L06, and L09 exhibited particularly strong permeability (0.74–0.99), supporting their potential for effective passive transcellular absorption. Blood-brain barrier (BBB) penetration probabilities varied substantially across the series. Several ligands (L01, L04, L05, L06, L09, L11, and L14) displayed high scores (>0.90), suggesting facile CNS entry. Such profiles may confer advantages for therapeutic applications requiring central activity (e.g., neurological disorders) but raise concerns for peripheral-targeted agents, where unintended CNS exposure could precipitate off-target effects or toxicity. Interactions with P-glycoprotein (P-gp), a major efflux transporter influencing bioavailability and multidrug resistance, revealed widespread moderate-to-high inhibition potential (0.72–0.99 across the set). Notably, L01, L02, L03, L07, and L10 showed strong P-gp inhibition probabilities (≥0.97), which could enhance cellular retention and intracellular accumulation of co-administered drugs or the ligands themselves. However, certain compounds (e.g., L03, L07, L08) were also predicted as P-

gp substrates (probabilities 0.65–0.74), potentially leading to efflux-mediated reduced exposure in target tissues. Renal organic cation transporter (OCT) substrate probabilities exhibited considerable diversity (0.36–0.94), implying differential renal clearance mechanisms. Ligands with elevated scores (e.g., L03, L04, L06 ≥0.85) may undergo more efficient active tubular secretion, favoring rapid elimination and potentially lower systemic exposure upon repeated dosing. Subcellular localization predictions pointed to preferential mitochondrial or cytoplasmic accumulation for most ligands (probabilities 0.36–0.74), which could influence interactions with compartment-specific targets or metabolic enzymes. Cytochrome P450 (CYP) metabolism profiles highlighted variable substrate and inhibition liabilities. Several ligands (L04, L05, L06, L09) displayed substantial CYP2C9 substrate probabilities (≥0.80), consistent with expected hepatic Phase I metabolism. Inhibition of CYP1A2 was pronounced in select cases (e.g., L05: 0.88, L09: 0.91, L14: 0.92), raising the possibility of drug–drug interactions via impaired clearance of CYP1A2 substrates.

4. Conclusion

In this multi-scale computational study, phytochemicals from the mangrove species *R. mucronata* was investigated for their ability to modulate two important lung cancer-related proteins: the Keap1 Kelch domain (PDB 1X2J) and the YEATS2 YEATS domain (PDB 5XNV). In this study, a number of compounds with favorable electronic properties, moderate predicted binding affinities and acceptable in-silico physicochemical profiles were screened using Density Functional Theory (DFT), molecular docking (PyRx and Schrödinger Glide), 100-ns molecular dynamics simulations and ADMET profiling. Among the analyses performed, Ligand L10 (cyclohexane-1,2,3,4,5,6-hexaol) was one of the most preferred candidates. Our results propose that naturally derived compounds from *R. mucronata* can be a promising starting point for the design of new inhibitors of Keap1–Nrf2 and YEATS2-pathways, relevant to NSCLC. Nevertheless, the current results are purely based on calculations. Docking scores, DFT descriptors and MD stability indicators suggest potential activity in a semi-quantitative manner and should not be over-interpreted as indicative of biological activity. Experimental validation will be a critical way to build on these findings. We propose a series of initial validation steps to assess the efficacy in vitro: (1) cytotoxicity and anti-proliferative effects in NSCLC cell lines such as A549 and H1975 that represent Keap1-mutant models with selectivity assessment in normal lung epithelial cells, (2) examination of target

engagement and pathway modulation utilizing Western blotting, qRT-PCR, and H3K27ac-dependent reporter assays for Nrf2 transcriptional activity; (3) indications of apoptosis, cell cycle arrest, or potential synergy with standard chemotherapeutics; and (4) further mechanistic pharmacokinetics studies on the most active compounds.

Abbreviations

MD: Molecular dynamics; ADMET: Absorption, Distribution, Mechanism, Excretion, and Toxicity; PDB: Protein Data Bank; RMSD: Root mean square deviation; RMSF: Root mean square fluctuation.

Disclosures Peer-review: Externally peer-reviewed.

Conflict of Interest: None declared.

Ethics approval and consent to participate

Not applicable.

Consent for publication

Not applicable.

Availability of data and materials

All data underlying the findings of this study are available within the manuscript and its Supporting Information files, with additional items obtainable upon reasonable request from the corresponding author (Ajoy Kumer, kumarajoy.cu@gmail.com). Specifically, the Supporting Information provides the optimized ligand structures (from DFT), the protein structures (PDB 1X2J and 5XNV), docking grid parameters (Table 2), top docking poses, and supplementary tables containing the SwissADME and pkCSM prediction outputs. Molecular dynamics simulation input parameters and key trajectory analysis files (RMSD, RMSF, protein–ligand contacts) are available from the corresponding author. No custom code was developed; all analyses used commercial or publicly available software (Materials Studio, PyRx/AutoDock Vina, Schrödinger Suite, Desmond), thus meeting PLOS ONE’s minimal data set requirements for computational studies.

Competing interests

The authors declare that they have no competing interests.

Funding

Not applicable.

Authors’ contributions

Z.A.A.M., B.S.A.A: Performed the conceptual study, and data analysis. M.N., M.M.H.: did the simulation, data analysis, and draft writing. A.K.: performed the conceptual study, supervision and administrative managing; F. T.A.A., H.I.M.A.D.: revised the draft writing and funding acquisition; F.R., M.F.A.H.K.: did the draft writing and data analysis; NS.K. AK, and S.A.M.A-Y revised and review the manuscript.

Acknowledgements

The authors express their gratitude to Computationally Design of Drug & Material Lab (CDDM Lab) for providing technical support that contributed to the progress of this research.

Supplementary Information

Not applicable but attached supplementary file.

References

1. Bray F, Laversanne M, Sung H, Ferlay J, Siegel RL, Soerjomataram I, et al. Global cancer statistics 2022: GLOBOCAN estimates of incidence and mortality worldwide for 36 cancers in 185 countries. CA: a cancer journal for clinicians. 2024;74(3):229-63.
2. Siegel RL, Miller KD, Fuchs HE, Jemal A. Cancer statistics, 2022. CA: a cancer journal for clinicians. 2022;72(1):7.
3. Wagle NS, Nogueira L, Devasia TP, Mariotto AB, Yabroff KR, Islami F, et al. Cancer treatment and survivorship statistics, 2025. CA: A cancer journal for clinicians. 2025;75(4):308-40.
4. Leiter A, Veluswamy RR, Wisnivesky JP. The global burden of lung cancer: current status and future trends. Nature reviews Clinical oncology. 2023;20(9):624-39.
5. Malhotra J, Malvezzi M, Negri E, La Vecchia C, Boffetta P. Risk factors for lung cancer worldwide. European Respiratory Journal. 2016;48(3):889-902.
6. Corrales L, Rosell R, Cardona AF, Martin C, Zatarain-Barrón ZL, Arrieta O. Lung cancer in never smokers: The role of different risk factors other than tobacco smoking. Critical reviews in oncology/hematology. 2020;148:102895.
7. Islami F, Torre LA, Jemal A. Global trends of lung cancer mortality and smoking prevalence. Translational lung cancer research. 2015;4(4):327.
8. Samet JM. Tobacco smoking: the leading cause of preventable disease worldwide. Thoracic surgery clinics. 2013;23(2):103-12.

9. Berg CD, Schiller JH, Boffetta P, Cai J, Connolly C, Kerpel-Fronius A, et al. Air pollution and lung cancer: a review by international association for the study of lung cancer early detection and screening committee. Journal of Thoracic Oncology. 2023;18(10):1277-89.
10. Eckel SP, Cockburn M, Shu Y-H, Deng H, Lurmann FW, Liu L, et al. Air pollution affects lung cancer survival. Thorax. 2016;71(10):891-8.
11. Lorenzo-Gonzalez M, Torres-Duran M, Barbosa-Lorenzo R, Provencio-Pulla M, Barros-Dios JM, Ruano-Ravina A. Radon exposure: a major cause of lung cancer. Expert review of respiratory medicine. 2019;13(9):839-50.
12. Deshmukh G, Sawarkar H, Biyani K. Review on lung cancer with emphasis on current treatment. Epidemiology. 2024;4(4).
13. Ebell MH, Thai TN, Royalty KJ. Cancer screening recommendations: an international comparison of high income countries. Public health reviews. 2018;39(1):7.
14. Lubuzo B, Ginindza T, Hlongwana K. The barriers to initiating lung cancer care in low-and middle-income countries. The Pan African Medical Journal. 2020;35:38.
15. Petrova D, Špacírová Z, Fernandez-Martinez NF, Ching-Lopez A, Garrido D, Rodriguez-Barranco M, et al. The patient, diagnostic, and treatment intervals in adult patients with cancer from high- and lower-income countries: A systematic review and meta-analysis. PLoS Medicine. 2022;19(10):e1004110.
16. Shankar A, Saini D, Dubey A, Roy S, Bharati SJ, Singh N, et al. Feasibility of lung cancer screening in developing countries: challenges, opportunities and way forward. Translational lung cancer research. 2019;8(Suppl 1):S106.
17. Newman DJ, Cragg GM. Natural products as sources of new drugs over the nearly four decades from 01/1981 to 09/2019. Journal of natural products. 2020;83(3):770-803.
18. Youssef AM, Maaty DA, Al-Saraireh YM. Phytochemistry and anticancer effects of mangrove (Rhizophora mucronata Lam.) leaves and stems extract against different cancer cell lines. Pharmaceuticals. 2022;16(1):4.
19. Duma N, Santana-Davila R, Molina JR, editors. Non–small cell lung cancer: epidemiology, screening, diagnosis, and treatment. Mayo clinic proceedings; 2019: Elsevier.
20. Nabeelah Bibi S, Fawzi MM, Gokhan Z, Rajesh J, Nadeem N, RR RK, et al. Ethnopharmacology, phytochemistry, and global distribution of mangroves―A comprehensive review. Marine drugs. 2019;17(4):231.

21. Chakraborty K. Chemistry and biological activities of rhizophora mucronata lam. Bioactive Compounds in Mangroves and their Associates: Springer; 2025. p. 1-26.
22. Sadeer NB, Rocchetti G, Senizza B, Montesano D, Zengin G, Uysal A, et al. Untargeted metabolomic profiling, multivariate analysis and biological evaluation of the true mangrove (Rhizophora mucronata Lam.). Antioxidants. 2019;8(10):489.
23. Khatun MCS, Muhit MA, Maruf MMH, Iqbal MA, Rahman SA. Pharmacological evaluation of mangrove plant Rhizophora mucronata (Lam.) grown in the coastal area of Sundarbans. Plos one. 2026;21(1):e0340646.
24. Suganthy N, Pandima Devi K. In vitro antioxidant and anti-cholinesterase activities of Rhizophora mucronata. Pharmaceutical biology. 2016;54(1):118-29.
25. Li X, Zhang D, Hannink M, Beamer LJ. Crystal structure of the Kelch domain of human Keap1. Journal of Biological Chemistry. 2004;279(52):54750-8.
26. Padmanabhan B, Tong KI, Ohta T, Nakamura Y, Scharlock M, Ohtsuji M, et al. Structural basis for defects of Keap1 activity provoked by its point mutations in lung cancer. Molecular cell. 2006;21(5):689-700.
27. Mi W, Guan H, Lyu J, Zhao D, Xi Y, Jiang S, et al. YEATS2 links histone acetylation to tumorigenesis of non-small cell lung cancer. Nature communications. 2017;8(1):1088.
28. Kohn W, Becke AD, Parr RG. Density functional theory of electronic structure. The journal of physical chemistry. 1996;100(31):12974-80.
29. Parr R, Yang W. Horizons of quantum chemistry. Horizons of Quantum Chemistry Springer https://doi org/101007/978-94-009-9027-2. 1980.
30. Parr RG, Yang W. Density functional approach to the frontier-electron theory of chemical reactivity. Journal of the American Chemical Society. 1984;106(14):4049-50.
31. Padmanabhan B, Tong KI, Ohta T, Nakamura Y, Scharlock M, Ohtsuji M, et al. Structural basis for defects of Keap1 activity provoked by its point mutations in lung cancer. 2006;21(5):689-700.
32. Mi W, Guan H, Lyu J, Zhao D, Xi Y, Jiang S, et al. YEATS2 links histone acetylation to tumorigenesis of non-small cell lung cancer. 2017;8(1):1088.
33. Spassov V, Yan L. Accelrys Software Inc., discovery studio modeling environment, release 4.0. Proteins Struct Funct Bioinforma. 2013;81:704-14.

34. Sandor M, Kiss R, Keserű GrM. Virtual fragment docking by Glide: a validation study on 190 protein− fragment complexes. Journal of Chemical Information and Modeling. 2010;50(6):1165-72.
35. Bowers KJ, Chow E, Xu H, Dror RO, Eastwood MP, Gregersen BA, et al., editors. Scalable algorithms for molecular dynamics simulations on commodity clusters. Proceedings of the 2006 ACM/IEEE Conference on Supercomputing; 2006.
36. Daina A, Michielin O, Zoete V. SwissADME: a free web tool to evaluate pharmacokinetics, drug-likeness and medicinal chemistry friendliness of small molecules. Scientific reports. 2017;7(1):42717.
37. Tsaioun K, Blaauboer BJ, Hartung T. Evidence-based absorption, distribution, metabolism, excretion (ADME) and its interplay with alternative toxicity methods. ALTEX-Alternatives to animal experimentation. 2016;33(4):343-58.
38. Chandrasekaran B, Abed SN, Al-Attraqchi O, Kuche K, Tekade RK. Computer-aided prediction of pharmacokinetic (ADMET) properties. Dosage form design parameters: Elsevier; 2018. p. 731-55.
39. Pires DE, Blundell TL, Ascher DB. pkCSM: predicting small-molecule pharmacokinetic and toxicity properties using graph-based signatures. Journal of medicinal chemistry. 2015;58(9):4066-72.
40. López J, Anitescu C, Valizadeh N, Rabczuk T, Alajlan N. Structural shape optimization using Bézier triangles and a CAD-compatible boundary representation. Engineering with Computers. 2020;36:1657-72.
41. Kumer A, Ahmed B, Sharif M, Al-Mamun A. A theoretical study of aniline and nitrobenzene by computational overview. Asian Journal of Physical and Chemical Sciences. 2017;4(2):1-12.
42. Kumer A, Paul S, Sarker MN, Islam MJ. The prediction of thermo physical, vibrational spectroscopy, chemical reactivity, biological properties of morpholinium borate, phosphate, chloride and bromide ionic liquid: a DFT study. International Journal of New Chemistry. 2019;6(4):236-53.
43. KUMER A, Sarker MN, Sunanda P. The theoretical investigation of HOMO, LUMO, thermophysical properties and QSAR study of some aromatic carboxylic acids using HyperChem programming. International Journal of Chemistry and Technology. 2019;3(1):26-37.

44. Kumer A, Sarker N, Paul S, Zannat A. The theoretical prediction of thermophysical properties, HOMO, LUMO, QSAR and biological indics of cannabinoids (CBD) and tetrahhdrocannabinol (THC) by computational chemistry. Advanced Journal of Chemistry-Section A. 2019;2(3):190-202.
45. Hoque MM, Kumer A, Hussen MS, Khan MW. Theoretical Evaluation of 5, 6-Diaroylisoindoline-1, 3-dione as Potential Carcinogenic Kinase PAK1 Inhibitor: DFT Calculation, Molecular Docking Study and ADMET Prediction. International Journal of Advanced Biological and Biomedical Research. 2021;9(1):77-104.
46. Islam MJ, Sarker MN, Kumer A, Paul S. The Evaluation and Comparison of Thermo-Physical, Chemical and Biological Properties of Palladium (II) Complexes on Binuclear Amine Ligands with Different Anions by DFT Study. International journal of Advanced Biological and Biomedical Research. 2019;7(4):315-34.
47. Kumer A, Chakma U, Chandro A, Howlader D, Akash S, Kobir ME, et al. MODIFIED D-GLUCOFURANOSE COMPUTATIONALLY SCREENING FOR INHIBITOR OF BREAST CANCER AND TRIPLE BREAST CANCER: CHEMICAL DESCRIPTOR, MOLECULAR DOCKING, MOLECULAR DYNAMICS AND QSAR. Journal of the Chilean Chemical Society. 2022;67(3):5623-35.
48. Kumer A, Chakma U, Rana MM, Chandro A, Akash S, Elseehy MM, et al. Investigation of the New Inhibitors by Sulfadiazine and Modified Derivatives of α-D-glucopyranoside for White Spot Syndrome Virus Disease of Shrimp by In Silico: Quantum Calculations, Molecular Docking, ADMET and Molecular Dynamics Study. Molecules. 2022;27(12):3694.
49. Kumer A, Jahidul M, Paul S. Effect of external electric field and temperature on entropy, heat of capacity, and chemical reactivity with QSAR study of morphonium chloride and nitrous ionic liquids crystal using DFT. Chemical Methodologies. 2020;4:595-604.
50. Cheng K, Zheng Q-Z, Qian Y, Shi L, Zhao J, Zhu H-L. Synthesis, antibacterial activities and molecular docking studies of peptide and Schiff bases as targeted antibiotics. Bioorganic & medicinal chemistry. 2009;17(23):7861-71.
51. Hermann T, Westhof E. Docking of cationic antibiotics to negatively charged pockets in RNA folds. Journal of medicinal chemistry. 1999;42(7):1250-61.

52. Yang Y, Yao K, Repasky MP, Leswing K, Abel R, Shoichet BK, et al. Efficient exploration of chemical space with docking and deep learning. Journal of Chemical Theory and Computation. 2021;17(11):7106-19.
53. Friesner RA, Murphy RB, Repasky MP, Frye LL, Greenwood JR, Halgren TA, et al. Extra precision glide: Docking and scoring incorporating a model of hydrophobic enclosure for protein−ligand complexes. Journal of medicinal chemistry. 2006;49(21):6177-96.
54. Friesner RA, Banks JL, Murphy RB, Halgren TA, Klicic JJ, Mainz DT, et al. Glide: a new approach for rapid, accurate docking and scoring. 1. Method and assessment of docking accuracy. Journal of medicinal chemistry. 2004;47(7):1739-49.
55. Alzain AA, Mukhtar RM, Abdelmoniem N, Shoaib TH, Osman W, Alsulaimany M, et al. Modulation of NRF2/KEAP1-mediated oxidative stress for cancer treatment by natural products using pharmacophore-based screening, molecular docking, and molecular dynamics studies. Molecules. 2023;28(16):6003.
56. Brogi S, Guarino I, Flori L, Sirous H, Calderone V. In silico identification of natural products and world-approved drugs targeting the KEAP1/NRF2 pathway endowed with potential antioxidant profile. Computation. 2023;11(12):255.
57. Boyenle ID, Divine UC, Adeyemi R, Ayinde KS, Olaoba OT, Apu C, et al. Direct Keap1-kelch inhibitors as potential drug candidates for oxidative stress-orchestrated diseases: A review on In silico perspective. Pharmacological Research. 2021;167:105577.
58. Li X, Li X-M, Jiang Y, Liu Z, Cui Y, Fung KY, et al. Structure-guided development of YEATS domain inhibitors by targeting π-π-π stacking. Nature chemical biology. 2018;14(12):1140-9.
59. Wang X, Cheng G, Zhao J, Gao P, Mao H, Yuan C, et al. Discovering potential inhibitors of the YEATS domain of YEATS2 through virtual screening, molecular optimization and molecular dynamics simulations. New Journal of Chemistry. 2023;47(42):19447-60.
60. Yang H, Lou C, Sun L, Li J, Cai Y, Wang Z, et al. admetSAR 2.0: web-service for prediction and optimization of chemical ADMET properties. Bioinformatics. 2019;35(6):1067-9.
61. Cheng F, Li W, Zhou Y, Shen J, Wu Z, Liu G, et al. admetSAR: a comprehensive source and free tool for assessment of chemical ADMET properties. ACS Publications; 2012.

Response to the Academic Editor and Reviewers

Manuscript ID: PONE-D-26-14113R1

Title: *Investigating the Anticancer Potential of Phytochemicals from the Mangrove Rhizophora mucronata as Inhibitors of Lung Cancer-Associated Proteins: Insights from Multiscale Computational and In Silico Analyses*

Dear Academic Editor,

We sincerely thank you and the reviewers for the careful evaluation of our revised manuscript and for the constructive comments. We greatly appreciate the time and effort devoted to assessing our work. The comments were valuable in identifying several remaining issues related to manuscript organization, scientific terminology, methodological clarity, data presentation, and interpretation.

We have carefully revised the manuscript accordingly. In particular, we consolidated the Introduction, improved the academic tone, standardized the biological nomenclature, clarified the independent AutoDock Vina and Schrödinger Glide workflows, completed the molecular dynamics methodology, corrected the DFT equations and descriptors, removed duplicated and inappropriate text, reconciled the docking results obtained from the two docking platforms, corrected the protein–ligand interaction discussion, and revised the benchmarking section to avoid inappropriate comparison of docking scores with logP.

Our point-by-point responses are provided below. The relevant section of the revised manuscript is identified after each response.

Reviewer #1

We sincerely thank Reviewer #1 for confirming that the previous concerns were adequately addressed and for recommending the manuscript for publication. We greatly appreciate the reviewer's positive assessment of the revised work.

Reviewer #2

Comment: Redundancy in the Introduction

The reviewer noted that epidemiological information, survival statistics, and the limitations of existing therapies were repeated across the opening paragraphs.

Response:
Thank you for this observation. We agree that the earlier version contained unnecessary repetition. The Introduction has been substantially condensed and reorganized. Repeated information regarding global lung cancer mortality, five-year survival, major risk factors, and limitations of existing treatments has been consolidated. The revised Introduction now progresses more directly from the clinical burden of lung cancer to the rationale for investigating natural-product-derived compounds and finally to the selection of Keap1 and YEATS2.

Location of correction: Section 1, *Introduction*, opening paragraphs.

Comment: Colloquial and non-academic expressions

The reviewer identified several expressions that were too conversational for a scientific manuscript.

Response:
We agree and have revised the manuscript extensively to improve its academic tone. Conversational expressions and informal analogies were removed and replaced with concise scientific language. Statements such as “lets us compare,” “grows silently,” and other informal descriptions were rewritten using formal biomedical terminology. Similar revisions were made throughout the Methods, Results and Discussion sections.

Location of correction: Throughout the manuscript, particularly Sections 1, 2 and 3.

Comment: Biological nomenclature of *Rhizophora mucronata*

The reviewer requested that the genus and species name be italicized throughout.

Response:
Thank you. The scientific name has been standardized throughout the revised manuscript. The full name *Rhizophora mucronata* is used at first mention where appropriate, followed by the abbreviated form *R. mucronata*. Both forms are italicized consistently.

Location of correction: Title, Abstract, Keywords, Introduction, Methods, Results and Discussion, Conclusion, and relevant figure/table captions.

Comment: Semantic error in the phrase “This study recovers a critical knowledge gap”

Response:
We agree. The inappropriate expression has been removed. The revised manuscript now states that the study “addresses a critical knowledge gap” rather than “recovers” a gap.

Location of correction: Section 1, *Introduction*, final part of the study-rationale paragraph.

Comment: Grammatical sentence fragments in the methodology

The reviewer identified incomplete expressions such as “Used the $DMol^3$ module…,” “Used the Keap1…,” and “Molecular docking was did…”.

Response:
Thank you. These sentence fragments have been rewritten using complete formal scientific sentences and appropriate passive constructions. The DFT, receptor-preparation, AutoDock Vina, and Glide procedures are now described as complete methodological workflows.

Location of correction: Sections 2.1, *Optimization and Ligand Preparation*; 2.2, *Protein Preparation and Collection*; and 2.3, *Molecular Docking and Visualization*.

Comment: Contradiction concerning Discovery Studio and Schrödinger receptor preparation

The reviewer questioned whether receptors prepared only in Discovery Studio were directly used for Glide docking.

Response:
We thank the reviewer for highlighting this important methodological point. The receptors for AutoDock Vina and Glide were prepared independently using platform-specific workflows.

For AutoDock Vina, receptor preparation was performed using Discovery Studio, including removal of non-essential crystallographic molecules and energy minimization.

For Glide docking, the receptor structures were independently processed in Schrödinger Maestro using the Protein Preparation Wizard. This workflow included assignment of bond orders, addition of hydrogen atoms, optimization of the hydrogen-bonding network, removal of unnecessary water molecules, and restrained energy minimization. The resulting structures were subsequently used for receptor-grid generation and Glide Standard Precision docking.

This distinction is now explicitly reported in the manuscript.

Location of correction: Section 2.2, *Protein Preparation and Collection*, and Section 2.3.2, *Molecular Docking by Glide from Schrödinger Suite*.

Comment: Duplicate Table 2 heading and incomplete Molecular Dynamics methodology

Response:
Thank you. The duplicated Table 2 heading has been removed.

The Molecular Dynamics methodology has also been completed and expanded. The revised section now specifies the selected complexes, 100-ns simulation duration, Desmond version, OPLS-AA force field, TIP3P solvent model, orthorhombic periodic box with a 10 Å buffer, system neutralization, energy minimization, NVT and NPT equilibration, simulation temperature and pressure, thermostat and barostat, integration time step, SHAKE constraints, and the trajectory parameters evaluated.

Location of correction: Table 2 and Section 2.4, *Molecular Dynamics*.

Comment: Poor formatting of Equations 4–8

Response:
We agree. Equations 1–8 have been reformatted and standardized. The notation for HOMO–LUMO energy gap, ionization potential, electron affinity, electronegativity, chemical potential, hardness, softness, and electrophilicity has been corrected and presented consistently.

The equations are now defined as:

$\Delta Egap = ELUMO - EHOMO$
$I = -EHOMO$
$A = -ELUMO$
$\chi = (I + A)/2$
$\mu = -(I + A)/2$
$\eta = (I - A)/2$
$S = 1/\eta$
$\omega = \mu^2/(2\eta)$

Location of correction: Section 2.1, *Optimization and Ligand Preparation,* Equations 1–8.

Comment: Sign and variable inconsistencies in the DFT descriptors

The reviewer noted inconsistencies involving ionization potential, electron affinity, hardness, softness, electrophilicity, and the HOMO/LUMO values.

Response:
We appreciate this important observation. The DFT-derived descriptors were rechecked against the original HOMO and LUMO outputs and recalculated using the equations defined in the Methods. Table 3 has been revised accordingly so that the reported ionization potential, electron affinity, hardness, softness, chemical potential, electronegativity, and electrophilicity values are internally consistent with the underlying frontier orbital energies.

We also revised the accompanying discussion to avoid interpreting the HOMO–LUMO gap or related DFT descriptors as direct indicators of anticancer activity. These quantities are now described as supportive physicochemical descriptors of intrinsic electronic properties.

Location of correction: Section 3.1.2, *HOMO, LUMO, and Chemical Reactivity Descriptors*, and Table 3.

Comment: Duplicate paragraphs in the DFT section

Response:
Thank you. The duplicated text has been removed. The DFT discussion has been consolidated into a single concise interpretation emphasizing electronic stability and reactivity while avoiding overinterpretation of these descriptors as predictors of biological activity.

Location of correction: Section 3.1.2, immediately following Table 3.

Comment: Use of the term "fungicidal ligands"

Response:
We agree that this was an inappropriate copy-paste artifact unrelated to the present anticancer study. All references to "fungicidal ligands," "fungicidal candidates," and related terminology have been removed. The compounds are now consistently described as phytochemicals, screened ligands, or candidate anticancer compounds as appropriate.

Location of correction: Sections 3.1.3 and 3.2.1.

Comment: Section 3.1.4 was truncated

Response:
Thank you. Section 3.1.4 has been completed and revised so that the discussion of the electrostatic potential/FMO maps is presented as a complete paragraph and appropriately linked to the corresponding figures.

Location of correction: Section 3.1.4, *Electrostatic Potential Map for Frontier Molecular Orbital*.

Comment: Glide discussion duplicated the AutoDock Vina discussion

Response:
We agree. The duplicated AutoDock Vina material has been removed from the Glide subsection. Section 3.2.5 now discusses only the Glide-selected 1X2J–L10 and 5XNV–L02 complexes and their corresponding interaction patterns.

Location of correction: Section 3.2.5, *Protein–Ligand Interaction from Glide Docking*.

Comment: Different lead ligands were identified by PyRx and Glide without explanation

Response:
Thank you for raising this important issue. We have now explicitly explained why different ligand rankings were obtained from the two docking platforms.

AutoDock Vina identified L11 and L14 as the highest-ranked ligands against 1X2J and 5XNV, respectively, whereas Glide identified L10 and L02 as the highest-ranked ligands against the corresponding targets. These differences are expected because AutoDock Vina and Glide use different receptor-preparation procedures, conformational-search algorithms, scoring functions, and treatments of intermolecular interactions.

The two docking approaches are therefore interpreted as complementary rather than numerically interchangeable. To maintain methodological consistency, the Glide-ranked complexes 1X2J–L10 and 5XNV–L02 were selected for subsequent 100-ns molecular dynamics simulations.

Location of correction: Section 3.2.2, immediately following Table 5, and Section 3.3.1, *RMSD*.

Comment: Contradictory description of L12 hydrogen bonding

The reviewer noted that L12 was initially described as forming no hydrogen bonds but was subsequently described as forming several hydrogen bonds.

Response:
We agree and have corrected this inconsistency. According to the interaction data reported in Table 4, L12 against 5XNV forms two hydrogen bonds and four hydrophobic contacts, with a PyRx docking score of −6.0 kcal/mol. L12 is therefore now described consistently using these interaction values.

We also clarify that L12 is shown as a representative YEATS2-binding pose and was not the highest-ranked PyRx ligand for 5XNV; L14 was the highest-ranked ligand for this target in the AutoDock Vina analysis.

Location of correction: Section 3.2.4, *Protein–Ligand Interaction by PyRx Docking*, and Figure 6 discussion.

Comment: Conceptual error involving docking energy and octanol–water partition coefficient

Response:
We fully agree with the reviewer. The previous wording incorrectly associated docking energies expressed in kcal/mol with the octanol–water partition coefficient.

The benchmarking section has been revised to clearly distinguish docking scores from logP. Docking scores are software-dependent scoring quantities used for ranking predicted binding poses, whereas logP is a dimensionless measure of molecular lipophilicity. No direct equivalence between these quantities is now implied.

Reported Keap1 and YEATS-domain inhibitors are used primarily as qualitative structural and pharmacological references, and we explicitly state that docking scores obtained using different protocols should not be interpreted as experimentally measured binding free energies.

Location of correction: Section 3.2.6, *Benchmarking with Known Inhibitors*.

Reviewer #3

Comment 1: The rationale for selecting the protein targets was insufficiently summarized

Response:
We thank the reviewer for this important suggestion. The Introduction has been expanded to provide a concise mechanistic rationale for selecting Keap1 and YEATS2.

Keap1 was selected because it is a principal negative regulator of Nrf2, and disruption of the Keap1–Nrf2 pathway is frequently associated with NSCLC, enhanced antioxidant defense, metabolic adaptation, cell survival, and treatment resistance. YEATS2 was selected because it functions as an epigenetic reader of H3K27 acetylation and has been associated with oncogenic transcriptional regulation, proliferation, and survival in NSCLC.

The revised manuscript therefore presents Keap1 and YEATS2 as complementary targets representing redox-regulatory and epigenetic mechanisms relevant to NSCLC pathogenesis.

Location of correction: Section 1, *Introduction*, final two paragraphs.

Comment 9: The locations of the revisions were not specified

Response:
We apologize for this omission in the previous response. In the present rebuttal, the location of each substantive revision has been explicitly identified by manuscript section and subsection. Major revisions can be found in:

Section 1 – Introduction: consolidation of epidemiological information, formalization of academic language, and expanded rationale for Keap1 and YEATS2.

Section 2.1 – Optimization and Ligand Preparation: corrected DFT methodology and equations.

Section 2.2 – Protein Preparation and Collection: clarified independent AutoDock Vina and Schrödinger Protein Preparation Wizard workflows.

Section 2.3 – Molecular Docking: clarified AutoDock Vina and Glide protocols.

Section 2.4 – Molecular Dynamics: completed simulation methodology and parameters.

Section 3.1.2 – HOMO, LUMO, and Chemical Reactivity Descriptors: corrected DFT descriptors and revised interpretation.

Section 3.1.3–3.1.4: removed inappropriate terminology and completed FMO/electrostatic-potential discussion.

Sections 3.2.1–3.2.6 – Molecular Docking: reconciled AutoDock Vina and Glide results, corrected interaction counts, clarified ligand prioritization, and revised benchmarking.

Section 3.3 – Molecular Dynamics: clarified the rationale for selecting 1X2J–L10 and 5XNV–L02 for simulation.

We have also provided both a marked-up manuscript showing all revisions and a clean version with the revisions incorporated.

Closing Response

We sincerely thank the Academic Editor and all reviewers for their constructive and detailed comments. Their suggestions have substantially improved the clarity, methodological transparency, internal consistency, and scientific interpretation of the manuscript.

We have carefully revised the manuscript to ensure that computational predictions are presented conservatively and are not interpreted as direct evidence of biological efficacy. We believe that the revised manuscript now provides a clearer distinction between DFT-derived electronic descriptors, docking scores, molecular dynamics stability, and experimentally measurable biological activity.

We hope that the revised manuscript and detailed responses satisfactorily address all remaining concerns, and we respectfully submit the manuscript for further consideration for publication in PLOS ONE.

Sincerely,

Ajoy Kumer
Corresponding Author
on behalf of all authors
