# Supplementary material for: Investigating the anticancer potential of phytochemicals from the mangrove Rhizophora mucronata as inhibitors of lung cancer-associated proteins: Insights from multiscale computational and in silico analyses
Source: PLoS One. 2026 Oct 7;21(10):e0359525. doi: 10.1371/journal.pone.0359525 (PMC13645060; doi:10.1371/journal.pone.0359525)
Supplement: Supplementary file 8 [file pone.0359525.s007.docx]

I acknowledge the effort of the authors on this study. This manuscript presents a multi-layered computational investigation—including density functional theory (DFT), molecular docking, molecular dynamics (MD) simulations, and ADMET prediction—to identify potential anti-cancer compounds derived from *Rhizophora mucronata* for lung cancer targets. While the topic is relevant and the computational workflow aligns with commonly used *in silico* screening pipelines, the study suffers from substantial conceptual inconsistencies, limited biological grounding, and significant overinterpretation of computational outputs. In its current form, the manuscript does not provide sufficient evidence to support its central claims of therapeutic relevance.

**Major Comments**

1. **Page 3, lines 60–68:** **Wrong classification:** A fundamental issue arises from the characterization of the selected targets as “lung cancer proteases.” As described on page 3, lines 60–68, the study focuses on Keap1 (PDB: 1X2J) and YEATS2 (PDB: 5XNV). While both proteins have recognized roles in cancer biology—particularly in oxidative stress regulation and epigenetic transcriptional control—they are not proteases. The repeated classification of these targets as proteases throughout the manuscript (e.g., page 1, lines 10–12; page 2, lines 25–27) reflects a conceptual error that undermines the scientific framing of the study. This mischaracterization should be corrected, and the rationale for selecting these targets should be expanded to reflect their specific roles in lung cancer pathogenesis, including their relevance to defined molecular subtypes of non-small cell lung cancer (NSCLC).
2. **Lines 140–148:** **The interpretation of docking results is also problematic**. On page 6, lines 140–148, the authors report binding affinities in the range of approximately −5.4 to −7.8 kcal/mol and subsequently infer that ligand L10 represents a potent inhibitor. However, these values fall within a moderate binding range and are not contextualized against known inhibitors or co-crystallized ligands. The absence of benchmarking makes it difficult to assess the significance of these findings. More critically, the manuscript extends these observations into unsupported claims of therapeutic superiority (page 12, lines 310–315), suggesting that the identified compounds may outperform existing NSCLC chemotherapeutics. This conclusion is not justified by docking data alone, as binding affinity predictions do not directly translate to biological activity or clinical efficacy. A more cautious interpretation is required, and inclusion of comparative controls would significantly strengthen the analysis.
3. **Lines 110–125:** **The reliance on DFT-derived descriptors is similarly overstated**. As presented on page 5, lines 110–125, parameters such as HOMO–LUMO gap, electrophilicity, and chemical hardness are used to rank compound efficacy, with L10 identified as the most promising candidate based on its “reactivity.” While these descriptors provide insight into electronic properties, they are not validated predictors of anticancer activity. The assertion that a lower HOMO–LUMO gap implies superior therapeutic potential reflects an oversimplification and should be revised. These results would be more appropriately presented as supportive chemical characterization rather than as determinants of biological performance.
4. **Lines 180–200:** The molecular dynamics analysis, described on page 8, lines 180–200, reports RMSD stability below 3 Å over a 100 ns simulation, which is interpreted as evidence of stable ligand–protein interaction. However, RMSD alone is insufficient to establish binding strength or specificity. The analysis lacks deeper interrogation, such as binding free energy calculations (e.g., MM-PBSA), residue-level interaction mapping, or comparison with apo protein dynamics. Without these elements, the MD component remains descriptive rather than mechanistically informative.
5. **Lines 240–255:** **The ADMET analysis further illustrates a pattern of overinterpretation**. On page 10, lines 240–255, the authors conclude that the lead compounds exhibit high oral bioavailability and negligible toxicity, and again suggest superiority over existing chemotherapeutics. These conclusions are based solely on predictive tools such as SwissADME and pkCSM, which provide preliminary estimates rather than definitive pharmacokinetic or toxicological profiles. The comparison to clinically approved drugs is therefore inappropriate in the absence of experimental validation, and these claims should be substantially tempered.
6. It’s very unfortunate that a broader limitation of the manuscript is the lack of integration with lung cancer biology, the central thematic content! Although the targets are introduced, there is minimal discussion of how their inhibition would influence tumor progression, treatment response, or resistance mechanisms. For instance, the implications of modulating the Keap1–Nrf2 pathway or disrupting YEATS2-mediated transcription are not explored in the context of tumor microenvironment, oxidative stress adaptation, or therapeutic synergy. This absence of biological interpretation reduces the translational relevance of the findings.
7. **Lines 330–335:** Finally, the manuscript does not provide a clear pathway toward experimental validation. While it is noted on page 13, lines 330–335 that the findings “lay the groundwork for future wet-lab studies,” no specific experimental strategies are outlined. The inclusion of a proposed validation framework—such as testing in established NSCLC cell lines (e.g., A549 or H1975), assessing pathway modulation, or evaluating cytotoxicity—would significantly strengthen the manuscript’s impact.

**Other important areas of concern:**

**Methodological and Reporting Concerns**

Additional methodological concerns are evident throughout the manuscript. The ligand selection process, described on page 4, lines 90–100, lacks a clear rationale, with only 14 compounds screened and no discussion of chemical diversity or selection criteria. The docking methodology is insufficiently detailed; for example, page 6, lines 130–135 does not provide grid box parameters, protocol validation, or redocking results, limiting reproducibility. Furthermore, the statement on page 14, lines 360–362 that “data availability is not applicable” is inconsistent with current standards for computational research, where input structures, parameters, and output files should be made accessible.

**Conclusion and Recommendation**

In summary, while the manuscript employs a standard and technically acceptable computational pipeline, however, it is limited by conceptual inaccuracies, insufficient methodological transparency, and substantial overinterpretation of *in silico* results. The conclusions extend well beyond the level of evidence provided, particularly with respect to claims of therapeutic efficacy and clinical relevance. A major revision is required to correct target misclassification, contextualize computational findings, and align interpretations with the limitations of the methods used.

With significant revision and a more restrained, biologically grounded interpretation, the study could contribute as a preliminary computational exploration. However, in its current form, it does not meet the standards expected for publication in its present scope.
