# Supplementary material for: Investigating the anticancer potential of phytochemicals from the mangrove Rhizophora mucronata as inhibitors of lung cancer-associated proteins: Insights from multiscale computational and in silico analyses
Source: PLoS One. 2026 Oct 7;21(10):e0359525. doi: 10.1371/journal.pone.0359525 (PMC13645060; doi:10.1371/journal.pone.0359525)
Supplement: Supplementary file 9 [file pone.0359525.s008.docx]

**Response to Reviewers**

Dear Academic Editor and Reviewers,

We are grateful to the Academic Editor and all three reviewers for their detailed and constructive comments, which have significantly improved the quality and scientific rigor of our manuscript. We have carefully addressed every point raised. Below is a point-by-point response.

**Reviewer #1**

**Q1) There are few typos’ errors.**
**Reply:** We have carefully proofread the entire manuscript and corrected all typographical and spelling errors. The revised version has been checked by multiple co-authors to ensure language accuracy.

**Q2) Author should check “DMol³ module in Materials Studio to do DFT calculations with the B3LYP functional and the 6–31G basis set. Used the COSMO model”: *
Reply:** We have verified and clarified the methodology. In the revised manuscript (Section 2.1), we explicitly describe the use of DMol³ with the B3LYP functional, the 6-31G* basis set, and the COSMO solvation model where needed. The text now reads: *“Used the DMol³ module in Materials Studio to perform DFT calculations with the B3LYP functional and the 6–31G* basis set. The COSMO model was employed to include solvent effects when required…” *

**Q3) Why did author use solvent effects? For optimization, it might be errors.**
**Reply:** We used the COSMO model to mimic an aqueous environment (water, dielectric constant 78.54), which better represents the physiological conditions under which ligand–protein interactions occur. To avoid optimization errors, we tightened convergence thresholds (energy, gradient, and displacement) and verified that all optimized structures had no imaginary frequencies, confirming true minima. This justification has been added to Section 2.1.

**Q4) The correlation of DFT study is their biological activity need more clearly explain.**
**Reply:** We have substantially revised the interpretation of DFT results. In Section 3.1.2, we now explicitly state that DFT-derived descriptors (HOMO–LUMO gap, electrophilicity, etc.) provide only supportive chemical characterization and are not direct predictors of biological or anticancer activity. We caution that a smaller HOMO–LUMO gap should not be interpreted as implying superior therapeutic potential. These electronic properties are discussed as complementary to docking and dynamics data.

**Q5) Make a more explanation for Protein-Ligand Interaction for PyRx in view of superpose/position.**
**Reply:** We have expanded Section 3.2.3 to better explain the superposition analysis. The text now describes how docked poses of all ligands were aligned, compared for common binding modes, and how this approach reveals pharmacophoric hot spots and ligand-specific interaction patterns within the Keap1 and YEATS2 binding pockets.

**Q6) Why author did PyRx and Glide from Schrodinger suite docking?**
**Reply:** We used two different docking engines—AutoDock Vina (via PyRx) and Glide (Schrödinger)—as an orthogonal validation strategy. PyRx provides a rapid, widely used initial screening, while Glide employs a more sophisticated scoring function that accounts for desolvation, hydrophobic enclosure, and polar interactions. The consistent identification of top ligands across both platforms strengthens confidence in the predicted binding modes. This rationale is now clarified in Sections 2.3.1 and 2.3.2.

**Q7) Explain the validation docking, protein.**
**Reply:** We have added a sentence in the Methodology (Section 2.3) stating that the docking protocols were validated by redocking the co-crystallized ligands into their respective proteins. The docked poses reproduced the experimental binding modes with RMSD values < 2.0 Å, confirming the ability of both AutoDock Vina and Glide to accurately recapitulate known interactions.

**Q8) What are the technical validation of ligand?**
**Reply:** Ligands underwent rigorous preparation: initial geometry optimization using the Universal Force Field (UFF) to eliminate steric clashes, followed by DFT optimization at the B3LYP/6-31G* level to obtain minimum-energy conformations. For Glide docking, LigPrep generated correct ionization states at pH 7.4, enumerated tautomers, and produced low-energy 3D conformers. All optimized structures were free of imaginary frequencies, confirming they correspond to true energy minima. This process is detailed in Sections 2.1 and 2.3.2.

**Q9) It must add the Ramachandran for protein before Docking of protein with ligand, then compare the stability before and after docking.**
**Reply:** We have added Ramachandran plots for the docked protein–ligand complexes (Figure 13). In the revised manuscript, Table 1 provides the initial Ramachandran statistics for the crystal structures (1X2J: 96.18% favored; 5XNV: 96.92% favored). The post-docking Ramachandran analysis (Figure 13) shows that the backbone dihedral angles remain in favored or additionally allowed regions for >95% of residues in both complexes, indicating that ligand binding does not perturb the native backbone geometry. A direct comparison with wild-type distributions is now discussed in Section 3.4.

**Q10) The section provides a detailed interpretation of pharmacokinetic predictions, but it could be strengthened by including a clearer comparative discussion between ligands.**
**Reply:** We have added a comparative discussion in Section 3.5.2, explicitly identifying ligands with the most balanced ADME profiles. For example, L01, L02, L06, and L09 exhibit high intestinal absorption and Caco-2 permeability with relatively low CYP inhibition risk, while L07 and L08 have higher tPSA values but acceptable bioavailability. The revised text now highlights which compounds show the most favorable overall pharmacokinetic profiles, improving clarity and practical insight.

**Reviewer #2**

**Q1. The title is overly long and contains redundant phrasing (“multi-scale computational” and “in silico”) ambiguous phrase: “lung cancer protease inhibitors”.**
**Reply:** We have revised the title to: *“Investigating the anti-cancer properties of phytochemicals from mangrove tree, Rhizophora mucronata, as Lung Cancer Protein Inhibitors: Insights from Multi-scale Computational and In silico Analysis”*. This version removes redundancy, corrects the misclassification of targets, and accurately reflects the scope.

**Q2. The abstract lacks a clear structured flow, includes excessive methodological detail, and contains grammatical errors and repetition. Overstates conclusions.**
**Reply:** The abstract has been completely rewritten following a clear logical flow: background → methods → key results → conclusion. We condensed methodological details (e.g., specific basis sets, software names) and removed all overstated clinical claims. The revised abstract focuses on the main findings without suggesting direct therapeutic superiority.

**Q3. Rewrite the abstract following a logical structure (background → methods → results → conclusion).**
**Reply:** Done. The abstract now adheres to this structure.

**Q4. Focus on key findings rather than technical detail and avoid overstated clinical claims.**
**Reply:** We have removed excessive technical detail (e.g., “B3LYP/6-31G*”, “COSMO model”) from the abstract and eliminated unsupported claims about patient survival or superiority over existing drugs.

**Q5. The Introduction is comprehensive and well-referenced…**
**Reply:** We thank the reviewer for this positive comment and have preserved the well-referenced content while addressing the concerns below.

**Q6. However, it is overly long (repeats epidemiological statistics multiple times, over-explains well-known facts), stylistically inconsistent, and occasionally imprecise.**
**Reply:** We have significantly shortened the Introduction by removing redundant epidemiological statistics and condensing the general lung cancer background. The length has been reduced by approximately 30%, improving focus and readability.

**Q7. The language and Style Inconsistency.**
**Reply:** We have carefully edited the entire manuscript to ensure a uniform, formal scientific tone throughout.

**Q8. There is a noticeable shift between informal/plain phrasing “Lung cancer often grows silently…” and highly technical language.**
**Reply:** All informal phrasing has been replaced. For instance, “Lung cancer often grows silently…” has been rephrased to a more precise description of asymptomatic progression and late-stage diagnosis.

**Q9. Authors should maintain a uniform scientific tone throughout.**
**Reply:** We have thoroughly revised the manuscript to maintain a consistent scientific tone.

**Q10. There is a lot of grammatical and structural errors. Examples:**

- *“Natural products derived phytochemicals, low cost and affordable medicine…”*
- *“This study recovers a critical knowledge gap…”*
- *“screening the phytochemical from Rhizophora mucronata…”*
  **Reply:** All these errors have been corrected. The sentences now read: *“Natural product-derived phytochemicals offer low-cost and affordable medicinal options…”*; *“This study addresses a critical knowledge gap…”*; *“screening phytochemicals from Rhizophora mucronata…”*.

**Q11. These issues persist throughout and require editing.**
**Reply:** The entire manuscript has undergone rigorous language editing, and we trust that the revised version meets the required standard.

**Q12. The study repeatedly frames targets as “proteases”, but Keap1 (PDB: 1X2J) is not a protease, and YEATS2 (PDB: 5XNV) is an epigenetic reader protein, not a protease.**
**Reply:** We sincerely apologize for this fundamental error. We have completely corrected the misclassification throughout the manuscript. The title, abstract, introduction, methodology, results, and conclusion now consistently refer to these proteins as “lung cancer-associated proteins” and the inhibitors as “protein inhibitors” or “target-specific inhibitors”. We have added explicit clarification in the Introduction that Keap1 is a substrate adaptor for a ubiquitin ligase complex and YEATS2 is a histone acetylation reader—neither is a protease.

**Q13. Kindly revise terminology, replacing “protease inhibitors” with “protein inhibitors” or “target-specific inhibitors”.**
**Reply:** Done. All instances of “protease” have been replaced with “protein”.

**Q14. Claims like “no systematic multi-scale investigation has yet explored…” need strong justification with citations.**
**Reply:** We have revised this claim and supported it with appropriate citations. The introduction now states: *“Although mangrove-derived extracts have demonstrated anticancer activity against several cell lines, including lung carcinoma models [20,24], a systematic computational screening of Rhizophora mucronata phytochemicals specifically targeting Keap1 and YEATS2 has not been reported to date.”* This is justified by referencing the existing literature that lacks such an integrated approach.

**Q15. The gap is present but not sharply framed. Introduction should clearly answer: What is known? What is unknown? Why this study matters specifically?**
**Reply:** We have restructured the final paragraph of the Introduction to explicitly answer these questions. It now clearly states (i) known: mangrove extracts show anticancer activity but their molecular targets are unexplored; (ii) unknown: the potential of specific R. mucronata compounds to inhibit Keap1 and YEATS2; (iii) importance: identifying computational leads can accelerate the discovery of novel NSCLC modulators.

**Q16. The choice of 1X2J (Keap1), 5XNV (YEATS2) is actually a strong point, but buried.**
**Reply:** We have moved the rationale for target selection to a prominent, dedicated paragraph in the Introduction and expanded it to highlight their critical roles in NSCLC chemoresistance (Keap1–Nrf2) and epigenetic transcriptional reprogramming (YEATS2). This strategic choice is now clearly communicated.

**Q17. The methodology employs a comprehensive computational workflow… However, the section suffers from substantial issues in clarity, reproducibility, and scientific precision. Key methodological details are missing… A critical concern is the continued misclassification of the selected targets as proteases. Additionally, the ADMET section contains erroneous references to “fungicide” properties.**
**Reply:** We have thoroughly revised the Methodology. The misclassification has been corrected. We removed all inappropriate terminology such as “fungicide” (replaced with “anticancer agent” or “inhibitor”). We have also added missing details: grid box parameters (Table 2), docking validation by redocking, and a clear description of the ligand selection rationale (Section 2.1). The ADMET section no longer contains any reference to fungicide.

**Q18. The Results and Discussion section… suffers from significant issues in clarity, consistency, and scientific interpretation. A critical concern is the persistent misclassification of targets… Notable inconsistencies in reported results across docking platforms, and criteria for lead compound selection are not clearly justified. Several claims overinterpret computational findings.**
**Reply:** We have comprehensively revised the Results and Discussion. The misclassification has been eradicated. Inconsistencies between PyRx and Glide scores are now discussed as expected variations due to different scoring functions, and we present both sets of results transparently. A new subsection “Benchmarking with Known Inhibitors” (3.2.6) has been added to contextualize our docking scores relative to known Keap1 and YEATS2 inhibitors, showing that our compounds are moderate-affinity initial hits. The selection criteria for L10 and L02 are clearly justified based on combined DFT, docking (especially Glide hydrogen bond networks), MD stability, and acceptable ADMET profiles. All overinterpretations have been removed, and conclusions are now appropriately cautious.

**Q19. The section also contains inappropriate terminology (e.g., references to “fungicide” activity) and non-scientific narrative elements.**
**Reply:** All such language has been excised. The revised manuscript uses precise scientific terminology.

**Reviewer #3 (Major Comments)**

**Comment 1. Wrong classification: the study focuses on Keap1 (PDB: 1X2J) and YEATS2 (PDB: 5XNV). Both are not proteases. This mischaracterization should be corrected, and the rationale for selecting these targets should be expanded.**
**Reply:** We have completely corrected this error. The title now reads *“…Lung Cancer Protein Inhibitors”*. Every occurrence of “protease” throughout the manuscript has been replaced with “protein”. We have expanded the Introduction (pages 2–3) to clearly explain the biological roles of Keap1 in the Keap1–Nrf2 pathway (oxidative stress, chemoresistance) and YEATS2 in epigenetic regulation (H3K27ac reader), and why they are mechanistically relevant targets in NSCLC.

**Comment 2. The interpretation of docking results is problematic… binding affinities in the range of −5.4 to −7.8 kcal/mol are moderate and not contextualized against known inhibitors. Unsupported claims of therapeutic superiority.**
**Reply:** We have substantially tempered all claims. A new subsection “Benchmarking with Known Inhibitors” (3.2.6) has been added, which compares our docking scores with literature values for known Keap1 inhibitors (GlideScores < −9.0 kcal/mol for optimized inhibitors) and YEATS2 inhibitors. We now clearly state that our compounds exhibit moderate binding affinities typical of initial natural product screening hits and are generally weaker than many optimized synthetic inhibitors. All suggestions of superiority over existing chemotherapeutics have been removed.

**Comment 3. The reliance on DFT-derived descriptors is similarly overstated… assertion that a lower HOMO–LUMO gap implies superior therapeutic potential reflects an oversimplification.**
**Reply:** We have revised the interpretation in Sections 3.1.2 and 3.1.3. The text now includes an explicit caution: *“However, these DFT-derived parameters provide only supportive insights into the intrinsic electronic characteristics of the compounds. They are not validated predictors of biological or anticancer activity, and a smaller HOMO–LUMO gap should not be interpreted as implying superior therapeutic potential.”*

**Comment 4. MD analysis reports RMSD stability below 3 Å, but lacks deeper interrogation (e.g., MM-PBSA, residue-level mapping, comparison with apo dynamics).**
**Reply:** We acknowledge this limitation. In Section 3.3.1, we now explicitly state that RMSD alone is insufficient to confirm binding strength and that further analyses such as MM-PBSA/MM-GBSA, detailed per-residue energy decomposition, and comparison with apo-protein simulations would be required for deeper mechanistic insight. The MD results are presented as preliminary supportive evidence of complex stability.

**Comment 5. ADMET analysis overinterprets results, concluding high oral bioavailability and negligible toxicity, and suggesting superiority over existing chemotherapeutics.**
**Reply:** All such statements have been removed or heavily moderated. We now describe the ADMET profiles as “acceptable predicted properties suitable for early-stage screening”. We no longer compare our compounds directly to approved drugs or claim negligible toxicity.

**Comment 6. A broader limitation is the lack of integration with lung cancer biology… minimal discussion of how inhibition would influence tumor progression, treatment response, or resistance mechanisms.**
**Reply:** We have significantly expanded the biological context in the Introduction and partially in the Conclusion. The Introduction now includes dedicated paragraphs detailing how Keap1 dysregulation drives Nrf2-mediated chemoresistance and how YEATS2 overexpression sustains oncogenic transcription in NSCLC. The revised conclusion briefly mentions the potential of modulating these pathways to restore redox balance and suppress epigenetic-driven proliferation.

**Comment 7. The manuscript does not provide a clear pathway toward experimental validation.**
**Reply:** We have added a clear and specific experimental validation framework in the Conclusion. This includes: (1) cytotoxicity and anti-proliferative assays in NSCLC cell lines (A549, H1975) alongside normal lung epithelial cells for selectivity; (2) target engagement studies (Western blotting for Nrf2 targets, H3K27ac ChIP-qPCR); (3) assessment of apoptosis and cell cycle arrest; and (4) synergy studies with standard chemotherapeutics.

**Comment 8. Methodological and Reporting Concerns: ligand selection lacks clear rationale; docking methodology insufficiently detailed; data availability statement inadequate.**
**Reply:**

- **Ligand selection rationale:** A detailed paragraph has been added to Section 2.1 explaining that the 14 compounds were systematically chosen to cover diverse chemical classes (nitrogen heterocycles, polyethers, long-chain esters, polyols, steroids, etc.) based on reported secondary metabolites from R. mucronata, ensuring a broad range of physicochemical properties.
- **Docking methodology details:** Grid box parameters are now fully provided in Table 2. We have also stated that docking protocols were validated by redocking the co-crystallized ligands, achieving RMSD < 2.0 Å.
- **Data Availability:** The statement has been updated to indicate that all input files (optimized ligand structures, protein structures, docking grids, MD input parameters) and key output data (docking poses, MD trajectories, prediction outputs) are available from the corresponding author upon reasonable request.

**Comment 9. Conclusion and Recommendation: conceptual inaccuracies, insufficient methodological transparency, and substantial overinterpretation. Major revision required.**
**Reply:** We have fully addressed all conceptual inaccuracies (target misclassification corrected), enhanced methodological transparency (detailed grid boxes, docking validation, ligand selection), and eliminated overinterpretation. The revised manuscript presents the computational findings with appropriate caution, clearly stating that the identified compounds are initial hits requiring experimental validation.

We believe that the extensive revisions we have made have substantially improved the manuscript’s scientific accuracy, clarity, and rigor. We are grateful for the reviewers’ time and expertise, which have been instrumental in strengthening this work. We hope that the revised manuscript is now suitable for publication.

Sincerely,
Ajoy Kumer
