# Supplementary material for: Investigating the anticancer potential of phytochemicals from the mangrove Rhizophora mucronata as inhibitors of lung cancer-associated proteins: Insights from multiscale computational and in silico analyses
Source: PLoS One. 2026 Oct 7;21(10):e0359525. doi: 10.1371/journal.pone.0359525 (PMC13645060; doi:10.1371/journal.pone.0359525)
Supplement: Supplementary file 10 [file pone.0359525.s010.docx]

Response to the Academic Editor and Reviewers

Manuscript ID: PONE-D-26-14113R1

Title: *Investigating the Anticancer Potential of Phytochemicals from the Mangrove Rhizophora mucronata as Inhibitors of Lung Cancer-Associated Proteins: Insights from Multiscale Computational and In Silico Analyses*

Dear Academic Editor,

We sincerely thank you and the reviewers for the careful evaluation of our revised manuscript and for the constructive comments. We greatly appreciate the time and effort devoted to assessing our work. The comments were valuable in identifying several remaining issues related to manuscript organization, scientific terminology, methodological clarity, data presentation, and interpretation.

We have carefully revised the manuscript accordingly. In particular, we consolidated the Introduction, improved the academic tone, standardized the biological nomenclature, clarified the independent AutoDock Vina and Schrödinger Glide workflows, completed the molecular dynamics methodology, corrected the DFT equations and descriptors, removed duplicated and inappropriate text, reconciled the docking results obtained from the two docking platforms, corrected the protein–ligand interaction discussion, and revised the benchmarking section to avoid inappropriate comparison of docking scores with logP.

Our point-by-point responses are provided below. The relevant section of the revised manuscript is identified after each response.

**Reviewer #1**

We sincerely thank Reviewer #1 for confirming that the previous concerns were adequately addressed and for recommending the manuscript for publication. We greatly appreciate the reviewer’s positive assessment of the revised work.

**Reviewer #2**

**Comment: Redundancy in the Introduction**

The reviewer noted that epidemiological information, survival statistics, and the limitations of existing therapies were repeated across the opening paragraphs.

**Response:**
Thank you for this observation. We agree that the earlier version contained unnecessary repetition. The Introduction has been substantially condensed and reorganized. Repeated information regarding global lung cancer mortality, five-year survival, major risk factors, and limitations of existing treatments has been consolidated. The revised Introduction now progresses more directly from the clinical burden of lung cancer to the rationale for investigating natural-product-derived compounds and finally to the selection of Keap1 and YEATS2.

**Location of correction:** Section 1, *Introduction*, opening paragraphs.

**Comment: Colloquial and non-academic expressions**

The reviewer identified several expressions that were too conversational for a scientific manuscript.

**Response:**
We agree and have revised the manuscript extensively to improve its academic tone. Conversational expressions and informal analogies were removed and replaced with concise scientific language. Statements such as “lets us compare,” “grows silently,” and other informal descriptions were rewritten using formal biomedical terminology. Similar revisions were made throughout the Methods, Results and Discussion sections.

**Location of correction:** Throughout the manuscript, particularly Sections 1, 2 and 3.

**Comment: Biological nomenclature of *Rhizophora mucronata***

The reviewer requested that the genus and species name be italicized throughout.

**Response:**
Thank you. The scientific name has been standardized throughout the revised manuscript. The full name *Rhizophora mucronata* is used at first mention where appropriate, followed by the abbreviated form *R. mucronata*. Both forms are italicized consistently.

**Location of correction:** Title, Abstract, Keywords, Introduction, Methods, Results and Discussion, Conclusion, and relevant figure/table captions.

**Comment: Semantic error in the phrase “This study recovers a critical knowledge gap”**

**Response:**
We agree. The inappropriate expression has been removed. The revised manuscript now states that the study “addresses a critical knowledge gap” rather than “recovers” a gap.

**Location of correction:** Section 1, *Introduction*, final part of the study-rationale paragraph.

**Comment: Grammatical sentence fragments in the methodology**

The reviewer identified incomplete expressions such as “Used the DMol³ module…,” “Used the Keap1…,” and “Molecular docking was did…”.

**Response:**
Thank you. These sentence fragments have been rewritten using complete formal scientific sentences and appropriate passive constructions. The DFT, receptor-preparation, AutoDock Vina, and Glide procedures are now described as complete methodological workflows.

**Location of correction:** Sections 2.1, *Optimization and Ligand Preparation*; 2.2, *Protein Preparation and Collection*; and 2.3, *Molecular Docking and Visualization*.

Comment: Contradiction concerning Discovery Studio and Schrödinger receptor preparation

The reviewer questioned whether receptors prepared only in Discovery Studio were directly used for Glide docking.

**Response:**
We thank the reviewer for highlighting this important methodological point. The receptors for AutoDock Vina and Glide were prepared independently using platform-specific workflows.

For AutoDock Vina, receptor preparation was performed using Discovery Studio, including removal of non-essential crystallographic molecules and energy minimization.

For Glide docking, the receptor structures were independently processed in Schrödinger Maestro using the Protein Preparation Wizard. This workflow included assignment of bond orders, addition of hydrogen atoms, optimization of the hydrogen-bonding network, removal of unnecessary water molecules, and restrained energy minimization. The resulting structures were subsequently used for receptor-grid generation and Glide Standard Precision docking.

This distinction is now explicitly reported in the manuscript.

**Location of correction:** Section 2.2, *Protein Preparation and Collection*, and Section 2.3.2, *Molecular Docking by Glide from Schrödinger Suite*.

**Comment: Duplicate Table 2 heading and incomplete Molecular Dynamics methodology**

**Response:**
Thank you. The duplicated Table 2 heading has been removed.

The Molecular Dynamics methodology has also been completed and expanded. The revised section now specifies the selected complexes, 100-ns simulation duration, Desmond version, OPLS-AA force field, TIP3P solvent model, orthorhombic periodic box with a 10 Å buffer, system neutralization, energy minimization, NVT and NPT equilibration, simulation temperature and pressure, thermostat and barostat, integration time step, SHAKE constraints, and the trajectory parameters evaluated.

**Location of correction:** Table 2 and Section 2.4, *Molecular Dynamics*.

**Comment: Poor formatting of Equations 4–8**

**Response:**
We agree. Equations 1–8 have been reformatted and standardized. The notation for HOMO–LUMO energy gap, ionization potential, electron affinity, electronegativity, chemical potential, hardness, softness, and electrophilicity has been corrected and presented consistently.

The equations are now defined as:

ΔEgap = ELUMO − EHOMO
I = −EHOMO
A = −ELUMO
χ = (I + A)/2
μ = −(I + A)/2
η = (I − A)/2
S = 1/η
ω = μ²/(2η)

Location of correction: Section 2.1, *Optimization and Ligand Preparation*, Equations 1–8.

**Comment: Sign and variable inconsistencies in the DFT descriptors**

The reviewer noted inconsistencies involving ionization potential, electron affinity, hardness, softness, electrophilicity, and the HOMO/LUMO values.

**Response:**
We appreciate this important observation. The DFT-derived descriptors were rechecked against the original HOMO and LUMO outputs and recalculated using the equations defined in the Methods. Table 3 has been revised accordingly so that the reported ionization potential, electron affinity, hardness, softness, chemical potential, electronegativity, and electrophilicity values are internally consistent with the underlying frontier orbital energies.

We also revised the accompanying discussion to avoid interpreting the HOMO–LUMO gap or related DFT descriptors as direct indicators of anticancer activity. These quantities are now described as supportive physicochemical descriptors of intrinsic electronic properties.

**Location of correction:** Section 3.1.2, *HOMO, LUMO, and Chemical Reactivity Descriptors*, and Table 3.

**Comment: Duplicate paragraphs in the DFT section**

**Response:**
Thank you. The duplicated text has been removed. The DFT discussion has been consolidated into a single concise interpretation emphasizing electronic stability and reactivity while avoiding overinterpretation of these descriptors as predictors of biological activity.

**Location of correction:** Section 3.1.2, immediately following Table 3.

**Comment: Use of the term “fungicidal ligands”**

**Response:**
We agree that this was an inappropriate copy-paste artifact unrelated to the present anticancer study. All references to “fungicidal ligands,” “fungicidal candidates,” and related terminology have been removed. The compounds are now consistently described as phytochemicals, screened ligands, or candidate anticancer compounds as appropriate.

**Location of correction:** Sections 3.1.3 and 3.2.1.

**Comment:** Section 3.1.4 was truncated

**Response:**
Thank you. Section 3.1.4 has been completed and revised so that the discussion of the electrostatic potential/FMO maps is presented as a complete paragraph and appropriately linked to the corresponding figures.

**Location of correction:** Section 3.1.4, *Electrostatic Potential Map for Frontier Molecular Orbital*.

**Comment:** Glide discussion duplicated the AutoDock Vina discussion

**Response:**
We agree. The duplicated AutoDock Vina material has been removed from the Glide subsection. Section 3.2.5 now discusses only the Glide-selected 1X2J–L10 and 5XNV–L02 complexes and their corresponding interaction patterns.

**Location of correction:** Section 3.2.5, *Protein–Ligand Interaction from Glide Docking*.

**Comment: Different lead ligands were identified by PyRx and Glide without explanation**

**Response:**
Thank you for raising this important issue. We have now explicitly explained why different ligand rankings were obtained from the two docking platforms.

AutoDock Vina identified L11 and L14 as the highest-ranked ligands against 1X2J and 5XNV, respectively, whereas Glide identified L10 and L02 as the highest-ranked ligands against the corresponding targets. These differences are expected because AutoDock Vina and Glide use different receptor-preparation procedures, conformational-search algorithms, scoring functions, and treatments of intermolecular interactions.

The two docking approaches are therefore interpreted as complementary rather than numerically interchangeable. To maintain methodological consistency, the Glide-ranked complexes 1X2J–L10 and 5XNV–L02 were selected for subsequent 100-ns molecular dynamics simulations.

**Location of correction:** Section 3.2.2, immediately following Table 5, and Section 3.3.1, *RMSD*.

**Comment: Contradictory description of L12 hydrogen bonding**

The reviewer noted that L12 was initially described as forming no hydrogen bonds but was subsequently described as forming several hydrogen bonds.

**Response:**
We agree and have corrected this inconsistency. According to the interaction data reported in Table 4, L12 against 5XNV forms two hydrogen bonds and four hydrophobic contacts, with a PyRx docking score of −6.0 kcal/mol. L12 is therefore now described consistently using these interaction values.

We also clarify that L12 is shown as a representative YEATS2-binding pose and was not the highest-ranked PyRx ligand for 5XNV; L14 was the highest-ranked ligand for this target in the AutoDock Vina analysis.

**Location of correction:** Section 3.2.4, *Protein–Ligand Interaction by PyRx Docking*, and Figure 6 discussion.

**Comment: Conceptual error involving docking energy and octanol–water partition coefficient**

**Response:**
We fully agree with the reviewer. The previous wording incorrectly associated docking energies expressed in kcal/mol with the octanol–water partition coefficient.

The benchmarking section has been revised to clearly distinguish docking scores from logP. Docking scores are software-dependent scoring quantities used for ranking predicted binding poses, whereas logP is a dimensionless measure of molecular lipophilicity. No direct equivalence between these quantities is now implied.

Reported Keap1 and YEATS-domain inhibitors are used primarily as qualitative structural and pharmacological references, and we explicitly state that docking scores obtained using different protocols should not be interpreted as experimentally measured binding free energies.

**Location of correction:** Section 3.2.6, *Benchmarking with Known Inhibitors*.

**Reviewer #3**

**Comment 1: The rationale for selecting the protein targets was insufficiently summarized**

**Response:**
We thank the reviewer for this important suggestion. The Introduction has been expanded to provide a concise mechanistic rationale for selecting Keap1 and YEATS2.

Keap1 was selected because it is a principal negative regulator of Nrf2, and disruption of the Keap1–Nrf2 pathway is frequently associated with NSCLC, enhanced antioxidant defense, metabolic adaptation, cell survival, and treatment resistance. YEATS2 was selected because it functions as an epigenetic reader of H3K27 acetylation and has been associated with oncogenic transcriptional regulation, proliferation, and survival in NSCLC.

The revised manuscript therefore presents Keap1 and YEATS2 as complementary targets representing redox-regulatory and epigenetic mechanisms relevant to NSCLC pathogenesis.

**Location of correction:** Section 1, *Introduction*, final two paragraphs.

**Comment 9:** The locations of the revisions were not specified

**Response:**
We apologize for this omission in the previous response. In the present rebuttal, the location of each substantive revision has been explicitly identified by manuscript section and subsection. Major revisions can be found in:

Section 1 – Introduction: consolidation of epidemiological information, formalization of academic language, and expanded rationale for Keap1 and YEATS2.

Section 2.1 – Optimization and Ligand Preparation: corrected DFT methodology and equations.

Section 2.2 – Protein Preparation and Collection: clarified independent AutoDock Vina and Schrödinger Protein Preparation Wizard workflows.

Section 2.3 – Molecular Docking: clarified AutoDock Vina and Glide protocols.

Section 2.4 – Molecular Dynamics: completed simulation methodology and parameters.

Section 3.1.2 – HOMO, LUMO, and Chemical Reactivity Descriptors: corrected DFT descriptors and revised interpretation.

Section 3.1.3–3.1.4: removed inappropriate terminology and completed FMO/electrostatic-potential discussion.

Sections 3.2.1–3.2.6 – Molecular Docking: reconciled AutoDock Vina and Glide results, corrected interaction counts, clarified ligand prioritization, and revised benchmarking.

Section 3.3 – Molecular Dynamics: clarified the rationale for selecting 1X2J–L10 and 5XNV–L02 for simulation.

We have also provided both a marked-up manuscript showing all revisions and a clean version with the revisions incorporated.

**Closing Response**

We sincerely thank the Academic Editor and all reviewers for their constructive and detailed comments. Their suggestions have substantially improved the clarity, methodological transparency, internal consistency, and scientific interpretation of the manuscript.

We have carefully revised the manuscript to ensure that computational predictions are presented conservatively and are not interpreted as direct evidence of biological efficacy. We believe that the revised manuscript now provides a clearer distinction between DFT-derived electronic descriptors, docking scores, molecular dynamics stability, and experimentally measurable biological activity.

We hope that the revised manuscript and detailed responses satisfactorily address all remaining concerns, and we respectfully submit the manuscript for further consideration for publication in PLOS ONE.

Sincerely,

Ajoy Kumer
Corresponding Author
on behalf of all authors
