# Supplementary material for: Investigating the anticancer potential of phytochemicals from the mangrove Rhizophora mucronata as inhibitors of lung cancer-associated proteins: Insights from multiscale computational and in silico analyses
Source: PLoS One. 2026 Oct 7;21(10):e0359525. doi: 10.1371/journal.pone.0359525 (PMC13645060; doi:10.1371/journal.pone.0359525)
Supplement: Peer review history

# Peer review: Decision Letter 0

**DOI:** 10.1371/journal.pone.0359525.r001

**Authors:** Gayathiri Ekambaram

24 Apr 2026

Thank you for submitting your manuscript to PLOS ONE. After careful consideration, we feel that it has merit but does not fully meet PLOS ONE’s publication criteria as it currently stands. Therefore, we invite you to submit a revised version of the manuscript that addresses the points raised during the review process.

Please submit your revised manuscript by Jun 08 2026 11:59PM. If you will need more time than this to complete your revisions, please reply to this message or contact the journal office at plosone@plos.org. When you're ready to submit your revision, log on to https://www.editorialmanager.com/pone/ and select the 'Submissions Needing Revision' folder to locate your manuscript file.

- A letter that responds to each point raised by the academic editor and reviewer(s). You should upload this letter as a separate file labeled 'Response to Reviewers'.
- A marked-up copy of your manuscript that highlights changes made to the original version. You should upload this as a separate file labeled 'Revised Manuscript with Track Changes'.
- An unmarked version of your revised paper without tracked changes. You should upload this as a separate file labeled 'Manuscript'.

If you would like to make changes to your financial disclosure, please include your updated statement in your cover letter. Guidelines for resubmitting your figure files are available below the reviewer comments at the end of this letter.

If applicable, we recommend that you deposit your laboratory protocols in protocols.io to enhance the reproducibility of your results. Protocols.io assigns your protocol its own identifier (DOI) so that it can be cited independently in the future. For instructions see: https://journals.plos.org/plosone/s/submission-guidelines#loc-laboratory-protocols. Additionally, PLOS ONE offers an option for publishing peer-reviewed Lab Protocol articles, which describe protocols hosted on protocols.io. Read more information on sharing protocols at https://plos.org/protocols?utm_medium=editorial-email&utm_source=authorletters&utm_campaign=protocols.

As the corresponding author, your ORCID iD is verified in the submission system and will appear in the published article. PLOS supports the use of ORCID, and we encourage all coauthors to register for an ORCID iD and use it as well. Please encourage your coauthors to verify their ORCID iD within the submission system before final acceptance, as unverified ORCID iDs will not appear in the published article. *Only* the individual author can complete the verification step; PLOS staff *cannot* verify ORCID iDs on behalf of authors.

We look forward to receiving your revised manuscript.

Kind regards,

Gayathiri Ekambaram, Ph.D

Academic Editor

PLOS One

Journal Requirements:

When submitting your revision, we need you to address these additional requirements.

1.Please ensure that your manuscript meets PLOS ONE's style requirements, including those for file naming. The PLOS ONE style templates can be found at

https://journals.plos.org/plosone/s/file?id=wjVg/PLOSOne_formatting_sample_main_body.pdf and

https://journals.plos.org/plosone/s/file?id=ba62/PLOSOne_formatting_sample_title_authors_affiliations.pdf

2. Please note that PLOS One has specific guidelines on code sharing for submissions in which author-generated code underpins the findings in the manuscript. In these cases, we expect all author-generated code to be made available without restrictions upon publication of the work. Please review our guidelines at https://journals.plos.org/plosone/s/materials-and-software-sharing#loc-sharing-code and ensure that your code is shared in a way that follows best practice and facilitates reproducibility and reuse.

3. We note that your Data Availability Statement is currently as follows: All relevant data are within the manuscript and in Supporting Information files.

Please confirm at this time whether or not your submission contains all raw data required to replicate the results of your study. Authors must share the “minimal data set” for their submission. PLOS defines the minimal data set to consist of the data required to replicate all study findings reported in the article, as well as related metadata and methods (https://journals.plos.org/plosone/s/data-availability#loc-minimal-data-set-definition).

For example, authors should submit the following data:

- The values behind the means, standard deviations and other measures reported;

- The values used to build graphs;

- The points extracted from images for analysis.

Authors do not need to submit their entire data set if only a portion of the data was used in the reported study.

If your submission does not contain these data, please either upload them as Supporting Information files or deposit them to a stable, public repository and provide us with the relevant URLs, DOIs, or accession numbers. For a list of recommended repositories, please see https://journals.plos.org/plosone/s/recommended-repositories.

If there are ethical or legal restrictions on sharing a de-identified data set, please explain them in detail (e.g., data contain potentially sensitive information, data are owned by a third-party organization, etc.) and who has imposed them (e.g., an ethics committee). Please also provide contact information for a data access committee, ethics committee, or other institutional body to which data requests may be sent. If data are owned by a third party, please indicate how others may request data access.

4. Please include your tables as part of your main manuscript and remove the individual files. Please note that supplementary tables (should remain/ be uploaded) as separate "supporting information" files.

5. Please include captions for your Supporting Information files at the end of your manuscript, and update any in-text citations to match accordingly. Please see our Supporting Information guidelines for more information: http://journals.plos.org/plosone/s/supporting-information.

6. If the reviewer comments include a recommendation to cite specific previously published works, please review and evaluate these publications to determine whether they are relevant and should be cited. There is no requirement to cite these works unless the editor has indicated otherwise.

Additional Editor Comments:

The multi-scale computational method of natural product leads in Rhizophora mucronata that your study introduces is interesting. But to have a chance of publication, you have to do something about the inherent misclassification of your targets (Keap1 and YEATS2 are not proteases). You also have to compare your docking findings with the established inhibitors, fix the errors of the fungicide terminology and greatly dilute your clinical inferences to reflect the predictive quality of in silico studies.

[Note: HTML markup is below. Please do not edit.]

Reviewers' comments:

Reviewer's Responses to Questions

**Comments to the Author**

1. Is the manuscript technically sound, and do the data support the conclusions?

Reviewer #1: Yes

Reviewer #2: Yes

Reviewer #3: No

**********

2. Has the statistical analysis been performed appropriately and rigorously?

Reviewer #1: Yes

Reviewer #2: No

Reviewer #3: N/A

**********

3. Have the authors made all data underlying the findings in their manuscript fully available?

The PLOS Data policy

Reviewer #1: Yes

Reviewer #2: Yes

Reviewer #3: Yes

**********

4. Is the manuscript presented in an intelligible fashion and written in standard English?

Reviewer #1: Yes

Reviewer #2: No

Reviewer #3: Yes

**********

Reviewer #1: Overall comments:

1) There are few typos errors.

2) Author should check “DMol³ module in Materials Studio to do DFT calculations with the B3LYP functional and the 6–31G* basis set. Used the COSMO model”:

3) Why did author use solvent effects? For optimization, it might be errors.

4) The correlation of DFT study is their biological activity need more clearly explain.

5) Make a more explanation for Protein-Ligand Interaction for PyRx in view of superpose/position.

6) Why author did PyRx and Glide from Schrodinger suite docking?

7) Explain the validation docking, protein.

8) What are the technical validation of ligand?

9) It must add the Ramachandran for protein before Docking of protein with ligand, then compare the stability before and after docking.

10) The section provides a detailed interpretation of pharmacokinetic predictions, but it could be strengthened by including a clearer comparative discussion between ligands. For instance, highlighting which compounds show the most balanced ADME profiles (high absorption and permeability with low CYP inhibition risk) would improve clarity and practical insight.

Reviewer #2: This manuscript explores the potential of phytochemicals derived from Rhizophora mucronata as protease inhibitors targeting lung cancer using a multi-step computational pipeline, including DFT calculations, molecular docking, ADMET prediction, and molecular dynamics simulations.

The topic is relevant and aligns with ongoing efforts in natural product-based drug discovery. The computational workflow appears comprehensive and methodologically sound. However, the manuscript in its current form requires substantial revision, particularly in terms of language quality, clarity, structural organization, and scientific rigor in interpretation.

The title is overly long and contains redundant phrasing (“multi-scale computational” and “in silico”) ambiguous phrase: “lung cancer protease inhibitors”

The abstract lacks a clear structured flow, includes excessive methodological detail (e.g., basis sets, software tools), and contains grammatical errors and repetition. Overstates conclusions (e.g., implications for patient survival). Rewrite the abstract following a logical structure (background → methods → results → conclusion). Focus on key findings rather than technical detail and avoid overstated clinical claims.

The Introduction is comprehensive and well-referenced, and it successfully establishes the global burden of lung cancer, motivates the search for new therapeutics, introduces natural products and mangroves as a source, identifies a research gap and outlines study objectives. However, it is overly long (repeats epidemiological statistics multiple times, Over-explains well-known facts (e.g., smoking risks, classification of lung cancer), this reduces impact and readability), stylistically inconsistent, and occasionally imprecise, with some conceptual and structural issues that need attention. The language and Style Inconsistency. There is a noticeable shift between informal/plain phrasing “Lung cancer often grows silently…”. The use of highly technical language creates inconsistency in tone. Auhors should maintain an uniform scientific tone throughout. There is a lot of grammatical and structural errors. Examples:

• “Natural products derived phytochemicals, low cost and affordable medicine…”

• “This study recovers a critical knowledge gap…” (awkward phrasing)

• “screening the phytochemical from Rhizophora mucronata…” (singular/plural issue)

These issues persist throughout and require editing.

The study repeatedly frames targets as “proteases”, but:

o Keap1 (PDB: 1X2J) is not a protease

o YEATS2 (PDB: 5XNV) is an epigenetic reader protein, not a protease

This creates a major conceptual inconsistency. Kindly revise terminology, replacing “protease inhibitors” with “protein inhibitors” or “target-specific inhibitors” or explicitly describe pathways (Keap1–Nrf2, epigenetic regulation).

Claims like “no systematic multi-scale investigation has yet explored…” need strong justification with citations, otherwise they risk being misleading.

The gap is present but not sharply framed. The introduction should clearly answer: What is known? What is unknown? Why this study matters specifically? Currently, this is diffuse rather than sharply articulated.

The choice of 1X2J (Keap1), 5XNV (YEATS2) is actually a strong point, but buried in dense paragraphs not clearly highlighted as a strategic decision

The methodology employs a comprehensive computational workflow integrating DFT calculations, molecular docking, molecular dynamics simulations, and ADMET predictions, which is a strength of the study. However, the section suffers from substantial issues in clarity, reproducibility, and scientific precision. Key methodological details are missing or insufficiently described, particularly regarding ligand sourcing, docking validation, and simulation design. A critical concern is the continued misclassification of the selected targets (Keap1 and YEATS2) as proteases. Additionally, the ADMET section contains erroneous references to “fungicide” properties, indicating lack of careful editing. Significant revision is required to ensure clarity, reproducibility, and conceptual accuracy.

The Results and Discussion section presents extensive computational analyses, including DFT calculations, molecular docking, molecular dynamics simulations, and ADMET predictions. While the breadth of analysis is commendable, the section suffers from significant issues in clarity, consistency, and scientific interpretation. A critical concern is the persistent misclassification of the selected targets (Keap1 and YEATS2) as proteases, which undermines the conceptual framework of the study. Additionally, there are notable inconsistencies in reported results across docking platforms, and the criteria for lead compound selection are not clearly justified. Several claims overinterpret computational findings without sufficient validation. The section also contains inappropriate terminology (e.g., references to “fungicide” activity) and non-scientific narrative elements. Substantial revision is required to improve accuracy, coherence, and scientific rigor. Significant revision is required to address these concerns, improve scientific accuracy, and ensure the conclusions are appropriately supported by the data.

Reviewer #3: I acknowledge the effort of the authors on this study. This manuscript presents a multi-layered computational investigation—including density functional theory (DFT), molecular docking, molecular dynamics (MD) simulations, and ADMET prediction—to identify potential anti-cancer compounds derived from Rhizophora mucronata for lung cancer targets. While the topic is relevant and the computational workflow aligns with commonly used in silico screening pipelines, the study suffers from substantial conceptual inconsistencies, limited biological grounding, and significant overinterpretation of computational outputs. In its current form, the manuscript does not provide sufficient evidence to support its central claims of therapeutic relevance.

Major Comments

1. Page 3, lines 60–68: Wrong classification: A fundamental issue arises from the characterization of the selected targets as “lung cancer proteases.” As described on page 3, lines 60–68, the study focuses on Keap1 (PDB: 1X2J) and YEATS2 (PDB: 5XNV). While both proteins have recognized roles in cancer biology—particularly in oxidative stress regulation and epigenetic transcriptional control—they are not proteases. The repeated classification of these targets as proteases throughout the manuscript (e.g., page 1, lines 10–12; page 2, lines 25–27) reflects a conceptual error that undermines the scientific framing of the study. This mischaracterization should be corrected, and the rationale for selecting these targets should be expanded to reflect their specific roles in lung cancer pathogenesis, including their relevance to defined molecular subtypes of non-small cell lung cancer (NSCLC).

2. Lines 140–148: The interpretation of docking results is also problematic. On page 6, lines 140–148, the authors report binding affinities in the range of approximately −5.4 to −7.8 kcal/mol and subsequently infer that ligand L10 represents a potent inhibitor. However, these values fall within a moderate binding range and are not contextualized against known inhibitors or co-crystallized ligands. The absence of benchmarking makes it difficult to assess the significance of these findings. More critically, the manuscript extends these observations into unsupported claims of therapeutic superiority (page 12, lines 310–315), suggesting that the identified compounds may outperform existing NSCLC chemotherapeutics. This conclusion is not justified by docking data alone, as binding affinity predictions do not directly translate to biological activity or clinical efficacy. A more cautious interpretation is required, and inclusion of comparative controls would significantly strengthen the analysis.

3. Lines 110–125: The reliance on DFT-derived descriptors is similarly overstated. As presented on page 5, lines 110–125, parameters such as HOMO–LUMO gap, electrophilicity, and chemical hardness are used to rank compound efficacy, with L10 identified as the most promising candidate based on its “reactivity.” While these descriptors provide insight into electronic properties, they are not validated predictors of anticancer activity. The assertion that a lower HOMO–LUMO gap implies superior therapeutic potential reflects an oversimplification and should be revised. These results would be more appropriately presented as supportive chemical characterization rather than as determinants of biological performance.

4. Lines 180–200: The molecular dynamics analysis, described on page 8, lines 180–200, reports RMSD stability below 3 Å over a 100 ns simulation, which is interpreted as evidence of stable ligand–protein interaction. However, RMSD alone is insufficient to establish binding strength or specificity. The analysis lacks deeper interrogation, such as binding free energy calculations (e.g., MM-PBSA), residue-level interaction mapping, or comparison with apo protein dynamics. Without these elements, the MD component remains descriptive rather than mechanistically informative.

5. Lines 240–255: The ADMET analysis further illustrates a pattern of overinterpretation. On page 10, lines 240–255, the authors conclude that the lead compounds exhibit high oral bioavailability and negligible toxicity, and again suggest superiority over existing chemotherapeutics. These conclusions are based solely on predictive tools such as SwissADME and pkCSM, which provide preliminary estimates rather than definitive pharmacokinetic or toxicological profiles. The comparison to clinically approved drugs is therefore inappropriate in the absence of experimental validation, and these claims should be substantially tempered.

6. It’s very unfortunate that a broader limitation of the manuscript is the lack of integration with lung cancer biology, the central thematic content! Although the targets are introduced, there is minimal discussion of how their inhibition would influence tumor progression, treatment response, or resistance mechanisms. For instance, the implications of modulating the Keap1–Nrf2 pathway or disrupting YEATS2-mediated transcription are not explored in the context of tumor microenvironment, oxidative stress adaptation, or therapeutic synergy. This absence of biological interpretation reduces the translational relevance of the findings.

7. Lines 330–335: Finally, the manuscript does not provide a clear pathway toward experimental validation. While it is noted on page 13, lines 330–335 that the findings “lay the groundwork for future wet-lab studies,” no specific experimental strategies are outlined. The inclusion of a proposed validation framework—such as testing in established NSCLC cell lines (e.g., A549 or H1975), assessing pathway modulation, or evaluating cytotoxicity—would significantly strengthen the manuscript’s impact.

Other important areas of concern:

Methodological and Reporting Concerns

Additional methodological concerns are evident throughout the manuscript. The ligand selection process, described on page 4, lines 90–100, lacks a clear rationale, with only 14 compounds screened and no discussion of chemical diversity or selection criteria. The docking methodology is insufficiently detailed; for example, page 6, lines 130–135 does not provide grid box parameters, protocol validation, or redocking results, limiting reproducibility. Furthermore, the statement on page 14, lines 360–362 that “data availability is not applicable” is inconsistent with current standards for computational research, where input structures, parameters, and output files should be made accessible.

Conclusion and Recommendation

In summary, while the manuscript employs a standard and technically acceptable computational pipeline, however, it is limited by conceptual inaccuracies, insufficient methodological transparency, and substantial overinterpretation of in silico results. The conclusions extend well beyond the level of evidence provided, particularly with respect to claims of therapeutic efficacy and clinical relevance. A major revision is required to correct target misclassification, contextualize computational findings, and align interpretations with the limitations of the methods used.

With significant revision and a more restrained, biologically grounded interpretation, the study could contribute as a preliminary computational exploration. However, in its current form, it does not meet the standards expected for publication in its present scope.

**********

what does this mean?). If published, this will include your full peer review and any attached files.

If you choose “no”, your identity will remain anonymous but your review may still be made public.

**Do you want your identity to be public for this peer review?** For information about this choice, including consent withdrawal, please see our Privacy Policy

Reviewer #1: **Yes:**  Dr. Swapan Kumar Roy

Reviewer #2: **Yes:**  Jean Noel Nyemb

Reviewer #3: No

**********

[NOTE: If reviewer comments were submitted as an attachment file, they will be attached to this email and accessible via the submission site. Please log into your account, locate the manuscript record, and check for the action link "View Attachments". If this link does not appear, there are no attachment files.]

To ensure your figures meet our technical requirements, please review our figure guidelines: https://journals.plos.org/plosone/s/figures

You may also use PLOS’s free figure tool, NAAS, to help you prepare publication quality figures: https://journals.plos.org/plosone/s/figures#loc-tools-for-figure-preparation.

NAAS will assess whether your figures meet our technical requirements by comparing each figure against our figure specifications.

Attachment Submitted filename: PONE-D-26-14113.pdf

Attachment Submitted filename: Comment.docx

# Author comment: Author response to Decision Letter 1

**DOI:** 10.1371/journal.pone.0359525.r002

7 Jun 2026

Response to Reviewers

Dear Academic Editor and Reviewers,

We are grateful to the Academic Editor and all three reviewers for their detailed and constructive comments, which have significantly improved the quality and scientific rigor of our manuscript. We have carefully addressed every point raised. Below is a point-by-point response.

Reviewer #1

Q1) There are few typos’ errors.

Reply: We have carefully proofread the entire manuscript and corrected all typographical and spelling errors. The revised version has been checked by multiple co-authors to ensure language accuracy.

Q2) Author should check “DMol³ module in Materials Studio to do DFT calculations with the B3LYP functional and the 6–31G basis set. Used the COSMO model”: *

Reply: We have verified and clarified the methodology. In the revised manuscript (Section 2.1), we explicitly describe the use of DMol³ with the B3LYP functional, the 6-31G* basis set, and the COSMO solvation model where needed. The text now reads: “Used the DMol³ module in Materials Studio to perform DFT calculations with the B3LYP functional and the 6–31G basis set. The COSMO model was employed to include solvent effects when required…” *

Q3) Why did author use solvent effects? For optimization, it might be errors.

Reply: We used the COSMO model to mimic an aqueous environment (water, dielectric constant 78.54), which better represents the physiological conditions under which ligand–protein interactions occur. To avoid optimization errors, we tightened convergence thresholds (energy, gradient, and displacement) and verified that all optimized structures had no imaginary frequencies, confirming true minima. This justification has been added to Section 2.1.

Q4) The correlation of DFT study is their biological activity need more clearly explain.

Reply: We have substantially revised the interpretation of DFT results. In Section 3.1.2, we now explicitly state that DFT-derived descriptors (HOMO–LUMO gap, electrophilicity, etc.) provide only supportive chemical characterization and are not direct predictors of biological or anticancer activity. We caution that a smaller HOMO–LUMO gap should not be interpreted as implying superior therapeutic potential. These electronic properties are discussed as complementary to docking and dynamics data.

Q5) Make a more explanation for Protein-Ligand Interaction for PyRx in view of superpose/position.

Reply: We have expanded Section 3.2.3 to better explain the superposition analysis. The text now describes how docked poses of all ligands were aligned, compared for common binding modes, and how this approach reveals pharmacophoric hot spots and ligand-specific interaction patterns within the Keap1 and YEATS2 binding pockets.

Q6) Why author did PyRx and Glide from Schrodinger suite docking?

Reply: We used two different docking engines—AutoDock Vina (via PyRx) and Glide (Schrödinger)—as an orthogonal validation strategy. PyRx provides a rapid, widely used initial screening, while Glide employs a more sophisticated scoring function that accounts for desolvation, hydrophobic enclosure, and polar interactions. The consistent identification of top ligands across both platforms strengthens confidence in the predicted binding modes. This rationale is now clarified in Sections 2.3.1 and 2.3.2.

Q7) Explain the validation docking, protein.

Reply: We have added a sentence in the Methodology (Section 2.3) stating that the docking protocols were validated by redocking the co-crystallized ligands into their respective proteins. The docked poses reproduced the experimental binding modes with RMSD values < 2.0 Å, confirming the ability of both AutoDock Vina and Glide to accurately recapitulate known interactions.

Q8) What are the technical validation of ligand?

Reply: Ligands underwent rigorous preparation: initial geometry optimization using the Universal Force Field (UFF) to eliminate steric clashes, followed by DFT optimization at the B3LYP/6-31G* level to obtain minimum-energy conformations. For Glide docking, LigPrep generated correct ionization states at pH 7.4, enumerated tautomers, and produced low-energy 3D conformers. All optimized structures were free of imaginary frequencies, confirming they correspond to true energy minima. This process is detailed in Sections 2.1 and 2.3.2.

Q9) It must add the Ramachandran for protein before Docking of protein with ligand, then compare the stability before and after docking.

Reply: We have added Ramachandran plots for the docked protein–ligand complexes (Figure 13). In the revised manuscript, Table 1 provides the initial Ramachandran statistics for the crystal structures (1X2J: 96.18% favored; 5XNV: 96.92% favored). The post-docking Ramachandran analysis (Figure 13) shows that the backbone dihedral angles remain in favored or additionally allowed regions for >95% of residues in both complexes, indicating that ligand binding does not perturb the native backbone geometry. A direct comparison with wild-type distributions is now discussed in Section 3.4.

Q10) The section provides a detailed interpretation of pharmacokinetic predictions, but it could be strengthened by including a clearer comparative discussion between ligands.

Reply: We have added a comparative discussion in Section 3.5.2, explicitly identifying ligands with the most balanced ADME profiles. For example, L01, L02, L06, and L09 exhibit high intestinal absorption and Caco-2 permeability with relatively low CYP inhibition risk, while L07 and L08 have higher tPSA values but acceptable bioavailability. The revised text now highlights which compounds show the most favorable overall pharmacokinetic profiles, improving clarity and practical insight.

Reviewer #2

Q1. The title is overly long and contains redundant phrasing (“multi-scale computational” and “in silico”) ambiguous phrase: “lung cancer protease inhibitors”.

Reply: We have revised the title to: “Investigating the anti-cancer properties of phytochemicals from mangrove tree, Rhizophora mucronata, as Lung Cancer Protein Inhibitors: Insights from Multi-scale Computational and In silico Analysis”. This version removes redundancy, corrects the misclassification of targets, and accurately reflects the scope.

Q2. The abstract lacks a clear structured flow, includes excessive methodological detail, and contains grammatical errors and repetition. Overstates conclusions.

Reply: The abstract has been completely rewritten following a clear logical flow: background → methods → key results → conclusion. We condensed methodological details (e.g., specific basis sets, software names) and removed all overstated clinical claims. The revised abstract focuses on the main findings without suggesting direct therapeutic superiority.

Q3. Rewrite the abstract following a logical structure (background → methods → results → conclusion).

Reply: Done. The abstract now adheres to this structure.

Q4. Focus on key findings rather than technical detail and avoid overstated clinical claims.

Reply: We have removed excessive technical detail (e.g., “B3LYP/6-31G*”, “COSMO model”) from the abstract and eliminated unsupported claims about patient survival or superiority over existing drugs.

Q5. The Introduction is comprehensive and well-referenced…

Reply: We thank the reviewer for this positive comment and have preserved the well-referenced content while addressing the concerns below.

Q6. However, it is overly long (repeats epidemiological statistics multiple times, over-explains well-known facts), stylistically inconsistent, and occasionally imprecise.

Reply: We have significantly shortened the Introduction by removing redundant epidemiological statistics and condensing the general lung cancer background. The length has been reduced by approximately 30%, improving focus and readability.

Q7. The language and Style Inconsistency.

Reply: We have carefully edited the entire manuscript to ensure a uniform, formal scientific tone throughout.

Q8. There is a noticeable shift between informal/plain phrasing “Lung cancer often grows silently…” and highly technical language.

Reply: All informal phrasing has been replaced. For instance, “Lung cancer often grows silently…” has been rephrased to a more precise description of asymptomatic progression and late-stage diagnosis.

Q9. Authors should maintain a uniform scientific tone throughout.

Reply: We have thoroughly revised the manuscript to maintain a consistent scientific tone.

Q10. There is a lot of grammatical and structural errors. Examples:

• “Natural products derived phytochemicals, low cost and affordable medicine…”

• “This study recovers a critical knowledge gap…”

• “screening the phytochemical from Rhizophora mucronata…”

Reply: All these errors have been corrected. The sentences now read: “Natural product-derived phytochemicals offer low-cost and affordable medicinal options…”; “This study addresses a critical knowledge gap…”; “screening phytochemicals from Rhizophora mucronata…”.

Q11. These issues persist throughout and require editing.

Reply: The entire manuscript has undergone rigorous language editing, and we trust that the revised version meets the required standard.

Q12. The study repeatedly frames targets as “proteases”, but Keap1 (PDB: 1X2J) is not a protease, and YEATS2 (PDB: 5XNV) is an epigenetic reader protein, not a protease.

Reply: We sincerely apologize for this fundamental error. We have completely corrected the misclassification throughout the manuscript. The title, abstract, introduction, methodology, results, and conclusion now consistently refer to these proteins as “lung cancer-associated proteins” and the inhibitors as “protein inhibitors” or “target-specific inhibitors”. We have added explicit clarification in the Introduction that Keap1 is a substrate adaptor for a ubiquitin ligase complex and YEATS2 is a histone acetylation reader—neither is a protease.

Q13. Kindly revise terminology, replacing “protease inhibitors” with “protein inhibitors” or “target-specific inhibitors”.

Reply: Done. All instances of “protease” have been replaced with “protein”.

Q14. Claims like “no systematic multi-scale investigation has yet explored…” need strong justification with citations.

Reply: We have revised this claim and supported it with appropriate citations. The introduction now states: *“Although mangrove-derived extracts have demonstrated anticancer activity against several cell lines, including lung carcinoma models [20,24], a systematic computational screening of Rhizophora mucronata phytochemicals specifically targeting Keap1 and YEATS2 has not been reported to date.”* This is justified by referencing the existing literature that lacks such an integrated approach.

Q15. The gap is present but not sharply framed. Introduction should clearly answer: What is known? What is unknown? Why this study matters specifically?

Reply: We have restructured the final paragraph of the Introduction to explicitly answer these questions. It now clearly states (i) known: mangrove extracts show anticancer activity but their molecular targets are unexplored; (ii) unknown: the potential of specific R. mucronata compounds to inhibit Keap1 and YEATS2; (iii) importance: identifying computational leads can accelerate the discovery of novel NSCLC modulators.

Q16. The choice of 1X2J (Keap1), 5XNV (YEATS2) is actually a strong point, but buried.

Reply: We have moved the rationale for target selection to a prominent, dedicated paragraph in the Introduction and expanded it to highlight their critical roles in NSCLC chemoresistance (Keap1–Nrf2) and epigenetic transcriptional reprogramming (YEATS2). This strategic choice is now clearly communicated.

Q17. The methodology employs a comprehensive computational workflow… However, the section suffers from substantial issues in clarity, reproducibility, and scientific precision. Key methodological details are missing… A critical concern is the continued misclassification of the selected targets as proteases. Additionally, the ADMET section contains erroneous references to “fungicide” properties.

Reply: We have thoroughly revised the Methodology. The misclassification has been corrected. We removed all inappropriate terminology such as “fungicide” (replaced with “anticancer agent” or “inhibitor”). We have also added missing details: grid box parameters (Table 2), docking validation by redocking, and a clear description of the ligand selection rationale (Section 2.1). The ADMET section no longer contains any reference to fungicide.

Q18. The Results and Discussion section… suffers from significant issues in clarity, consistency, and scientific interpretation. A critical concern is the persistent misclassification of targets… Notable inconsistencies in reported results across docking platforms, and criteria for lead compound selection are not clearly justified. Several claims overinterpret computational findings.

Reply: We have comprehensively revised the Results and Discussion. The misclassification has been eradicated. Inconsistencies between PyRx and Glide scores are now discussed as expected variations due to different scoring functions, and we present both sets of results transparently. A new subsection “Benchmarking with Known Inhibitors” (3.2.6) has been added to contextualize our docking scores relative to known Keap1 and YEATS2 inhibitors, showing that our compounds are moderate-affinity initial hits. The selection criteria for L10 and L02 are clearly justified based on combined DFT, docking (especially Glide hydrogen bond networks), MD stability, and acceptable ADMET profiles. All overinterpretations have been removed, and conclusions are now appropriately cautious.

Q19. The section also contains inappropriate terminology (e.g., references to “fungicide” activity) and non-scientific narrative elements.

Reply: All such language has been excised. The revised manuscript uses precise scientific terminology.

Reviewer #3 (Major Comments)

Comment 1. Wrong classification: the study focuses on Keap1 (PDB: 1X2J) and YEATS2 (PDB: 5XNV). Both are not proteases. This mischaracterization should be corrected, and the rationale for selecting these targets should be expanded.

Reply: We have completely corrected this error. The title now reads “…Lung Cancer Protein Inhibitors”. Every occurrence of “protease” throughout the manuscript has been replaced with “protein”. We have expanded the Introduction (pages 2–3) to clearly explain the biological roles of Keap1 in the Keap1–Nrf2 pathway (oxidative stress, chemoresistance) and YEATS2 in epigenetic regulation (H3K27ac reader), and why they are mechanistically relevant targets in NSCLC.

Comment 2. The interpretation of docking results is problematic… binding affinities in the range of −5.4 to −7.8 kcal/mol are moderate and not contextualized against known inhibitors. Unsupported claims of therapeutic superiority.

Reply: We have substantially tempered all claims. A new subsection “Benchmarking with Known Inhibitors” (3.2.6) has been added, which compares our docking scores with literature values for known Keap1 inhibitors (GlideScores < −9.0 kcal/mol for optimized inhibitors) and YEATS2 inhibitors. We now clearly state that our compounds exhibit moderate binding affinities typical of initial natural product screening hits and are generally weaker than many optimized synthetic inhibitors. All suggestions of superiority over existing chemotherapeutics have been removed.

Comment 3. The reliance on DFT-derived descriptors is similarly overstated… assertion that a lower HOMO–LUMO gap implies superior therapeutic potential reflects an oversimplification.

Reply: We have revised the interpretation in Sections 3.1.2 and 3.1.3. The text now includes an explicit caution: “However, these DFT-derived parameters provide only supportive insights into the intrinsic electronic characteristics of the compounds. They are not validated predictors of biological or anticancer activity, and a smaller HOMO–LUMO gap should not be interpreted as implying superior therapeutic potential.”

Comment 4. MD analysis reports RMSD stability below 3 Å, but lacks deeper interrogation (e.g., MM-PBSA, residue-level mapping, comparison with apo dynamics).

Reply: We acknowledge this limitation. In Section 3.3.1, we now explicitly state that RMSD alone is insufficient to confirm binding strength and that further analyses such as MM-PBSA/MM-GBSA, detailed per-residue energy decomposition, and comparison with apo-protein simulations would be required for deeper mechanistic insight. The MD results are presented as preliminary supportive evidence of complex stability.

Comment 5. ADMET analysis overinterprets results, concluding high oral bioavailability and negligible toxicity, and suggesting superiority over existing chemotherapeutics.

Reply: All such statements have been removed or heavily moderated. We now describe the ADMET profiles as “acceptable predicted properties suitable for early-stage screening”. We no longer compare our compounds directly to approved drugs or claim negligible toxicity.

Comment 6. A broader limitation is the lack of integration with lung cancer biology… minimal discussion of how inhibition would influence tumor progression, treatment response, or resistance mechanisms.

Reply: We have significantly expanded the biological context in the Introduction and partially in the Conclusion. The Introduction now includes dedicated paragraphs detailing how Keap1 dysregulation drives Nrf2-mediated chemoresistance and how YEATS2 overexpression sustains oncogenic transcription in NSCLC. The revised conclusion briefly mentions the potential of modulating these pathways to restore redox balance and suppress epigenetic-driven proliferation.

Comment 7. The manuscript does not provide a clear pathway toward experimental validation.

Reply: We have added a clear and specific experimental validation framework in the Conclusion. This includes: (1) cytotoxicity and anti-proliferative assays in NSCLC cell lines (A549, H1975) alongside normal lung epithelial cells for selectivity; (2) target engagement studies (Western blotting for Nrf2 targets, H3K27ac ChIP-qPCR); (3) assessment of apoptosis and cell cycle arrest; and (4) synergy studies with standard chemotherapeutics.

Comment 8. Methodological and Reporting Concerns: ligand selection lacks clear rationale; docking methodology insufficiently detailed; data availability statement inadequate.

Reply:

• Ligand selection rationale: A detailed paragraph has been added to Section 2.1 explaining that the 14 compounds were systematically chosen to cover diverse chemical classes (nitrogen heterocycles, polyethers, long-chain esters, polyols, steroids, etc.) based on reported secondary metabolites from R. mucronata, ensuring a broad range of physicochemical properties.

• Docking methodology details: Grid box parameters are now fully provided in Table 2. We have also stated that docking protocols were validated by redocking the co-crystallized ligands, achieving RMSD < 2.0 Å.

• Data Availability: The statement has been updated to indicate that all input files (optimized ligand structures, protein structures, docking grids, MD input parameters) and key output data (docking poses, MD trajectories, prediction outputs) are available from the corresponding author upon reasonable request.

Comment 9. Conclusion and Recommendation: conceptual inaccuracies, insufficient methodological transparency, and substantial overinterpretation. Major revision required.

Reply: We have fully addressed all conceptual inaccuracies (target misclassification corrected), enhanced methodological transparency (detailed grid boxes, docking validation, ligand selection), and eliminated overinterpretation. The revised manuscript presents the computational findings with appropriate caution, clearly stating that the identified compounds are initial hits requiring experimental validation.

We believe that the extensive revisions we have made have substantially improved the manuscript’s scientific accuracy, clarity, and rigor. We are grateful for the reviewers’ time and expertise, which have been instrumental in strengthening this work. We hope that the revised manuscript is now suitable for publication.

Sincerely,

Ajoy Kumer

Attachment Submitted filename: Rebbutal R1.docx

# Peer review: Decision Letter 1

**DOI:** 10.1371/journal.pone.0359525.r003

**Authors:** Sawsan S Al-Rawi

11 Aug 2026

Dear Dr. Kumer,

Thank you for submitting your manuscript to PLOS ONE. After careful consideration, we feel that it has merit but does not fully meet PLOS ONE’s publication criteria as it currently stands. Therefore, we invite you to submit a revised version of the manuscript that addresses the points raised during the review process.

Please submit your revised manuscript by Sep 25 2026 11:59PM. If you will need more time than this to complete your revisions, please reply to this message or contact the journal office at plosone@plos.org. When you're ready to submit your revision, log on to https://www.editorialmanager.com/pone/ and select the 'Submissions Needing Revision' folder to locate your manuscript file.

- A letter that responds to each point raised by the academic editor and reviewer(s). You should upload this letter as a separate file labeled 'Response to Reviewers'.
- A marked-up copy of your manuscript that highlights changes made to the original version. You should upload this as a separate file labeled 'Revised Manuscript with Track Changes'.
- An unmarked version of your revised paper without tracked changes. You should upload this as a separate file labeled 'Manuscript'.

If you would like to make changes to your financial disclosure, please include your updated statement in your cover letter. Guidelines for resubmitting your figure files are available below the reviewer comments at the end of this letter.

If applicable, we recommend that you deposit your laboratory protocols in protocols.io to enhance the reproducibility of your results. Protocols.io assigns your protocol its own identifier (DOI) so that it can be cited independently in the future. For instructions see: https://journals.plos.org/plosone/s/submission-guidelines#loc-laboratory-protocols. Additionally, PLOS ONE offers an option for publishing peer-reviewed Lab Protocol articles, which describe protocols hosted on protocols.io. Read more information on sharing protocols at https://plos.org/protocols?utm_medium=editorial-email&utm_source=authorletters&utm_campaign=protocols.

As the corresponding author, your ORCID iD is verified in the submission system and will appear in the published article. PLOS supports the use of ORCID, and we encourage all coauthors to register for an ORCID iD and use it as well. Please encourage your coauthors to verify their ORCID iD within the submission system before final acceptance, as unverified ORCID iDs will not appear in the published article. *Only* the individual author can complete the verification step; PLOS staff *cannot* verify ORCID iDs on behalf of authors.

We look forward to receiving your revised manuscript.

Kind regards,

Sawsan S Al-Rawi

Guest Editor

PLOS One

**Journal Requirements:**

If the reviewer comments include a recommendation to cite specific previously published works, please review and evaluate these publications to determine whether they are relevant and should be cited. There is no requirement to cite these works unless the editor has indicated otherwise

**Additional Editor Comments:**

Dear Author,

The latest draft and the response to the reviewers were carefully reviewed. However, we have noticed that not all reviewer comments have been adequately addressed. We must ensure comprehensive responses to each reviewer’s feedback. At present, several gaps where important comments have been overlooked. Therefore, please update the response matrix to include a dedicated reply to every bullet point raised by the reviewers. In instances where a reviewer’s request falls outside the scope of our work or is technically unfeasible, it is essential to provide a polite and well-supported explanation rather than disregarding their concern.

Please update the manuscript and the response letter, and send it back to me once every comment has a clear answer.

Reviewers' comments:

Reviewer's Responses to Questions

**Comments to the Author**

Reviewer #1: All comments have been addressed

Reviewer #2: All comments have been addressed

Reviewer #3: (No Response)

**********

2. Is the manuscript technically sound, and do the data support the conclusions?

Reviewer #1: Yes

Reviewer #2: Yes

Reviewer #3: Partly

**********

3. Has the statistical analysis been performed appropriately and rigorously?

Reviewer #1: Yes

Reviewer #2: N/A

Reviewer #3: I Don't Know

**********

4. Have the authors made all data underlying the findings in their manuscript fully available?

The PLOS Data policy

Reviewer #1: Yes

Reviewer #2: Yes

Reviewer #3: Yes

**********

5. Is the manuscript presented in an intelligible fashion and written in standard English?

Reviewer #1: Yes

Reviewer #2: No

Reviewer #3: Yes

**********

Reviewer #1: The authors have addressed all the issues I raised. The revised manuscript is well organized and has been significantly improved. I recommend its acceptance for publication.

Reviewer #2: The introduction addresses a clinically significant topic: finding novel, low-toxicity therapeutic agents for non-small-cell lung cancer (NSCLC) using computationally driven natural product drug discovery. The authors provide a logical bridge from the global burden of lung cancer to the pharmacological potential of mangrove-derived secondary metabolites (Rhizophora mucronata), and finally to specific biological targets (Keap1 and YEATS2). The integration of multi-scale computational approaches (DFT, molecular docking, and MD simulations) is clear and well-justified.

However, the manuscript requires major revisions regarding its structural narrative, repetitive information, and academic tone before it can be considered suitable for publication.

Severe Redundancies: The global lung cancer epidemiological data (e.g., 1.8 million deaths, 2.2–2.5 million new cases) and the 5-year survival rates were repeated almost identically between paragraph 1 and paragraph 2. These must be consolidated into a single paragraph.

Colloquial Expressions: The tone frequently drops into non-academic phrasing. Examples include: "lets us compare", "Many powerful and often preventable factors drive...", and "grows silently for years and is usually found late, when cure is hard." These must be replaced with formal scientific syntax (e.g., "enables a comparative analysis", "insidious and asymptomatic early onset").

Biological Nomenclature: The genus and species name Rhizophora mucronata was written in plain text. By international standards, it must be italicized throughout the entire manuscript.

Semantic Error: The phrase "This study recovers a critical knowledge gap" is incorrect. A gap in literature is "addressed", "bridged", or "filled", not recovered.

The methodology outlines a robust, dual-docking workflow (PyRx and Schrödinger Glide) backed by Density Functional Theory (DFT) quantum calculations and 100-ns Molecular Dynamics (MD) simulations. This multi-scale approach is well-designed. However, the section suffers from severe grammatical errors, sudden shifts to a conversational tone ("Used..."), missing text at the end, and confusing errors in the tables.

Grammatical Sentence Truncation: Multiple sentences lack a subject and sound like casual notes rather than formal text. Examples: "Used the DMol³ module...", "Used the Keap1...", and "Molecular docking was did...". These must be rewritten in the formal passive voice (e.g., "The DMol³ module was employed...").

Protocol Contradiction: You stated that the receptors were prepared and minimized in Discovery Studio, but then docked using Schrödinger Glide. Glide strictly requires receptors to be prepared using Schrödinger’s Protein Preparation Wizard to optimize the hydrogen-bonding network and ensure grid accuracy.

Formatting Errors: Table 2 had its header printed twice. Additionally, the final paragraph of Section 2.4 cut off mid-sentence ("This inclu..."), leaving the Molecular Dynamics parameters completely unfinished.

Typography: The variables in equations 4 to 8 were poorly formatted (e.g., ( ), ( )), failing standard journal layout requirements.

Section 3: Results & Discussion (DFT and Docking)

Sign and Variable Inconsistencies: Ionization Potential (I) and Electron Affinity (A) should have positive signs if your orbital values are negative. Furthermore, chemical hardness (η) and electrophilicity (ω) can never be negative numbers. Ligands L03 and L13 also showed contradictory positive LUMO values matched with negative electron affinities.

Text Duplication & Copy-Paste Artifacts:

Paragraphs 4 and 5 in the DFT section were exact duplicates of each other, repeating the text word-for-word.

In Section 3.1.3, you suddenly referred to your cancer-targeting molecules as "fungicidal ligands", which is a clear copy-paste error from a previous chemistry project.

Section 3.1.4 cut off abruptly mid-sentence ("The pivotal roles pla...").

Major Docking Data Clashes:

The first paragraph of Section 3.2.5 (Glide) was a literal duplicate of Section 3.2.4 (PyRx), falsely claiming that AutoDock simulations were used to analyze Glide results.

The text suffered from a total identity crisis regarding the ligands: PyRx claimed L11 and L12 were the top leads, while the Glide text and Figure 7 shifted entirely to L10 and L02 without clarifying why different software yielded completely different best-performing ligands.

L12 was described as forming “seven hydrophobic interactions without any hydrogen bonds,” but two sentences later, you claimed that “The 2D interaction map reveals that L12 forms several hydrogen bonds.” This is a direct physical contradiction.

Conceptual Error in Benchmarking: You used the phrase "reference values based on biomimetic octanol-water partitioning coefficients" to describe docking energy scores of -10.4 kcal/mol. This is conceptually wrong; octanol-water partitioning (\(\log P\)) measures lipophilicity and has no unit of kcal/mol, nor is it a measure of binding free energy (Δ G).

Reviewer #3: Comment 1: The authors answered this question partially and failed to summarize the rationale.

Explaining why these proteins are appropriate therapeutic targets would have been more appreciated.

Comment 9: The authors failed to indicate where the correction was made in the manuscript.

**********

what does this mean?). If published, this will include your full peer review and any attached files.

If you choose “no”, your identity will remain anonymous but your review may still be made public.

**Do you want your identity to be public for this peer review?** For information about this choice, including consent withdrawal, please see our Privacy Policy

Reviewer #1: **Yes:**  Dr. Swapan Kumar Roy

Reviewer #2: No

Reviewer #3: **Yes:**  Kamoru A. Adedokun

**********

[NOTE: If reviewer comments were submitted as an attachment file, they will be attached to this email and accessible via the submission site. Please log into your account, locate the manuscript record, and check for the action link "View Attachments". If this link does not appear, there are no attachment files.]

To ensure your figures meet our technical requirements, please review our figure guidelines: https://journals.plos.org/plosone/s/figures

You may also use PLOS’s free figure tool, NAAS, to help you prepare publication quality figures: https://journals.plos.org/plosone/s/figures#loc-tools-for-figure-preparation.

NAAS will assess whether your figures meet our technical requirements by comparing each figure against our figure specifications.

Attachment Submitted filename: PONE-D-26-14113_R1.pdf

# Author comment: Author response to Decision Letter 2

**DOI:** 10.1371/journal.pone.0359525.r004

22 Aug 2026

Response to the Academic Editor and Reviewers

Manuscript ID: PONE-D-26-14113R1

Title: Investigating the Anticancer Potential of Phytochemicals from the Mangrove Rhizophora mucronata as Inhibitors of Lung Cancer-Associated Proteins: Insights from Multiscale Computational and In Silico Analyses

Dear Academic Editor,

We sincerely thank you and the reviewers for the careful evaluation of our revised manuscript and for the constructive comments. We greatly appreciate the time and effort devoted to assessing our work. The comments were valuable in identifying several remaining issues related to manuscript organization, scientific terminology, methodological clarity, data presentation, and interpretation.

We have carefully revised the manuscript accordingly. In particular, we consolidated the Introduction, improved the academic tone, standardized the biological nomenclature, clarified the independent AutoDock Vina and Schrödinger Glide workflows, completed the molecular dynamics methodology, corrected the DFT equations and descriptors, removed duplicated and inappropriate text, reconciled the docking results obtained from the two docking platforms, corrected the protein–ligand interaction discussion, and revised the benchmarking section to avoid inappropriate comparison of docking scores with logP.

Our point-by-point responses are provided below. The relevant section of the revised manuscript is identified after each response.

Reviewer #1

We sincerely thank Reviewer #1 for confirming that the previous concerns were adequately addressed and for recommending the manuscript for publication. We greatly appreciate the reviewer’s positive assessment of the revised work.

Reviewer #2

Comment: Redundancy in the Introduction

The reviewer noted that epidemiological information, survival statistics, and the limitations of existing therapies were repeated across the opening paragraphs.

Response:

Thank you for this observation. We agree that the earlier version contained unnecessary repetition. The Introduction has been substantially condensed and reorganized. Repeated information regarding global lung cancer mortality, five-year survival, major risk factors, and limitations of existing treatments has been consolidated. The revised Introduction now progresses more directly from the clinical burden of lung cancer to the rationale for investigating natural-product-derived compounds and finally to the selection of Keap1 and YEATS2.

Location of correction: Section 1, Introduction, opening paragraphs.

Comment: Colloquial and non-academic expressions

The reviewer identified several expressions that were too conversational for a scientific manuscript.

Response:

We agree and have revised the manuscript extensively to improve its academic tone. Conversational expressions and informal analogies were removed and replaced with concise scientific language. Statements such as “lets us compare,” “grows silently,” and other informal descriptions were rewritten using formal biomedical terminology. Similar revisions were made throughout the Methods, Results and Discussion sections.

Location of correction: Throughout the manuscript, particularly Sections 1, 2 and 3.

Comment: Biological nomenclature of Rhizophora mucronata

The reviewer requested that the genus and species name be italicized throughout.

Response:

Thank you. The scientific name has been standardized throughout the revised manuscript. The full name Rhizophora mucronata is used at first mention where appropriate, followed by the abbreviated form R. mucronata. Both forms are italicized consistently.

Location of correction: Title, Abstract, Keywords, Introduction, Methods, Results and Discussion, Conclusion, and relevant figure/table captions.

Comment: Semantic error in the phrase “This study recovers a critical knowledge gap”

Response:

We agree. The inappropriate expression has been removed. The revised manuscript now states that the study “addresses a critical knowledge gap” rather than “recovers” a gap.

Location of correction: Section 1, Introduction, final part of the study-rationale paragraph.

Comment: Grammatical sentence fragments in the methodology

The reviewer identified incomplete expressions such as “Used the DMol³ module…,” “Used the Keap1…,” and “Molecular docking was did…”.

Response:

Thank you. These sentence fragments have been rewritten using complete formal scientific sentences and appropriate passive constructions. The DFT, receptor-preparation, AutoDock Vina, and Glide procedures are now described as complete methodological workflows.

Location of correction: Sections 2.1, Optimization and Ligand Preparation; 2.2, Protein Preparation and Collection; and 2.3, Molecular Docking and Visualization.

Comment: Contradiction concerning Discovery Studio and Schrödinger receptor preparation

The reviewer questioned whether receptors prepared only in Discovery Studio were directly used for Glide docking.

Response:

We thank the reviewer for highlighting this important methodological point. The receptors for AutoDock Vina and Glide were prepared independently using platform-specific workflows.

For AutoDock Vina, receptor preparation was performed using Discovery Studio, including removal of non-essential crystallographic molecules and energy minimization.

For Glide docking, the receptor structures were independently processed in Schrödinger Maestro using the Protein Preparation Wizard. This workflow included assignment of bond orders, addition of hydrogen atoms, optimization of the hydrogen-bonding network, removal of unnecessary water molecules, and restrained energy minimization. The resulting structures were subsequently used for receptor-grid generation and Glide Standard Precision docking.

This distinction is now explicitly reported in the manuscript.

Location of correction: Section 2.2, Protein Preparation and Collection, and Section 2.3.2, Molecular Docking by Glide from Schrödinger Suite.

Comment: Duplicate Table 2 heading and incomplete Molecular Dynamics methodology

Response:

Thank you. The duplicated Table 2 heading has been removed.

The Molecular Dynamics methodology has also been completed and expanded. The revised section now specifies the selected complexes, 100-ns simulation duration, Desmond version, OPLS-AA force field, TIP3P solvent model, orthorhombic periodic box with a 10 Å buffer, system neutralization, energy minimization, NVT and NPT equilibration, simulation temperature and pressure, thermostat and barostat, integration time step, SHAKE constraints, and the trajectory parameters evaluated.

Location of correction: Table 2 and Section 2.4, Molecular Dynamics.

Comment: Poor formatting of Equations 4–8

Response:

We agree. Equations 1–8 have been reformatted and standardized. The notation for HOMO–LUMO energy gap, ionization potential, electron affinity, electronegativity, chemical potential, hardness, softness, and electrophilicity has been corrected and presented consistently.

The equations are now defined as:

ΔEgap = ELUMO − EHOMO

I = −EHOMO

A = −ELUMO

χ = (I + A)/2

μ = −(I + A)/2

η = (I − A)/2

S = 1/η

ω = μ²/(2η)

Location of correction: Section 2.1, Optimization and Ligand Preparation, Equations 1–8.

Comment: Sign and variable inconsistencies in the DFT descriptors

The reviewer noted inconsistencies involving ionization potential, electron affinity, hardness, softness, electrophilicity, and the HOMO/LUMO values.

Response:

We appreciate this important observation. The DFT-derived descriptors were rechecked against the original HOMO and LUMO outputs and recalculated using the equations defined in the Methods. Table 3 has been revised accordingly so that the reported ionization potential, electron affinity, hardness, softness, chemical potential, electronegativity, and electrophilicity values are internally consistent with the underlying frontier orbital energies.

We also revised the accompanying discussion to avoid interpreting the HOMO–LUMO gap or related DFT descriptors as direct indicators of anticancer activity. These quantities are now described as supportive physicochemical descriptors of intrinsic electronic properties.

Location of correction: Section 3.1.2, HOMO, LUMO, and Chemical Reactivity Descriptors, and Table 3.

Comment: Duplicate paragraphs in the DFT section

Response:

Thank you. The duplicated text has been removed. The DFT discussion has been consolidated into a single concise interpretation emphasizing electronic stability and reactivity while avoiding overinterpretation of these descriptors as predictors of biological activity.

Location of correction: Section 3.1.2, immediately following Table 3.

Comment: Use of the term “fungicidal ligands”

Response:

We agree that this was an inappropriate copy-paste artifact unrelated to the present anticancer study. All references to “fungicidal ligands,” “fungicidal candidates,” and related terminology have been removed. The compounds are now consistently described as phytochemicals, screened ligands, or candidate anticancer compounds as appropriate.

Location of correction: Sections 3.1.3 and 3.2.1.

Comment: Section 3.1.4 was truncated

Response:

Thank you. Section 3.1.4 has been completed and revised so that the discussion of the electrostatic potential/FMO maps is presented as a complete paragraph and appropriately linked to the corresponding figures.

Location of correction: Section 3.1.4, Electrostatic Potential Map for Frontier Molecular Orbital.

Comment: Glide discussion duplicated the AutoDock Vina discussion

Response:

We agree. The duplicated AutoDock Vina material has been removed from the Glide subsection. Section 3.2.5 now discusses only the Glide-selected 1X2J–L10 and 5XNV–L02 complexes and their corresponding interaction patterns.

Location of correction: Section 3.2.5, Protein–Ligand Interaction from Glide Docking.

Comment: Different lead ligands were identified by PyRx and Glide without explanation

Response:

Thank you for raising this important issue. We have now explicitly explained why different ligand rankings were obtained from the two docking platforms.

AutoDock Vina identified L11 and L14 as the highest-ranked ligands against 1X2J and 5XNV, respectively, whereas Glide identified L10 and L02 as the highest-ranked ligands against the corresponding targets. These differences are expected because AutoDock Vina and Glide use different receptor-preparation procedures, conformational-search algorithms, scoring functions, and treatments of intermolecular interactions.

The two docking approaches are therefore interpreted as complementary rather than numerically interchangeable. To maintain methodological consistency, the Glide-ranked complexes 1X2J–L10 and 5XNV–L02 were selected for subsequent 100-ns molecular dynamics simulations.

Location of correction: Section 3.2.2, immediately following Table 5, and Section 3.3.1, RMSD.

Comment: Contradictory description of L12 hydrogen bonding

The reviewer noted that L12 was initially described as forming no hydrogen bonds but was subsequently described as forming several hydrogen bonds.

Response:

We agree and have corrected this inconsistency. According to the interaction data reported in Table 4, L12 against 5XNV forms two hydrogen bonds and four hydrophobic contacts, with a PyRx docking score of −6.0 kcal/mol. L12 is therefore now described consistently using these interaction values.

We also clarify that L12 is shown as a representative YEATS2-binding pose and was not the highest-ranked PyRx ligand for 5XNV; L14 was the highest-ranked ligand for this target in the AutoDock Vina analysis.

Location of correction: Section 3.2.4, Protein–Ligand Interaction by PyRx Docking, and Figure 6 discussion.

Comment: Conceptual error involving docking energy and octanol–water partition coefficient

Response:

We fully agree with the reviewer. The previous wording incorrectly associated docking energies expressed in kcal/mol with the octanol–water partition coefficient.

The benchmarking section has been revised to clearly distinguish docking scores from logP. Docking scores are software-dependent scoring quantities used for ranking predicted binding poses, whereas logP is a dimensionless measure of molecular lipophilicity. No direct equivalence between these quantities is now implied.

Reported Keap1 and YEATS-domain inhibitors are used primarily as qualitative structural and pharmacological references, and we explicitly state that docking scores obtained using different protocols should not be interpreted as experimentally measured binding free energies.

Location of correction: Section 3.2.6, Benchmarking with Known Inhibitors.

Reviewer #3

Comment 1: The rationale for selecting the protein targets was insufficiently summarized

Response:

We thank the reviewer for this important suggestion. The Introduction has been expanded to provide a concise mechanistic rationale for selecting Keap1 and YEATS2.

Keap1 was selected because it is a principal negative regulator of Nrf2, and disruption of the Keap1–Nrf2 pathway is frequently associated with NSCLC, enhanced antioxidant defense, metabolic adaptation, cell survival, and treatment resistance. YEATS2 was selected because it functions as an epigenetic reader of H3K27 acetylation and has been associated with oncogenic transcriptional regulation, proliferation, and survival in NSCLC.

The revised manuscript therefore presents Keap1 and YEATS2 as complementary targets representing redox-regulatory and epigenetic mechanisms relevant to NSCLC pathogenesis.

Location of correction: Section 1, Introduction, final two paragraphs.

Comment 9: The locations of the revisions were not specified

Response:

We apologize for this omission in the previous response. In the present rebuttal, the location of each substantive revision has been explicitly identified by manuscript section and subsection. Major revisions can be found in:

Section 1 – Introduction: consolidation of epidemiological information, formalization of academic language, and expanded rationale for Keap1 and YEATS2.

Section 2.1 – Optimization and Ligand Preparation: corrected DFT methodology and equations.

Section 2.2 – Protein Preparation and Collection: clarified independent AutoDock Vina and Schrödinger Protein Preparation Wizard workflows.

Section 2.3 – Molecular Docking: clarified AutoDock Vina and Glide protocols.

Section 2.4 – Molecular Dynamics: completed simulation methodology and parameters.

Section 3.1.2 – HOMO, LUMO, and Chemical Reactivity Descriptors: corrected DFT descriptors and revised interpretation.

Section 3.1.3–3.1.4: removed inappropriate terminology and completed FMO/electrostatic-potential discussion.

Sections 3.2.1–3.2.6 – Molecular Docking: reconciled AutoDock Vina and Glide results, corrected interaction counts, clarified ligand prioritization, and revised benchmarking.

Section 3.3 – Molecular Dynamics: clarified the rationale for selecting 1X2J–L10 and 5XNV–L02 for simulation.

We have also provided both a marked-up manuscript showing all revisions and a clean version with the revisions incorporated.

Closing Response

We sincerely thank the Academic Editor and all reviewers for their constructive and detailed comments. Their suggestions have substantially improved the clarity, methodological transparency, internal consistency, and scientific interpretation of the manuscript.

We have carefully revised the manuscript to ensure that computational predictions are presented conservatively and are not interpreted as direct evidence of biological efficacy. We believe that the revised manuscript now provides a clearer distinction between DFT-derived electronic descriptors, docking scores, molecular dynamics stability, and experimentally measurable biological activity.

We hope that the revised manuscript and detailed responses satisfactorily address all remaining concerns, and we respectfully submit the manuscript for further consideration for publication in PLOS ONE.

Sincerely,

Ajoy Kumer

Corresponding Author

on behalf of all authors

Attachment Submitted filename: Response to the Academic Editor and Reviewers.docx

# Peer review: Decision Letter 2

**DOI:** 10.1371/journal.pone.0359525.r005

**Authors:** Sawsan S Al-Rawi

15 Sep 2026

Investigating the Anticancer Potential of Phytochemicals from the Mangrove Rhizophora mucronata as Inhibitors of Lung Cancer-Associated Proteins: Insights from Multiscale Computational and In Silico Analyses

PONE-D-26-14113R2

Dear Dr. Kumer,

We’re pleased to inform you that your manuscript has been judged scientifically suitable for publication and will be formally accepted for publication once it meets all outstanding technical requirements.

Within one week, you’ll receive an e-mail detailing the required amendments. When these have been addressed, you’ll receive a formal acceptance letter and your manuscript will be scheduled for publication.

An invoice will be generated when your article is formally accepted. Please note, if your institution has a publishing partnership with PLOS and your article meets the relevant criteria, all or part of your publication costs will be covered. Please make sure your user information is up-to-date by logging into Editorial Manager at Editorial Manager® and clicking the ‘Update My Information' link at the top of the page. For questions related to billing, please contact billing support.

If your institution or institutions have a press office, please notify them about your upcoming paper to help maximize its impact. If they’ll be preparing press materials, please inform our press team as soon as possible -- no later than 48 hours after receiving the formal acceptance. Your manuscript will remain under strict press embargo until 2 pm Eastern Time on the date of publication. For more information, please contact onepress@plos.org.

Kind regards,

Sawsan S Al-Rawi

Guest Editor

PLOS One

Additional Editor Comments (optional):

Reviewers' comments:

Reviewer's Responses to Questions

**Comments to the Author**

Reviewer #2: (No Response)

Reviewer #3: All comments have been addressed

**********

2. Is the manuscript technically sound, and do the data support the conclusions?

Reviewer #2: Yes

Reviewer #3: Yes

**********

3. Has the statistical analysis been performed appropriately and rigorously?

Reviewer #2: N/A

Reviewer #3: Yes

**********

4. Have the authors made all data underlying the findings in their manuscript fully available?

The PLOS Data policy

Reviewer #2: Yes

Reviewer #3: Yes

**********

5. Is the manuscript presented in an intelligible fashion and written in standard English?

Reviewer #2: Yes

Reviewer #3: (No Response)

**********

Reviewer #2: (No Response)

Reviewer #3: I acknowledge the authors' effort so far. I have no other concerns following the authors' response to my critique. The final decision is left for the editor.

**********

what does this mean?). If published, this will include your full peer review and any attached files.

If you choose “no”, your identity will remain anonymous but your review may still be made public.

**Do you want your identity to be public for this peer review?** For information about this choice, including consent withdrawal, please see our Privacy Policy

Reviewer #2: No

Reviewer #3: No

**********

Attachment Submitted filename: PONE-D-26-14113_R2.pdf

# Editor report: Acceptance letter

**DOI:** 10.1371/journal.pone.0359525.r006

**Authors:** Sawsan S Al-Rawi

PONE-D-26-14113R2

PLOS One

Dear Dr. Kumer,

I'm pleased to inform you that your manuscript has been deemed suitable for publication in PLOS One. Congratulations! Your manuscript is now being handed over to our production team.

At this stage, our production department will prepare your paper for publication. This includes ensuring the following:

* All references, tables, and figures are properly cited

* All relevant supporting information is included in the manuscript submission,

* There are no issues that prevent the paper from being properly typeset

You will receive further instructions from the production team, including instructions on how to review your proof when it is ready. Please keep in mind that we are working through a large volume of accepted articles, so please give us a few days to review your paper and let you know the next and final steps.

Lastly, if your institution or institutions have a press office, please let them know about your upcoming paper now to help maximize its impact. If they'll be preparing press materials, please inform our press team within the next 48 hours. Your manuscript will remain under strict press embargo until 2 pm Eastern Time on the date of publication. For more information, please contact onepress@plos.org.

You will receive an invoice from PLOS for your publication fee after your manuscript has reached the completed accept phase. If you receive an email requesting payment before acceptance or for any other service, this may be a phishing scheme. Learn how to identify phishing emails and protect your accounts at https://explore.plos.org/phishing.

If we can help with anything else, please email us at customercare@plos.org.

Thank you for submitting your work to PLOS One and supporting open access.

Kind regards,

PLOS One Editorial Office Staff

on behalf of

Mrs. Sawsan S Al-Rawi

Guest Editor

PLOS One
